# Spatial and Temporal Trends of Global Pollination Benefit

**DOI:** 10.1371/journal.pone.0035954

**Published:** 2012-04-26

**Authors:** Sven Lautenbach, Ralf Seppelt, Juliane Liebscher, Carsten F. Dormann

**Affiliations:** 1 Department of Computational Landscape Ecology, UFZ - Helmholtz Centre for Environmental Research, Leipzig, Germany; 2 Department of Urban Planning and Real Estate Management, Institute of Geodesy and Geoinformation- IGG, University Bonn, Bonn, Germany; 3 Department for Geography, Dresden University of Technology, Dresden, Germany; 4 Biometry & Environmental System Analysis, University of Freiburg, Freiburg, Germany; University of Northampton, United Kingdom

## Abstract

Pollination is a well-studied and at the same time a threatened ecosystem service. A significant part of global crop production depends on or profits from pollination by animals. Using detailed information on global crop yields of 60 pollination dependent or profiting crops, we provide a map of global pollination benefits on a 5′ by 5′ latitude-longitude grid. The current spatial pattern of pollination benefits is only partly correlated with climate variables and the distribution of cropland. The resulting map of pollination benefits identifies hot spots of pollination benefits at sufficient detail to guide political decisions on where to protect pollination services by investing in structural diversity of land use. Additionally, we investigated the vulnerability of the national economies with respect to potential decline of pollination services as the portion of the (agricultural) economy depending on pollination benefits. While the general dependency of the agricultural economy on pollination seems to be stable from 1993 until 2009, we see increases in producer prices for pollination dependent crops, which we interpret as an early warning signal for a conflict between pollination service and other land uses at the global scale. Our spatially explicit analysis of global pollination benefit points to hot spots for the generation of pollination benefits and can serve as a base for further planning of land use, protection sites and agricultural policies for maintaining pollination services.

## Introduction

The Millennium Ecosystem Assessment [1] and follow-up projects such as “The Economics of Ecosystems and Biodiversity” (TEEB, [2]) have raised awareness of the benefits humankind obtains from ecosystems, both in the scientific community and in decision maker circles [3], [4]. However, the ecosystem services concept still faces multiple challenges regarding research needs and its usefulness for policy support [4]–[7].

Pollination is a showcase of a well-studied ecosystem service that has consistently been described as being under threat from land-use change [8]–[12]. Pollination by animals is an important service for wild plant communities [9], [13] as well as for agricultural ecosystems [14]. A large number of crops depends upon or substantially profits from pollination by domesticated honeybees as well as by wild pollinators such as wild bees, bumblebees, butterflies, hoverflies or in some cases vertebrates such as bats and birds [15]. Although the crops with the highest production volume world-wide (rice, corn and wheat) are not dependent on pollination by animals, over a third of crop production does depend on pollinators and about 75% of all crop species profit to varying degrees from animal pollination, including most vegetables, fruits and spices [15]. These pollination-dependent or -profiting crops are also important for a number of nutrients essential for human diet [16]. For particularly valuable crops, such as vanilla or cacao, manual pollination is used to substitute the natural ecosystem service. The need for manual pollination is triggered by the absence of suitable pollinators outside the plant's native range (as in the case of vanilla: [17], [18]) or by undesirable side effects of uncontrolled pollination by pollinators (for cacao: [17], [18]).

There are clear indications of a loss of wild and domestic pollinators [8]–[12] with a number of negative ecological and economic impacts such as a decline of wild plant diversity, ecosystem stability, food production and human welfare (but see [19]). The main driving factors of pollinator declines [20], [21] are the loss and fragmentation of (semi-)natural habitats [14], [22]–[28] and other anthropogenic disturbances such as increasing use of pesticides [29], [30], environmental pollution [31], the spread of pathogens [32], introduced species (alternative plant species, competitors or enemies) [28], [33]–[35] and climate change [36], [37].

The first global estimate of the economic value of pollination was provided by Costanza et al. [38] at 117·10^9^ US $. Building on the extensive review of pollination dependencies of a huge number of crops by Klein et al. [15], Gallai et al. [39] provide an methodologically improved estimate of 153·10^9^ US $. An analysis of temporal trends for crop yields from 1961 to 2006 based on FAO data revealed no indication of pollination limitation, but of an increasing pollination dependency in both the developed as well as the developing world [40]. The increasing pollination dependency led to estimates for a complete loss of pollinators in the range of 3 to 8% of the current agricultural production [41]. To compensate for yield decreases, increasing demand for agricultural land can be expected, particularly in developing countries [41]. The importance of pollination dependency was recently further highlighted by the results of Aizen & Harder [42], who showed that trends in production of pollination-dependent crops and abundance of managed honey bees – using beehive numbers as a proxy - have been decoupled since the early 1990 s. In turn, wild pollinators have become increasingly significant for compensating decreasing honeybee abundances. Results by Garibaldi et al. [43] suggest that pollinator shortage might already decrease crop yields: while average crop yields and stability of crop yields increased from 1961–2008, decreasing trends for crops which profit from pollination were detected together with increasing variability for yields for those crops - crops essentially dependent on pollination did not show decreasing yield trends or increasing variability. Increasing production quantities for pollination profiting crops could be related to increases in areas used to cultivate the crops. It thus becomes clear that pollination is far more than an ecological-economical showcase, but rather is a service of global importance threatened by land-use change and agricultural intensification [20], [21].

While previous studies focused on economic aspects, we here aim at starting to fill the research gap with respect to spatial variance of pollination services. While pollination by animals is clearly dependent on land cover configuration at the landscape scale [14], [44], [45], an analysis at the global scale allows for the identification of hot spots as well as of particularly sensitive regions. Recent studies fall short in tackling these kinds of questions at satisfying resolution, as national FAO-statistics on agriculture have been used to assess the global value of pollination at the global scale [39] as well as for larger groups of countries [41].

Our study focuses on three aspects: the analysis of temporal changes in pollination benefits and in the vulnerability of national economies on pollination benefits; second, the use of sub-national data to derive a higher resolution representation of pollination dependency; and third, the analysis of driving factors of pollination dependency. Our analysis of temporal trends with respect to pollination benefits and vulnerability indicators extends existing work of [39], [41] by applying purchasing power parities, which draw a more realistic picture of producer prices and thereby pollination benefits. Effects of climatic conditions and of cropland area on the spatial pattern of pollination benefits were tested by means of a regression model. In addition to a summary map of all pollination dependent crops we investigate the spatial pattern of some pollination dependent crops in more detail.

## Materials and Methods

We used country-specific FAO-data on production prices and production quantities for crops that depend on or profit from pollination [46] to estimate the part of agricultural production that depends on pollination by animals. To avoid the potential problems of converting currencies, we restricted our analysis to the period from 1993 to 2009 for which data on production prices were already converted to US $. We used information from the World Bank [47] to correct the production prices for inflation, choosing 2009 as reference year. We adjusted production prices for differences in purchasing power among countries using the Penn World Table [48]. Data on GDP and percentage of agricultural GDP were taken from World Bank [49]. Pollination dependencies of crops were taken from Klein et al. [15].

Equation (1) was used to estimate the global value of pollination benefits. It calculates the product of producer price *pp* (US $/ton), production quantity *pq* (ton), pollination dependency ratio of the crop *dr*, inflation correction factor *inf* and the purchasing power parity factor *ppp* (equals 1 for the USA). The sum of the product is calculated for each country *j* and for each crop *i*. The quantification consists of two parts: first, we calculate the part of the harvest, which can be attributed to pollination and second, we value that part of the harvest with producer prices.

To analyze whether trends in pollination benefits originated from trends in producer prices or from trends in production quantities we calculated pollination-weighted production quantities. Values were also corrected for inflation and purchasing power parities (cf. equation 2). The time series for pollination-weighted production quantities as well as for pollination benefits were scaled relatively to 1993, making developments directly comparable. The weighted price signal was extracted by dividing the pollination value (equation1) by the pollination-weighted production quantity (equation 2). Since we were interested in comparing trends for pollination-dependent crops with non-dependent staple crops, we calculated weighted production quantities and prices for a set of staple crops (maize, rice, wheat, rye, yams, sorghum, and taro).
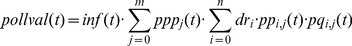
(1)

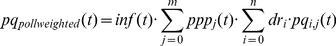
(2)

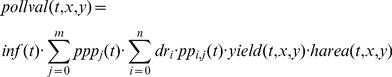
(3)To capture a part of the uncertainty involved in that calculation, we used the lower and the upper value for pollination dependency for each crop given by [15] in addition to the median value.

We quantified the vulnerability of an economy towards a decline of pollinators in two different ways: first, as the portion of pollination-dependent crops of a country's GDP and second as the dependency of the agricultural GDP on pollination services. The second vulnerability indicator, but without applying a purchasing power parities correction, has also been used by Gallai et al. [39].

We used the global maps on crop distribution of 60 pollination dependent or pollination profiting crops from Monfreda et al. [50] on a 5′ by 5′ (approx. 10 km by 10 km at the equator) latitude- longitude grid to derive a fine resolution representation of pollination benefits, based on equation (3). In contrast to equation (1), production quantities were now no longer set constant for each county, but were allowed to differ by longitude (*x*) and latitude (*y*), as well as time (*t*). Sub-national data were only available for the year 2000 so we had to restrict our analysis to that year. Data provided consists of yield information in US $ per hectare land on which the crop is cultivated as well as the percentage of the cell which is used to cultivate the crop. By multiplying both values we derived the average yield of the crop in US $ per hectare for the total area of the raster cells. Since this leads to a common reference area for all crops, these derived values can be summed over all crops. The data set offers crop yields in a mixture of national and sub-national levels. National averages for producer prices and purchasing power parities had to be used since this information was not available on sub-national levels. For a better understanding of the spatial pattern, maps of the pollination benefits of each crop were produced – a subset of seven crops will be shown here. Maps for all pollination profiting crops as well as data download options will be made available under http://geoportal.glues.geo.tu-dresden.de/geoportal/index_en.php.

We tested how far climatic conditions (mean annual temperature and mean yearly precipitation) [51] and the distribution of cropland area [52], [53] can be used to explain the spatial pattern of pollination benefits by means of a regression model. This is not intended to serve for predictions of climate change on pollination benefits, which would be highly uncertain given the complex interactions between climate, land management decisions and crop type selection involved. Instead, we aimed at describing the spatial pattern of pollination benefits using these predictors. We used trend-surface generalized additive models (GAM) [54] for that purpose. We choose GAM since we expected a non-linear relationship between pollination benefits and the predictors. The use of the coordinates as a two-dimensional smoothing term is intended to reduce model misspecification artifacts by capturing the effects of predictors not included in the model [55]. Beforehand we eliminated zero values and missing data and log-transformed the response. Calculations were performed in R [56] using the packages mgcv [54] and raster [57].

At the national scale we tested whether trends of pollination benefits were correlated with trends in agricultural production or with trends in the developments of the number of beehives. Linear models were used to calculate the trends between 1993 and 2009 for each of the countries. Beehive data were taken from FAO [46]. Results of the correlation analysis were double-checked by linear models and GAMs.

## Results

**Figure 1:**
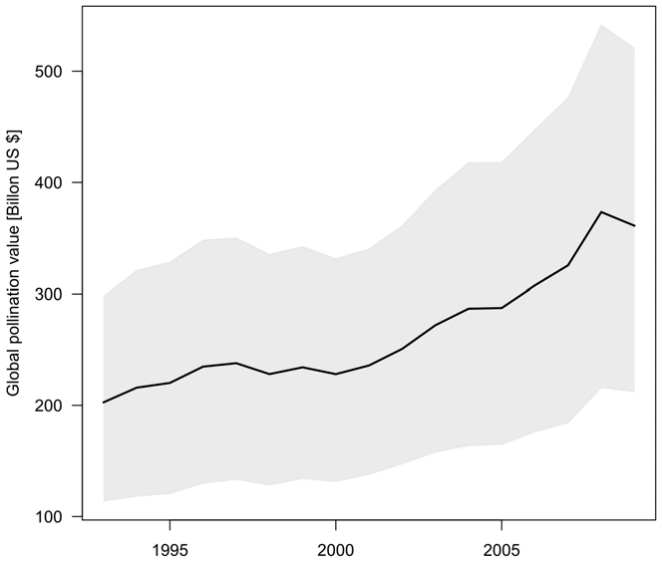
Temporal trend of global pollination benefit. Displayed are the values based on the average pollination dependency of crops (bold line) as well as on the upper and lower range of the values given by [15]. Values are in billion US $ inflation corrected for the year 2009.

Global pollination benefits show an increasing trend from 1993 to 2009 (Figure 1), consistent with previous findings [15] [40], and regardless of whether the median value of pollination dependency per crop dependency given by [15] was used or the extremes. The use of the purchasing power parities leads to increased pollination benefit estimation since it increases the value of pollination benefits in nearly all developing countries. This effect is stronger than the reduction of pollination benefit values in the majority of the developed world. As a consequence, our estimate for pollination benefits is higher than previous estimates. Compared to Gallai et al. [39], our estimate is increased by a factor of 1.9, which can be largely attributed to purchasing power parities, which was not employed there.

**Figure 2:**
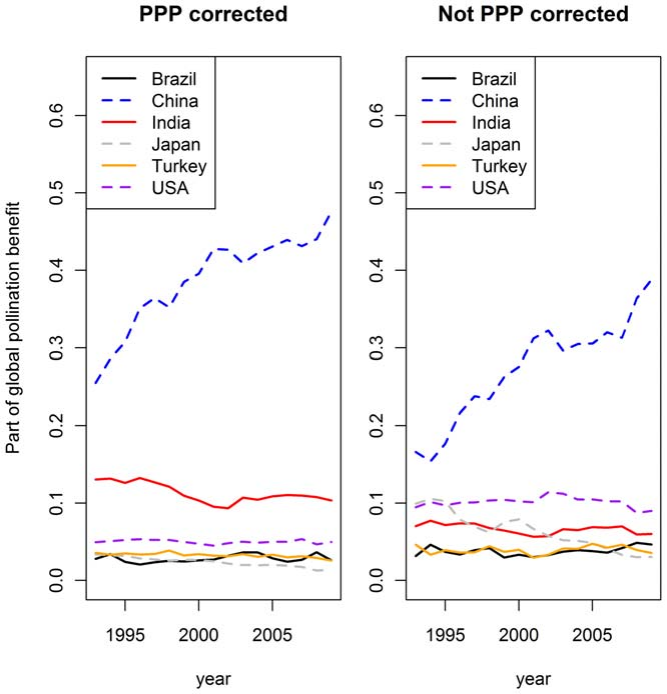
Share of the six most important countries on total pollination benefits. The left graph shows the part of global pollination benefits in each year that was produced in the different countries if the purchasing power parities correction were applied. The graph on the right side shows the same situation for the uncorrected pollination benefits.

Global pollination benefits are dominated by a small number of countries (cf. Figure 2): China is by far the most important country followed by India, the USA, Brazil, Japan and Turkey. The use of the purchasing power parities correction factor increased the importance of China and India further while the USA lost some importance.

**Figure 3:**
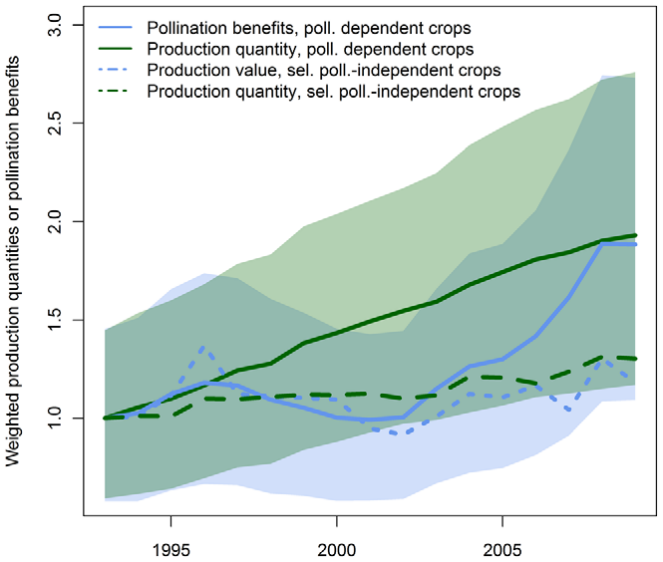
Temporal trend for pollination-weighted production quantities and pollination benefits (**equation (1)** and **2**) as well as production quantities and producer prices-weighted production quantities for selected pollination-independent crops (maize, rice, wheat, rye, yams, sorghum, and taro). For comparison all time series have been standardized to a value of 1 for 1993.

The comparison of pollination-weighted production quantities time series with pollination-benefit time series (cf. Figure 3) showed that the weighted production quantity has increased more or less steadily since 1993. The relative increase of pollination benefits was much slower between 1996 and 2001 – production quantity dominated pollination benefit estimates in that time period. Afterwards, pollination benefits increased faster and reached the same relative increase as production quantities in 2008. This implies that producer prices have globally increased stronger than production quantities. For 2009 producer prices seem to have stabilized while production quantity was still increasing.

**Figure 4:**
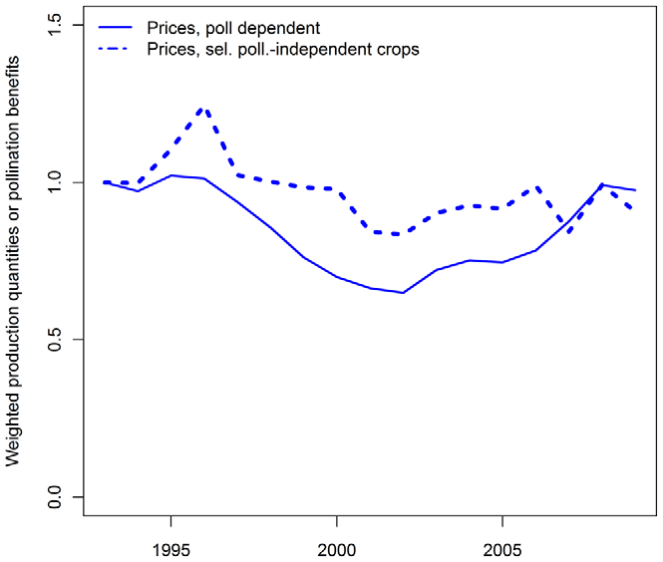
Temporal trend for prices for all pollination-dependent crops (bold line) and for selected pollination-independent crops (maize, rice, wheat, rye, yams, sorghum, taro, dashed line). All time series have been standardized to 1 for 1993.

Pollination independent crops showed a different pattern. Production quantity increased much slower than for pollination dependent or pollination profiting crops. The production value of pollination independent crops – which is comparable to pollination benefits of pollination dependent or pollination profiting crops – showed some fluctuations but generally followed the trend for production quantity more closely (cf. Figure 3). The price trend for pollination dependent and pollination profiting crops was below the price trend for pollination independent crops till 2007 (cf. Figure 4).

These trends for weighted production costs and weighted production quantities are different for the individual crops (cf. Figure S1). Soybeans, eggplants ( = aubergines), water melons, shea nuts, and rapeseed showed trends very similar to the global trend. Almonds, apples, avocados, broad beans and mustard seed indicated producer cost increasing towards the end of the time period. Blueberries show an exceptionally high increase in production costs, which is caused by high producer cost increases in the USA. Coconut, coffee and cacao beans show synchronous price fluctuations around a slightly increasing production quantity trend. Strong price fluctuations could also be observed for kola nut and vanilla. Production costs for cotton and pears develop similar to production quantities over most of the period.

**Figure 5:**
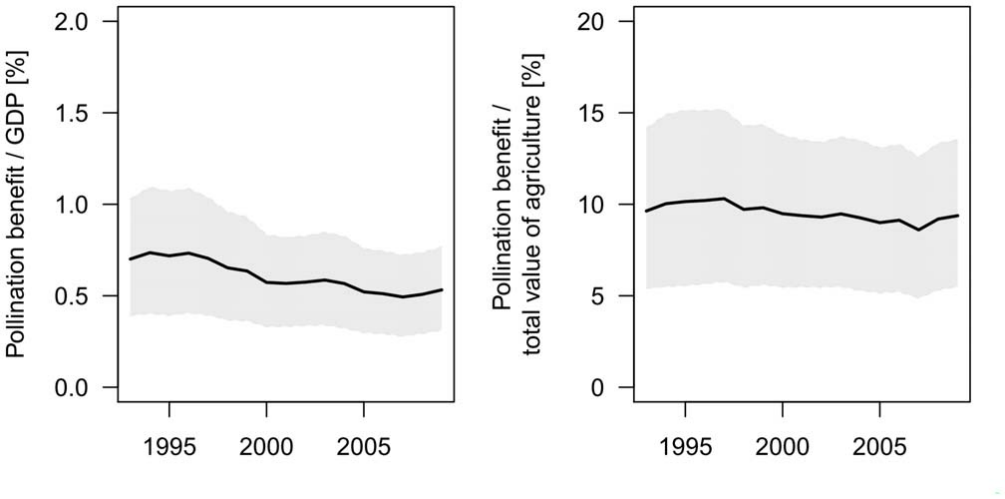
Temporal trend of vulnerability indicators. The left panel shows the development of the part of the global GDP that is dependent on pollination while the right panel shows the part of the agricultural GDP dependent on pollination.

The part of the GDP dependent on pollination (Figure 5, left panel) showed no clear indication of increase over the time period considered. For the second vulnerability indicator, the dependency of the agricultural GDP on pollination services (Figure 5, right panel) displays a slightly increasing trend over the period considered. These conclusions are again similarly supported for either the lowest and highest value of pollination dependency found in the literature [15]. These data demonstrate temporal trends of the economic importance of pollination, not whether pollination services are under threat, since the indicator focuses on the benefit side and not on the provision of the ecosystem service itself.

**Figure 6:**
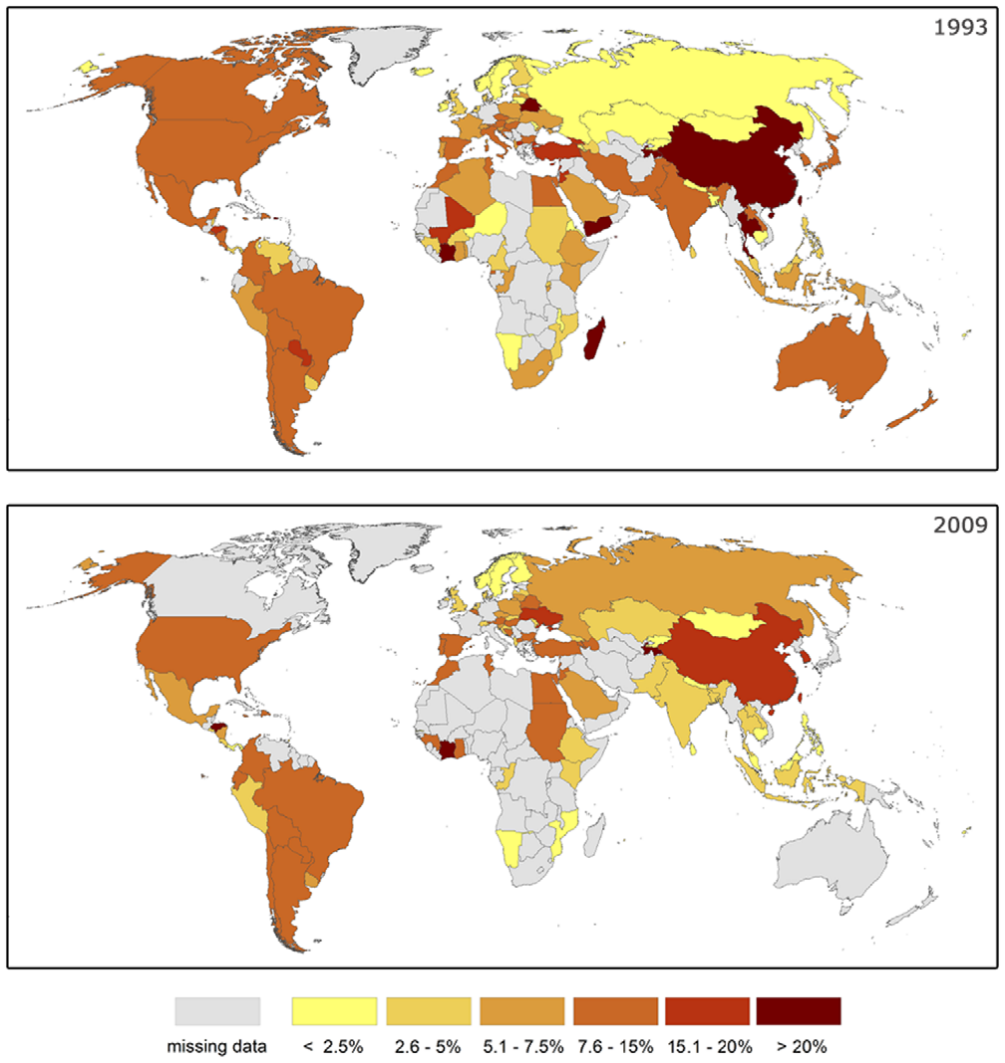
Spatial pattern of vulnerability. The maps show the national dependency of the agricultural GDP on pollination for the years 1993 and 2009 as an indicator of the vulnerability of agriculture in the different countries.

**Figure 7:**
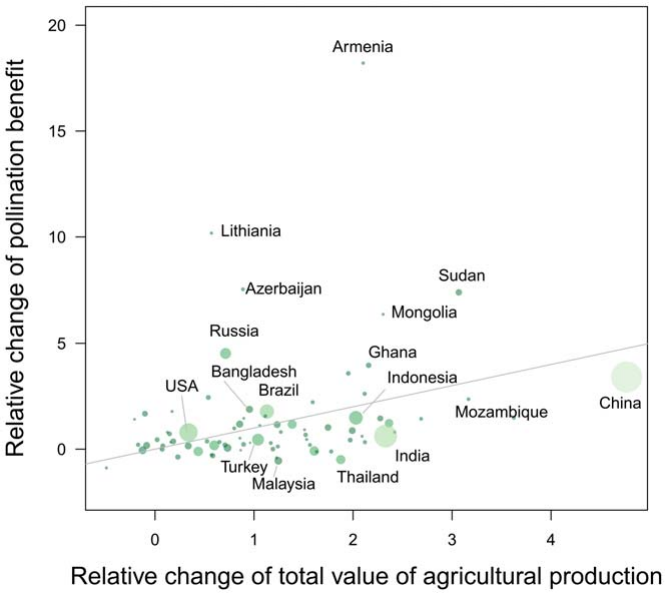
Changes in pollination benefit between 1993 and 2009 compared to changes in the agricultural GDP in the same time period. A value of one represents an increase by 100% relative to 1993. Bubble area as well as color intensity represents the size of the agricultural economy in 2009 – color intensity is inversely related to the size of the agricultural economy. The 1∶1 line (depicting proportional changes) has been added to aid interpretation. Fiji (rel. change in agricultural production = 0, relative change of pollination benefits = ∼80) has been excluded from the plot.

Results look different if considering the trends for national economies (cf. Figure 6). The spatial pattern of dependency on pollination of the agricultural part of the GDP shows high variance for both years. Countries with the highest dependency on pollination in 1993 were Côte d'Ivoire, Madagascar, Yemen, Belarus, Thailand and China. But not only countries with a low GDP show high vulnerability towards a decline in pollination: the USA, South Korea, Japan, Australia, Italy, Spain, Argentina and Brazil had relatively high dependencies of their agriculture on pollination services. Comparing the part of the agricultural GDP that depends on pollination for 1993 vs. 2009 shows a large variance compared to the global trend (9.6% in 1993 vs. 9.4 in 2009): countries like Azerbaijan (3% vs. 13.8%), the USA (8.2% vs. 11%), Russia (2% vs. 6.6%), Ghana (6.4% vs. 10%), Armenia (1.2% vs. 7.6%) have increased their pollination dependency, while China (20% vs. 15.3%), Jordan (16.7% vs. 12.8%), Egypt (11.5% vs. 7.6%), Brazil (15.9% vs. 10%), India (9.4% vs. 4.5%), Côte d'Ivoire (35.6% vs. 23%) or Turkey (16.9% vs. 12%) have decreased their vulnerability. Others such as Canada (7.7% vs. 7.6% in 2008) have stayed remained remarkable stable. The change of a country's pollination benefits is unrelated to changes of the total value of the country's agricultural production (*R^2^* = 0.07) or by the size of the agricultural economy (*R^2^* = 0.0) even after Fiji had been eliminated as a potential outlier (cf. Figure 7). Linear trends of pollination benefits from 1993 to 2009 were uncorrelated (Pearson correlation coefficient: *r* = 0.22) with linear trends in beehive numbers at the country level, even after excluding potential outliers trends (*r* = 0.05).

**Figure 8:**
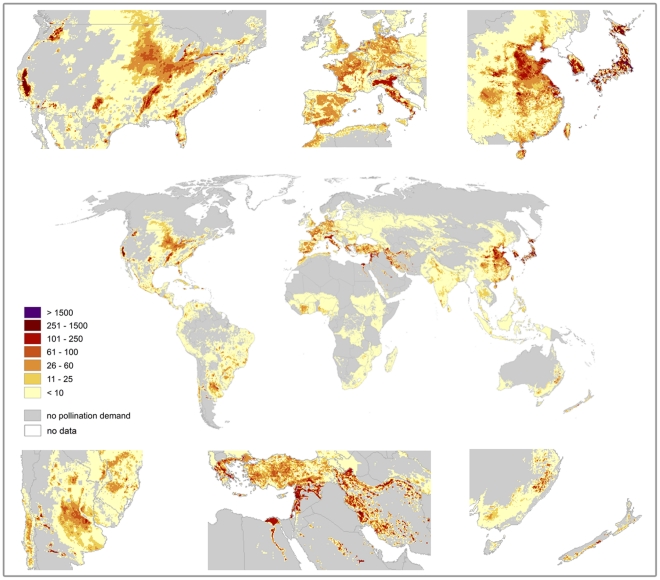
Global map of pollination benefits. Values are given as US $ per hectare for the year 2000. The values have been corrected for inflation (to the year 2009) as well as for purchasing power parities. The area we relate yields to is the total area of the raster cell.

Pollination benefits show a strong spatial pattern at the sub-national scale (Figure 8). Pollination benefits are clumped within agricultural regions – for the USA, highest values are observed in parts of California and further north along the West Coast, but pollination dependency in the Corn Belt is relatively low. Highest pollination benefits per hectare arable land in Asia can be found in Northeast China, Japan, South Korea, Taiwan, parts of India, the Levant, Turkey as well as in parts of Iran and Turkmenistan. In Europe, large parts of Italy as well as parts of Greece are exceptional. In Africa highest pollination benefits can be found along the Egyptian Nile north of Lake Nasser and in the Nile delta. With the exception of cacao production in Cote d'Ivoire, small areas in Nigeria, Tunisia and Libya, pollination benefits in the rest of Africa are low. South and Central America show some smaller peaks in southern Brazil, northern Argentina, Cuba, Jamaica and Northern Costa Rica.

Neither climatic conditions nor the amount of cropland area from Erb et al. [52] described the spatial pattern of pollination benefits satisfyingly. The cropland data from Ramankutty et al. [53] which is thematically close related to the crop distribution maps by Monfreda et al. [50] showed a better agreement with the distribution of pollination benefits. The correlation between raster cell values of cropland area from Erb et al. [52] or Ramankutty et al. [53] and pollination benefits was low (Pearson correlation coefficient *r = *0.32 or *r = *0.41). Generalized Additive Models with pollination benefits as the response and cropland area as the predictor found a significant positive effect of cropland area on pollination benefits but explained only around 20% of the variance for the data from Erb et al. – for the values from Ramankutty et al. a GAM was able to explain 52% of the variance. Climate variables (mean yearly temperature and yearly precipitation) [51] explained 29% of the variance, with a significant interaction between temperature and precipitation. A combined model of climate variables and cropland area explained 37% of the variance for the crop land area of Erb et al. and 67% for the cropland area of Ramankutty et al.

**Figure 9:**
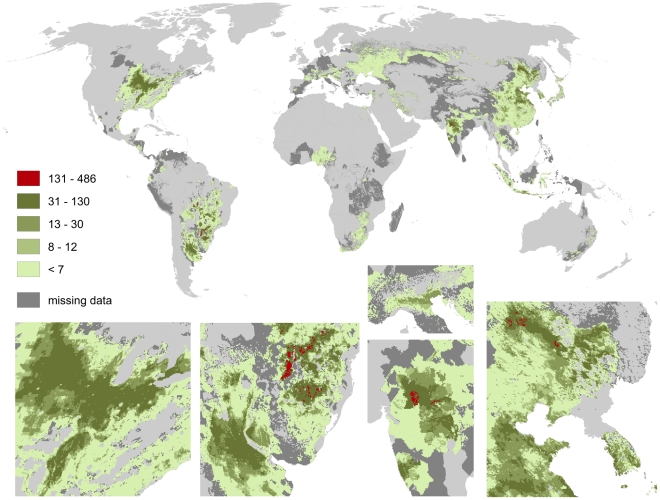
Global map of pollination benefits for soybeans. Values are given as US $ per hectare for the year 2000. The values have been corrected for inflation (to the year 2009) as well as for purchasing power parities. The area we relate yields to is the total area of the raster cell. Missing data refers to situations there yield information is available but no information on the cultivated area is available. Missing data typically occur in locations there yield per hectare agricultural is low.

Global pollination benefit is a spatial overlay of pollination benefits per crop. Distributions of the individual crops can be expected to follow climatic conditions more closely compared to the sum of all pollination profiting crops. Soybean (cf. Figure 9) is an example of a widely grown, pollination-profiting crop with relatively high impact on pollination benefits (values up to 490 US $ per hectare). Pollination benefits through soybean farming are high in southern Brazil (Paraná and Rio Grande do Sul), in the Buenos Aires-province of Argentina, in central India in the district of Madhya Prades, in the Chinese province Heilongjiang, as well as in the corn belt of the USA.

**Figure 10:**
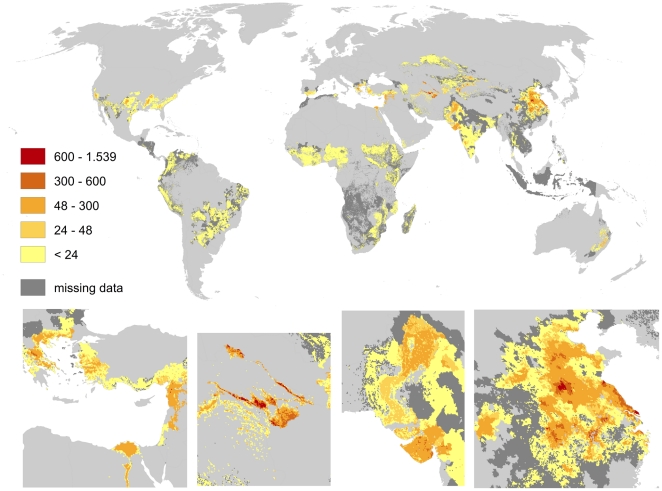
Global map of pollination benefits for cotton. Values are given as US $ per hectare for the year 2000. The values have been corrected for inflation (to the year 2009) as well as for purchasing power parities. The area we relate yields to is the total area of the raster cell. Missing data refers to situations there yield information is available but no information on the cultivated area is available. Missing data typically occur in locations there yield per hectare agricultural is low.

Pollination benefits through cotton (cf. Figure 10) show a similar widespread pattern that is generally shifted towards the Equator. Highest benefits (up to 1.500 US $ per hectare) can be identified on large scale in the Chinese provinces Jiangsu, Hubei and Shaanxi. Smaller peaks of pollination benefits by cotton can be found in Tajikistan, Xinjiang (China), Gujarat (India) and southern Queensland (Australia).

**Figure 11:**
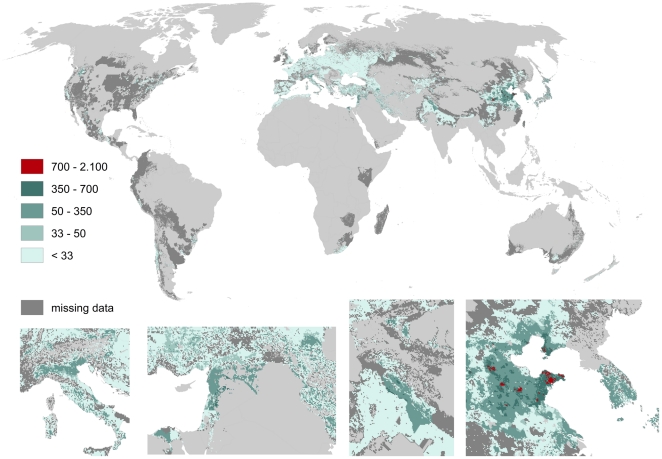
Global map of pollination benefits for apples. Values are given as US $ per hectare for the year 2000. The values have been corrected for inflation (to the year 2009) as well as for purchasing power parities. The area we relate yields to is the total area of the raster cell. Missing data refers to situations there yield information is available but no information on the cultivated area is available. Missing data typically occur in locations there yield per hectare agricultural is low.

**Figure 12:**
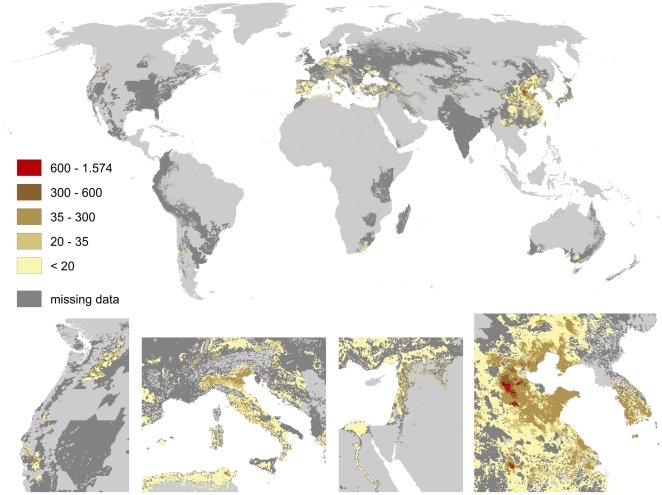
Global map of pollination benefits for pears. Values are given as US $ per hectare for the year 2000. The values have been corrected for inflation (to the year 2009) as well as for purchasing power parities. The area we relate yields to is the total area of the raster cell. Missing data refers to situations there yield information is available but no information on the cultivated area is available. Missing data typically occur in locations there yield per hectare agricultural is low.

Apples and pears show strong overlapping patterns of pollination benefits (cf. Figure 11 and Figure 12). This overlap fits well with their relatively similar optimal climatic growing conditions. Areas of high pollination benefits are small but significantly increase local pollination benefits (up to 2.000 US $/ha for apples and 1.500 US $/ha for pears). Peaks can be found in eastern Washington and southern Alberta, in the Nile delta, Syria and Lebanon, South Korea, small regions in Rio Grande do Sul (Brazil) and Argentina, as well as in large scale in the Chinese provinces Shandong, Liaoning, Hebei and Shanxi.

**Figure 13:**
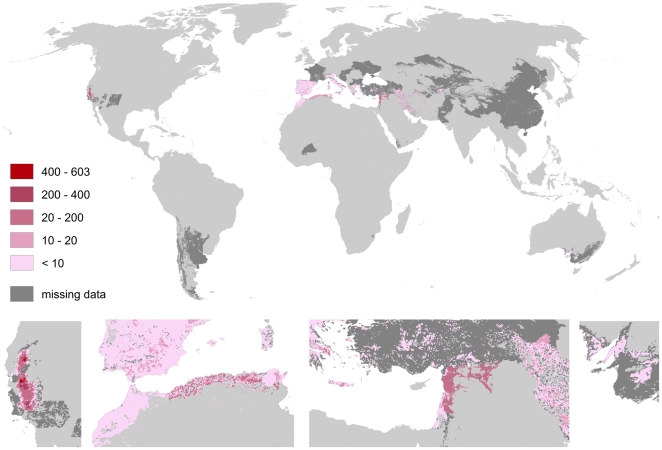
Global map of pollination benefits for almonds. Values are given as US $ per hectare for the year 2000. The values have been corrected for inflation (to the year 2009) as well as for purchasing power parities. The area we relate yields to is the total area of the raster cell. Missing data refers to situations there yield information is available but no information on the cultivated area is available. Missing data typically occur in locations there yield per hectare agricultural is low.

Almonds (cf. Figure 13) show a very narrow pattern of pollination benefits. There is a high peak (up to 600 US $/ha) in California and a secondary peak in Syria. Smaller benefits occur in southern Europe, South Australia, Iran, Turkey, Kirgizstan, and Chile as well as at the Moroccan and Libyan coast.

**Figure 14:**
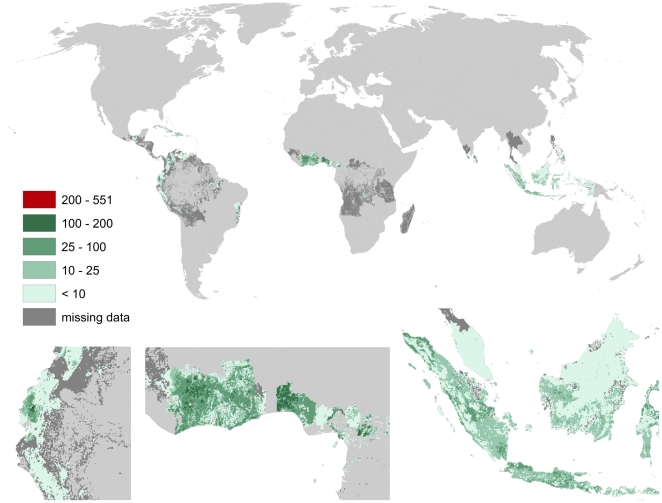
Global map of pollination benefits for cacao. Values are given as US $ per hectare for the year 2000. The values have been corrected for inflation (to the year 2009) as well as for purchasing power parities. The area we relate yields to is the total area of the raster cell. Missing data refers to situations there yield information is available but no information on the cultivated area is available. Missing data typically occur in locations there yield per hectare agricultural is low.

**Figure 15:**
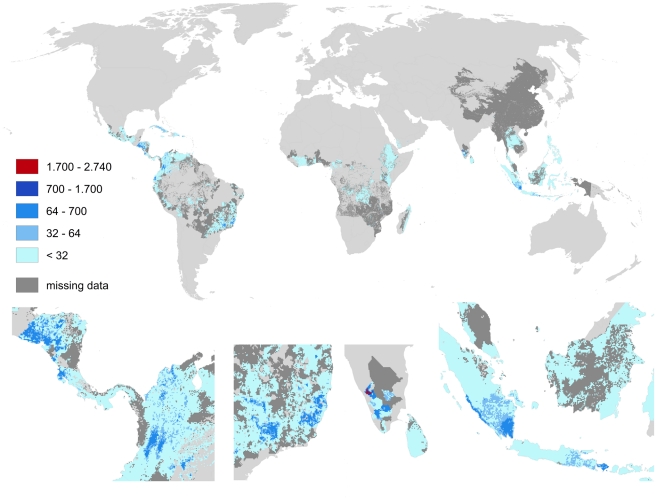
Global map of pollination benefits for coffee. Values are given as US $ per hectare for the year 2000. The values have been corrected for inflation (to the year 2009) as well as for purchasing power parities. The area we relate yields to is the total area of the raster cell. Missing data refers to situations there yield information is available but no information on the cultivated area is available. Missing data typically occur in locations there yield per hectare agricultural is low.

Both cacao and coffee are only produced in the inner tropics (cf. Figure 14 and Figure 15). Highest pollination benefits for cacao (up to 550 US $/ha) occurred in Côte d'Ivoire, Ghana, Nigeria, Cameroon, northern Ecuador, and Bahia (Brazil). Benefits occur further across Indonesia, Venezuela, Cuba, southern Mexico and the Dominican Republic. Highest pollination benefits for coffee (up to 2.000 US $/ha) occurred in Minas Gerais (Brazil), the Highland of Kenya, Honduras, El Salvador, Nicaragua as well as in southern Sumatra.

Replacing the median value of pollination dependency by the lowest (Figures S2) or the highest (Figures S3) pollination dependency reported in [15] changes the absolute values but does not change the spatial pattern. Correlation coefficients between all three responses are *r*>0.96. Without the purchasing power parities correction, pollination benefits are generally reduced in the developing countries (cf. Figure S4). Pollination benefits do tend to be higher in the middle latitudes if the correction factor is not applied.

## Discussion

We evaluated temporal trends of crop production's dependency on pollination, with a special focus on the potential vulnerability of the economies to a strong decline of wild and domesticated pollinators. The overall importance of pollination benefits for the GDP is low (around 0.5%), but probably a substantial underestimation as subsistence farming rarely contributes to the officially reported national GDPs. As expected, the analysis showed that the importance of pollination for agriculture is much higher (around 10%). As a consequence, countries where agriculture contributes substantially to GDP are especially dependent on pollination. While the general dependency of the agricultural economy on pollination seems to be stable from 1993 until 2009 at the global scale, we see increases in producer prices for pollination dependent crops, which we interpret as an early warning signal for a conflict between pollination service and other land uses at the global scale.

One should keep in mind that trends at the global scale are strongly dominated by a few countries, especially by China. A previous analysis of the pattern at the national scale showed diverse developments. Our analysis showed further, that even adjacent countries with similar economic systems such as Ghana and Côte d'Ivoire could show dissimilar trends. To aggregate trends from the country level, the analysis of clusters of countries based on the importance of agriculture, importance of pollination and trends in pollination benefits would be a next step. Compared to the analysis of the regional groups of countries by Gallai et al. [39] such a cluster analysis would have the advantage of using groups of common behavior instead of geographic regions.

While we found no indication for a global increase on pollination dependency, there are some national economies – such as the USA or Ghana - for which the trend shows an increase in the dependency of the agricultural part of the economy on pollination services. Other countries such as China or Madagascar show a decreasing dependency of their agricultural economy on pollination (Figure S5). Figure 7 shows the effect of land management decisions on pollination vulnerability: for the Russian Federation pollination benefits increased by 450%, while agriculture increased by only 71%. This increase in relative importance of pollination is mainly due to production increases for cucumbers, apples and sunflowers in the western and south-western regions of Russia. China increased its benefits from pollination by 350%, but agricultural production increased even stronger. India increased the value of agricultural production by 230%, but this increase depends only to a minor part on pollination-dependent crops. Changes in the vulnerability towards a decline of pollinators thus mainly reflect shifts in the relative importance of cultivated crops in each country, which are heavily influenced by agricultural policies, world market prices, or national political and economical developments. The agricultural developments in Eastern Europe and the successor states of the former Soviet Union as well as in China can be partly explained as effects of increasing productivity in market-based economies as well as by changes in the national demand and an increasing importance of world market prices. China has pushed fruit production enormously in the observed period of time to fulfill the demands of the increasing urban middle class as well as the demand for exports. This was clearly reflected in the increasing importance of pollination-dependent crops. Since China accounted for 30–50% of the global pollination benefits this shift in cultivated crops has significantly changed the global development as well. India as the second most important producer of pollination profiting crops has not changed its consumption and farming pattern so much. This might be due to still relatively low yields for rice and wheat, which force the country to focus on feeding the large poor rural population (http://www.ers.usda.gov/briefing/india/basicinformation.htm). Subsidies and other policy instruments have heavily influenced crop selection in the EU and the USA. In the EU for example, the biofuel directive lead to strong increases in rapeseed production. Developing countries with a high cash crop specialization such as Madagascar, Ghana or Côte d'Ivoire were affected by world market price changes for their most common produce. Increasing demand for meat in India and China increased soybean production in those countries as well as in Argentina, Brazil and the USA. Political instabilities and economic crisis effected producer prices and thereby the estimated pollination benefits – the civil war in Côte d'Ivoire or the financial crisis in Argentina are examples for that.

We here provide the first spatially explicit map of pollination demand at high resolution and identify the world's regions profiting most from pollination. As Figures 8, 9, 10, 11, 12, 13, 14, 15 clearly indicate, sub-national variance is high, e.g. for Egypt, China, India, the USA or Iran. Ignoring these patterns, focusing on the national level only, may mislead interpretations of pollination benefits. Additional variance occurs if we consider the range of pollination dependency per crop reported in the literature but this effect does not change the overall observed pattern. An increasing use of frameworks for the assessment of pollination benefits [58], [59] can be expected to decrease this uncertainties.

The quality of available global statistical datasets is variable, as becomes apparent when comparing data from different sources. FAO and World Bank statistics are not always consistent, which led to unrealistically high pollination dependencies for example for Argentina during its 2005 financial crisis or for countries with unstable internal markets, such as Tajikistan, Syria, Myanmar and Turkmenistan. The quality of the FAO data depends on the standards adopted by the reporting countries and there is no general way of checking the validity of theses values. For our analysis we assumed that introduced biases are consistent in space (sub-national level vs. national level) and time. In the absence of better data we had to rely on the FAO data.

Effects of international trade of pollination-dependent crops have not been considered in these calculations, since the FAO-statistics report import/export quantities for unprocessed as well as for processed goods. Incorporating the effects of trade can be expected to increase vulnerability in developed countries since they import significant amounts of animal pollination-supported crops such as coffee, cacao, soybeans and tropical fruits (http://faostat.fao.org/site/535/default.aspx). An analysis of trade effects similar to the water footprint [60], [61] would be an important next step in the analysis of global pollination benefits.

The sub-national data used had some shortcomings. First, the level of detail was different for the national economies – while some countries such as the USA made detailed yield data available others such as Germany reported only relatively high aggregated yield data. Second, the part of a cell used to cultivate each of the crops needs to sum up to 1. Due to the uncertainty in the reported administrative data, production areas summed over all crops might sum up to more than 100% of the area available per raster cell. The approach used by Monfreda et al [50] to distribute yield statistics to raster cells eliminates some crops for raster cells – i.e. some crops have reported yields for a raster cell but have no production unit in the raster cell. Blueberries for example do not report any production area in the USA or Poland, which are known to produce large quantities. We marked areas for which yields were reported but no production area in the maps for the single crops, but we are not able to provide such information for the sum over all crops. Therefore, total pollination benefits (cf. Figure 8) show a conservative estimate in that respect – real pollination benefits can be expected to be even higher. From a different perspective, our estimates might be too high since we used the production cost approach. Using the attributable net income method [62] would have lead to lower estimates of pollination benefits. This approach considers the fact, that yield decreases caused by sub-optimal pollination might be compensated by other production factors for crops that do not essentially depend on pollination. But since we were missing detailed information on production costs for the different production factors for the majority of the countries we had to rely on the production cost approach for the time being. The general spatial and temporal patterns of pollination benefits should stay the same for the production cost approach as well as for the attributable net income method.

Since sub-national data were only available for the year 2000 we can only make educated guesses what the spatial pattern for 2009 looks like. Given the pattern for 2000 and the trends for the different countries and the different crops (cf. Figure S6) some assumptions seem plausible. Given the strong geographic focus of the production of fruits in China together with the increasing amount of pollination dependent crops in China it can be assumed that the north-east of China would have gained even more importance. Given the increasing trend for cacao production in the countries of western Africa we can also assume that the present pattern of relatively high pollination value would have continued and increased. But we have also to keep in mind that military conflicts and civil wars might have lead to the producer price signal we see for some of the countries of West Africa. Given the high producer price increases for the former Soviet Union republics we can assume that this region gained more weight, presumably around the same locations as in 2000. But especially for some of these former Soviet Union republics like Belarus or Tajikistan we have to be aware of relatively high uncertainties in the reported FAO data. For the situation in the United States of America, it can be assumed that increases in the production value of almonds, blueberry, pears and apples have lead to increasing values in the pollination benefit peaks in California and Oregon/Washington. Increasing demand for bioenergy crops like rapeseed and canola can be assumed to have increased pollination benefits more widespread in Canada and Europe. Oil palm production as another bioenergy crop can be assumed to have taken place in Malaysia and Indonesia at cost of tropical rain forest.

The drawback of an analysis at this scale is that the supply of the service is difficult to capture, since important land-use configuration effects cannot be incorporated. We currently lack data and/or a mechanistic understanding of pollination at the landscape scale to represent it in large-scale assessments. Instead of the service supply, the benefits that people derive from animal pollination move into focus. This realized pollination benefit is a demand-side indicator that does not assess the biophysical properties of system. Therefore, it is difficult to judge whether the part of the socio-environmental system that provides pollination services is already affected by the global declines in pollinator abundances. Aizen & Harder [42] found that the number of honey beehives was increasing at the global scale but much slower than the demand for pollination expressed by the production of pollination dependent crops. This result was extended by our analysis that at the country level linear trends for pollination benefits and number of beehives were not correlated. This is in line with Breeze et al. [63] who showed for the UK that the supply of pollination services by honeybees dropped from 70% of the pollination demand in 1984 to 34% in 2007. Since the pollination supply by other managed pollinators such as bumblebees or mason bees (*Osmia* spp.) is much lower compared to the contribution of honey bees, wild pollinators had to fill the widening gap for the UK [63]. In sum, it seems that the supply of pollinator services by wild pollinators was important for global crop production and that the demand for this service is further increasing given the current agricultural trends at the global scale.

The default strategy to ensure pollination services so far focused on honeybees. Since honeybee hive numbers did not follow the increase of pollination dependent crops – neither at the global nor at national scale – and since honeybees are threatened [64] other strategies to enable pollination supply should the sought. One strategy would be the domestication of new species [65] or the breeding of specialized honeybees - but as indicated by Jaffe et al. [66], breeding activities do not compensate for the loss of wild honeybee colonies with regard to genetic diversity. Protecting wild honeybee colonies as well as honeybee breeding activities aiming at genetic diversity should be considered as an action to stabilize pollination supply for the future. Maintaining habitats for wild honeybees and other wild pollinators would probably be a win-win situation for species conservation and crop yields for pollination depending or pollination profiting crops. If the strategy of conserving pollination services is not applied it might be still possible to produce pollination dependent crops by increasing artificial pollination (e.g. [67]) or the use of other substitutes such as auxins (e.g. [68]). Hidden cost for pollination service would thereby become obvious. It is likely that costs for artificial alternatives would be much higher than maintaining the ecosystem service by clever land-use planning – especially if additional services produced by pollinator nesting habitat like recreation and biological control or biodiversity conservation are considered.

The integration of semi-natural areas into managed agricultural areas would increase pollination services by unmanaged pollinators [27], [69], [70]. Site selection could be based on the functional relationships between distance to nesting and foraging habitat by Ricketts et al. [14] – see [44], [45], [71] for example applications. In addition to unmanaged land, properly managed orchards or olive groves could build sufficient habitats for wild pollinators [72], [73]. Important aspects to consider for planning conservation efforts to ensure pollination by wild pollinators are pollinator diversity of pollinators [74] as well as redundancy in pollinator plant networks [75].

Since the availability of pollinators such as bees and hoverflies is linked to structural diversity [76] and visitation probability decreases with increasing distance to nesting habitats [14], we can assume a potential demand for structural diversity in regions with a high pollination benefit. Since the services by patches of unmanaged land have not been properly valued the potential demand is not realized. That is why an economic valuation of ecosystem services provided by patches of unmanaged land is so important. If benefits are at least roughly quantified they can be used in payments for ecosystem services schemes [77], [78] to correct incorrect market prices and to come up with cost-efficient actions in land management. A spatially explicit estimate of the pollination benefits, as presented here, delivers important information to set up such payment schemes. Results by Polasky et al. [79], [80] indicate that the integration of unmanaged land into agricultural areas can achieve conservation issues and a protection of ecosystem services at rather low economic costs. An increasing development of indicators and analysis of pollination services at the regional scale [21], [26], [27], [44], [71], [81], [82] broadened the knowledge base on the distribution of wild pollinators as well as about their contribution to pollination supply. Nevertheless, we lack large scale monitoring programs to report on the state of unmanaged pollinators.

If pollination demand by wild pollinators cannot be conserved, direct as well as indirect effects on agriculture must be expected. Rising demand for pollination services combined with a decrease of wild pollinator abundance and only slowly increasing honeybee numbers might result in decreasing yields or increasing prices for pollination services by managed honeybees – (see [83] for an example for the US). But it might also lead to a reduction of pollination-dependent farming in “pollinator-poor” nations and in term to an increase of pollination-dependent farming in countries still harboring a high abundance of pollinators. Such a shift might in turn lead to a further decrease of wild pollinator habitat if the value of the ecosystem service is not properly accounted for.

Land use planning and land management should also consider the different threads to wild pollinators. Increasing pesticide use on intensively managed areas might lead to a serious decrease of pollinators and therefore in service supply. The Egyptian Nile is a worrying example for a land-use system potentially very sensitive to a pollinator decline. If the rather diverse agricultural ecosystems in the Nile floodplain continue to be damaged by increasing pollution through increased pesticide use and through land clearing projects, local food production as well as cash crop production will be under serious thread given the high pollination benefits produced in the floodplains.

We expect the global map of pollination benefit to aid focusing the science of ecosystem services by pointing to hot spots for the generation of pollination benefits as well as for countries with a high vulnerability towards a decline in pollination service supply. Given the monetary value of the pollination benefit, decision makers should be able to compare costs and benefits for agricultural policies aiming at structural diversity. Therefore, the information provided in the map should be used when considering modifications of agricultural policies such as the common agricultural policy in the EU. Policy instruments should reflect location-specific information on tradeoffs between different management actions and land-use intensities. Pollination benefits as reported in Figure 8 are a valuable input in such a tradeoff analysis. The benefit from pollination is high enough in a large part of the world to seriously affect conservation strategies and land-use decisions if these values were taken into account. Implications reach from projects working with traditional local farmers to provide a sustainable livelihood (see e.g. [84]) to promoting pollinator restoration and conservation across the world.

## Supporting Information

Figure S1 **Temporal trend for pollination-weighted production quantities and pollination benefits (** **equation (1)** **and** **2** **) and price trends per country.** In addition, production quantities and producer prices-weighted production quantities for selected pollination-independent crops (maize, rice, wheat, rye, yams, sorghum, taro) are shown country. For comparison all time series have been standardized to a value of 1 for 1993.
(PDF)

Figure S2 **Lower bound of pollination benefits.** Values are given as US $ for the year 2000. The values have been corrected for inflation (to the year 2009) as well as for purchasing power parities. The area we relate yields to is the total area of the raster cell.
(PDF)

Figure S3 **Upper bound of pollination benefits.** Values are given as US $ for the year 2000. The values have been corrected for inflation (to the year 2009) as well as for purchasing power parities. The area we relate yields to is the total area of the raster cell.
(PDF)

Figure S4 **Global map of pollination benefits.** Values are given as US $ per hectare for the year 2000. The values have been corrected for inflation (to the year 2009) as but not for purchasing power parities. The area we relate yields to is the total area of the raster cell.
(PDF)

Figure S5 **Temporal trends of the vulnerability indicator for individual countries.** The 80 countries with the highest average part of the agricultural GDP that depends on pollination benefits have been selected for display. Values above 100% indicate incompatibilities between FAO and World Bank data.
(PDF)

Figure S6 **Trends for pollination benefits per crop for the 10 most important producing countries.**
(PDF)
