# Supplementary material for: Spatial and Temporal Trends of Global Pollination Benefit
Source: PLoS One. 2012 Apr 26;7(4):e35954. doi: 10.1371/journal.pone.0035954 (PMC3338563; doi:10.1371/journal.pone.0035954)
Supplement: Figure S1 — Temporal trend for pollination-weighted production quantities and pollination benefits ( equation (1) and 2 ) and price trends per country. In addition, production quantities and producer prices-weighted production quantities for selected pollination-independent crops (maize, rice, wheat, rye, yams, sorghum, taro) are shown country. For comparison all time series have been standardized to a value of 1 for 1993. (PDF) [file pone.0035954.s001.pdf]

Figure S1. Temporal trend for pollination-weighted production quantities and pollination benefits (equation (1) and 2) and price trends per country. In addition, production quantities and producer prices-weighted production quantities for selected pollination-independent crops (maize, rice, wheat, rye, yams, sorghum, taro) are shown country. For comparison all time series have been standardized to a value of 1 for 1993.

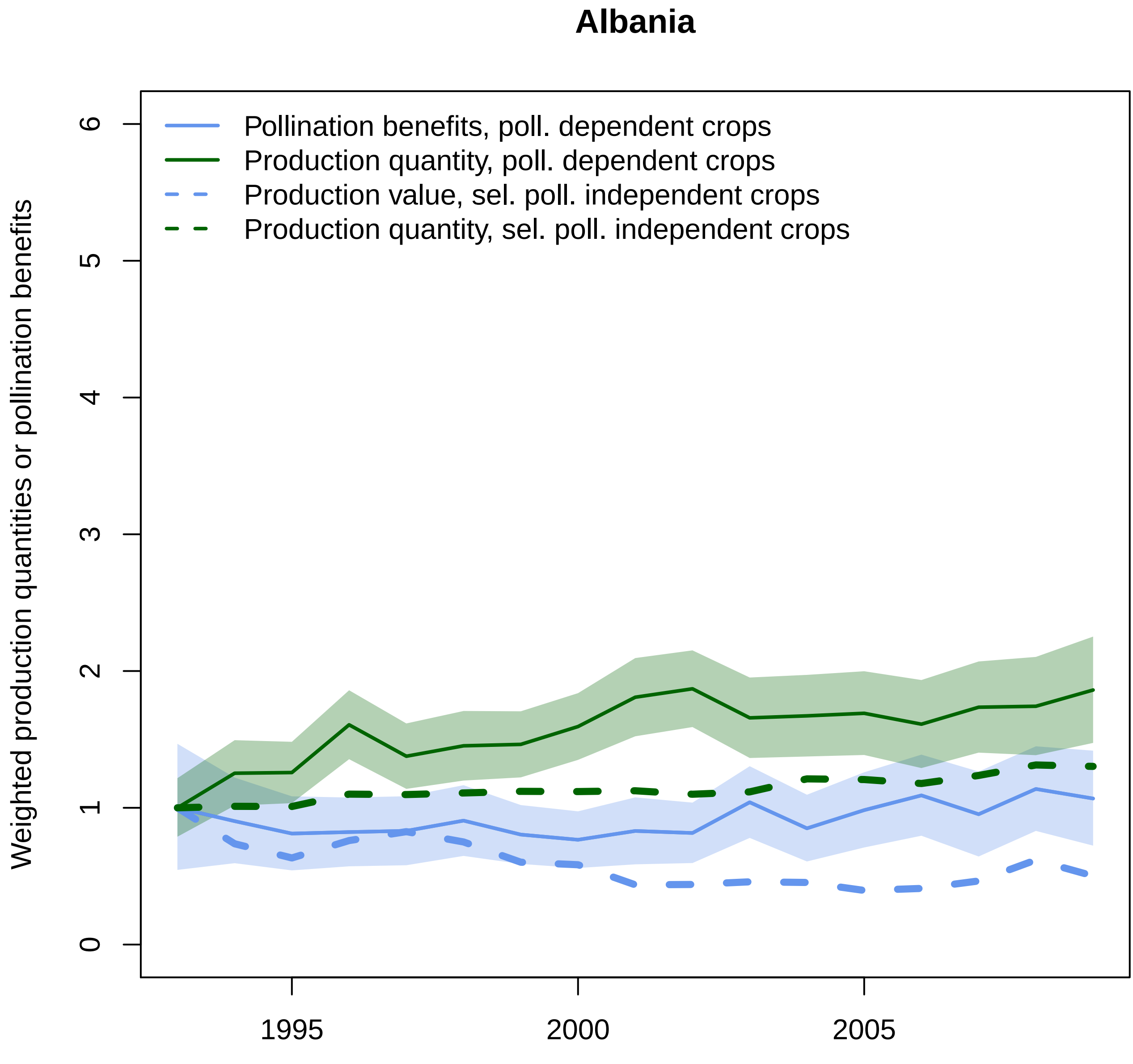
Albania
Pollination benefits, poll. dependent crops
Production quantity, poll. dependent crops
Production value, sel. poll. independent crops
Production quantity, sel. poll. independent crops
Weighted production quantities or pollination benefits
0
1
2
3
4
5
6
1995
2000
2005

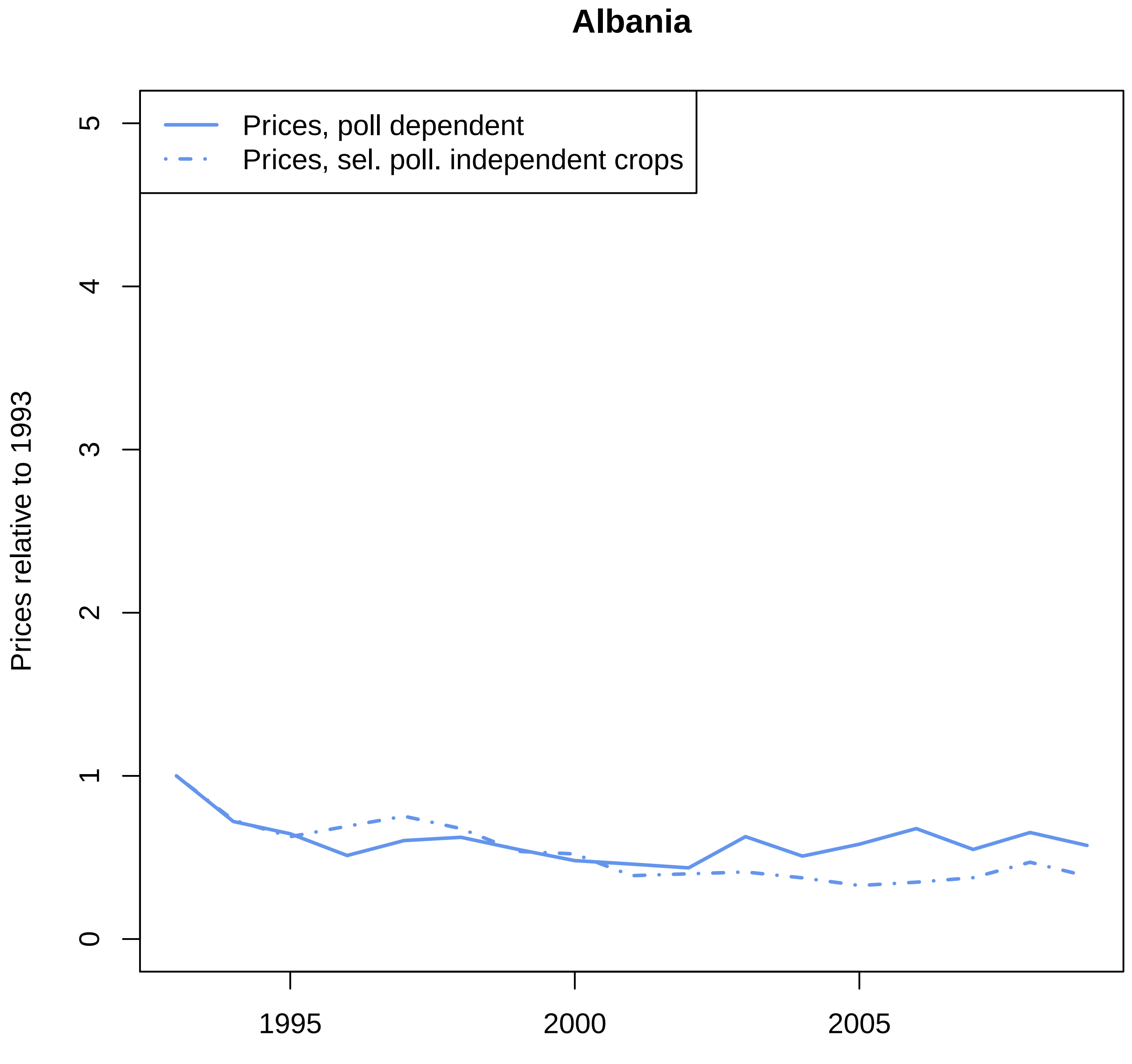

Albania
Prices, poll dependent
Prices, sel. poll. independent crops
Prices relative to 1993
0
1
2
3
4
5
1995
2000
2005

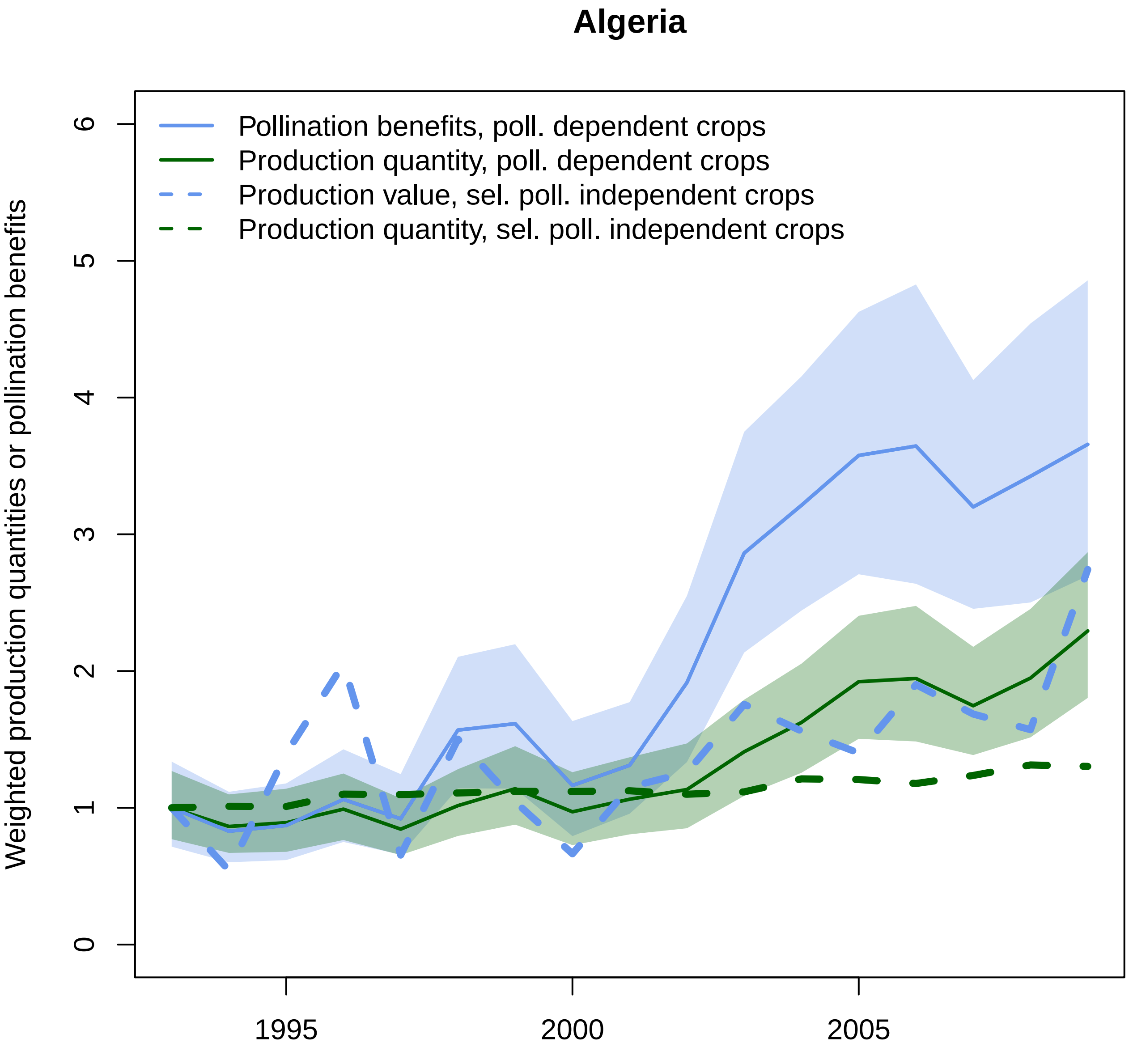
Algeria
Pollination benefits, poll. dependent crops
Production quantity, poll. dependent crops
Production value, sel. poll. independent crops
Production quantity, sel. poll. independent crops
Weighted production quantities or pollination benefits
0
1
2
3
4
5
6
1995
2000
2005

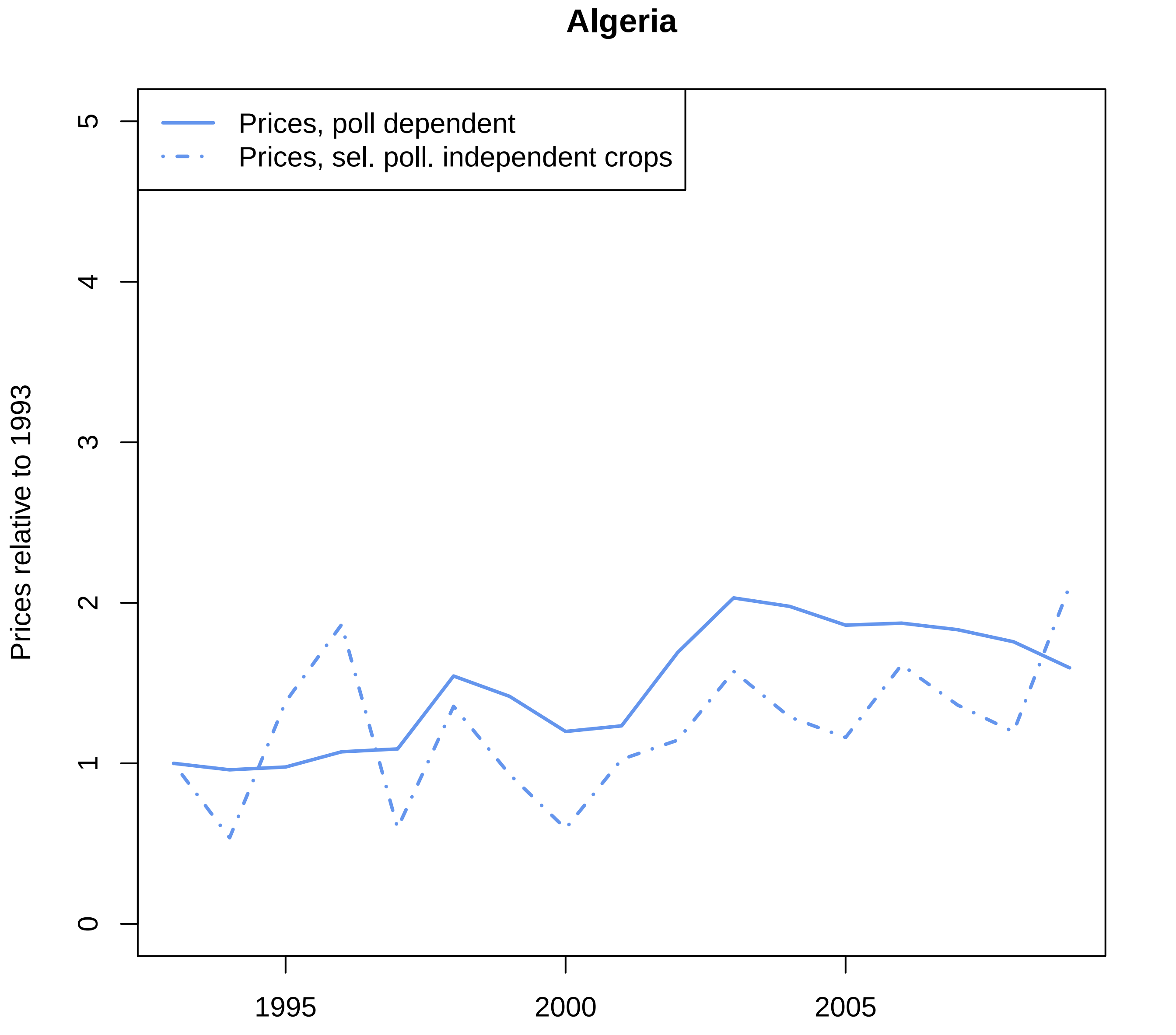
Algeria
Prices, poll dependent
Prices, sel. poll. independent crops
Prices relative to 1993
0
1
2
3
4
5
1995
2000
2005

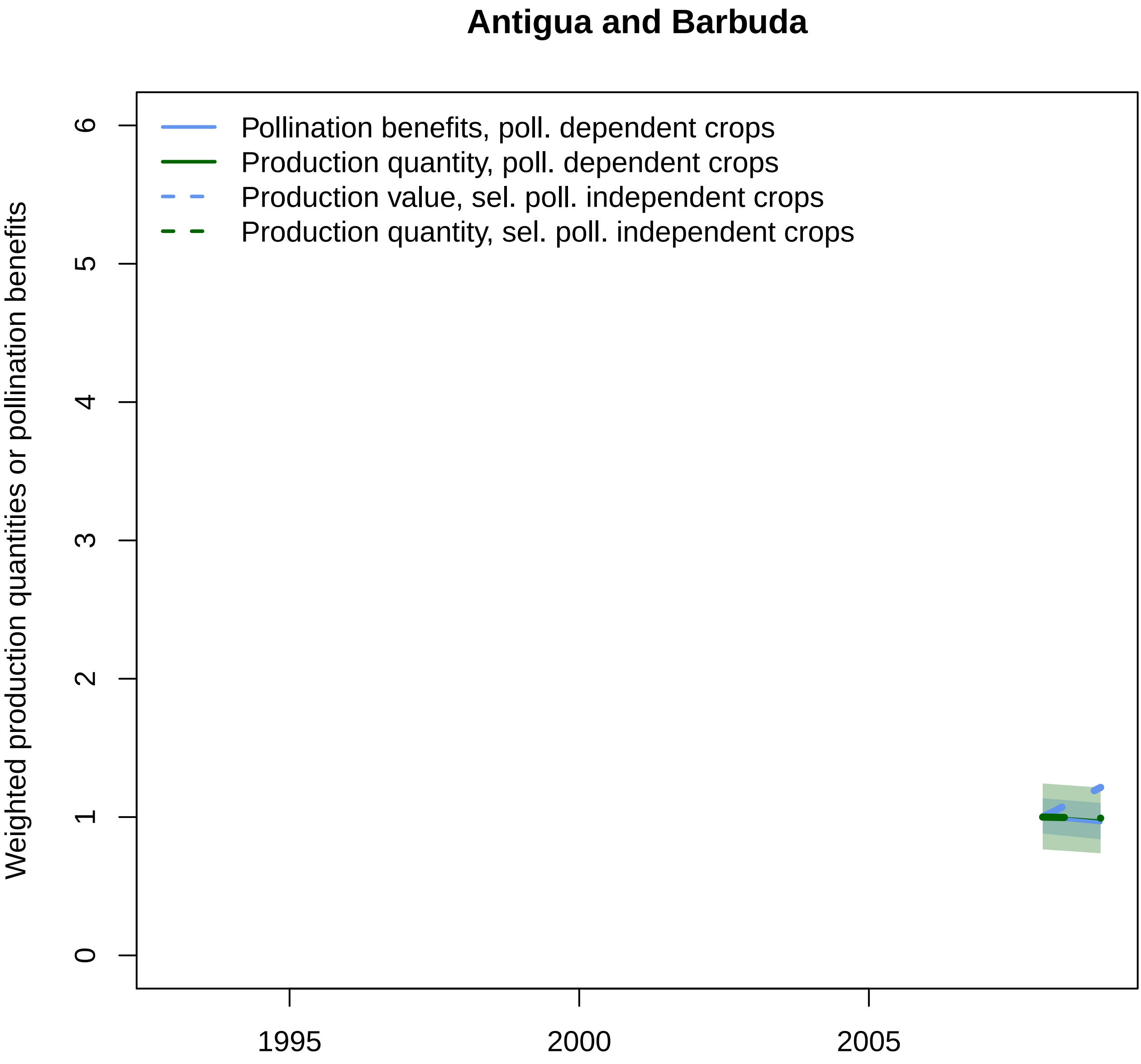
Antigua and Barbuda
Pollination benefits, poll. dependent crops
Production quantity, poll. dependent crops
Production value, sel. poll. independent crops
Production quantity, sel. poll. independent crops
Weighted production quantities or pollination benefits
0
1
2
3
4
5
6
1995
2000
2005

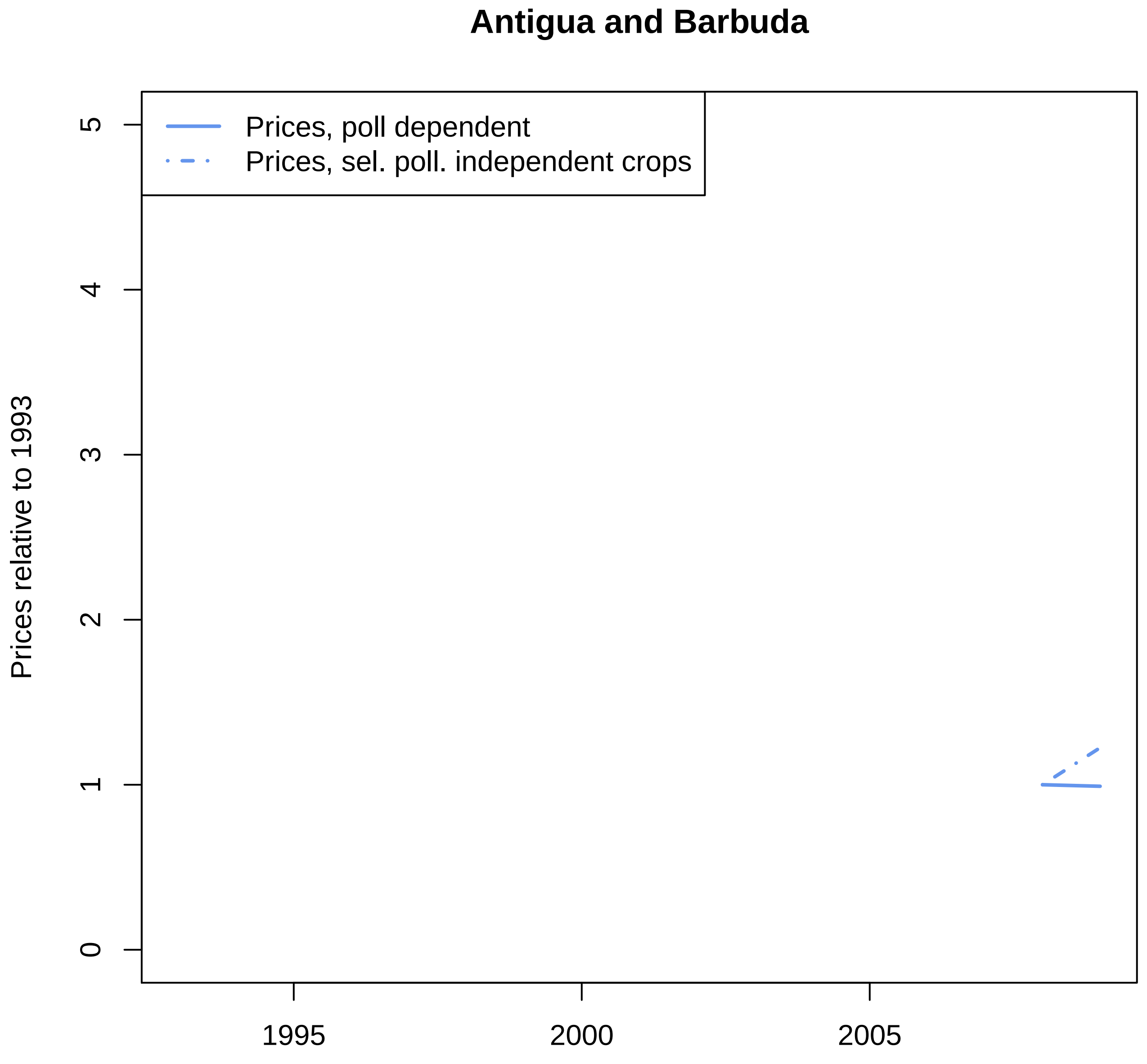

Antigua and Barbuda
Prices, poll dependent
Prices, sel. poll. independent crops
Prices relative to 1993
0
1
2
3
4
5
1995
2000
2005

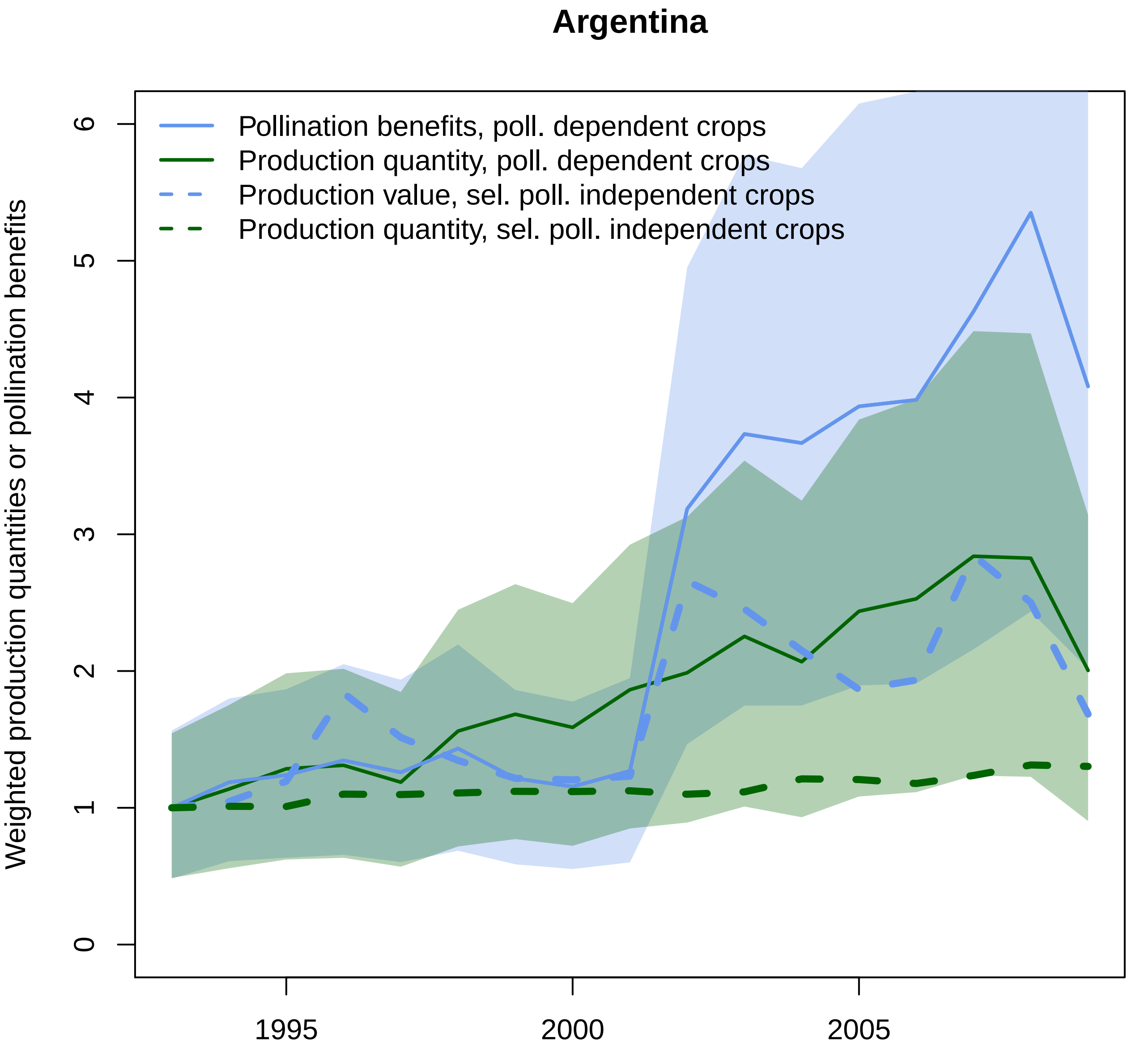

Argentina
Pollination benefits, poll. dependent crops
Production quantity, poll. dependent crops
Production value, sel. poll. independent crops
Production quantity, sel. poll. independent crops
Weighted production quantities or pollination benefits
0
1
2
3
4
5
6
1995
2000
2005

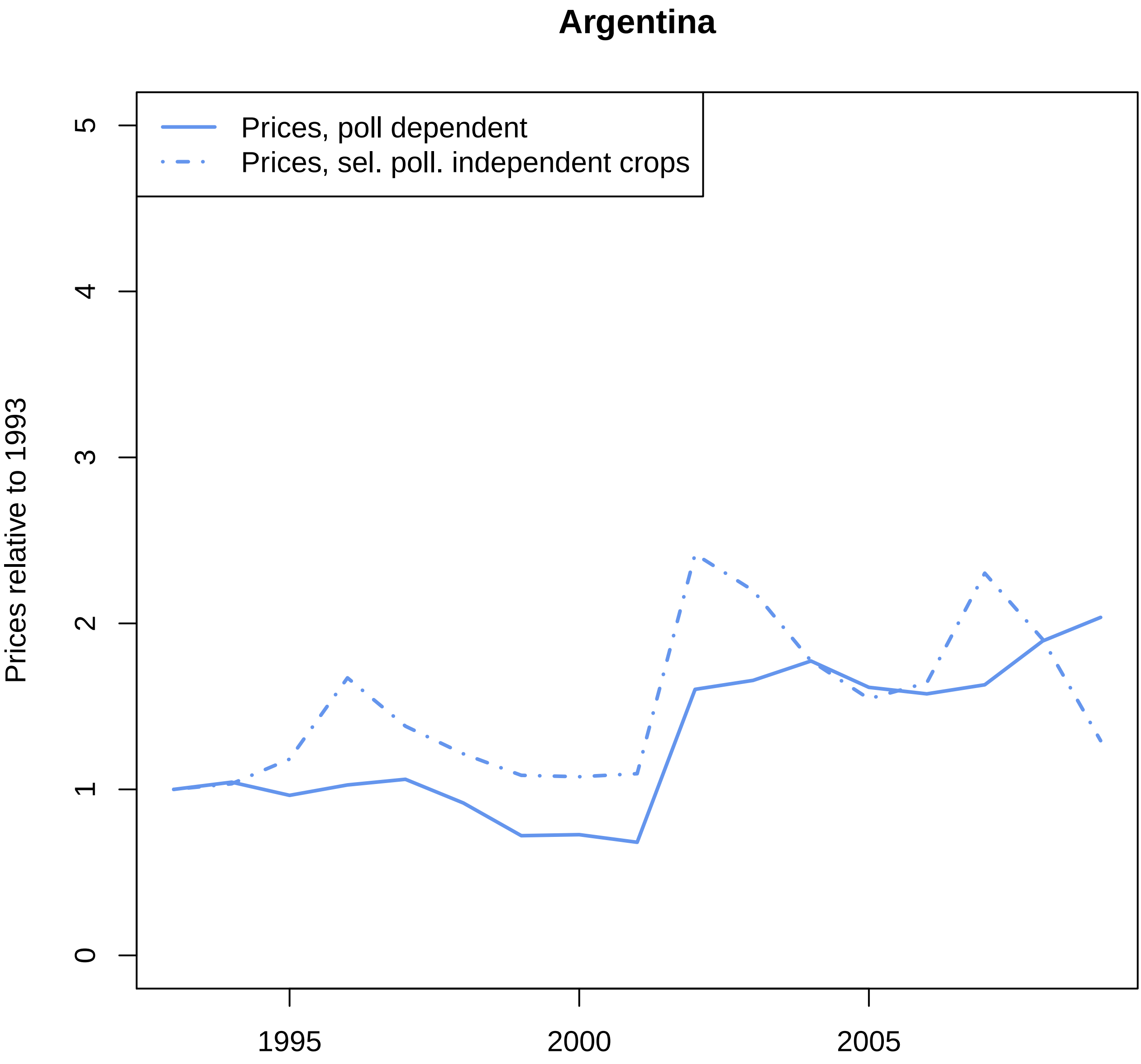
Argentina
Prices, poll dependent
Prices, sel. poll. independent crops
Prices relative to 1993
0
1
2
3
4
5
1995
2000
2005

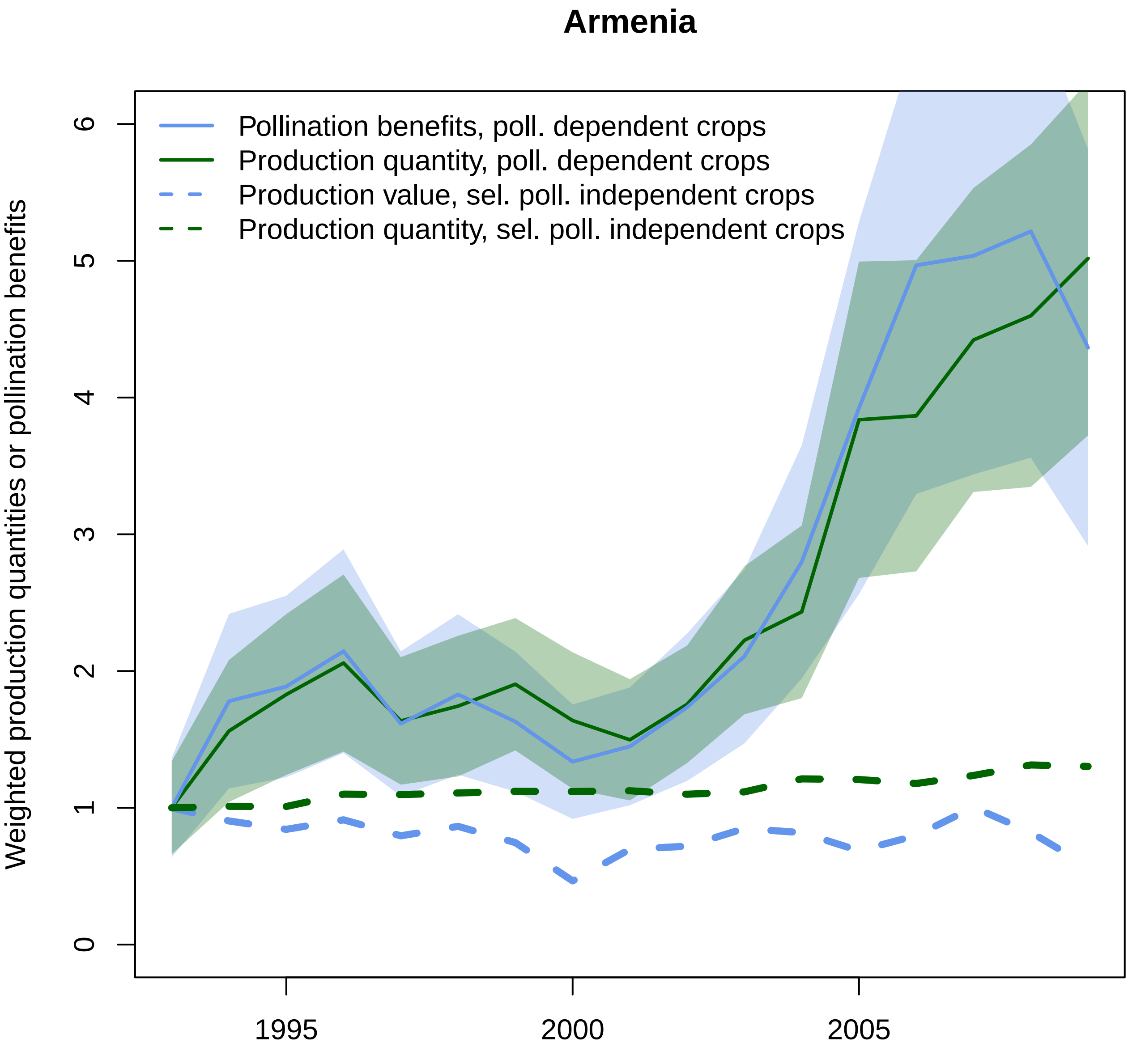
Armenia
Pollination benefits, poll. dependent crops
Production quantity, poll. dependent crops
Production value, sel. poll. independent crops
Production quantity, sel. poll. independent crops
Weighted production quantities or pollination benefits
0
1
2
3
4
5
6
1995
2000
2005

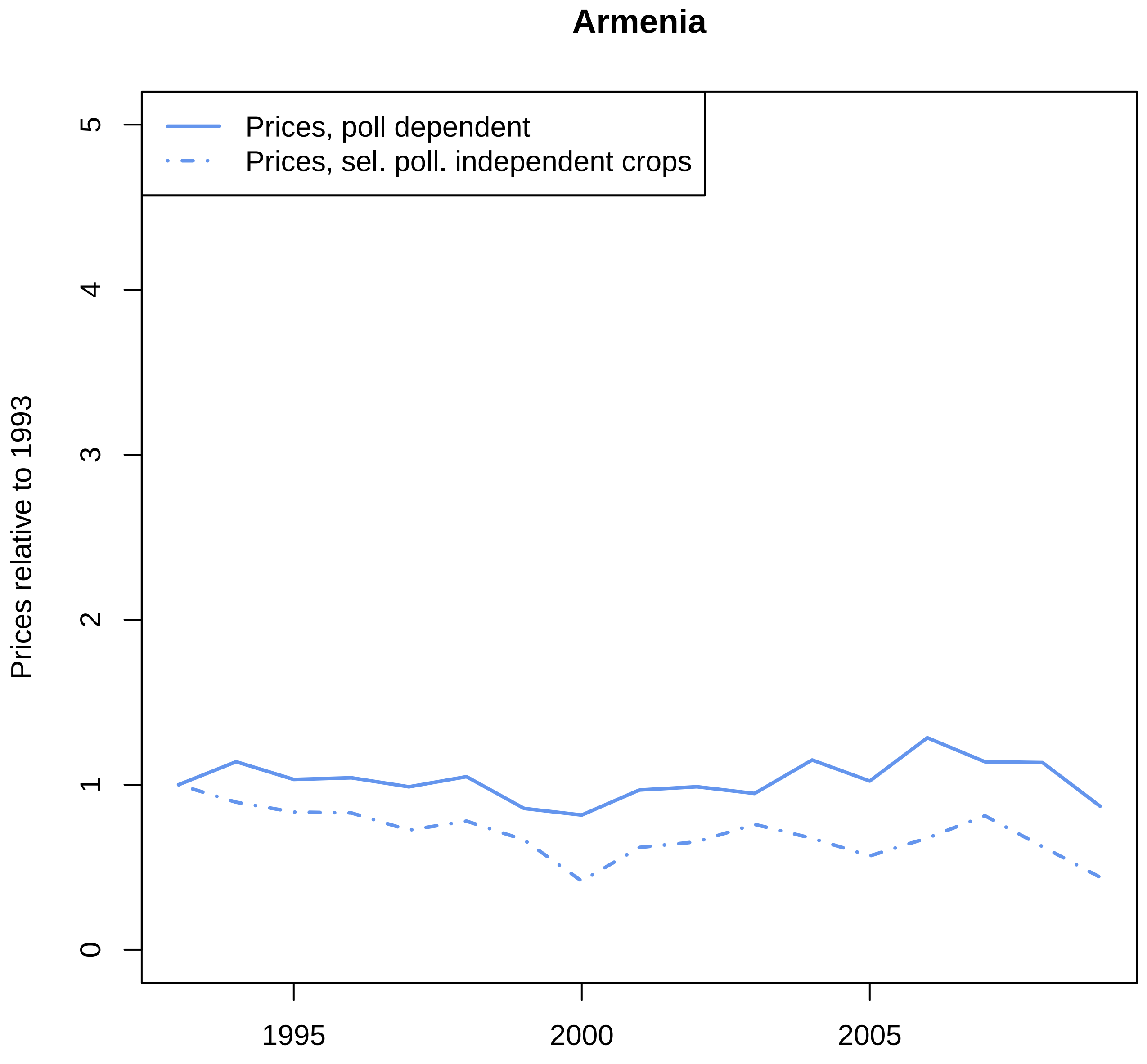
Armenia
Prices, poll dependent
Prices, sel. poll. independent crops
Prices relative to 1993
0
1
2
3
4
5
1995
2000
2005

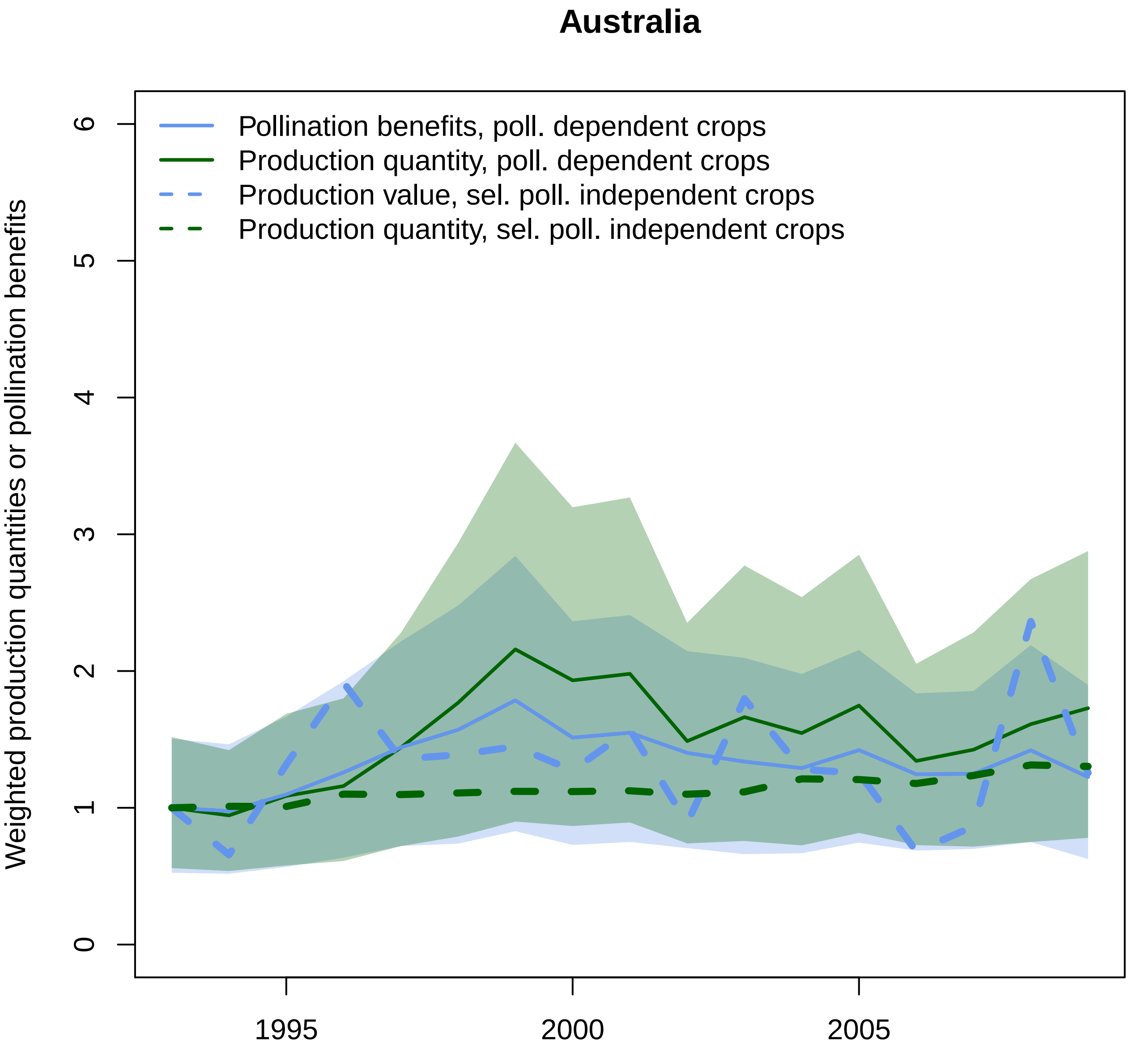
Australia
Pollination benefits, poll. dependent crops
Production quantity, poll. dependent crops
Production value, sel. poll. independent crops
Production quantity, sel. poll. independent crops
Weighted production quantities or pollination benefits
0
1
2
3
4
5
6
1995
2000
2005

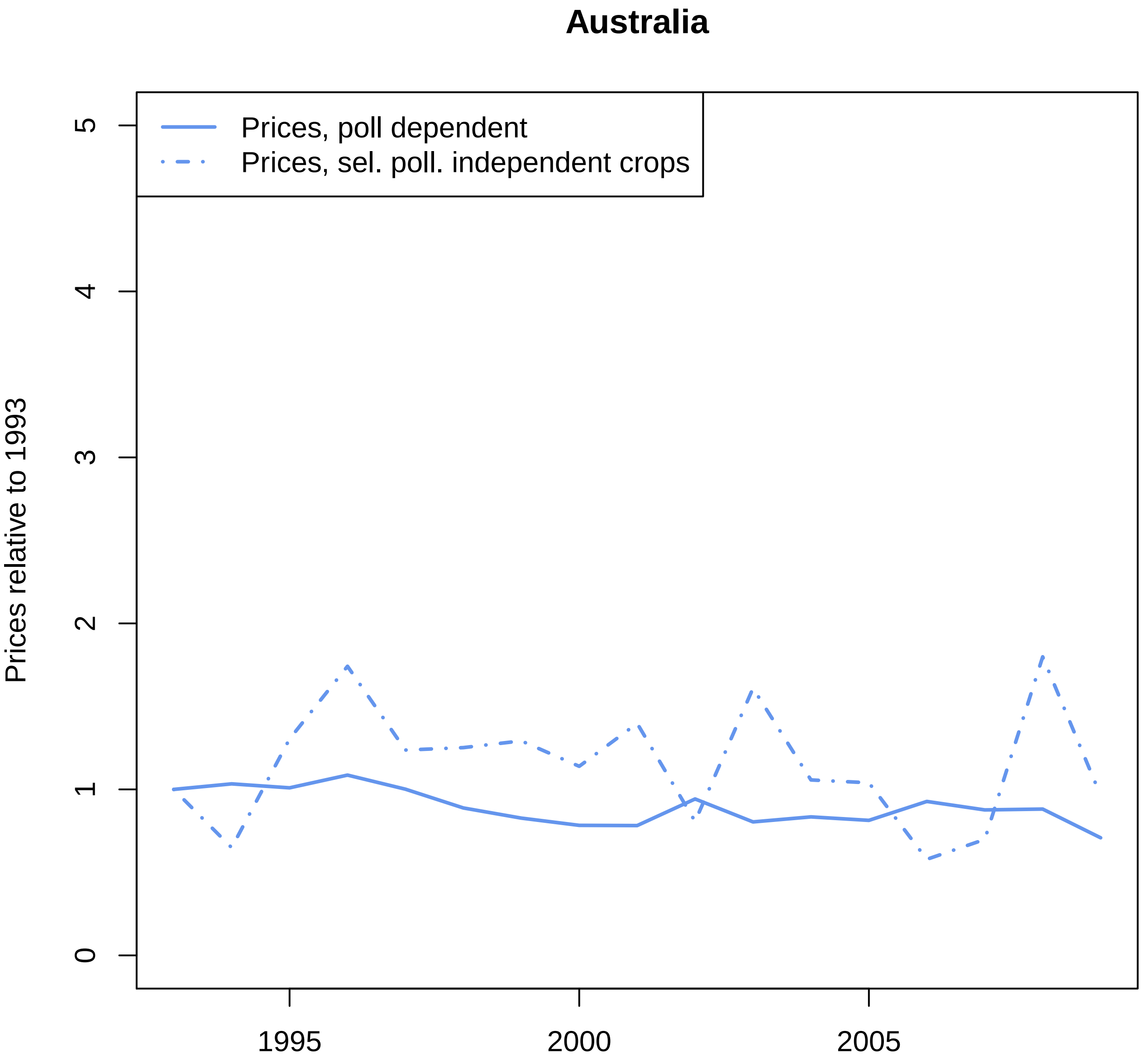
Australia
Prices, poll dependent
Prices, sel. poll. independent crops
Prices relative to 1993
0
1
2
3
4
5
1995
2000
2005

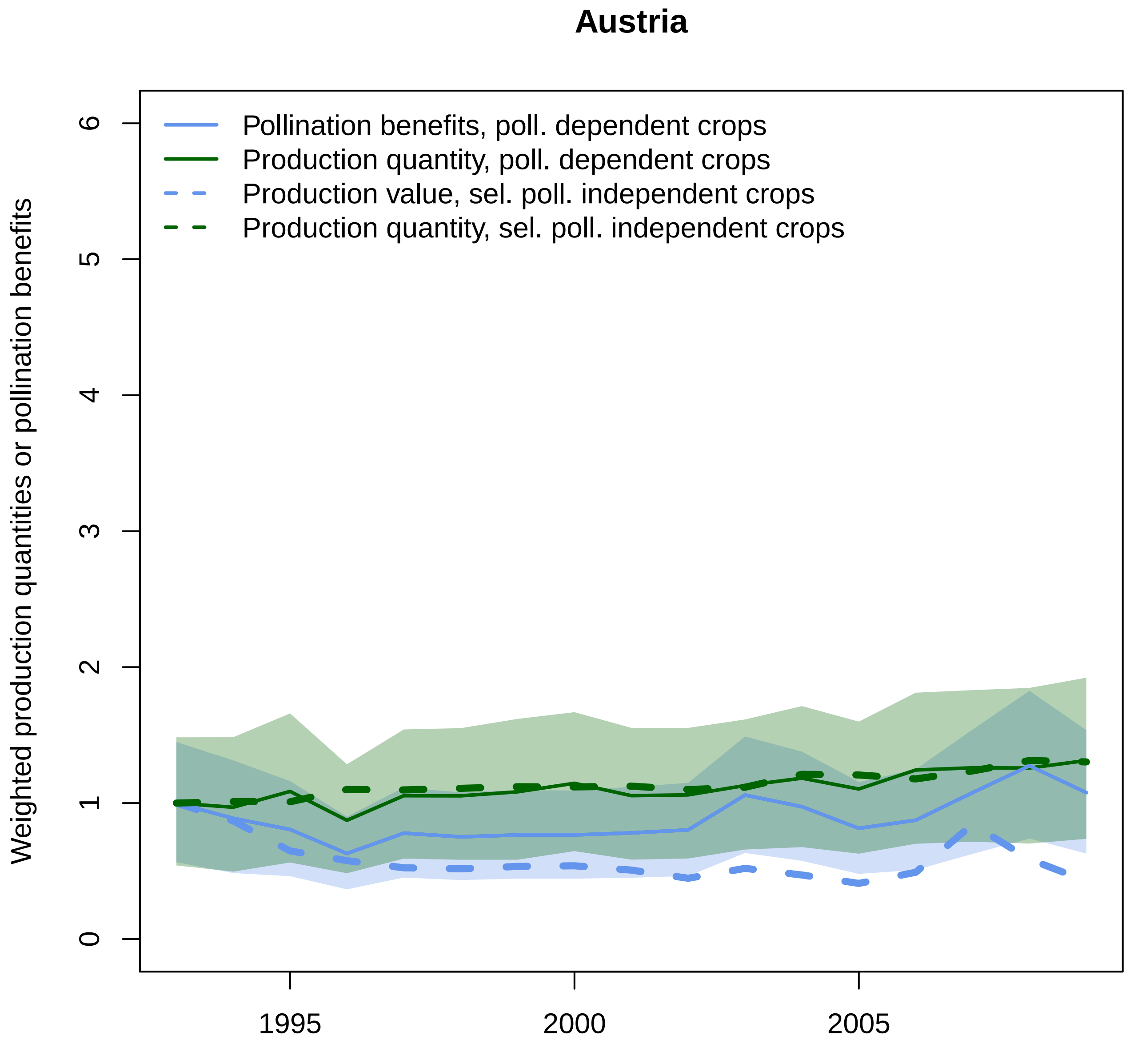
Austria
Pollination benefits, poll. dependent crops
Production quantity, poll. dependent crops
Production value, sel. poll. independent crops
Production quantity, sel. poll. independent crops
Weighted production quantities or pollination benefits
0
1
2
3
4
5
6
1995
2000
2005

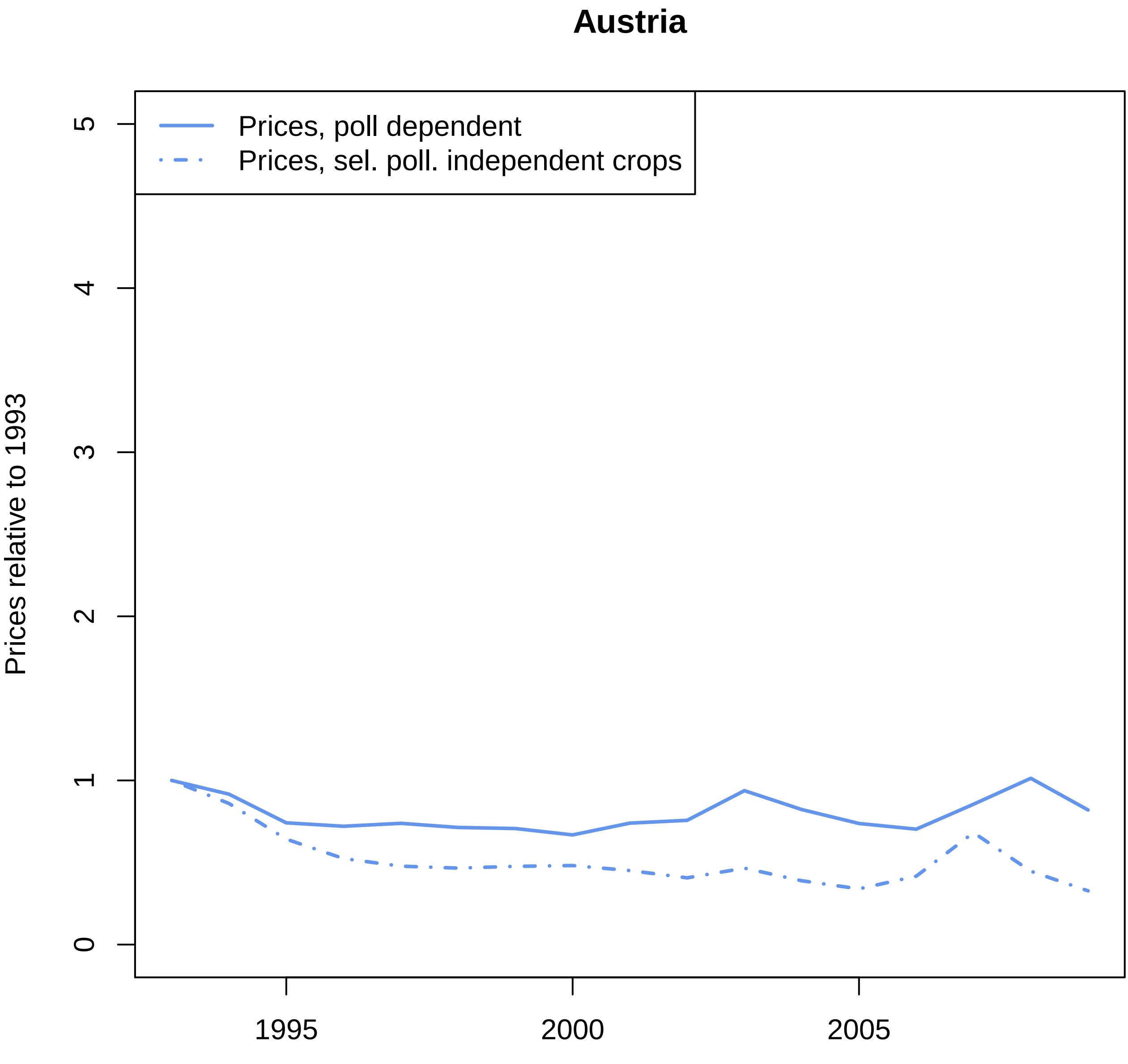

Austria
Prices, poll dependent
Prices, sel. poll. independent crops
Prices relative to 1993
0
1
2
3
4
5
1995
2000
2005

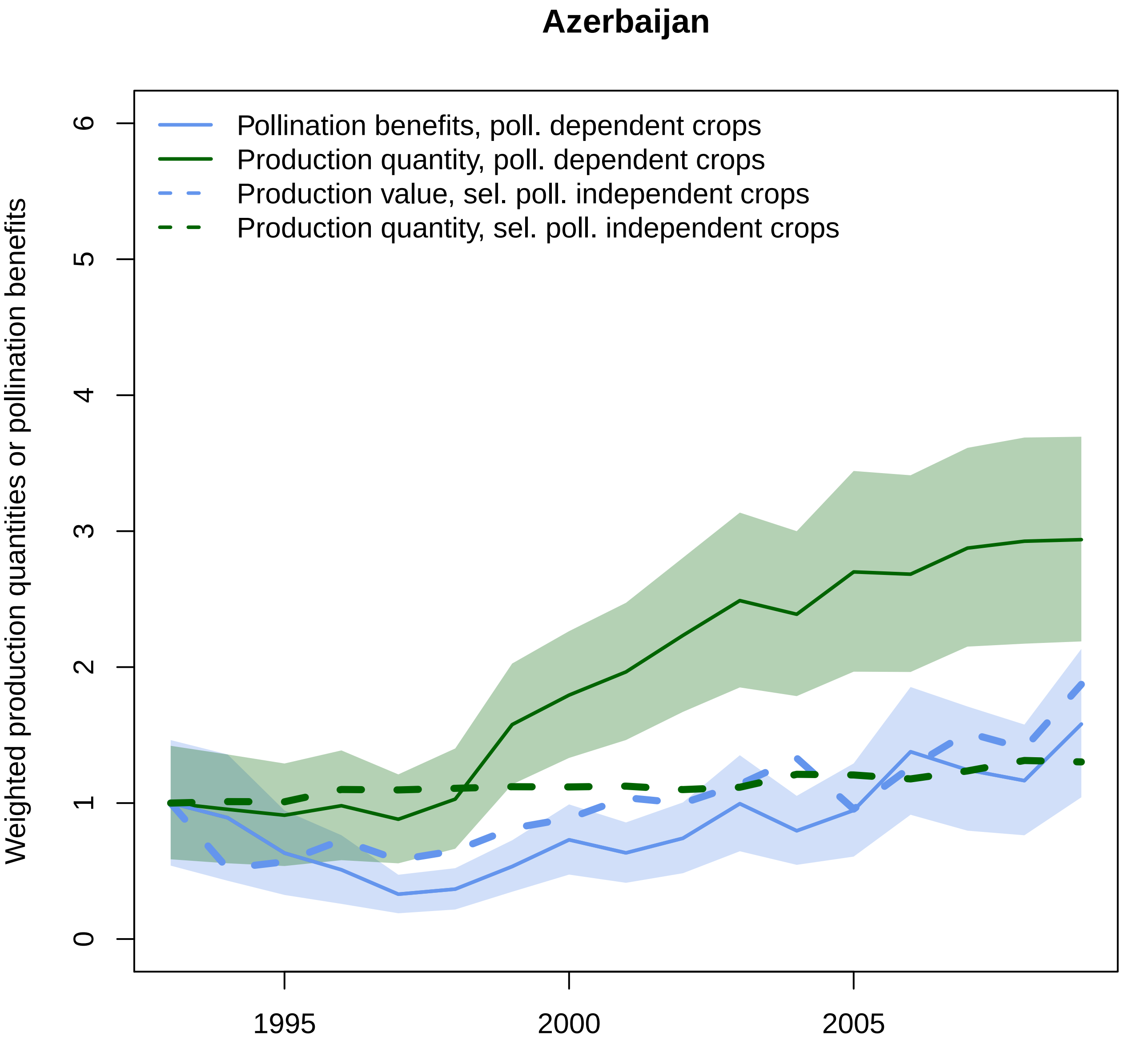

Azerbaijan
Pollination benefits, poll. dependent crops
Production quantity, poll. dependent crops
Production value, sel. poll. independent crops
Production quantity, sel. poll. independent crops
Weighted production quantities or pollination benefits
0
1
2
3
4
5
6
1995
2000
2005

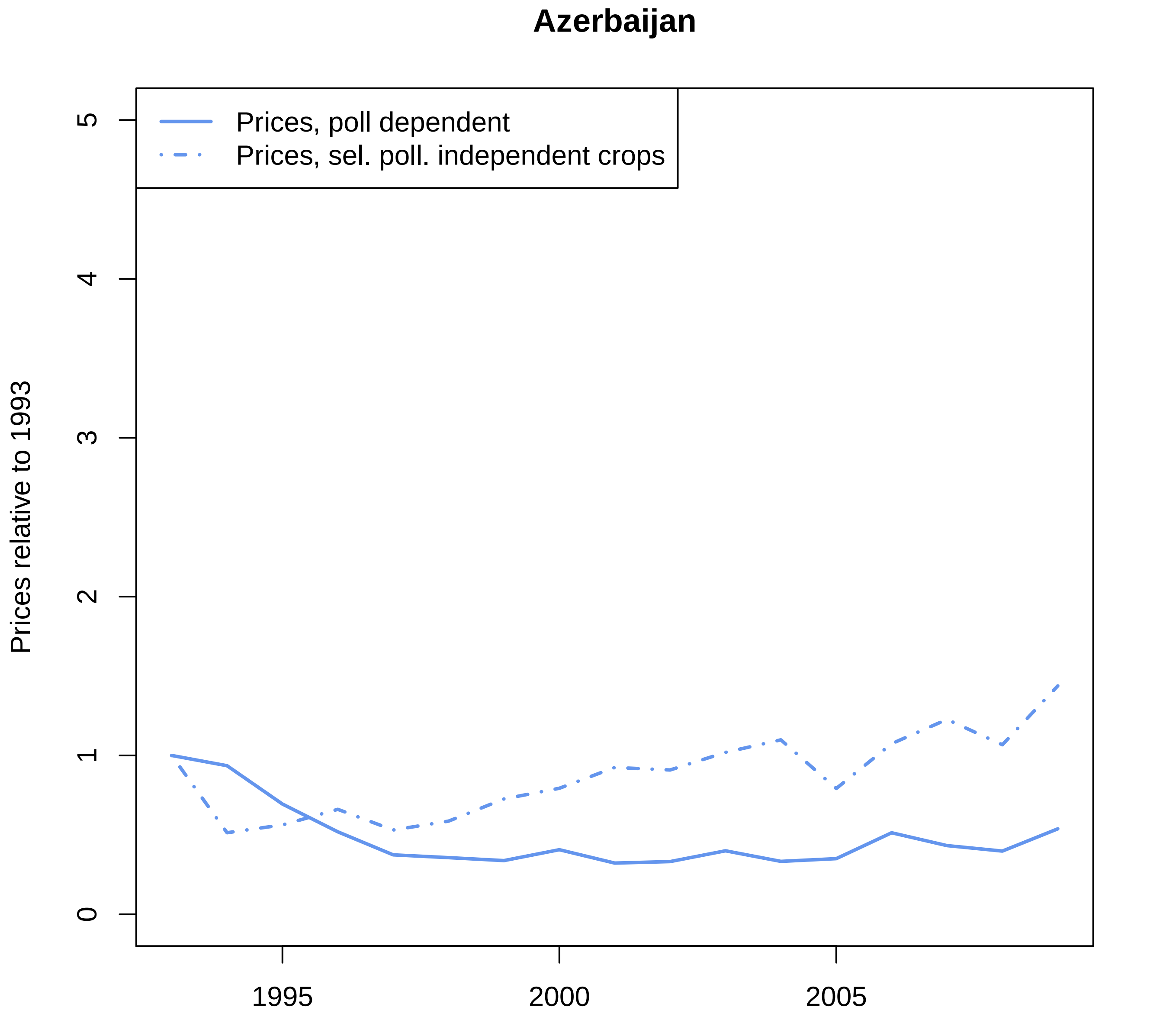

Azerbaijan
Prices, poll dependent
Prices, sel. poll. independent crops
Prices relative to 1993
0
1
2
3
4
5
1995
2000
2005

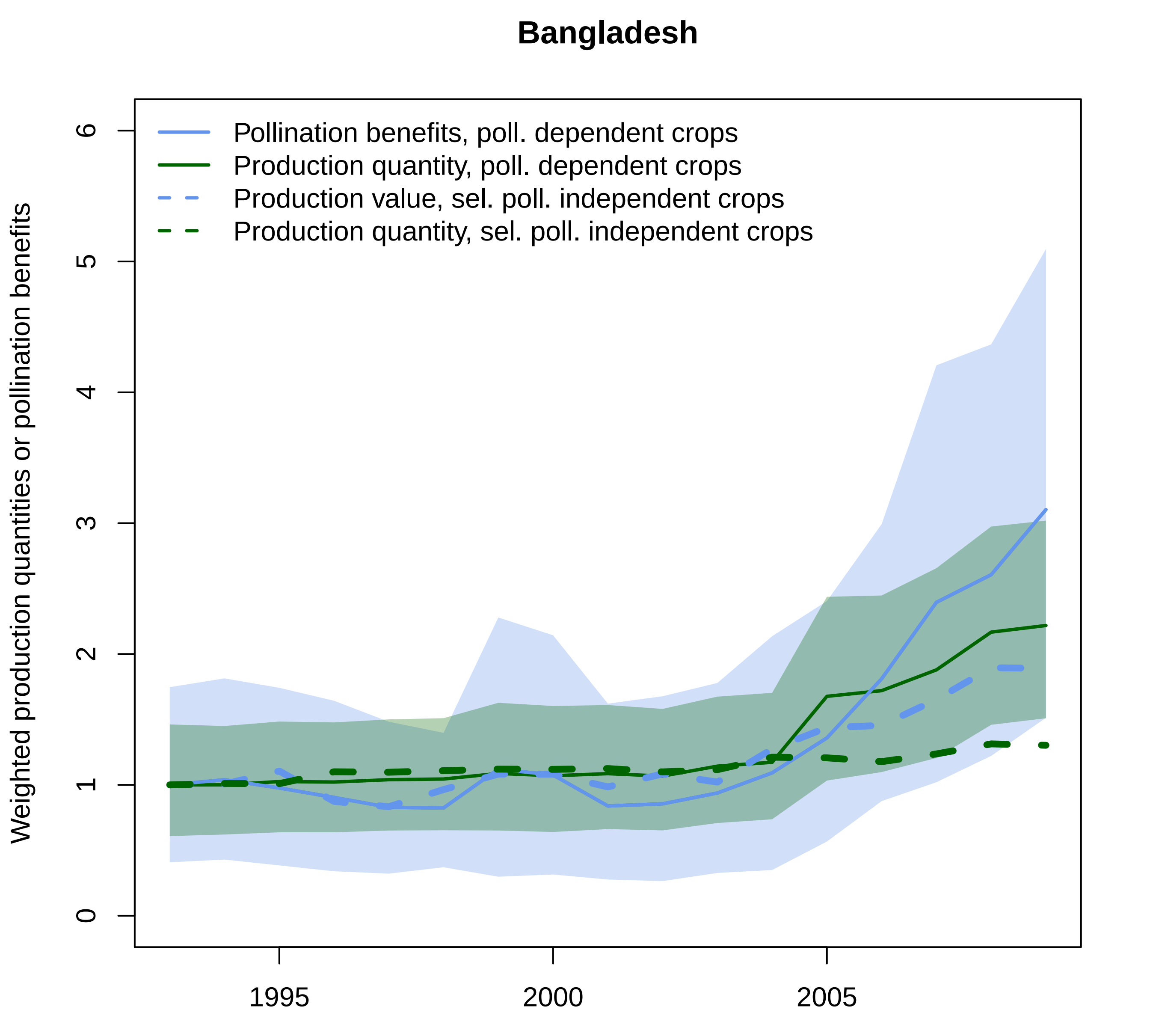
Bangladesh
Pollination benefits, poll. dependent crops
Production quantity, poll. dependent crops
Production value, sel. poll. independent crops
Production quantity, sel. poll. independent crops
Weighted production quantities or pollination benefits
0
1
2
3
4
5
6
1995
2000
2005

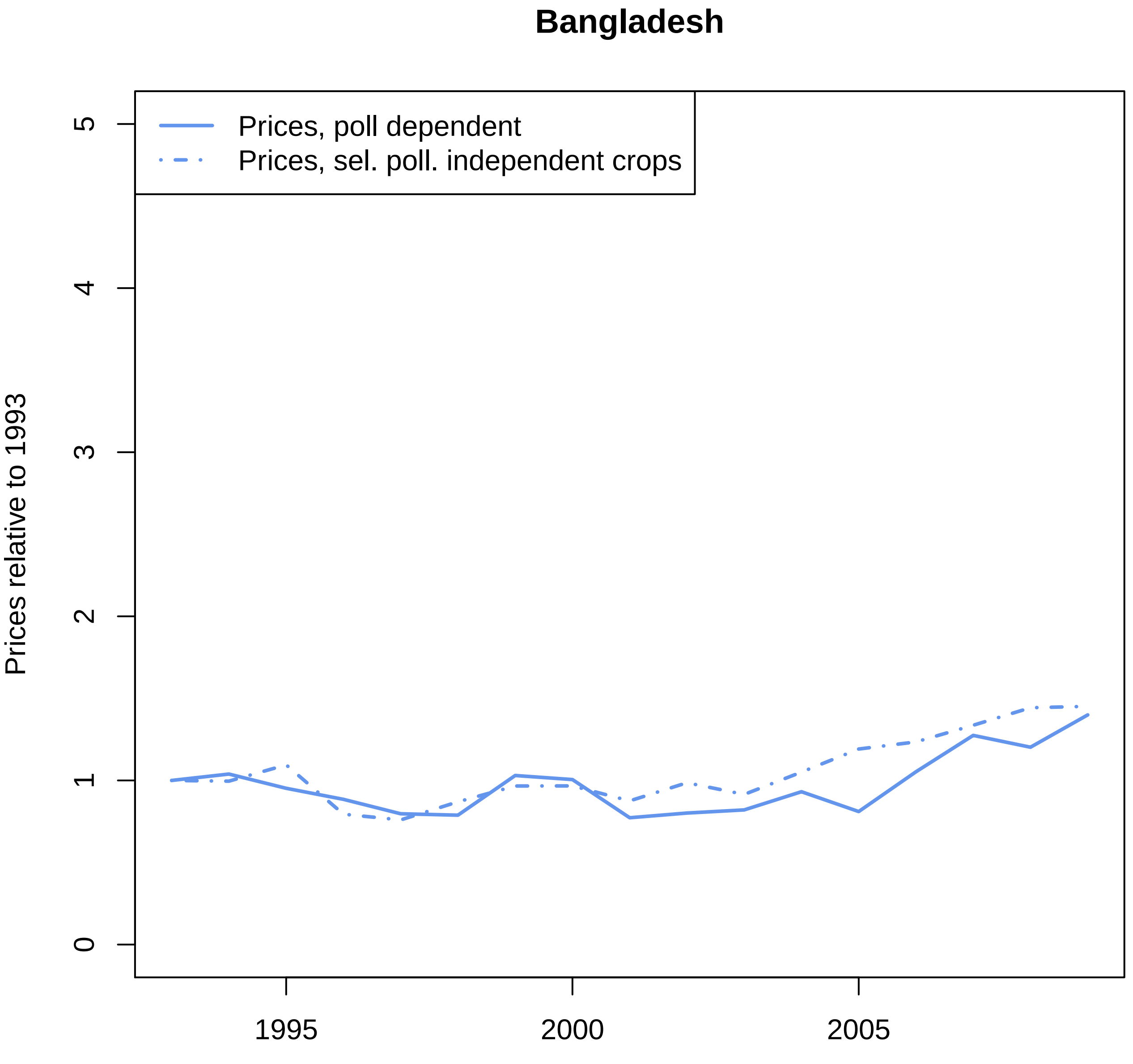
Bangladesh
Prices, poll dependent
Prices, sel. poll. independent crops
Prices relative to 1993
0
1
2
3
4
5
1995
2000
2005

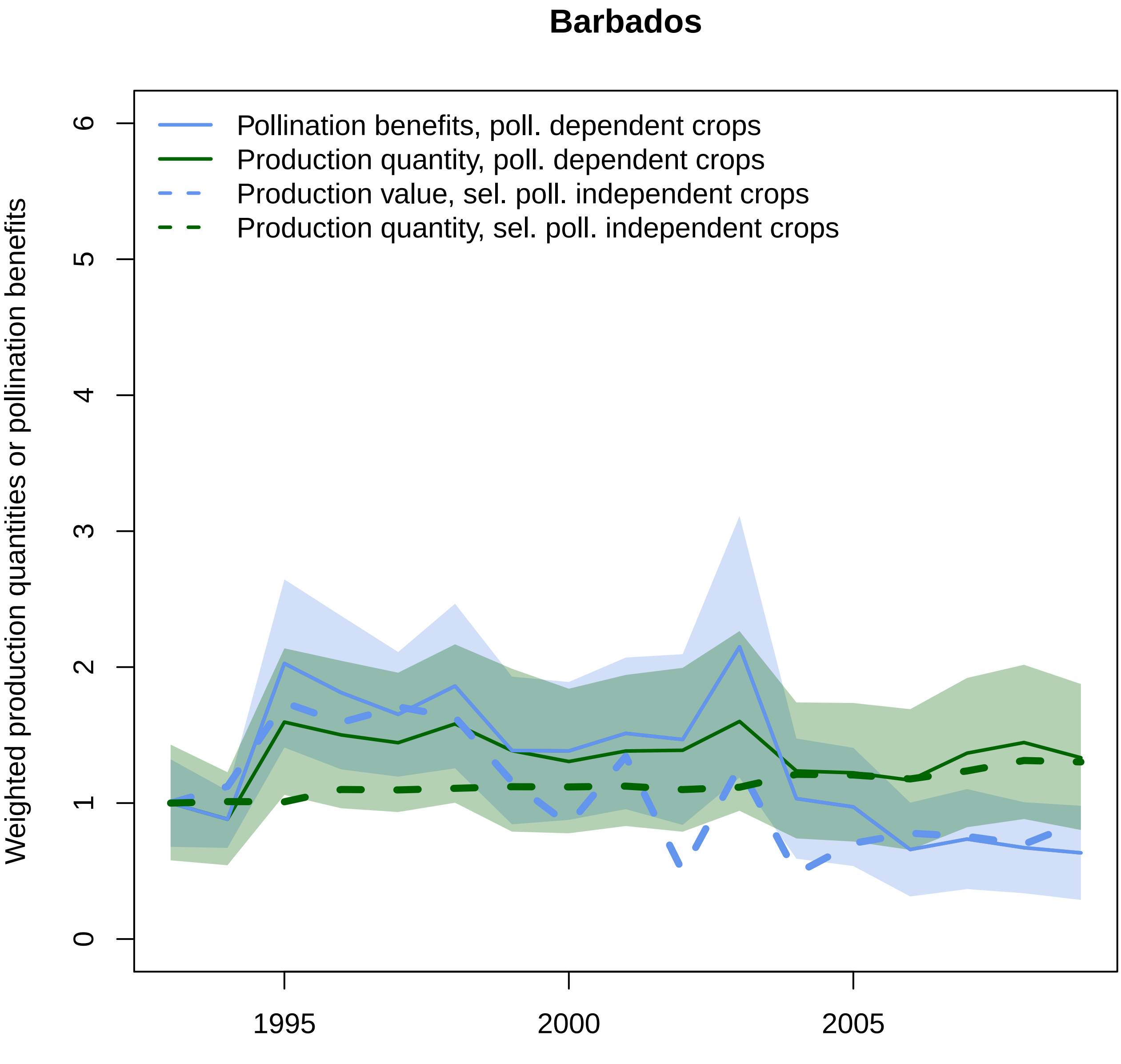

Barbados
Pollination benefits, poll. dependent crops
Production quantity, poll. dependent crops
Production value, sel. poll. independent crops
Production quantity, sel. poll. independent crops
Weighted production quantities or pollination benefits
0
1
2
3
4
5
6
1995
2000
2005

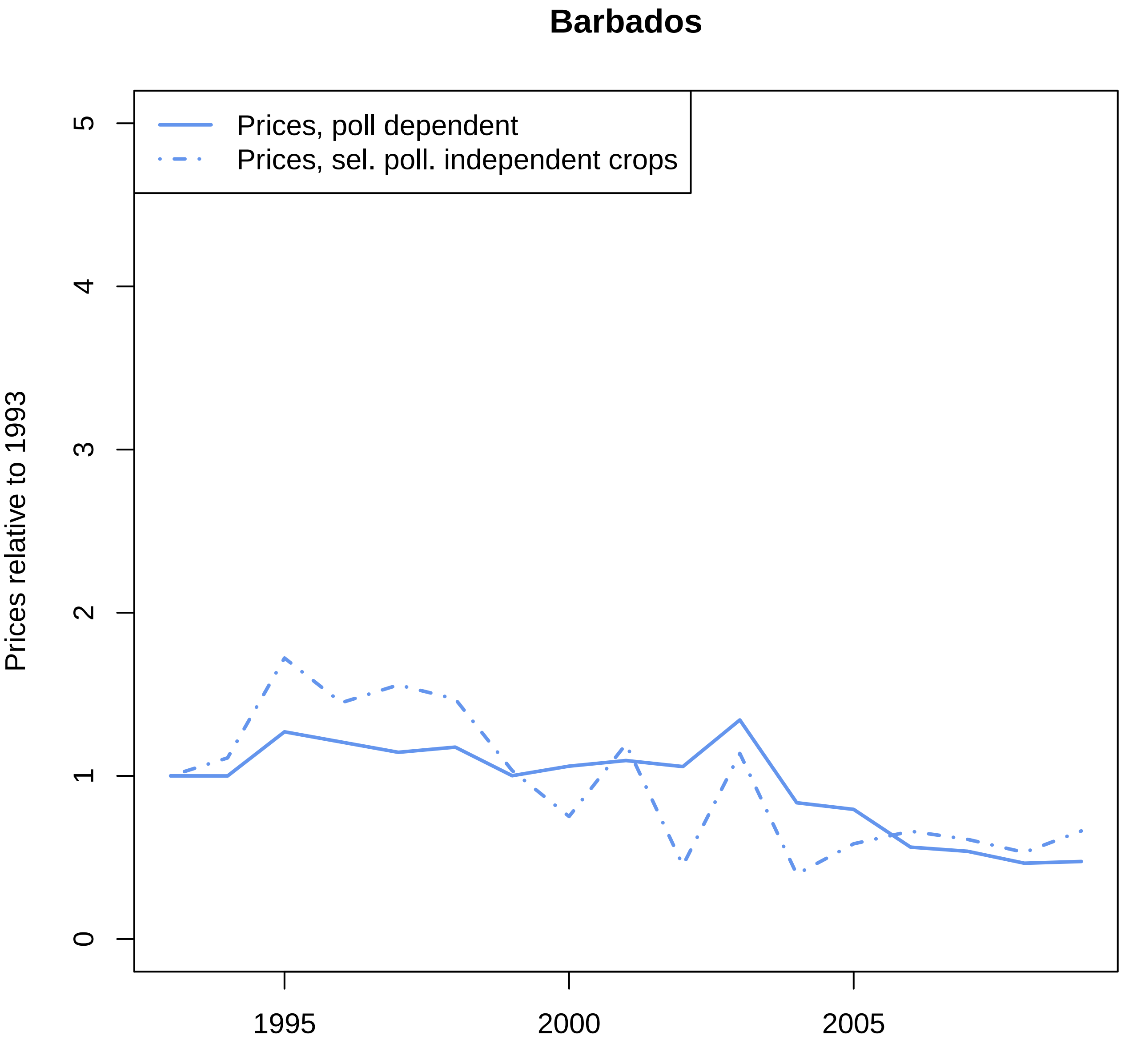

Barbados
Prices, poll dependent
Prices, sel. poll. independent crops
Prices relative to 1993
0
1
2
3
4
5
1995
2000
2005

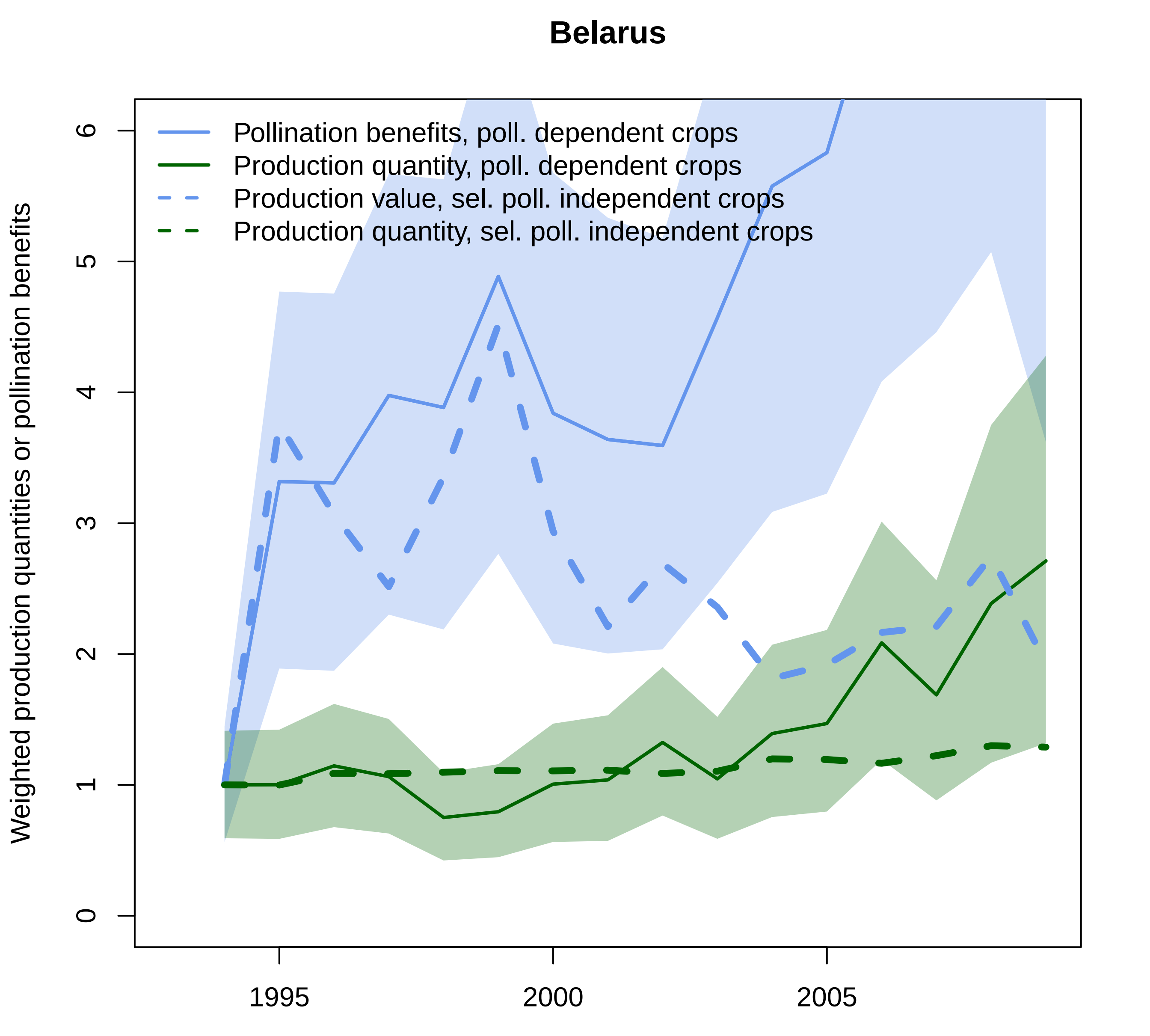
Belarus
Pollination benefits, poll. dependent crops
Production quantity, poll. dependent crops
Production value, sel. poll. independent crops
Production quantity, sel. poll. independent crops
Weighted production quantities or pollination benefits
0
1
2
3
4
5
6
1995
2000
2005

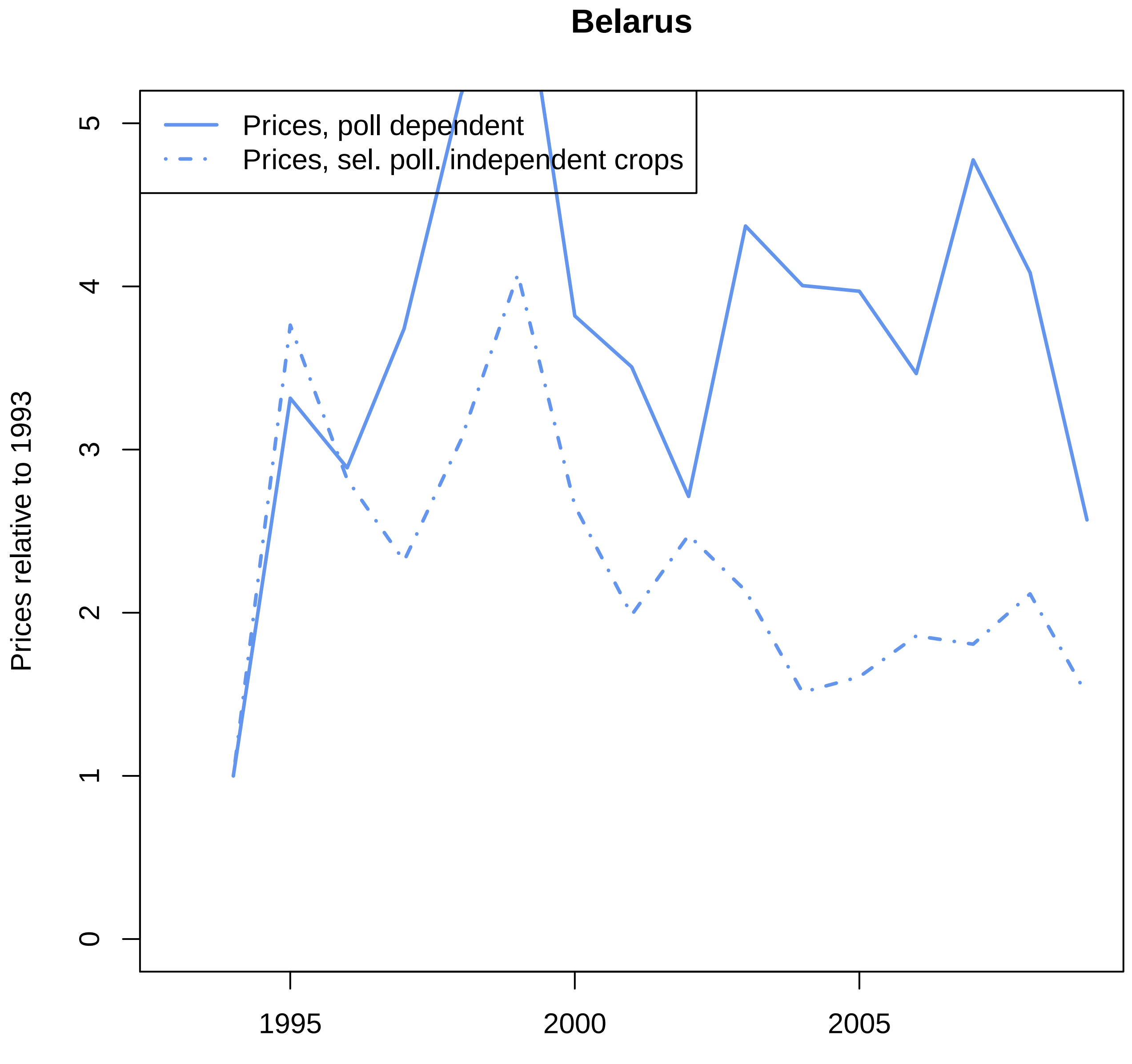

Belarus
Prices, poll dependent
Prices, sel. poll. independent crops
Prices relative to 1993
0
1
2
3
4
5
1995
2000
2005

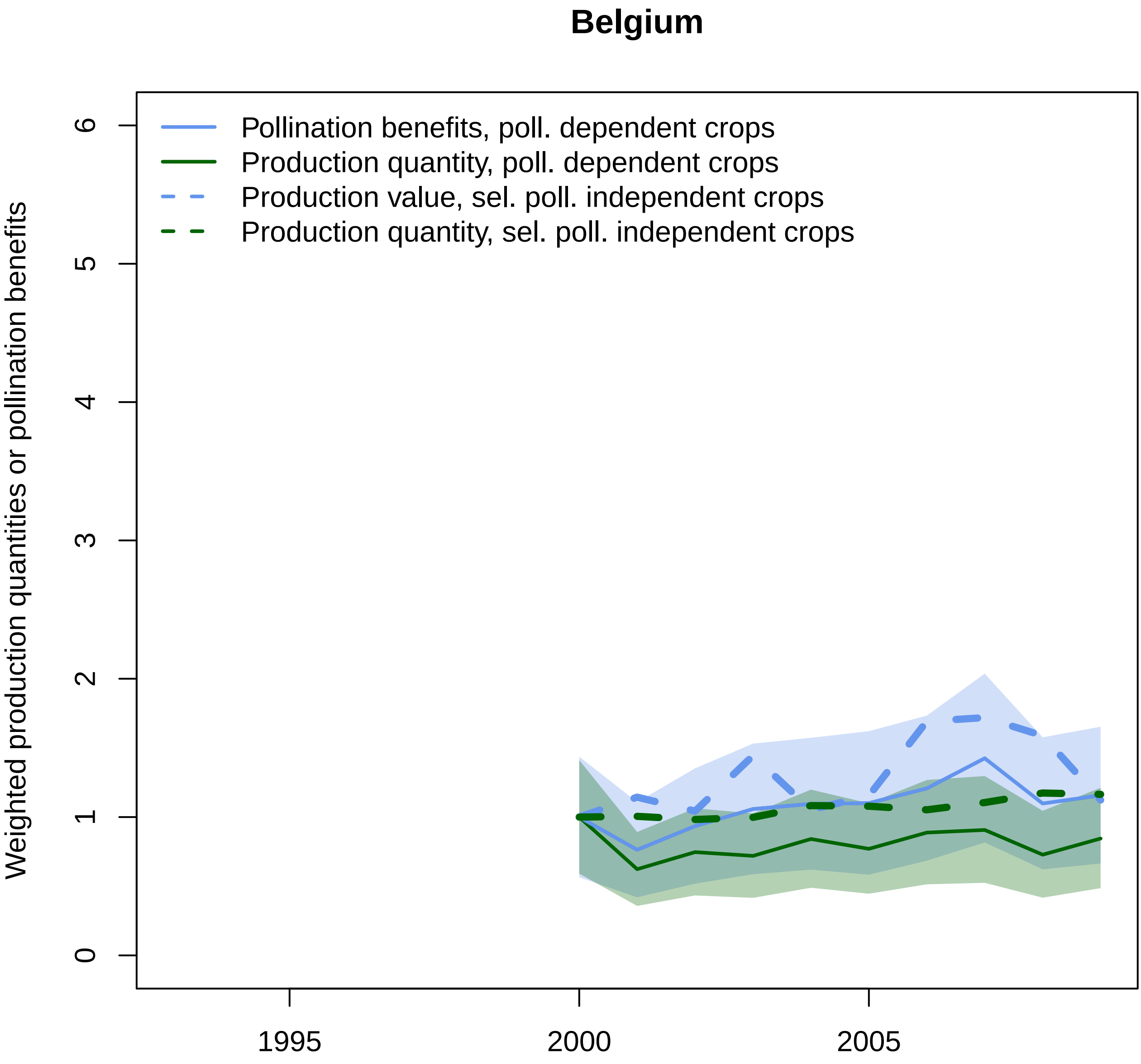
Belgium
Pollination benefits, poll. dependent crops
Production quantity, poll. dependent crops
Production value, sel. poll. independent crops
Production quantity, sel. poll. independent crops
Weighted production quantities or pollination benefits
0
1
2
3
4
5
6
1995
2000
2005

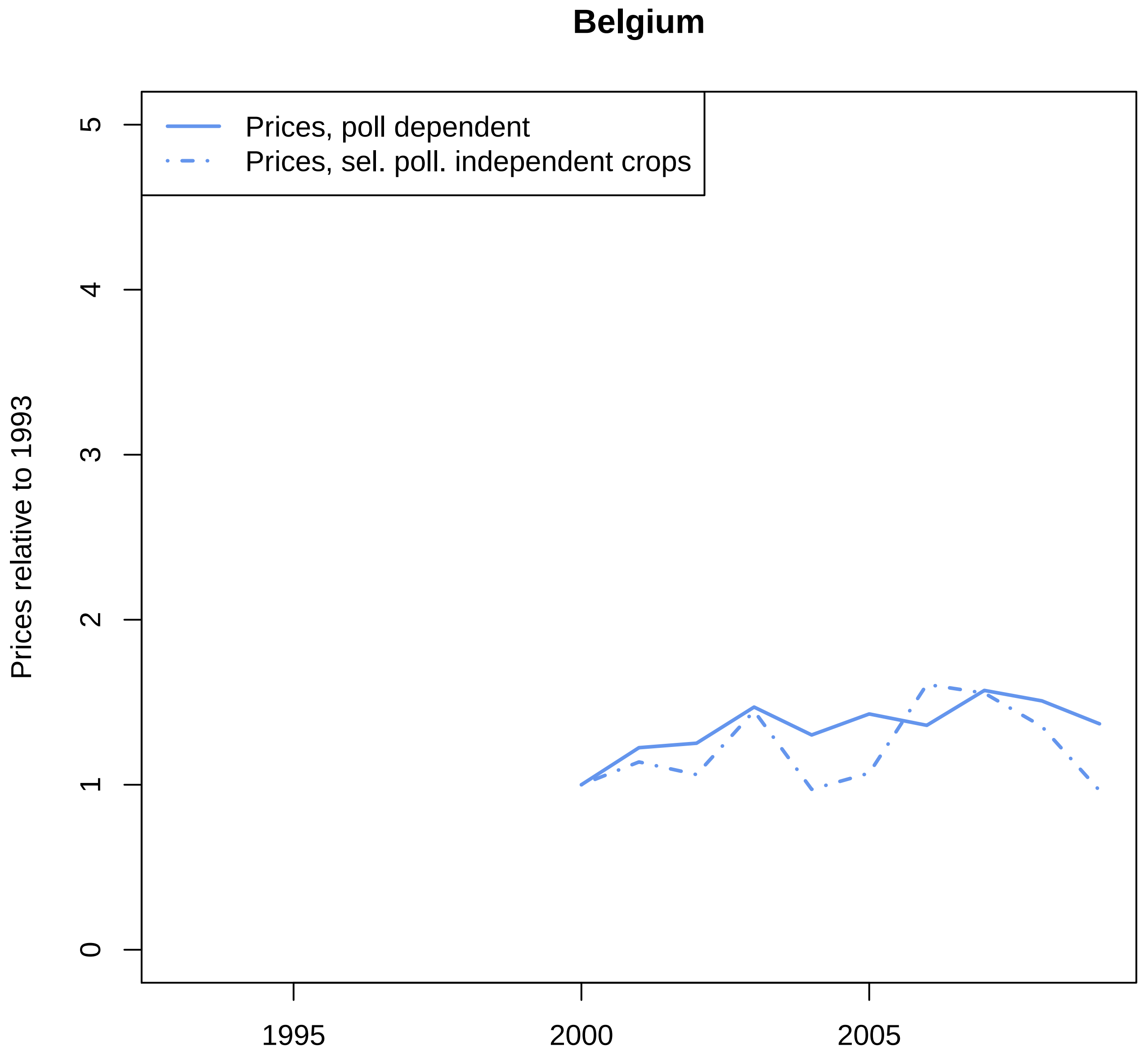
Belgium
Prices, poll dependent
Prices, sel. poll. independent crops
Prices relative to 1993
0
1
2
3
4
5
1995
2000
2005

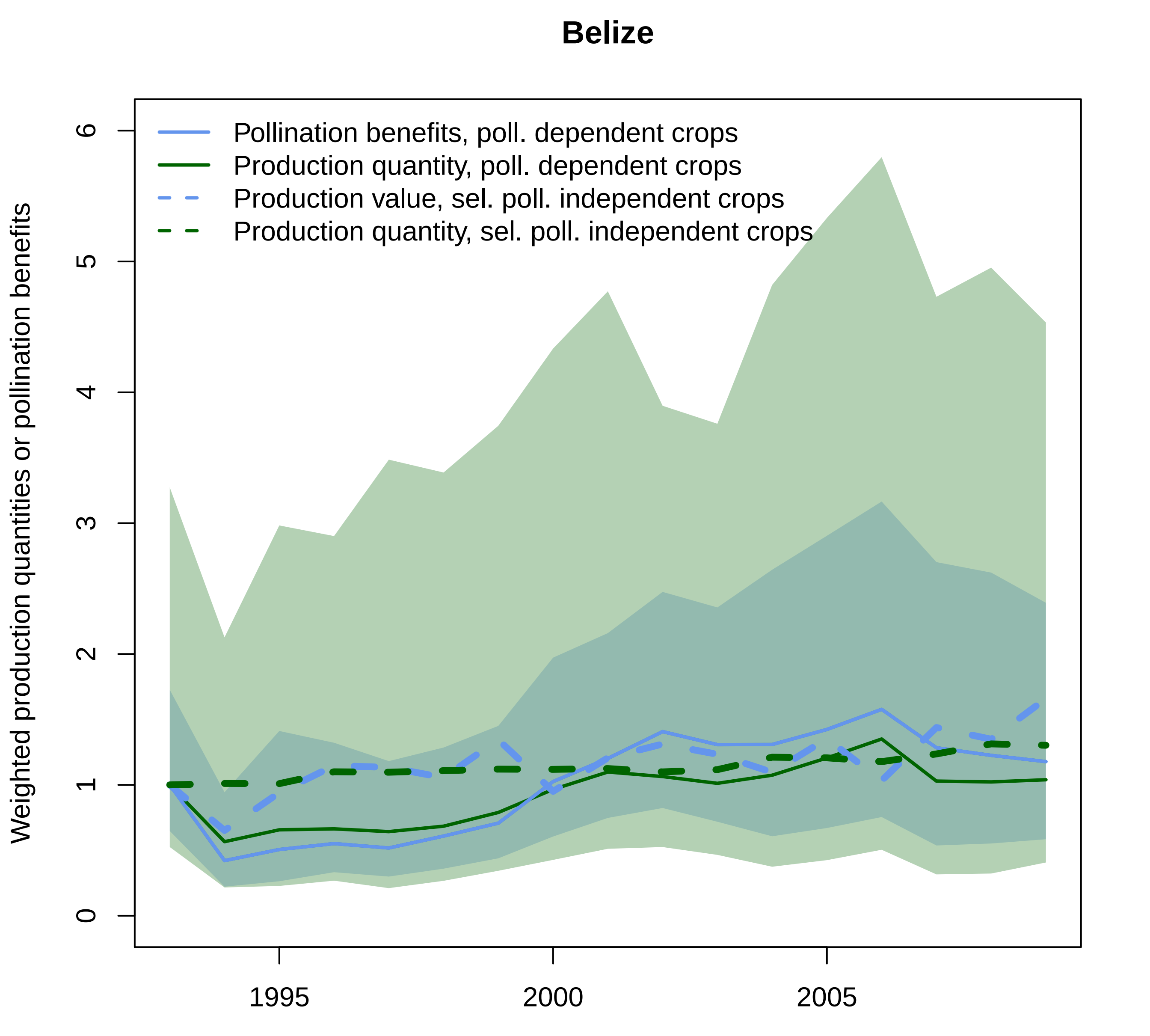
Belize
Pollination benefits, poll. dependent crops
Production quantity, poll. dependent crops
Production value, sel. poll. independent crops
Production quantity, sel. poll. independent crops
Weighted production quantities or pollination benefits
0
1
2
3
4
5
6
1995
2000
2005

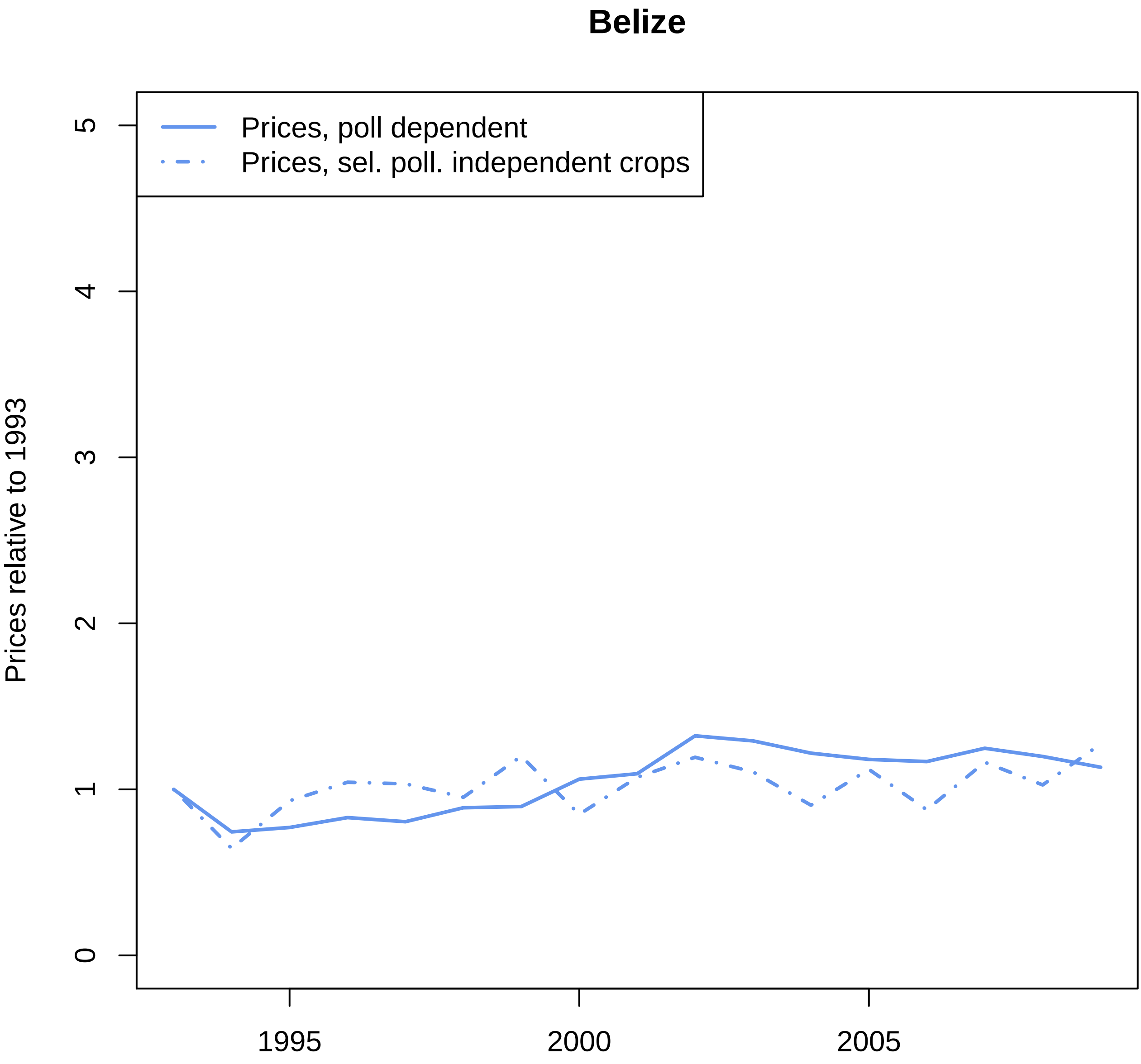

Belize
Prices, poll dependent
Prices, sel. poll. independent crops
Prices relative to 1993
0
1
2
3
4
5
1995
2000
2005

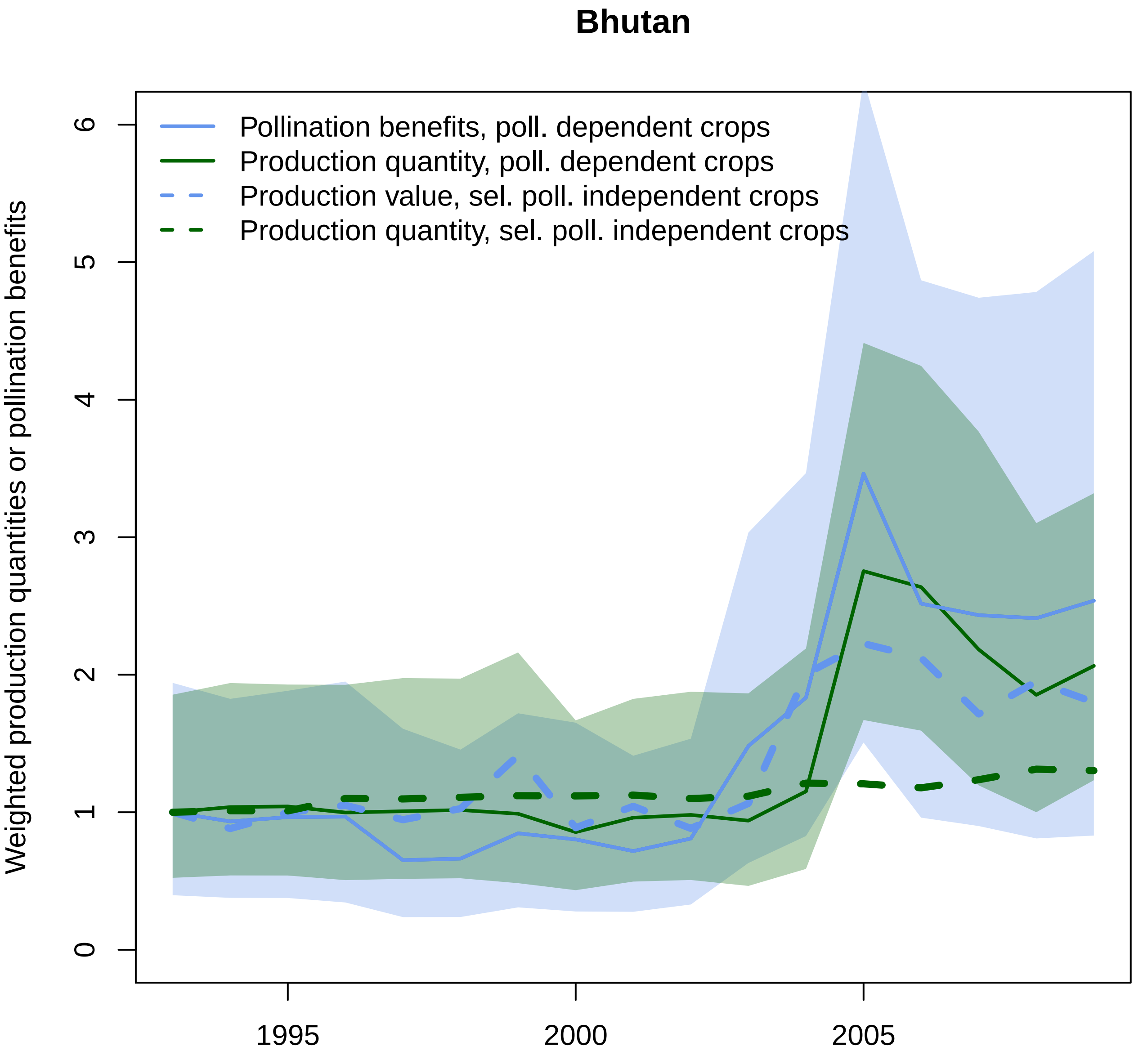

Bhutan
Pollination benefits, poll. dependent crops
Production quantity, poll. dependent crops
Production value, sel. poll. independent crops
Production quantity, sel. poll. independent crops
Weighted production quantities or pollination benefits
0
1
2
3
4
5
6
1995
2000
2005

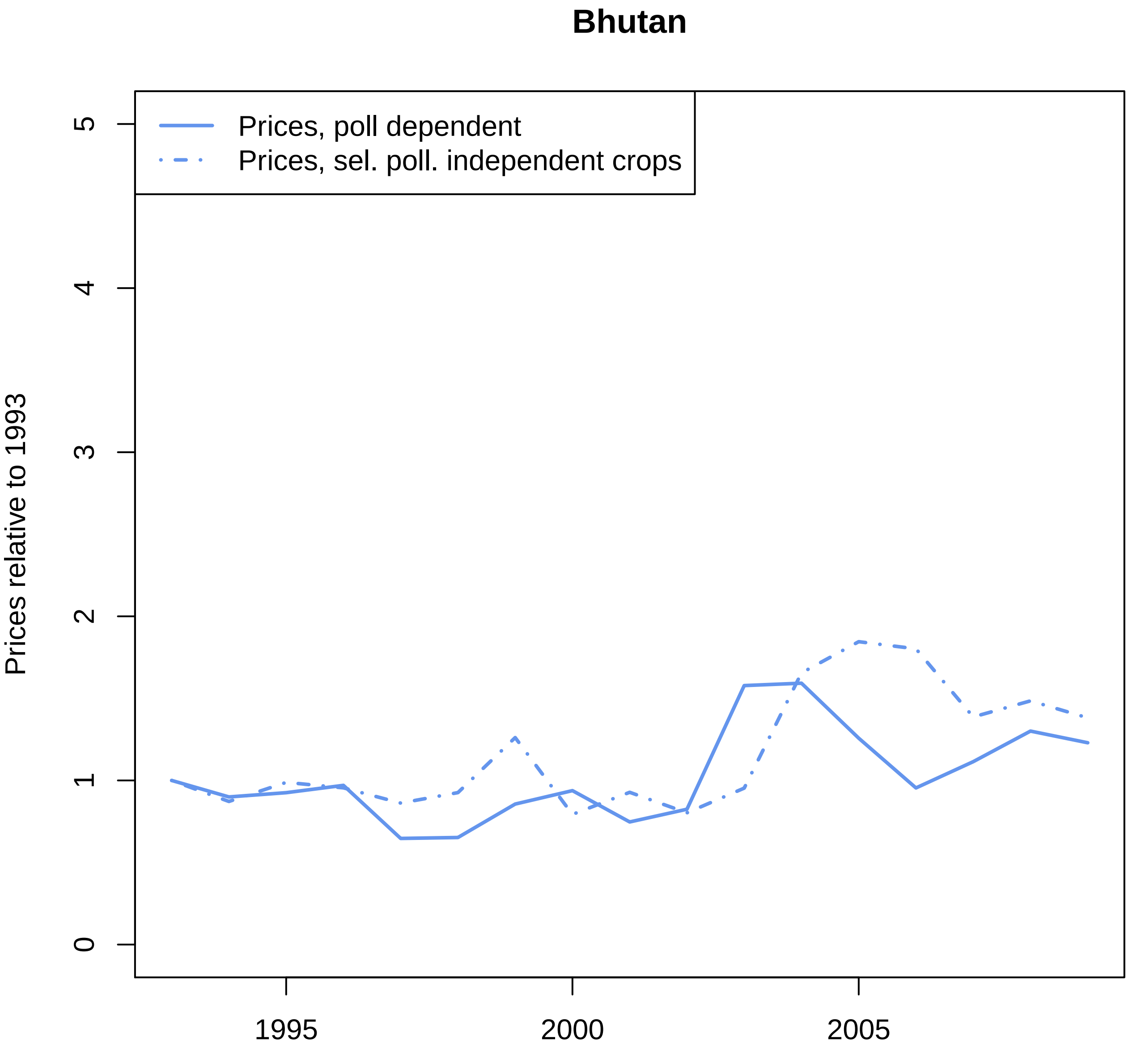
Bhutan
Prices, poll dependent
Prices, sel. poll. independent crops
Prices relative to 1993
0
1
2
3
4
5
1995
2000
2005

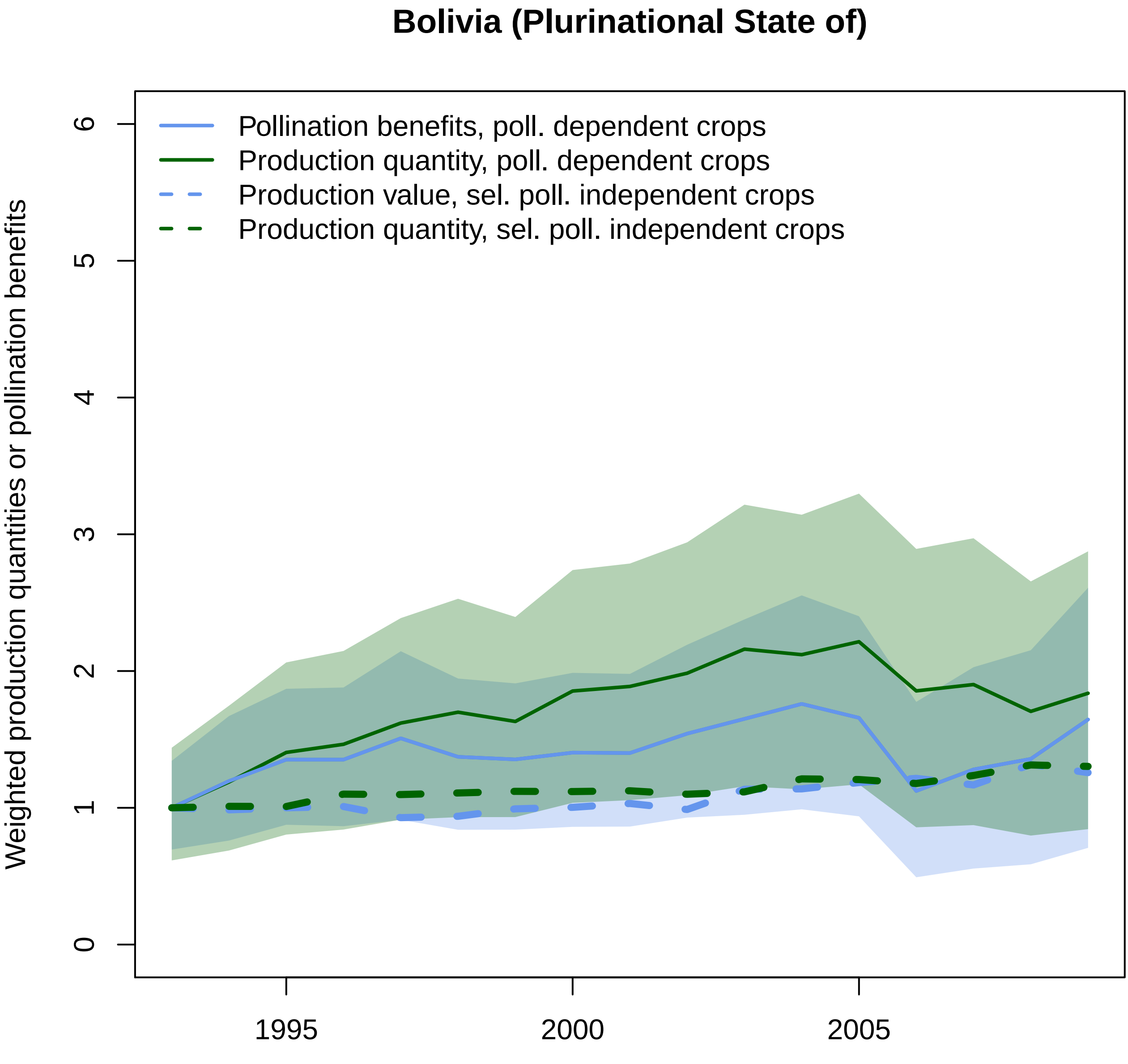

Bolivia (Plurinational State of)
Pollination benefits, poll. dependent crops
Production quantity, poll. dependent crops
Production value, sel. poll. independent crops
Production quantity, sel. poll. independent crops
Weighted production quantities or pollination benefits
0
1
2
3
4
5
6
1995
2000
2005

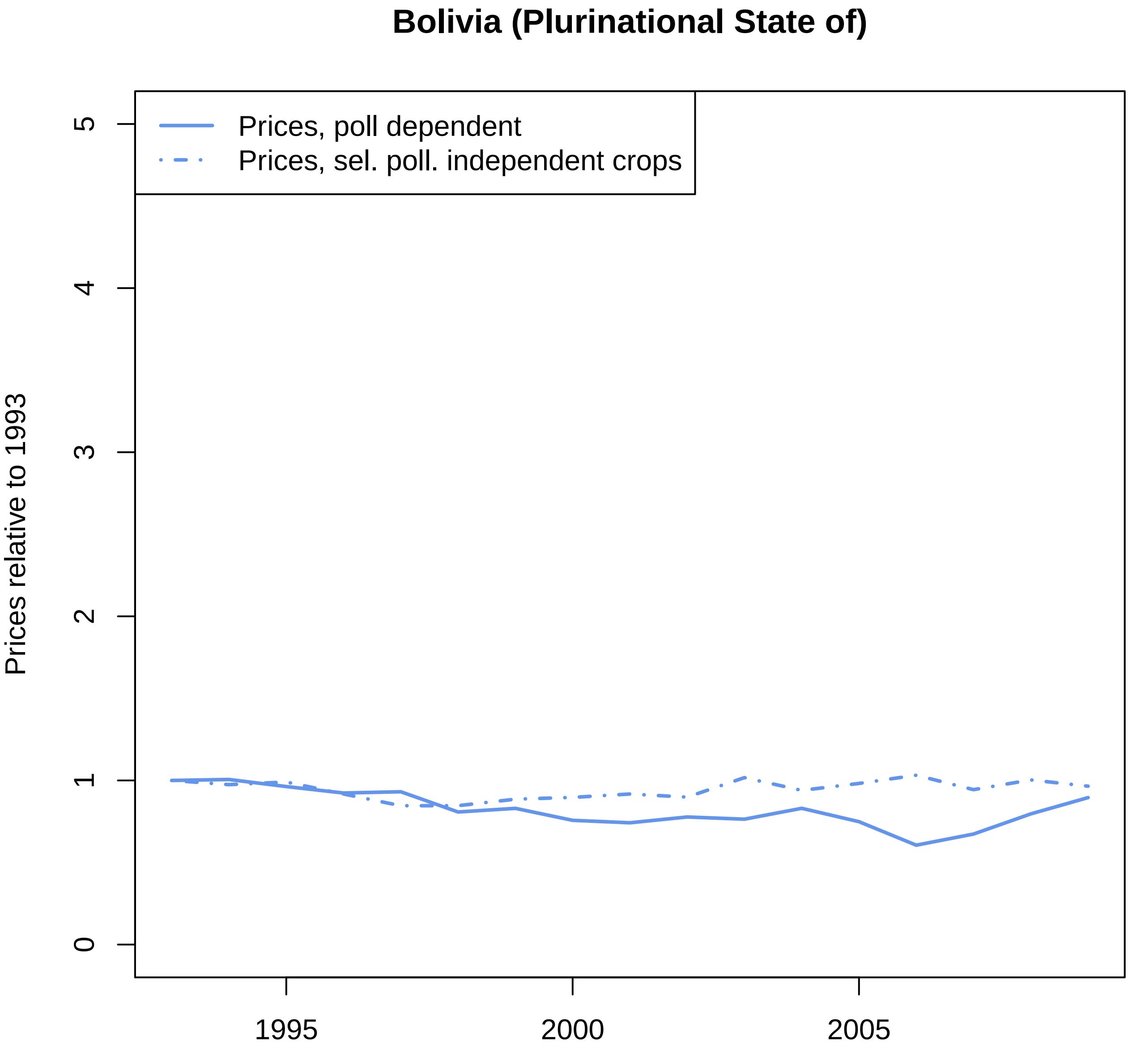
Bolivia (Plurinational State of)
Prices, poll dependent
Prices, sel. poll. independent crops
Prices relative to 1993
0
1
2
3
4
5
1995
2000
2005

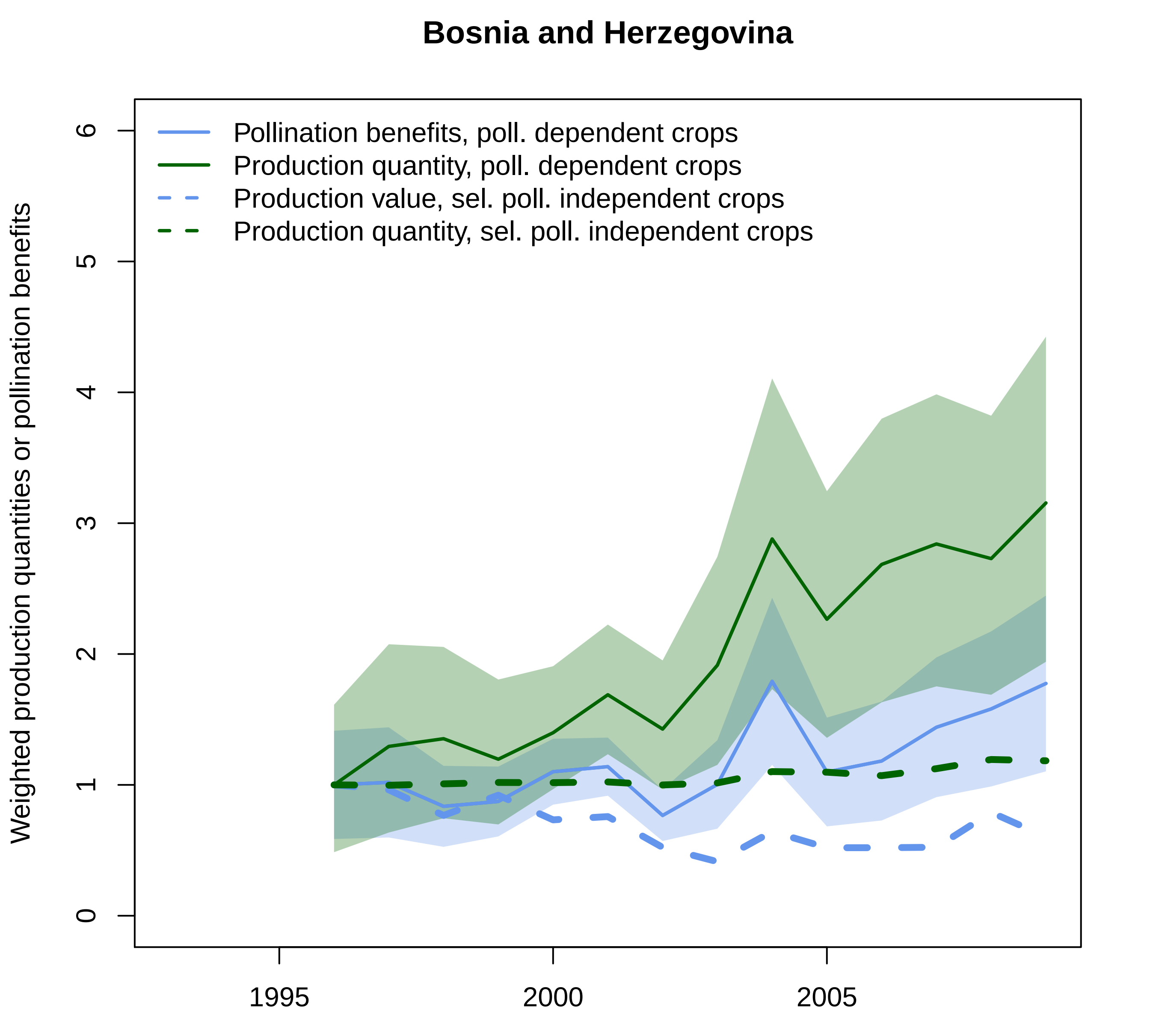
Bosnia and Herzegovina
Pollination benefits, poll. dependent crops
Production quantity, poll. dependent crops
Production value, sel. poll. independent crops
Production quantity, sel. poll. independent crops
Weighted production quantities or pollination benefits
0
1
2
3
4
5
6
1995
2000
2005

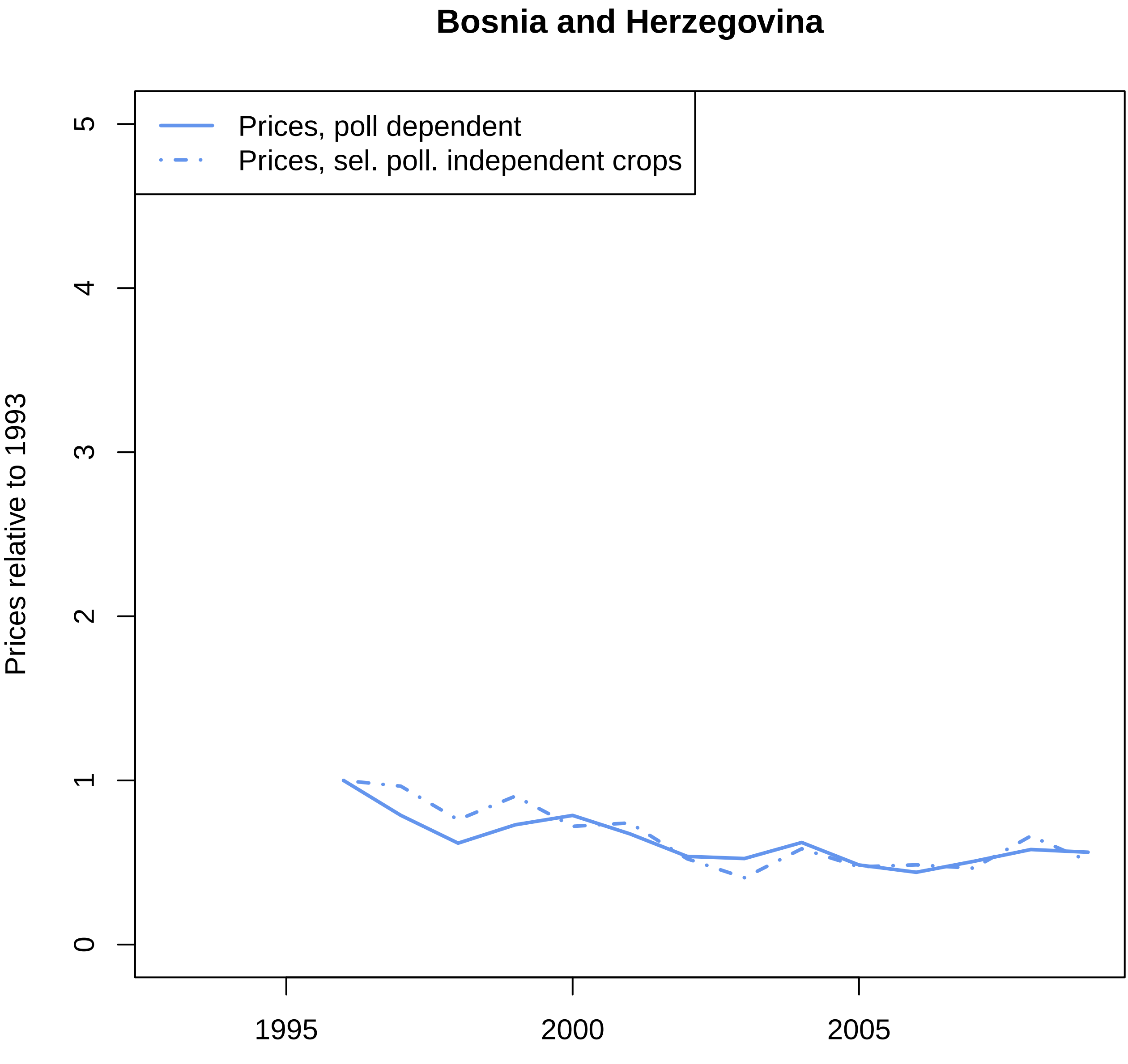

Bosnia and Herzegovina
Prices, poll dependent
Prices, sel. poll. independent crops
Prices relative to 1993
0
1
2
3
4
5
1995
2000
2005

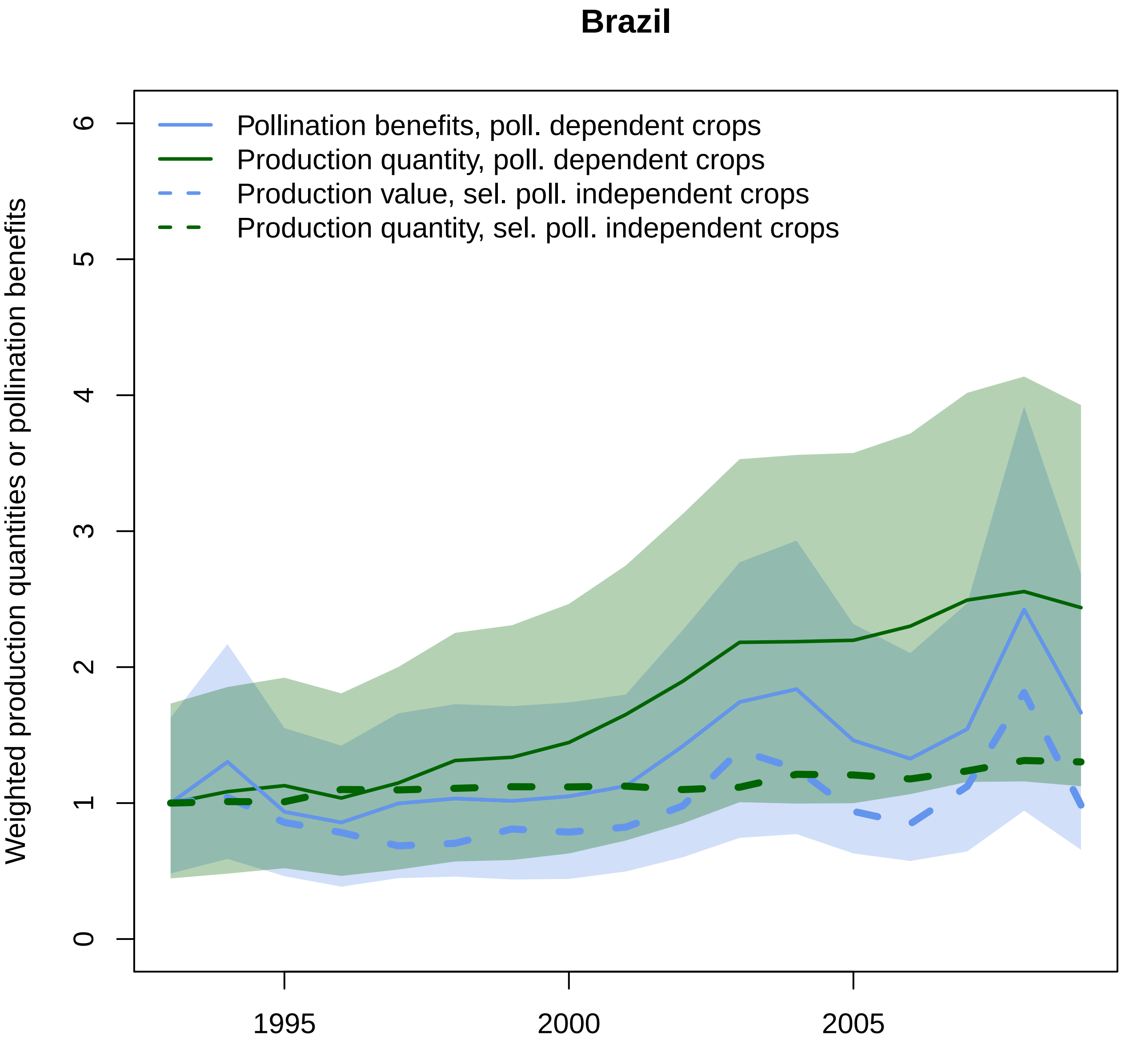
Brazil
Pollination benefits, poll. dependent crops
Production quantity, poll. dependent crops
Production value, sel. poll. independent crops
Production quantity, sel. poll. independent crops
Weighted production quantities or pollination benefits
0
1
2
3
4
5
6
1995
2000
2005

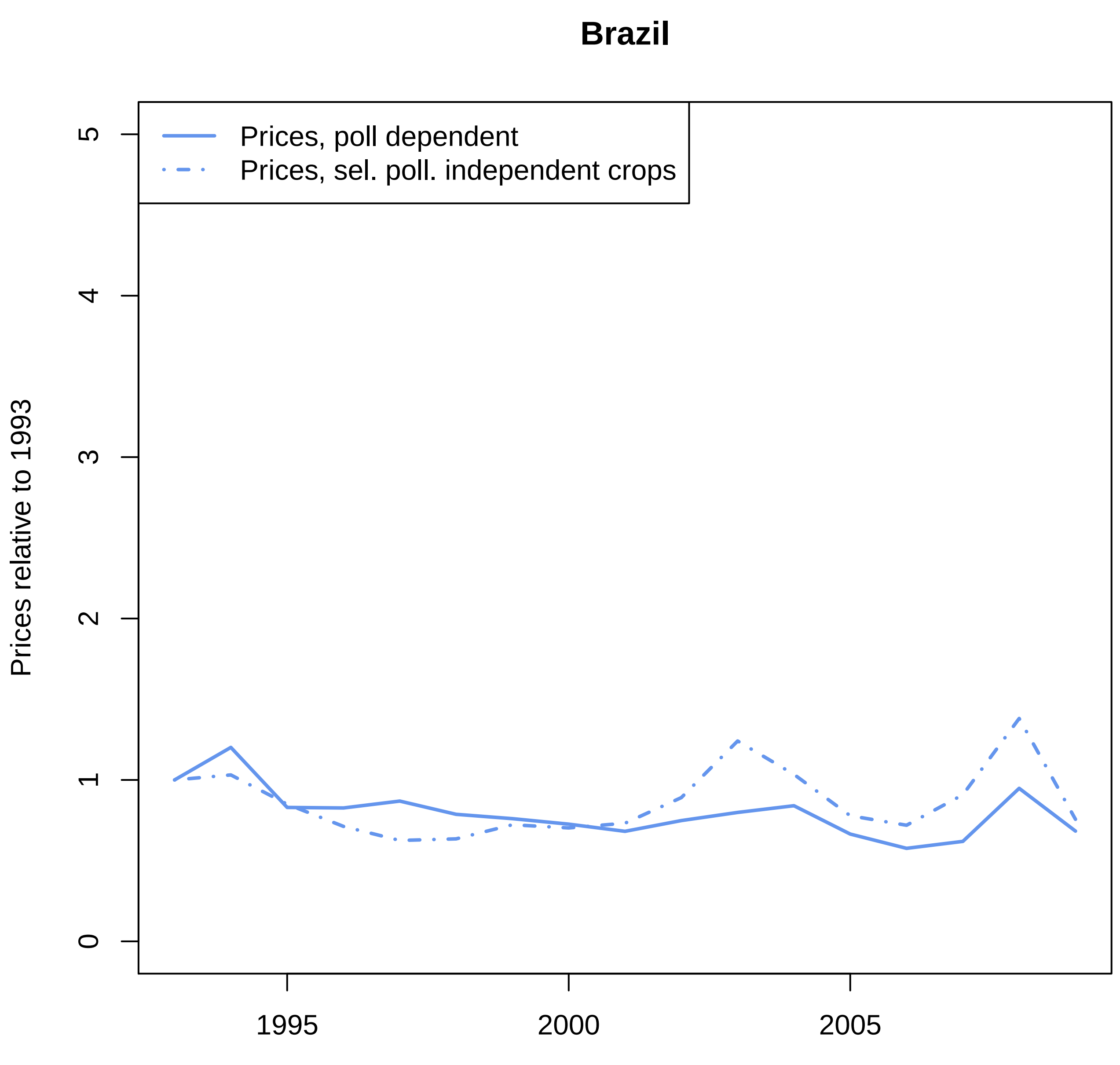
Brazil
Prices, poll dependent
Prices, sel. poll. independent crops
Prices relative to 1993
0
1
2
3
4
5
1995
2000
2005

Bulgaria

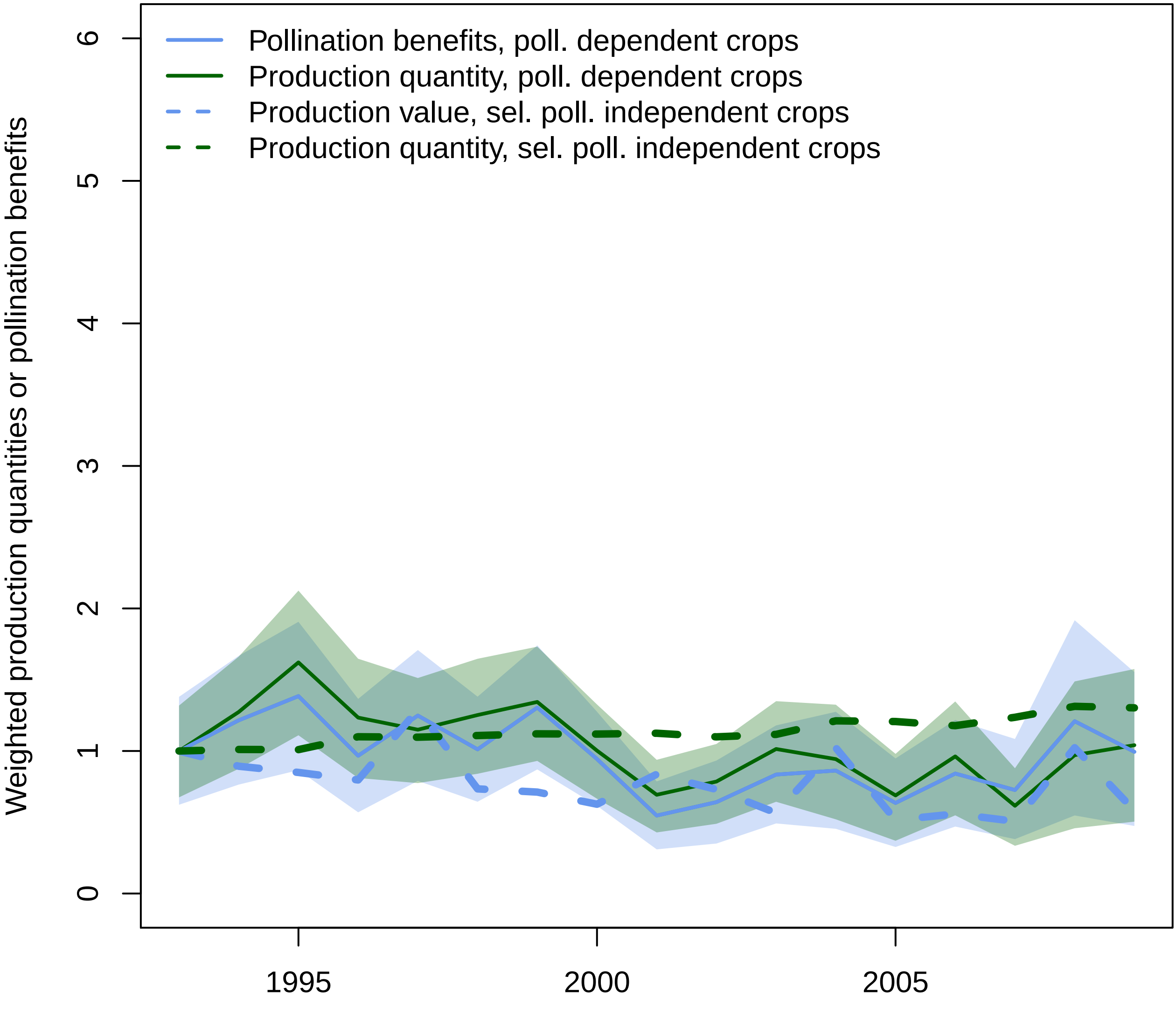

Pollination benefits, poll. dependent crops
Production quantity, poll. dependent crops
Production value, sel. poll. independent crops
Production quantity, sel. poll. independent crops
Weighted production quantities or pollination benefits
0
1
2
3
4
5
6
1995
2000
2005

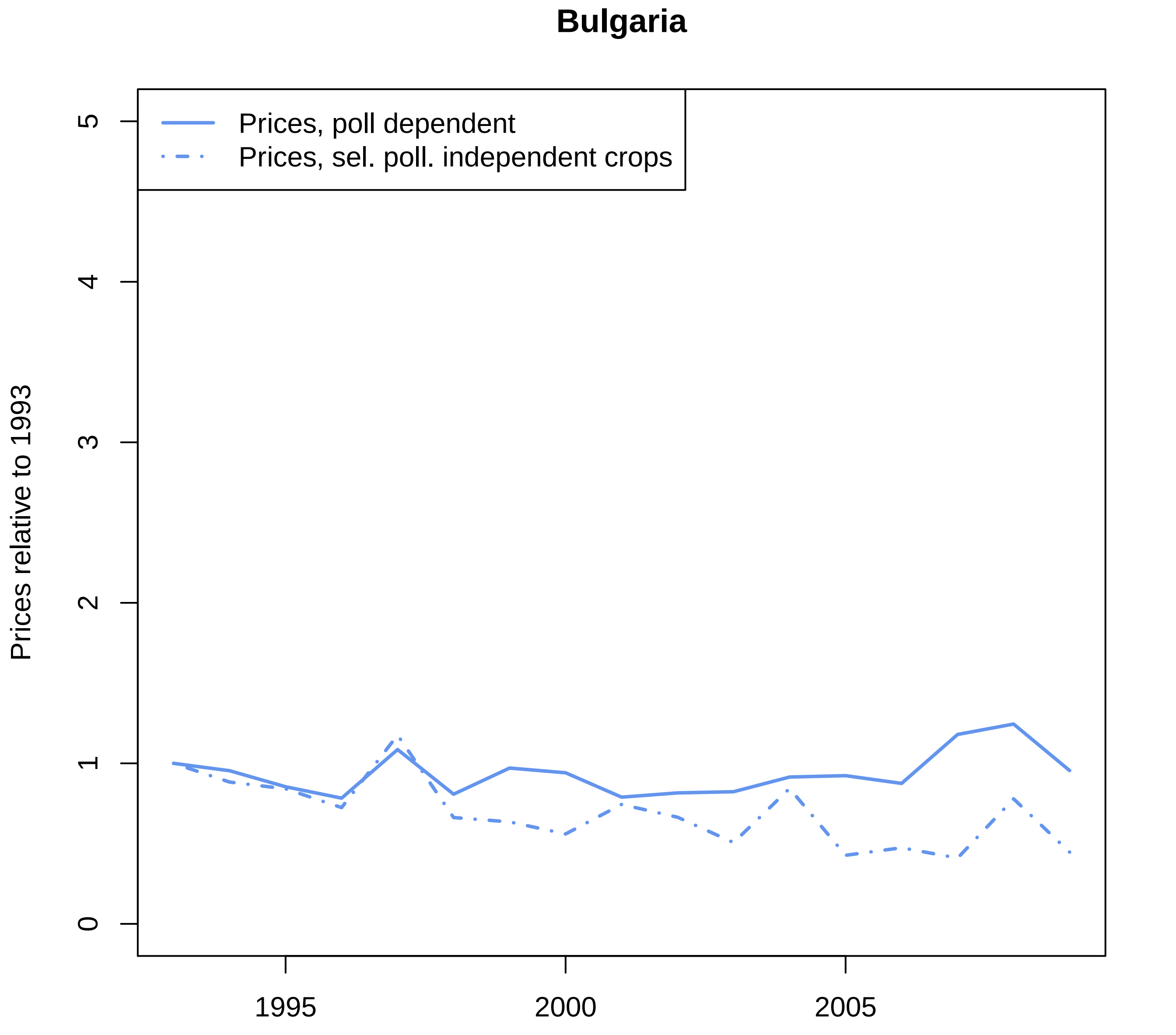
Bulgaria
Prices, poll dependent
Prices, sel. poll. independent crops
Prices relative to 1993
0
1
2
3
4
5
1995
2000
2005

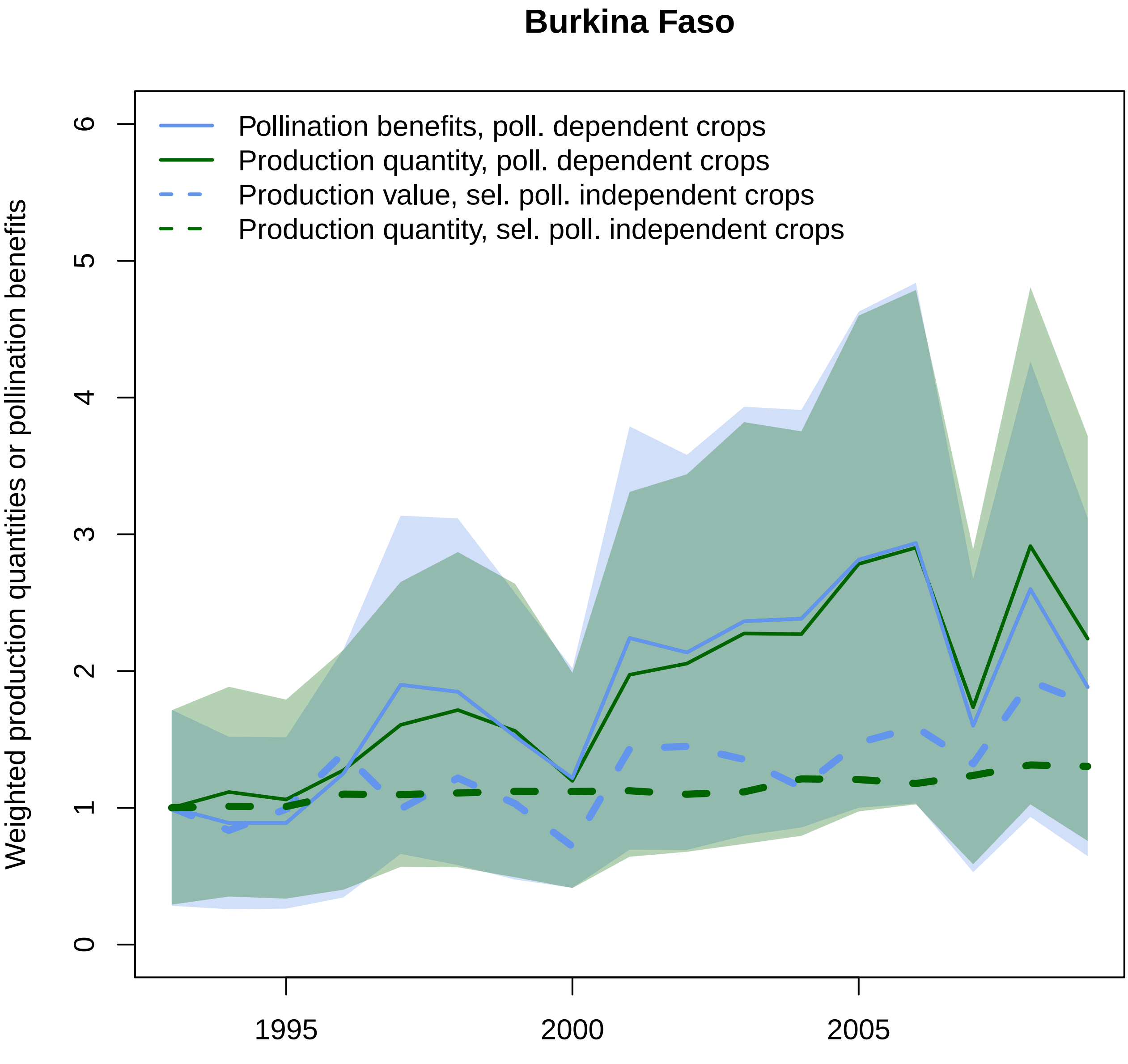

Burkina Faso
Pollination benefits, poll. dependent crops
Production quantity, poll. dependent crops
Production value, sel. poll. independent crops
Production quantity, sel. poll. independent crops
Weighted production quantities or pollination benefits
0
1
2
3
4
5
6
1995
2000
2005

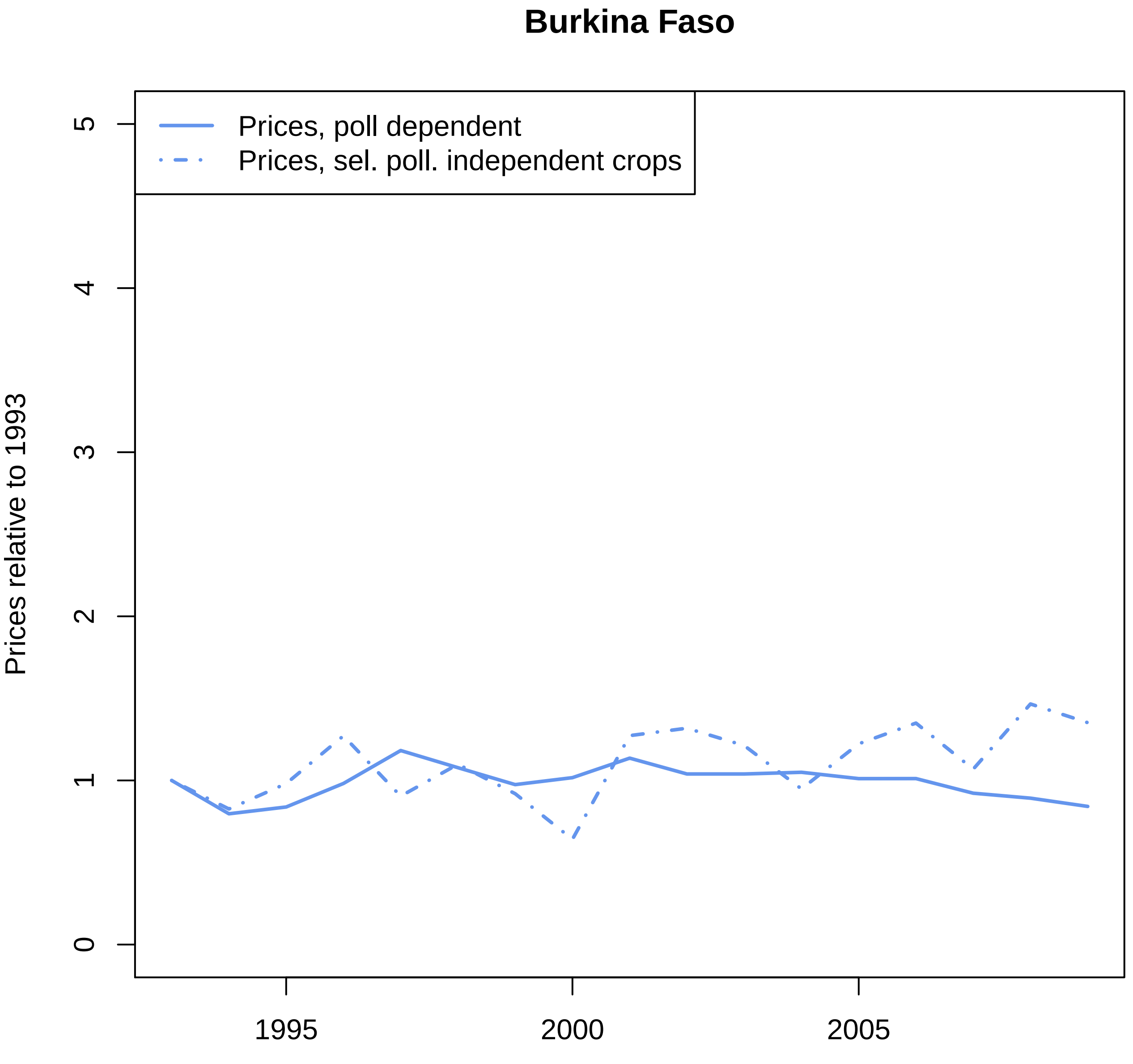

Burkina Faso
Prices, poll dependent
Prices, sel. poll. independent crops
Prices relative to 1993
0
1
2
3
4
5
1995
2000
2005

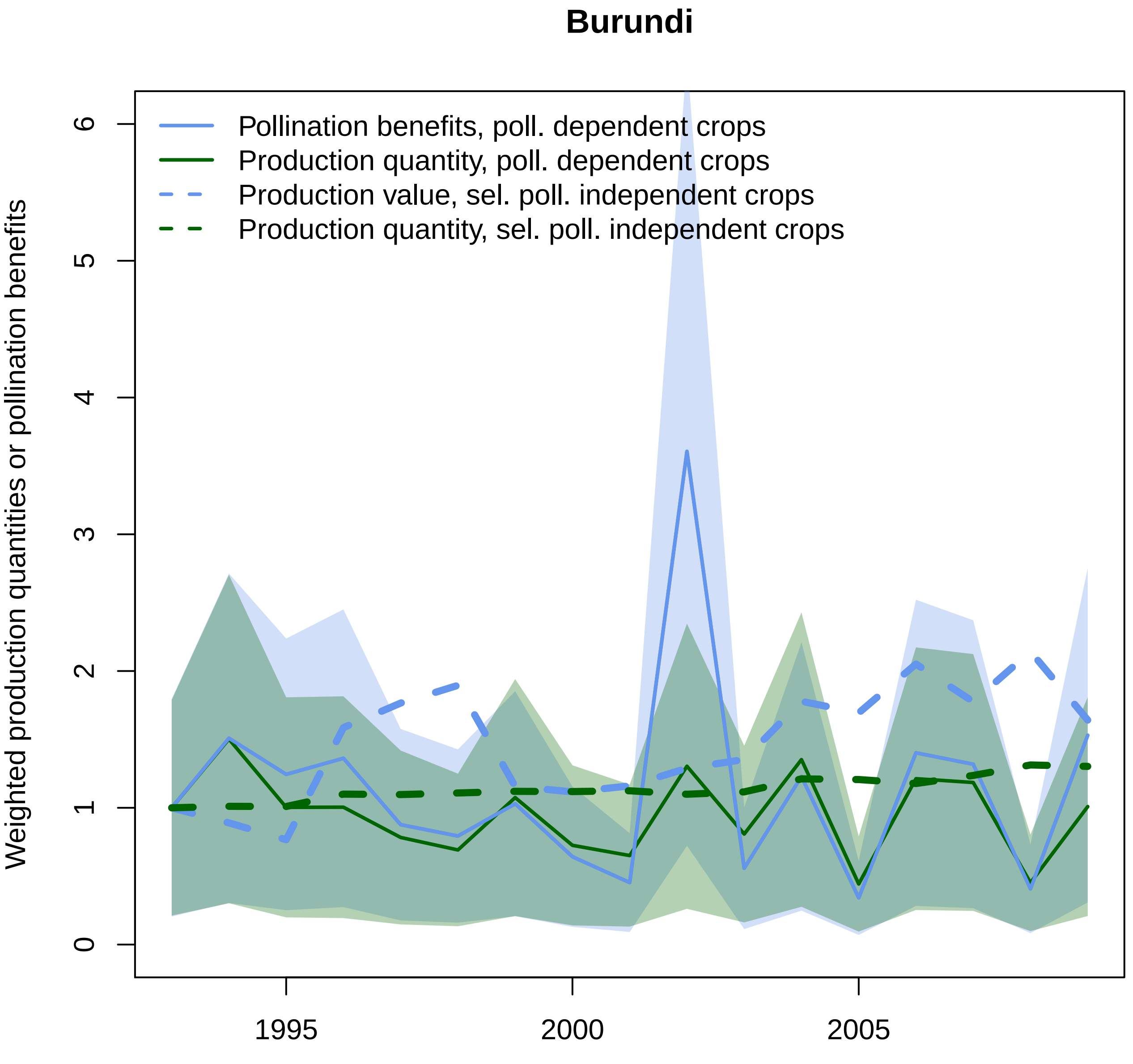

Burundi
Pollination benefits, poll. dependent crops
Production quantity, poll. dependent crops
Production value, sel. poll. independent crops
Production quantity, sel. poll. independent crops
Weighted production quantities or pollination benefits
0
1
2
3
4
5
6
1995
2000
2005

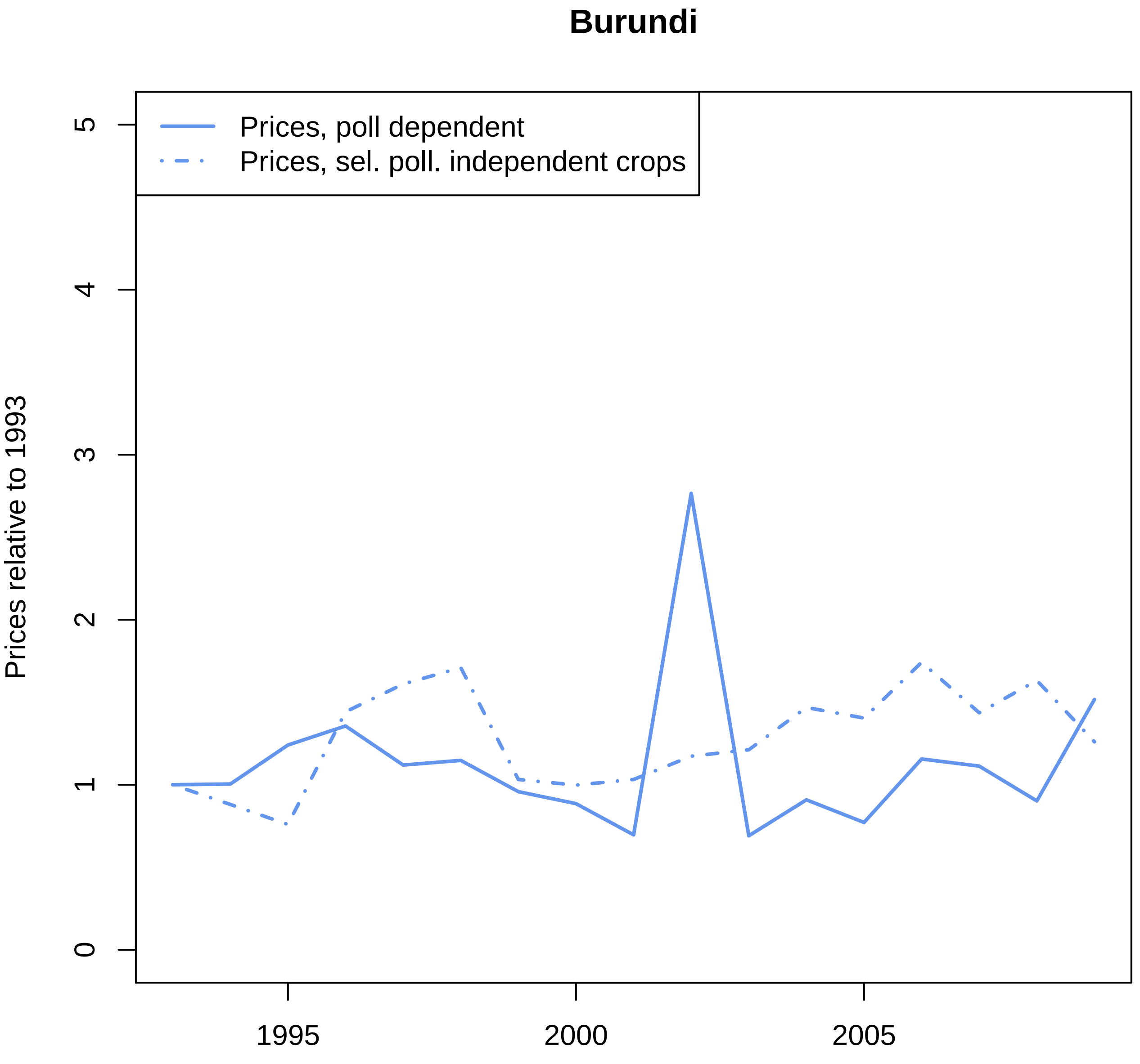
Burundi
Prices, poll dependent
Prices, sel. poll. independent crops
Prices relative to 1993
0
1
2
3
4
5
1995
2000
2005

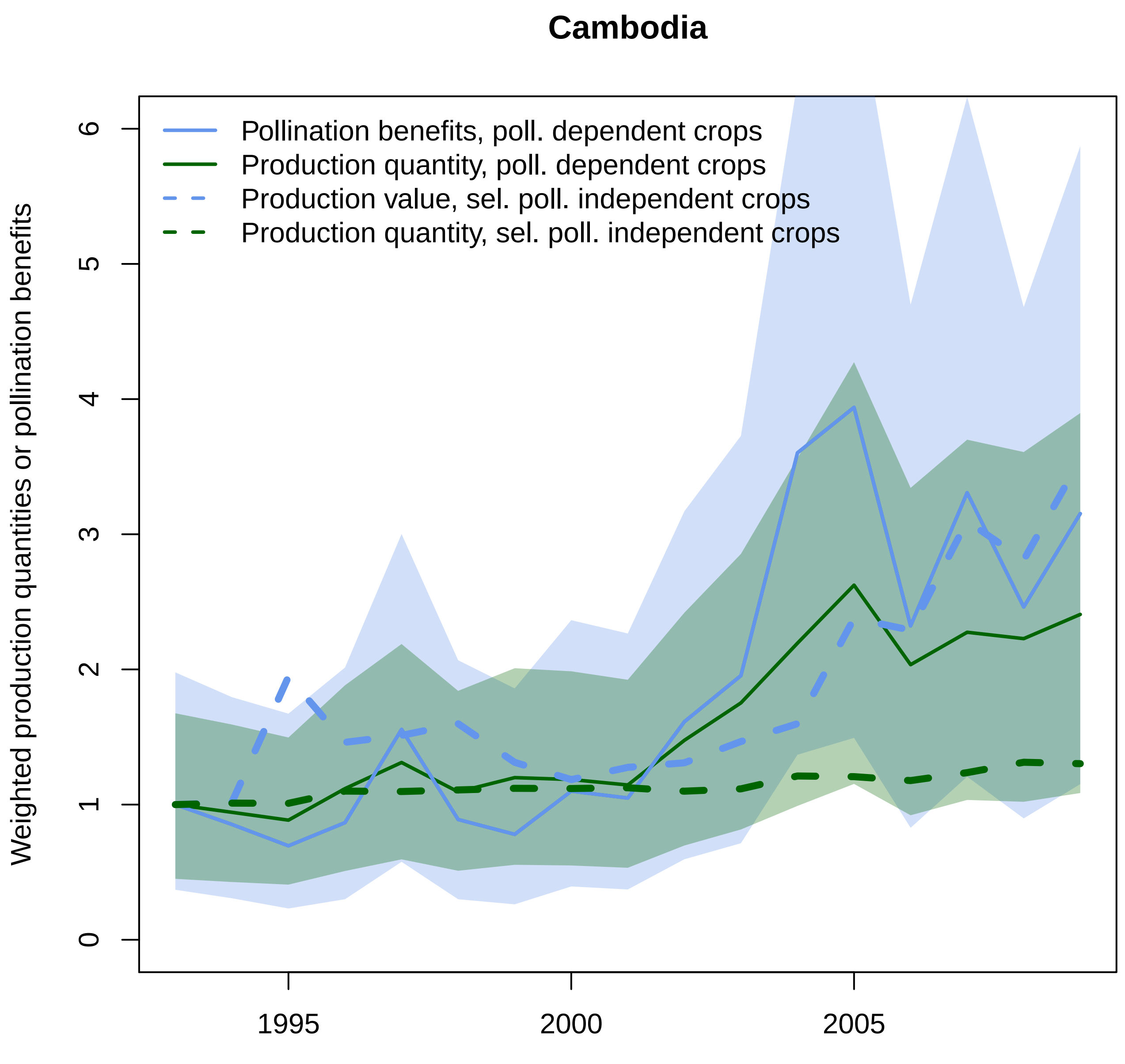
Cambodia
Pollination benefits, poll. dependent crops
Production quantity, poll. dependent crops
Production value, sel. poll. independent crops
Production quantity, sel. poll. independent crops
Weighted production quantities or pollination benefits
0
1
2
3
4
5
6
1995
2000
2005

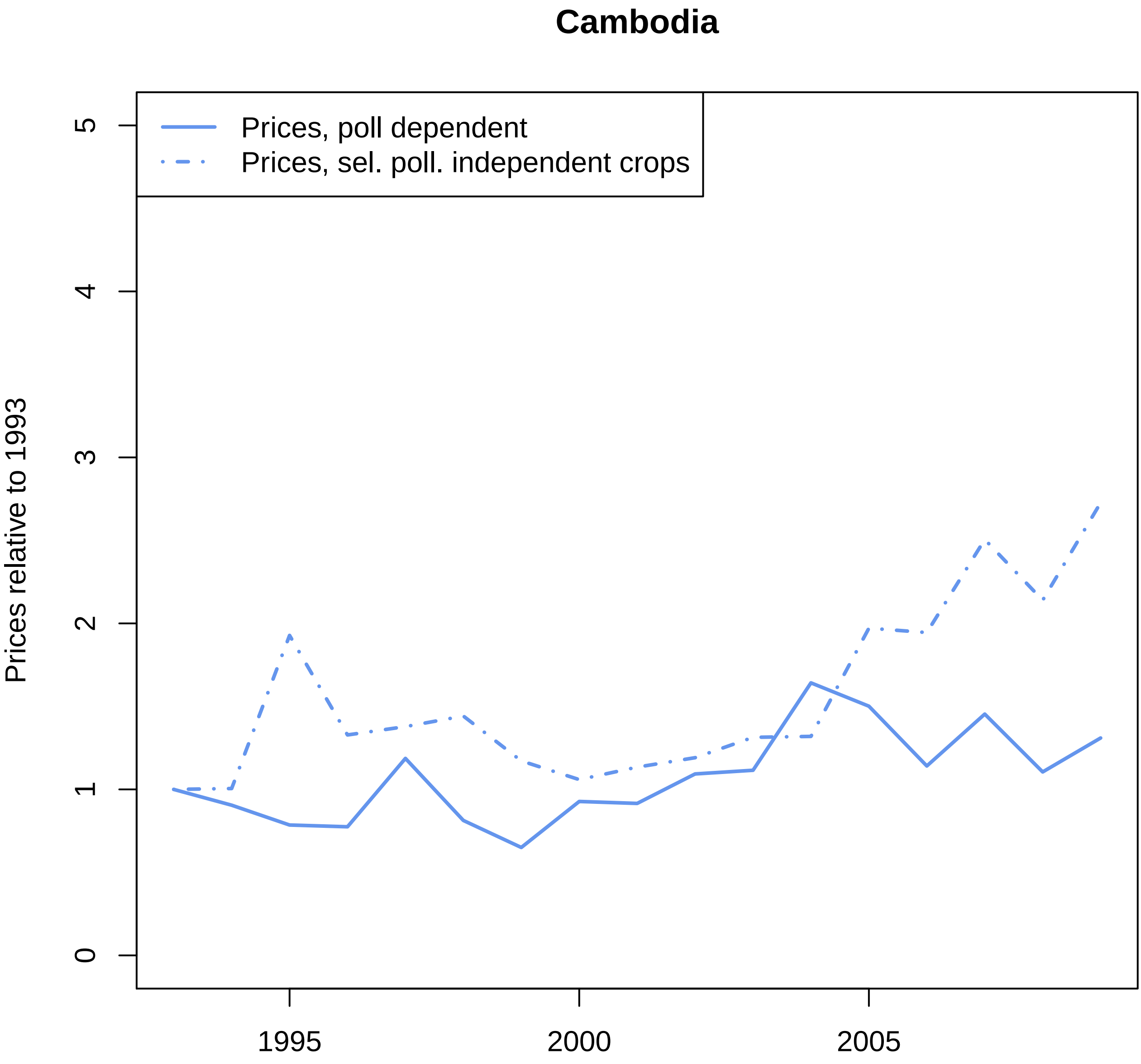
Cambodia
Prices, poll dependent
Prices, sel. poll. independent crops
Prices relative to 1993
0
1
2
3
4
5
1995
2000
2005

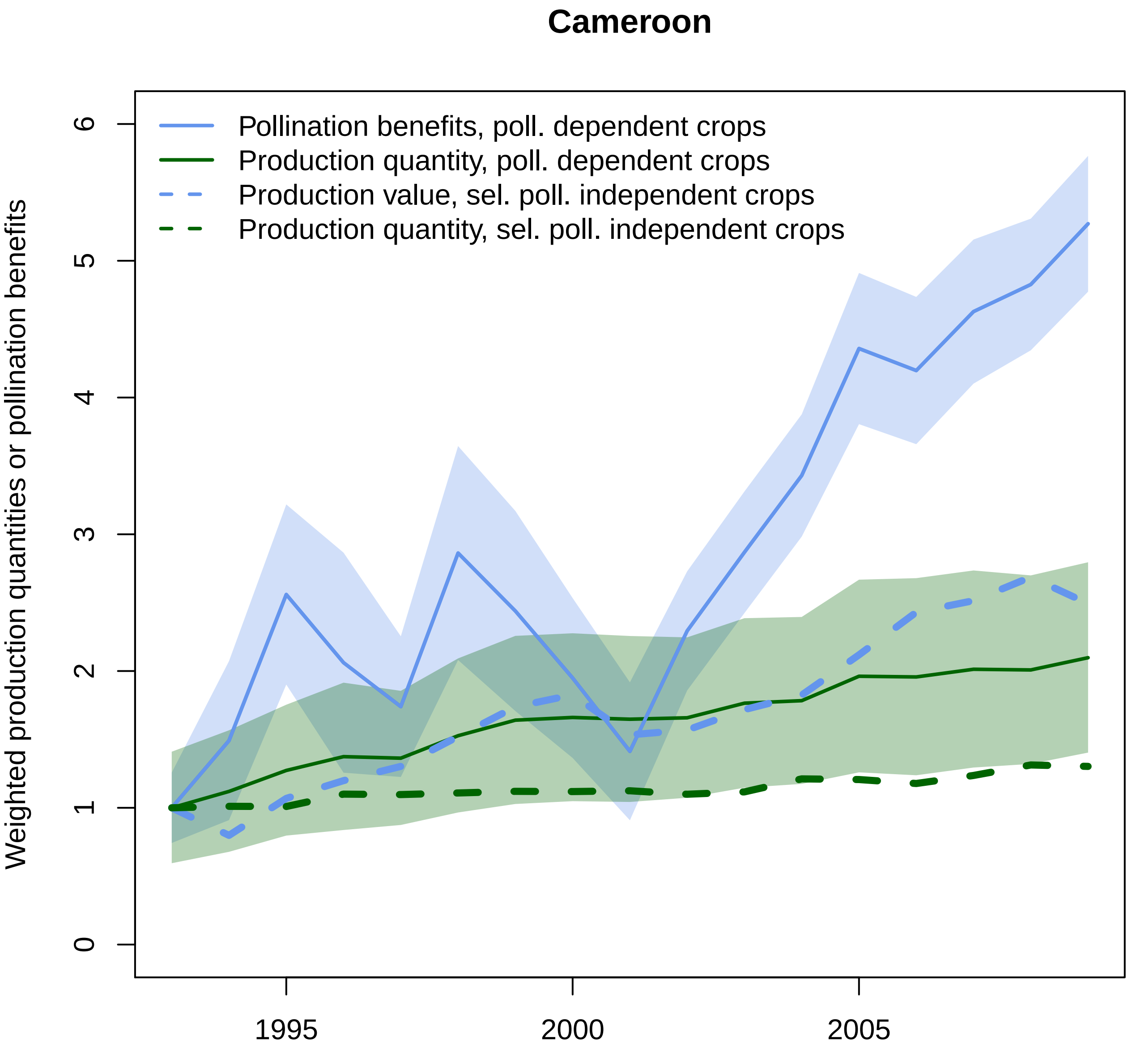
Cameroon
Pollination benefits, poll. dependent crops
Production quantity, poll. dependent crops
Production value, sel. poll. independent crops
Production quantity, sel. poll. independent crops
Weighted production quantities or pollination benefits
0
1
2
3
4
5
6
1995
2000
2005

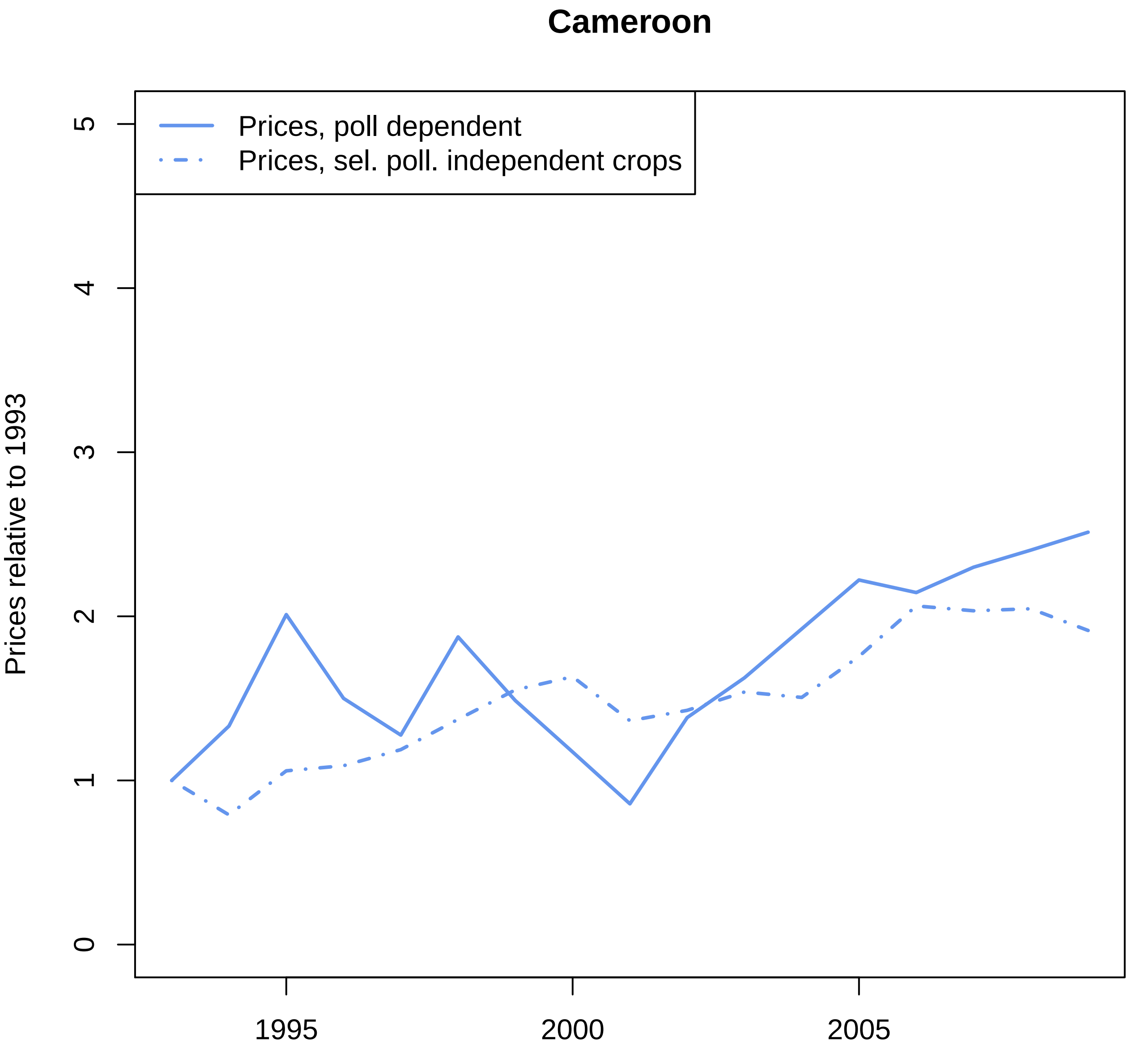
Cameroon
Prices, poll dependent
Prices, sel. poll. independent crops
Prices relative to 1993
0
1
2
3
4
5
1995
2000
2005

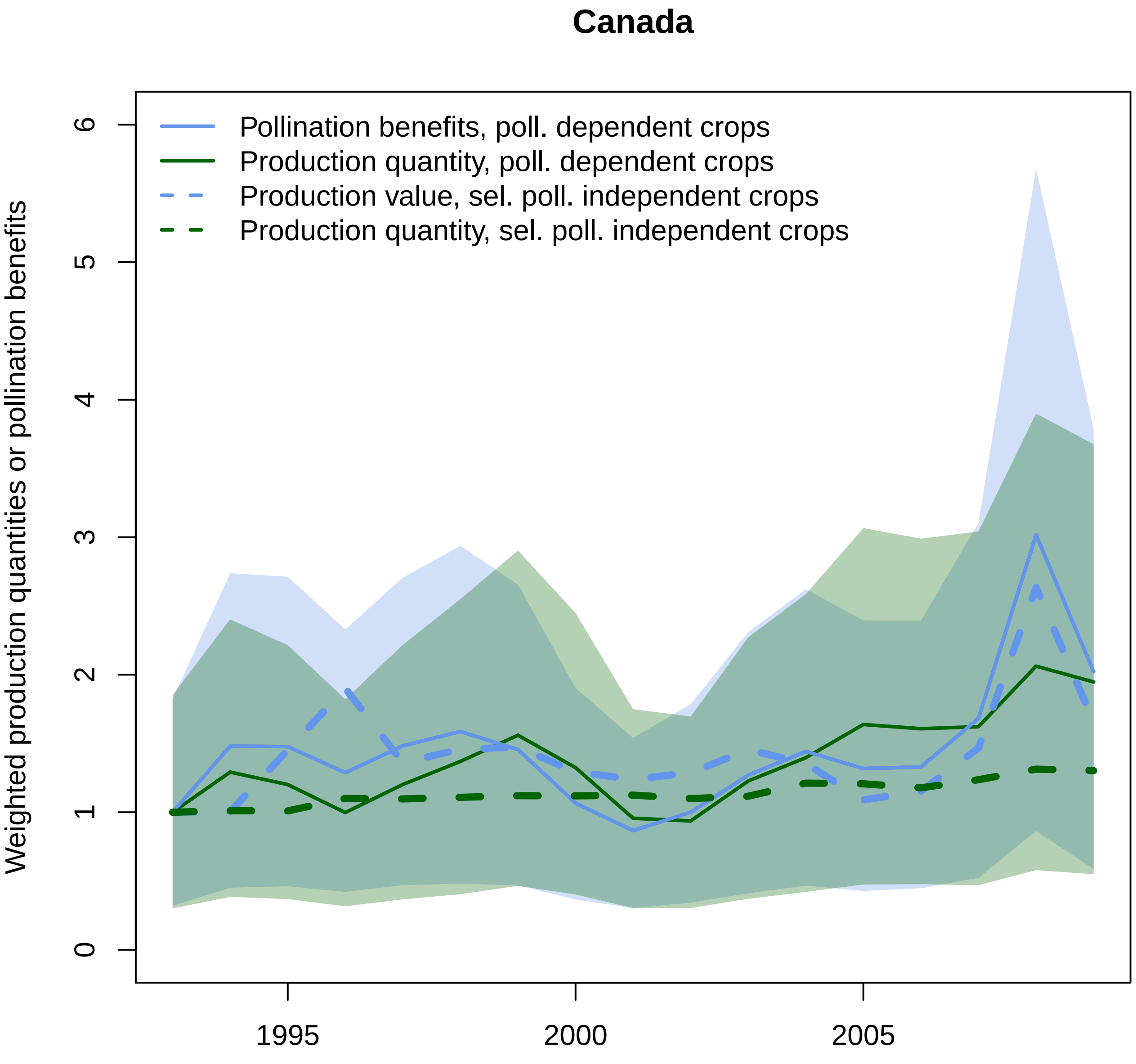
Canada
Pollination benefits, poll. dependent crops
Production quantity, poll. dependent crops
Production value, sel. poll. independent crops
Production quantity, sel. poll. independent crops
Weighted production quantities or pollination benefits
0
1
2
3
4
5
6
1995
2000
2005

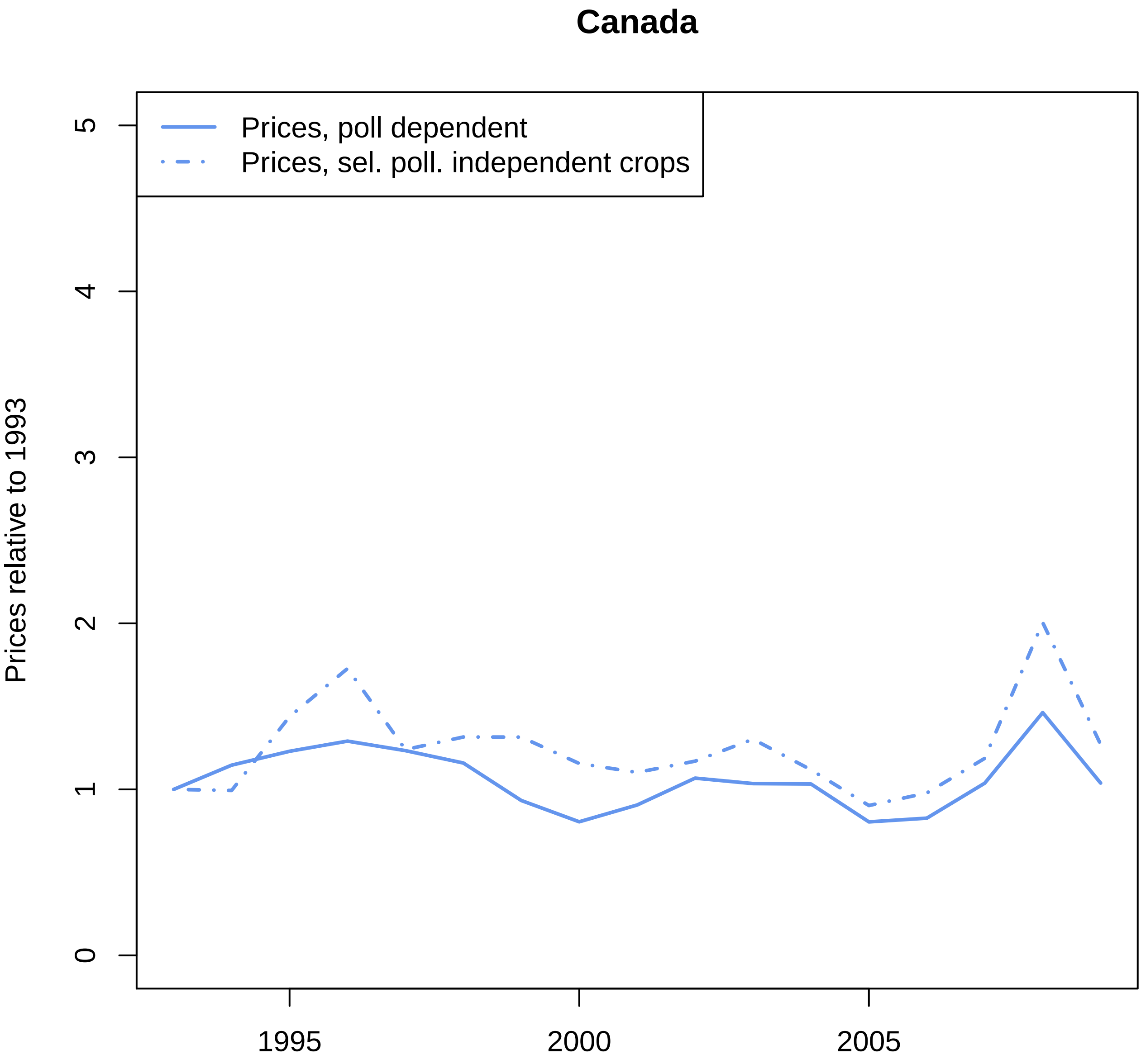
Canada
Prices, poll dependent
Prices, sel. poll. independent crops
Prices relative to 1993
0
1
2
3
4
5
1995
2000
2005

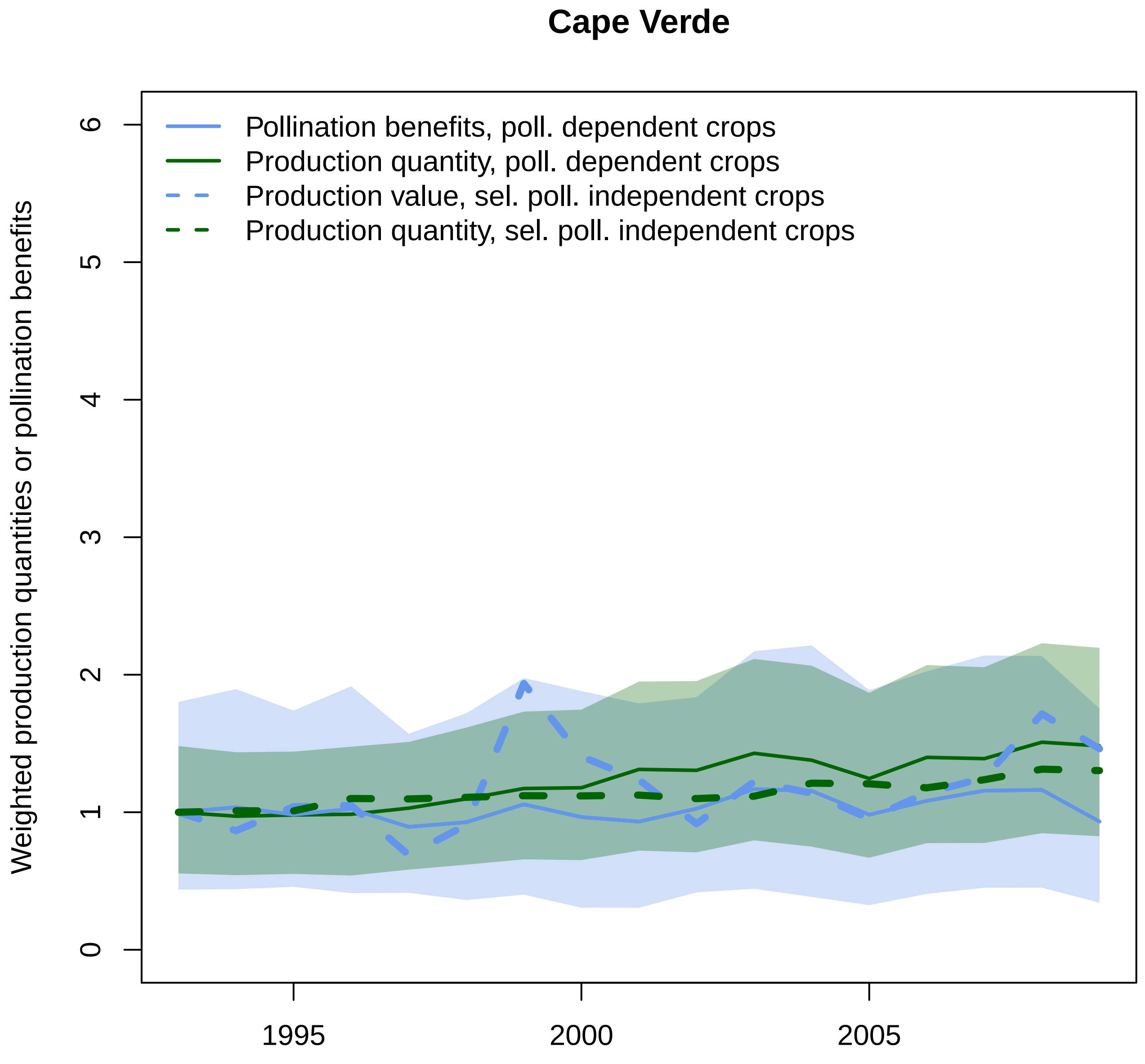

Cape Verde
Pollination benefits, poll. dependent crops
Production quantity, poll. dependent crops
Production value, sel. poll. independent crops
Production quantity, sel. poll. independent crops
Weighted production quantities or pollination benefits
0
1
2
3
4
5
6
1995
2000
2005

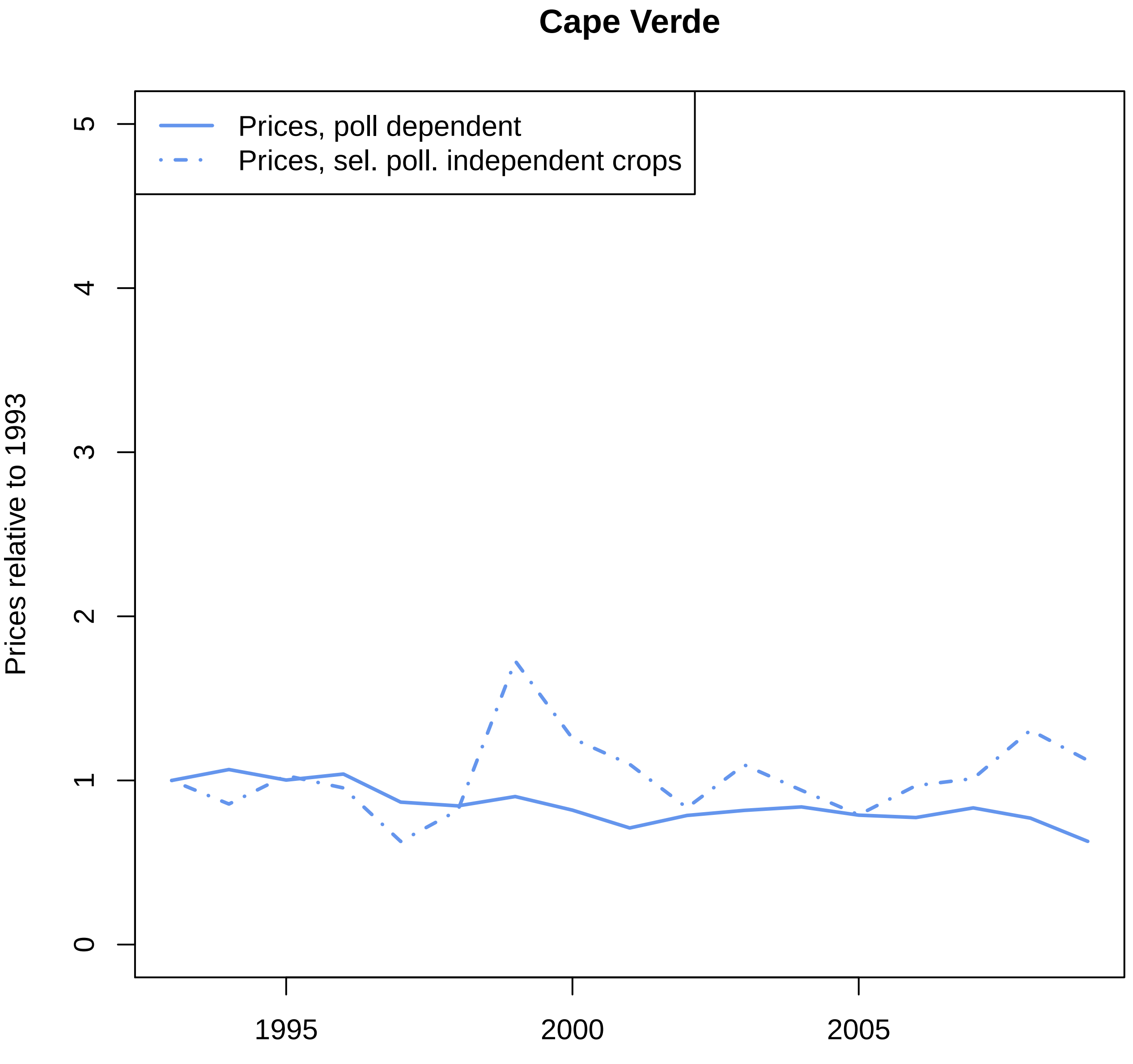
Cape Verde
Prices, poll dependent
Prices, sel. poll. independent crops
Prices relative to 1993
0
1
2
3
4
5
1995
2000
2005

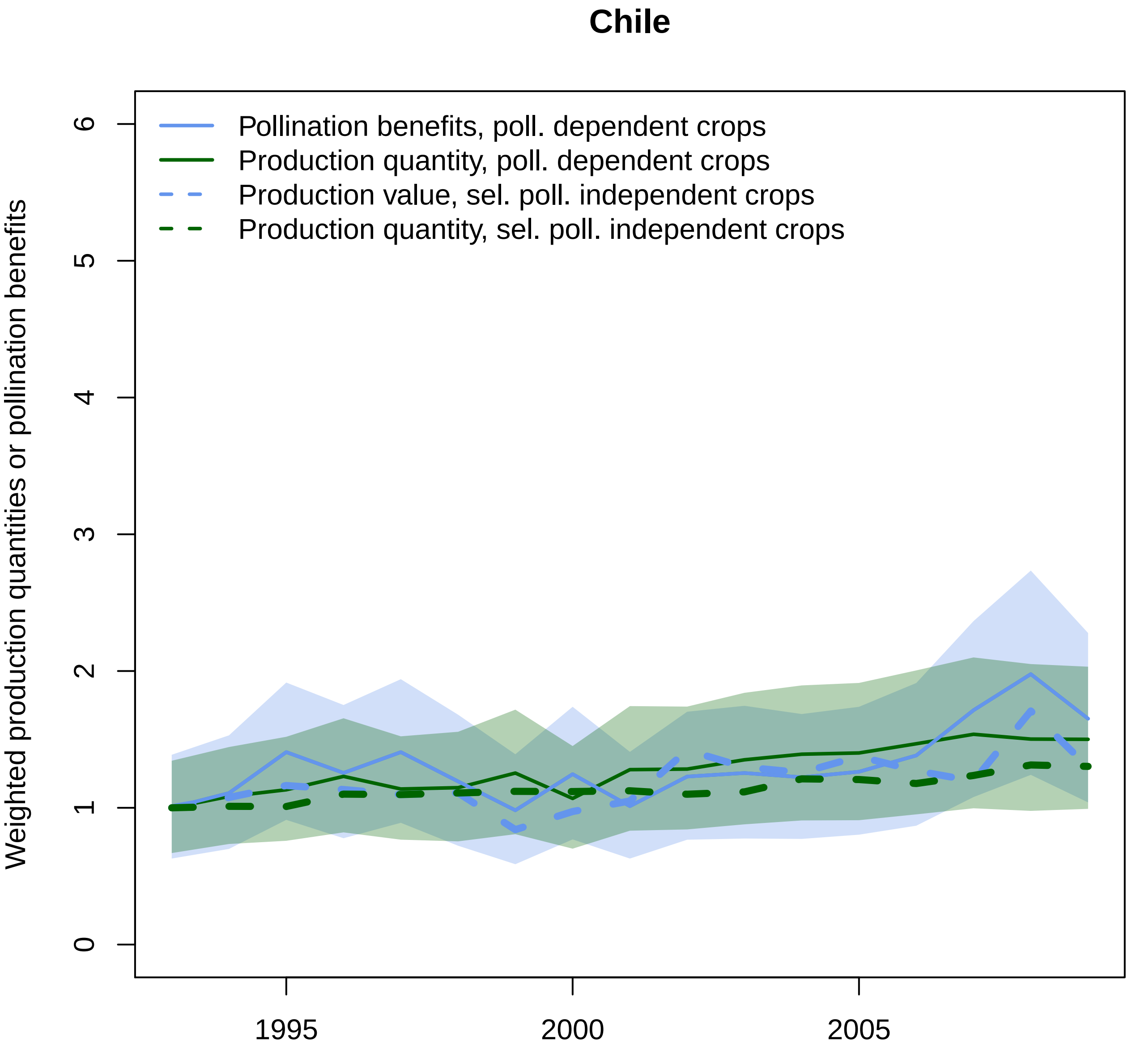
Chile
Pollination benefits, poll. dependent crops
Production quantity, poll. dependent crops
Production value, sel. poll. independent crops
Production quantity, sel. poll. independent crops
Weighted production quantities or pollination benefits
0
1
2
3
4
5
6
1995
2000
2005

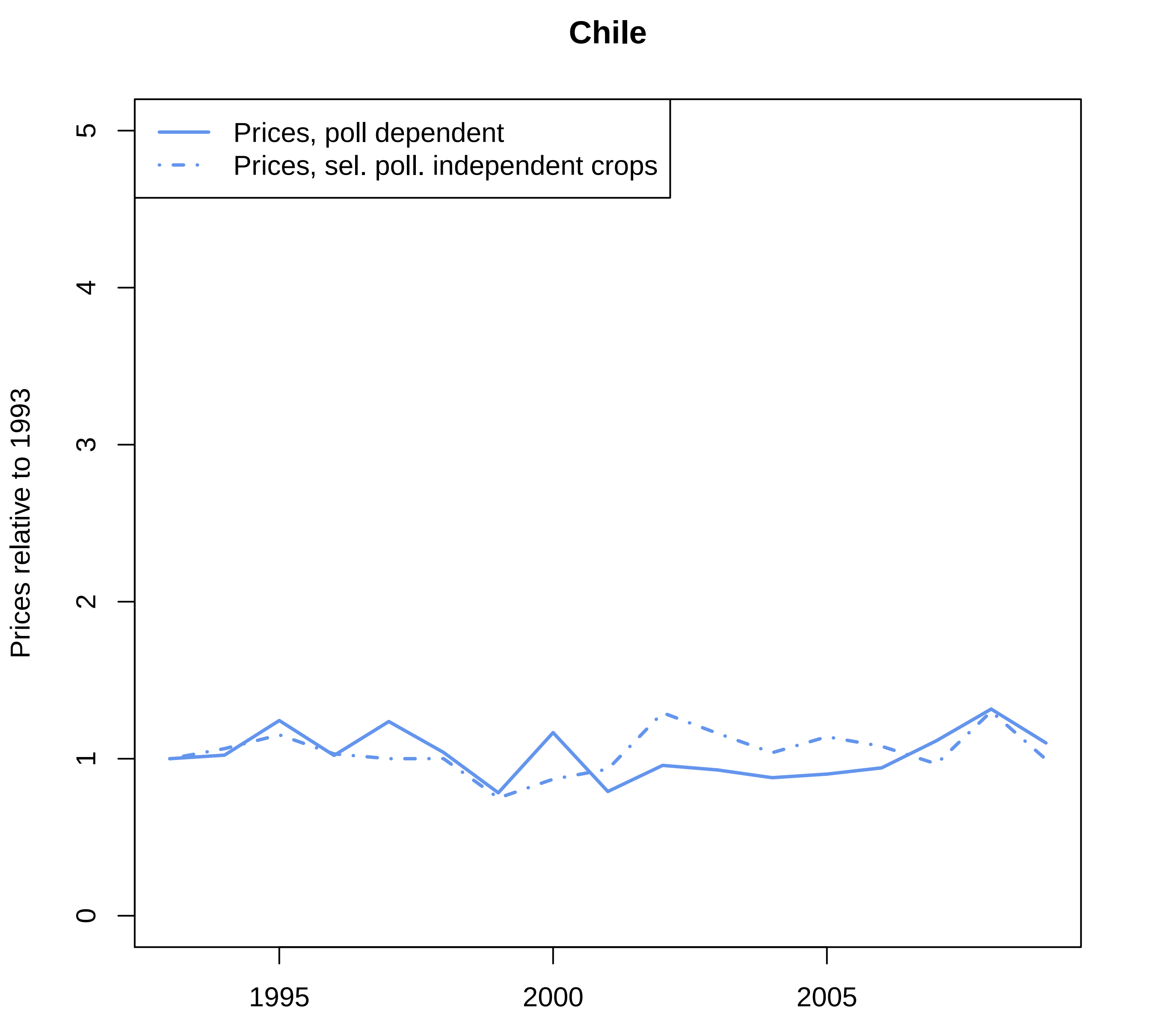
Chile
Prices, poll dependent
Prices, sel. poll. independent crops
Prices relative to 1993
0
1
2
3
4
5
1995
2000
2005

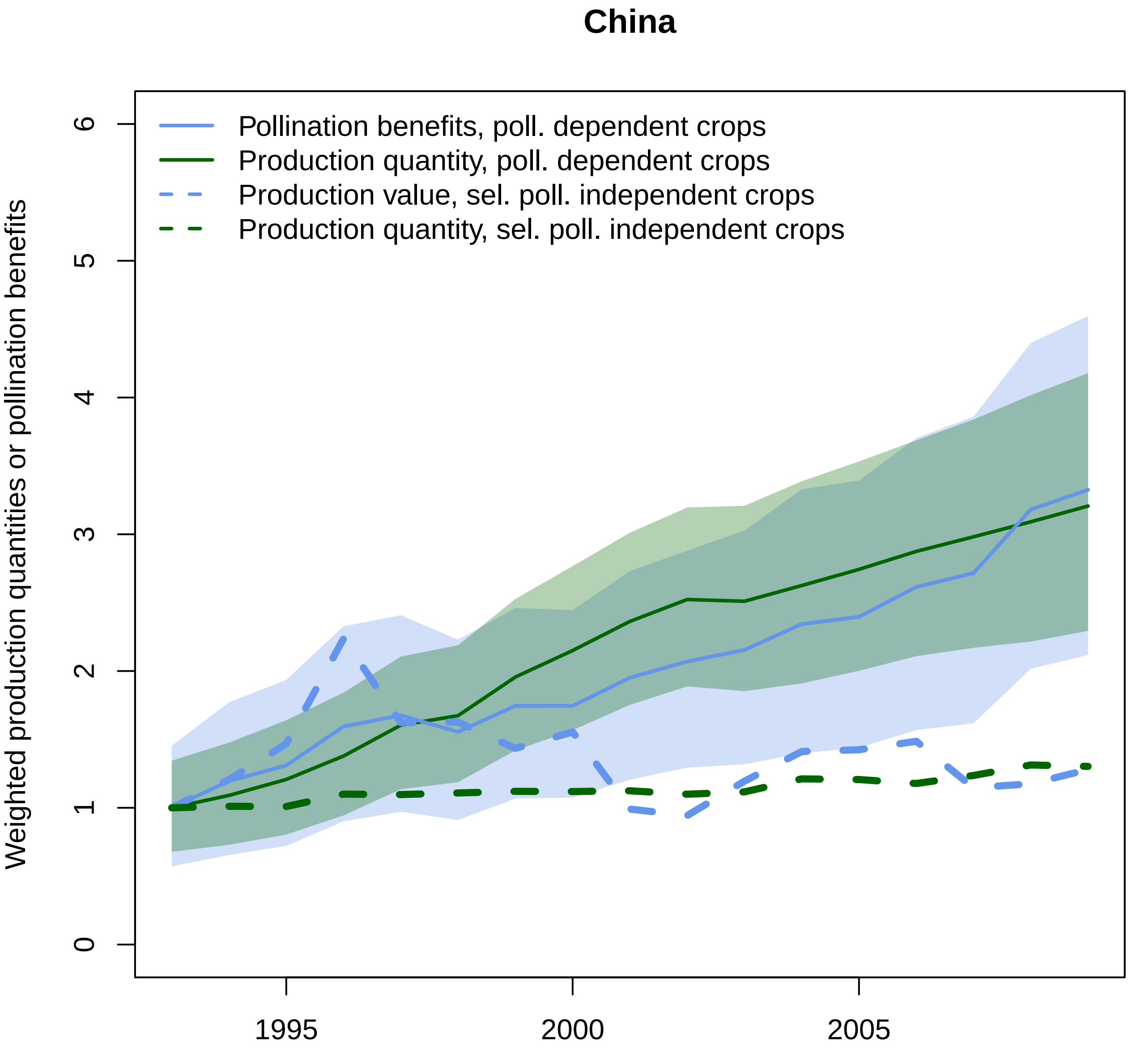
China
Pollination benefits, poll. dependent crops
Production quantity, poll. dependent crops
Production value, sel. poll. independent crops
Production quantity, sel. poll. independent crops
Weighted production quantities or pollination benefits
0
1
2
3
4
5
6
1995
2000
2005

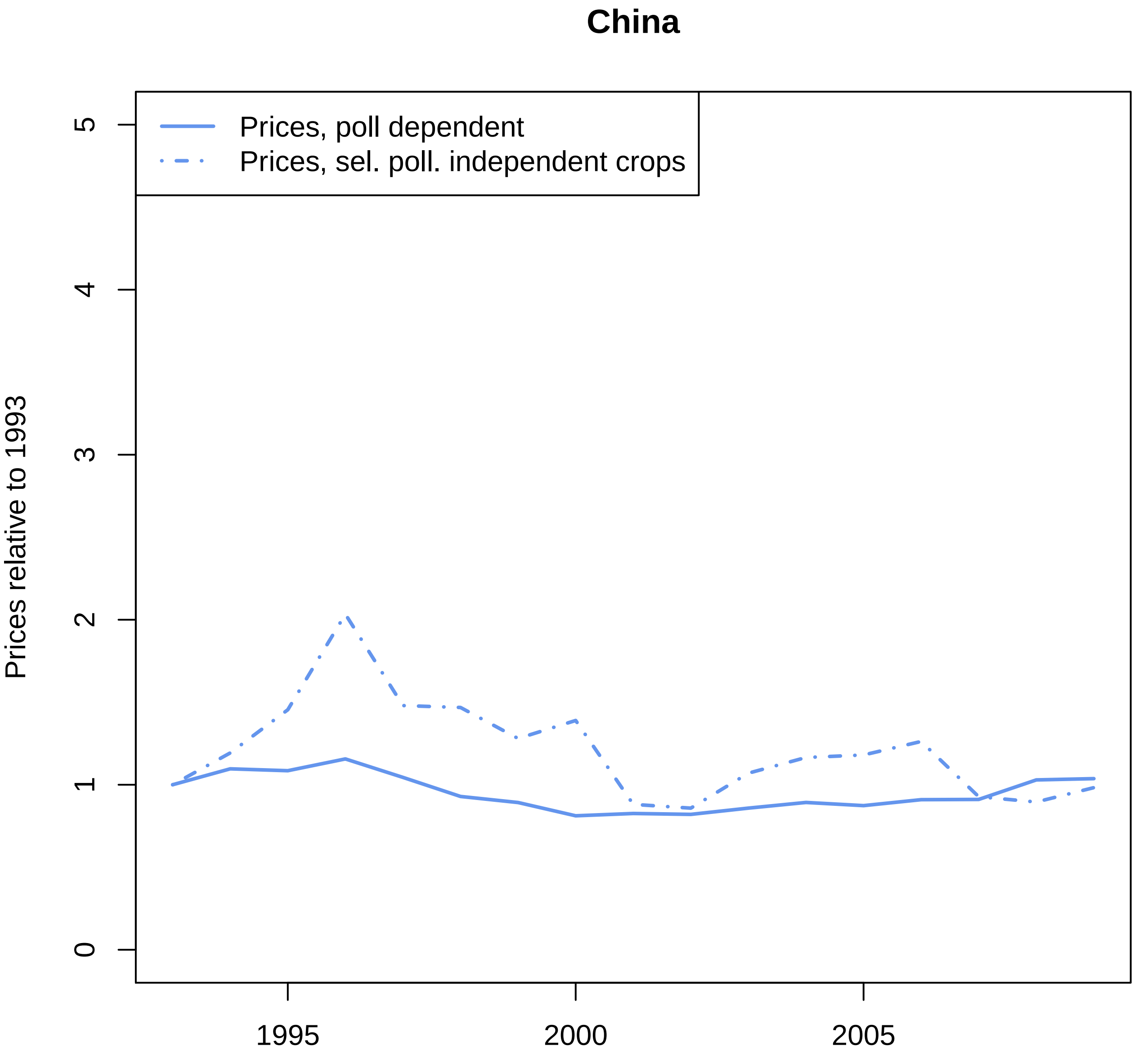
China
Prices, poll dependent
Prices, sel. poll. independent crops
Prices relative to 1993
0
1
2
3
4
5
1995
2000
2005

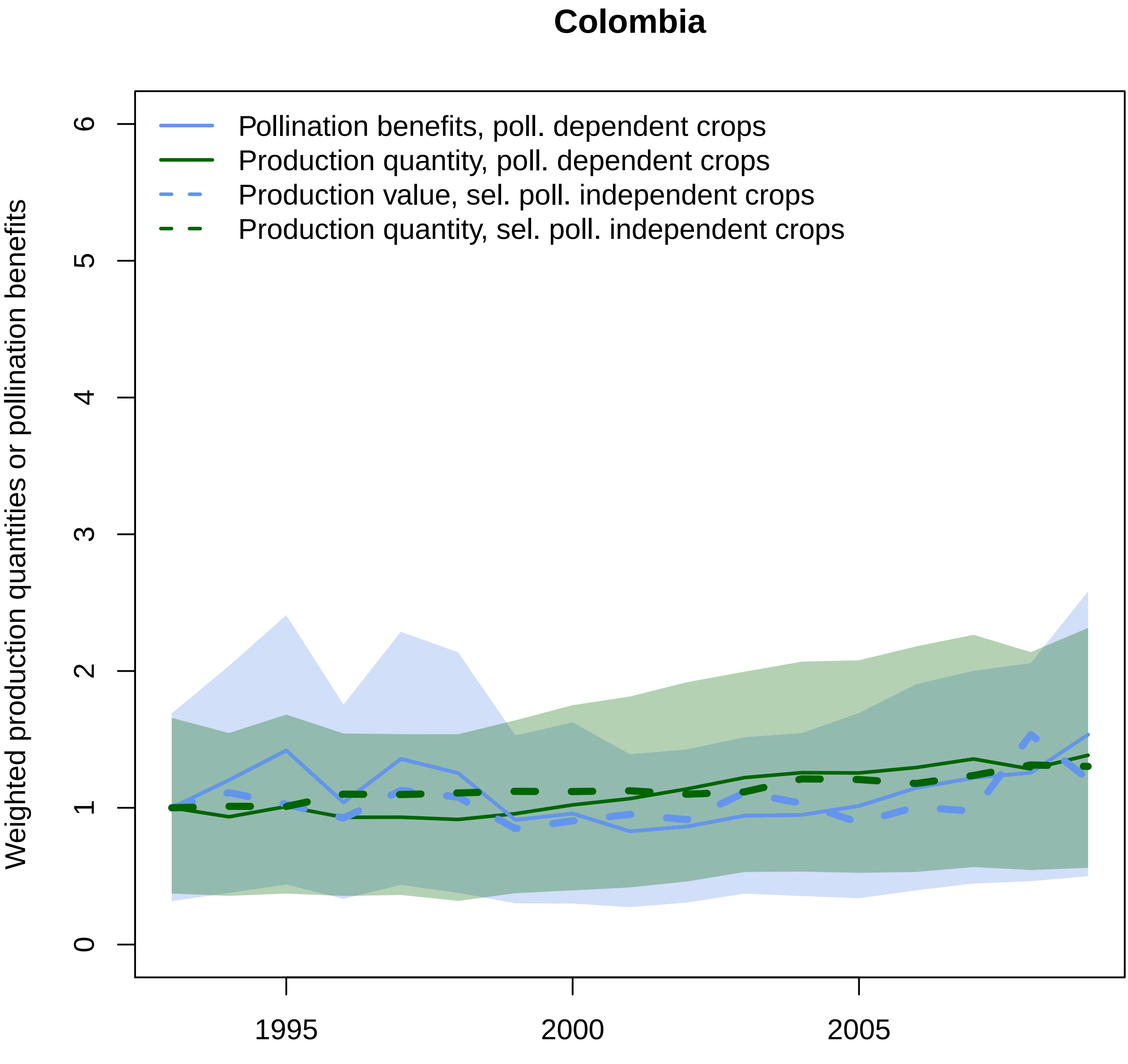

Colombia
Pollination benefits, poll. dependent crops
Production quantity, poll. dependent crops
Production value, sel. poll. independent crops
Production quantity, sel. poll. independent crops
Weighted production quantities or pollination benefits
0
1
2
3
4
5
6
1995
2000
2005

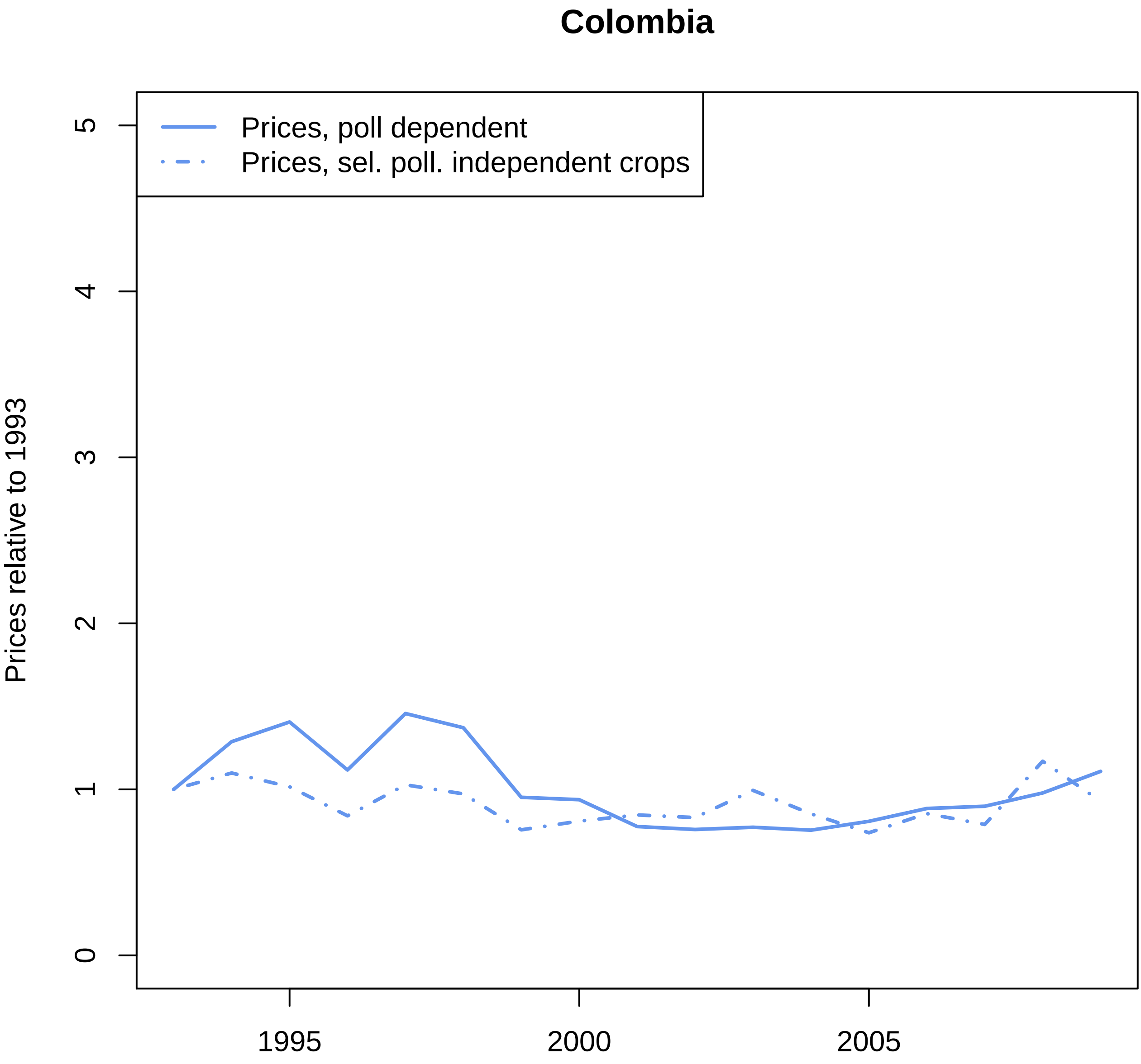
Colombia
Prices, poll dependent
Prices, sel. poll. independent crops
Prices relative to 1993
0
1
2
3
4
5
1995
2000
2005

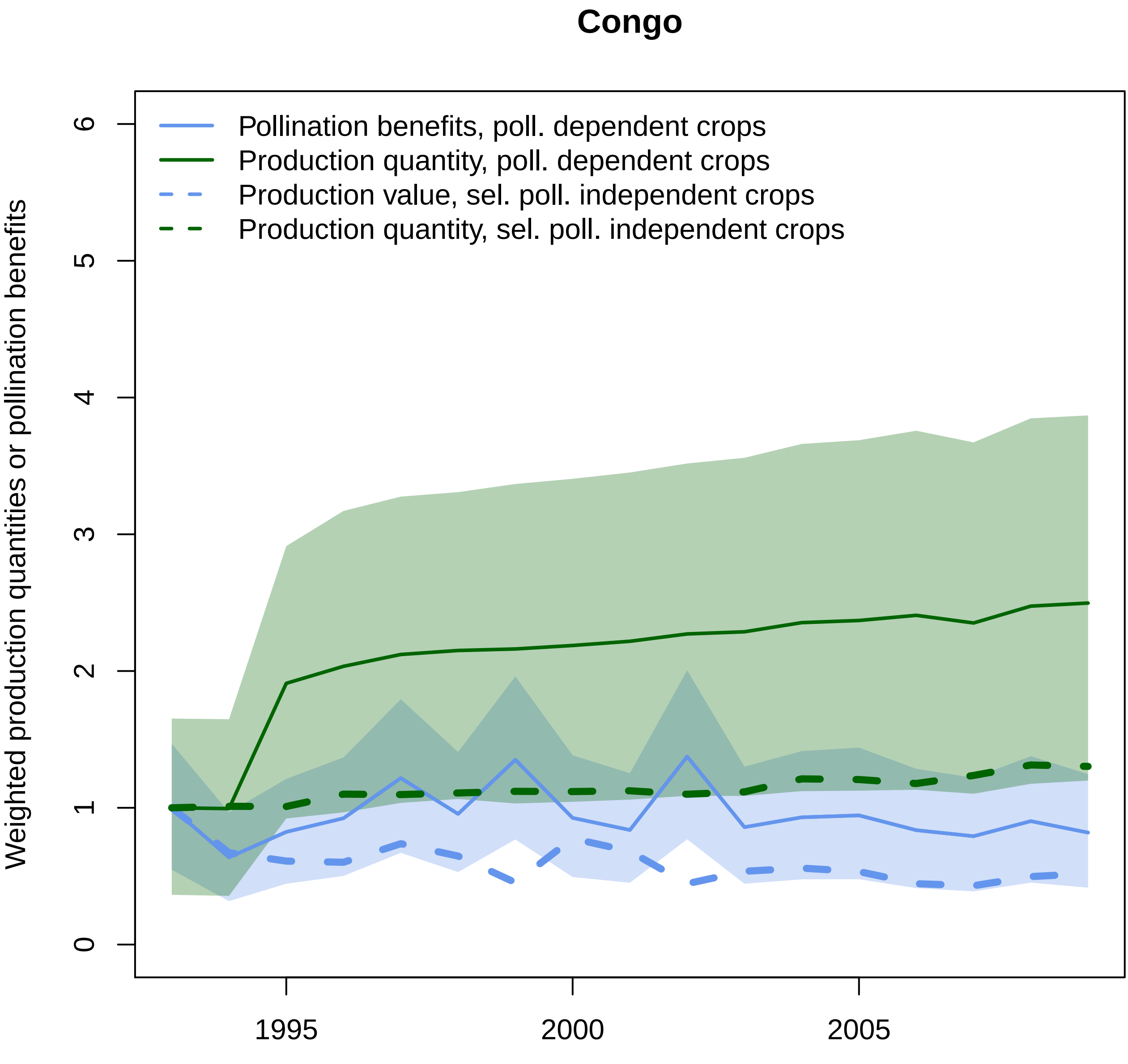
Congo
Pollination benefits, poll. dependent crops
Production quantity, poll. dependent crops
Production value, sel. poll. independent crops
Production quantity, sel. poll. independent crops
Weighted production quantities or pollination benefits
0
1
2
3
4
5
6
1995
2000
2005

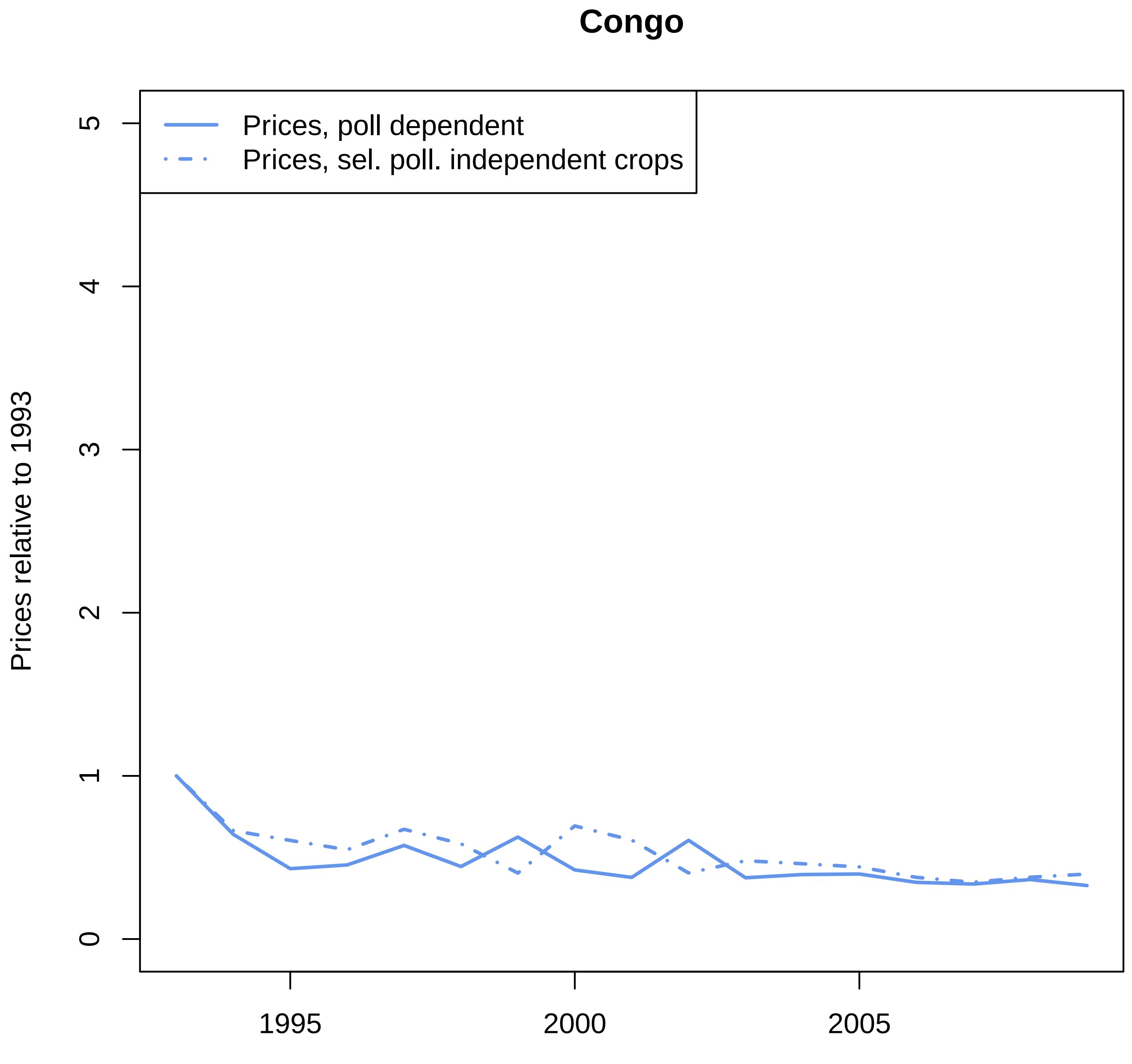
Congo
Prices, poll dependent
Prices, sel. poll. independent crops
Prices relative to 1993
0
1
2
3
4
5
1995
2000
2005

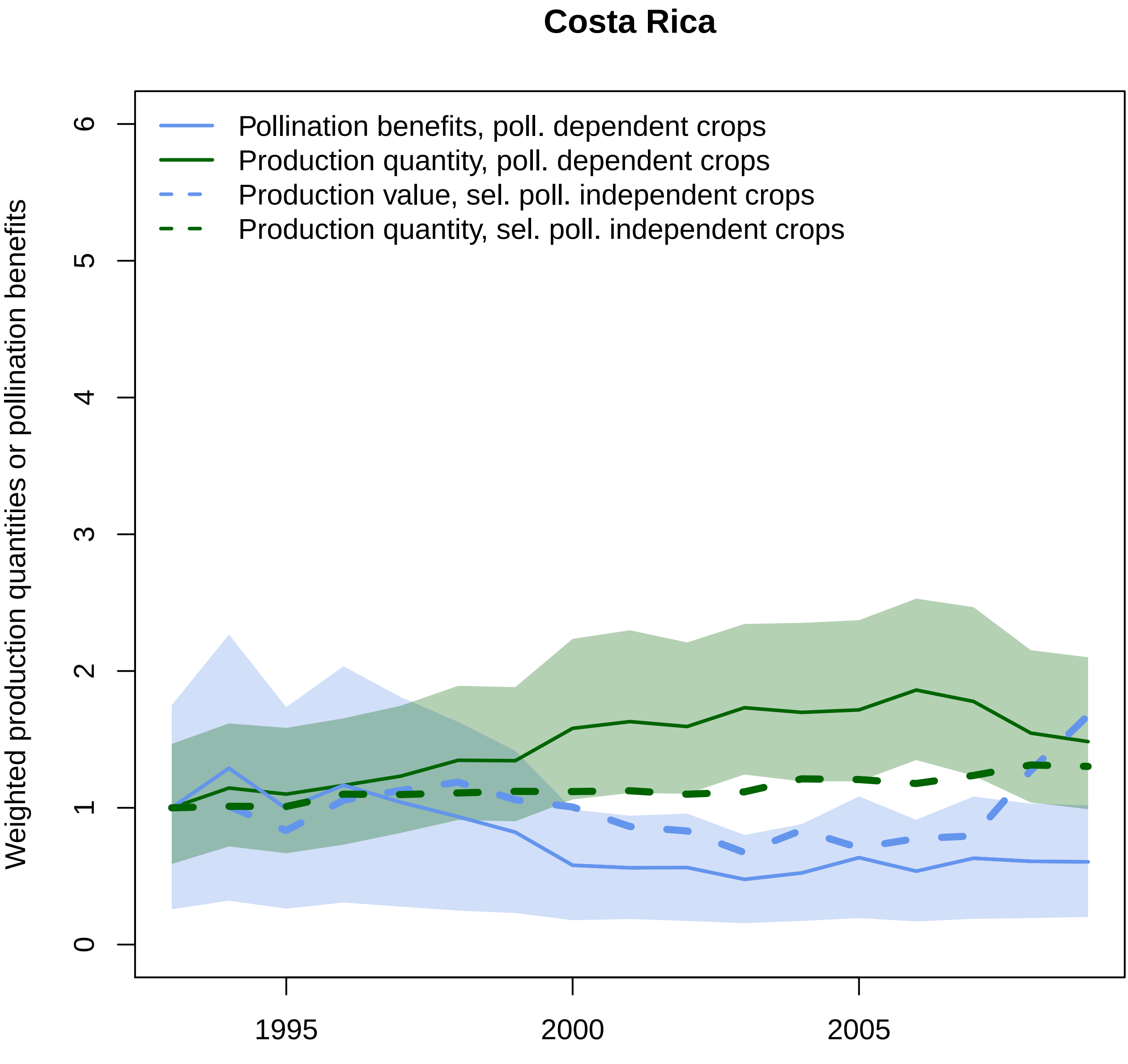

Costa Rica
Pollination benefits, poll. dependent crops
Production quantity, poll. dependent crops
Production value, sel. poll. independent crops
Production quantity, sel. poll. independent crops
Weighted production quantities or pollination benefits
0
1
2
3
4
5
6
1995
2000
2005

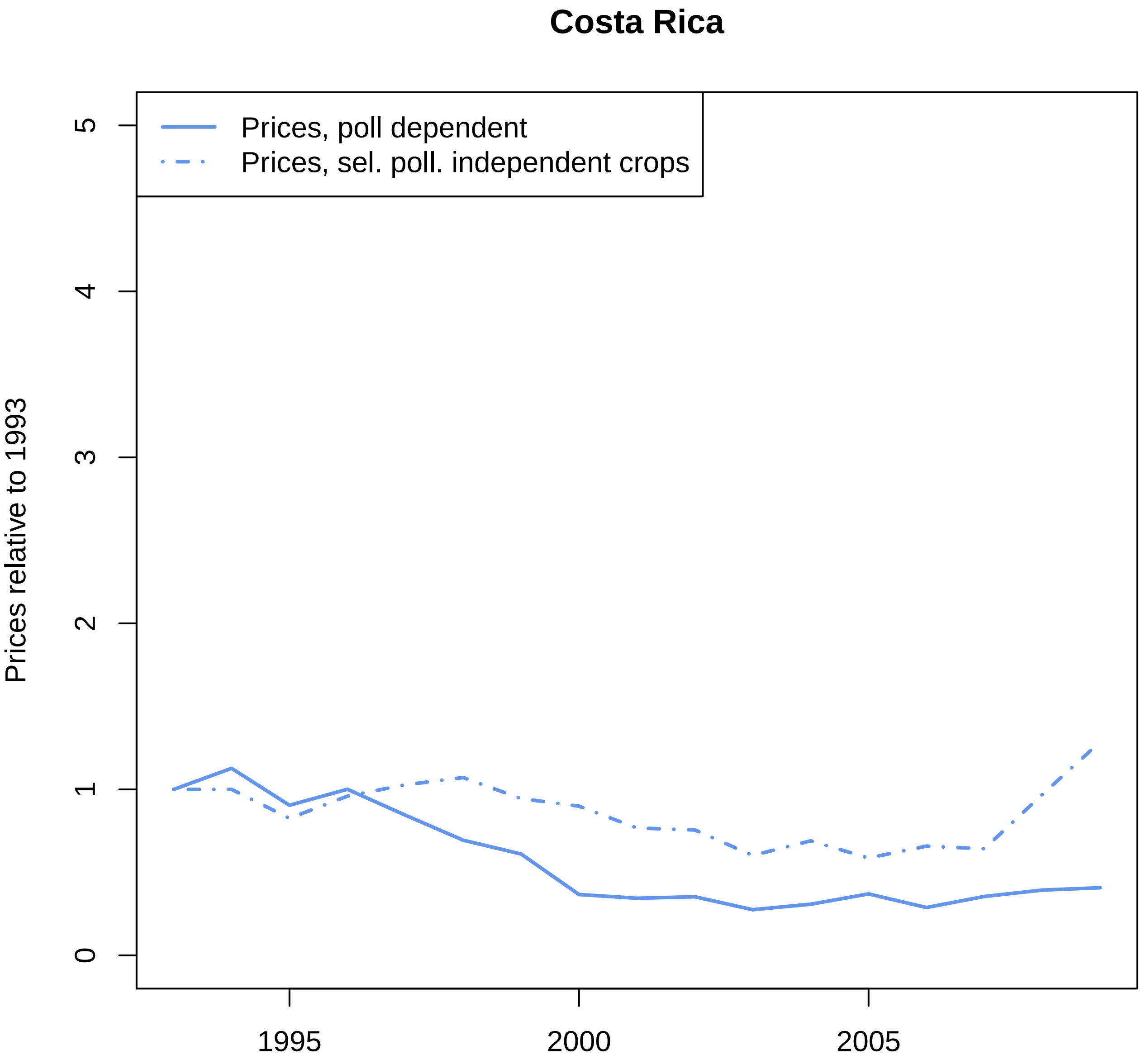

Costa Rica
Prices, poll dependent
Prices, sel. poll. independent crops
Prices relative to 1993
0
1
2
3
4
5
1995
2000
2005

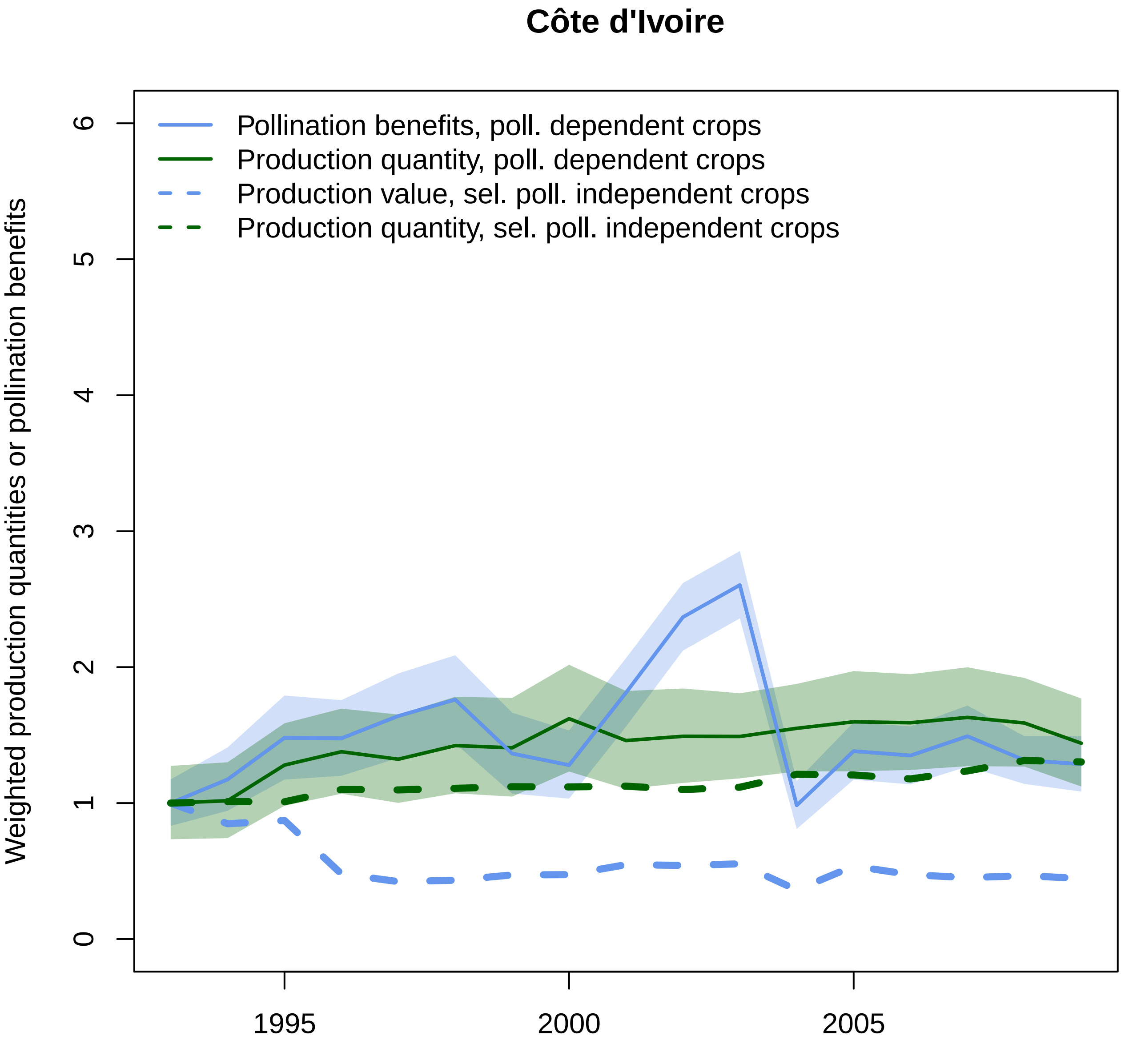

Côte d'Ivoire
Pollination benefits, poll. dependent crops
Production quantity, poll. dependent crops
Production value, sel. poll. independent crops
Production quantity, sel. poll. independent crops
Weighted production quantities or pollination benefits
0
1
2
3
4
5
6
1995
2000
2005

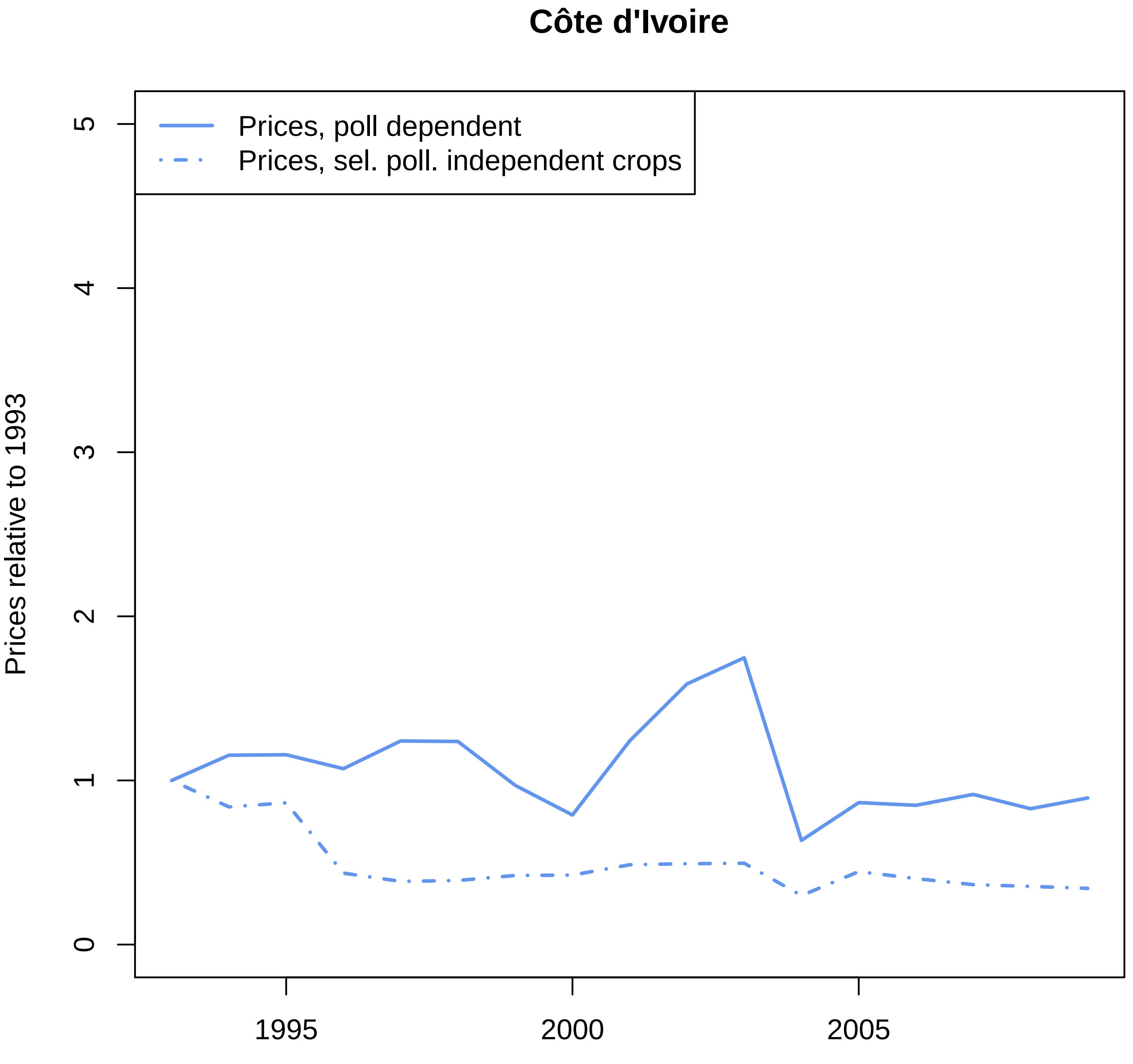
Côte d'Ivoire
Prices, poll dependent
Prices, sel. poll. independent crops
Prices relative to 1993
0
1
2
3
4
5
1995
2000
2005

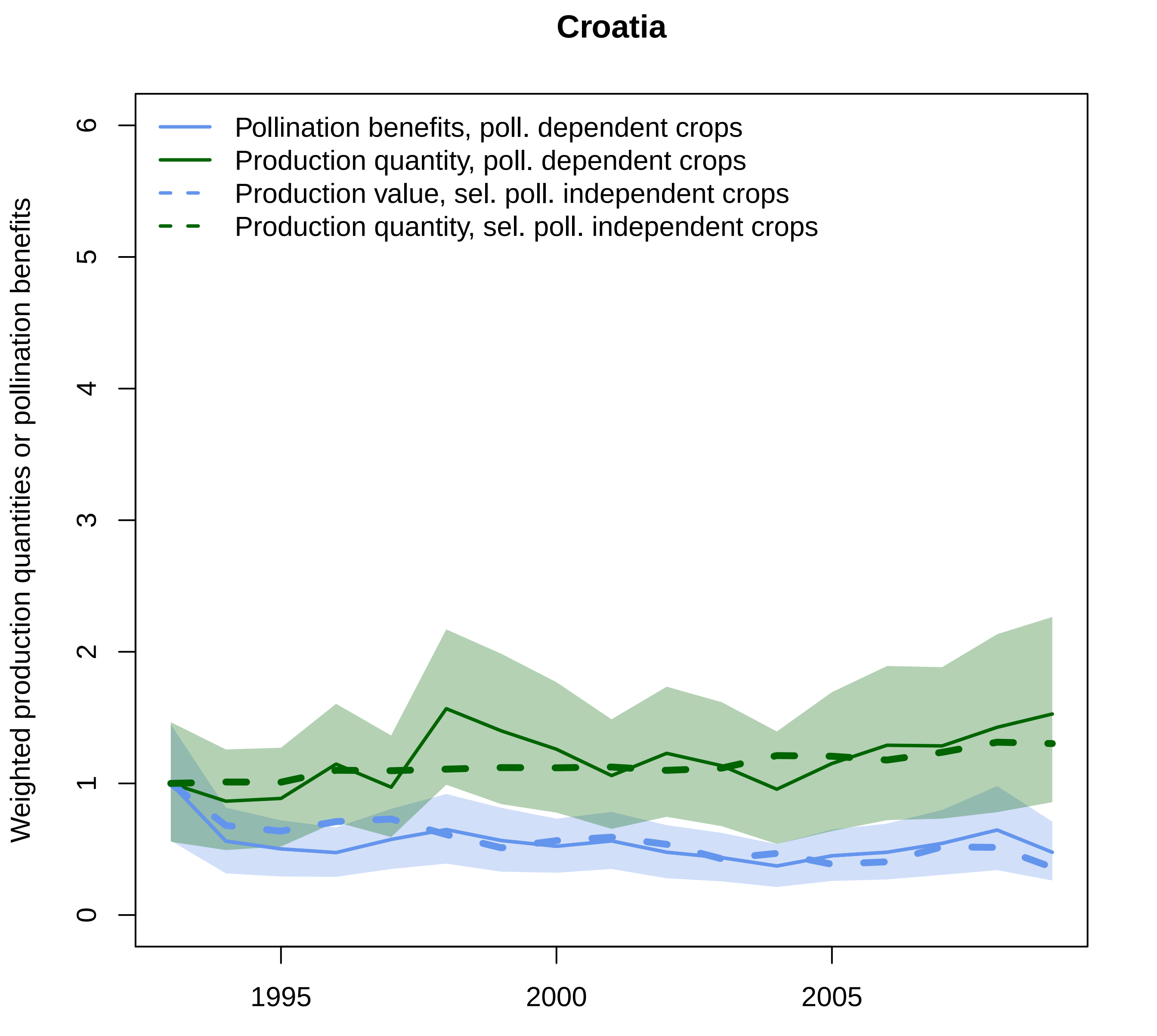

Croatia
Pollination benefits, poll. dependent crops
Production quantity, poll. dependent crops
Production value, sel. poll. independent crops
Production quantity, sel. poll. independent crops
Weighted production quantities or pollination benefits
0
1
2
3
4
5
6
1995
2000
2005

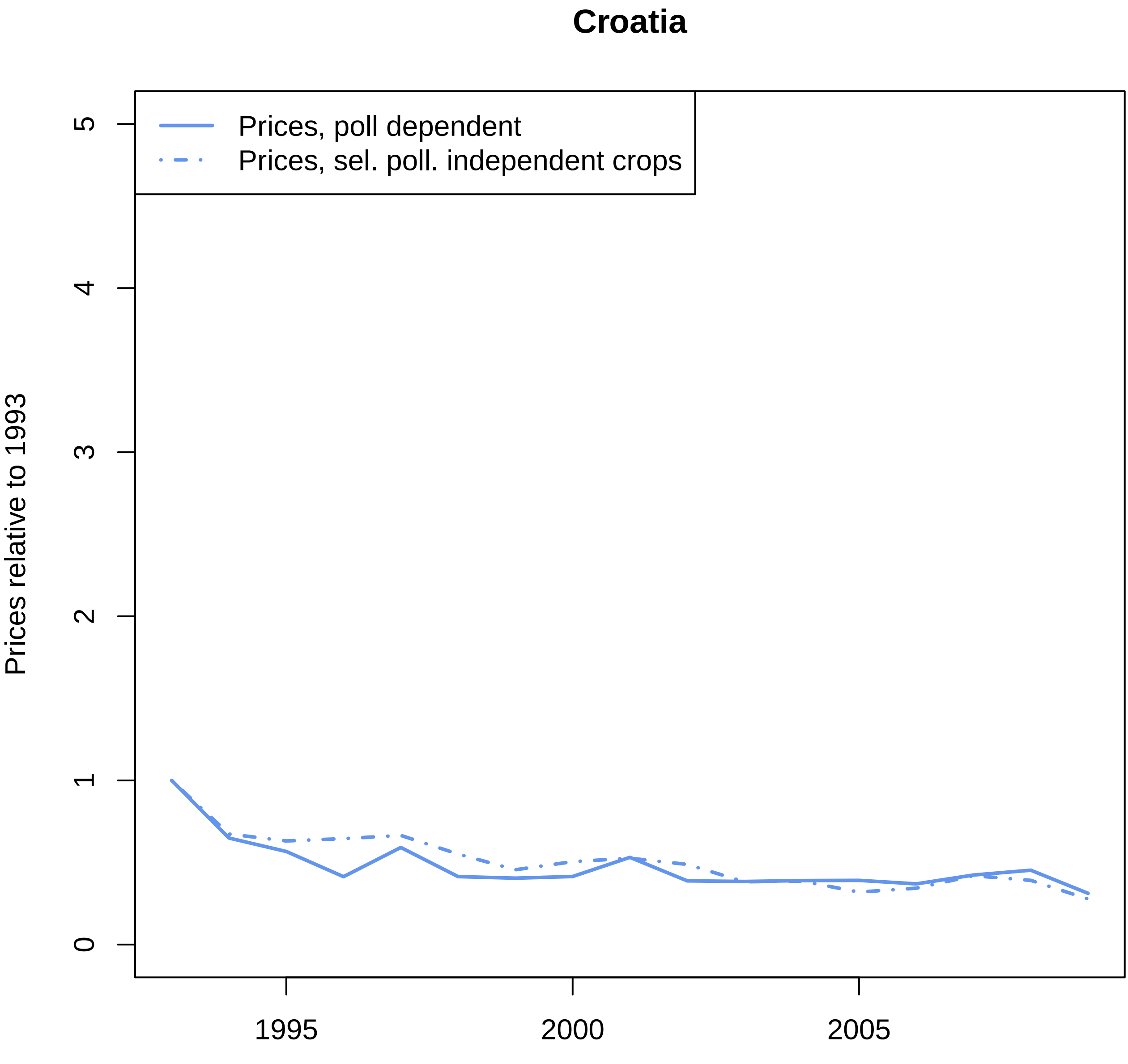
Croatia
Prices, poll dependent
Prices, sel. poll. independent crops
Prices relative to 1993
0
1
2
3
4
5
1995
2000
2005

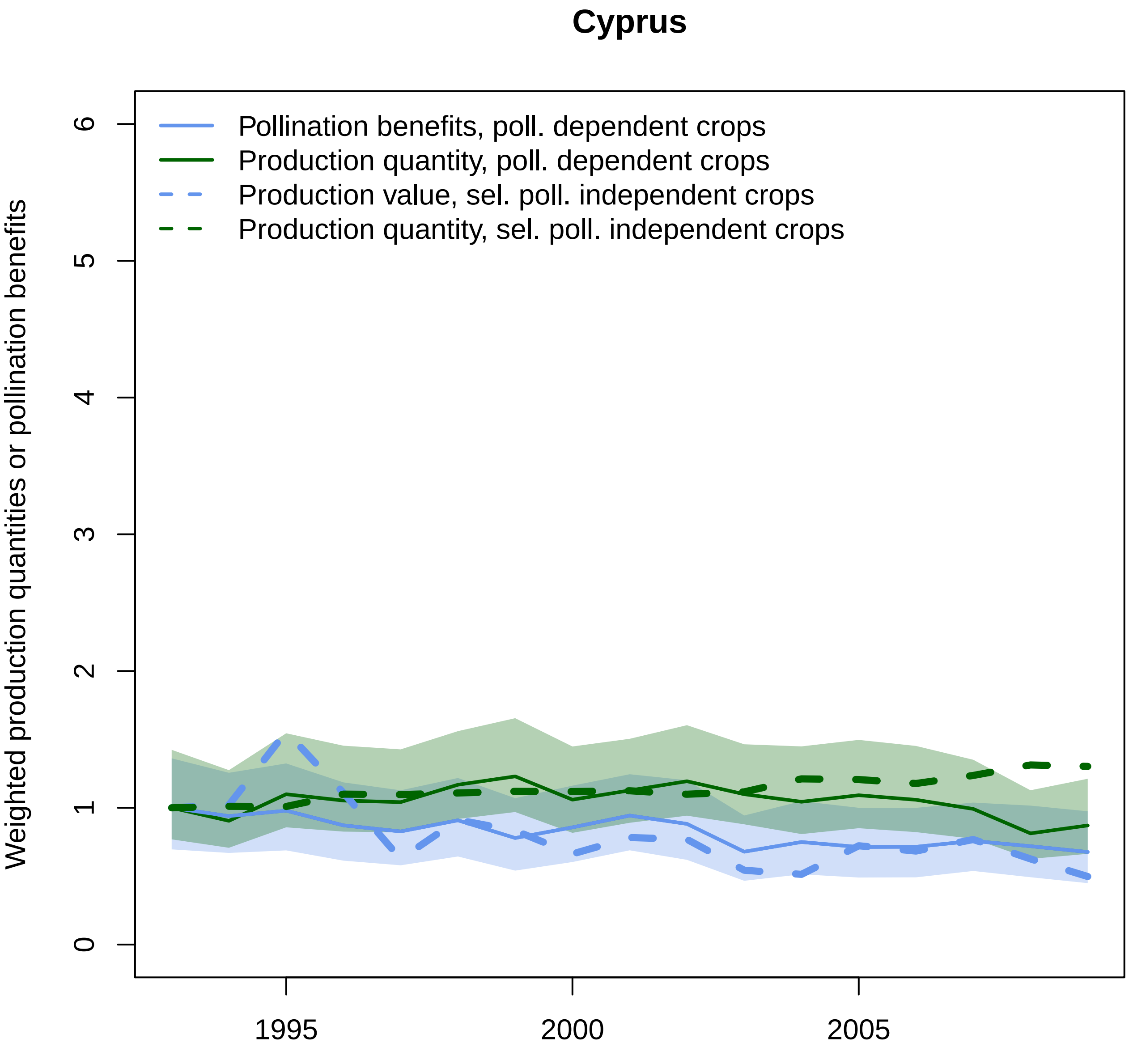

Cyprus
Pollination benefits, poll. dependent crops
Production quantity, poll. dependent crops
Production value, sel. poll. independent crops
Production quantity, sel. poll. independent crops
Weighted production quantities or pollination benefits
0
1
2
3
4
5
6
1995
2000
2005

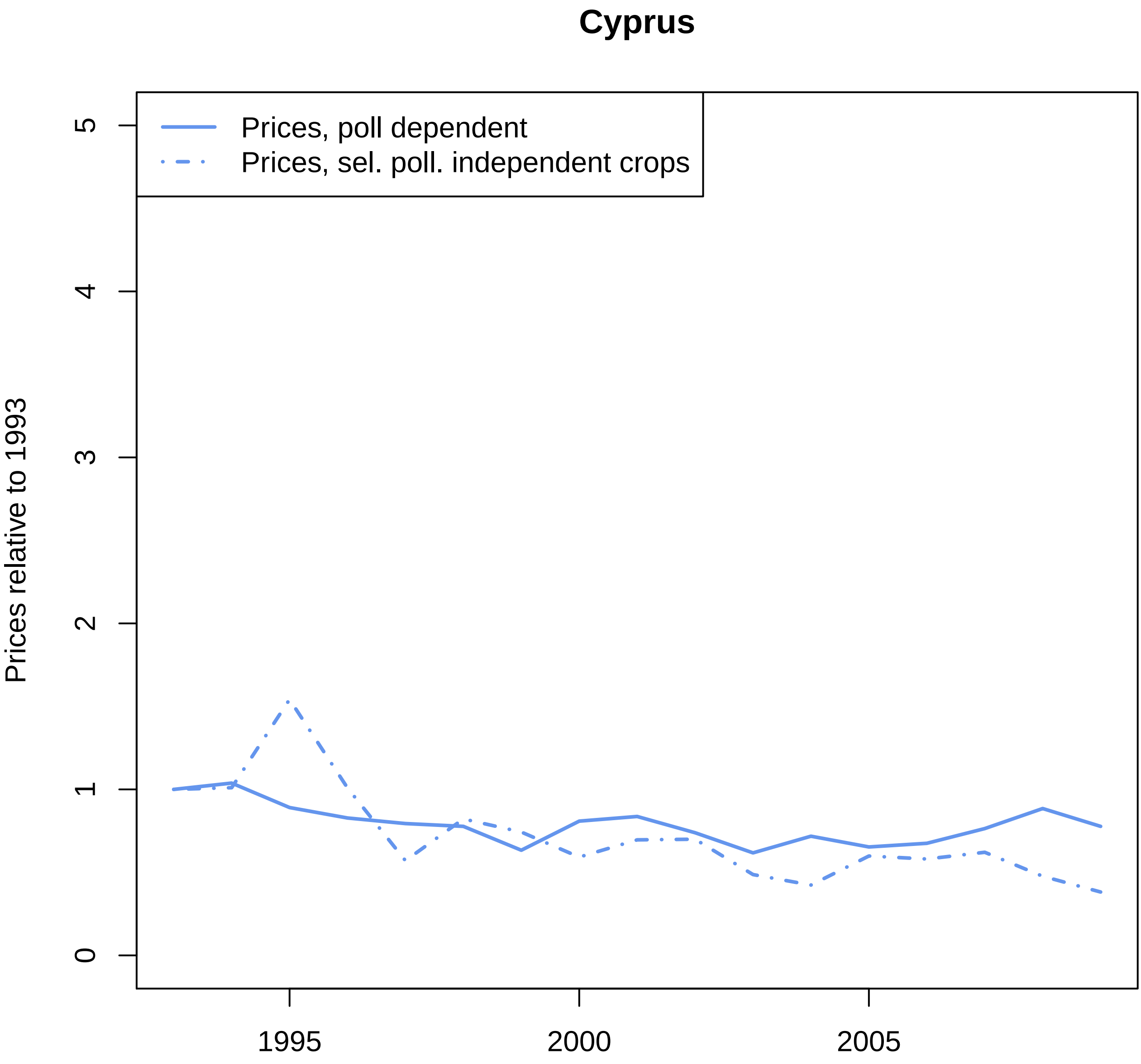

Cyprus
Prices, poll dependent
Prices, sel. poll. independent crops
Prices relative to 1993
0
1
2
3
4
5
1995
2000
2005

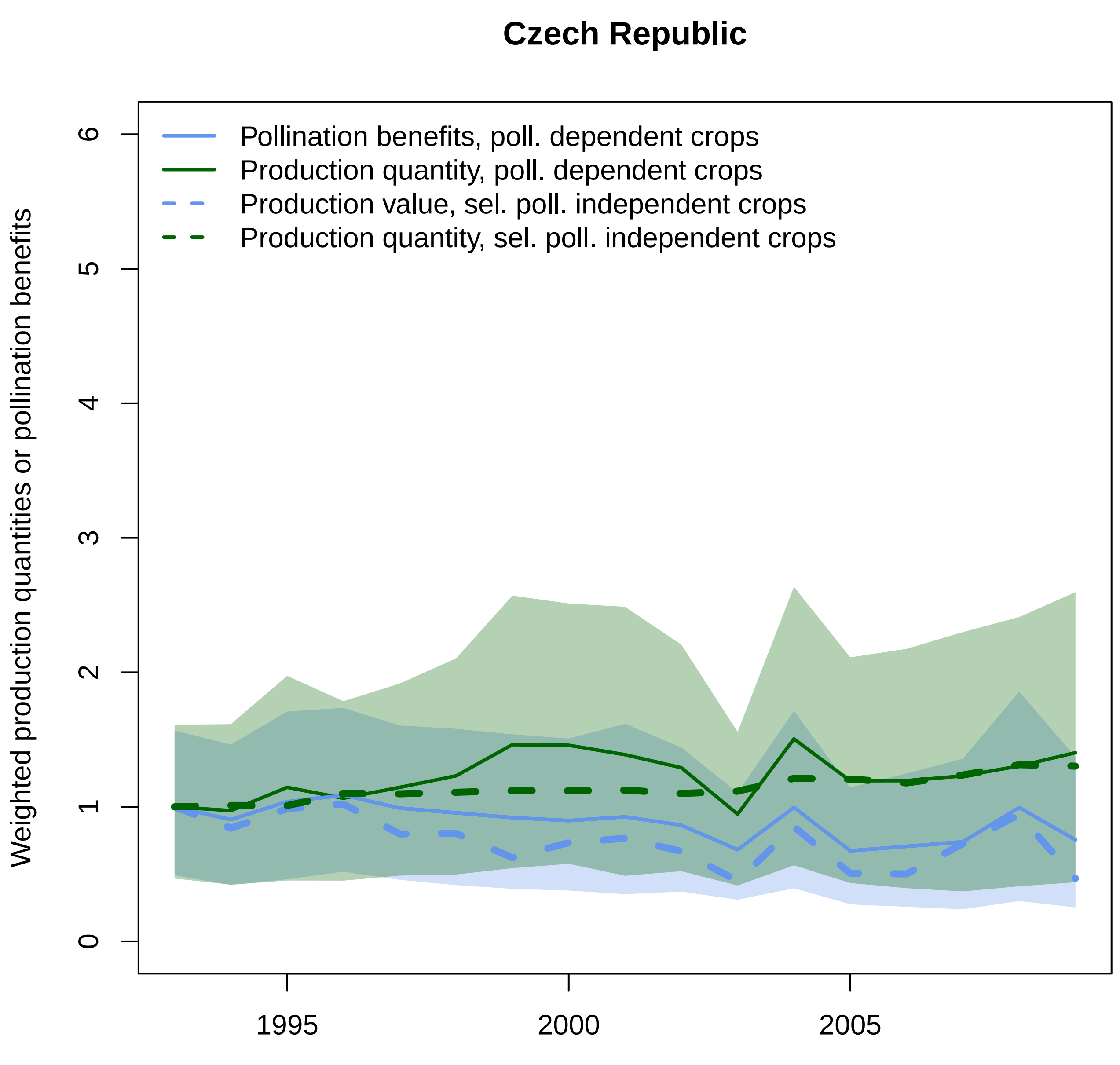

Czech Republic
Pollination benefits, poll. dependent crops
Production quantity, poll. dependent crops
Production value, sel. poll. independent crops
Production quantity, sel. poll. independent crops
Weighted production quantities or pollination benefits
0
1
2
3
4
5
6
1995
2000
2005

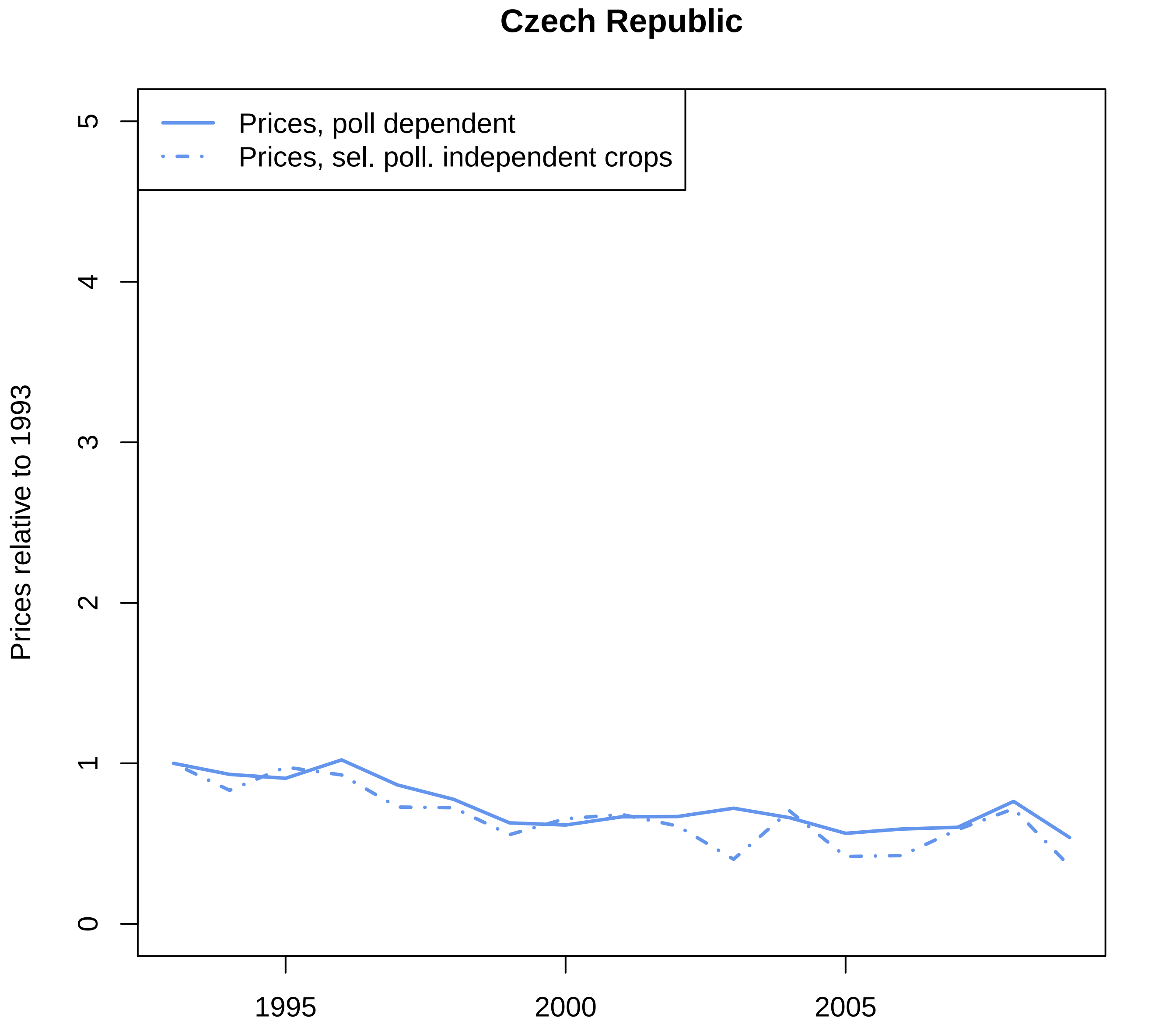
Czech Republic
Prices, poll dependent
Prices, sel. poll. independent crops
Prices relative to 1993
0
1
2
3
4
5
1995
2000
2005

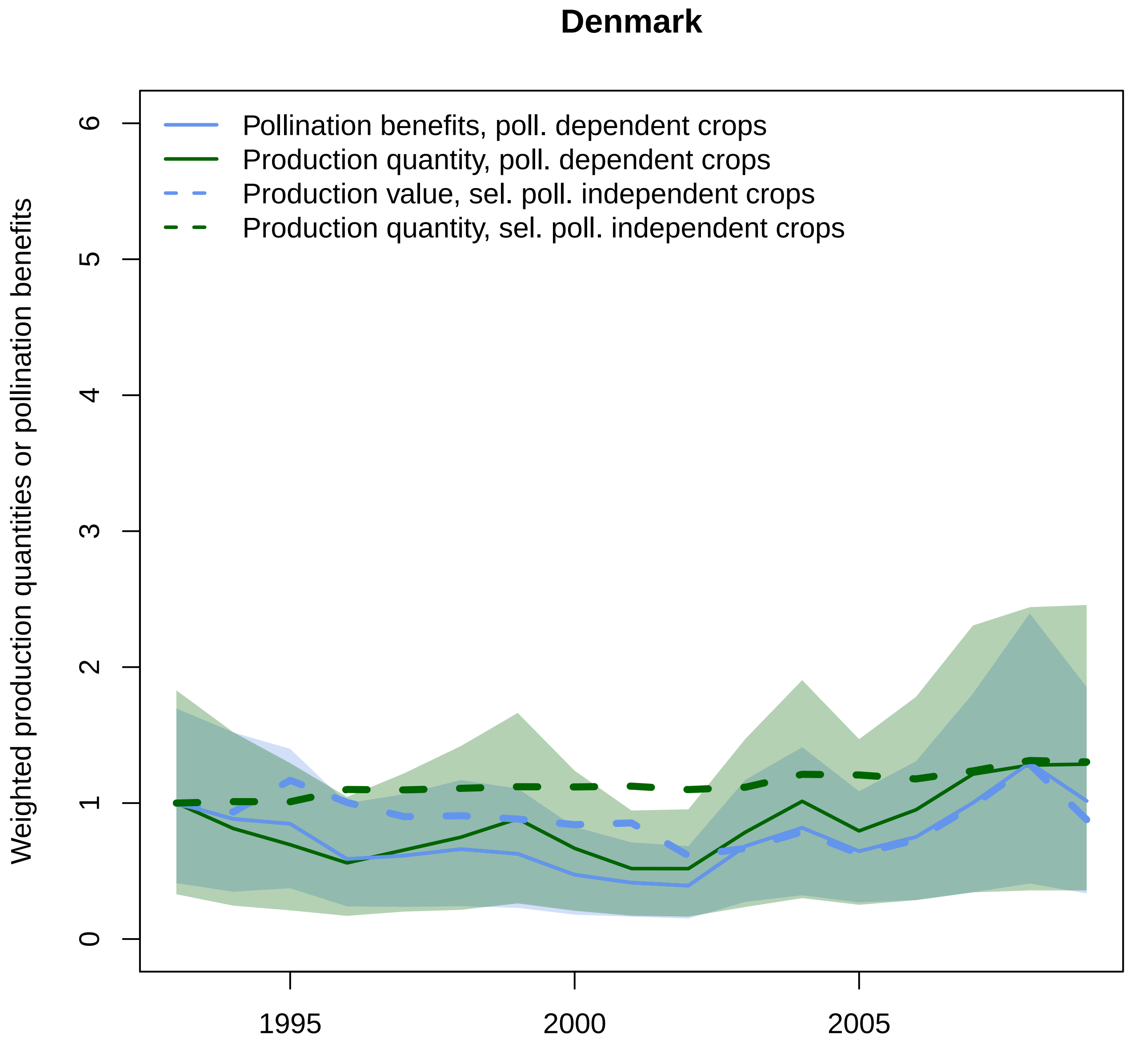

Denmark
Pollination benefits, poll. dependent crops
Production quantity, poll. dependent crops
Production value, sel. poll. independent crops
Production quantity, sel. poll. independent crops
Weighted production quantities or pollination benefits
0
1
2
3
4
5
6
1995
2000
2005

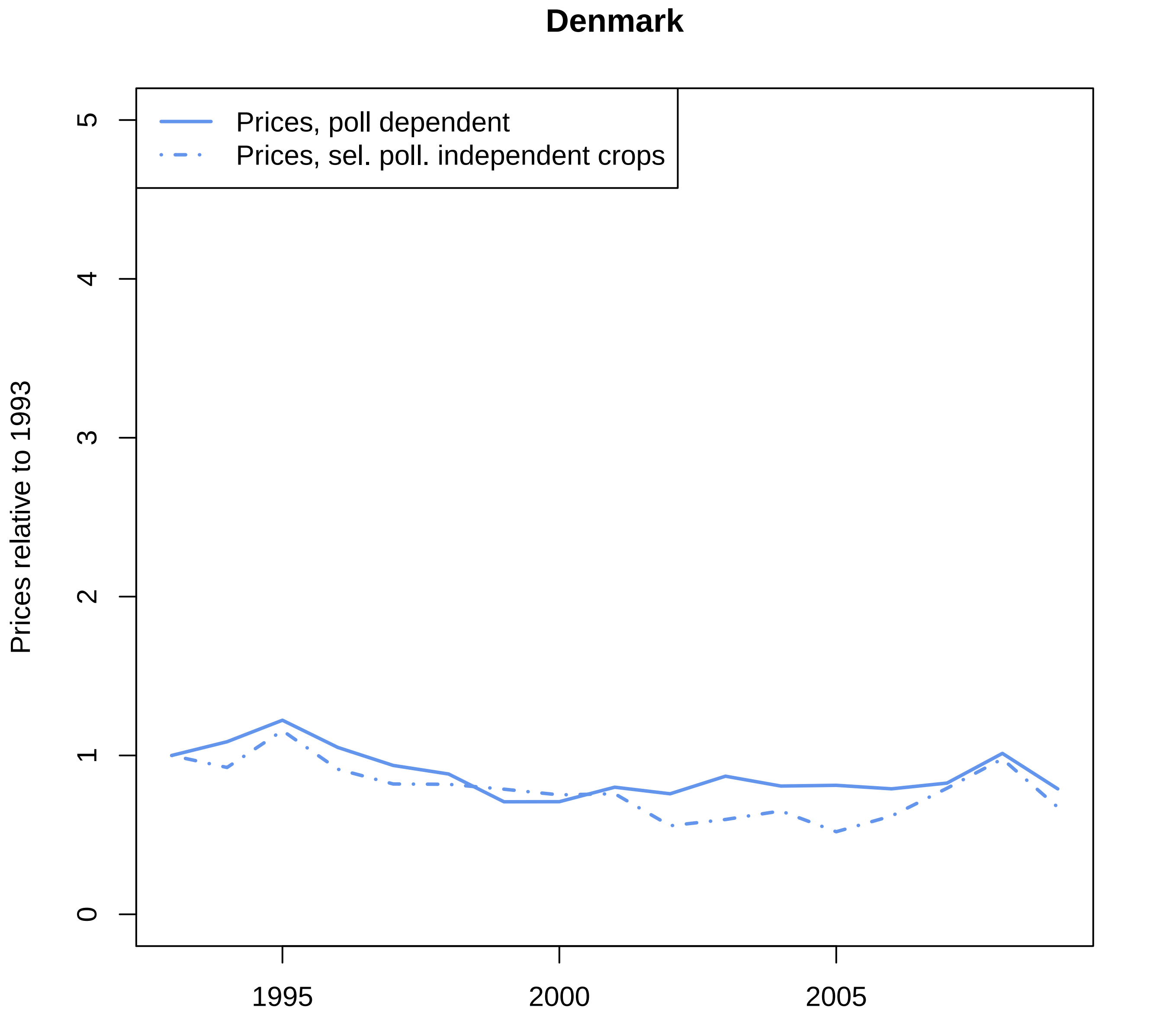

Denmark
Prices, poll dependent
Prices, sel. poll. independent crops
Prices relative to 1993
0
1
2
3
4
5
1995
2000
2005

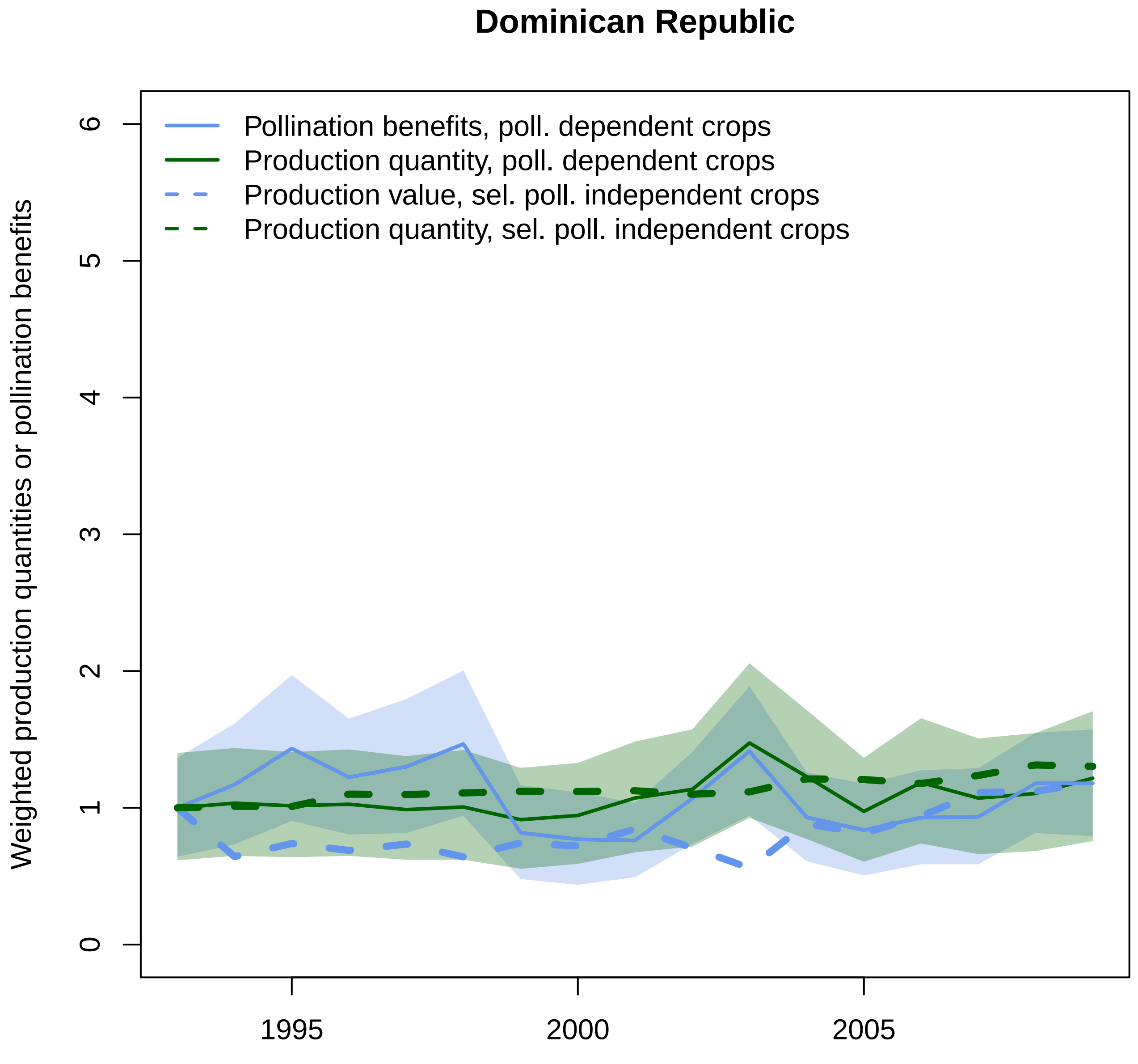
Dominican Republic
Pollination benefits, poll. dependent crops
Production quantity, poll. dependent crops
Production value, sel. poll. independent crops
Production quantity, sel. poll. independent crops
Weighted production quantities or pollination benefits
0
1
2
3
4
5
6
1995
2000
2005

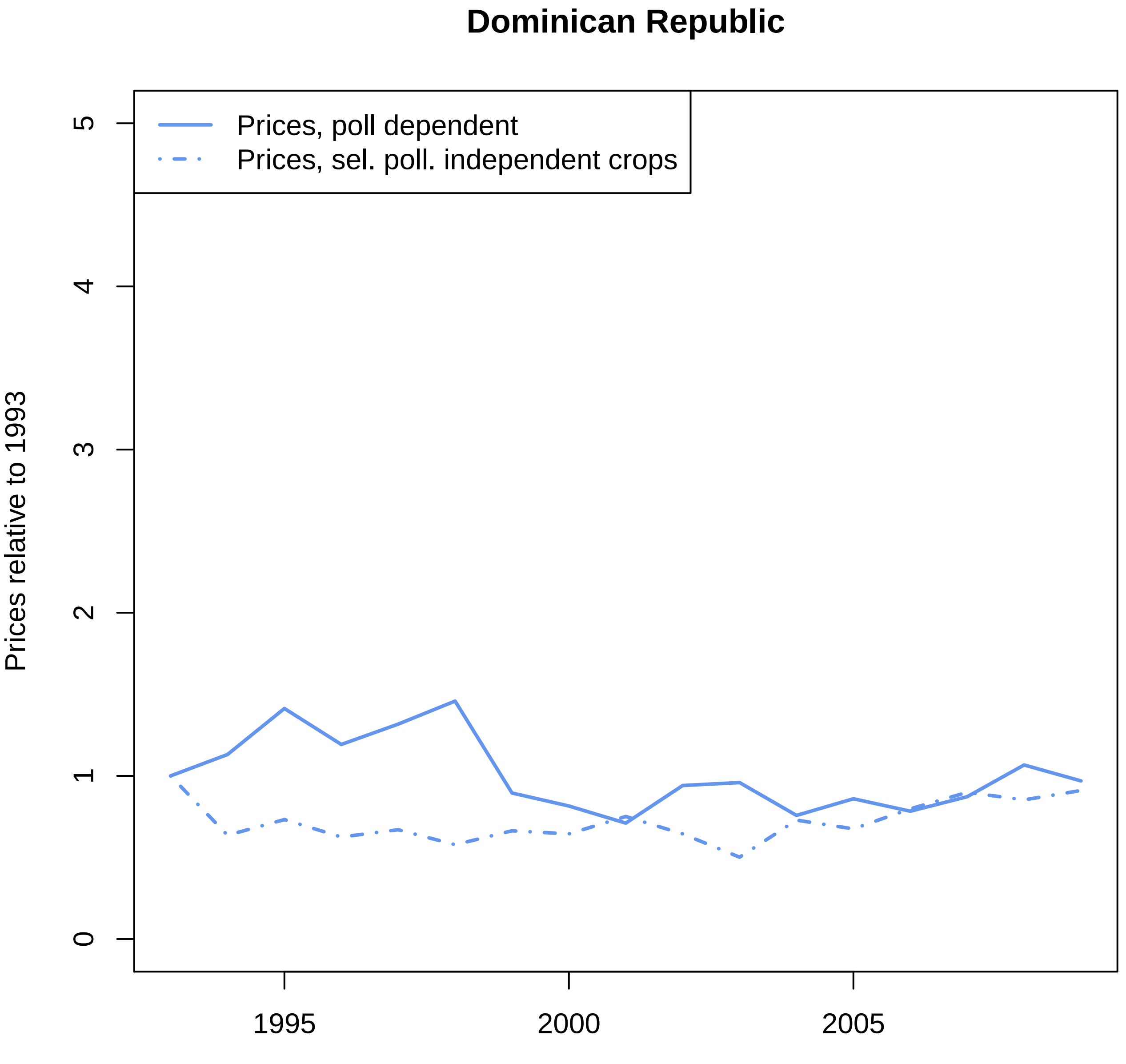

Dominican Republic
Prices, poll dependent
Prices, sel. poll. independent crops
Prices relative to 1993
0
1
2
3
4
5
1995
2000
2005

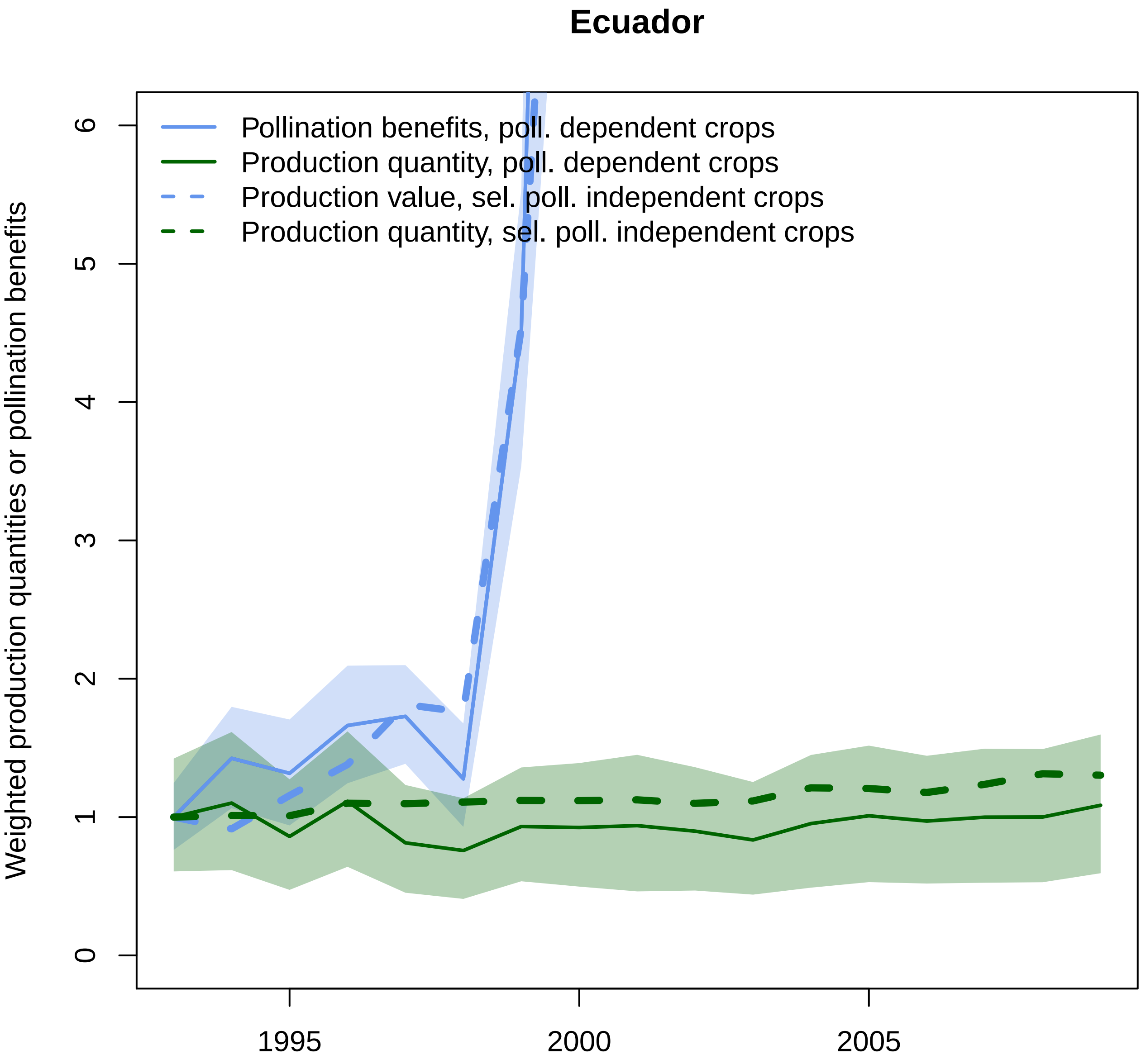

Ecuador
Pollination benefits, poll. dependent crops
Production quantity, poll. dependent crops
Production value, sel. poll. independent crops
Production quantity, sel. poll. independent crops
Weighted production quantities or pollination benefits
0
1
2
3
4
5
6
1995
2000
2005

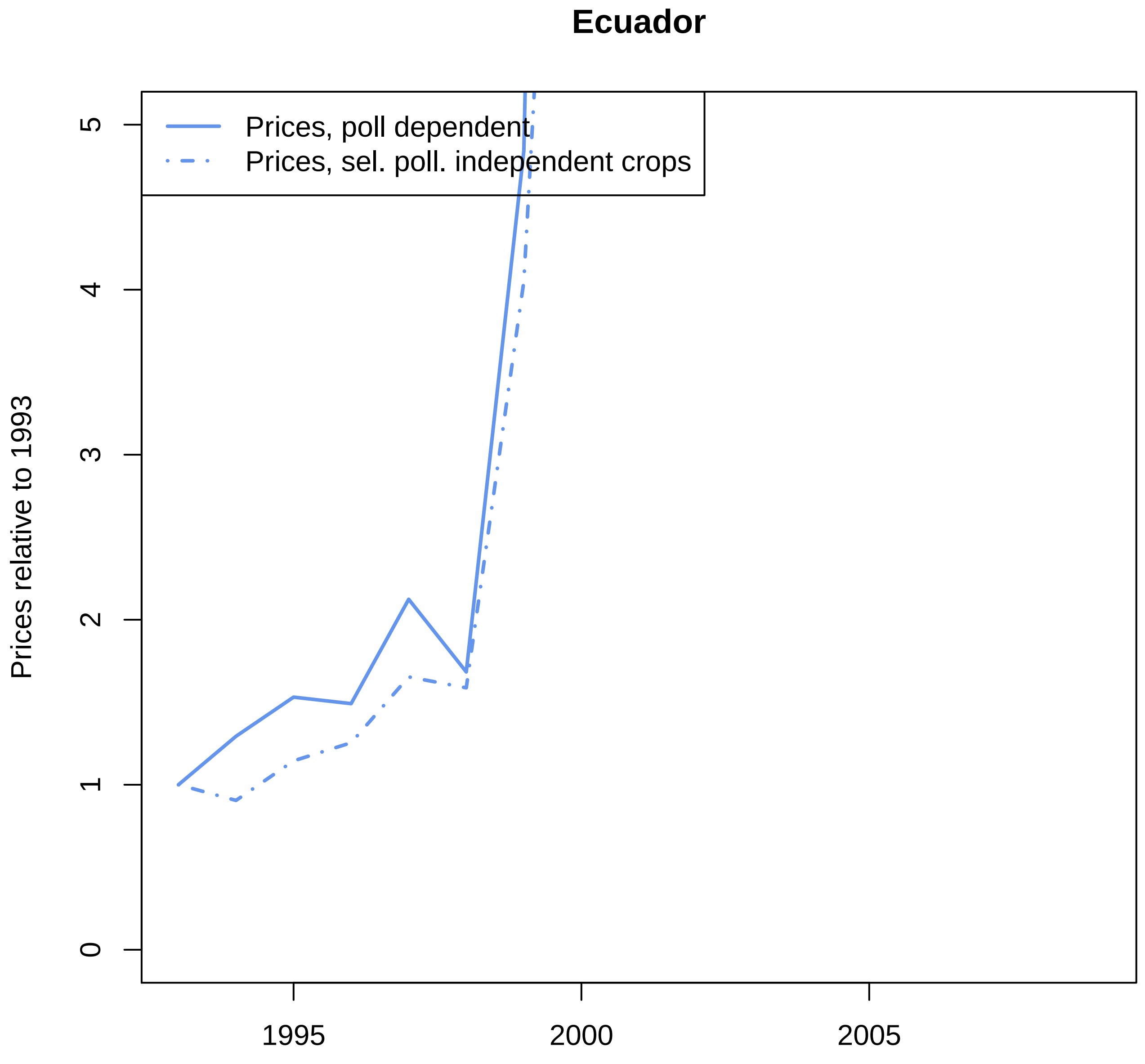
Ecuador
Prices, poll dependent
Prices, sel. poll. independent crops
Prices relative to 1993
0
1
2
3
4
5
1995
2000
2005

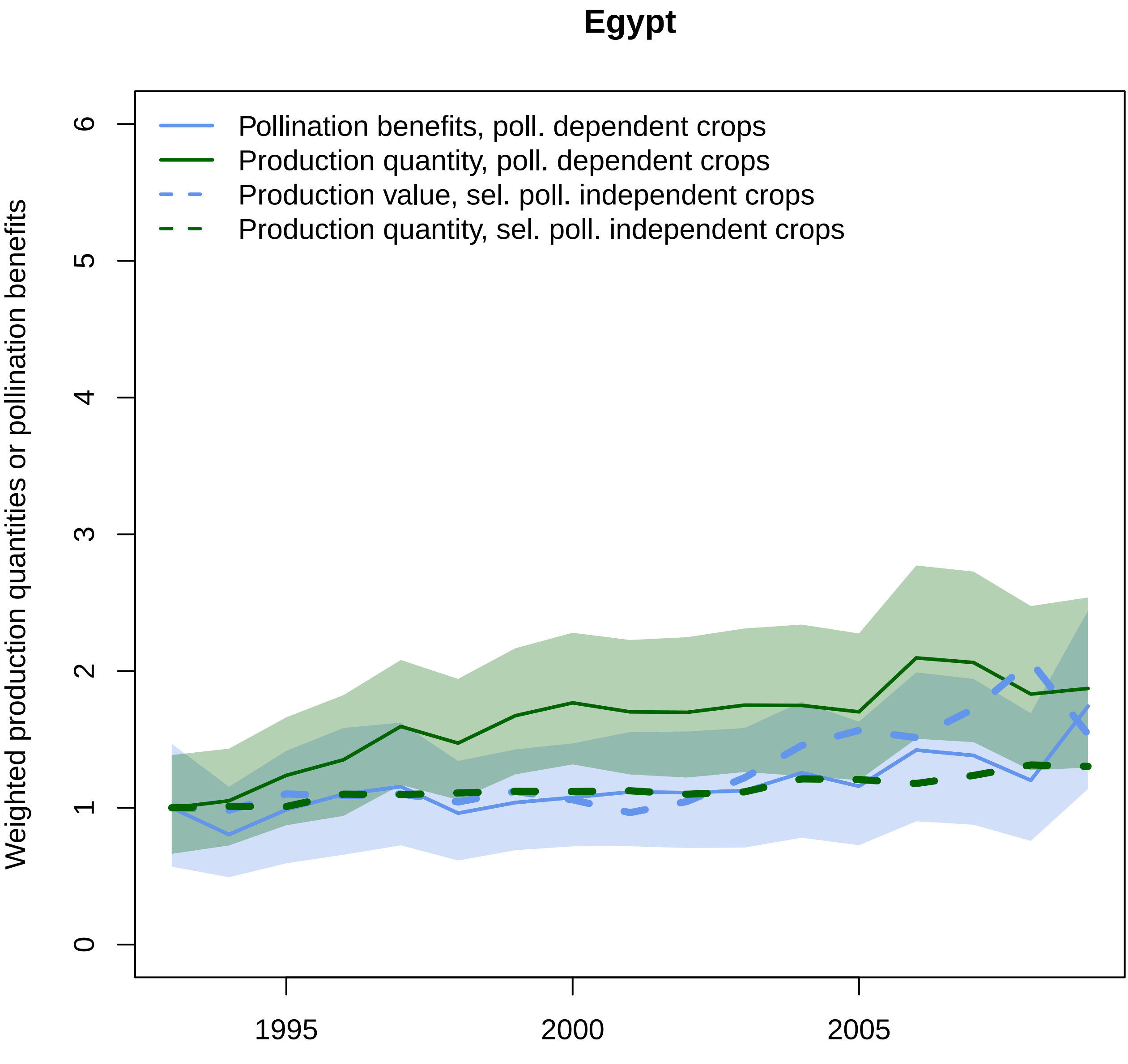
Egypt
Pollination benefits, poll. dependent crops
Production quantity, poll. dependent crops
Production value, sel. poll. independent crops
Production quantity, sel. poll. independent crops
Weighted production quantities or pollination benefits
0
1
2
3
4
5
6
1995
2000
2005

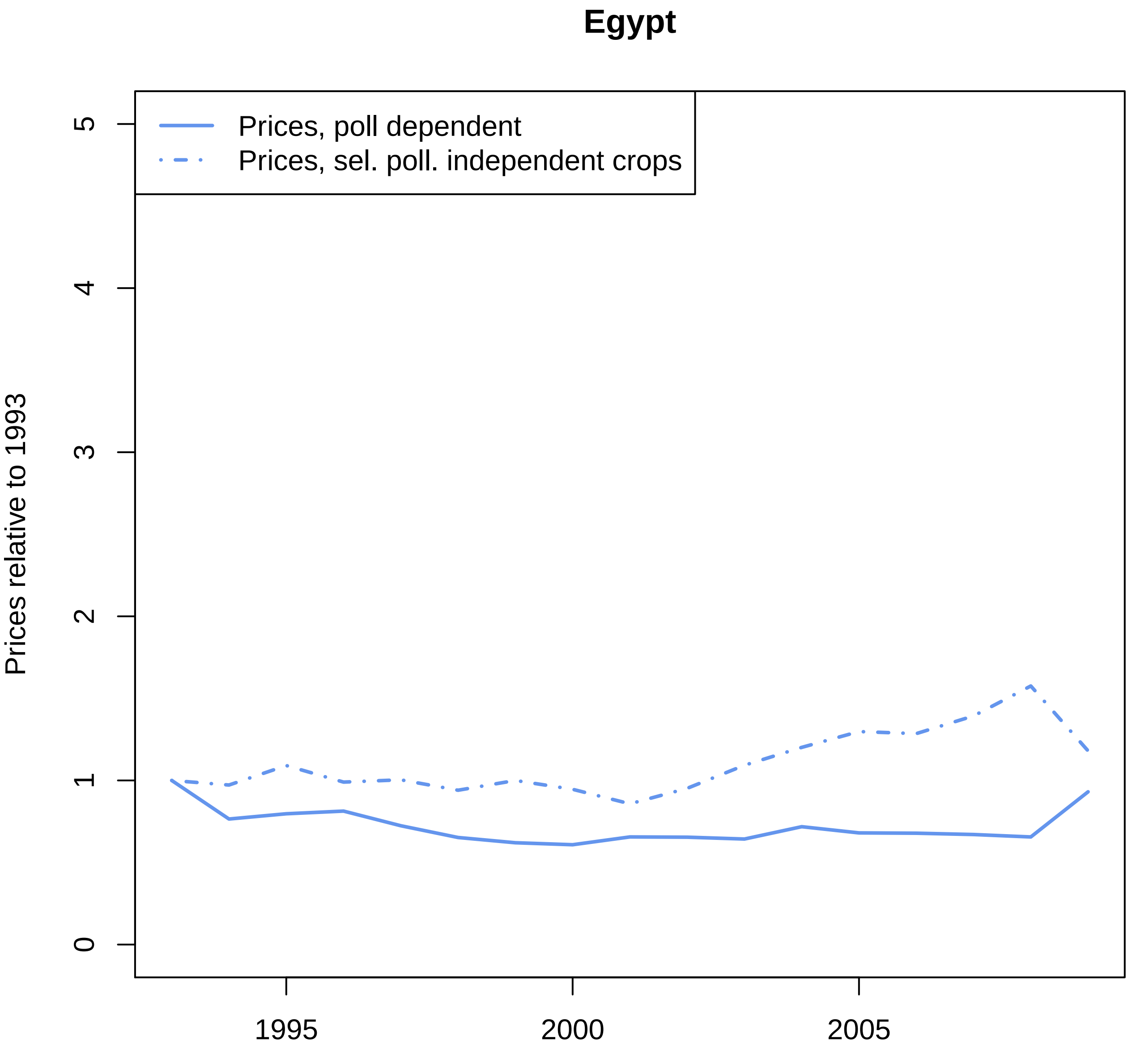
Egypt
Prices, poll dependent
Prices, sel. poll. independent crops
Prices relative to 1993
0
1
2
3
4
5
1995
2000
2005

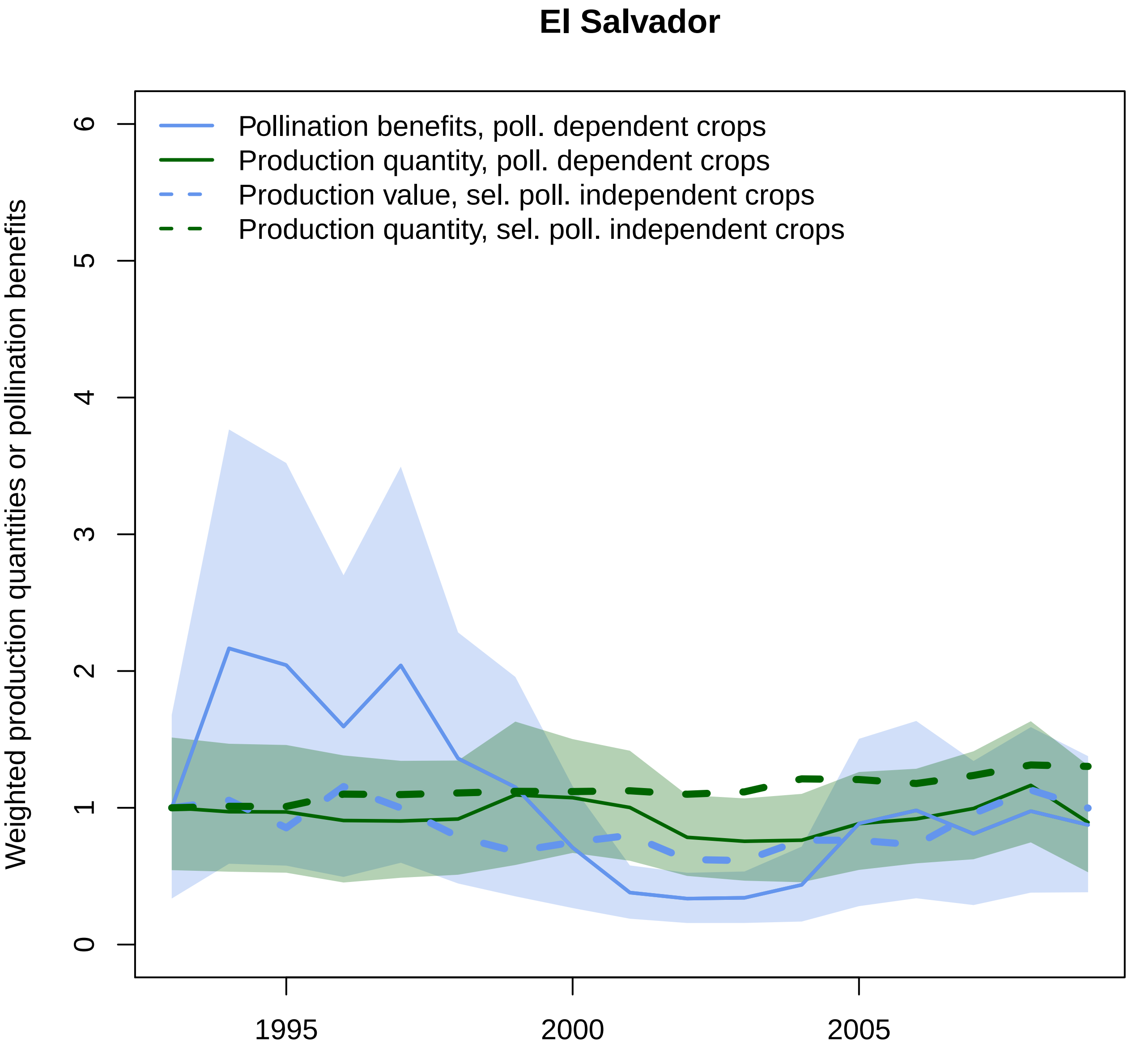

El Salvador
Pollination benefits, poll. dependent crops
Production quantity, poll. dependent crops
Production value, sel. poll. independent crops
Production quantity, sel. poll. independent crops
Weighted production quantities or pollination benefits
0
1
2
3
4
5
6
1995
2000
2005

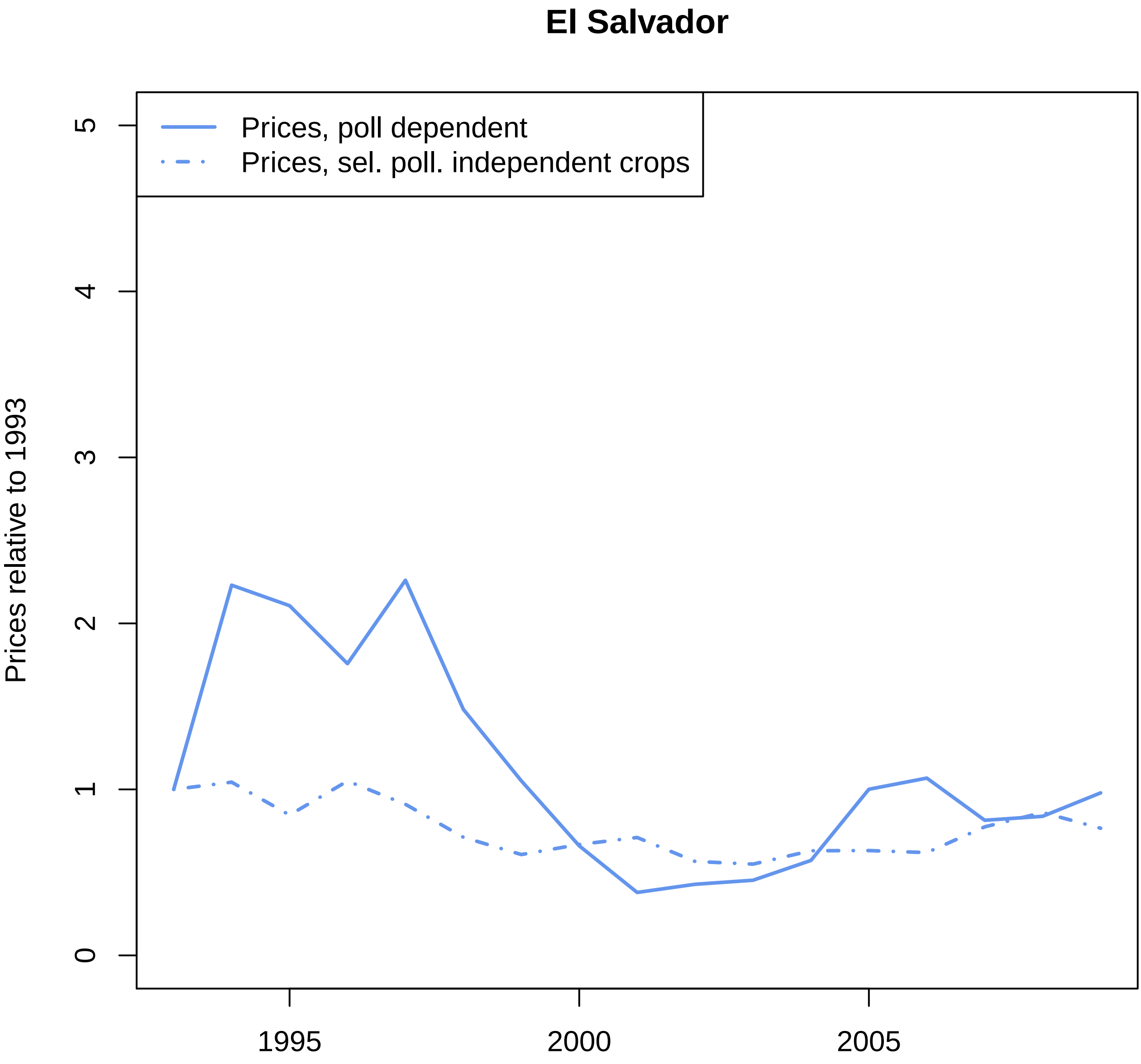

El Salvador
Prices, poll dependent
Prices, sel. poll. independent crops
Prices relative to 1993
0
1
2
3
4
5
1995
2000
2005

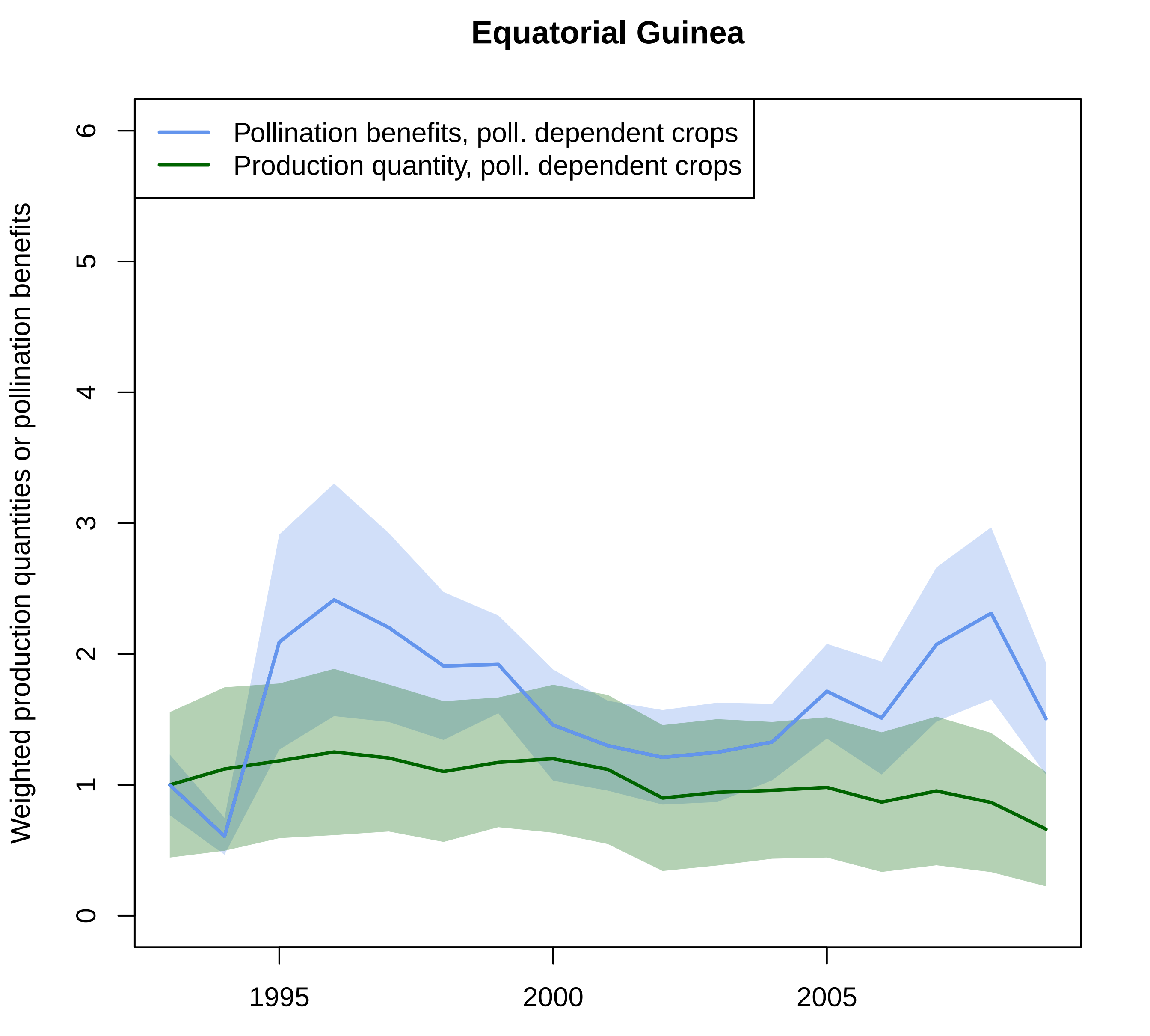

Equatorial Guinea
Pollination benefits, poll. dependent crops
Production quantity, poll. dependent crops
Weighted production quantities or pollination benefits
0
1
2
3
4
5
6
1995
2000
2005

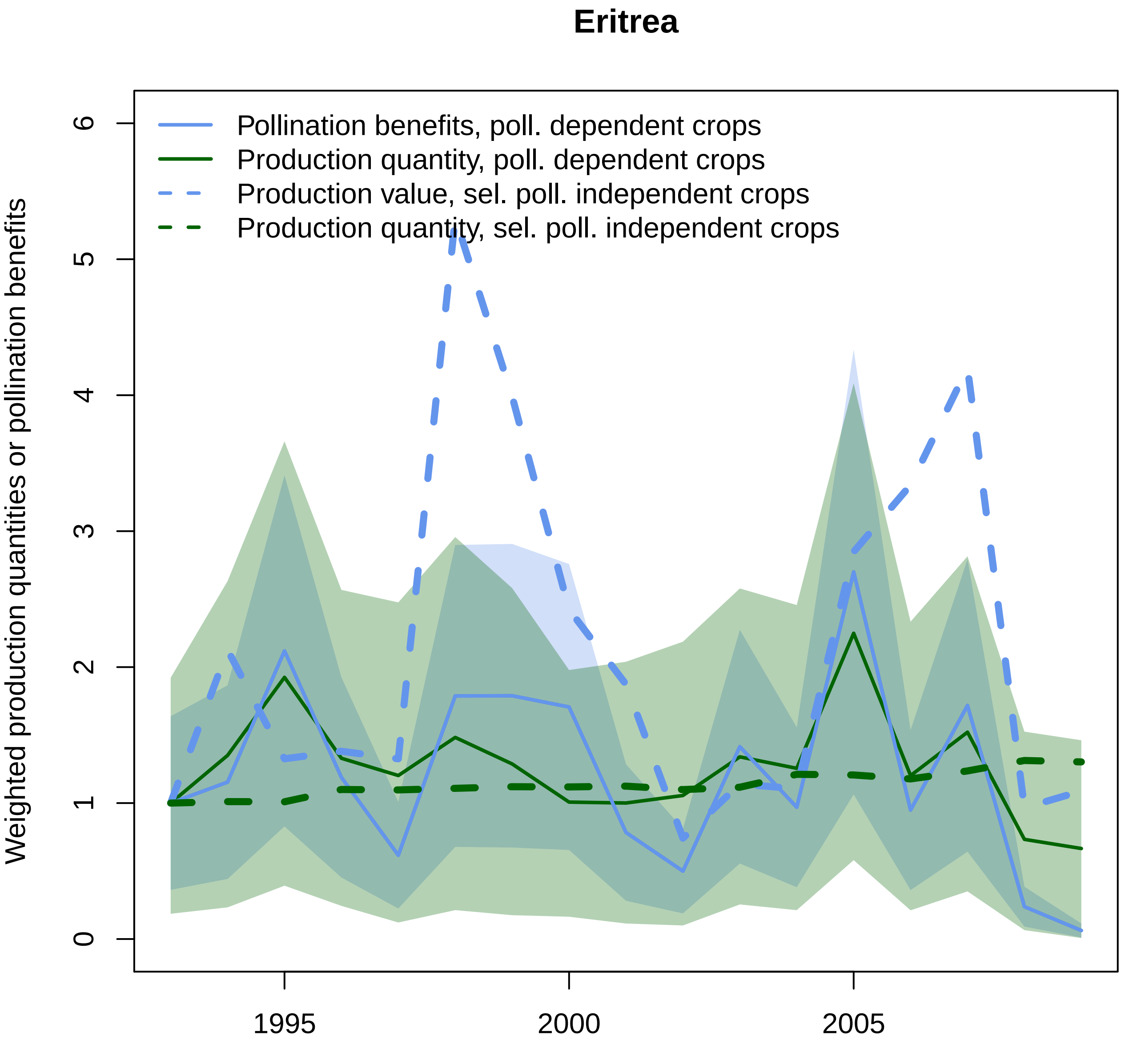
Eritrea
Pollination benefits, poll. dependent crops
Production quantity, poll. dependent crops
Production value, sel. poll. independent crops
Production quantity, sel. poll. independent crops
Weighted production quantities or pollination benefits
0
1
2
3
4
5
6
1995
2000
2005

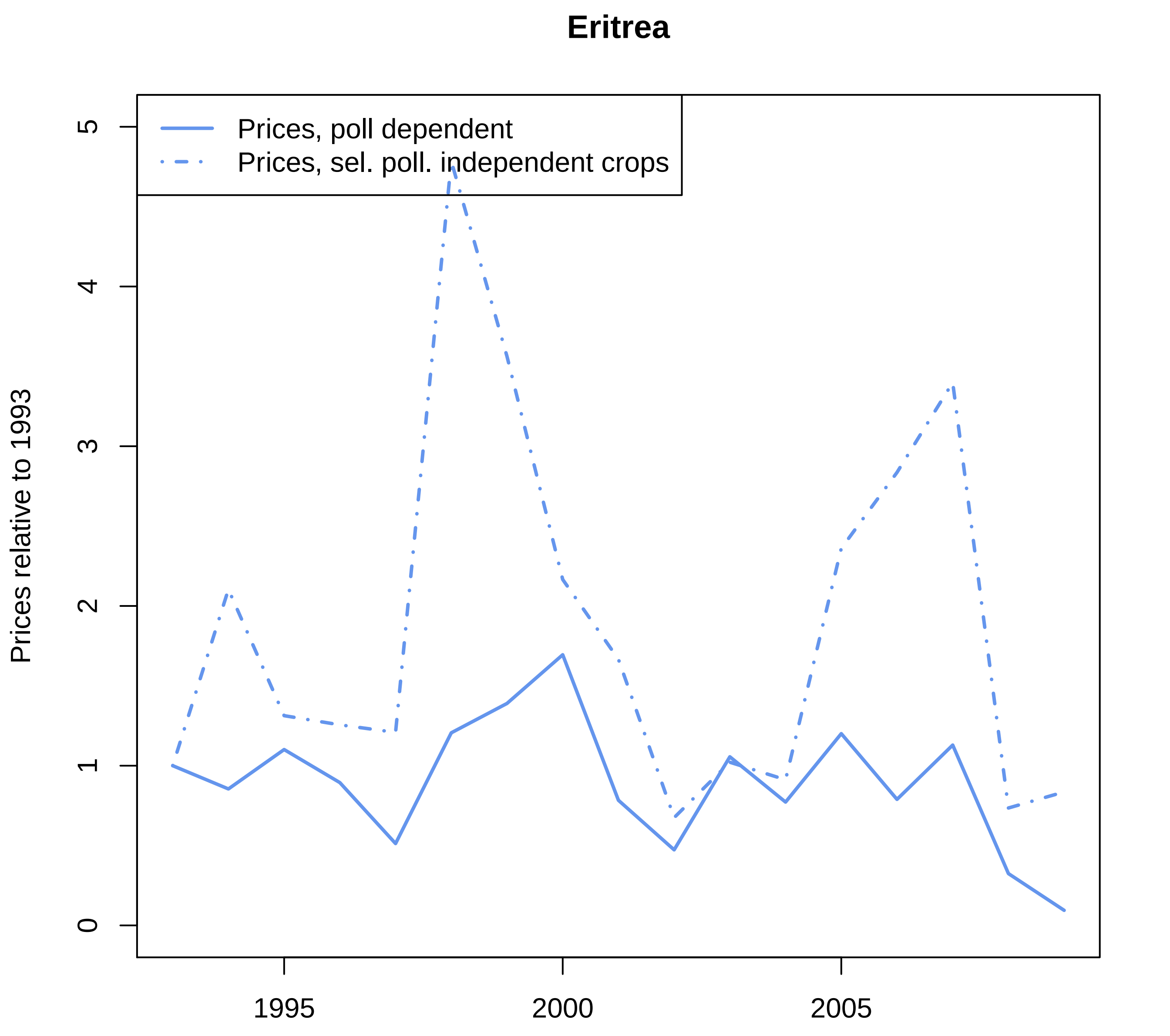

Eritrea
Prices, poll dependent
Prices, sel. poll. independent crops
Prices relative to 1993
0
1
2
3
4
5
1995
2000
2005

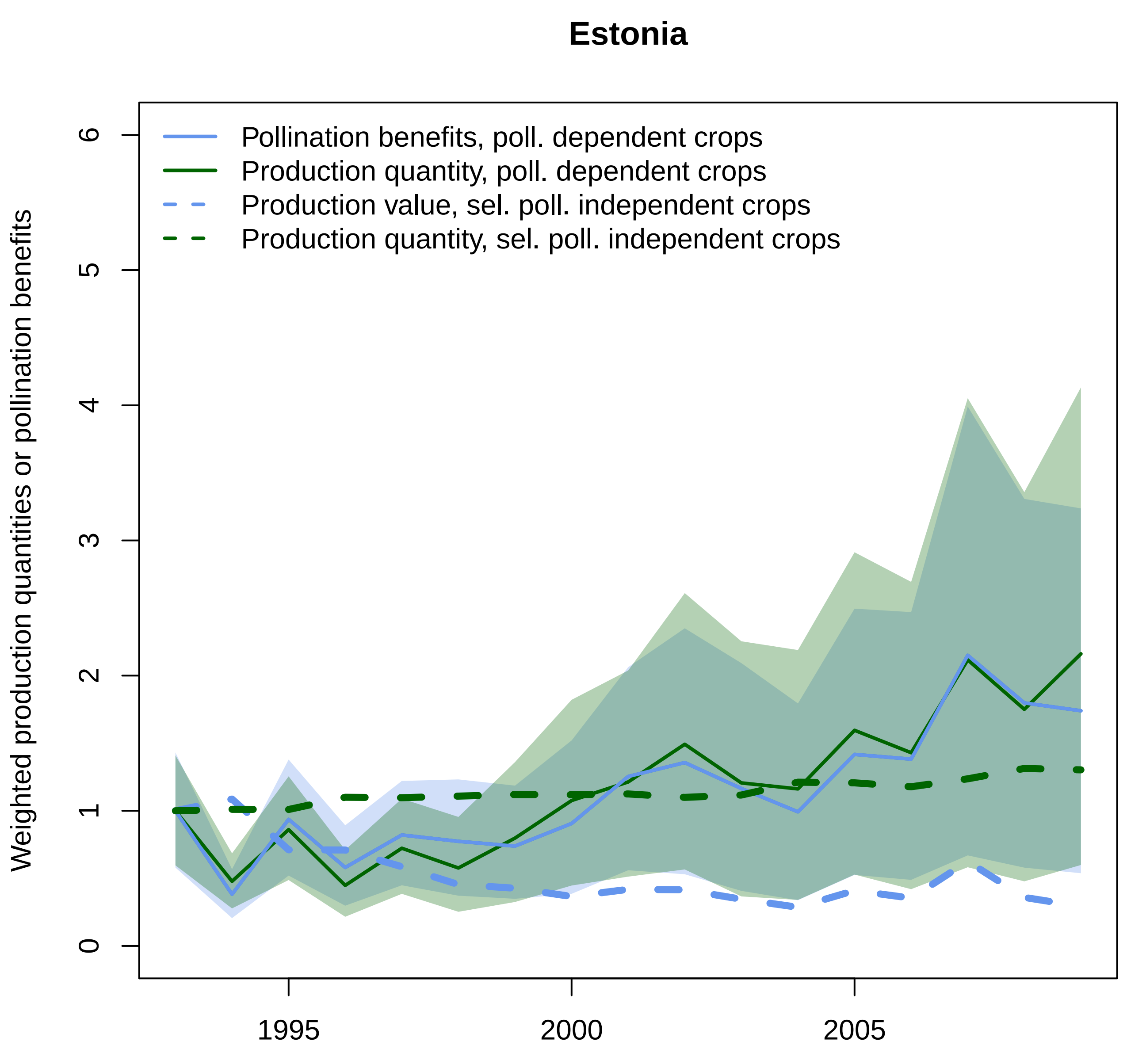

Estonia
Pollination benefits, poll. dependent crops
Production quantity, poll. dependent crops
Production value, sel. poll. independent crops
Production quantity, sel. poll. independent crops
Weighted production quantities or pollination benefits
0
1
2
3
4
5
6
1995
2000
2005

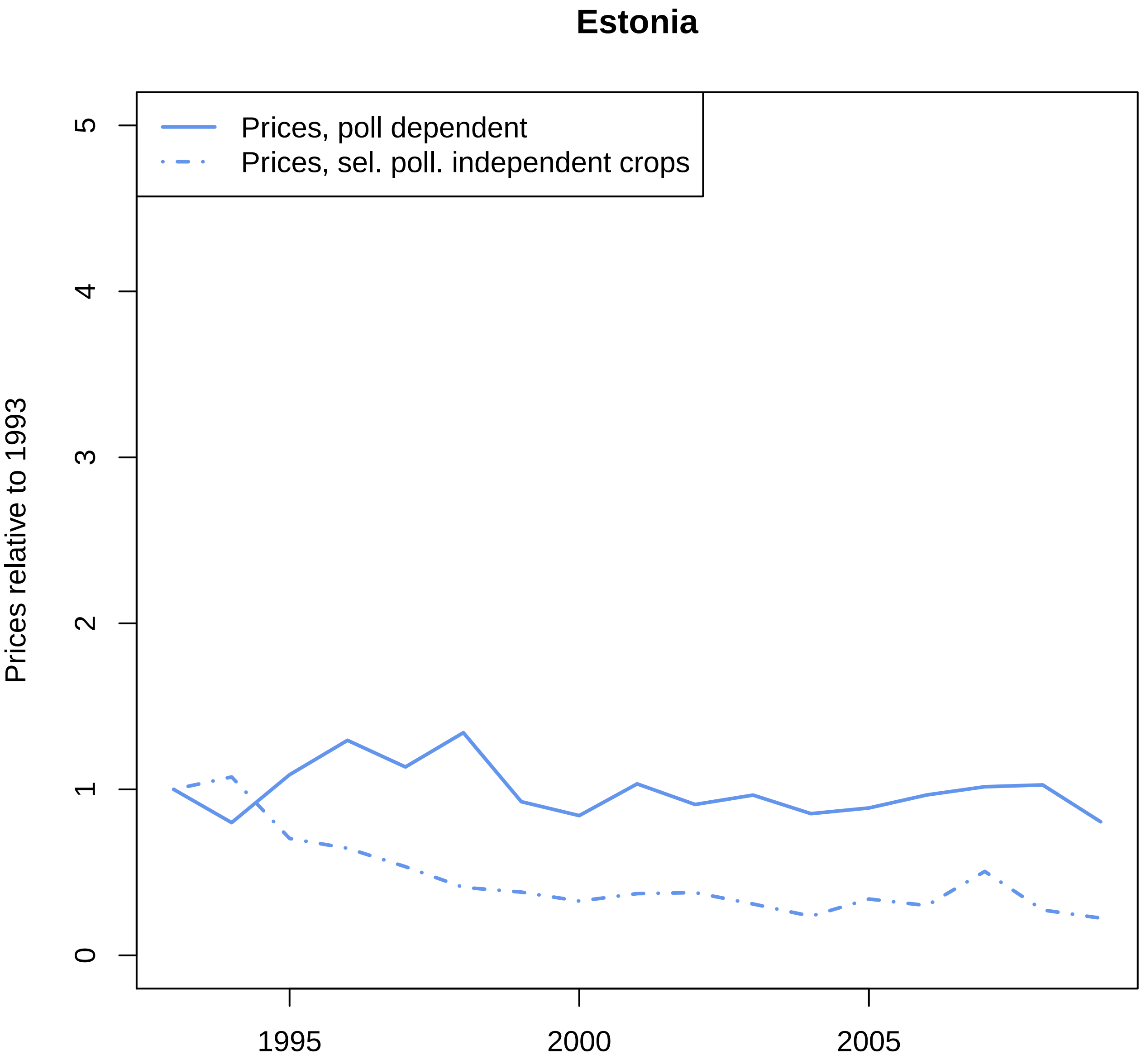
Estonia
Prices, poll dependent
Prices, sel. poll. independent crops
Prices relative to 1993
0
1
2
3
4
5
1995
2000
2005

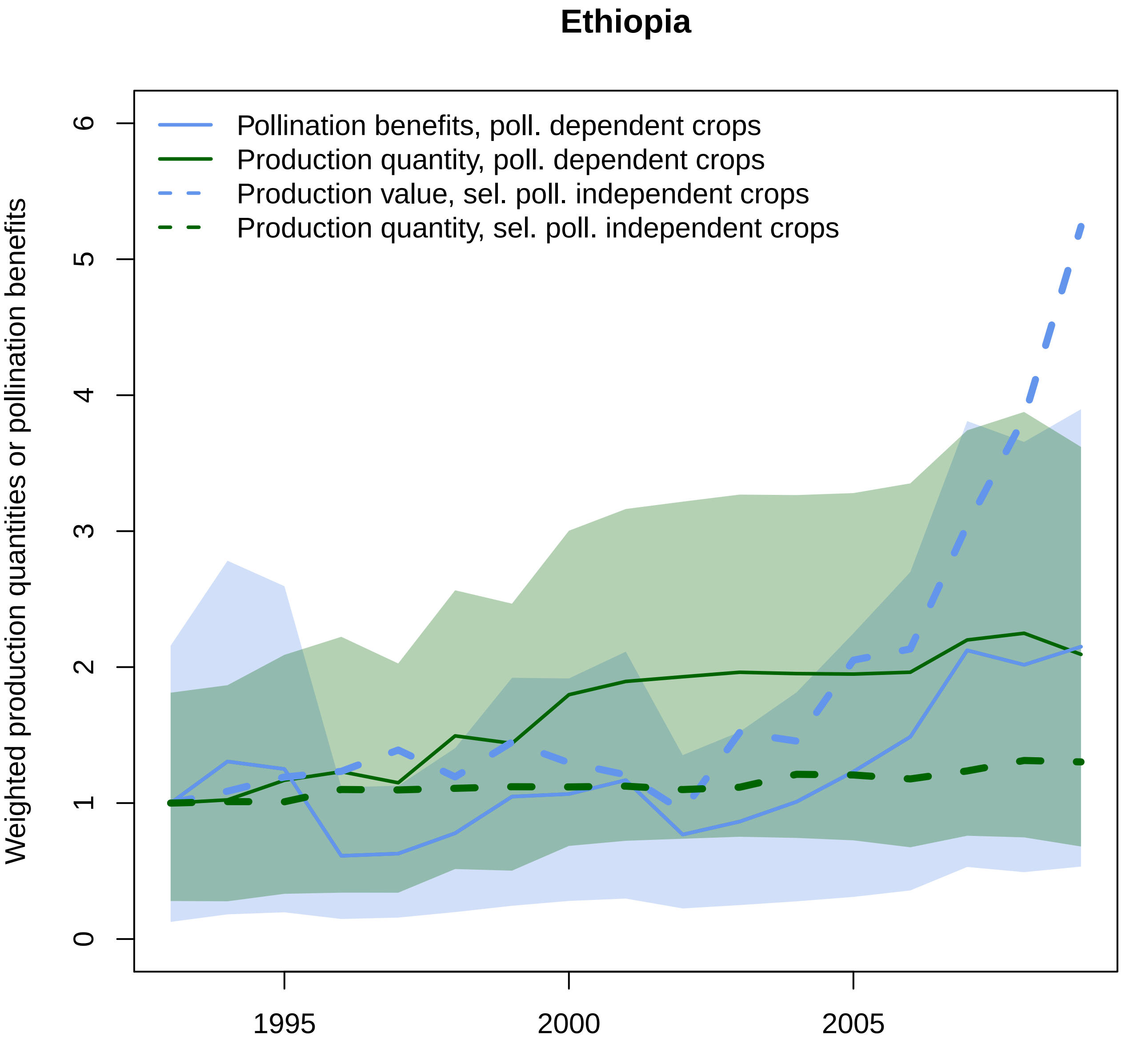

Ethiopia
Pollination benefits, poll. dependent crops
Production quantity, poll. dependent crops
Production value, sel. poll. independent crops
Production quantity, sel. poll. independent crops
Weighted production quantities or pollination benefits
0
1
2
3
4
5
6
1995
2000
2005

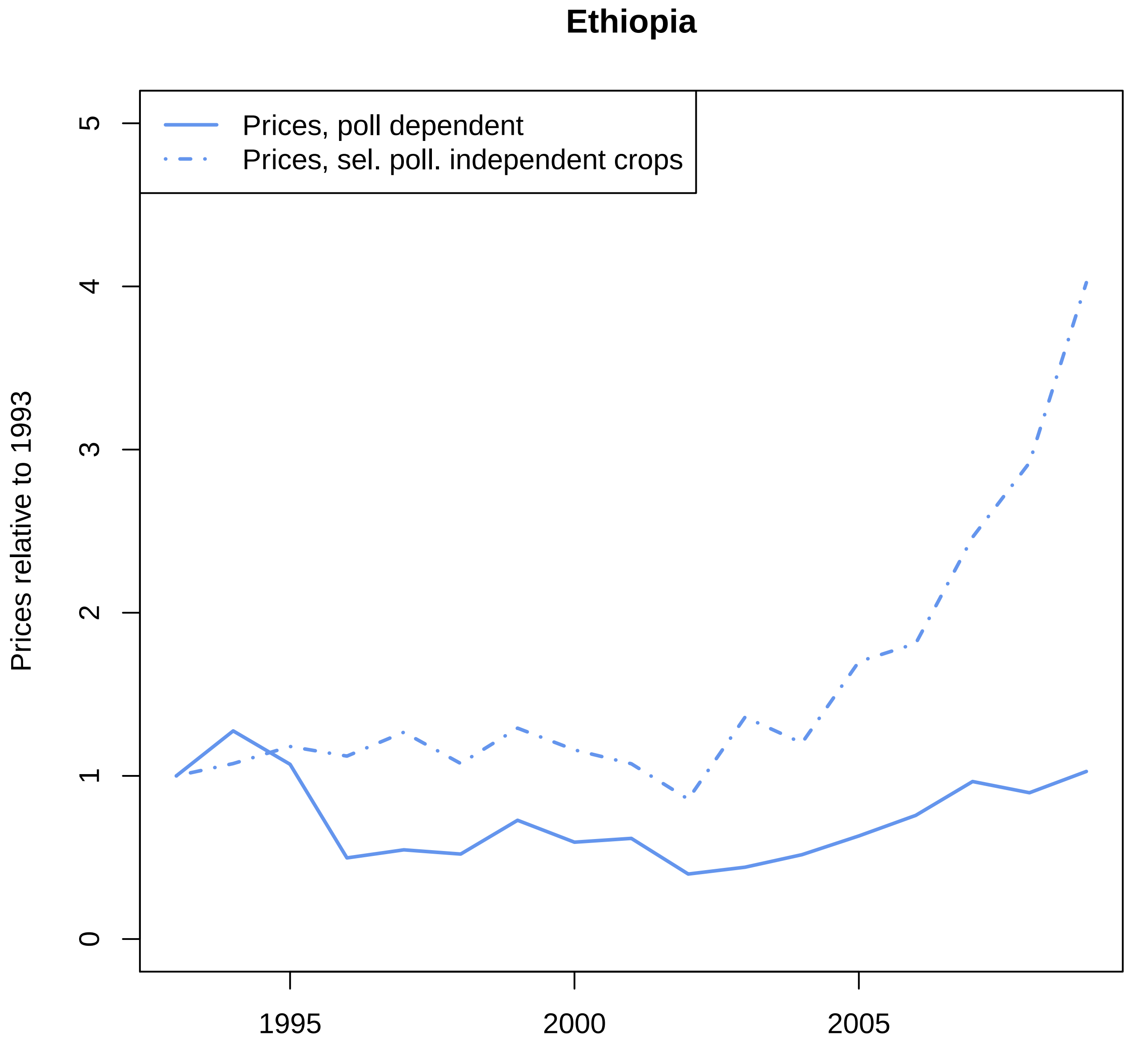

Ethiopia
Prices, poll dependent
Prices, sel. poll. independent crops
Prices relative to 1993
0
1
2
3
4
5
1995
2000
2005

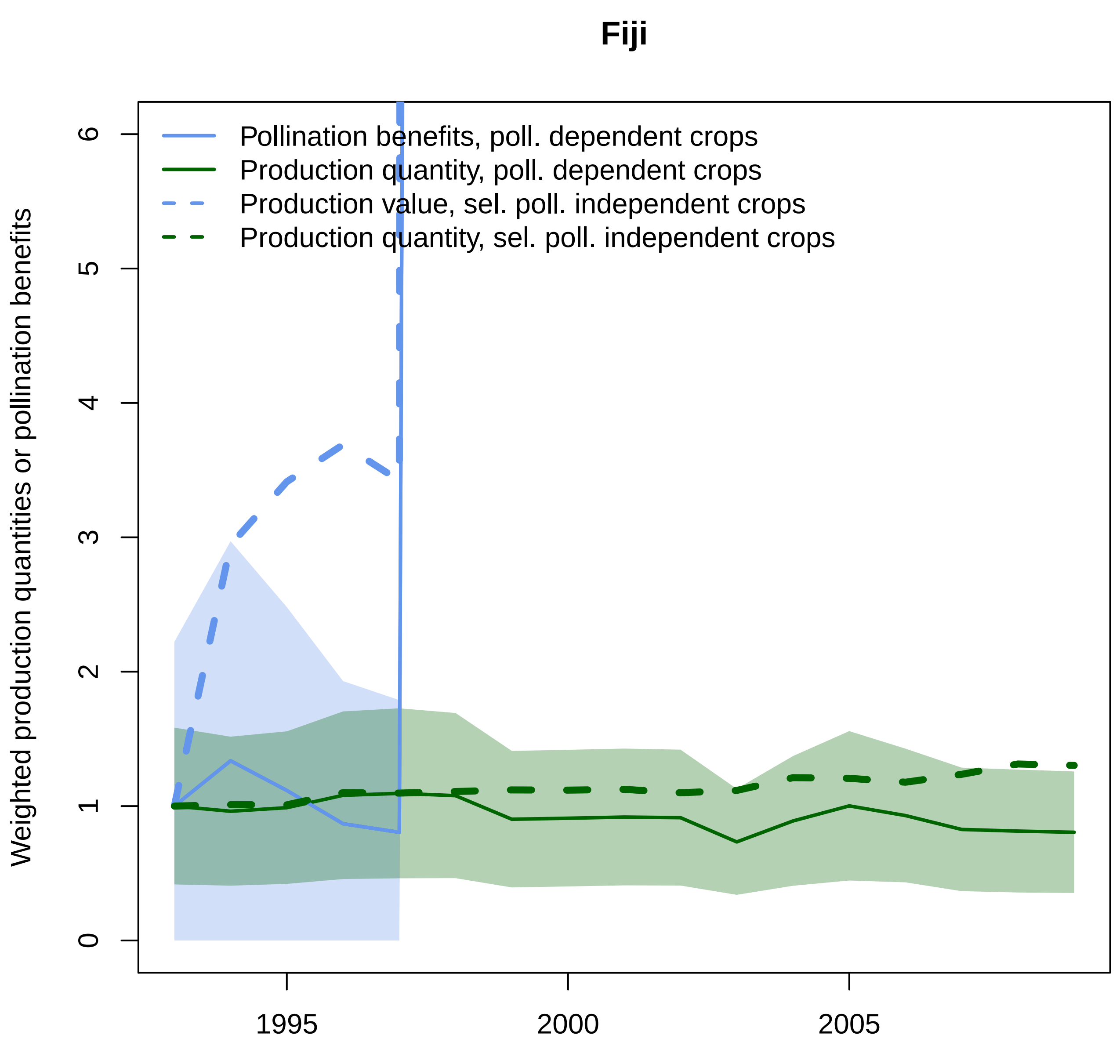

Fiji
Pollination benefits, poll. dependent crops
Production quantity, poll. dependent crops
Production value, sel. poll. independent crops
Production quantity, sel. poll. independent crops
Weighted production quantities or pollination benefits
0
1
2
3
4
5
6
1995
2000
2005

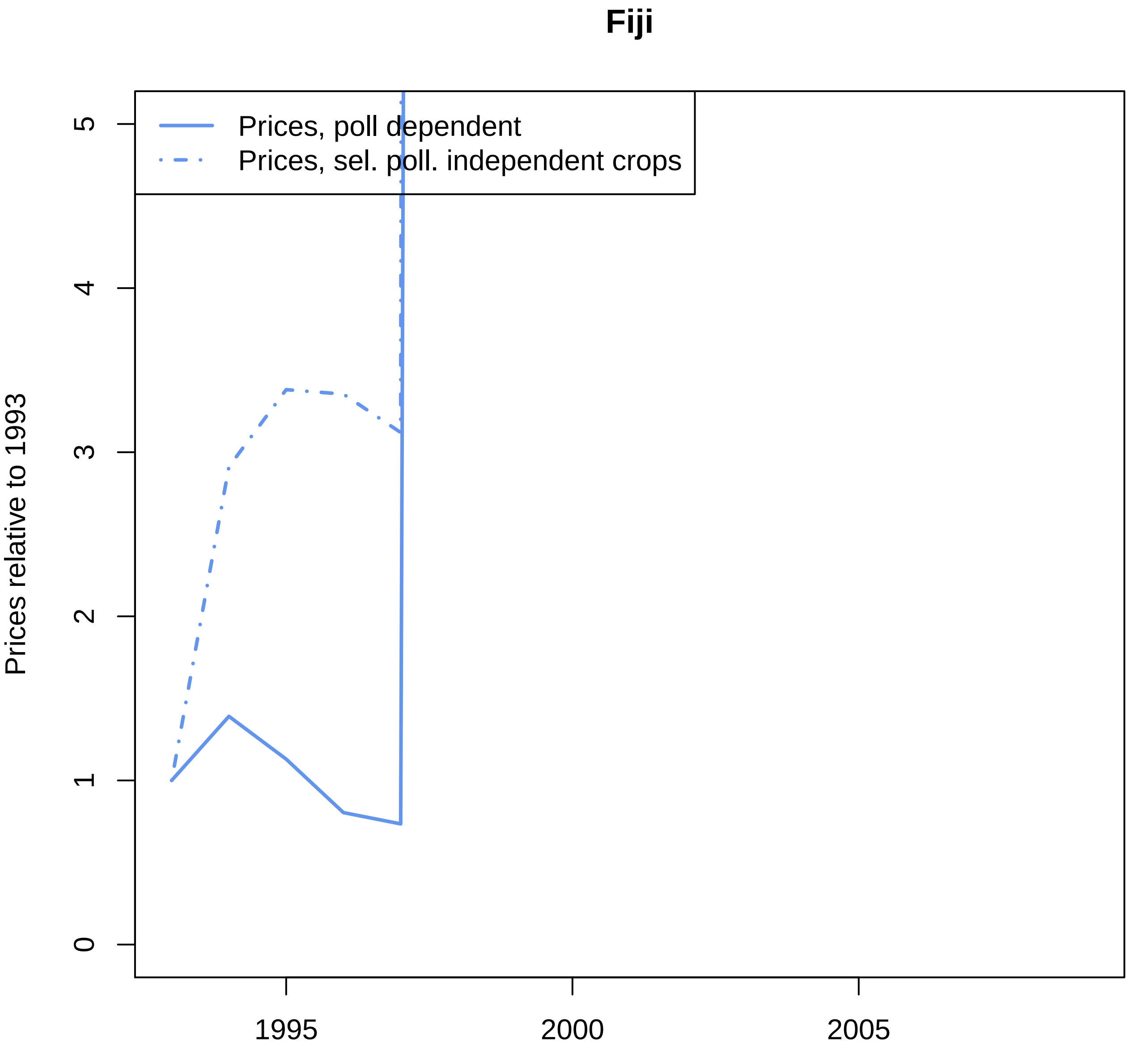
Fiji
Prices, poll dependent
Prices, sel. poll. independent crops
Prices relative to 1993
0
1
2
3
4
5
1995
2000
2005

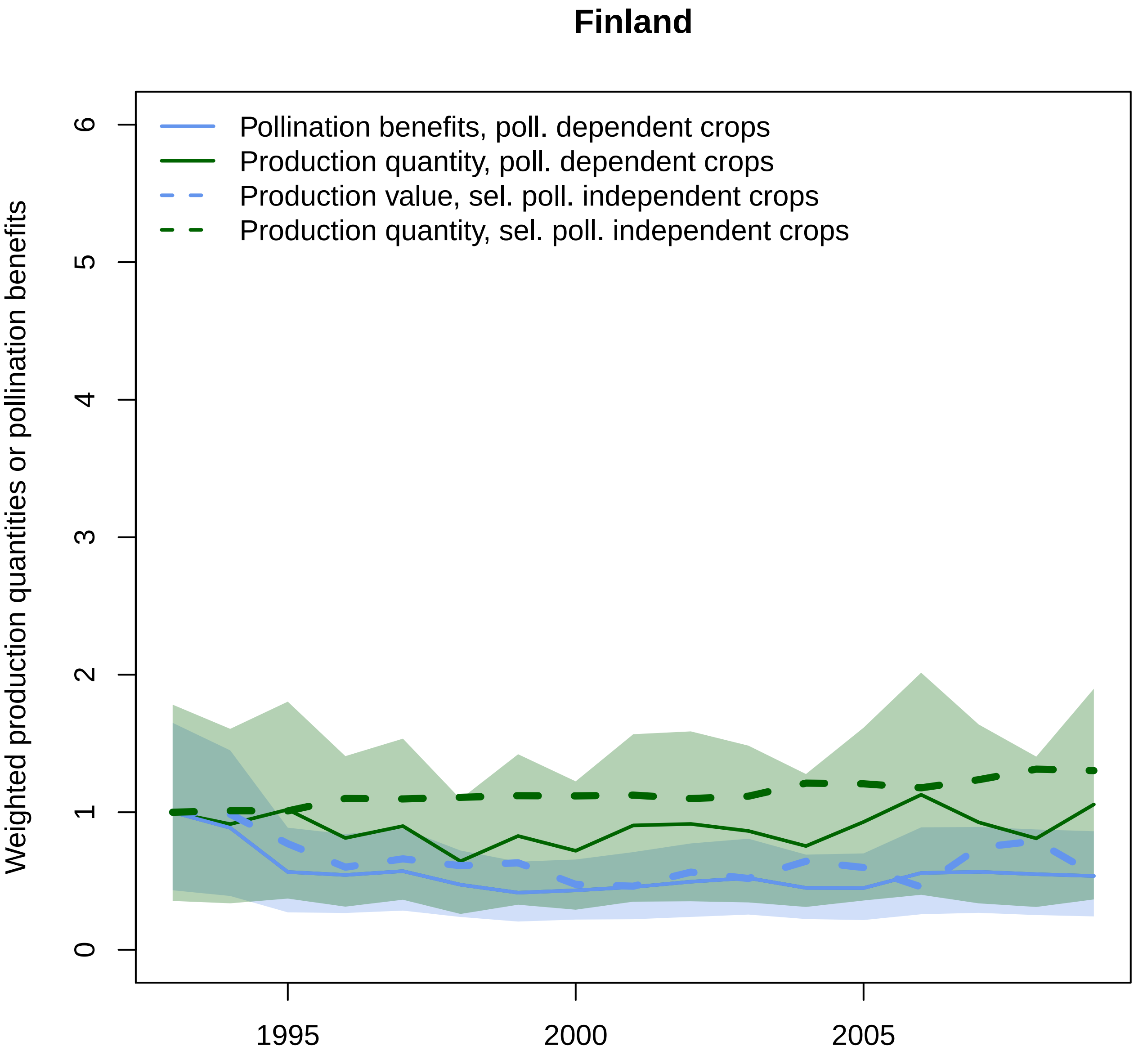
Finland
Pollination benefits, poll. dependent crops
Production quantity, poll. dependent crops
Production value, sel. poll. independent crops
Production quantity, sel. poll. independent crops
Weighted production quantities or pollination benefits
0
1
2
3
4
5
6
1995
2000
2005

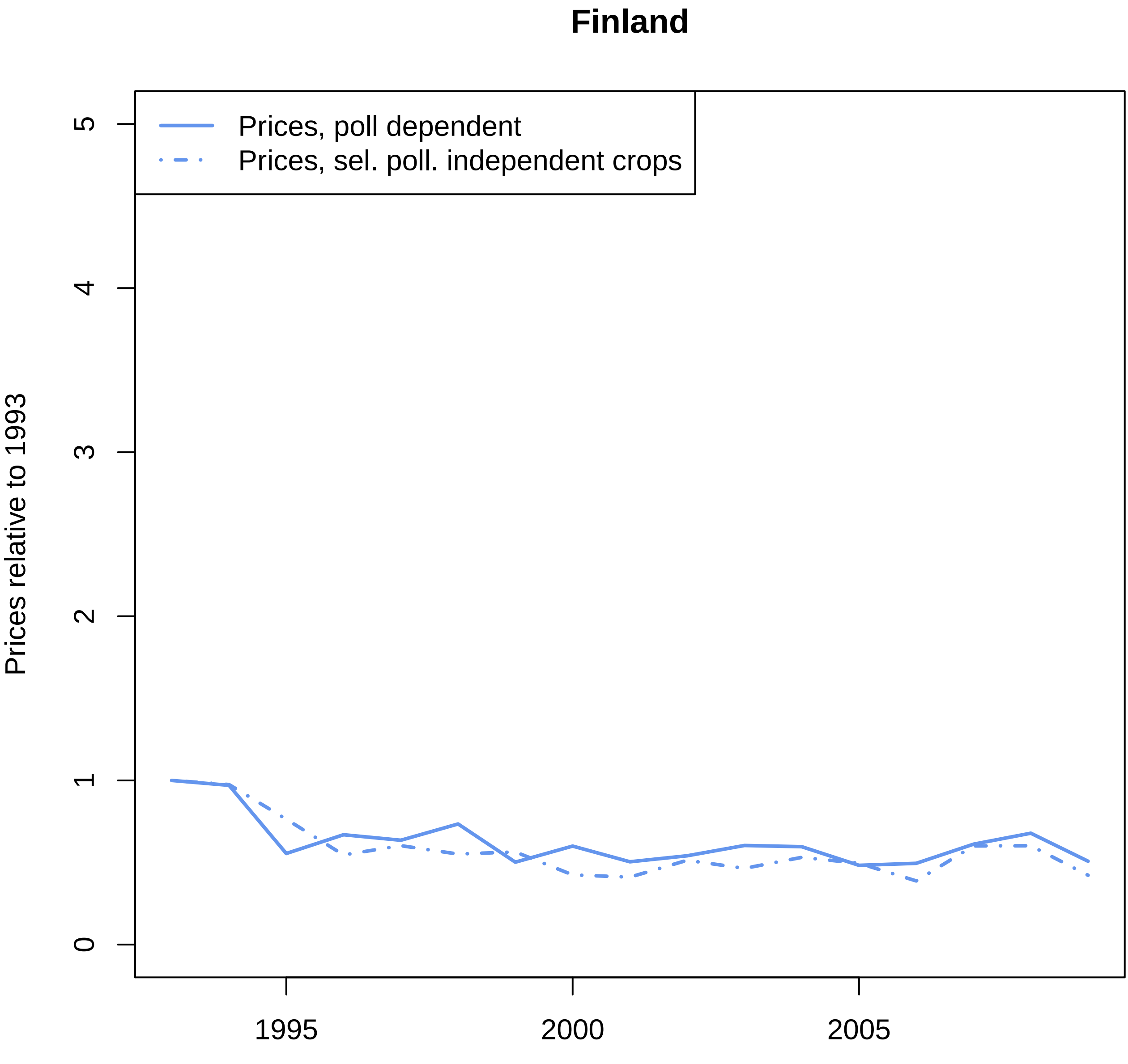

Finland
Prices, poll dependent
Prices, sel. poll. independent crops
Prices relative to 1993
0
1
2
3
4
5
1995
2000
2005

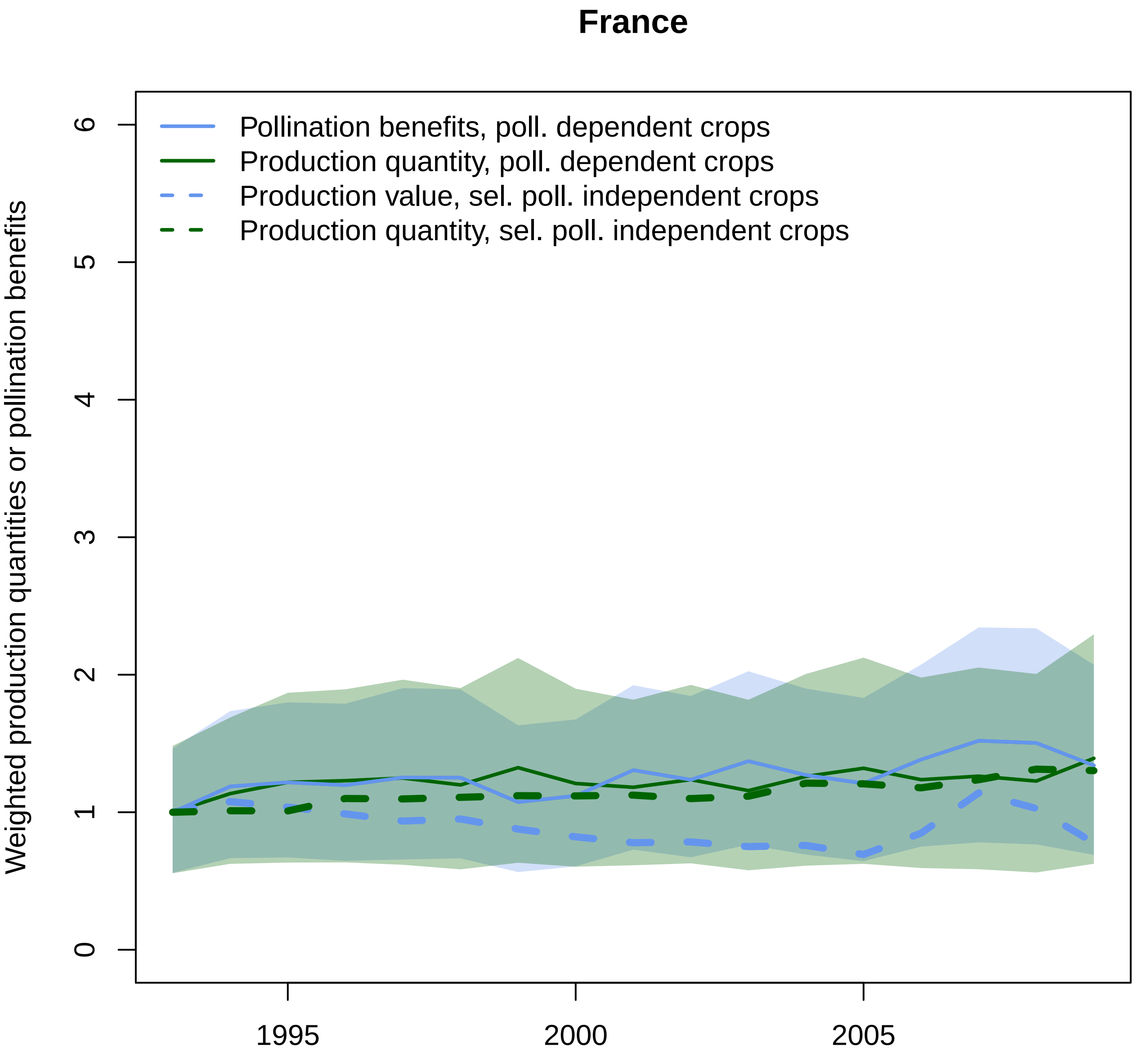
France
Pollination benefits, poll. dependent crops
Production quantity, poll. dependent crops
Production value, sel. poll. independent crops
Production quantity, sel. poll. independent crops
Weighted production quantities or pollination benefits
0
1
2
3
4
5
6
1995
2000
2005

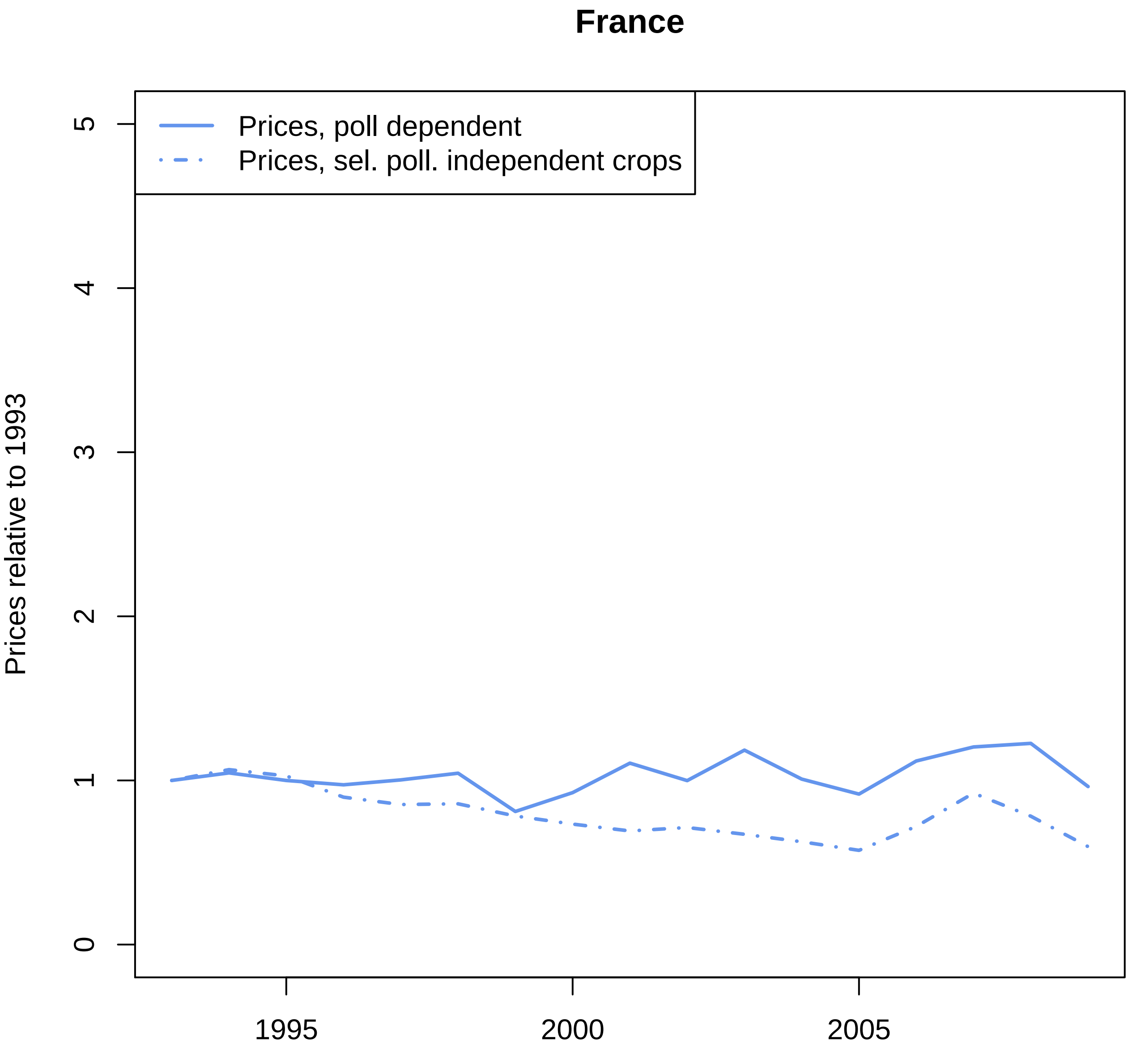
France
Prices, poll dependent
Prices, sel. poll. independent crops
Prices relative to 1993
0
1
2
3
4
5
1995
2000
2005

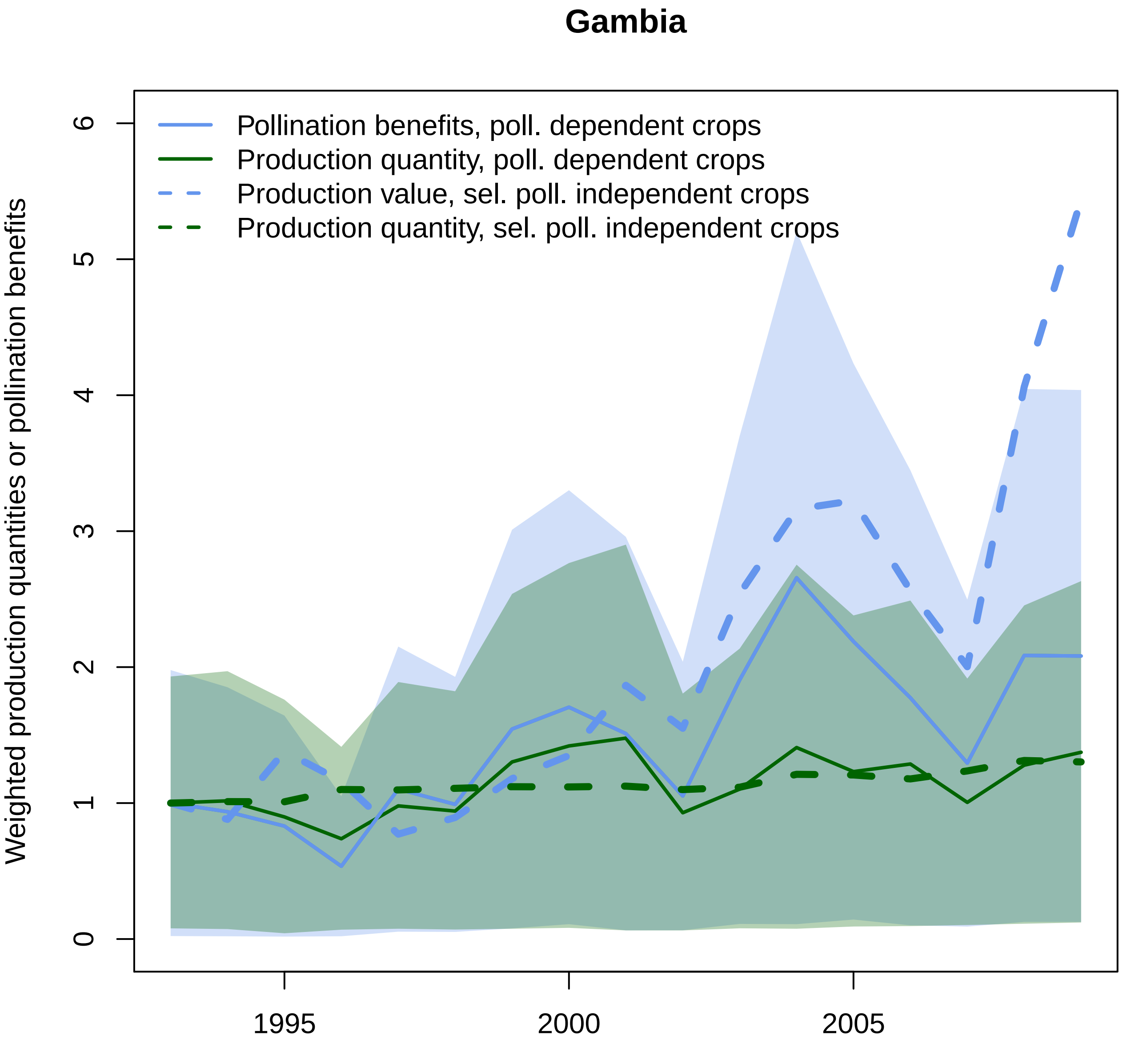

Gambia
Pollination benefits, poll. dependent crops
Production quantity, poll. dependent crops
Production value, sel. poll. independent crops
Production quantity, sel. poll. independent crops
Weighted production quantities or pollination benefits
0
1
2
3
4
5
6
1995
2000
2005

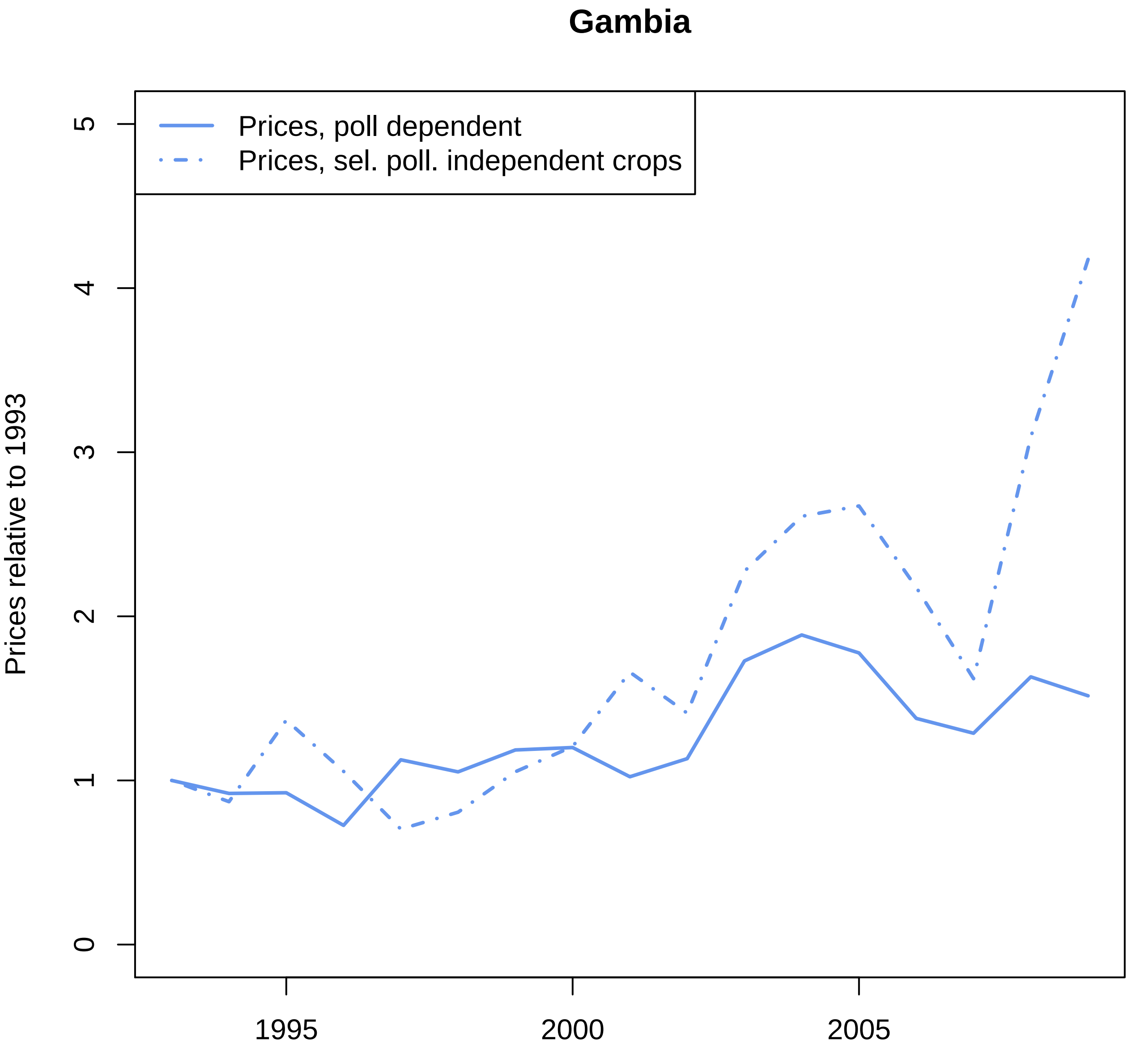

Gambia
Prices, poll dependent
Prices, sel. poll. independent crops
Prices relative to 1993
0
1
2
3
4
5
1995
2000
2005

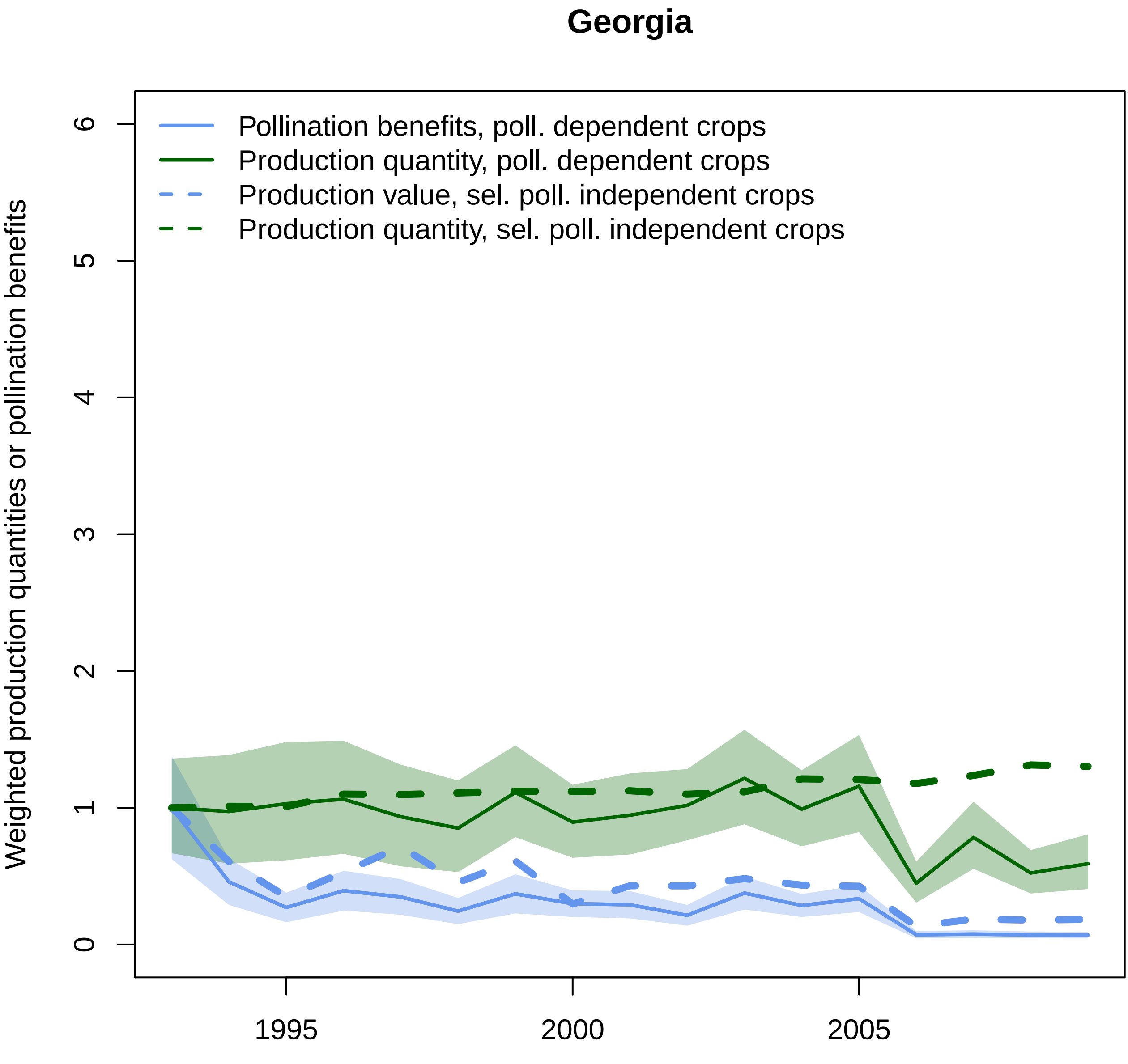
Georgia
Pollination benefits, poll. dependent crops
Production quantity, poll. dependent crops
Production value, sel. poll. independent crops
Production quantity, sel. poll. independent crops
Weighted production quantities or pollination benefits
0
1
2
3
4
5
6
1995
2000
2005

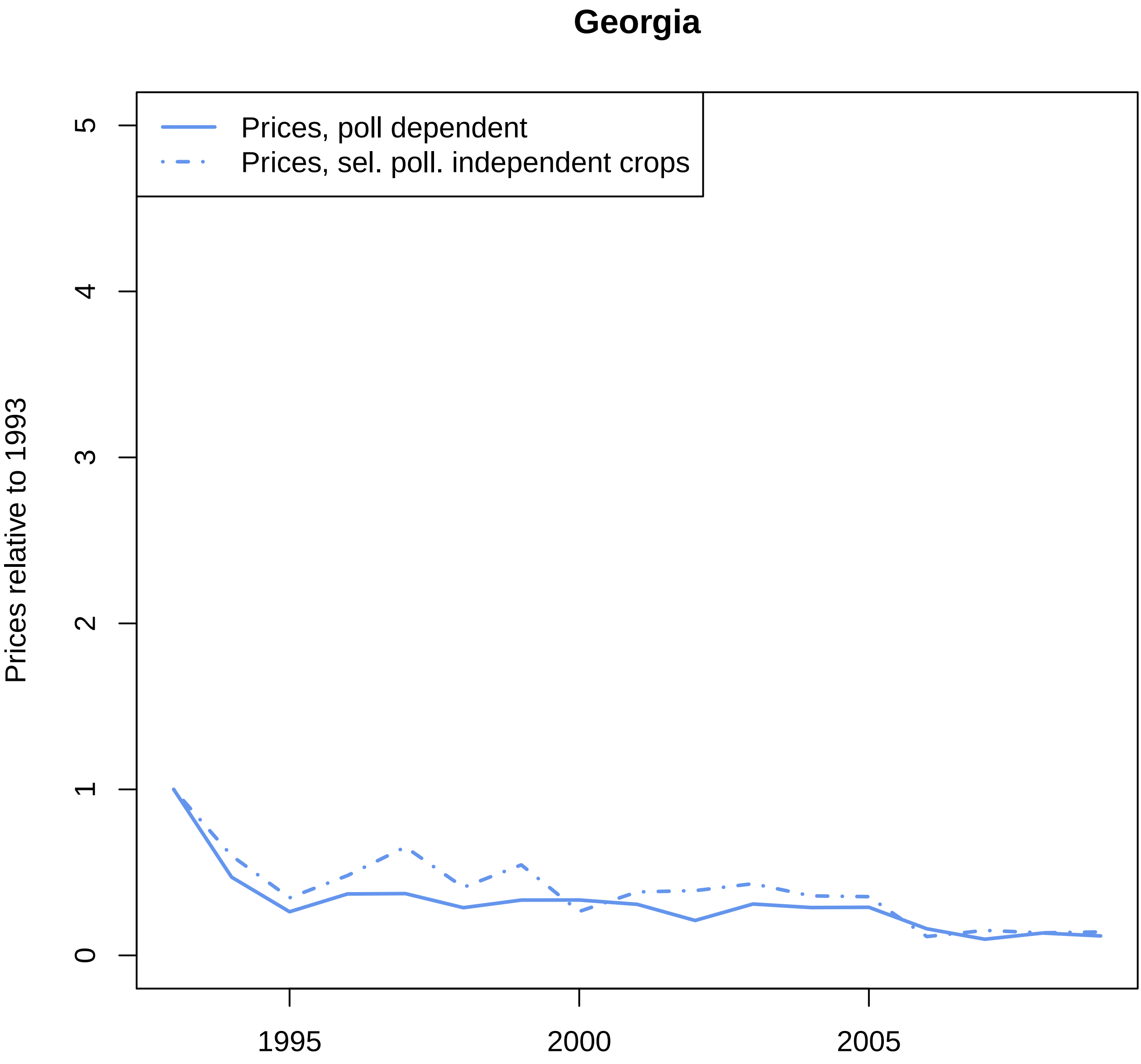

Georgia
Prices, poll dependent
Prices, sel. poll. independent crops
Prices relative to 1993
0
1
2
3
4
5
1995
2000
2005

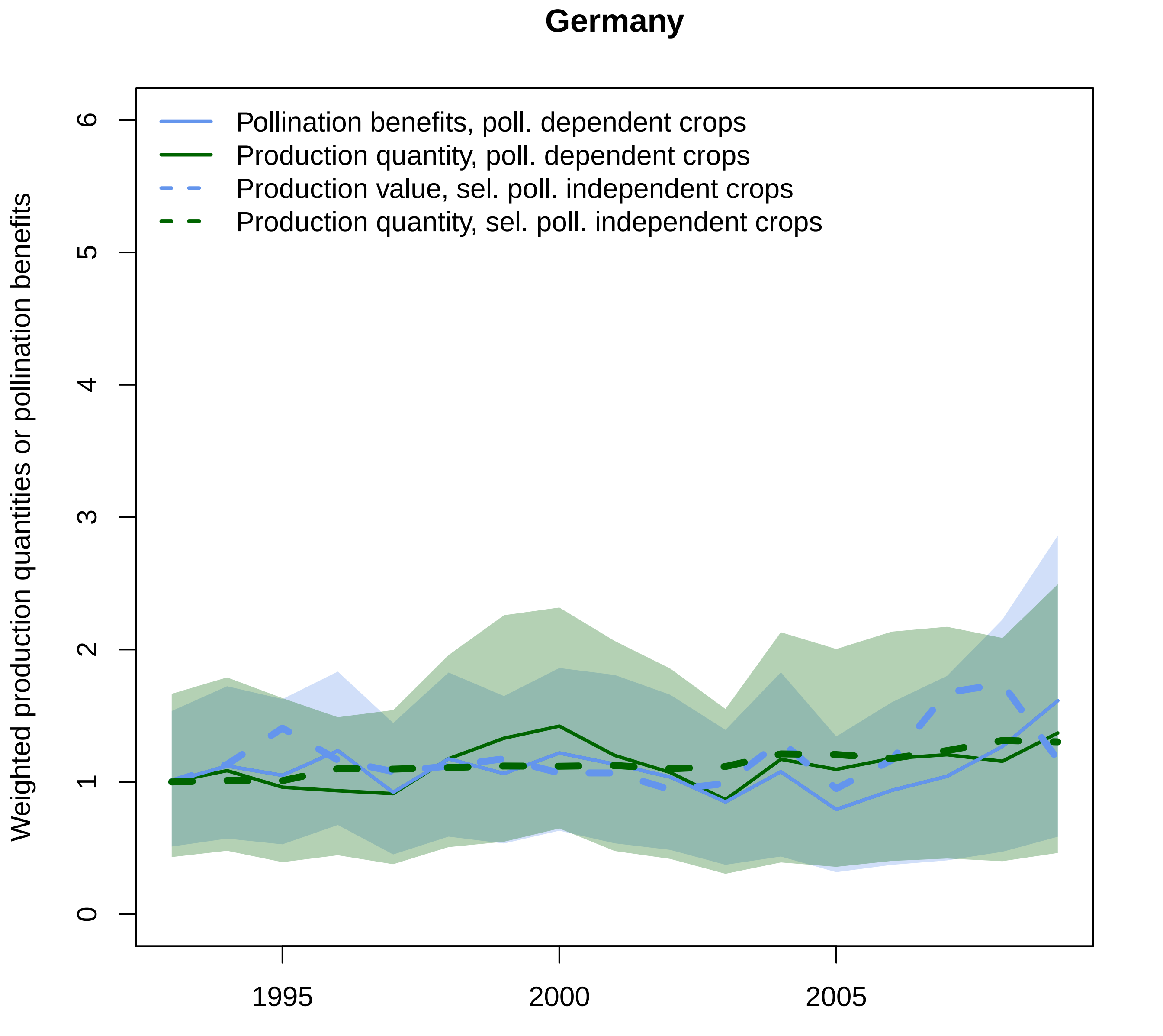
Germany
Pollination benefits, poll. dependent crops
Production quantity, poll. dependent crops
Production value, sel. poll. independent crops
Production quantity, sel. poll. independent crops
Weighted production quantities or pollination benefits
0
1
2
3
4
5
6
1995
2000
2005

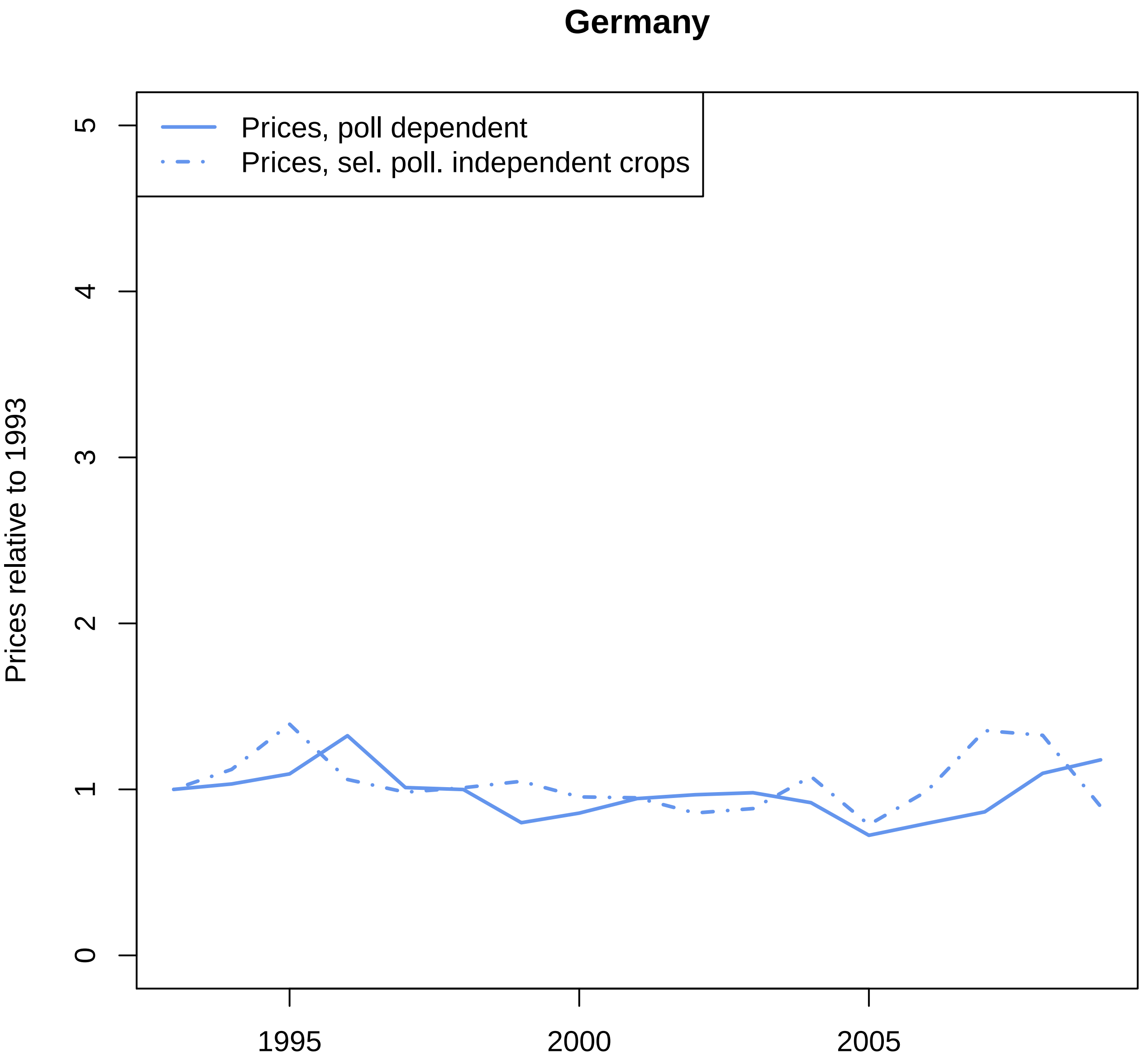

Germany
Prices, poll dependent
Prices, sel. poll. independent crops
Prices relative to 1993
0
1
2
3
4
5
1995
2000
2005

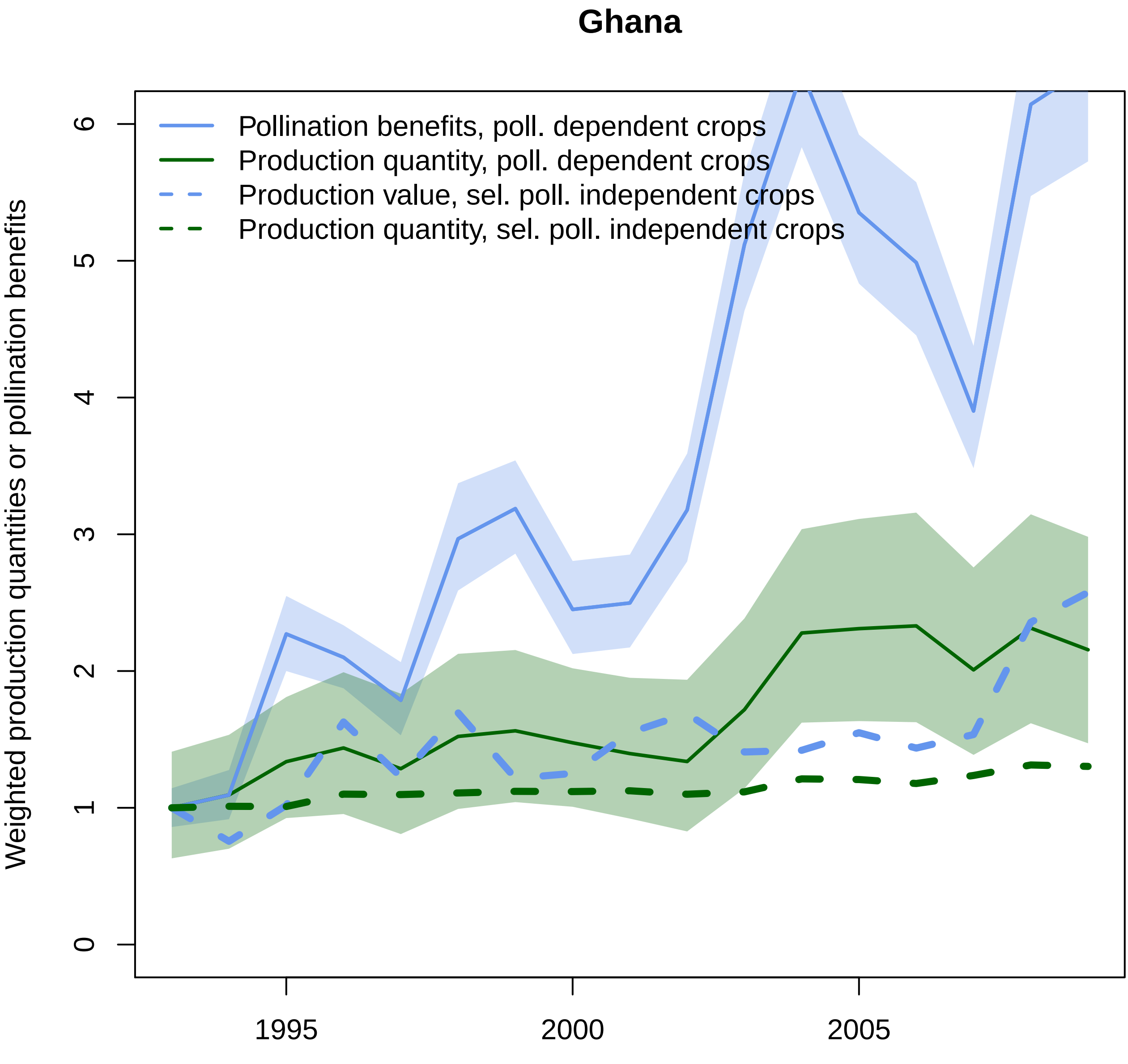

Ghana
Pollination benefits, poll. dependent crops
Production quantity, poll. dependent crops
Production value, sel. poll. independent crops
Production quantity, sel. poll. independent crops
Weighted production quantities or pollination benefits
0
1
2
3
4
5
6
1995
2000
2005

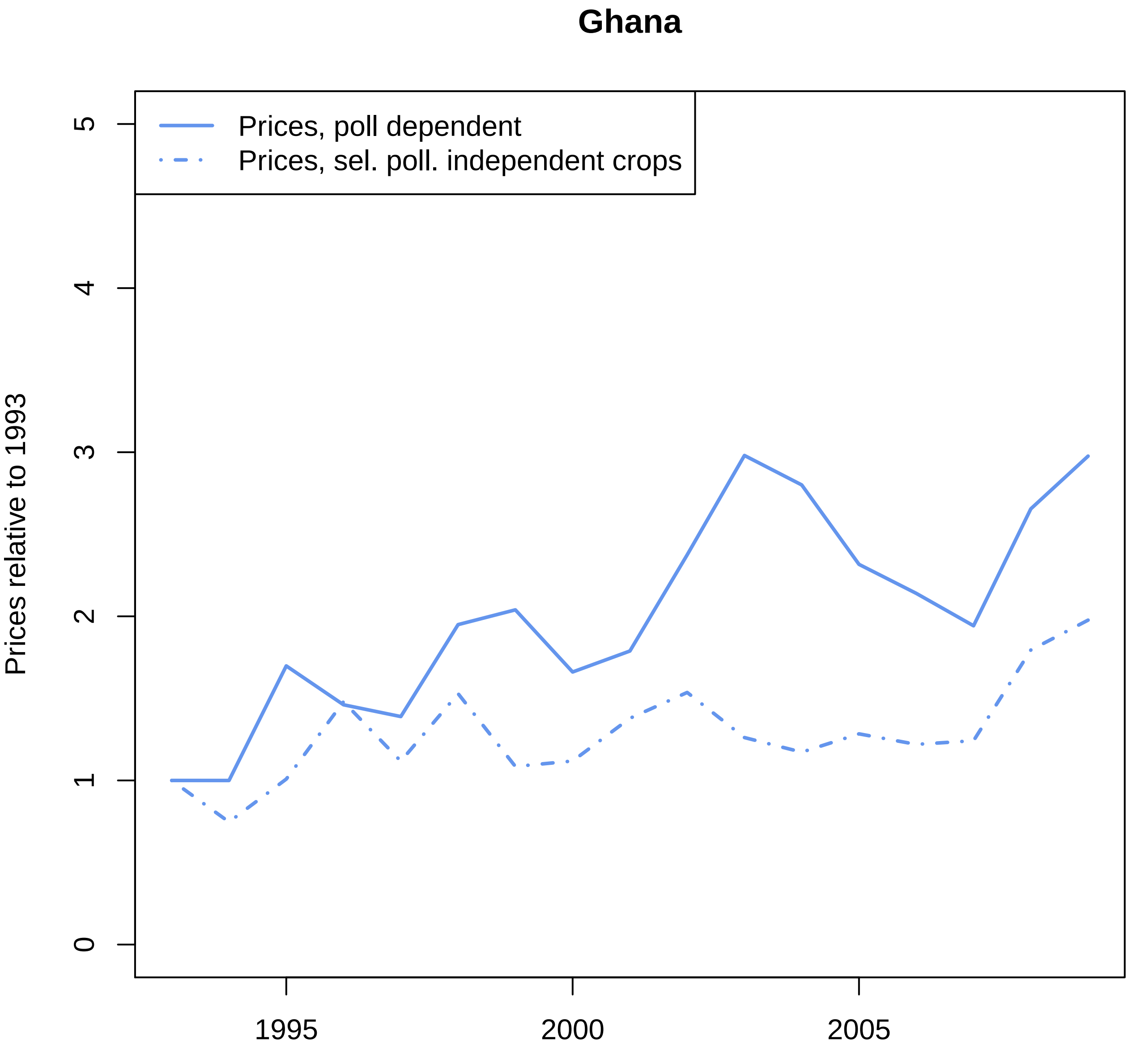
Ghana
Prices, poll dependent
Prices, sel. poll. independent crops
Prices relative to 1993
0
1
2
3
4
5
1995
2000
2005

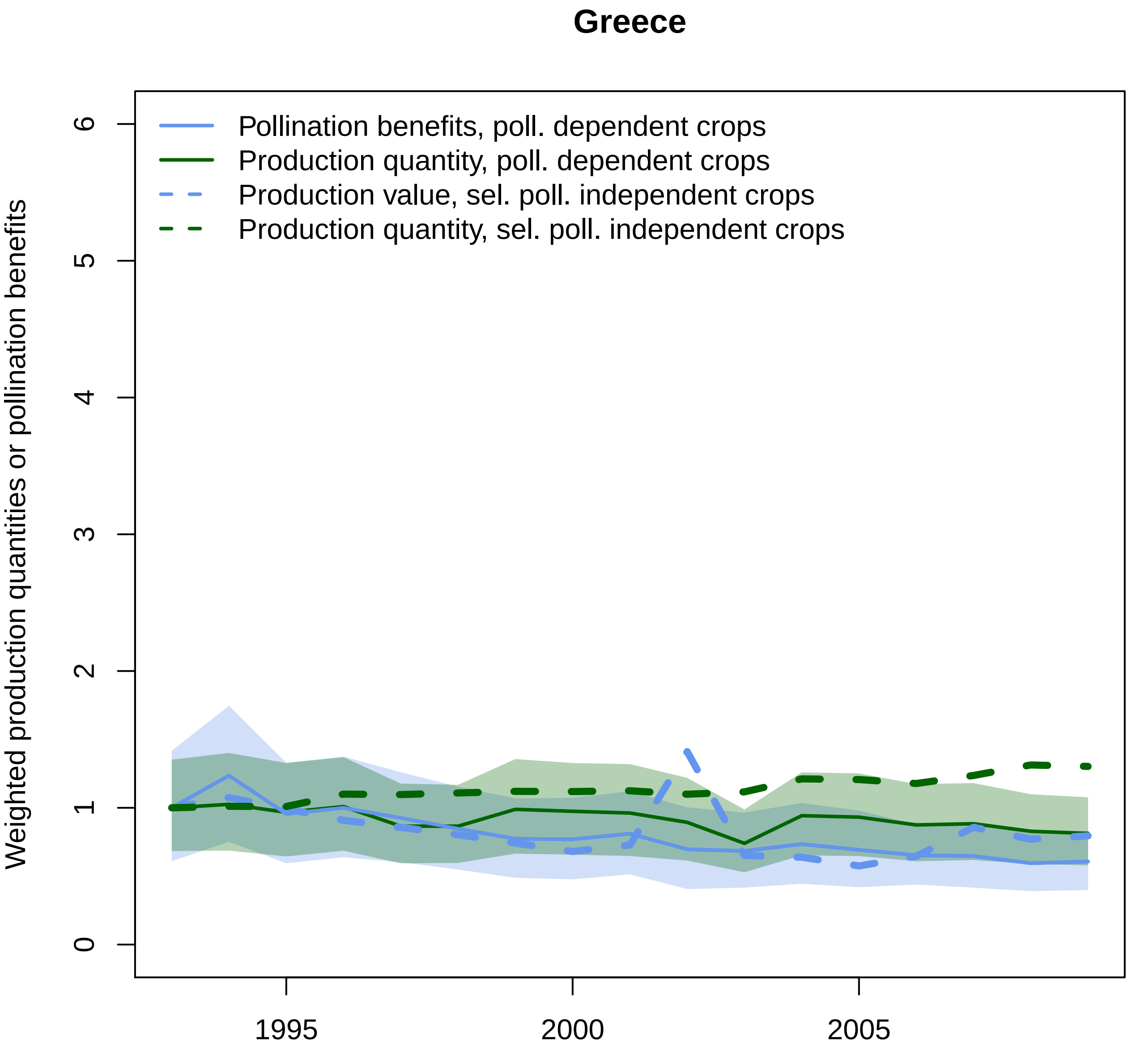

Greece
Pollination benefits, poll. dependent crops
Production quantity, poll. dependent crops
Production value, sel. poll. independent crops
Production quantity, sel. poll. independent crops
Weighted production quantities or pollination benefits
0
1
2
3
4
5
6
1995
2000
2005

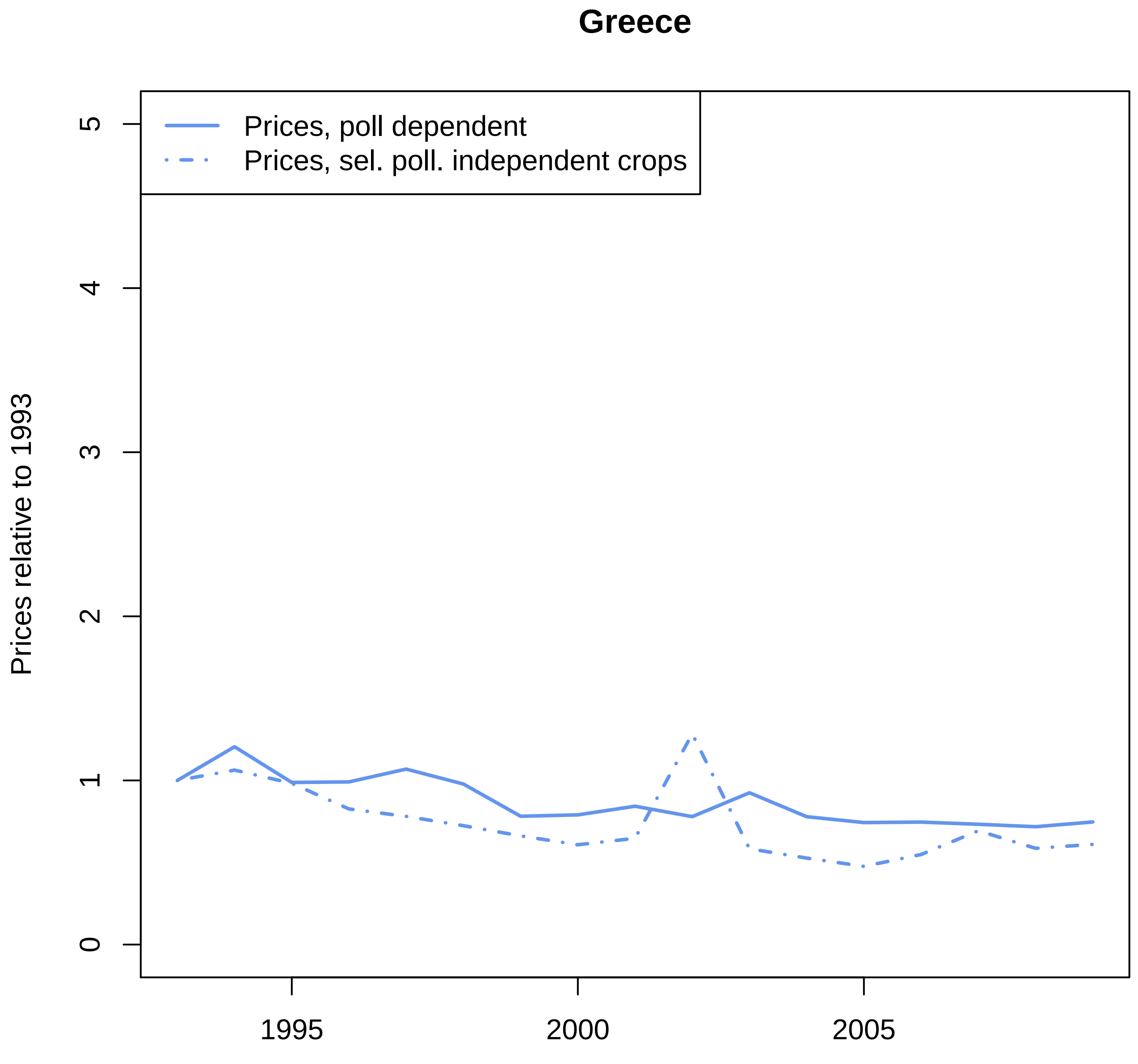

Greece
Prices, poll dependent
Prices, sel. poll. independent crops
Prices relative to 1993
0
1
2
3
4
5
1995
2000
2005

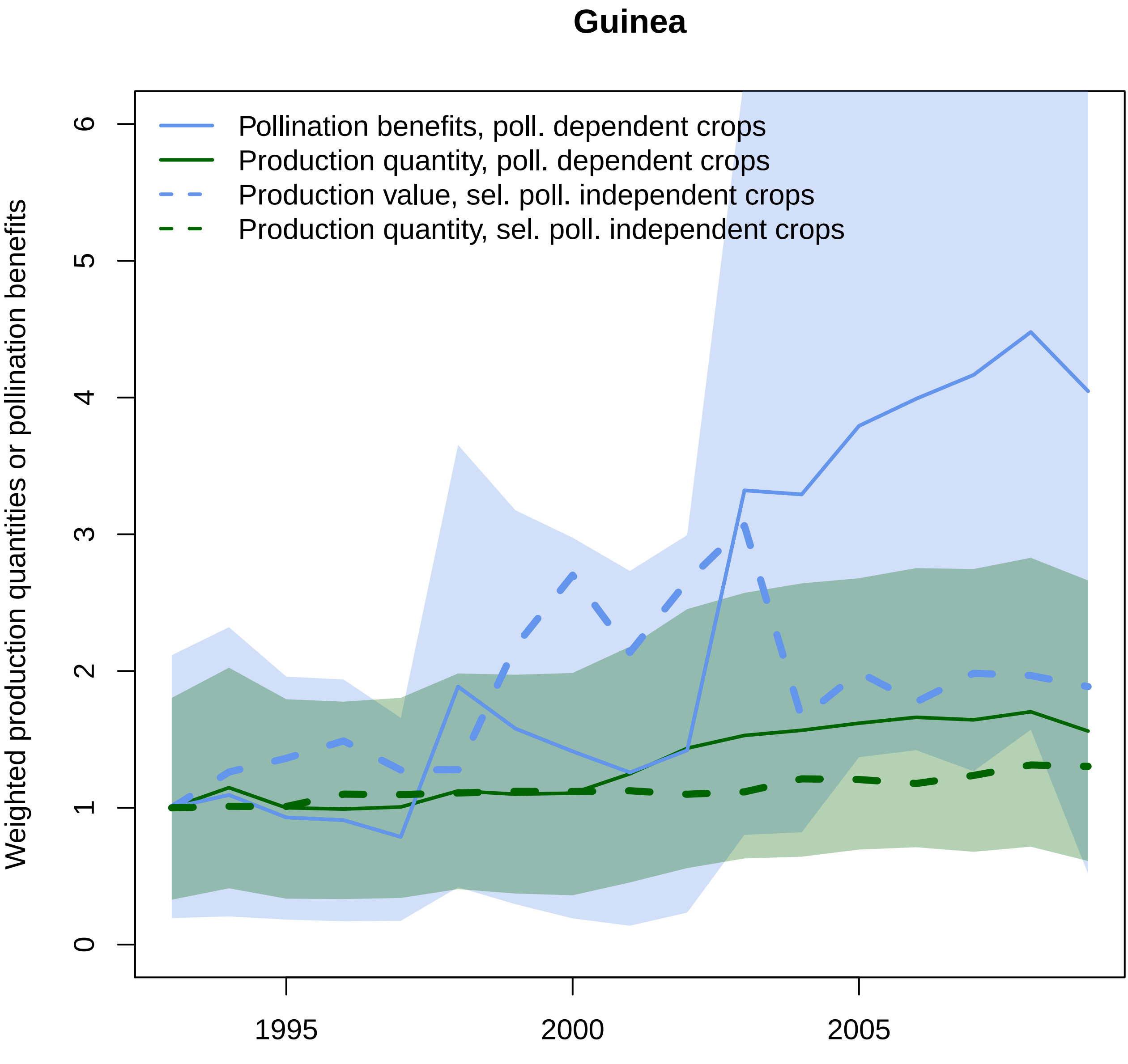
Guinea
Pollination benefits, poll. dependent crops
Production quantity, poll. dependent crops
Production value, sel. poll. independent crops
Production quantity, sel. poll. independent crops
Weighted production quantities or pollination benefits
0
1
2
3
4
5
6
1995
2000
2005

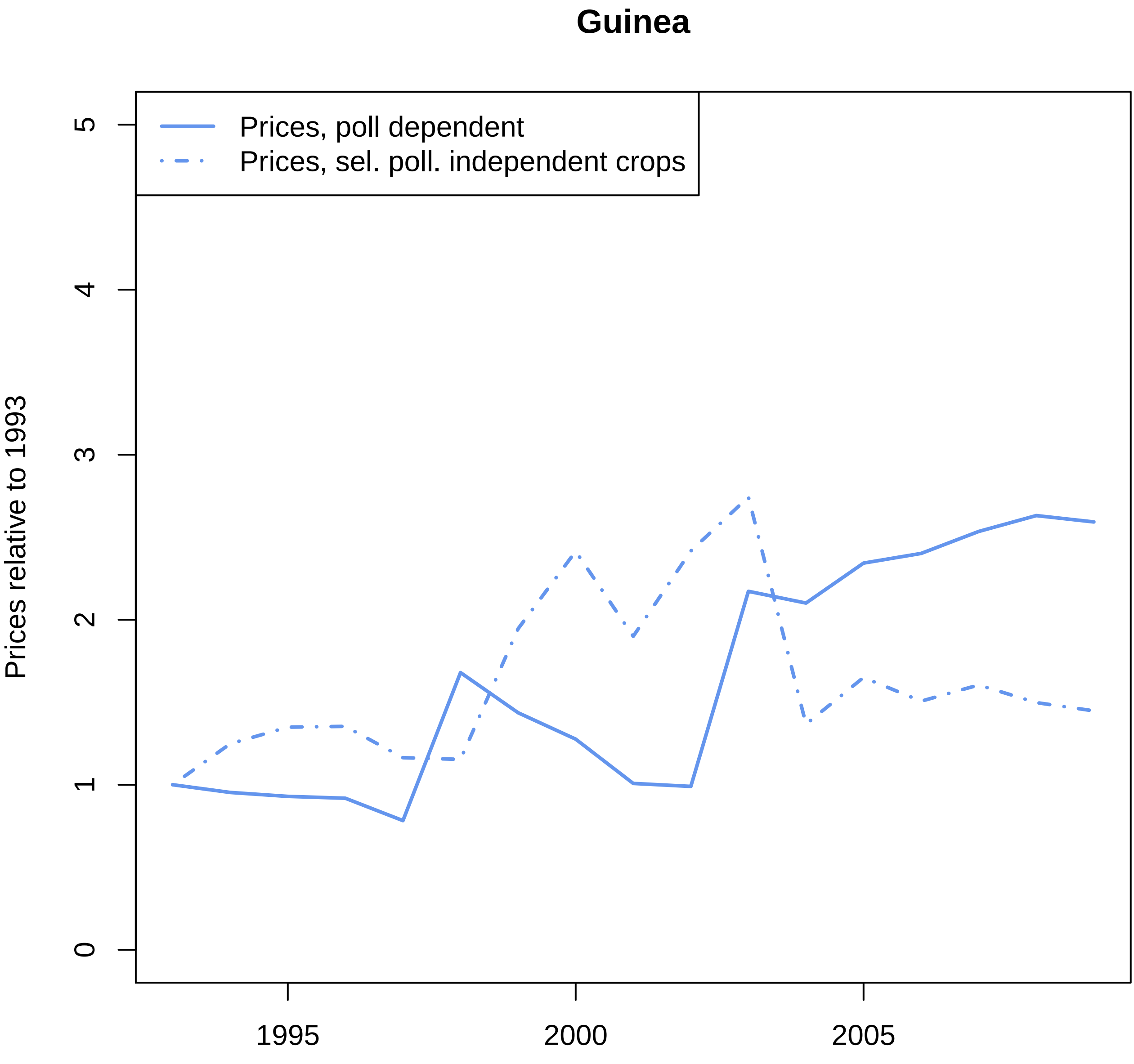

Guinea
Prices, poll dependent
Prices, sel. poll. independent crops
Prices relative to 1993
0
1
2
3
4
5
1995
2000
2005

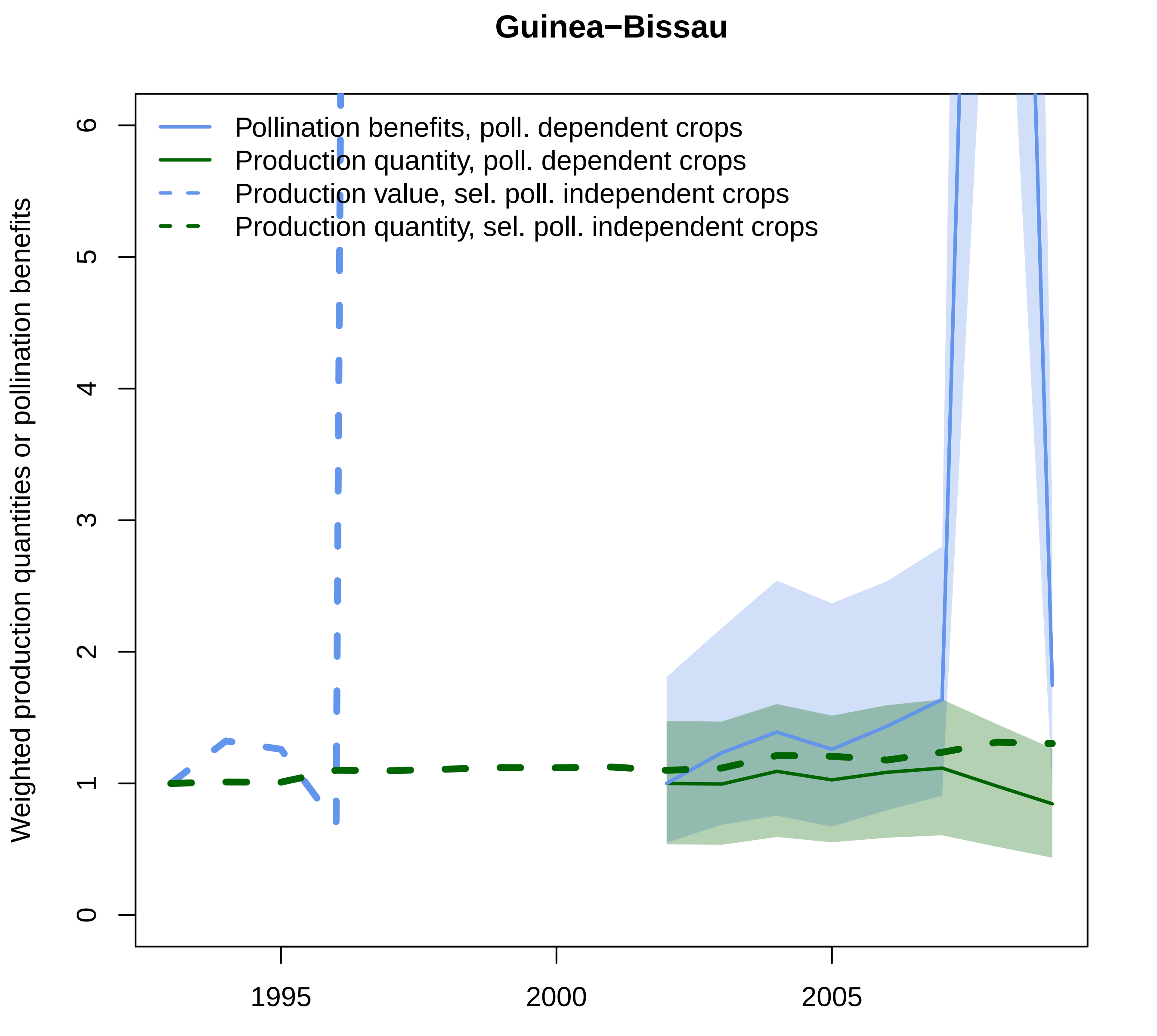

Guinea−Bissau
Pollination benefits, poll. dependent crops
Production quantity, poll. dependent crops
Production value, sel. poll. independent crops
Production quantity, sel. poll. independent crops
Weighted production quantities or pollination benefits
0
1
2
3
4
5
6
1995
2000
2005

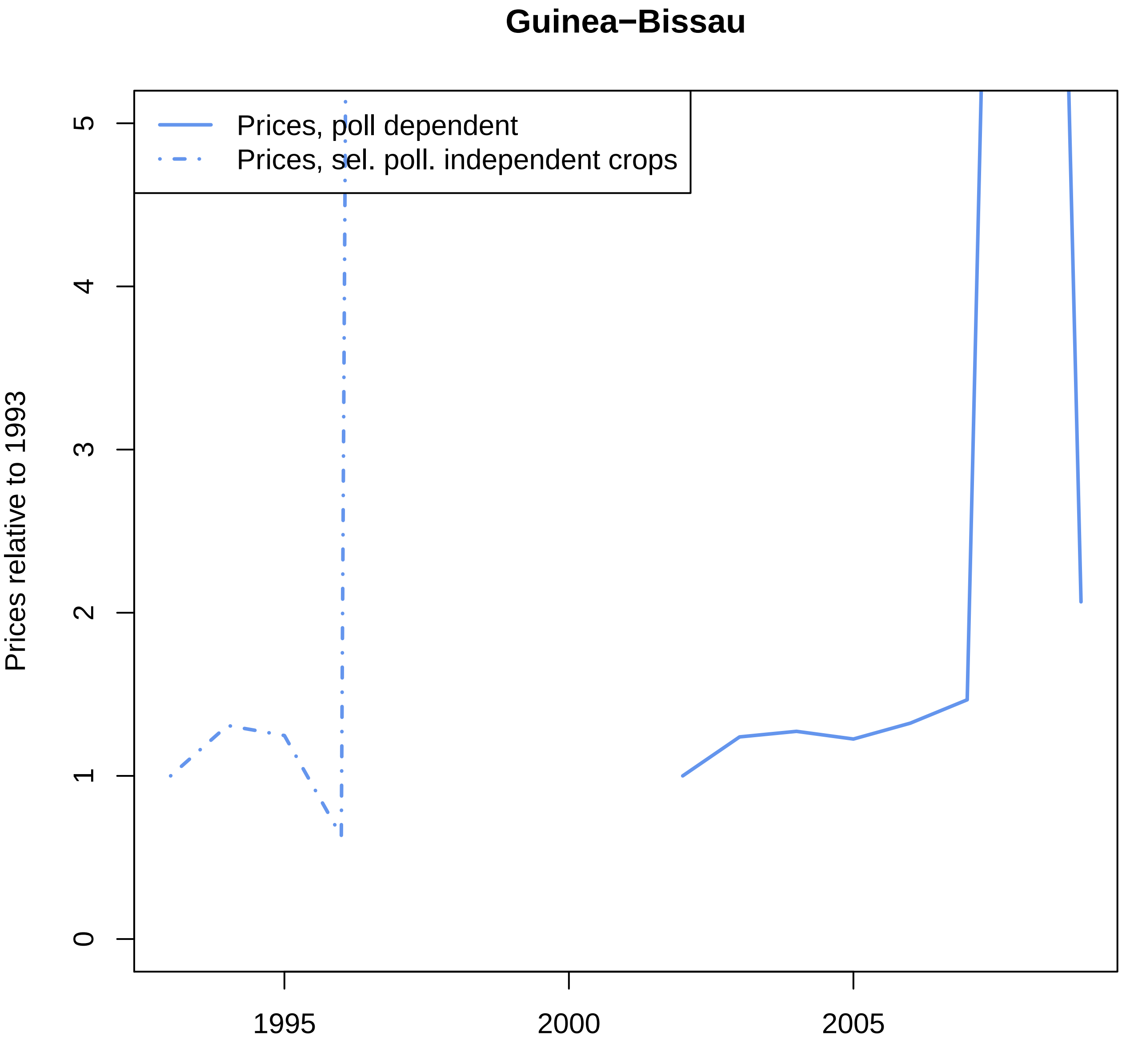

Guinea−Bissau
Prices, poll dependent
Prices, sel. poll. independent crops
Prices relative to 1993
0
1
2
3
4
5
1995
2000
2005

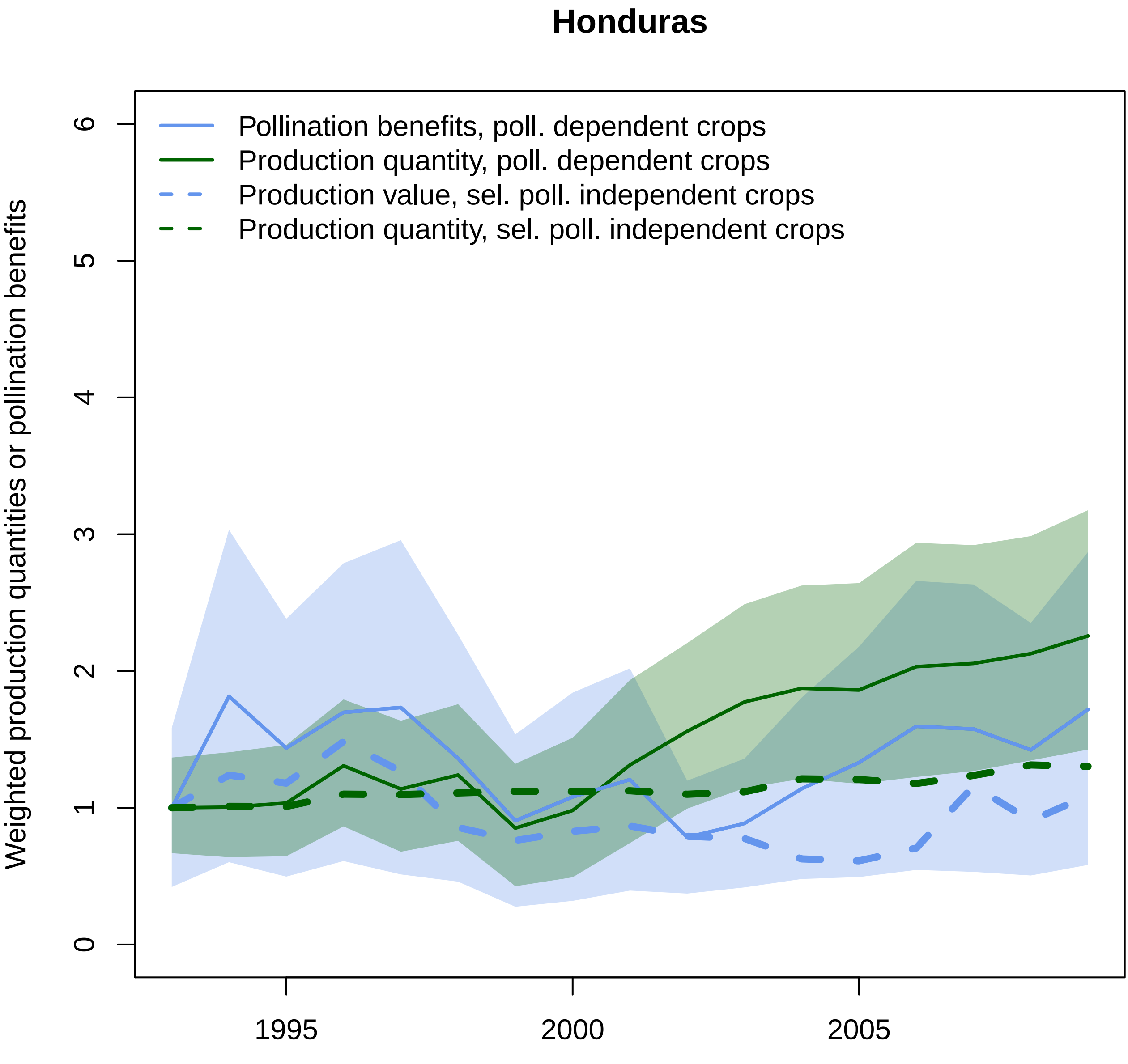
Honduras
Pollination benefits, poll. dependent crops
Production quantity, poll. dependent crops
Production value, sel. poll. independent crops
Production quantity, sel. poll. independent crops
Weighted production quantities or pollination benefits
0
1
2
3
4
5
6
1995
2000
2005

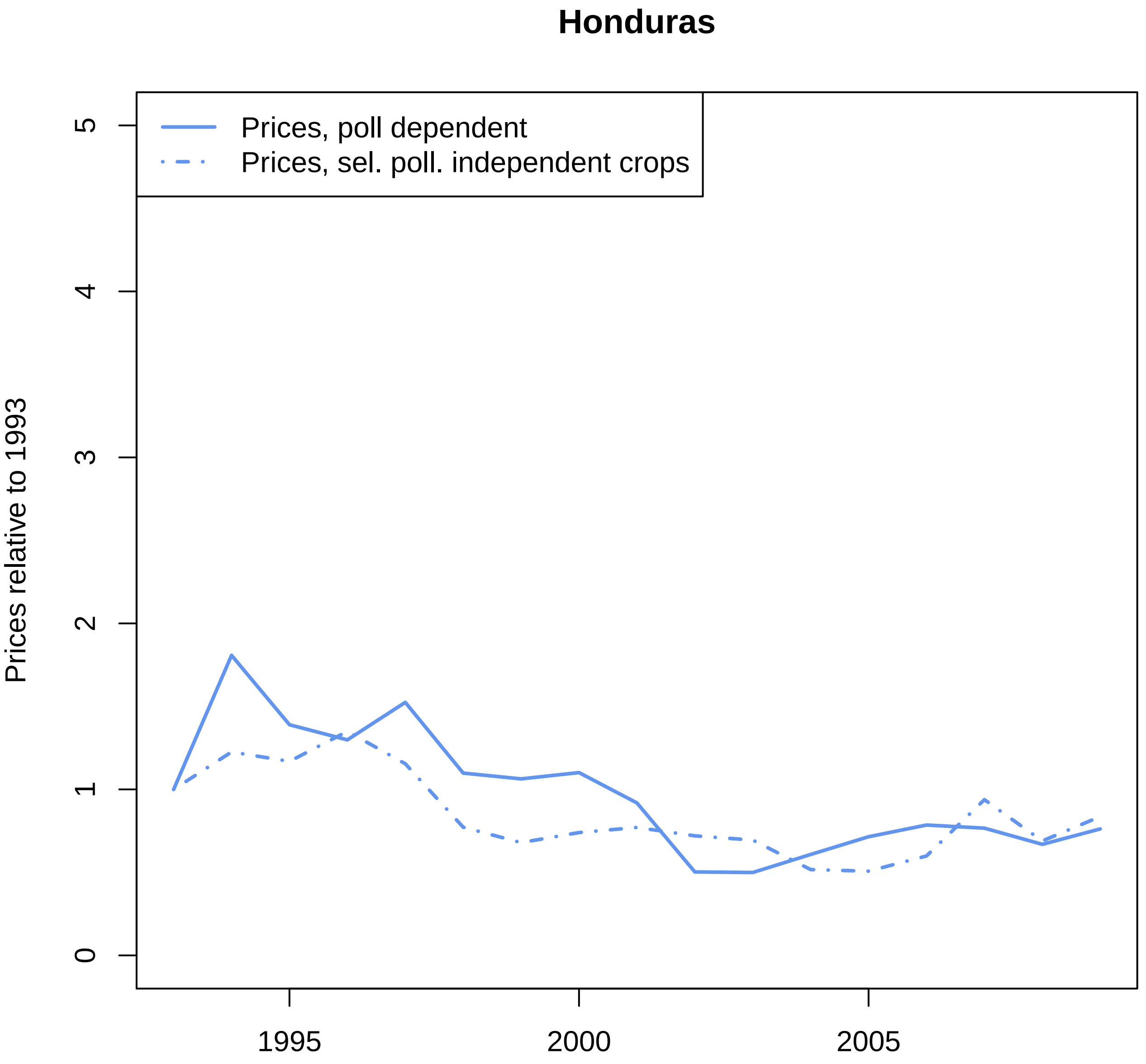
Honduras
Prices, poll dependent
Prices, sel. poll. independent crops
Prices relative to 1993
0
1
2
3
4
5
1995
2000
2005

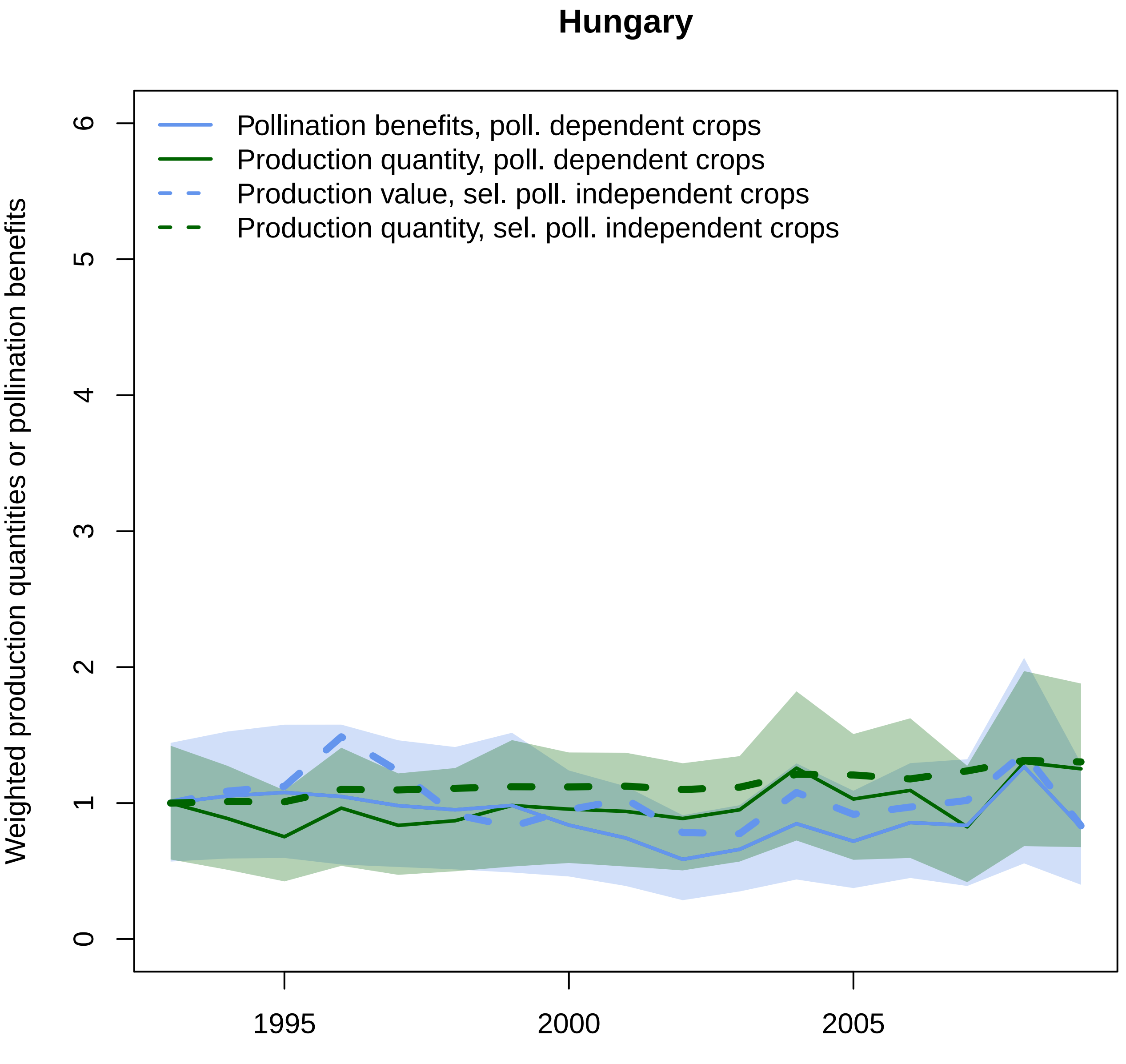
Hungary
Pollination benefits, poll. dependent crops
Production quantity, poll. dependent crops
Production value, sel. poll. independent crops
Production quantity, sel. poll. independent crops
Weighted production quantities or pollination benefits
0
1
2
3
4
5
6
1995
2000
2005

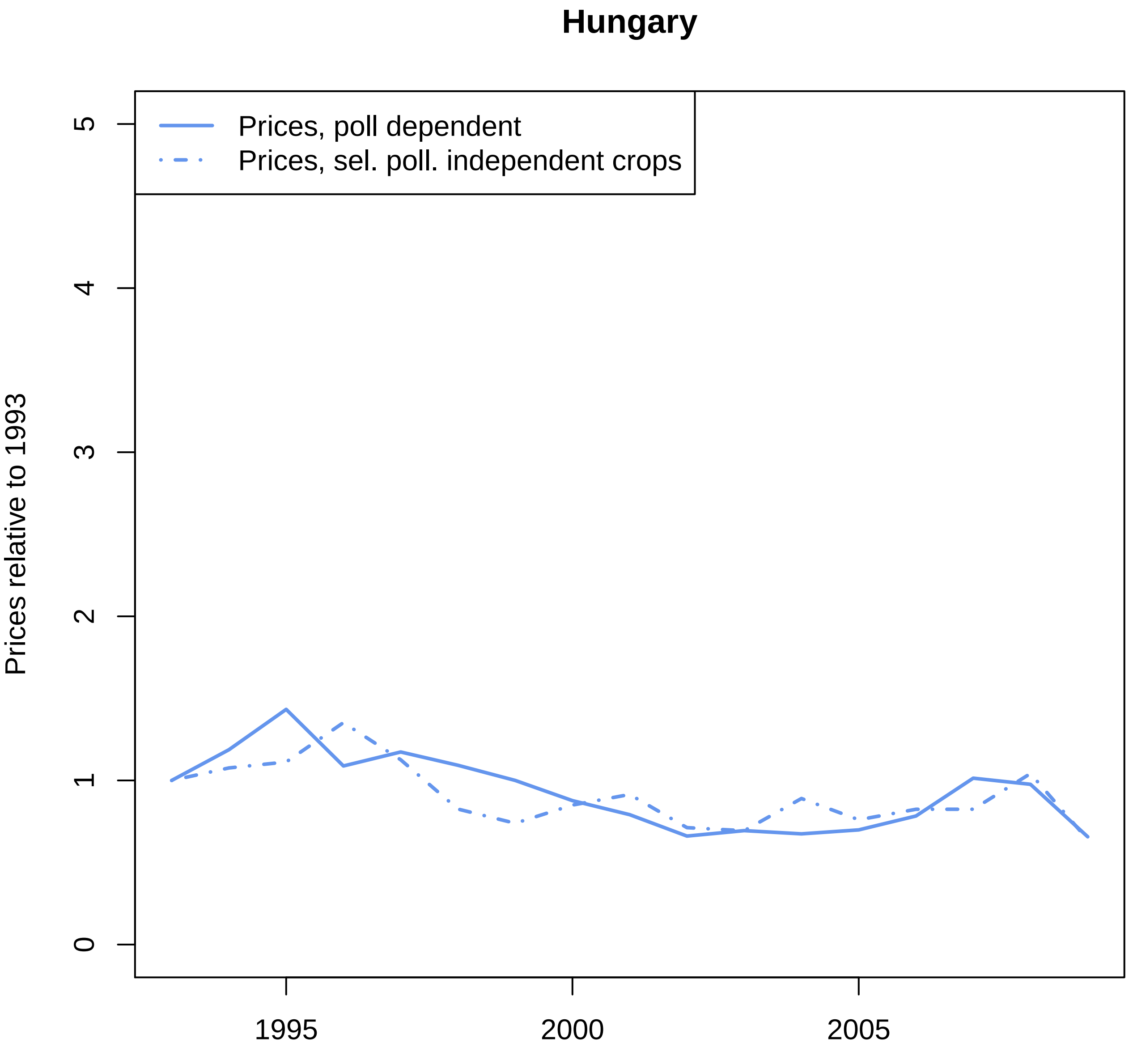
Hungary
Prices, poll dependent
Prices, sel. poll. independent crops
Prices relative to 1993
0
1
2
3
4
5
1995
2000
2005

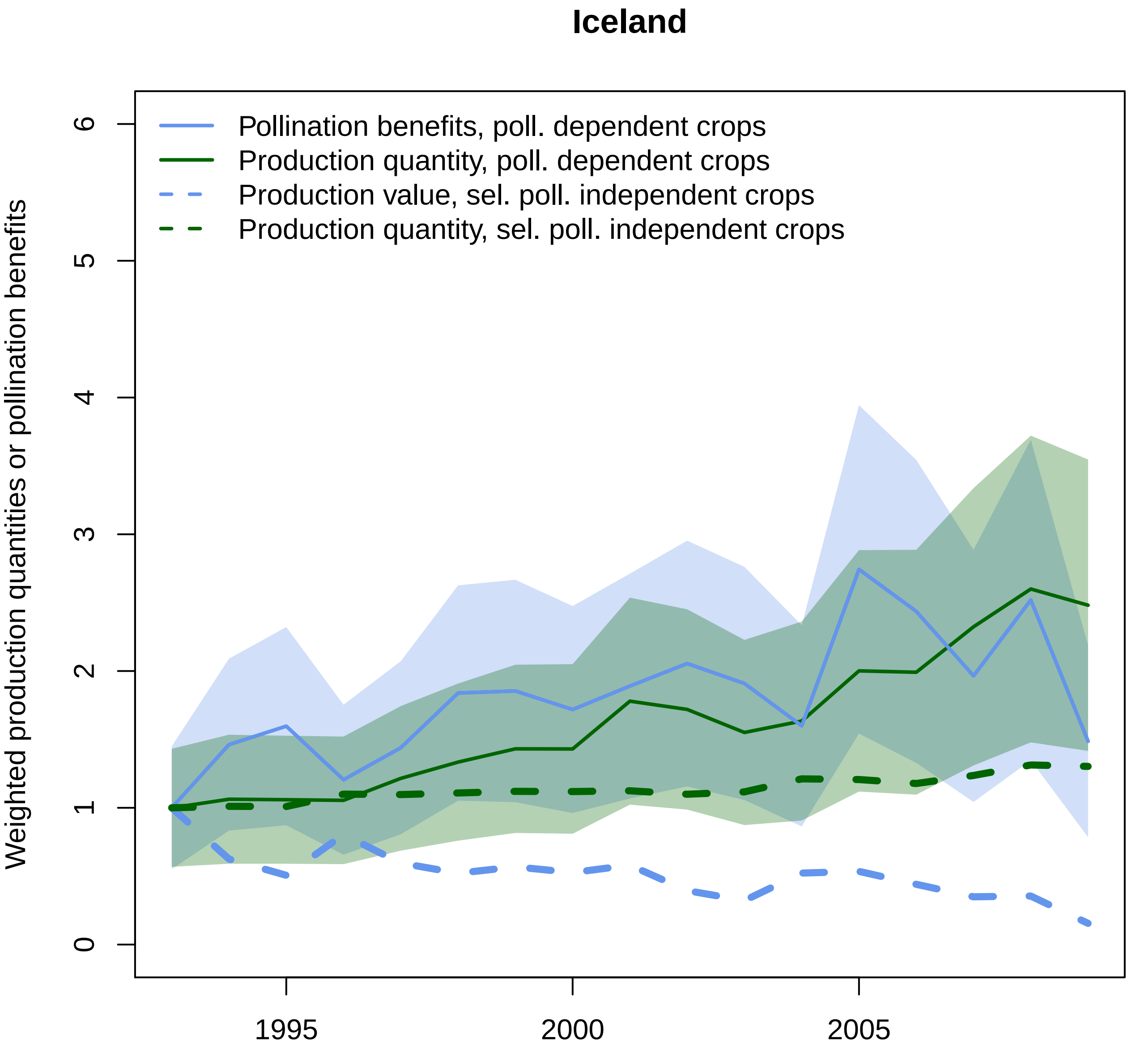

Iceland
Pollination benefits, poll. dependent crops
Production quantity, poll. dependent crops
Production value, sel. poll. independent crops
Production quantity, sel. poll. independent crops
Weighted production quantities or pollination benefits
0
1
2
3
4
5
6
1995
2000
2005

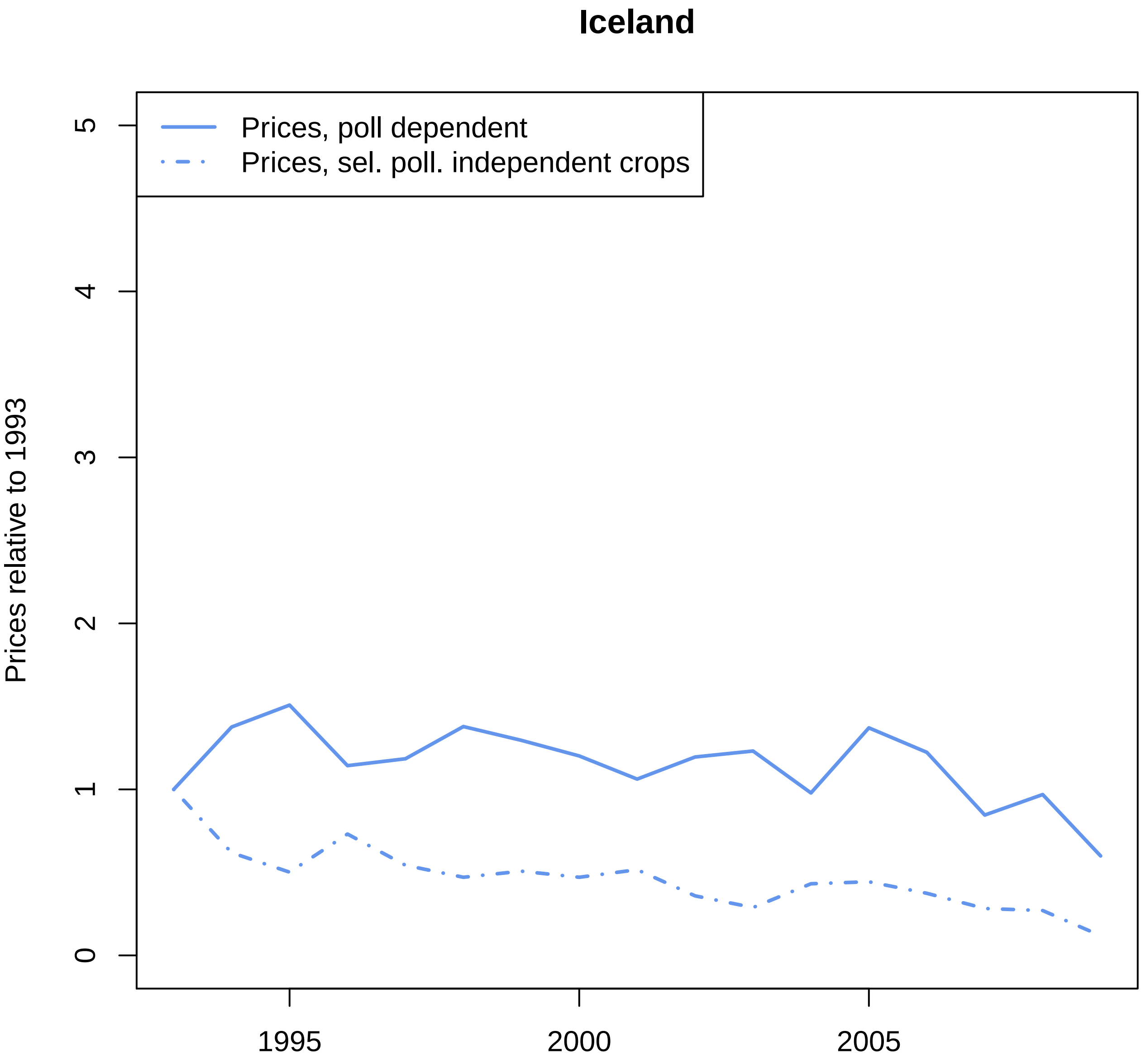
Iceland
Prices, poll dependent
Prices, sel. poll. independent crops
Prices relative to 1993
0
1
2
3
4
5
1995
2000
2005

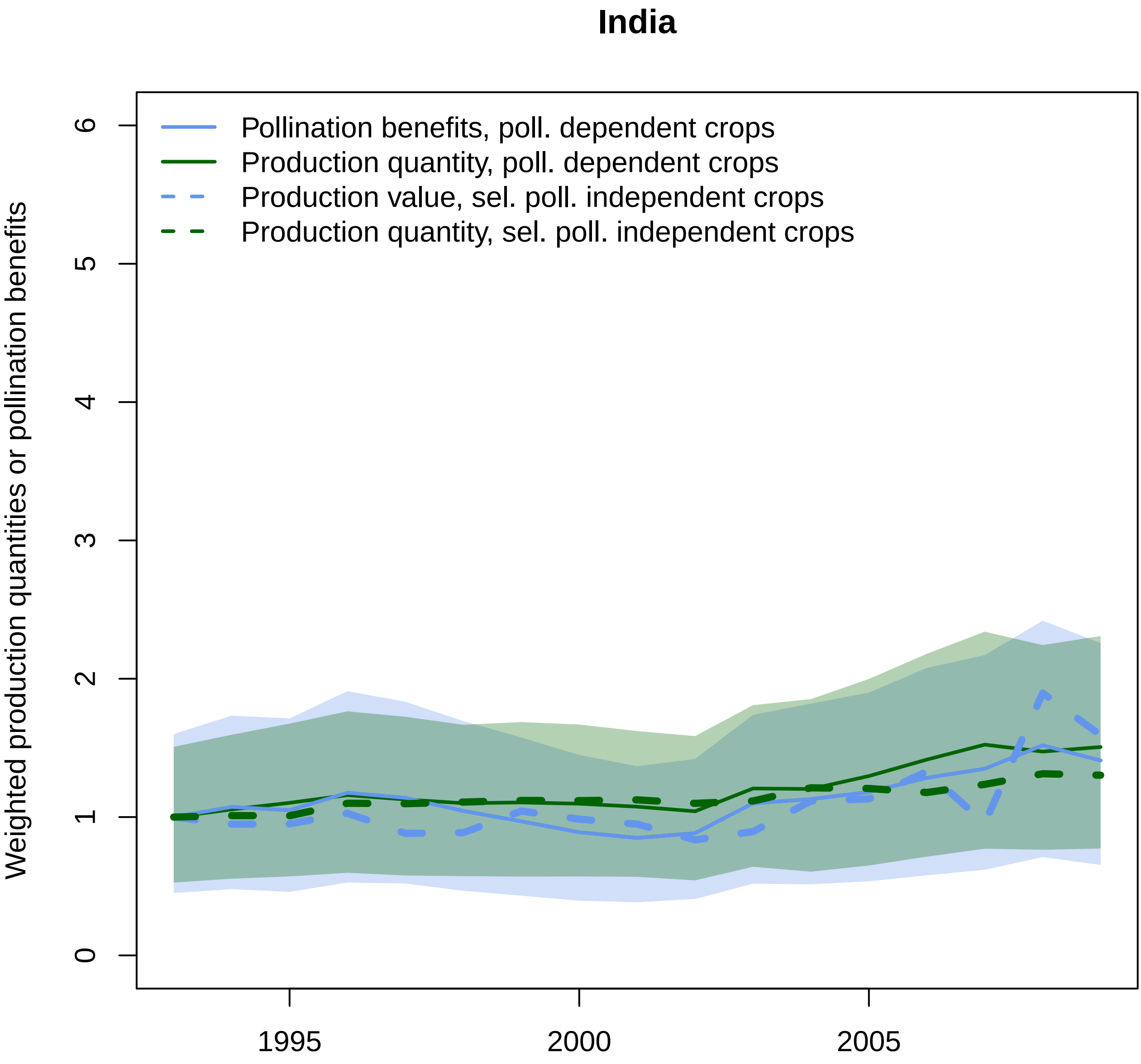
India
Pollination benefits, poll. dependent crops
Production quantity, poll. dependent crops
Production value, sel. poll. independent crops
Production quantity, sel. poll. independent crops
Weighted production quantities or pollination benefits
0
1
2
3
4
5
6
1995
2000
2005

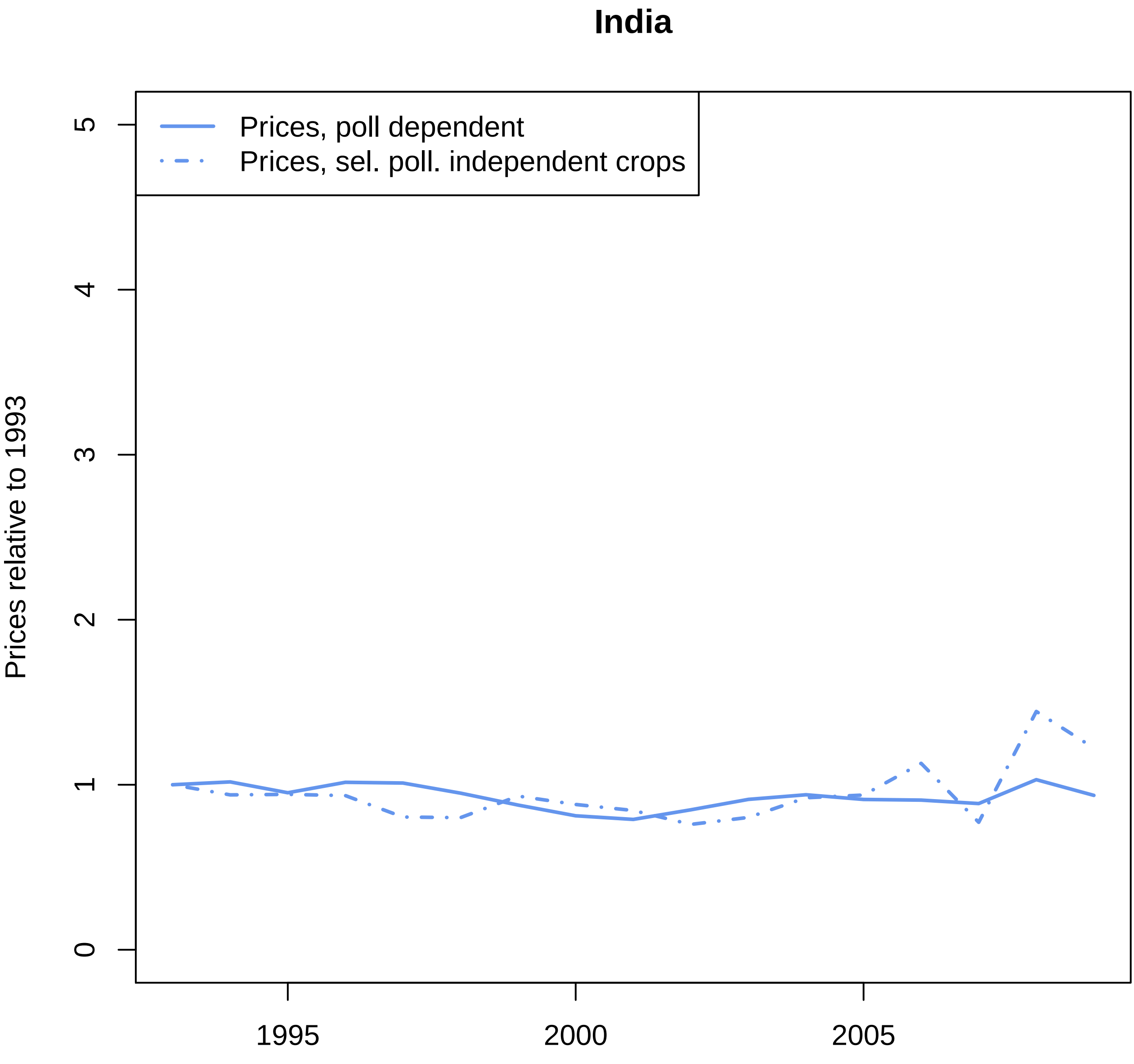
India
Prices, poll dependent
Prices, sel. poll. independent crops
Prices relative to 1993
0
1
2
3
4
5
1995
2000
2005

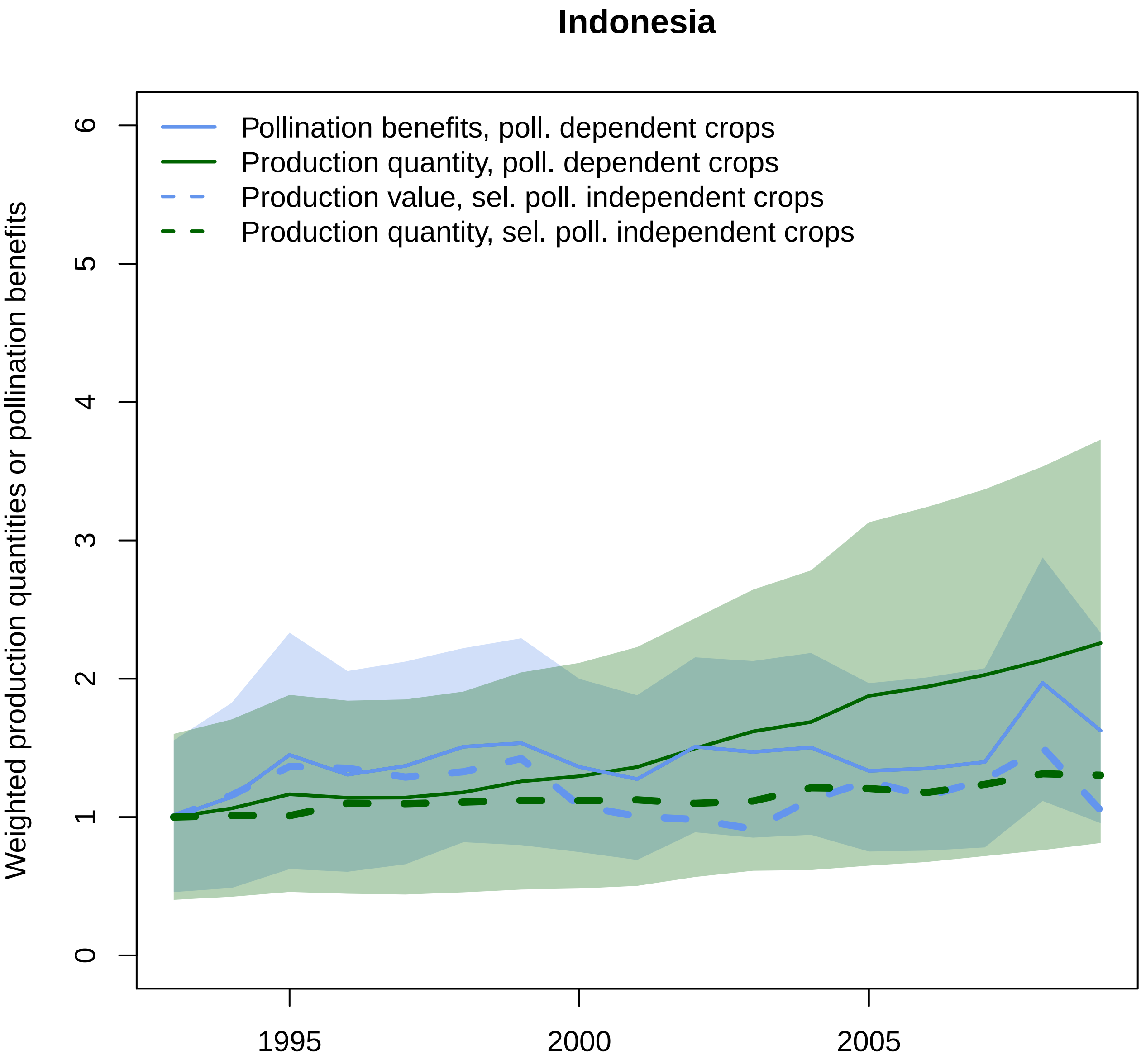
Indonesia
Weighted production quantities or pollination benefits
0
1
2
3
4
5
6
1995
2000
2005
Pollination benefits, poll. dependent crops
Production quantity, poll. dependent crops
Production value, sel. poll. independent crops
Production quantity, sel. poll. independent crops

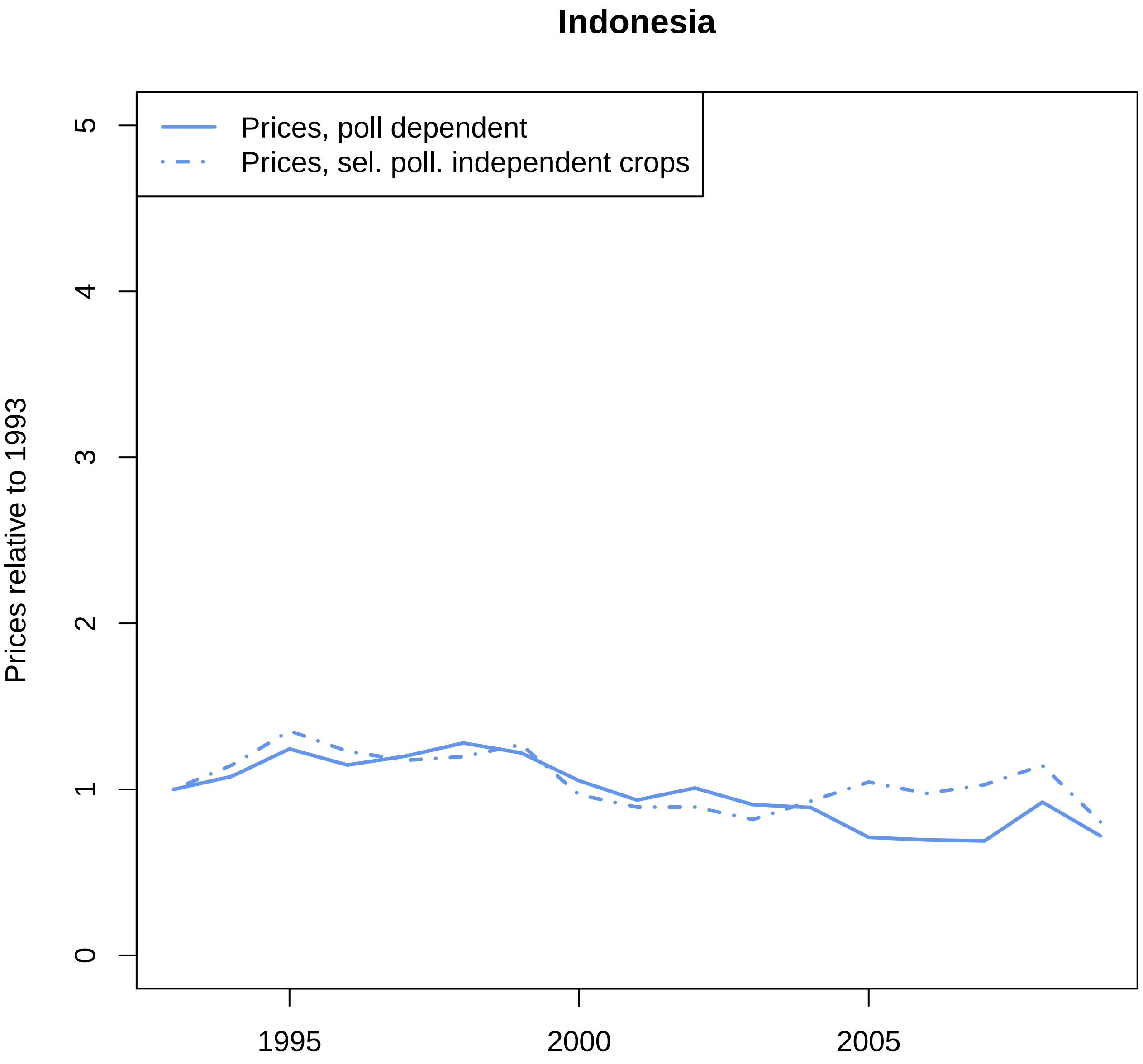

Indonesia
Prices, poll dependent
Prices, sel. poll. independent crops
Prices relative to 1993
0
1
2
3
4
5
1995
2000
2005

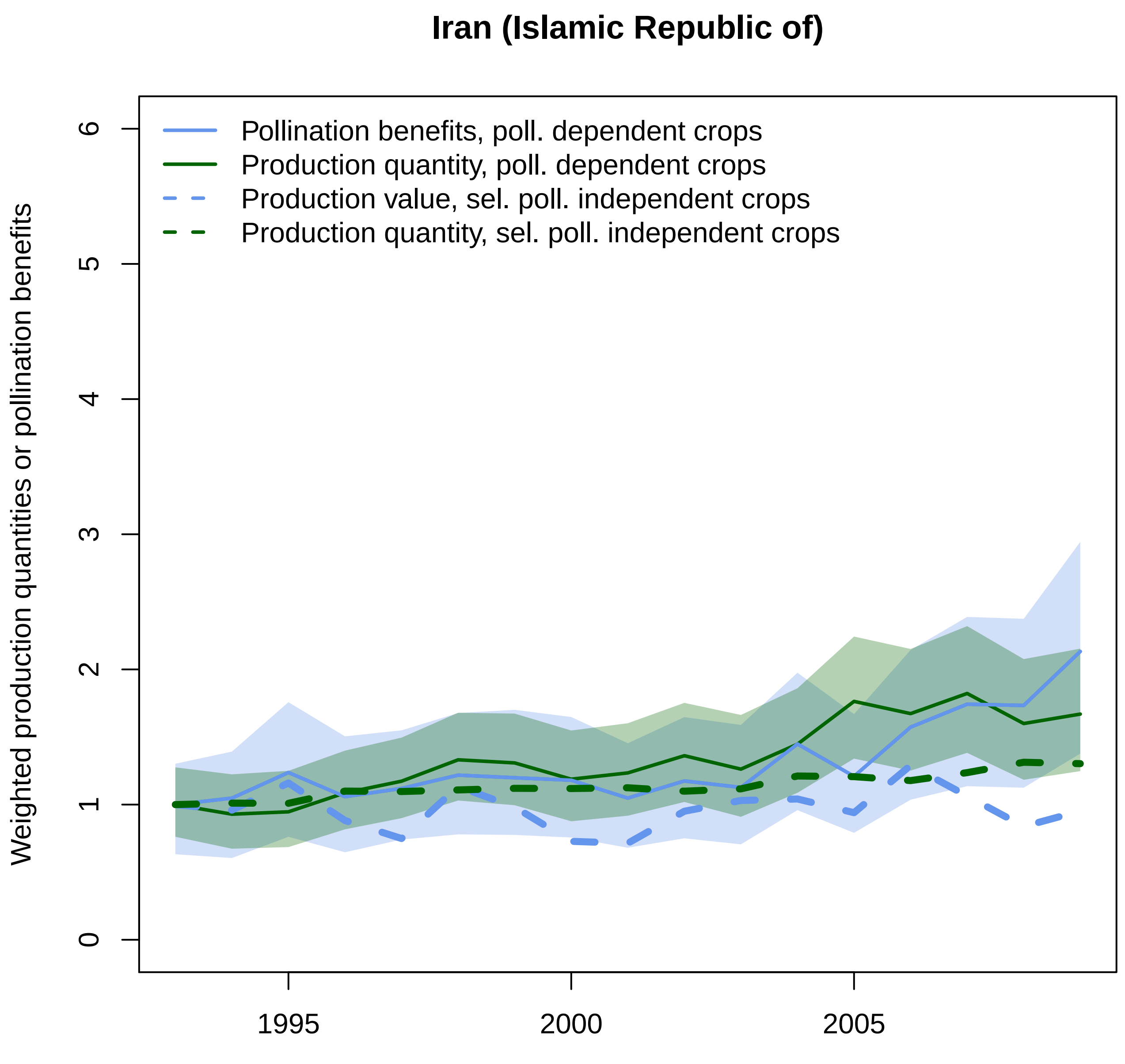

Iran (Islamic Republic of)
Pollination benefits, poll. dependent crops
Production quantity, poll. dependent crops
Production value, sel. poll. independent crops
Production quantity, sel. poll. independent crops
Weighted production quantities or pollination benefits
0
1
2
3
4
5
6
1995
2000
2005

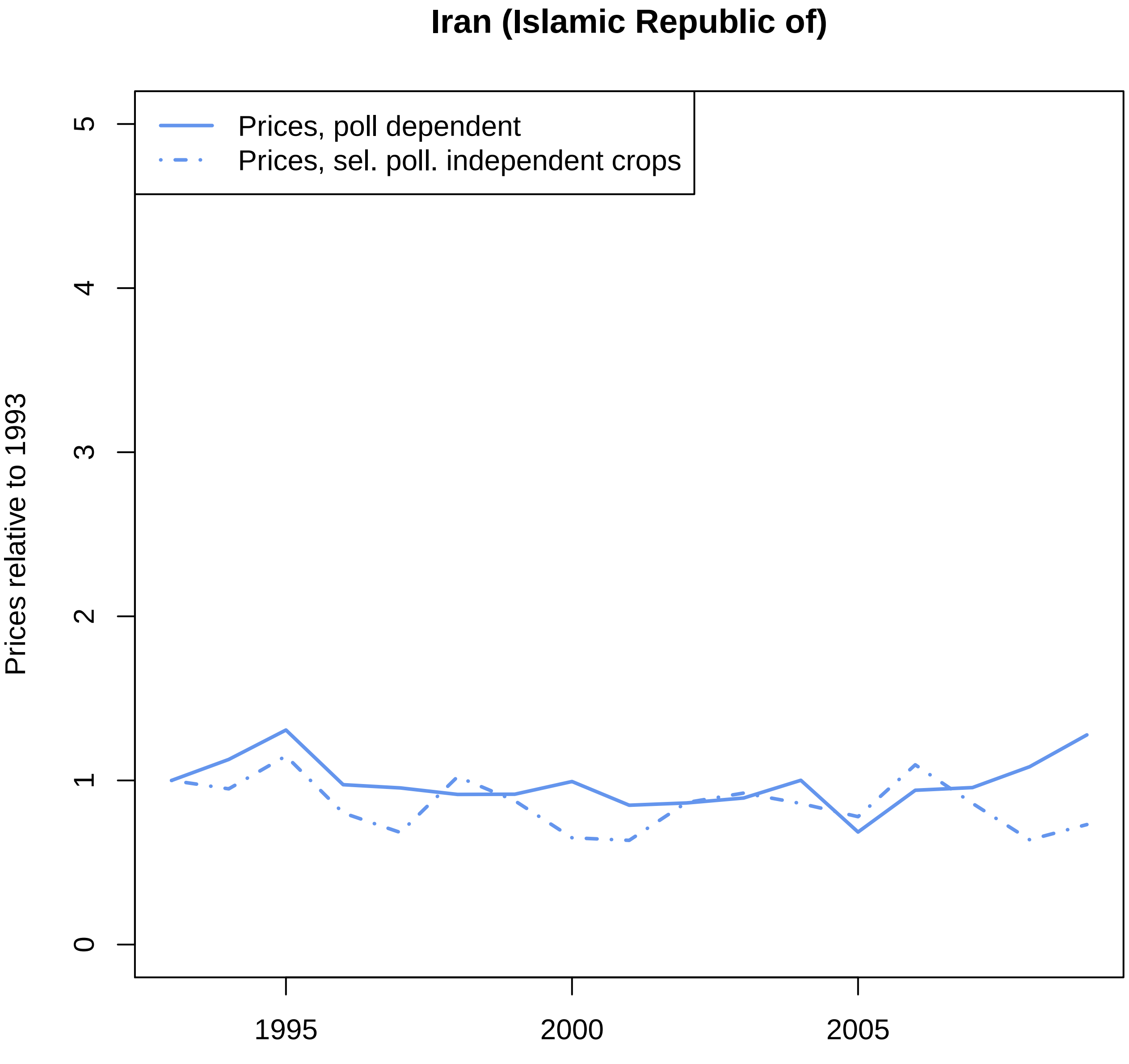
Iran (Islamic Republic of)
Prices, poll dependent
Prices, sel. poll. independent crops
Prices relative to 1993
0
1
2
3
4
5
1995
2000
2005

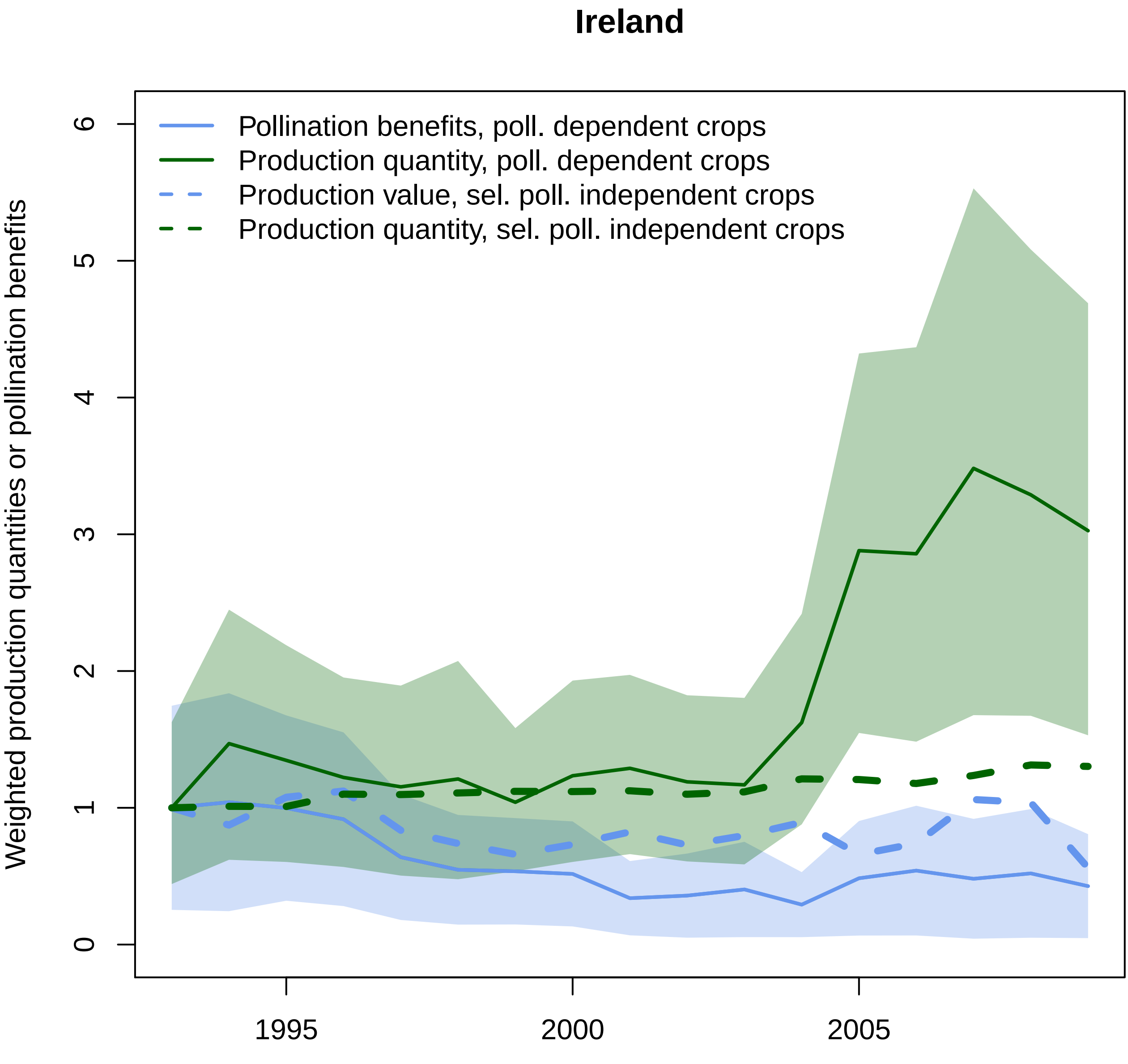
Ireland
Pollination benefits, poll. dependent crops
Production quantity, poll. dependent crops
Production value, sel. poll. independent crops
Production quantity, sel. poll. independent crops
Weighted production quantities or pollination benefits
0
1
2
3
4
5
6
1995
2000
2005

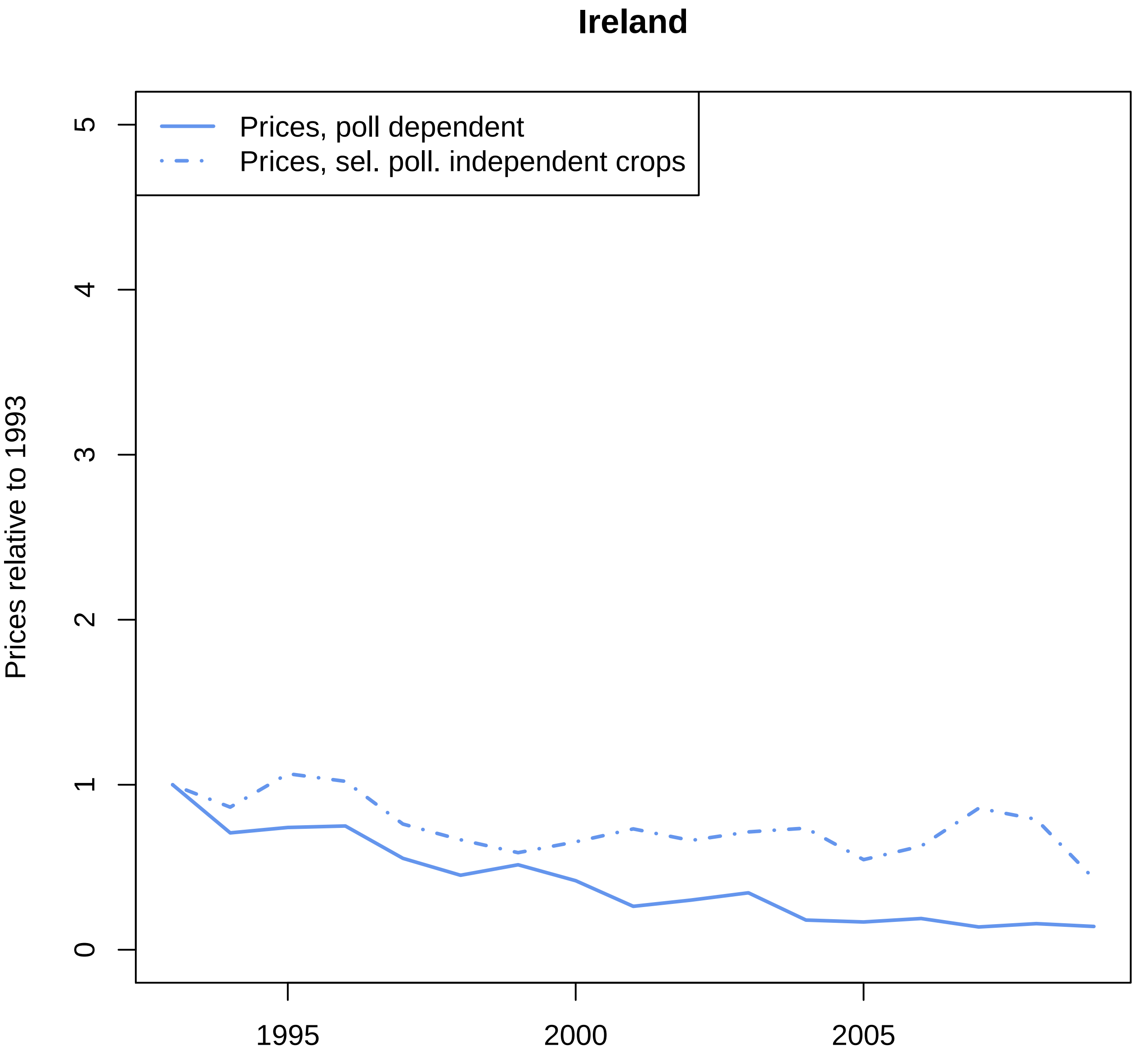
Ireland
Prices, poll dependent
Prices, sel. poll. independent crops
Prices relative to 1993
0
1
2
3
4
5
1995
2000
2005

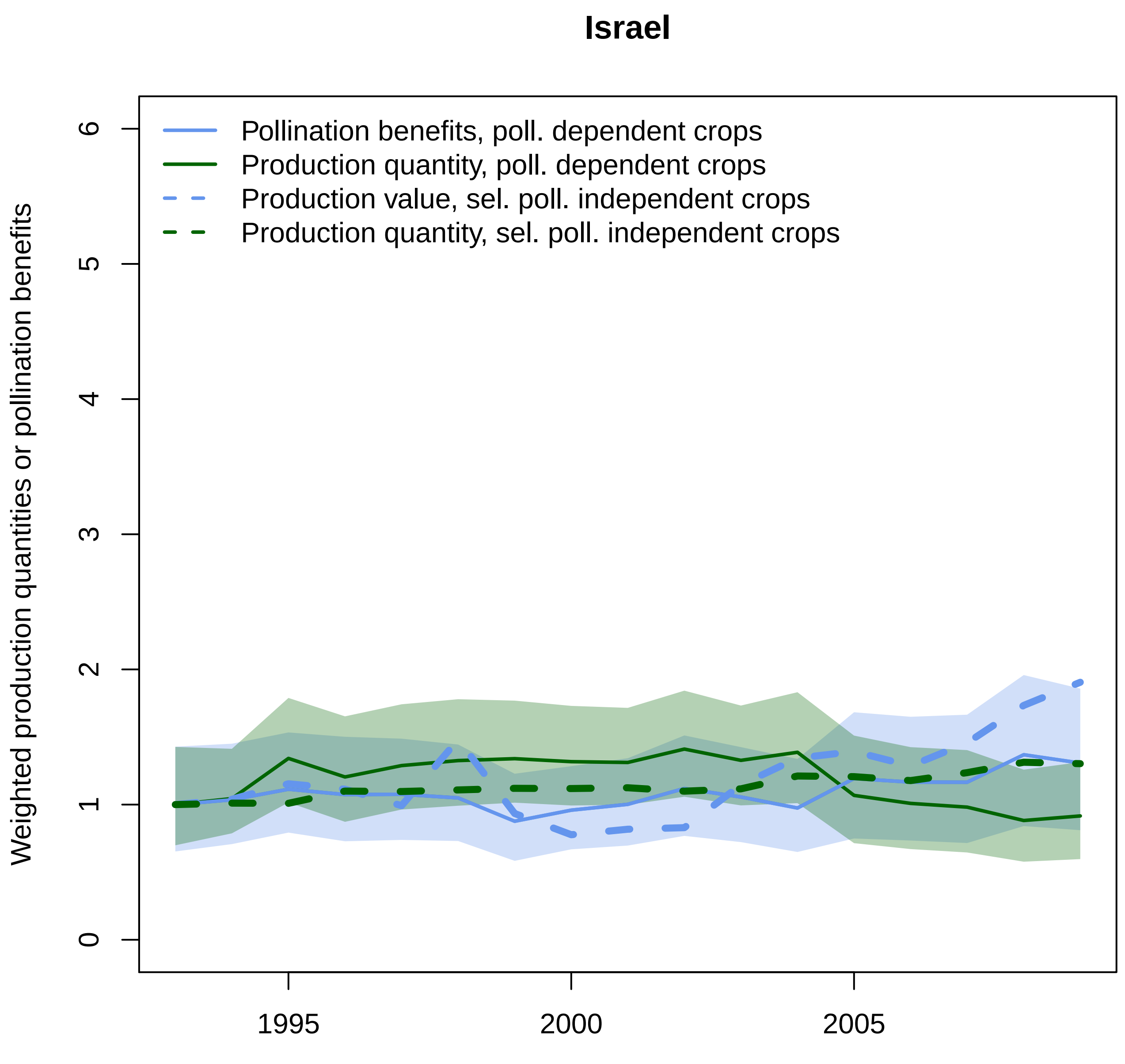
Israel
Pollination benefits, poll. dependent crops
Production quantity, poll. dependent crops
Production value, sel. poll. independent crops
Production quantity, sel. poll. independent crops
Weighted production quantities or pollination benefits
0
1
2
3
4
5
6
1995
2000
2005

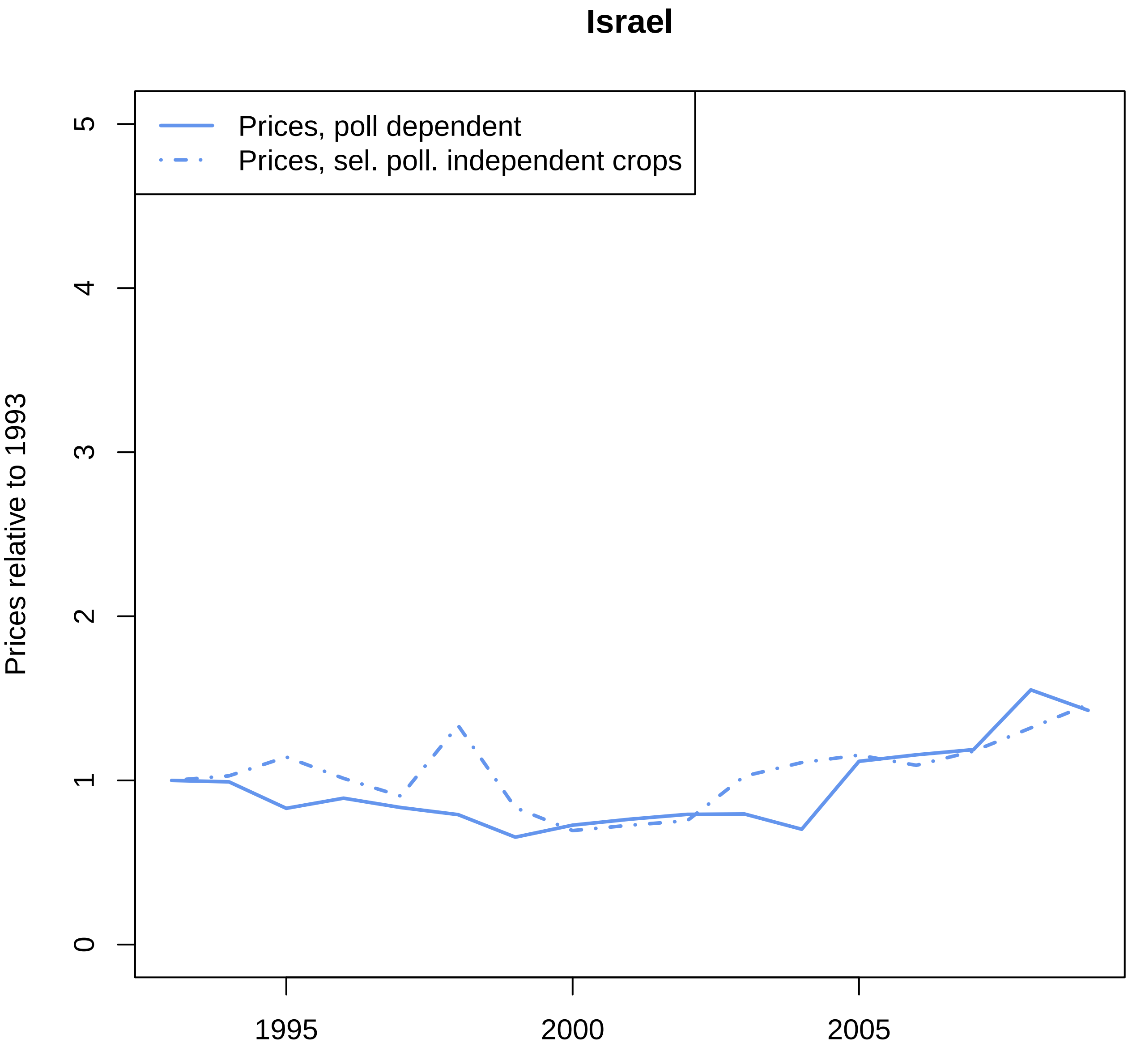
Israel
Prices, poll dependent
Prices, sel. poll. independent crops
Prices relative to 1993
0
1
2
3
4
5
1995
2000
2005

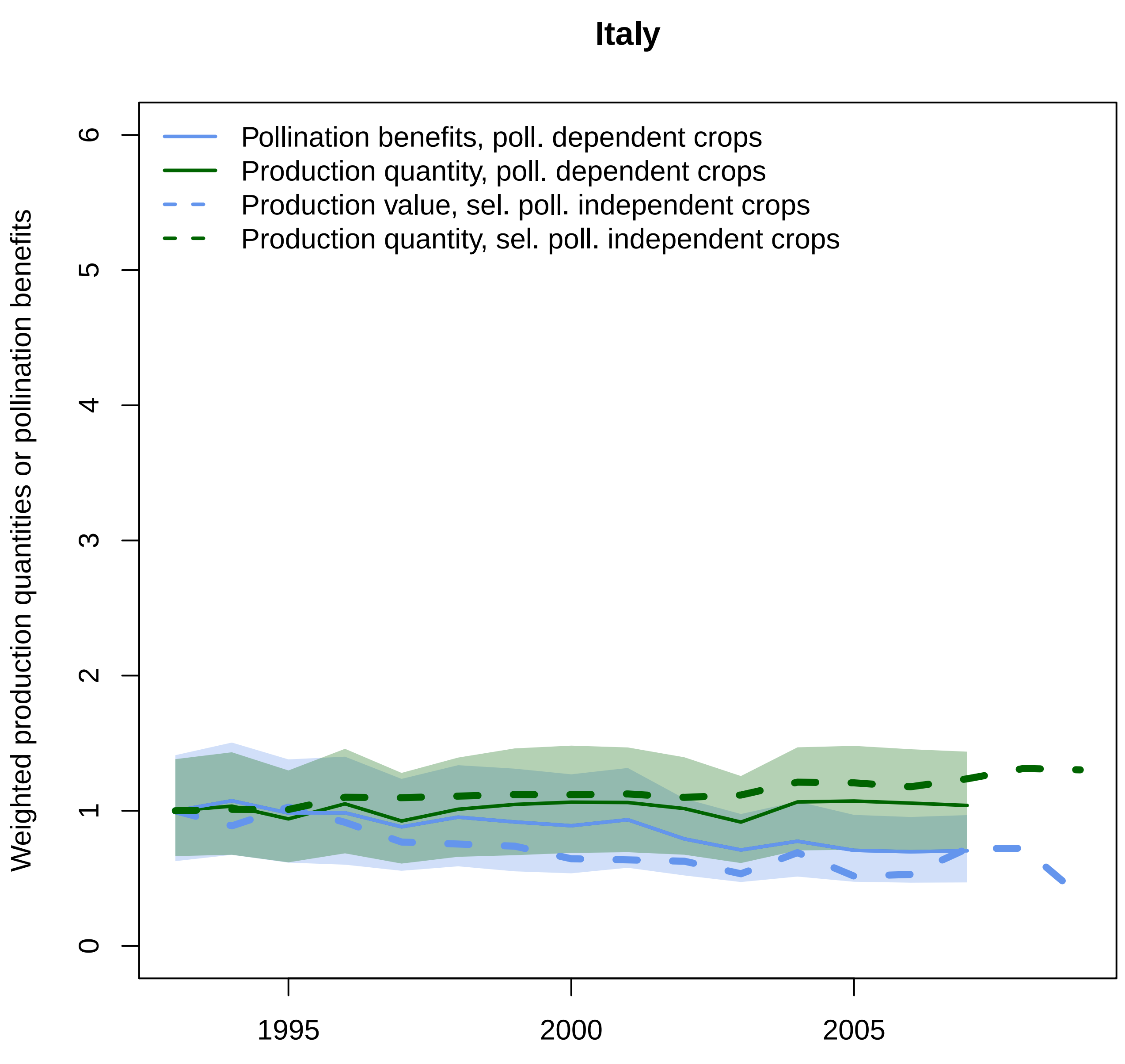

Italy
Pollination benefits, poll. dependent crops
Production quantity, poll. dependent crops
Production value, sel. poll. independent crops
Production quantity, sel. poll. independent crops
Weighted production quantities or pollination benefits
0
1
2
3
4
5
6
1995
2000
2005

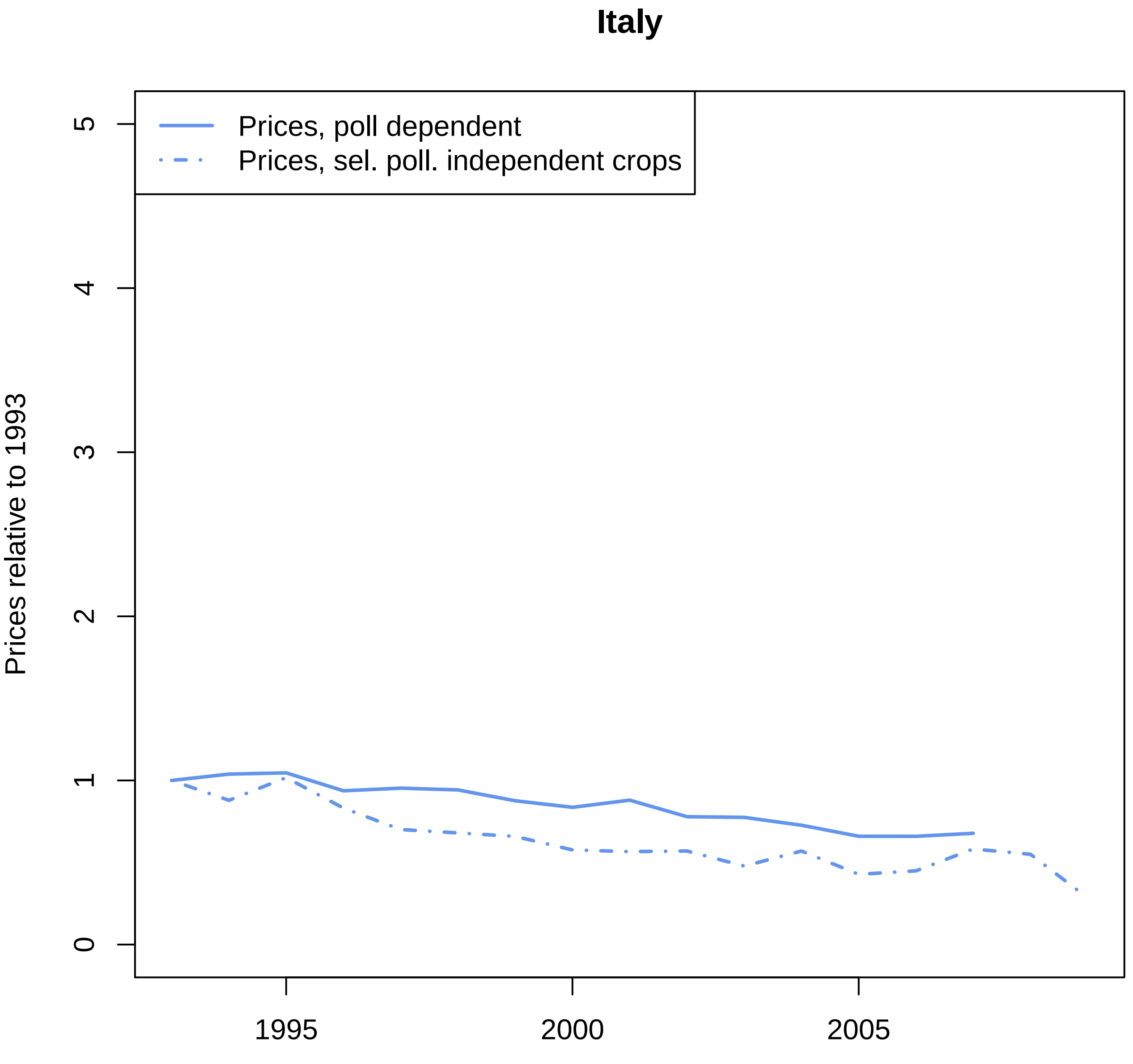

Italy
Prices, poll dependent
Prices, sel. poll. independent crops
Prices relative to 1993
0
1
2
3
4
5
1995
2000
2005

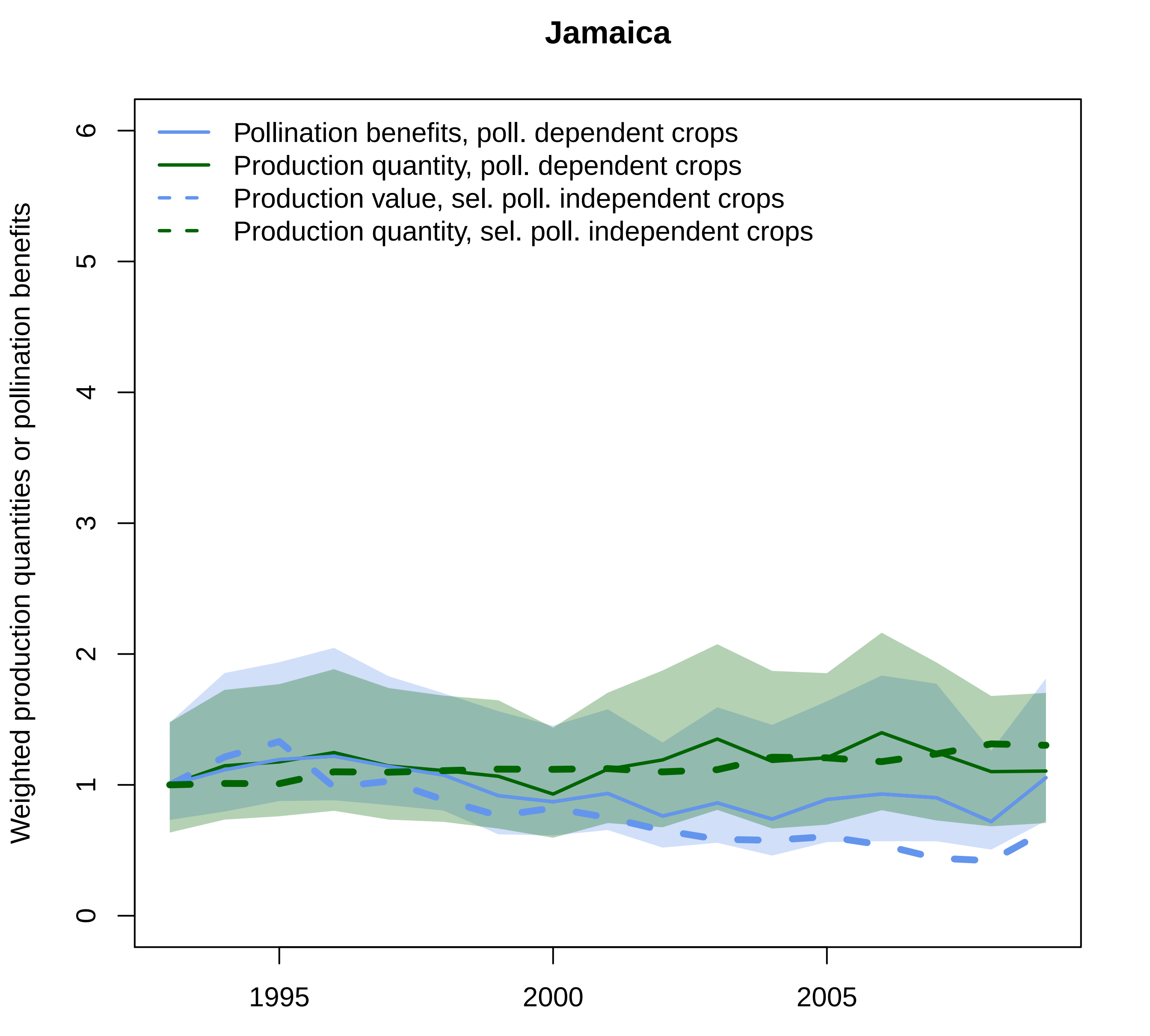
Jamaica
Pollination benefits, poll. dependent crops
Production quantity, poll. dependent crops
Production value, sel. poll. independent crops
Production quantity, sel. poll. independent crops
Weighted production quantities or pollination benefits
0
1
2
3
4
5
6
1995
2000
2005

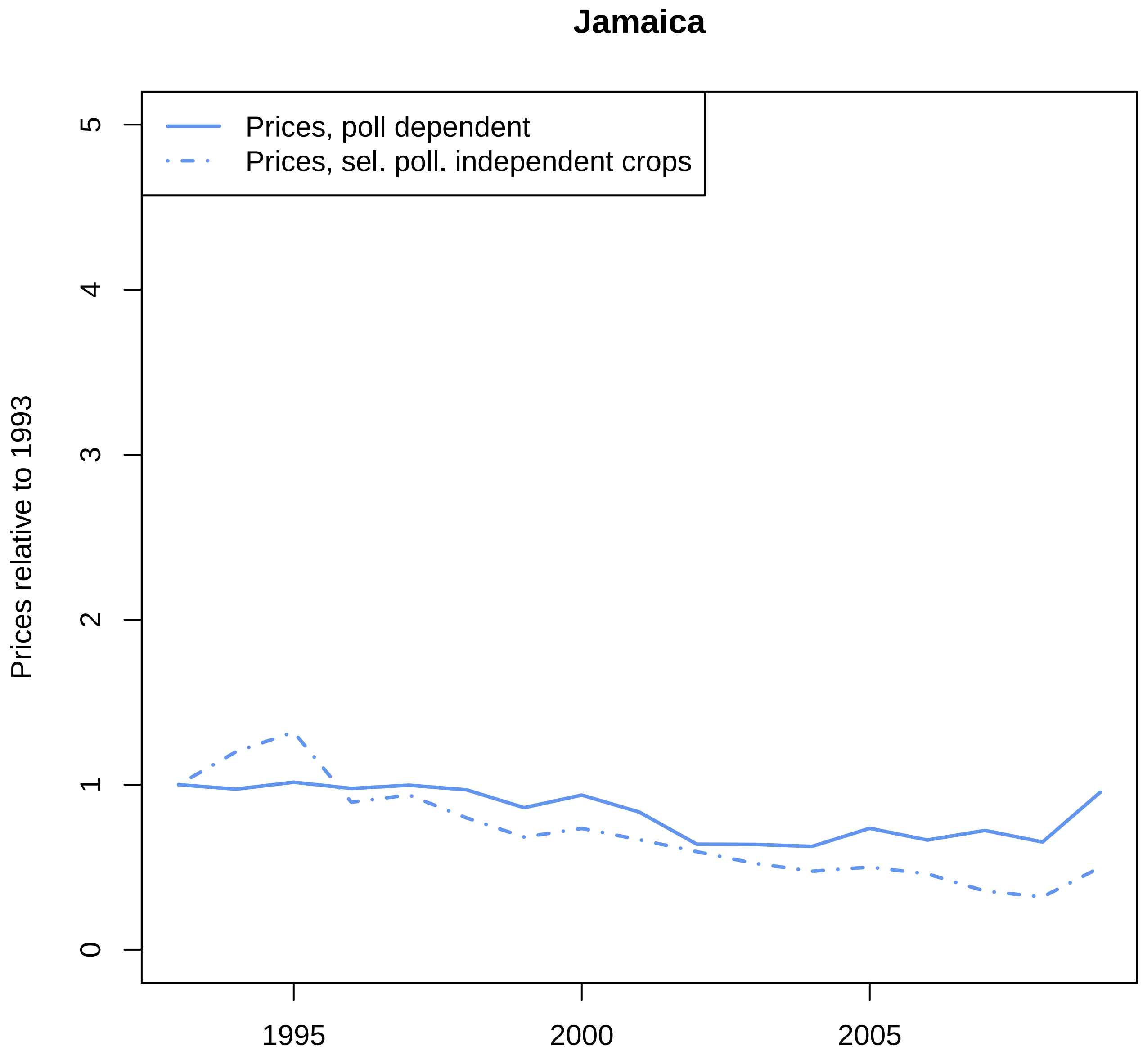

Jamaica
Prices, poll dependent
Prices, sel. poll. independent crops
Prices relative to 1993
0
1
2
3
4
5
1995
2000
2005

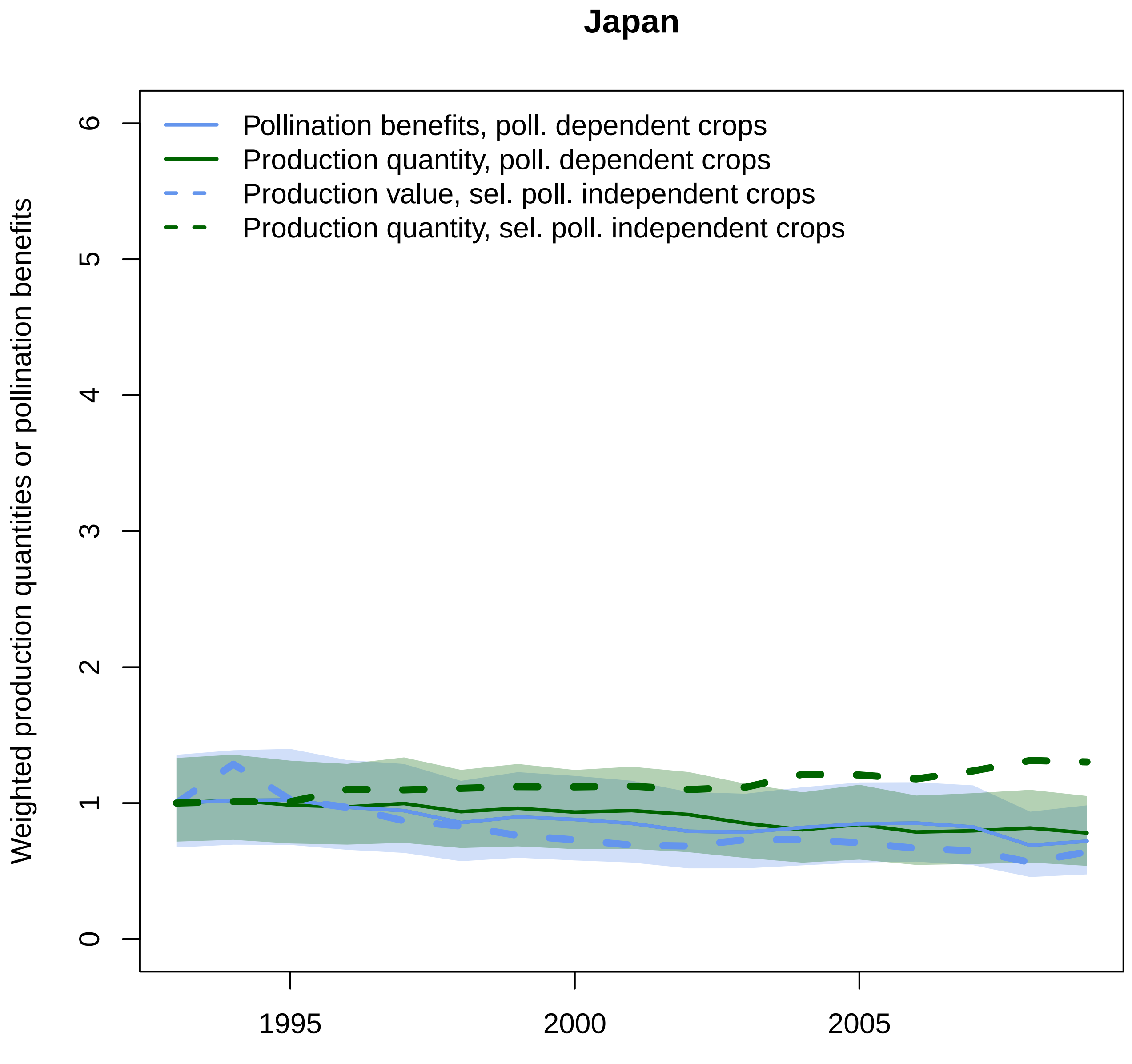
Japan
Pollination benefits, poll. dependent crops
Production quantity, poll. dependent crops
Production value, sel. poll. independent crops
Production quantity, sel. poll. independent crops
Weighted production quantities or pollination benefits
0
1
2
3
4
5
6
1995
2000
2005

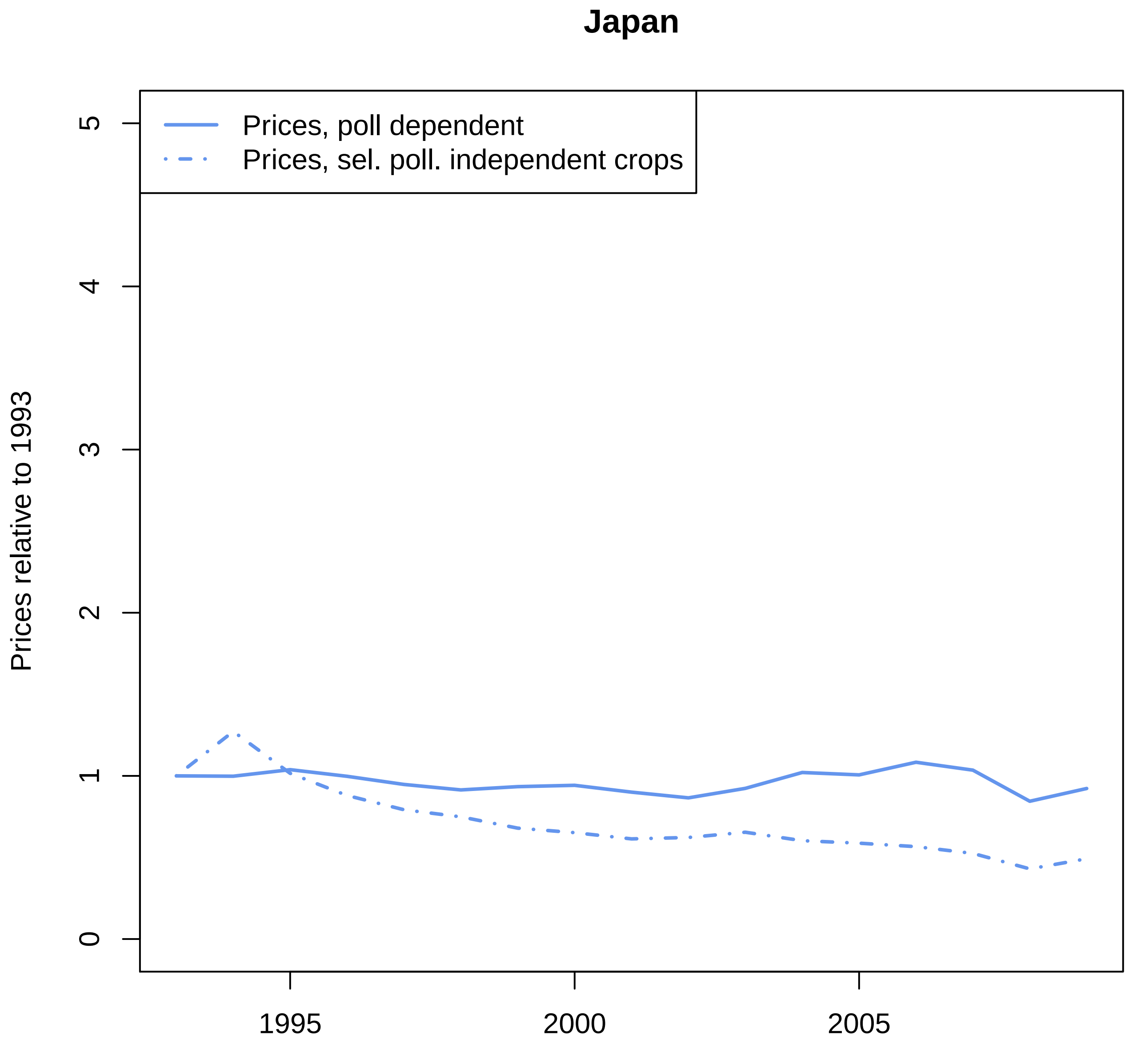
Japan
Prices, poll dependent
Prices, sel. poll. independent crops
Prices relative to 1993
0
1
2
3
4
5
1995
2000
2005

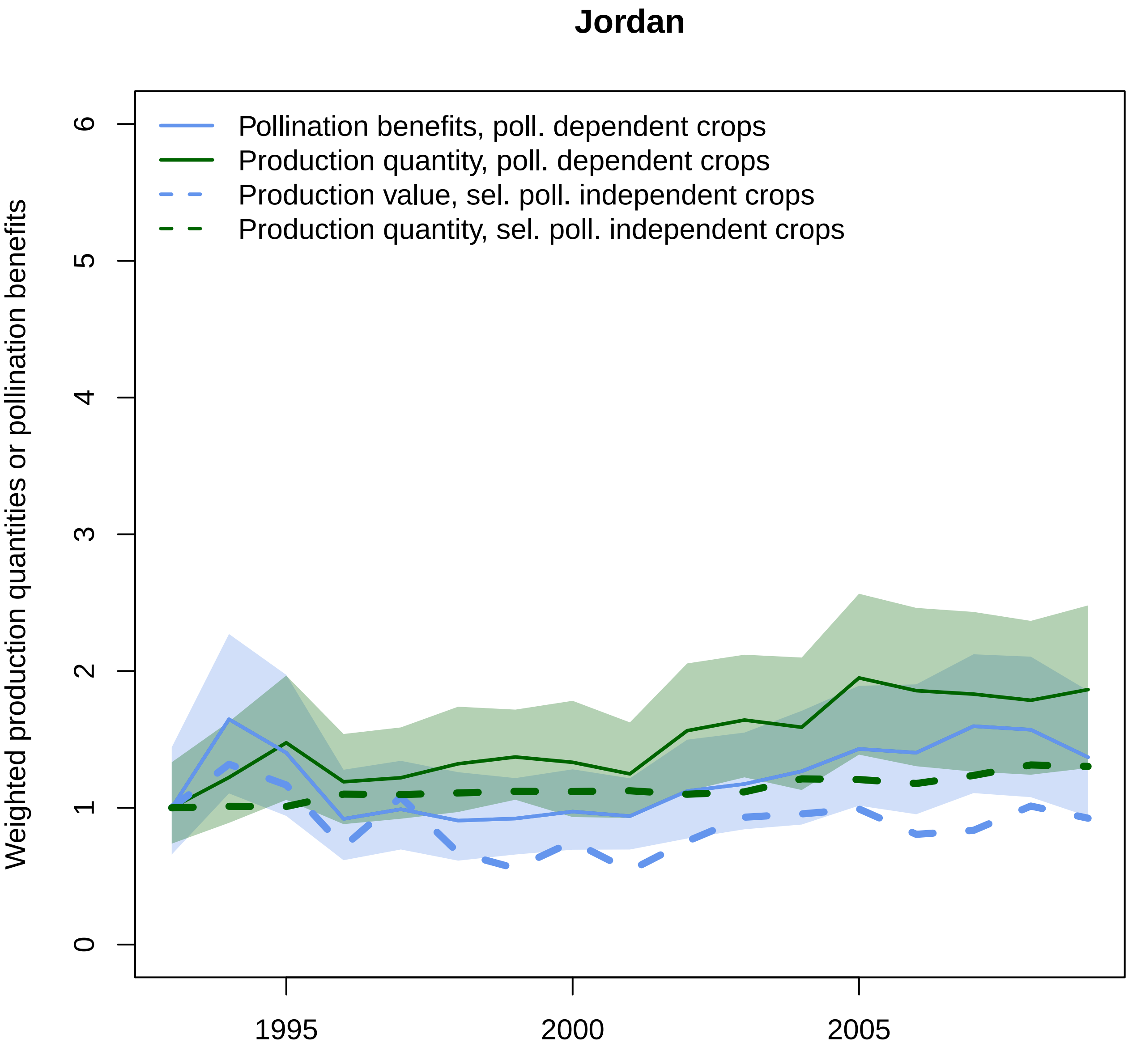

Jordan
Pollination benefits, poll. dependent crops
Production quantity, poll. dependent crops
Production value, sel. poll. independent crops
Production quantity, sel. poll. independent crops
Weighted production quantities or pollination benefits
0
1
2
3
4
5
6
1995
2000
2005

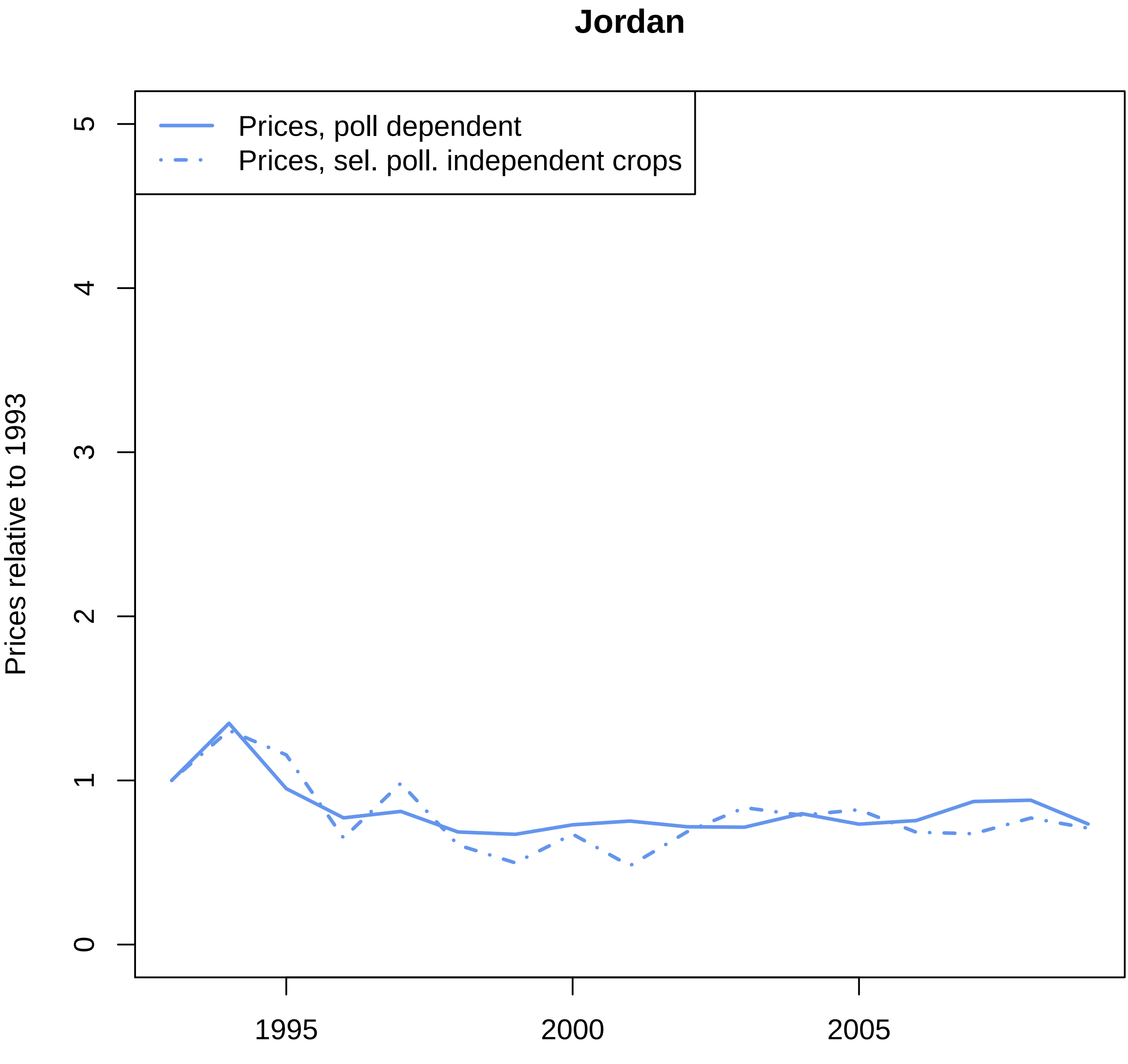

Jordan
Prices, poll dependent
Prices, sel. poll. independent crops
Prices relative to 1993
0
1
2
3
4
5
1995
2000
2005

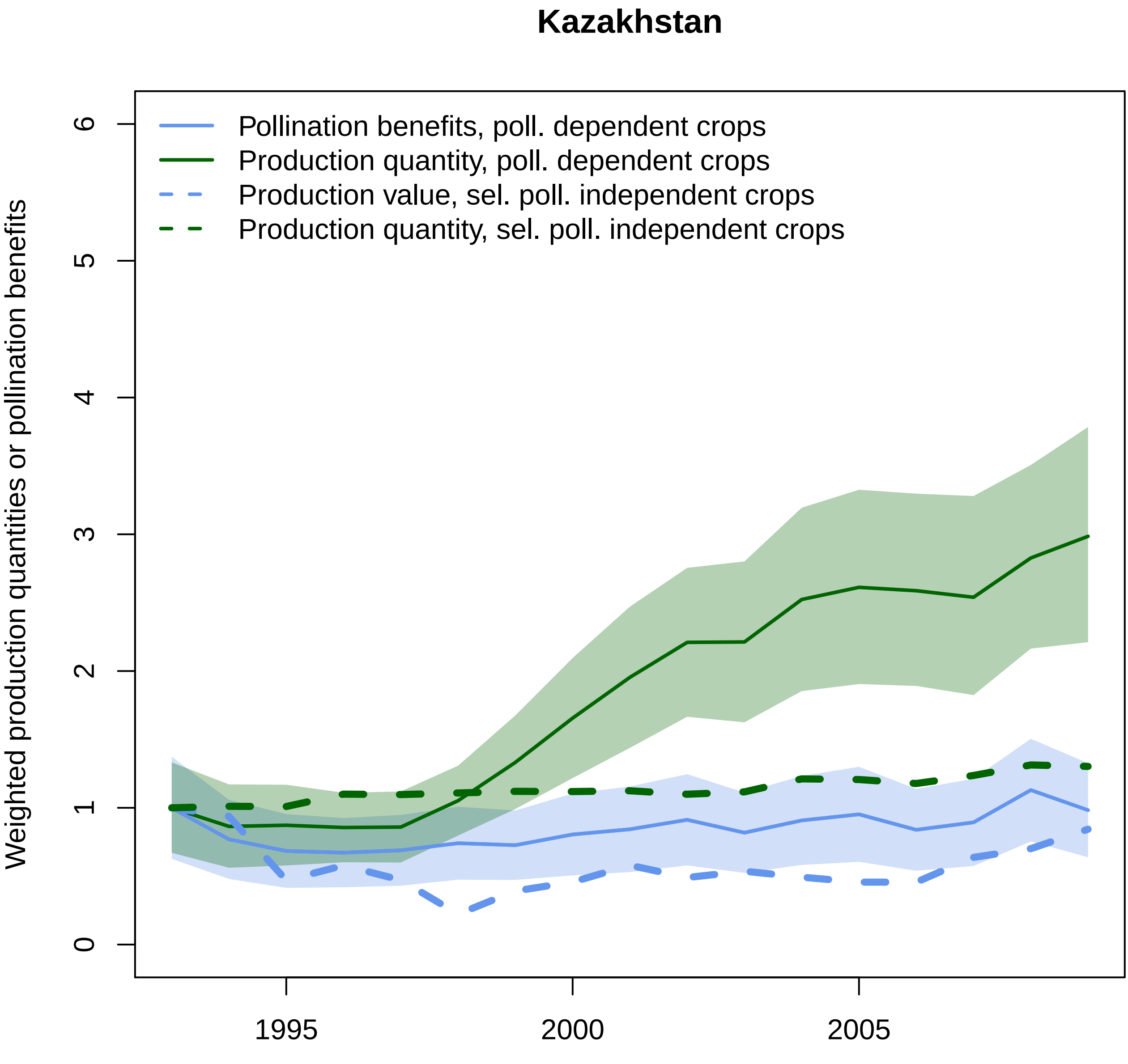

Kazakhstan
Pollination benefits, poll. dependent crops
Production quantity, poll. dependent crops
Production value, sel. poll. independent crops
Production quantity, sel. poll. independent crops
Weighted production quantities or pollination benefits
0
1
2
3
4
5
6
1995
2000
2005

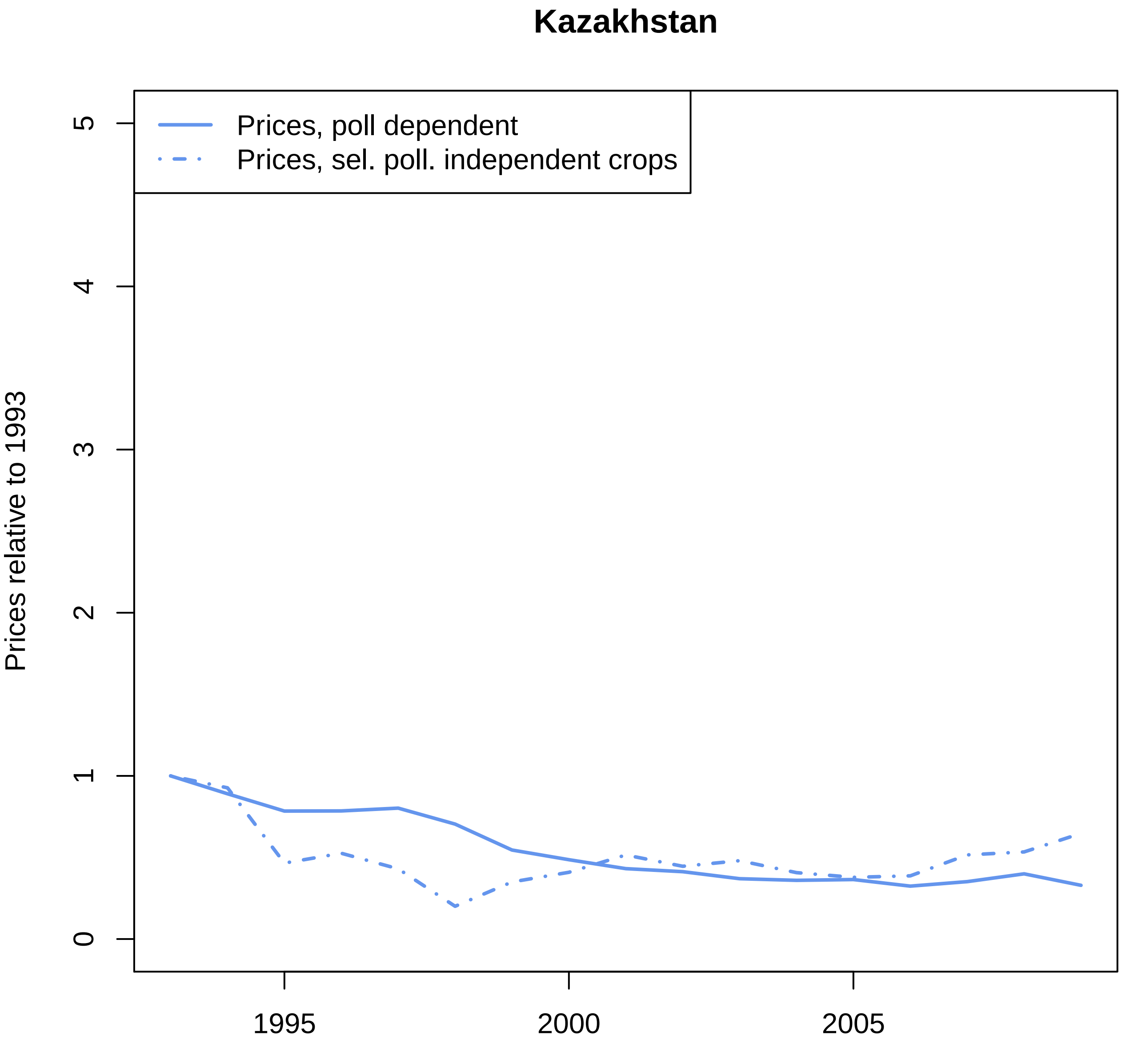
Kazakhstan
Prices, poll dependent
Prices, sel. poll. independent crops
Prices relative to 1993
0
1
2
3
4
5
1995
2000
2005

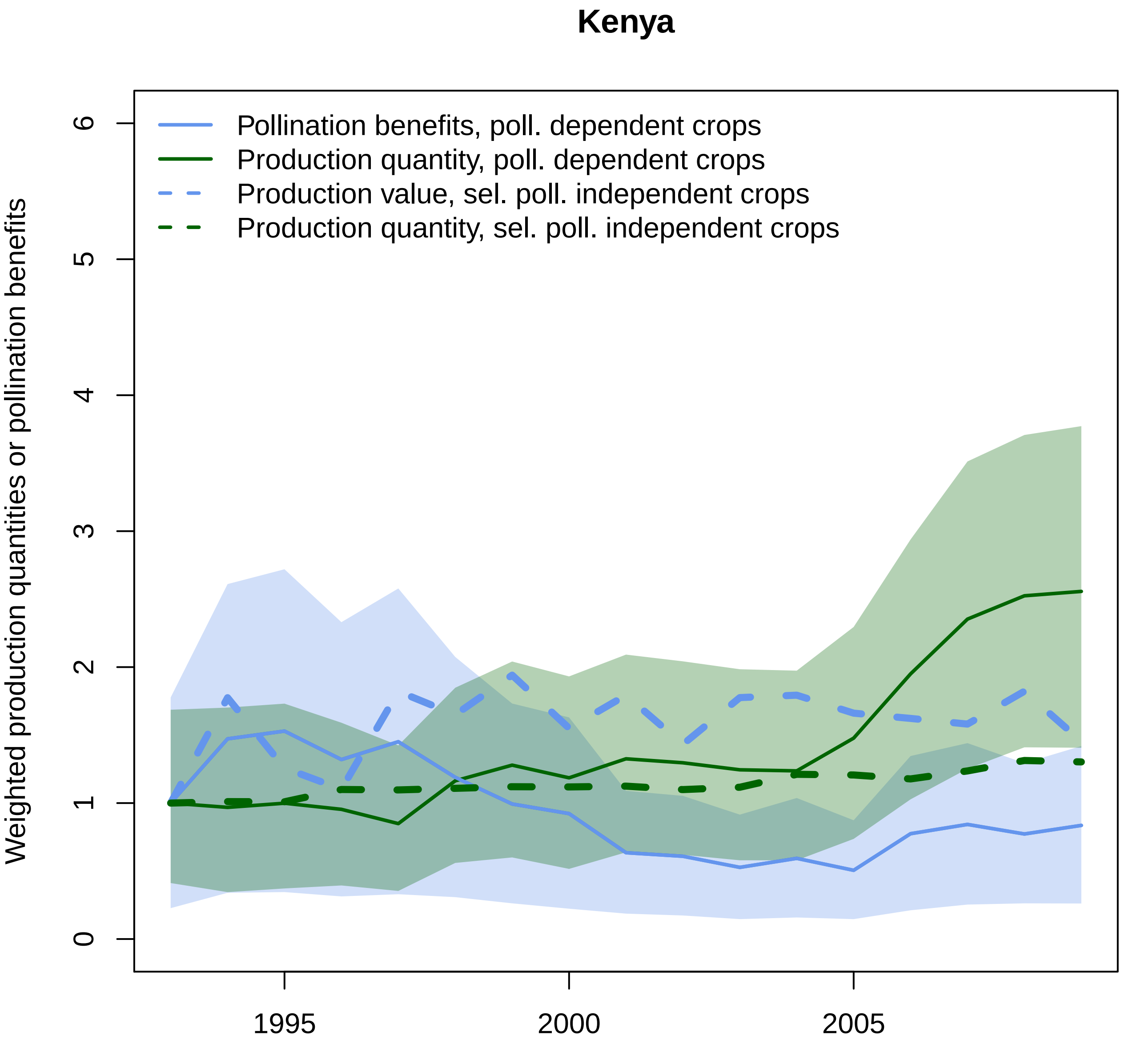
Kenya
Pollination benefits, poll. dependent crops
Production quantity, poll. dependent crops
Production value, sel. poll. independent crops
Production quantity, sel. poll. independent crops
Weighted production quantities or pollination benefits
0
1
2
3
4
5
6
1995
2000
2005

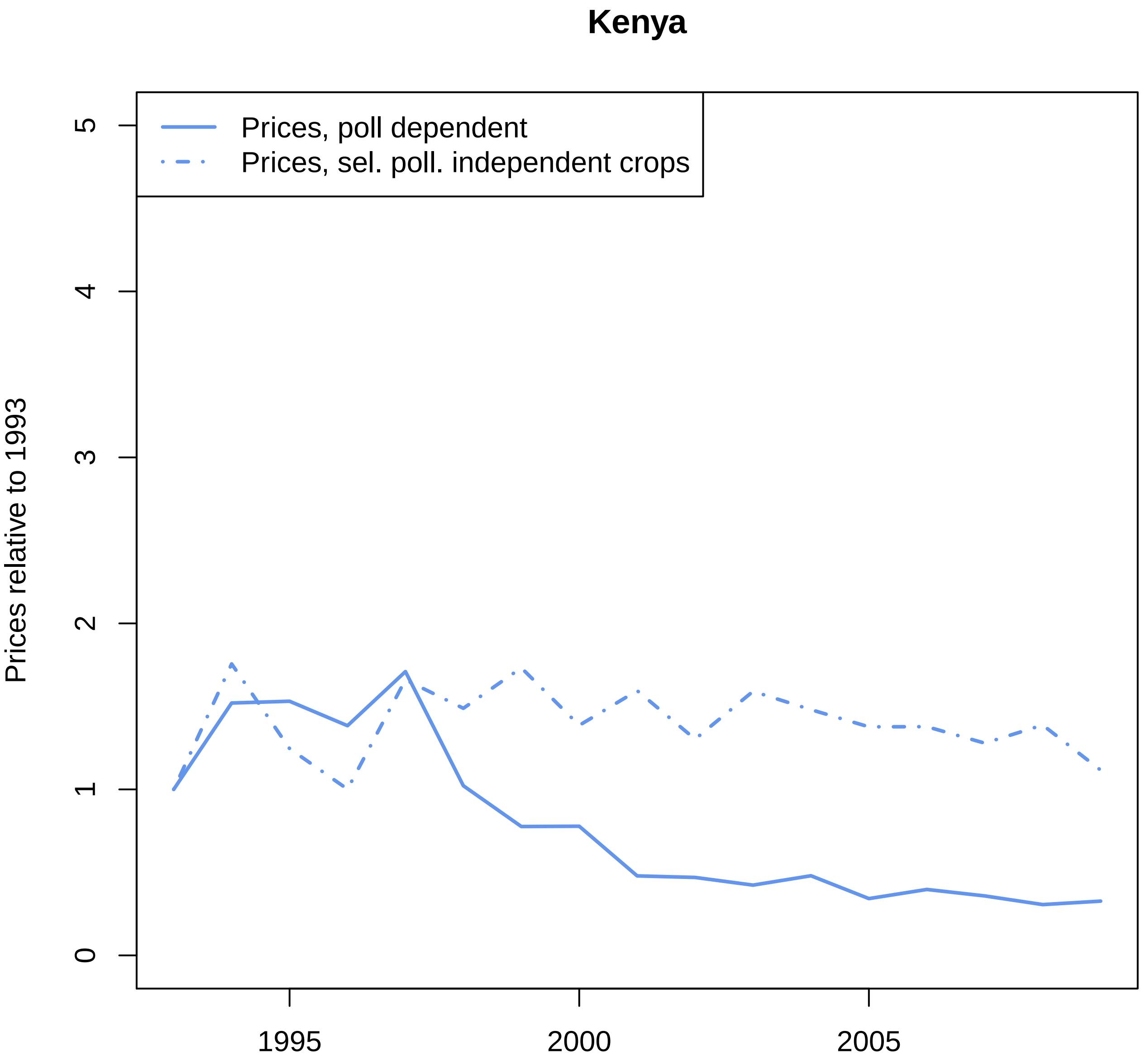

Kenya
Prices, poll dependent
Prices, sel. poll. independent crops
Prices relative to 1993
0
1
2
3
4
5
1995
2000
2005

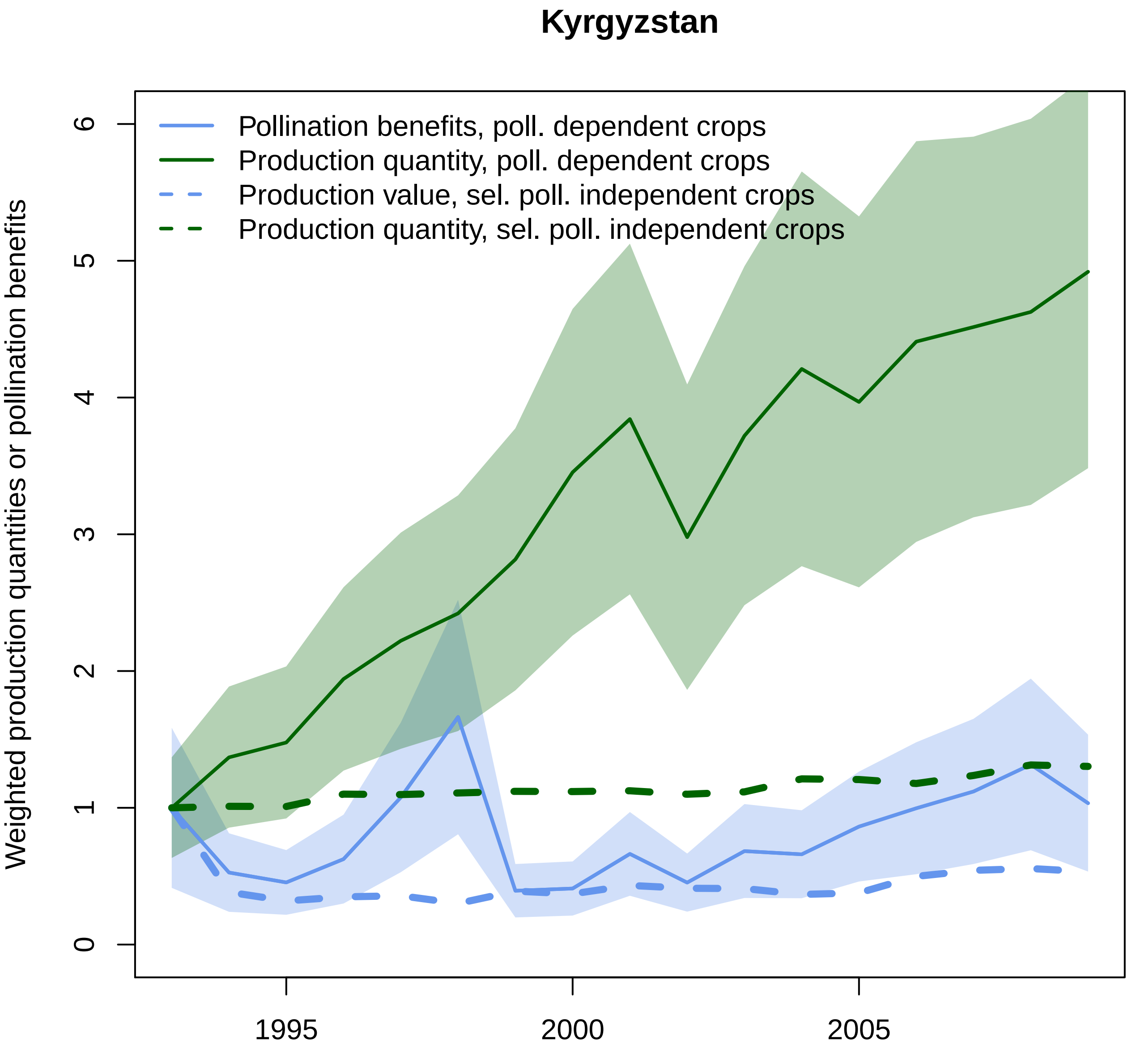

Kyrgyzstan
Pollination benefits, poll. dependent crops
Production quantity, poll. dependent crops
Production value, sel. poll. independent crops
Production quantity, sel. poll. independent crops
Weighted production quantities or pollination benefits
0
1
2
3
4
5
6
1995
2000
2005

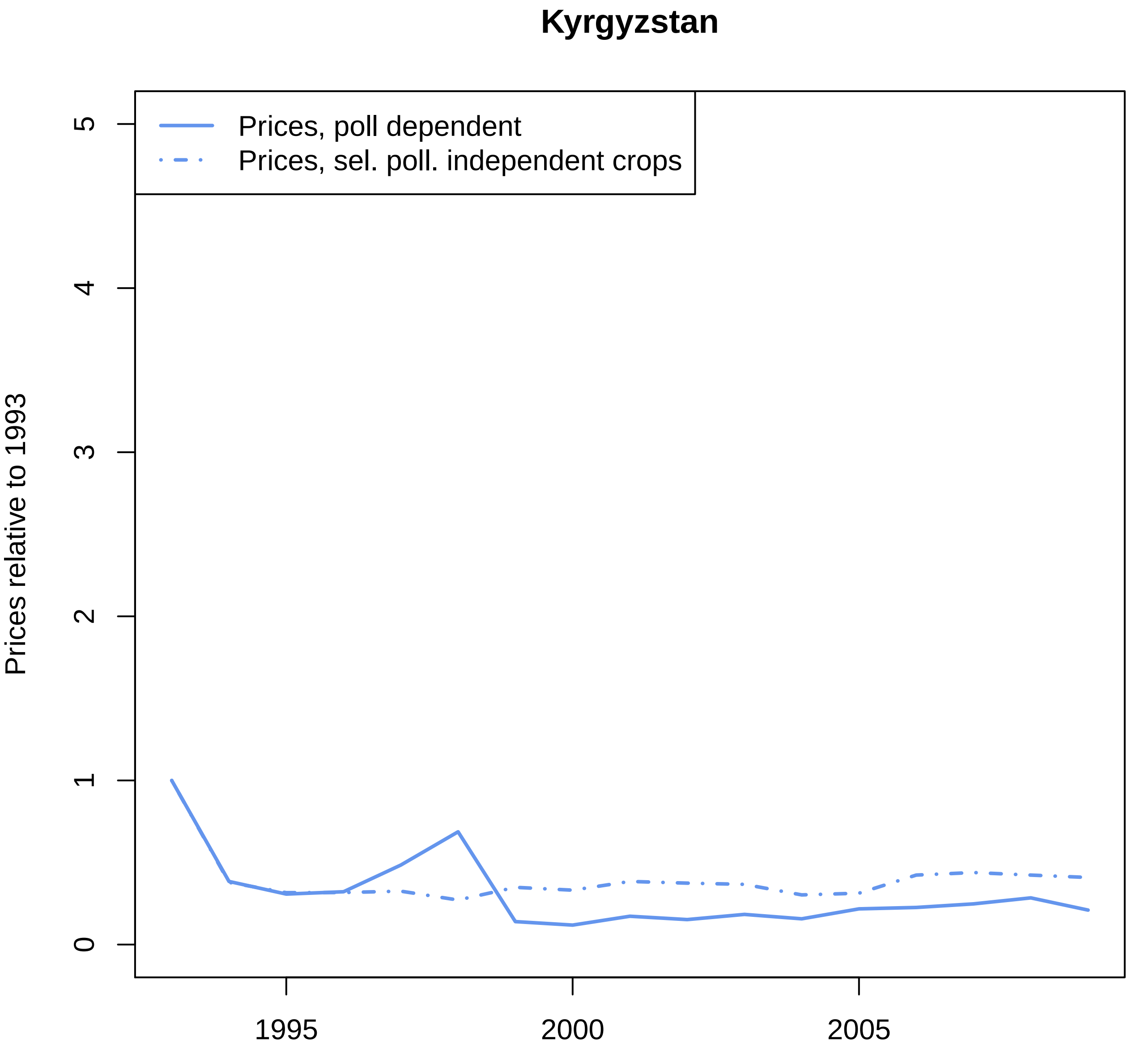
Kyrgyzstan
Prices, poll dependent
Prices, sel. poll. independent crops
Prices relative to 1993
0
1
2
3
4
5
1995
2000
2005

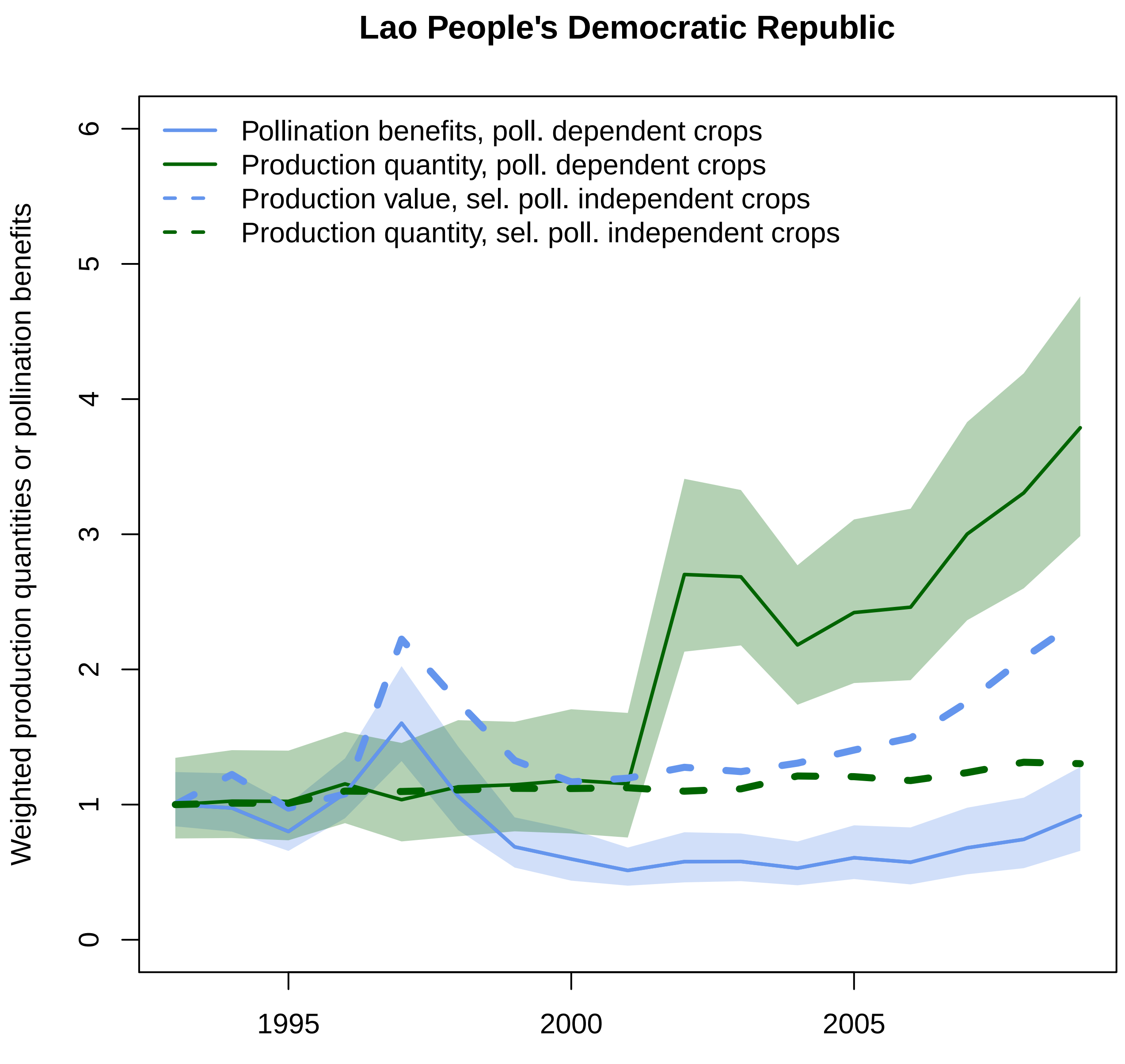

Lao People's Democratic Republic
Pollination benefits, poll. dependent crops
Production quantity, poll. dependent crops
Production value, sel. poll. independent crops
Production quantity, sel. poll. independent crops
Weighted production quantities or pollination benefits
0
1
2
3
4
5
6
1995
2000
2005

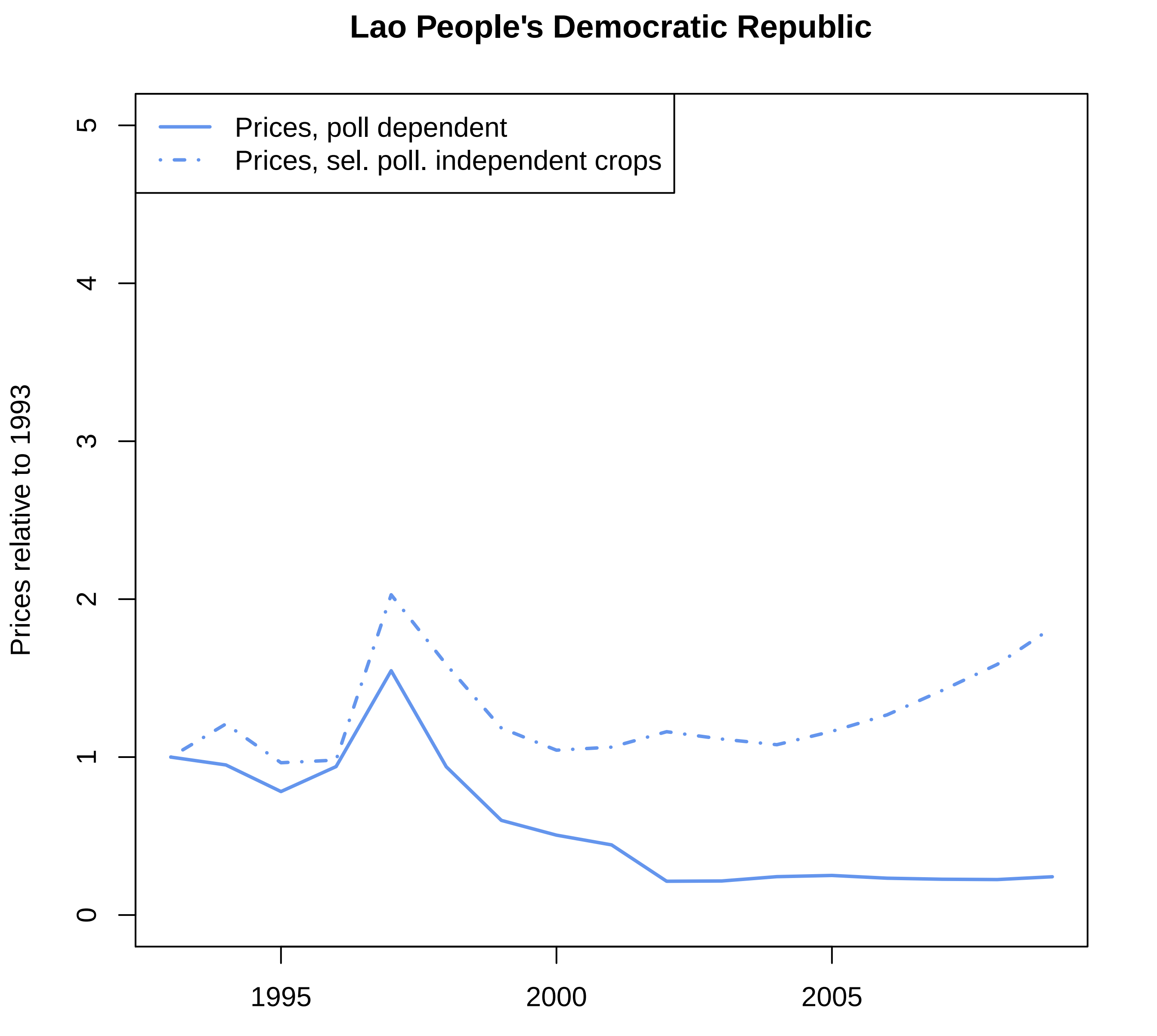

Lao People's Democratic Republic
Prices, poll dependent
Prices, sel. poll. independent crops
Prices relative to 1993
0
1
2
3
4
5
1995
2000
2005

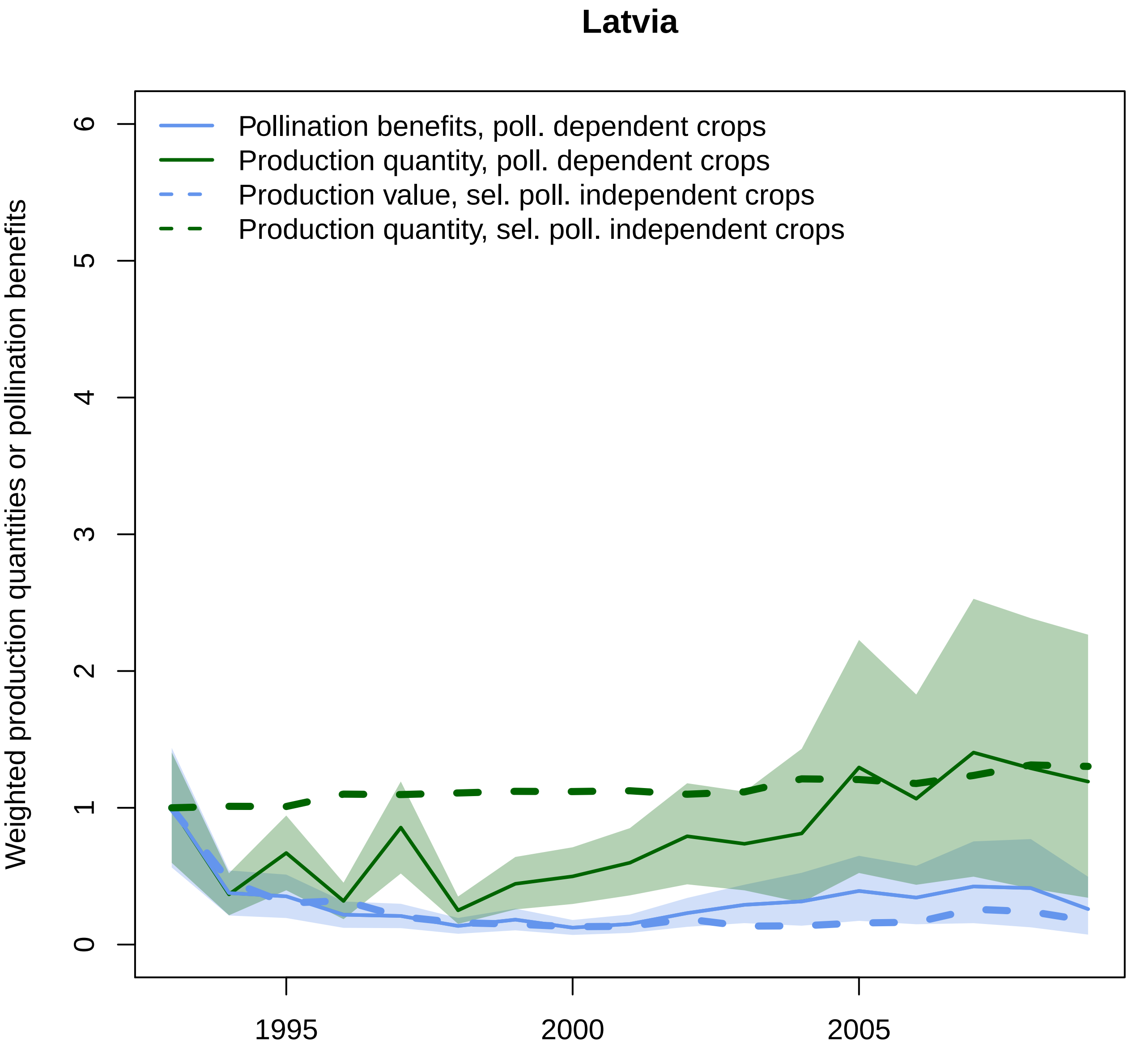

Latvia
Pollination benefits, poll. dependent crops
Production quantity, poll. dependent crops
Production value, sel. poll. independent crops
Production quantity, sel. poll. independent crops
Weighted production quantities or pollination benefits
0
1
2
3
4
5
6
1995
2000
2005

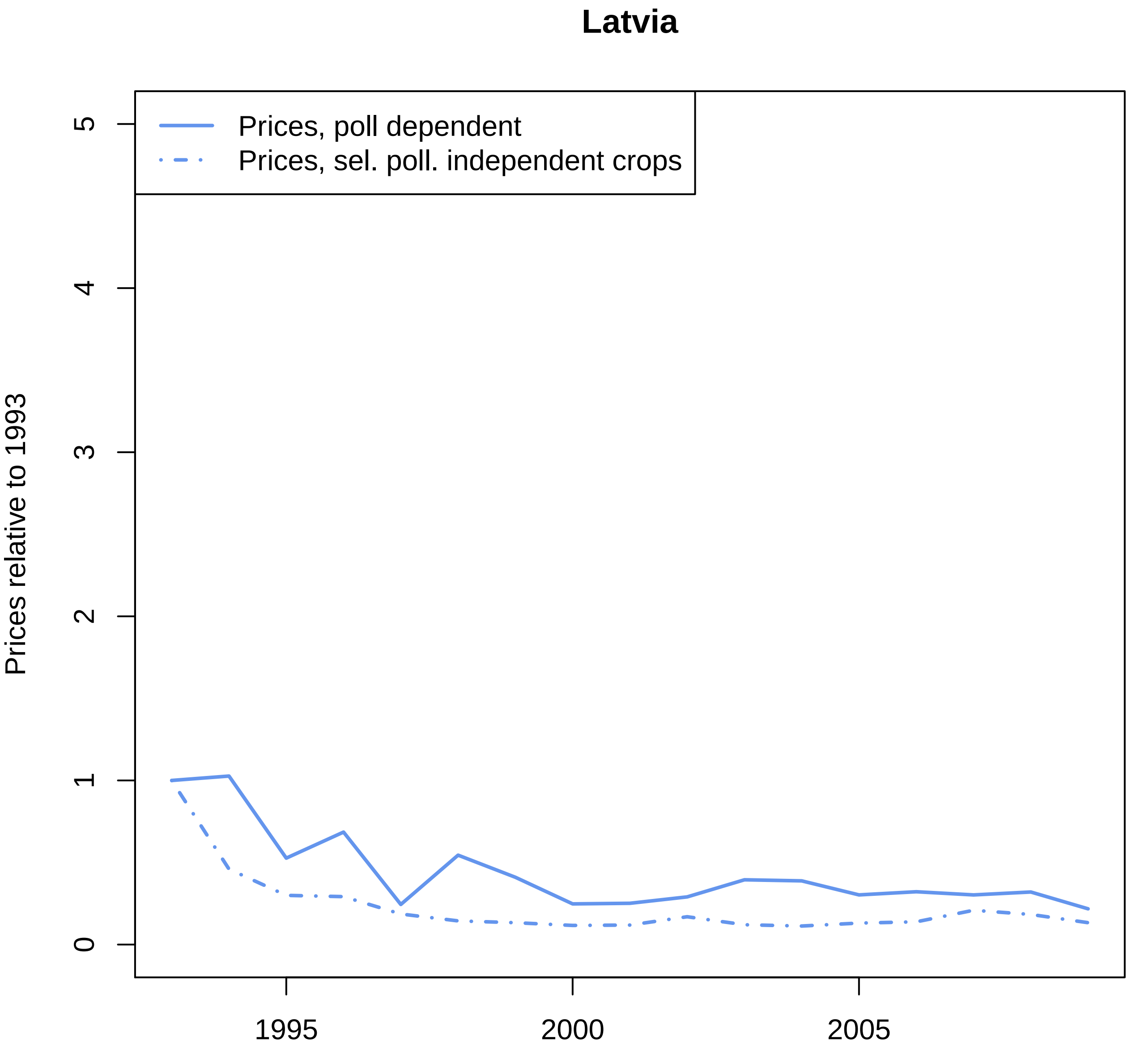

Latvia
Prices, poll dependent
Prices, sel. poll. independent crops
Prices relative to 1993
0
1
2
3
4
5
1995
2000
2005

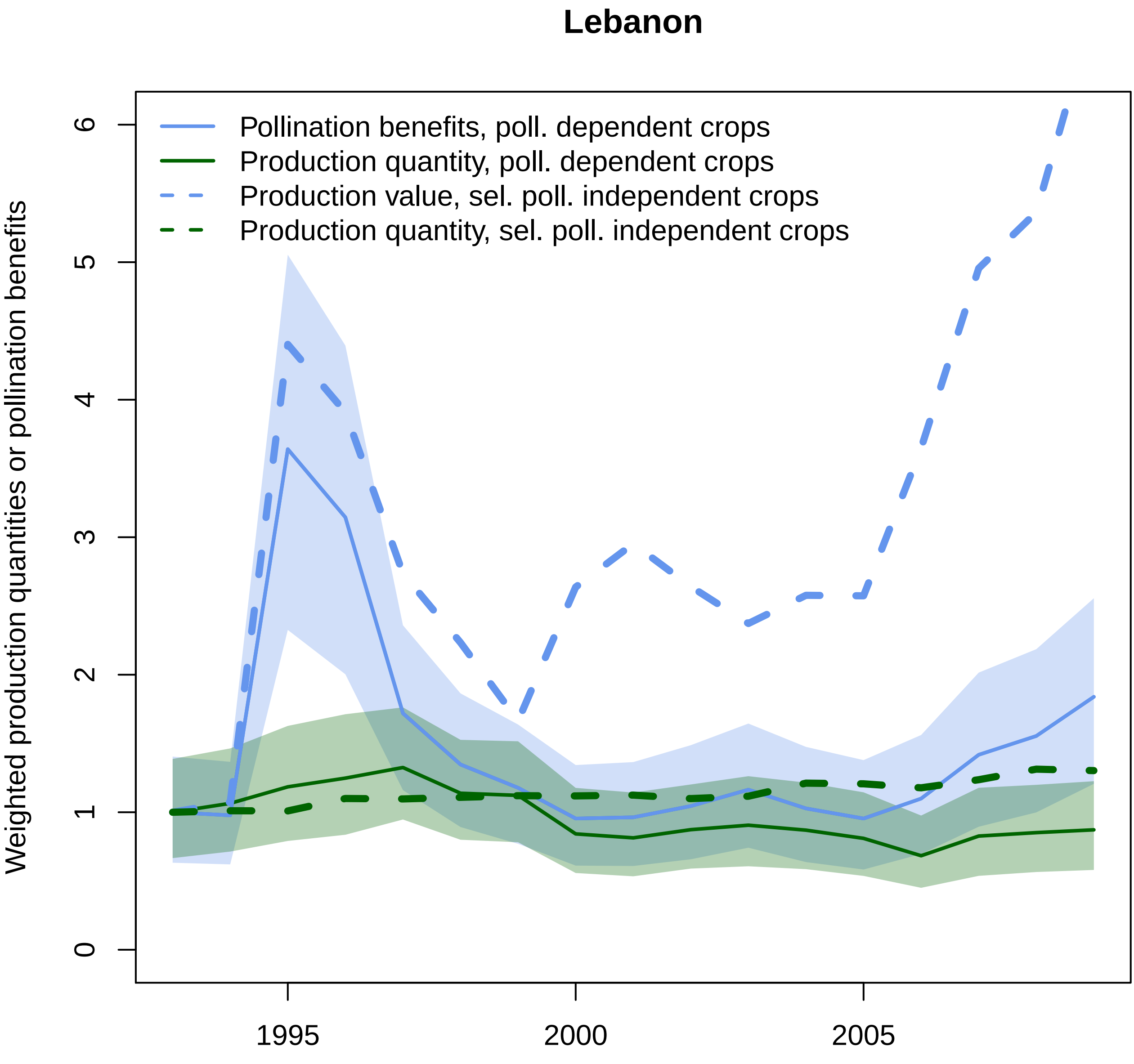

Lebanon
Pollination benefits, poll. dependent crops
Production quantity, poll. dependent crops
Production value, sel. poll. independent crops
Production quantity, sel. poll. independent crops
Weighted production quantities or pollination benefits
0
1
2
3
4
5
6
1995
2000
2005

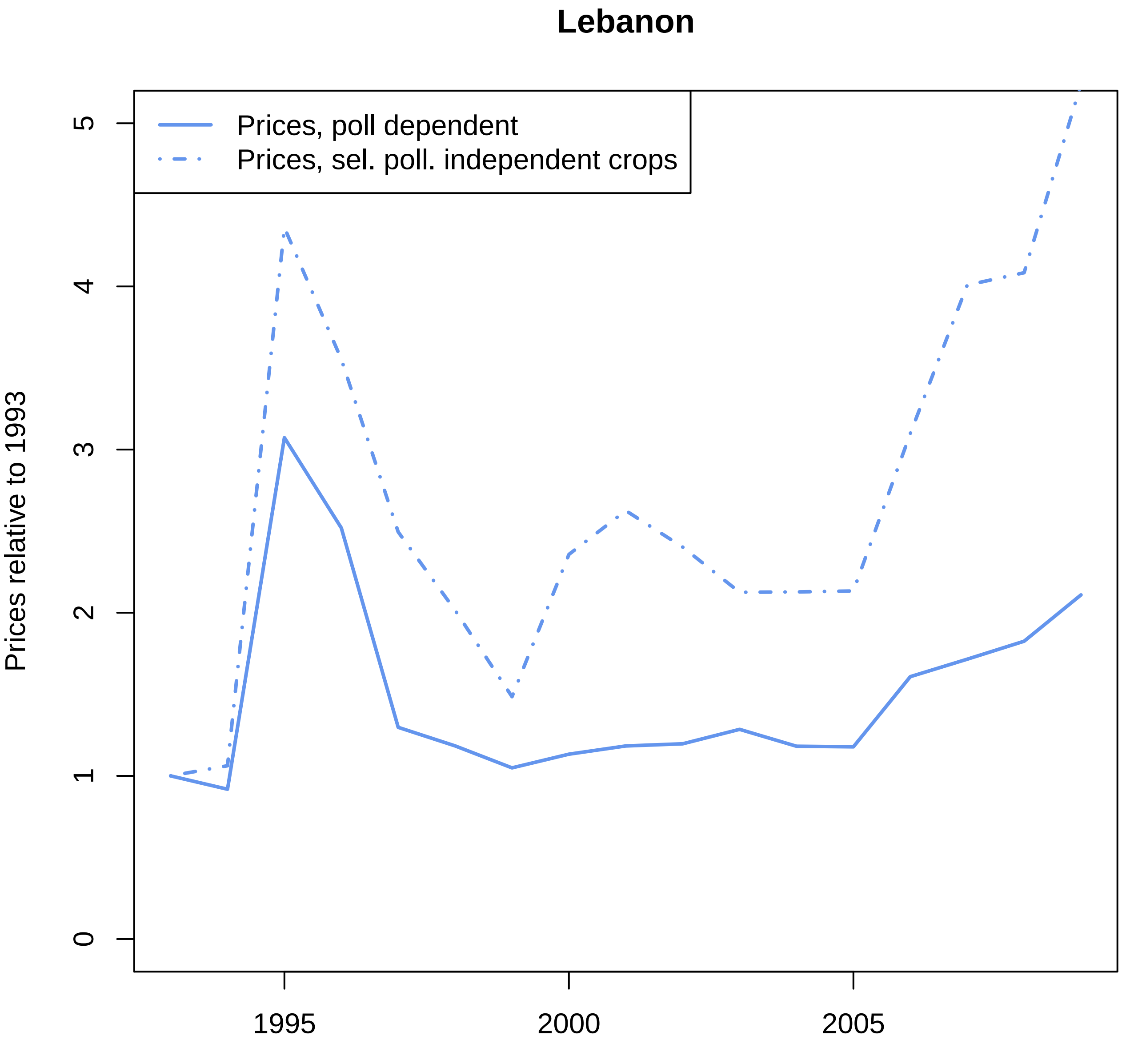
Lebanon
Prices, poll dependent
Prices, sel. poll. independent crops
Prices relative to 1993
0
1
2
3
4
5
1995
2000
2005

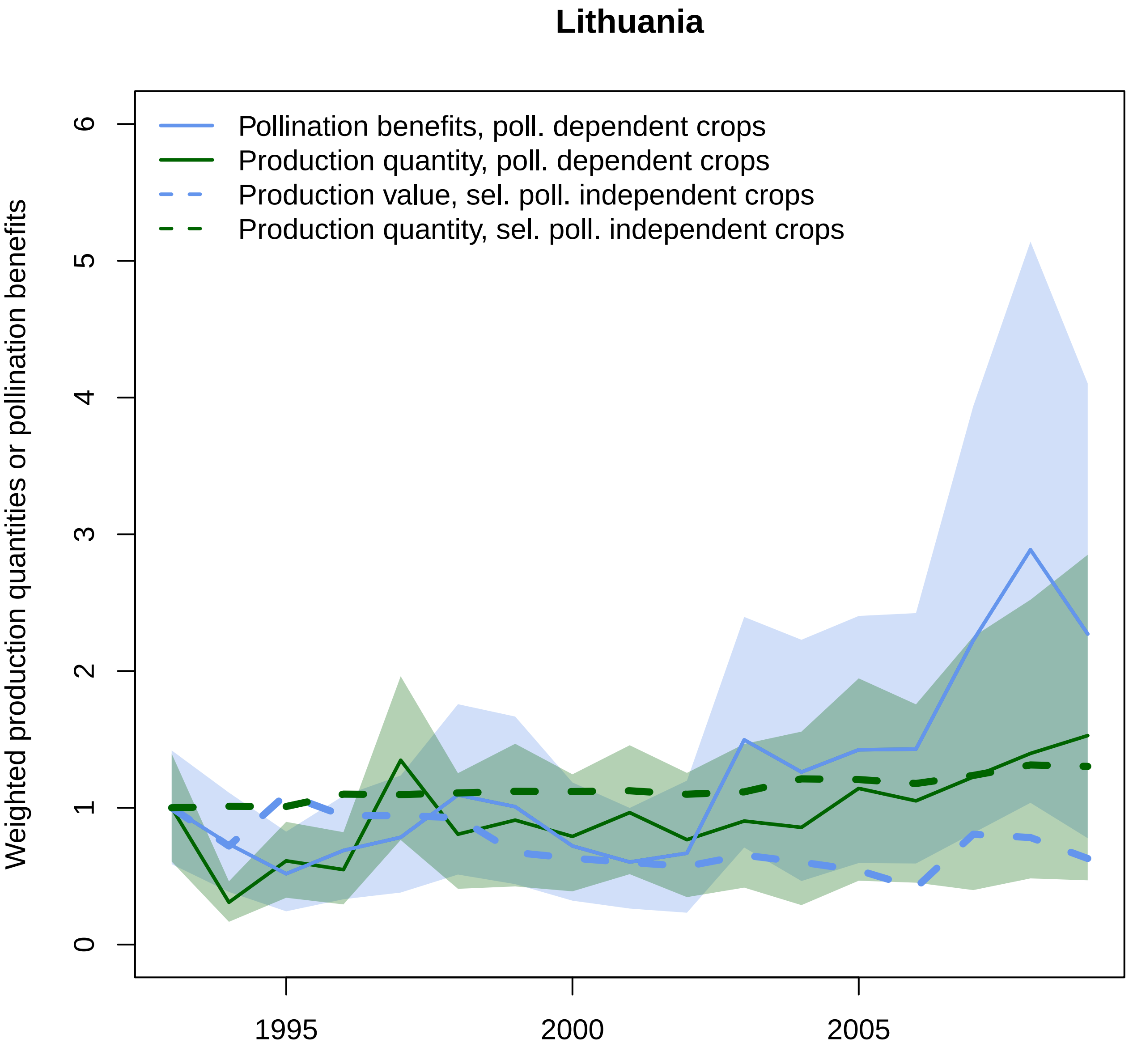

Lithuania
Pollination benefits, poll. dependent crops
Production quantity, poll. dependent crops
Production value, sel. poll. independent crops
Production quantity, sel. poll. independent crops
Weighted production quantities or pollination benefits
0
1
2
3
4
5
6
1995
2000
2005

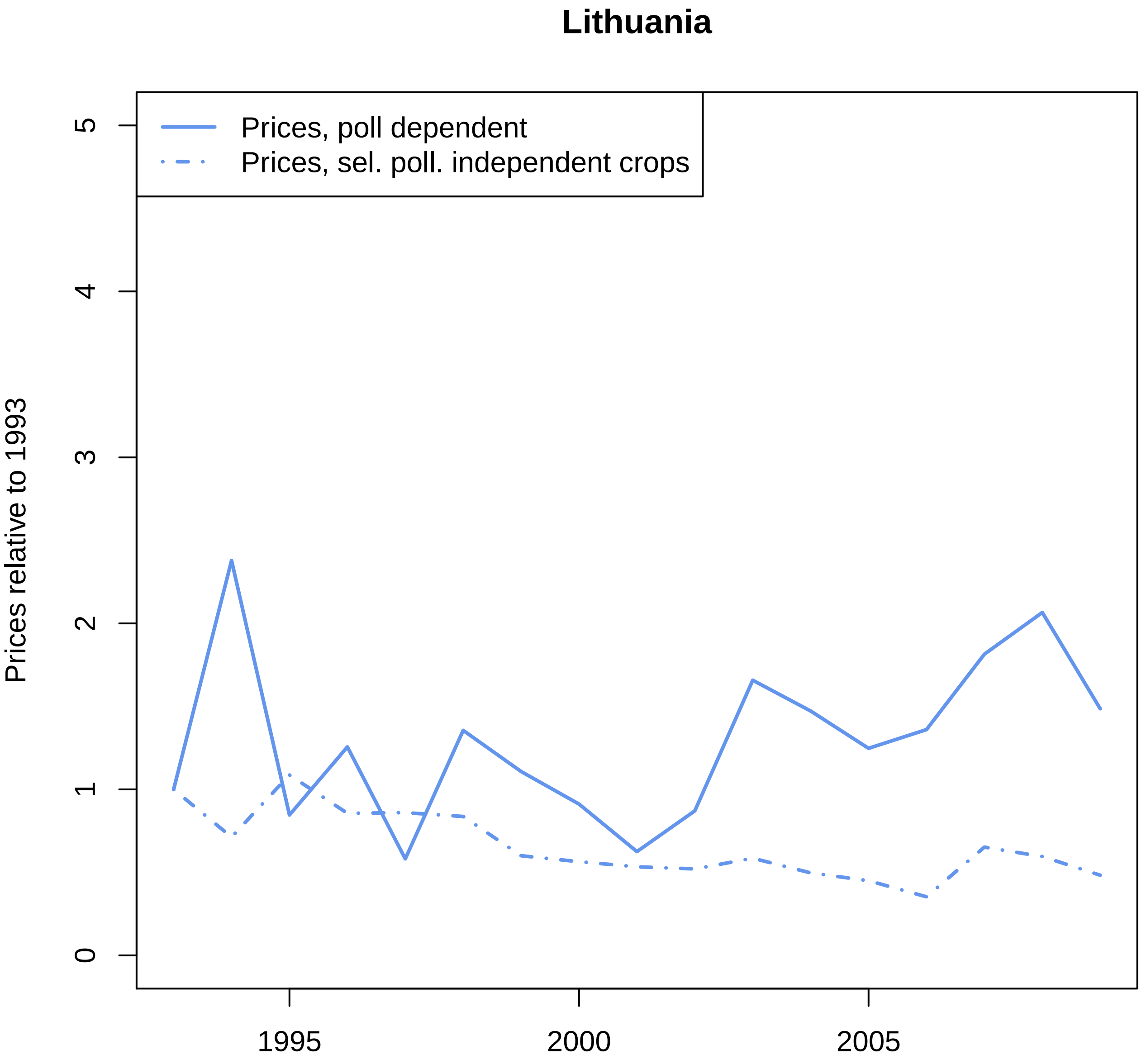
Lithuania
Prices, poll dependent
Prices, sel. poll. independent crops
Prices relative to 1993
0
1
2
3
4
5
1995
2000
2005

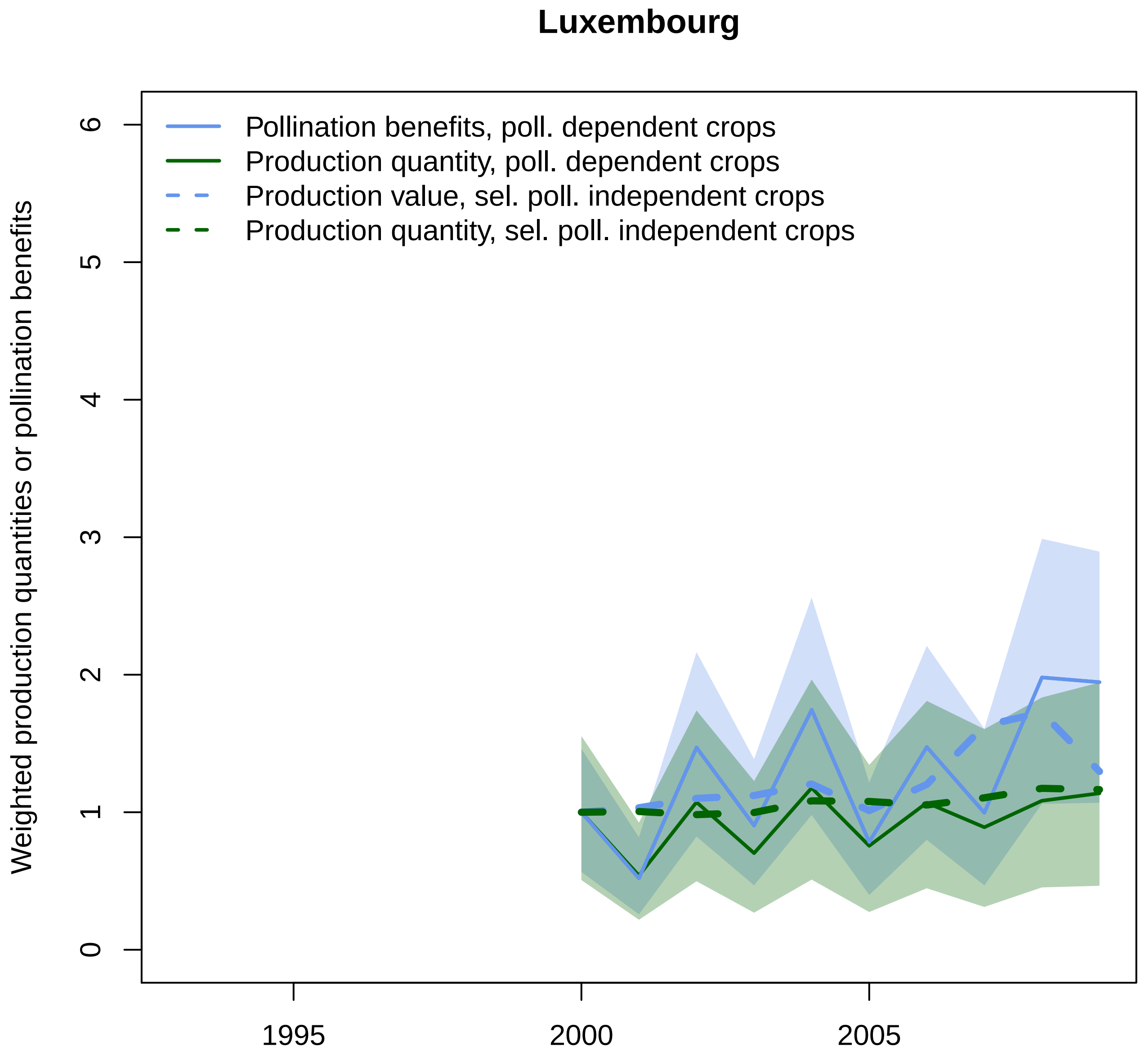
Luxembourg
Pollination benefits, poll. dependent crops
Production quantity, poll. dependent crops
Production value, sel. poll. independent crops
Production quantity, sel. poll. independent crops
Weighted production quantities or pollination benefits
0
1
2
3
4
5
6
1995
2000
2005

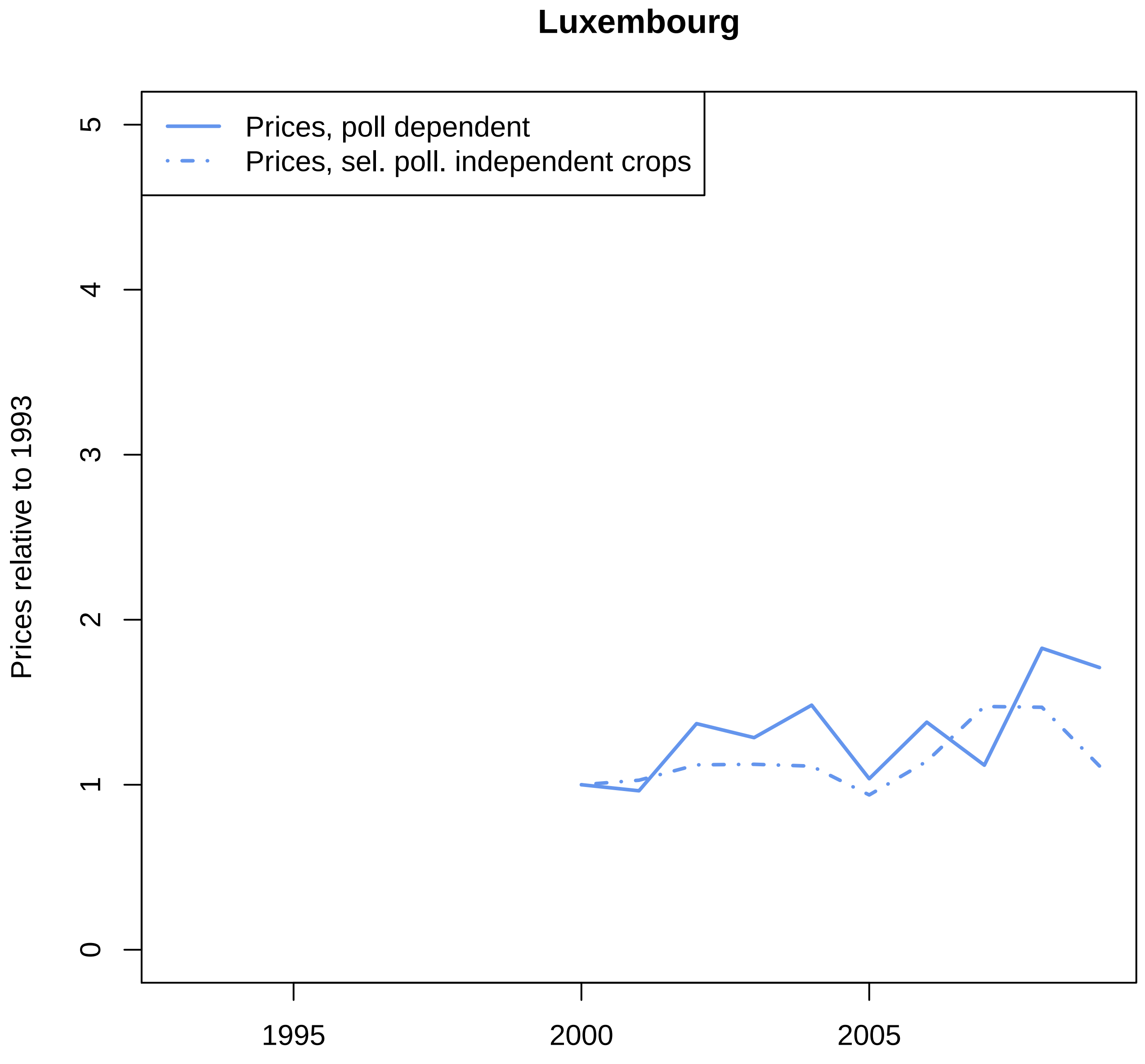
Luxembourg
Prices, poll dependent
Prices, sel. poll. independent crops
Prices relative to 1993
0
1
2
3
4
5
1995
2000
2005

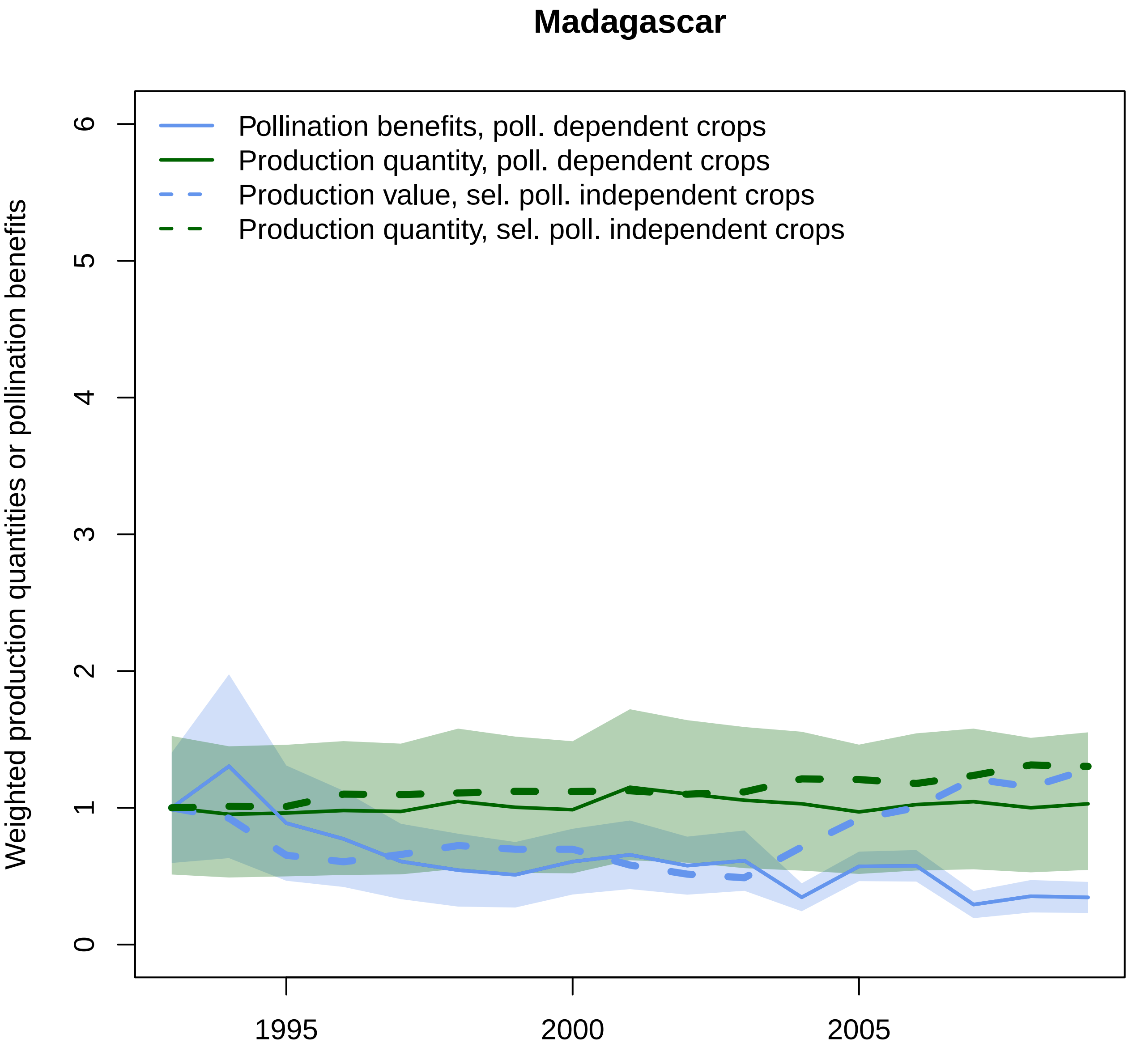
Madagascar
Pollination benefits, poll. dependent crops
Production quantity, poll. dependent crops
Production value, sel. poll. independent crops
Production quantity, sel. poll. independent crops
Weighted production quantities or pollination benefits
0
1
2
3
4
5
6
1995
2000
2005

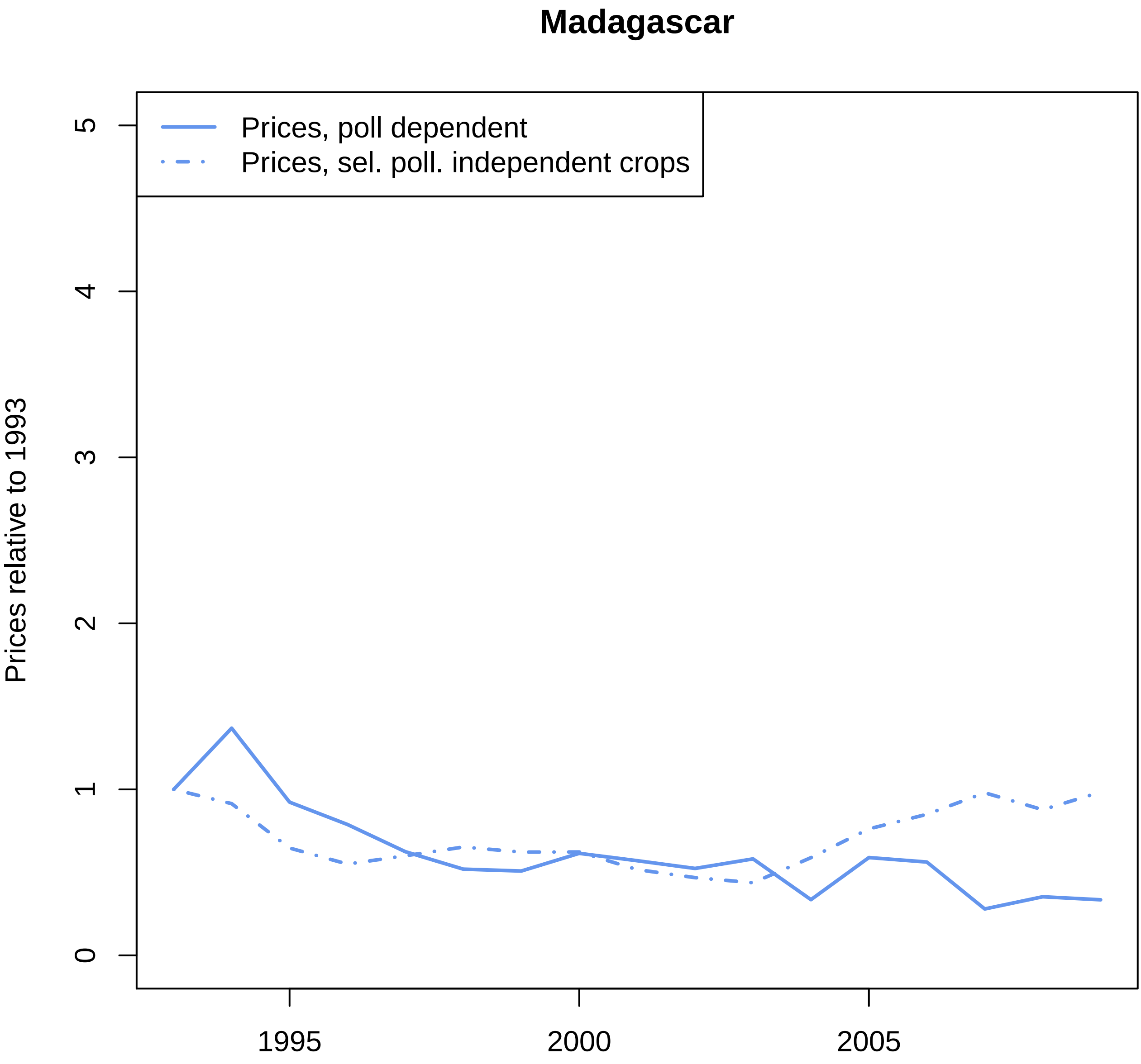

Madagascar
Prices, poll dependent
Prices, sel. poll. independent crops
Prices relative to 1993
0
1
2
3
4
5
1995
2000
2005

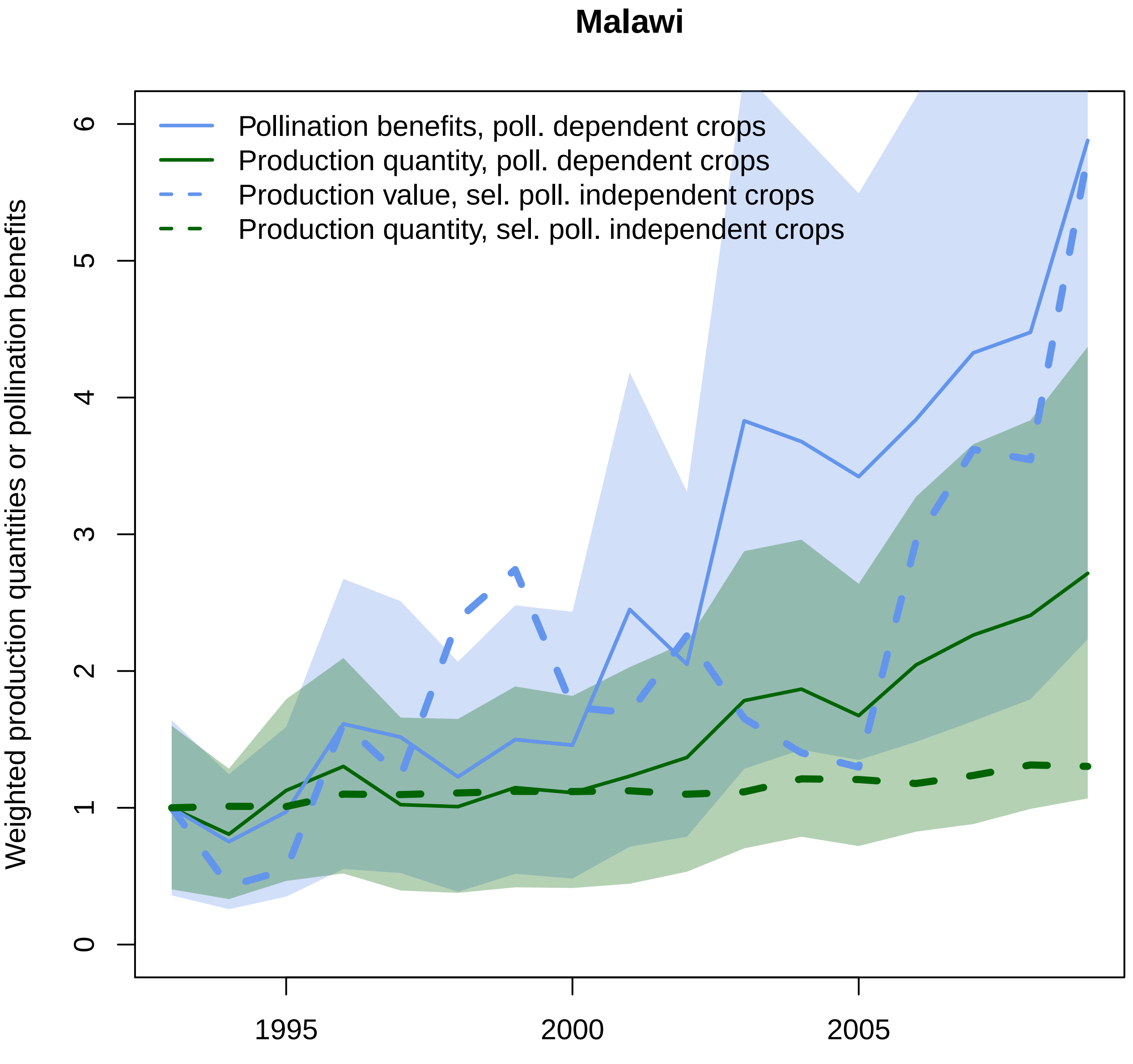
Malawi
Pollination benefits, poll. dependent crops
Production quantity, poll. dependent crops
Production value, sel. poll. independent crops
Production quantity, sel. poll. independent crops
Weighted production quantities or pollination benefits
0
1
2
3
4
5
6
1995
2000
2005

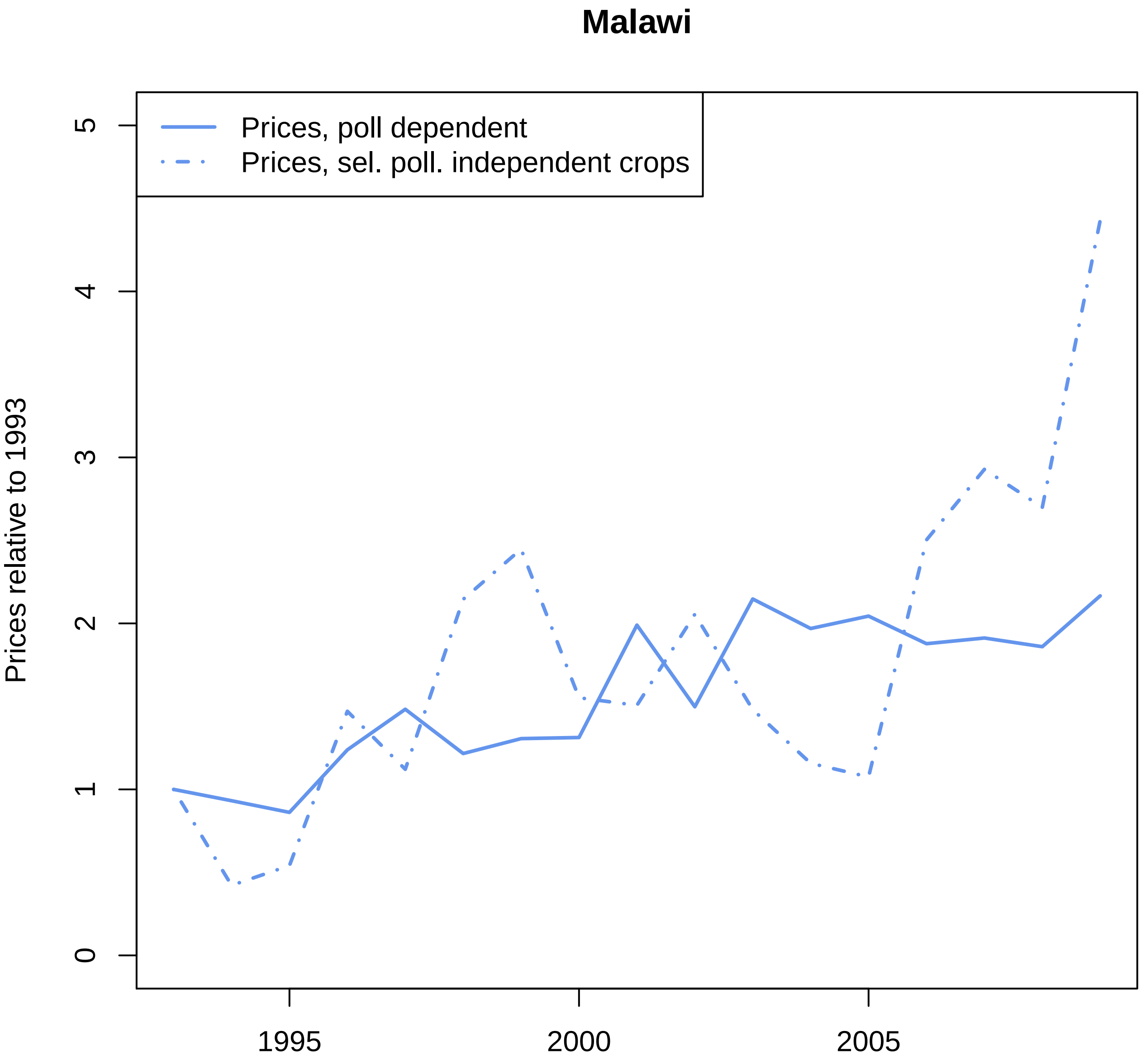

Malawi
Prices, poll dependent
Prices, sel. poll. independent crops
Prices relative to 1993
0
1
2
3
4
5
1995
2000
2005

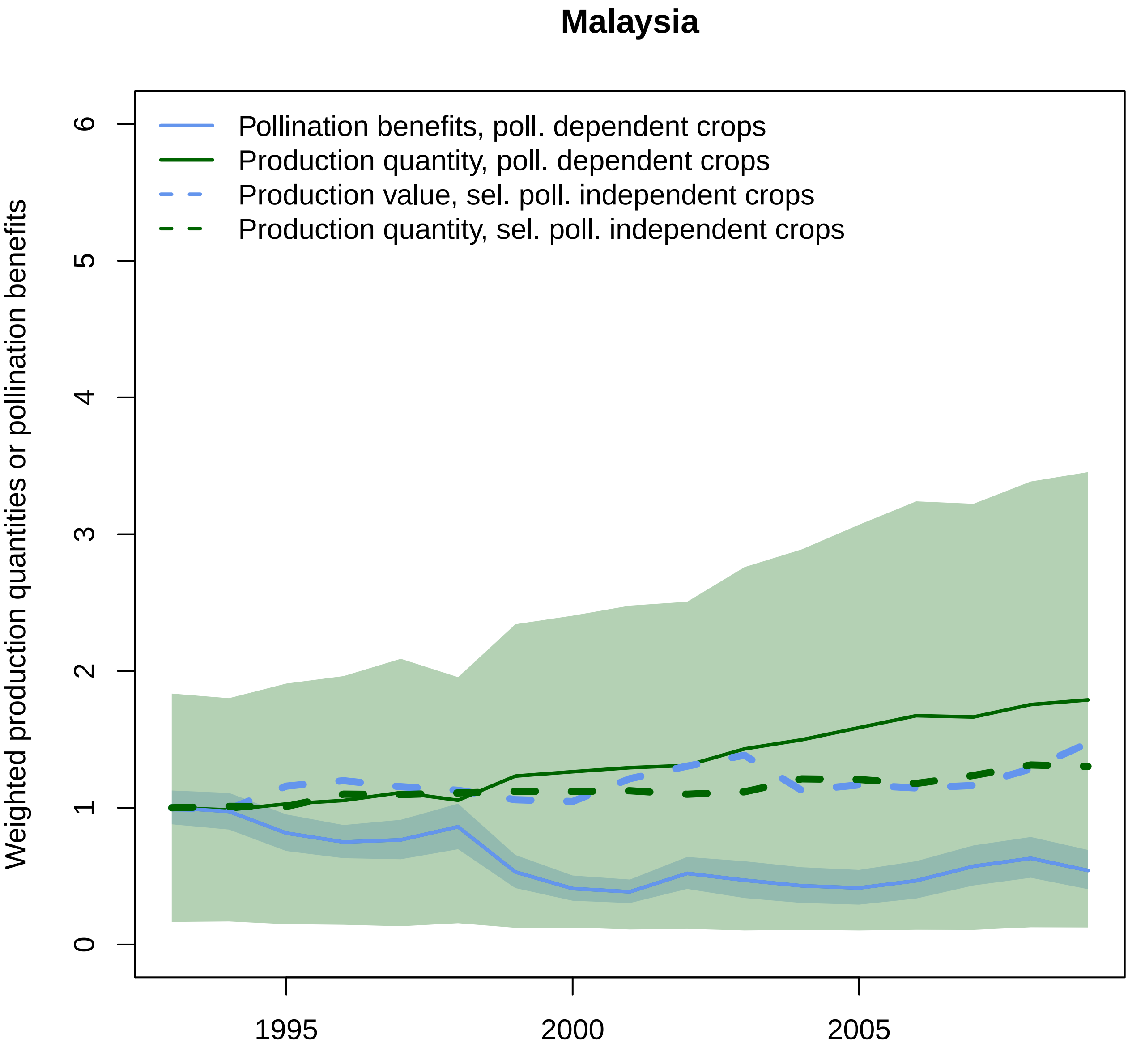

Malaysia
Pollination benefits, poll. dependent crops
Production quantity, poll. dependent crops
Production value, sel. poll. independent crops
Production quantity, sel. poll. independent crops
Weighted production quantities or pollination benefits
0
1
2
3
4
5
6
1995
2000
2005

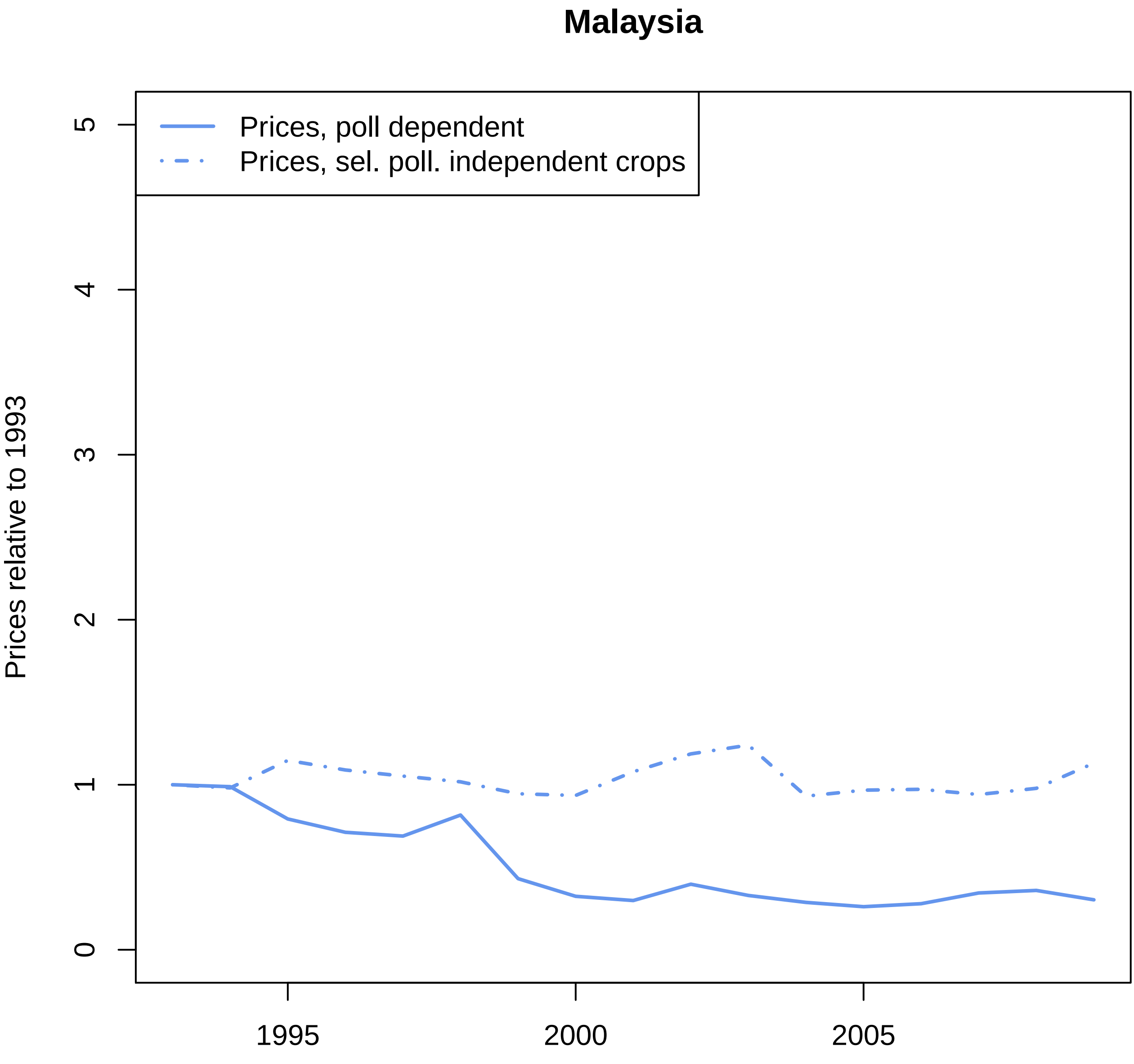

Malaysia
Prices, poll dependent
Prices, sel. poll. independent crops
Prices relative to 1993
0
1
2
3
4
5
1995
2000
2005

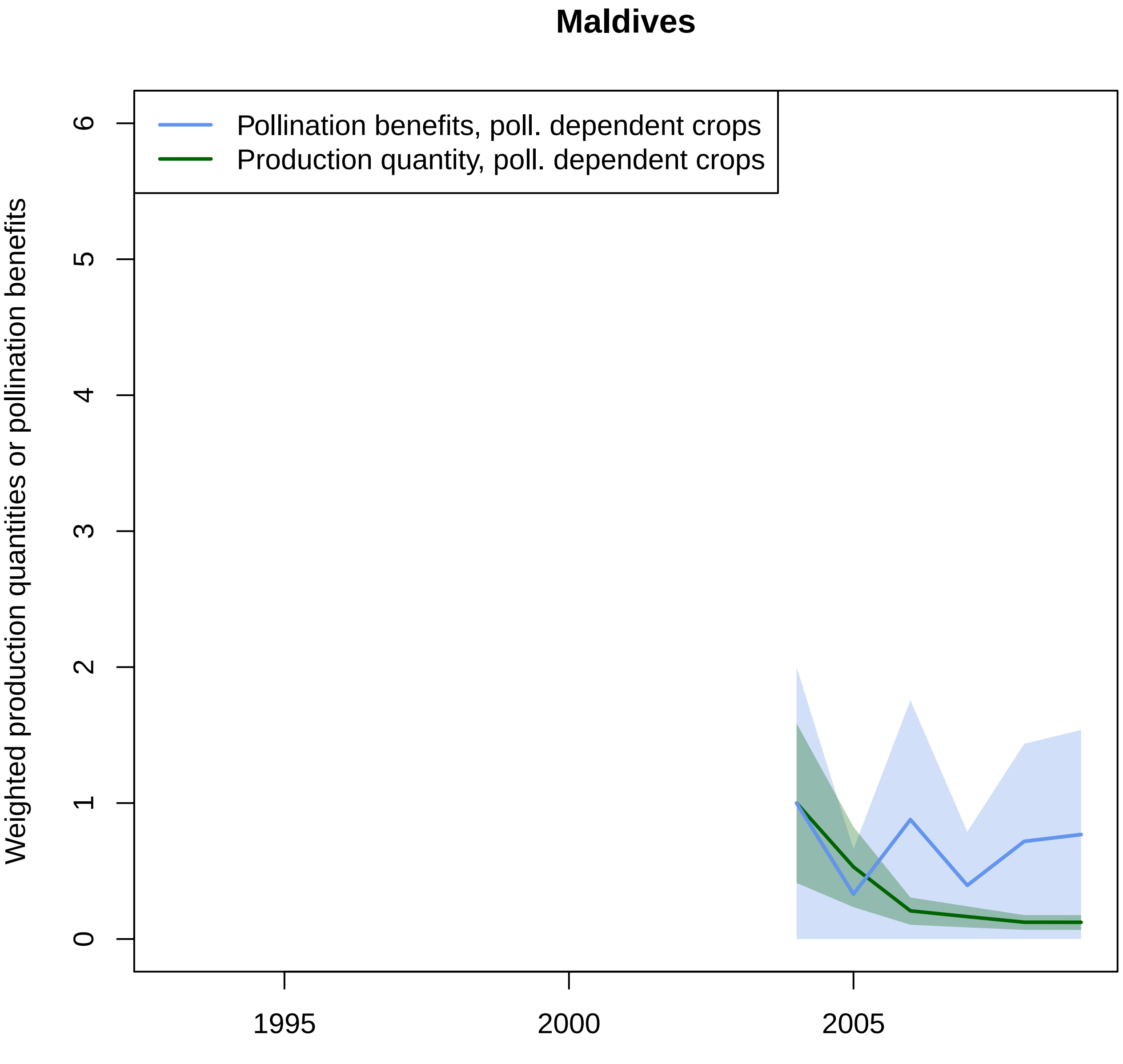

Maldives
Pollination benefits, poll. dependent crops
Production quantity, poll. dependent crops
Weighted production quantities or pollination benefits
0
1
2
3
4
5
6
1995
2000
2005

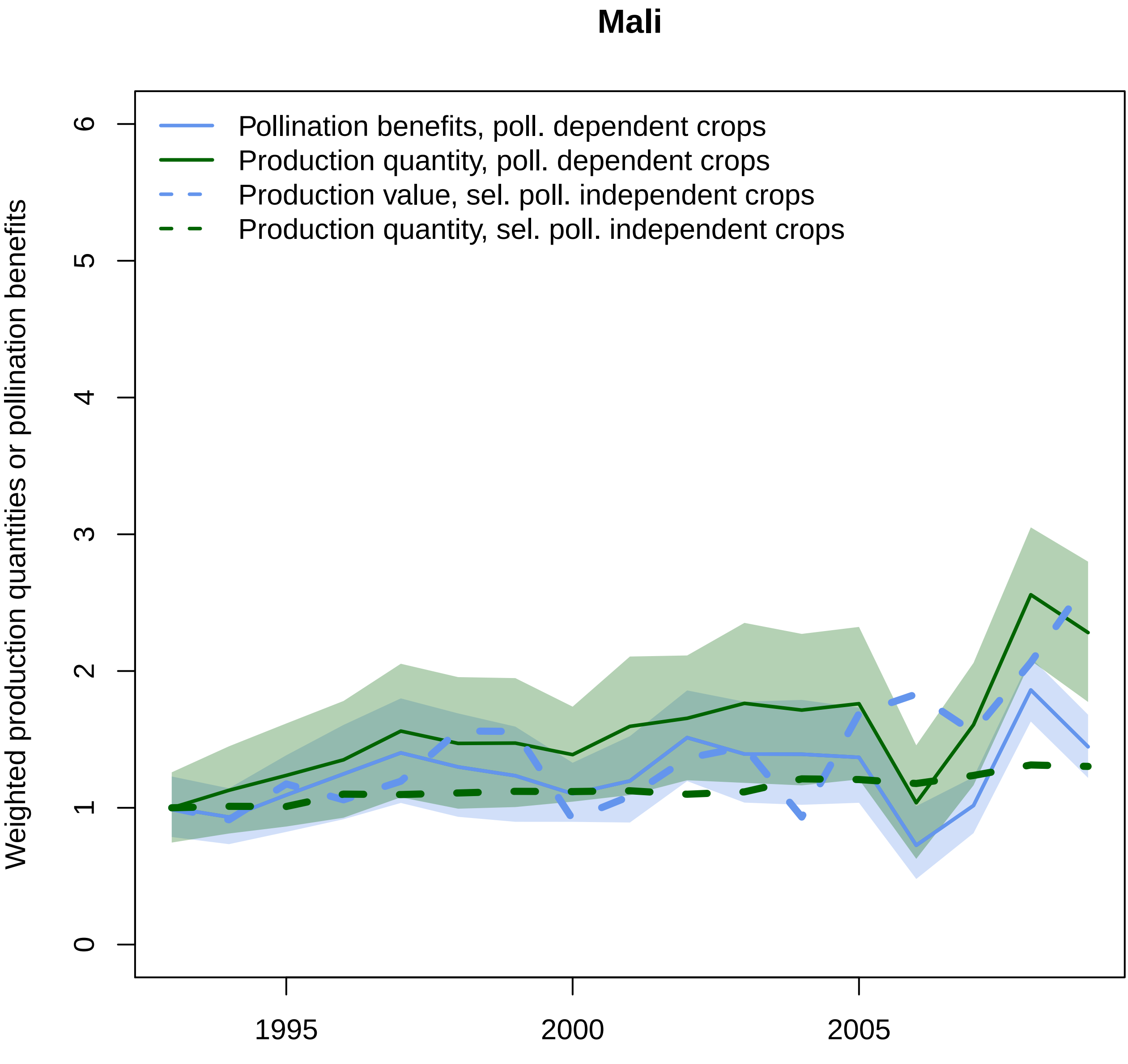
Mali
Pollination benefits, poll. dependent crops
Production quantity, poll. dependent crops
Production value, sel. poll. independent crops
Production quantity, sel. poll. independent crops
Weighted production quantities or pollination benefits
0
1
2
3
4
5
6
1995
2000
2005

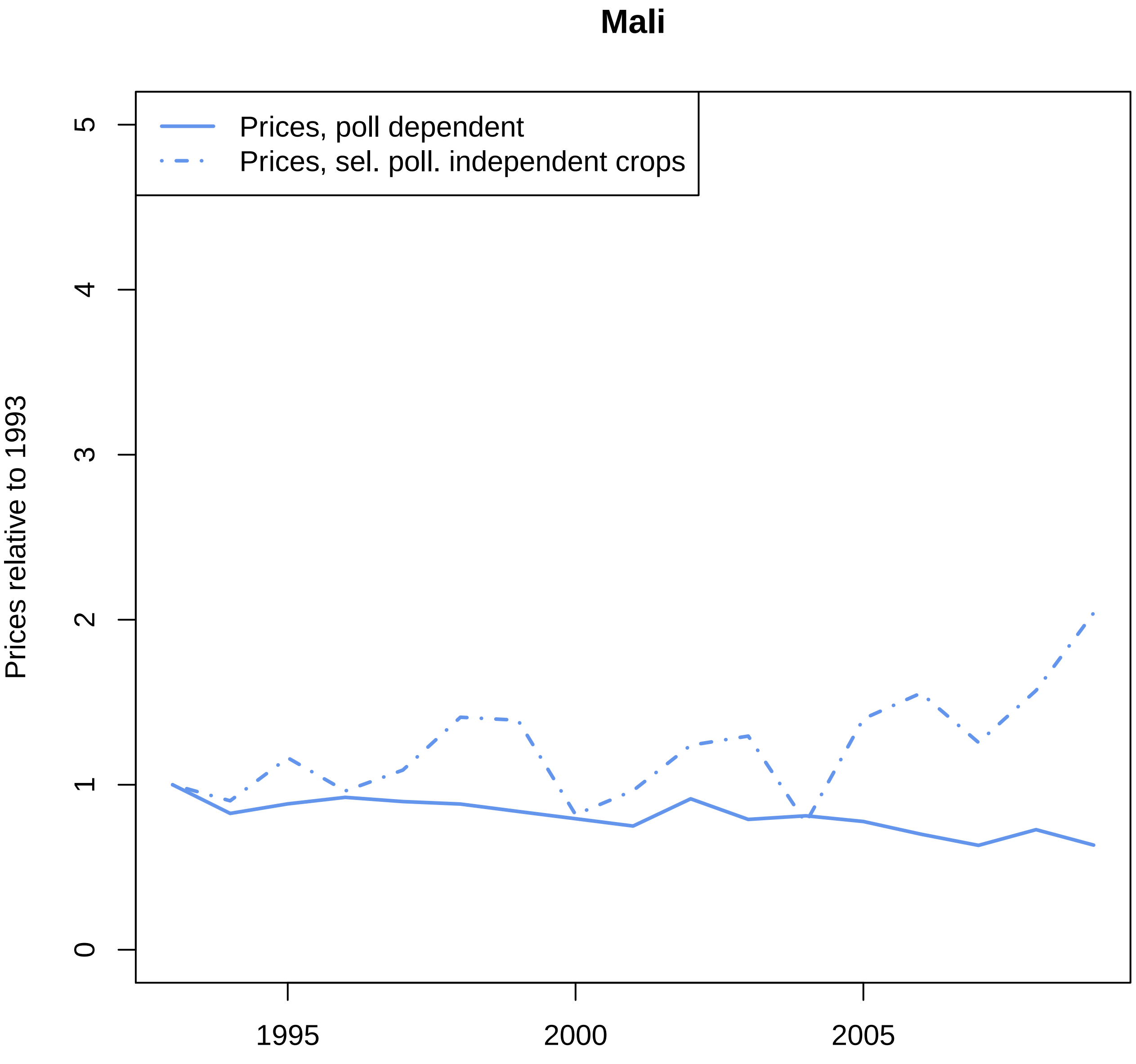

Mali
Prices, poll dependent
Prices, sel. poll. independent crops
Prices relative to 1993
0
1
2
3
4
5
1995
2000
2005

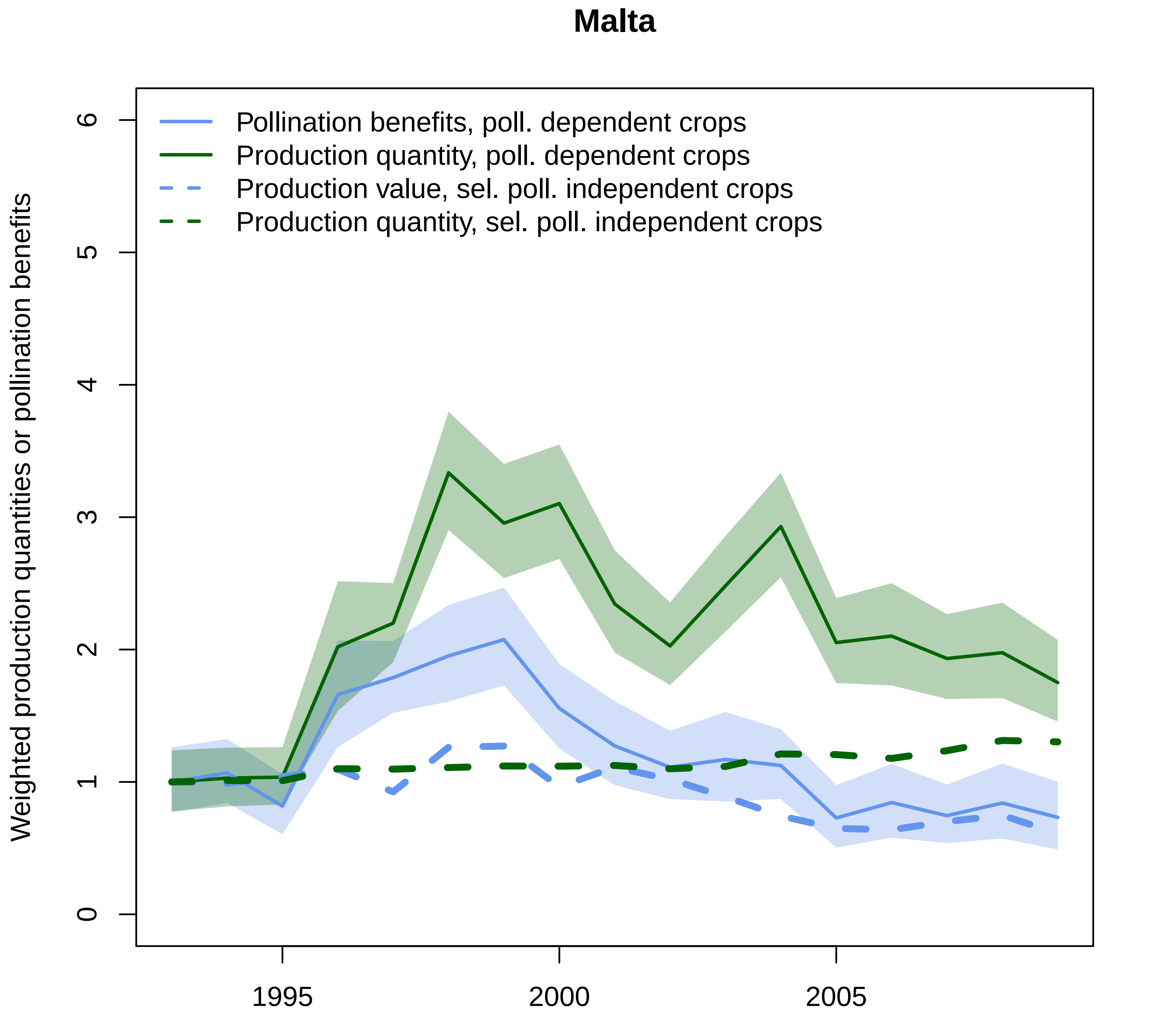

Malta
Pollination benefits, poll. dependent crops
Production quantity, poll. dependent crops
Production value, sel. poll. independent crops
Production quantity, sel. poll. independent crops
Weighted production quantities or pollination benefits
0
1
2
3
4
5
6
1995
2000
2005

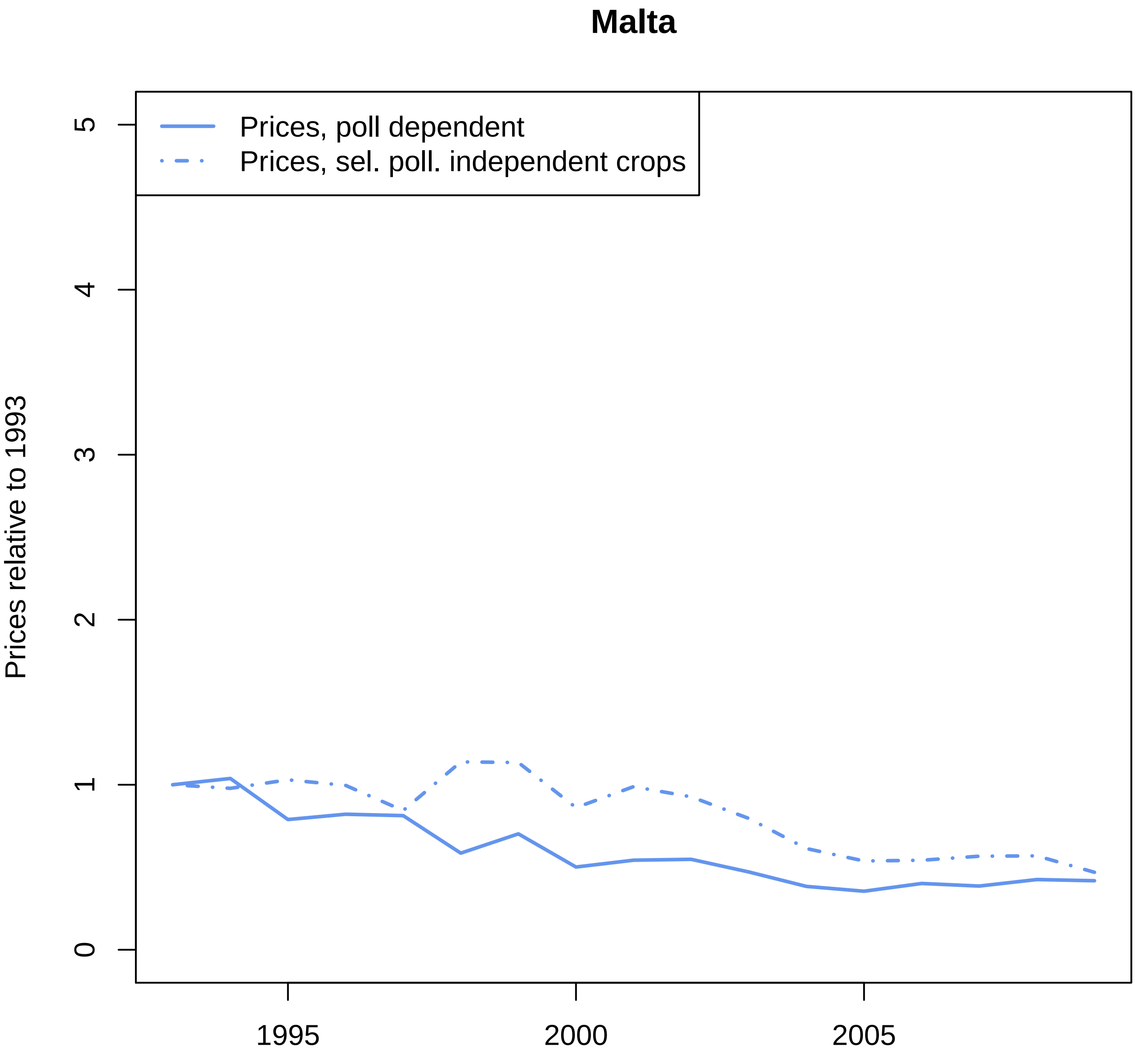

Malta
Prices, poll dependent
Prices, sel. poll. independent crops
Prices relative to 1993
0
1
2
3
4
5
1995
2000
2005

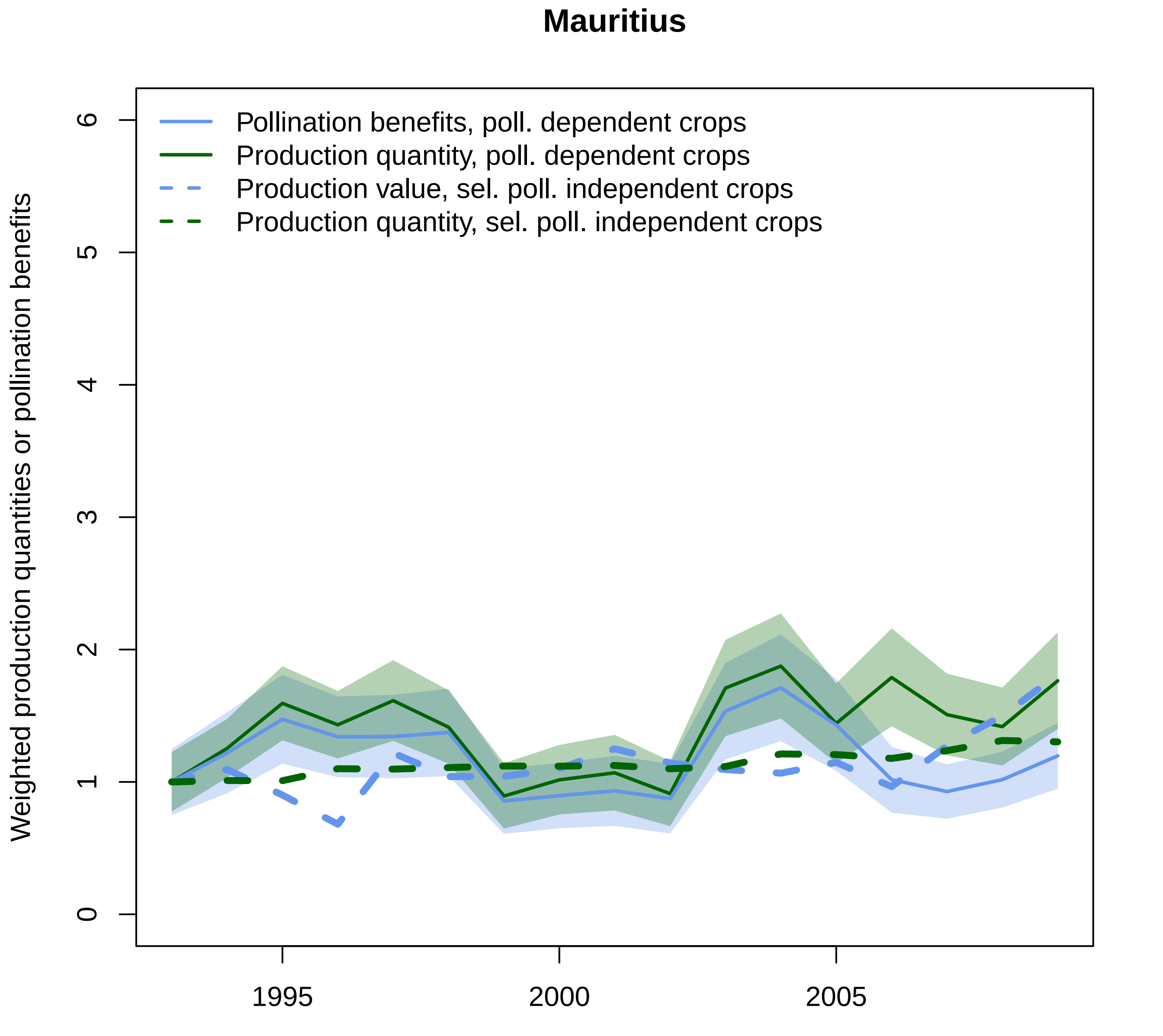

Mauritius
Pollination benefits, poll. dependent crops
Production quantity, poll. dependent crops
Production value, sel. poll. independent crops
Production quantity, sel. poll. independent crops
Weighted production quantities or pollination benefits
0
1
2
3
4
5
6
1995
2000
2005

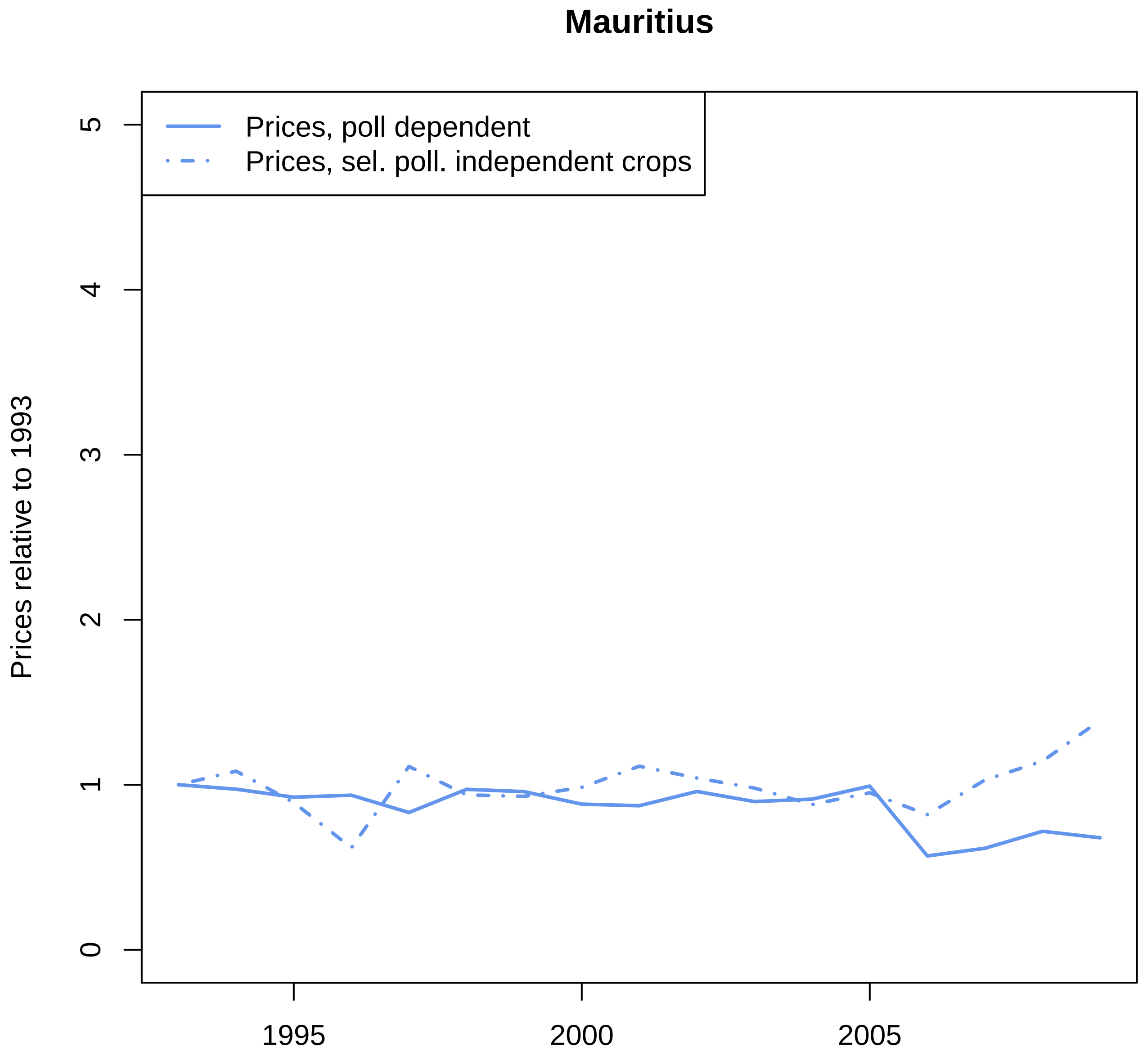
Mauritius
Prices, poll dependent
Prices, sel. poll. independent crops
Prices relative to 1993
0
1
2
3
4
5
1995
2000
2005

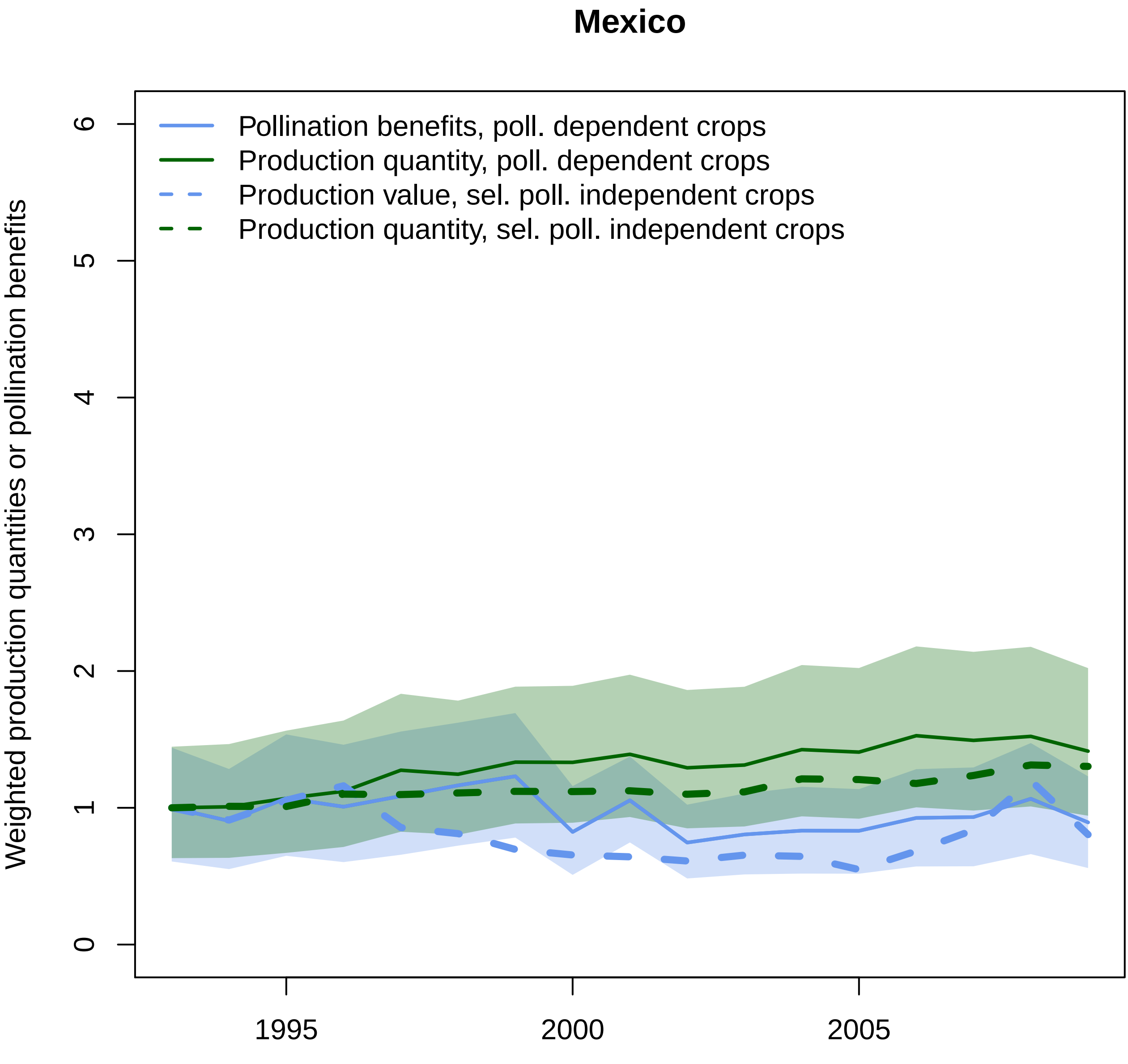
Mexico
Pollination benefits, poll. dependent crops
Production quantity, poll. dependent crops
Production value, sel. poll. independent crops
Production quantity, sel. poll. independent crops
Weighted production quantities or pollination benefits
0
1
2
3
4
5
6
1995
2000
2005

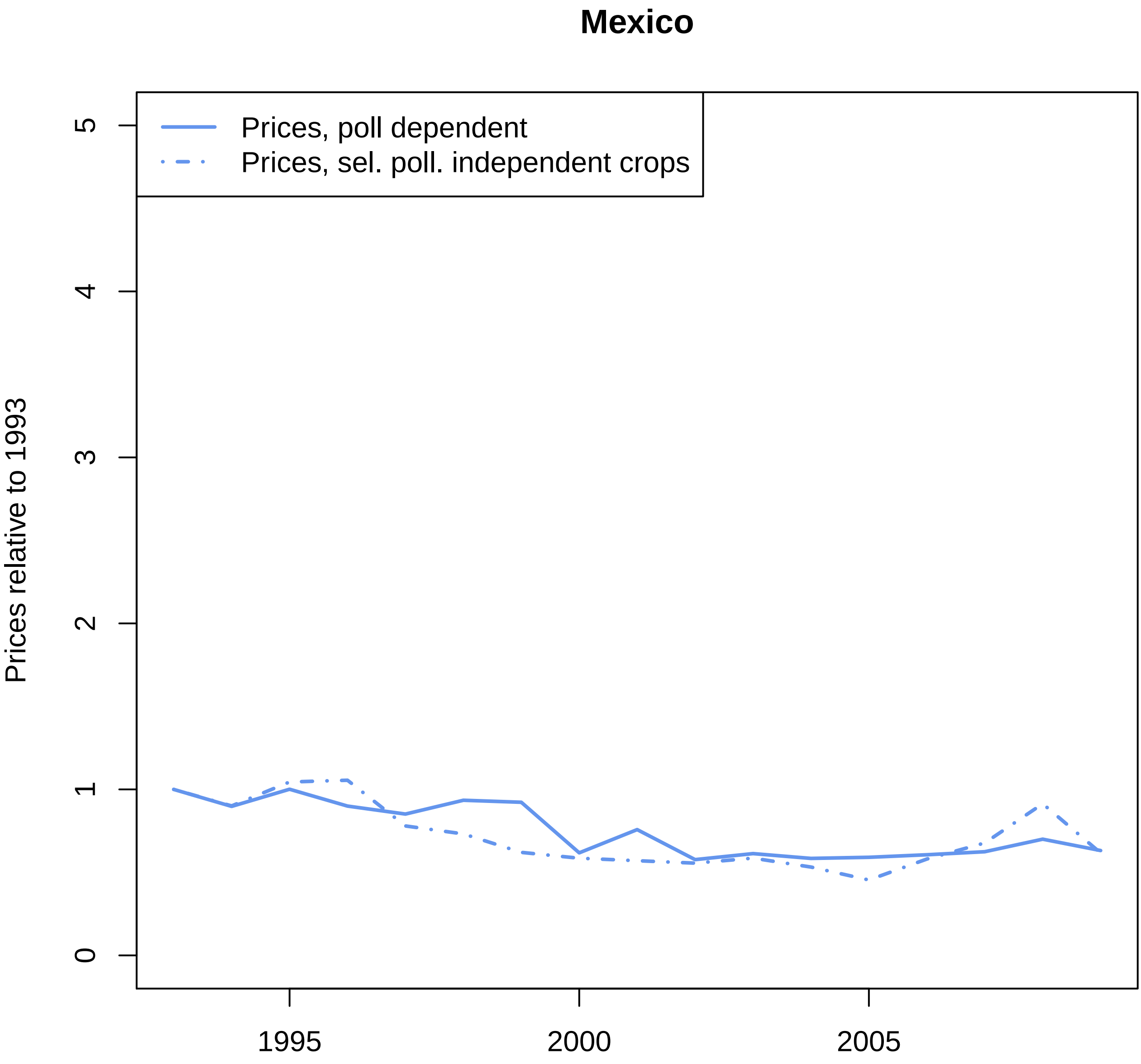
Mexico
Prices, poll dependent
Prices, sel. poll. independent crops
Prices relative to 1993
0
1
2
3
4
5
1995
2000
2005

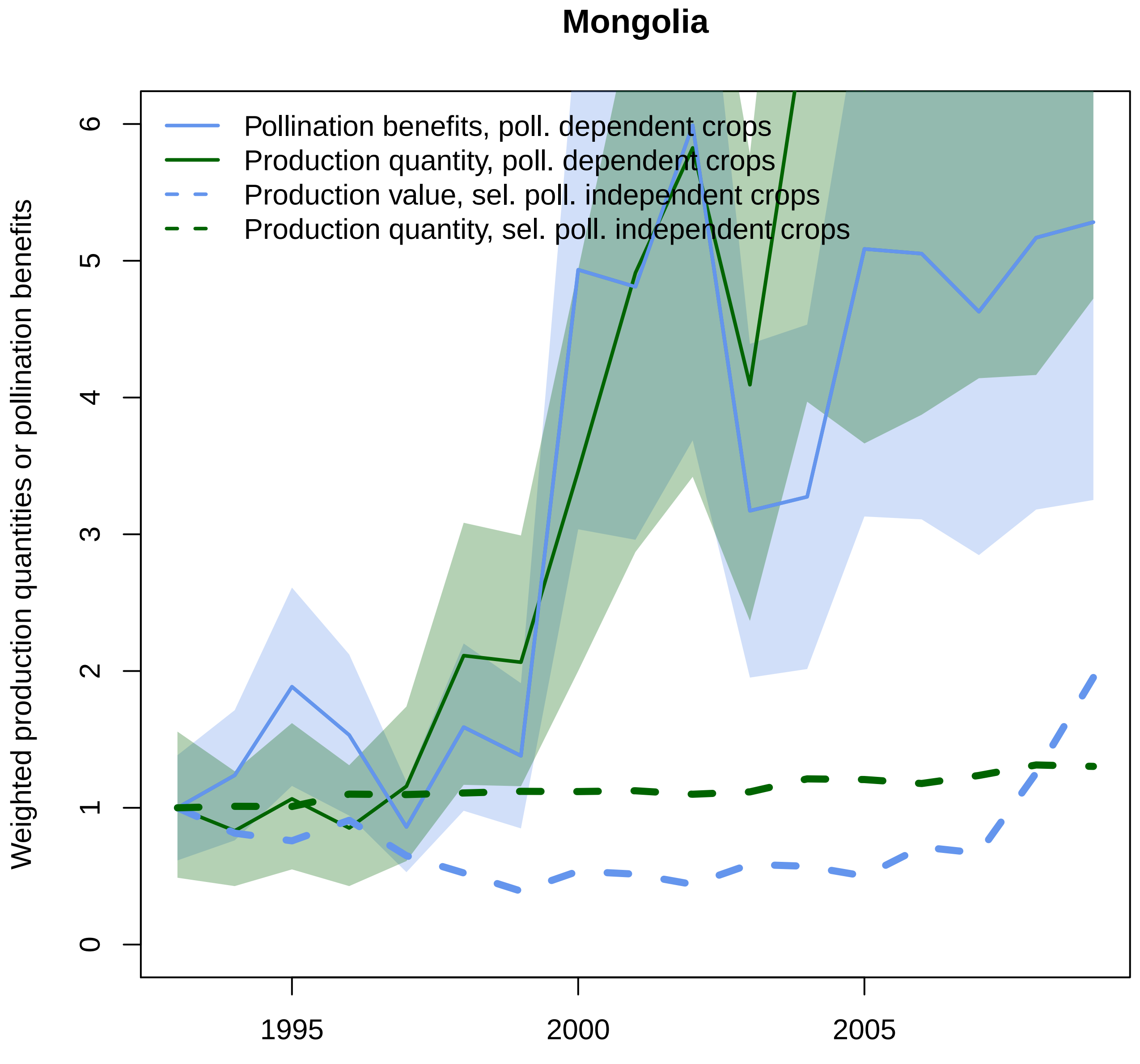
Mongolia
Pollination benefits, poll. dependent crops
Production quantity, poll. dependent crops
Production value, sel. poll. independent crops
Production quantity, sel. poll. independent crops
Weighted production quantities or pollination benefits
0
1
2
3
4
5
6
1995
2000
2005

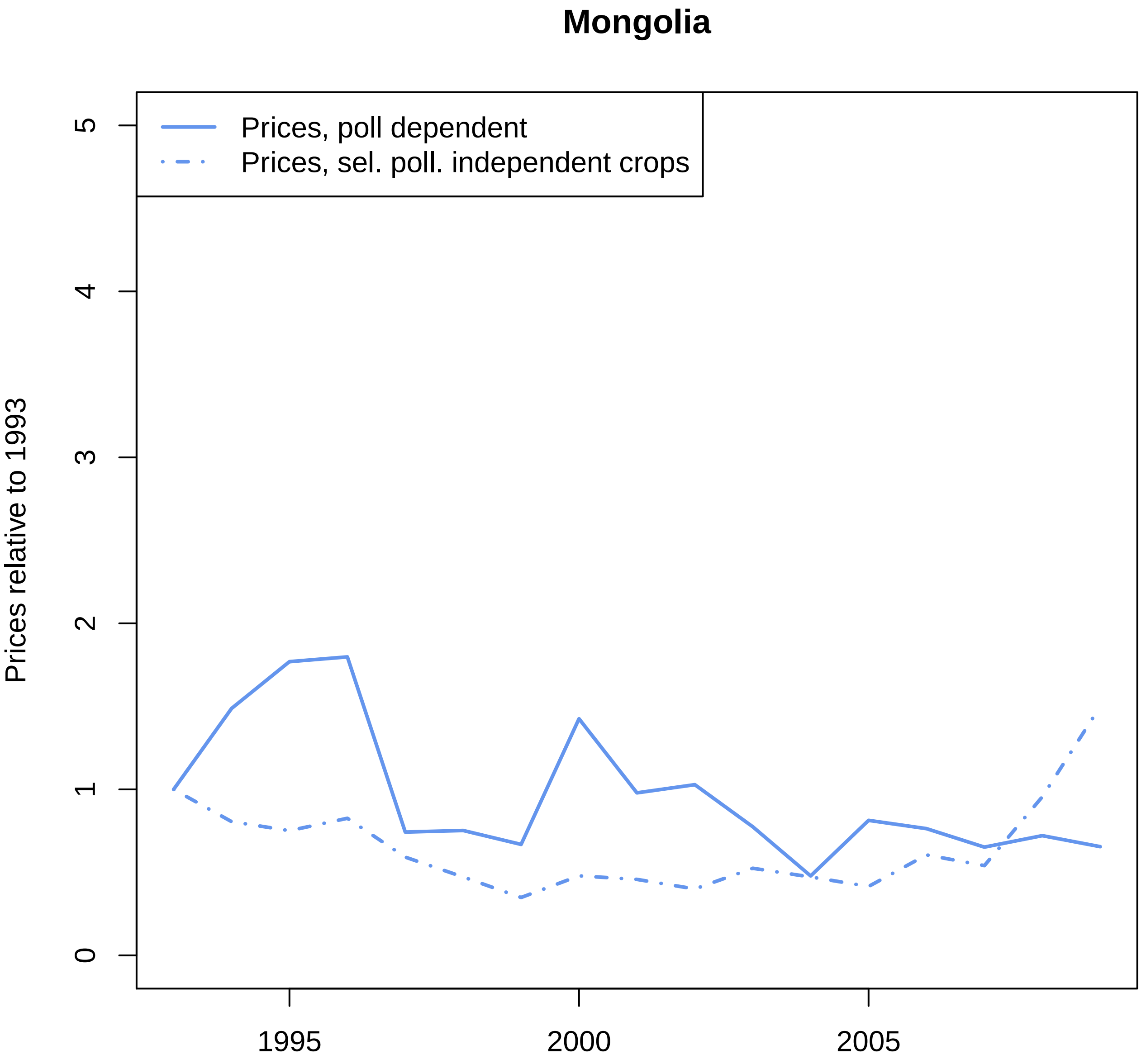

Mongolia
Prices, poll dependent
Prices, sel. poll. independent crops
Prices relative to 1993
0
1
2
3
4
5
1995
2000
2005

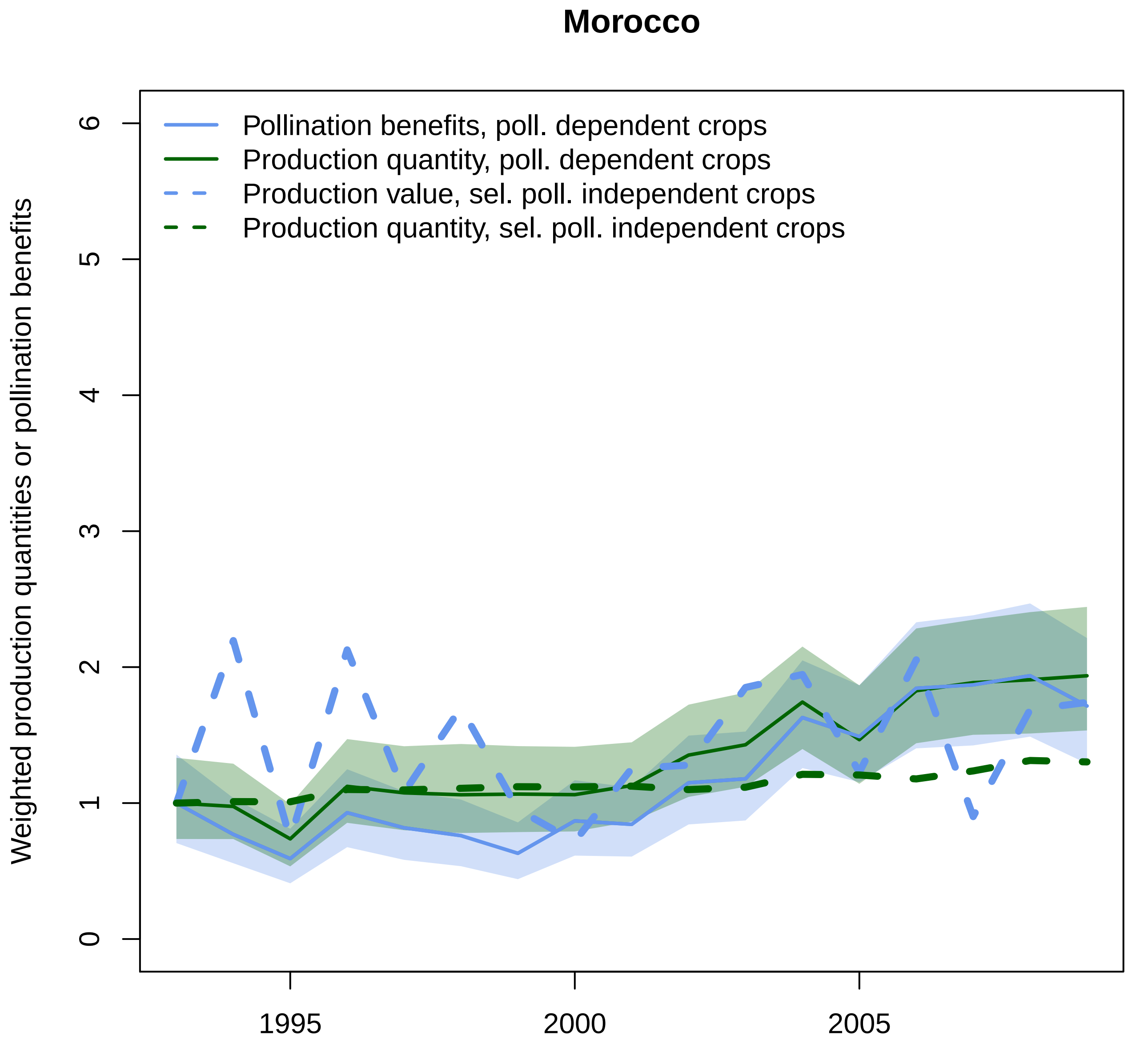

Morocco
Pollination benefits, poll. dependent crops
Production quantity, poll. dependent crops
Production value, sel. poll. independent crops
Production quantity, sel. poll. independent crops
Weighted production quantities or pollination benefits
0
1
2
3
4
5
6
1995
2000
2005

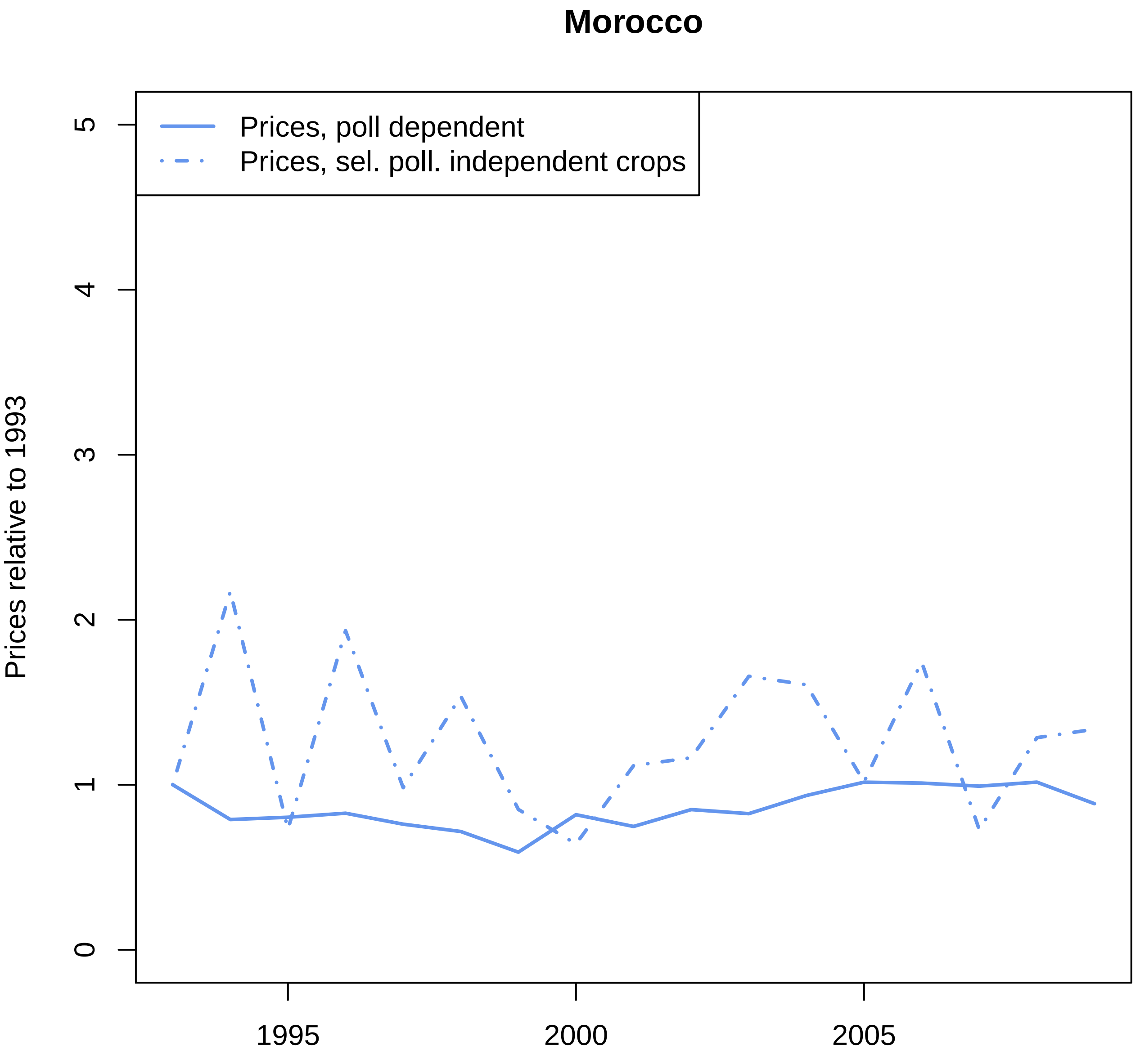

Morocco
Prices, poll dependent
Prices, sel. poll. independent crops
Prices relative to 1993
0
1
2
3
4
5
1995
2000
2005

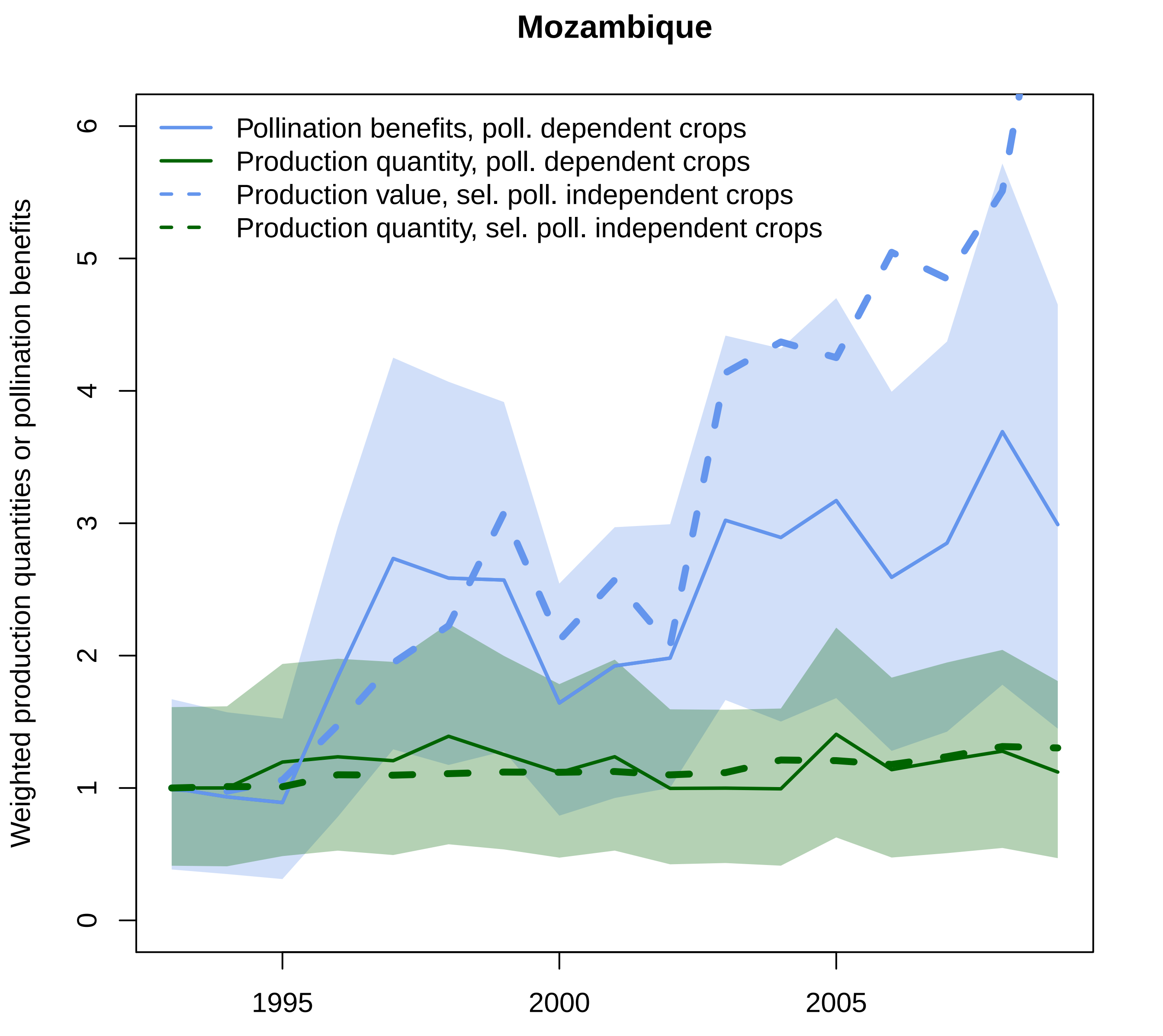

Mozambique
Pollination benefits, poll. dependent crops
Production quantity, poll. dependent crops
Production value, sel. poll. independent crops
Production quantity, sel. poll. independent crops
Weighted production quantities or pollination benefits
0
1
2
3
4
5
6
1995
2000
2005

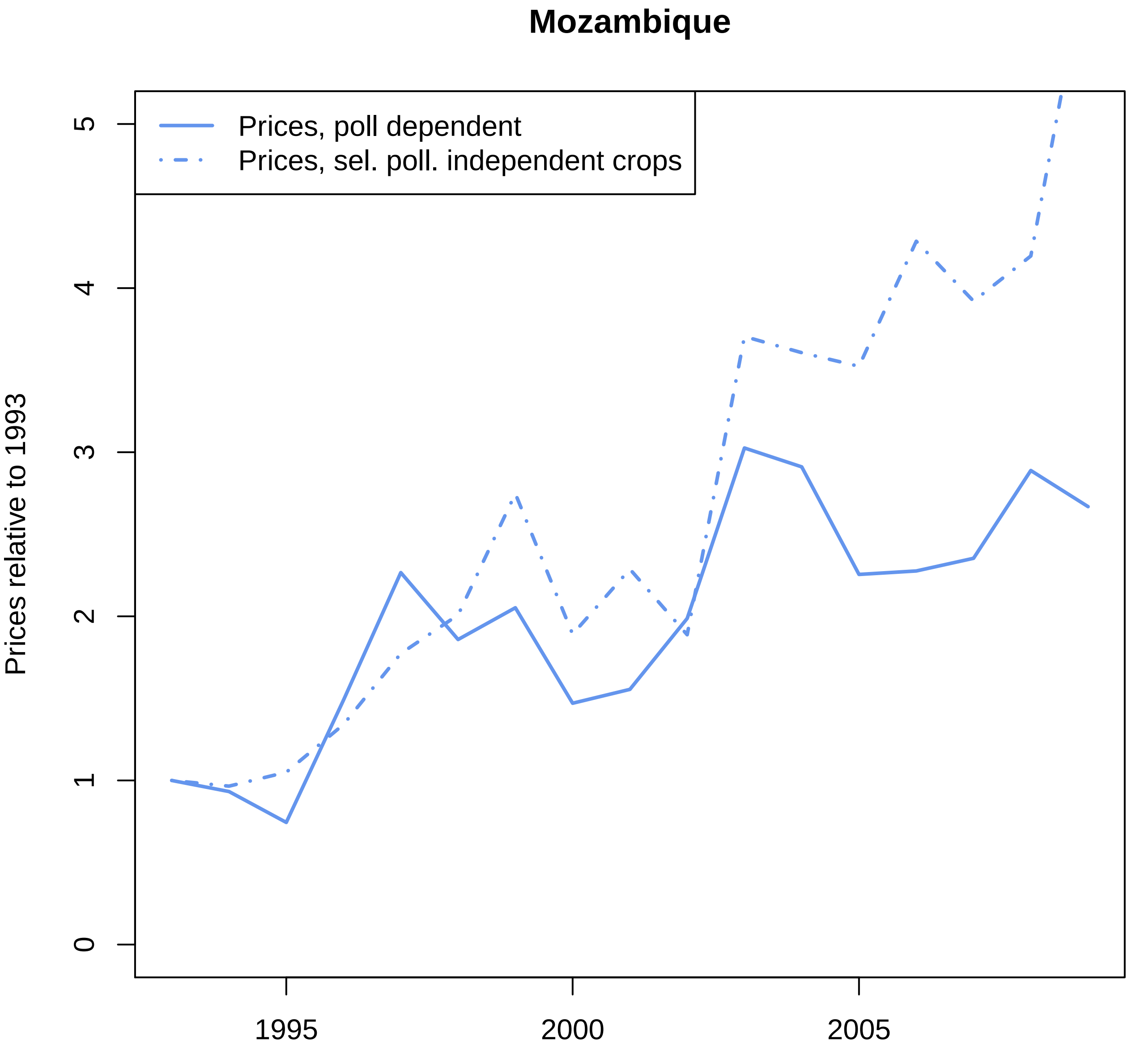

Mozambique
Prices, poll dependent
Prices, sel. poll. independent crops
Prices relative to 1993
0
1
2
3
4
5
1995
2000
2005

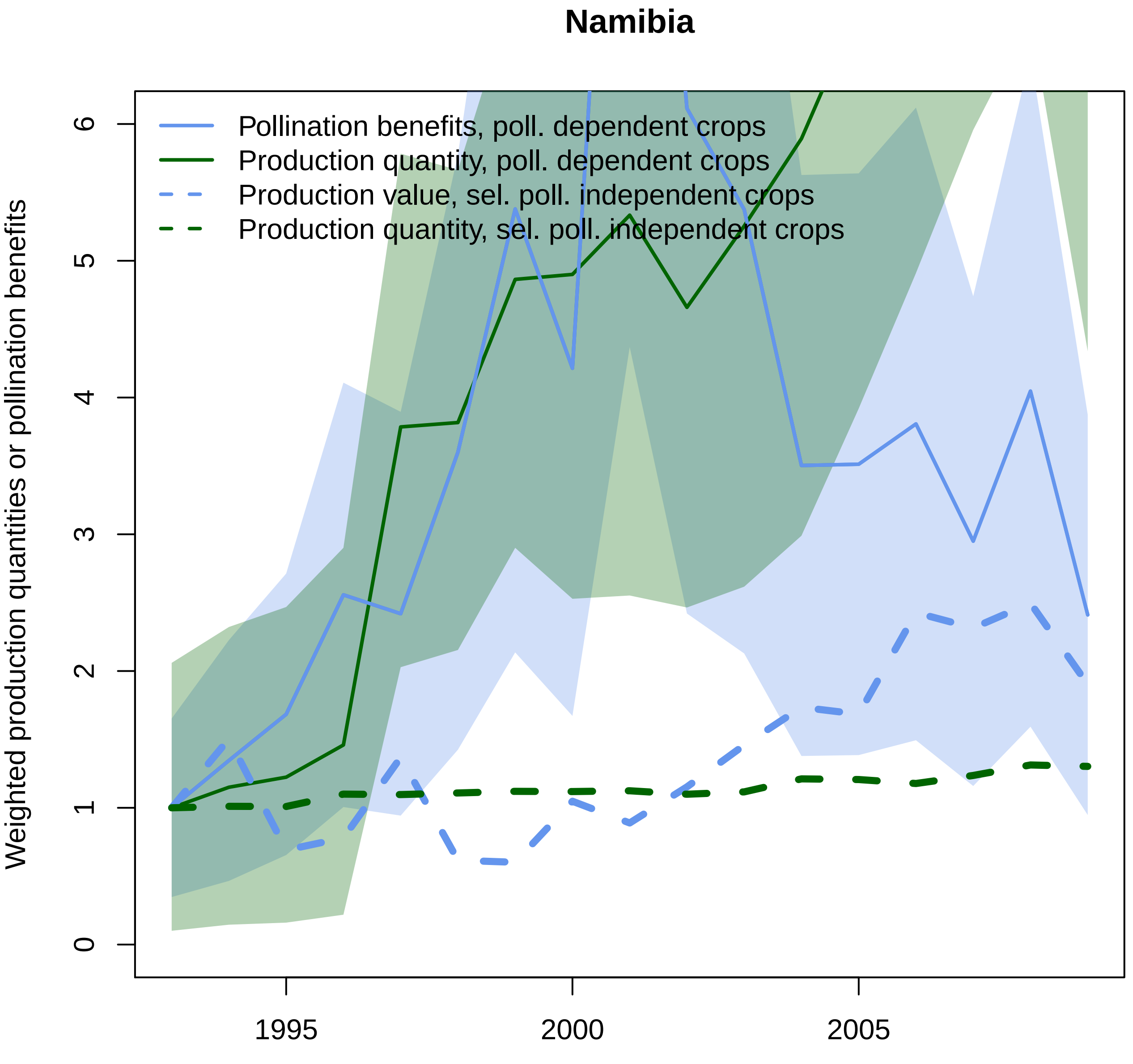
Namibia
Pollination benefits, poll. dependent crops
Production quantity, poll. dependent crops
Production value, sel. poll. independent crops
Production quantity, sel. poll. independent crops
Weighted production quantities or pollination benefits
0
1
2
3
4
5
6
1995
2000
2005

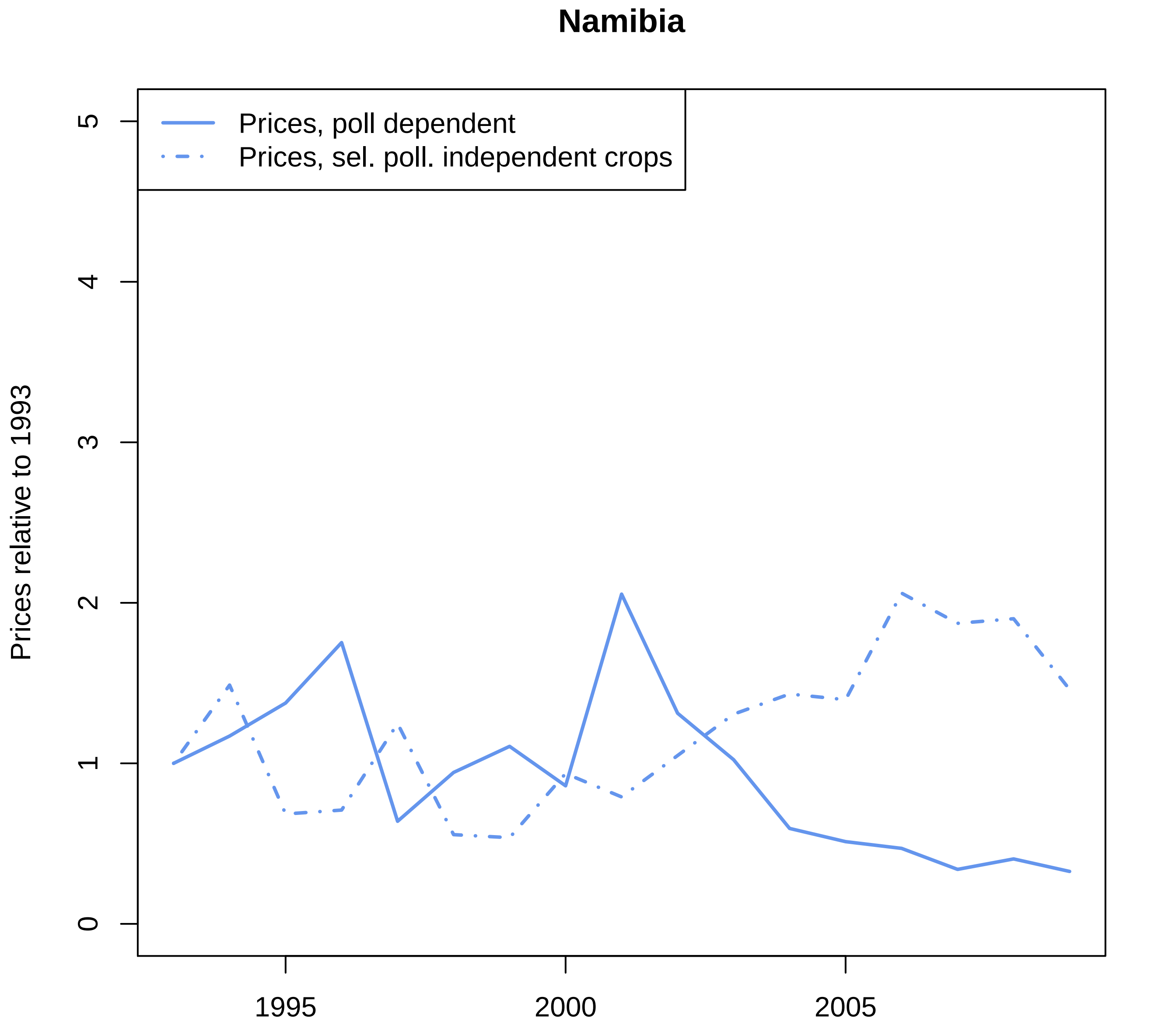

Namibia
Prices, poll dependent
Prices, sel. poll. independent crops
Prices relative to 1993
0
1
2
3
4
5
1995
2000
2005

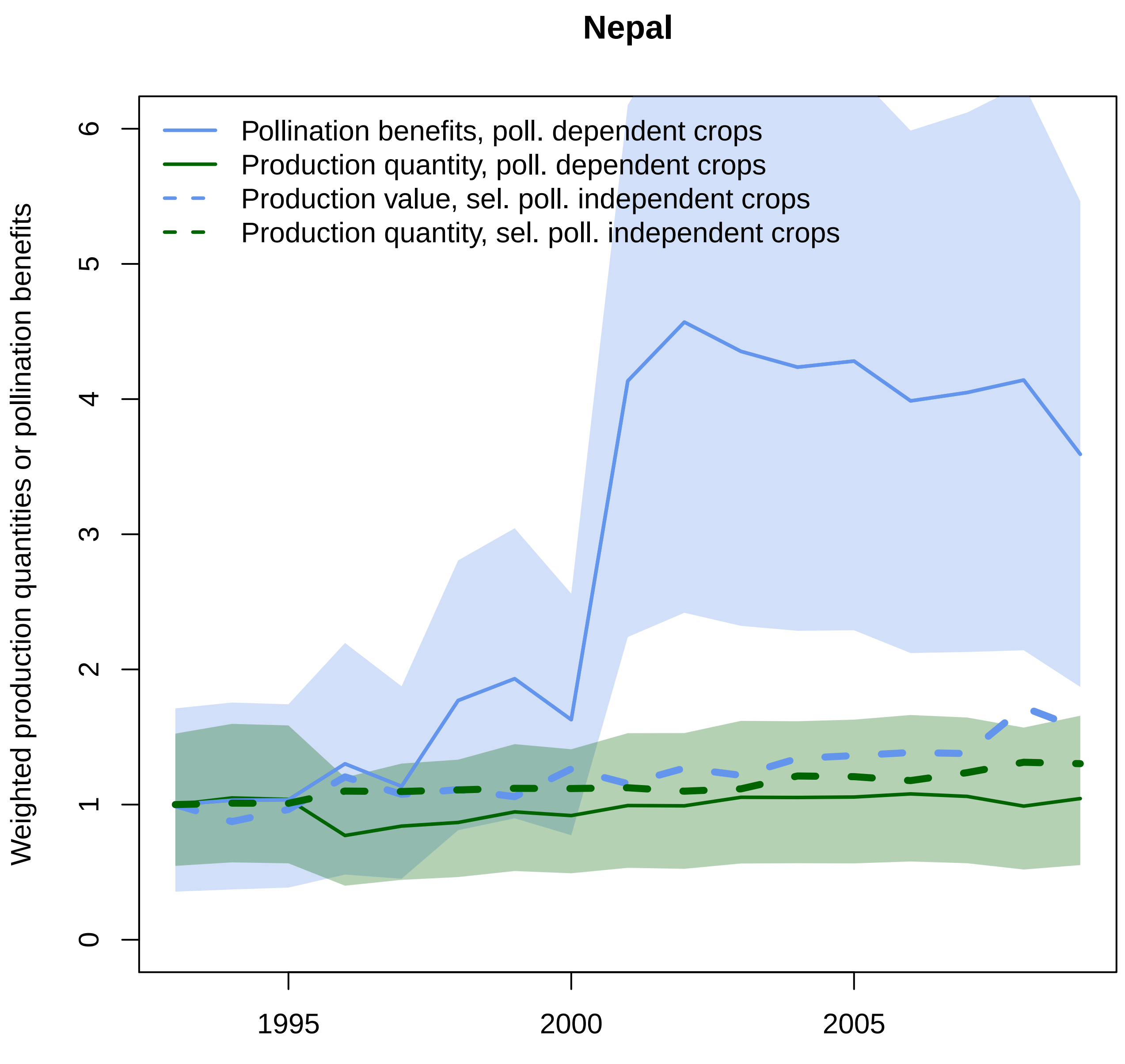
Nepal
Pollination benefits, poll. dependent crops
Production quantity, poll. dependent crops
Production value, sel. poll. independent crops
Production quantity, sel. poll. independent crops
Weighted production quantities or pollination benefits
0
1
2
3
4
5
6
1995
2000
2005

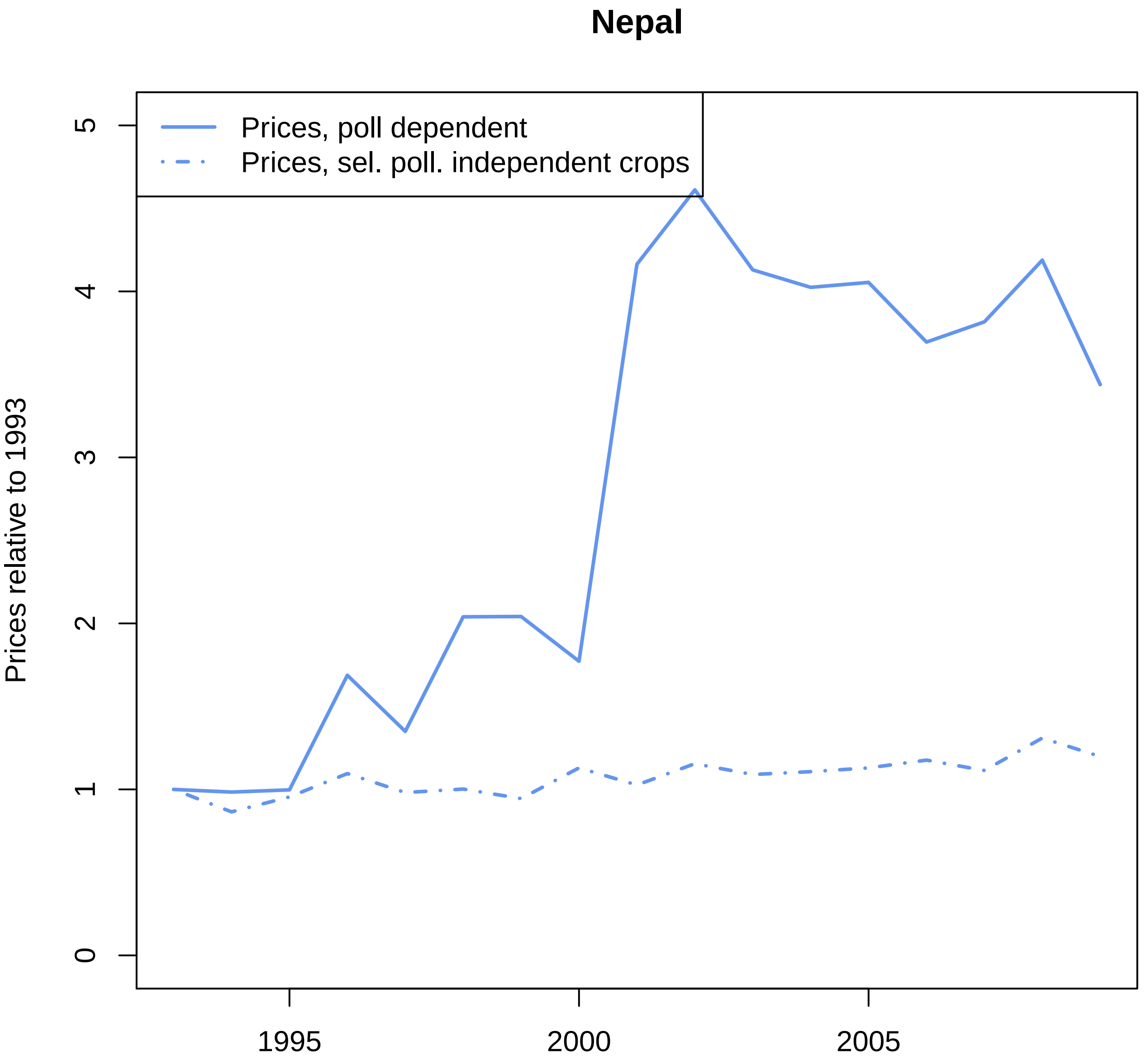

Nepal
Prices, poll dependent
Prices, sel. poll. independent crops
Prices relative to 1993
0
1
2
3
4
5
1995
2000
2005

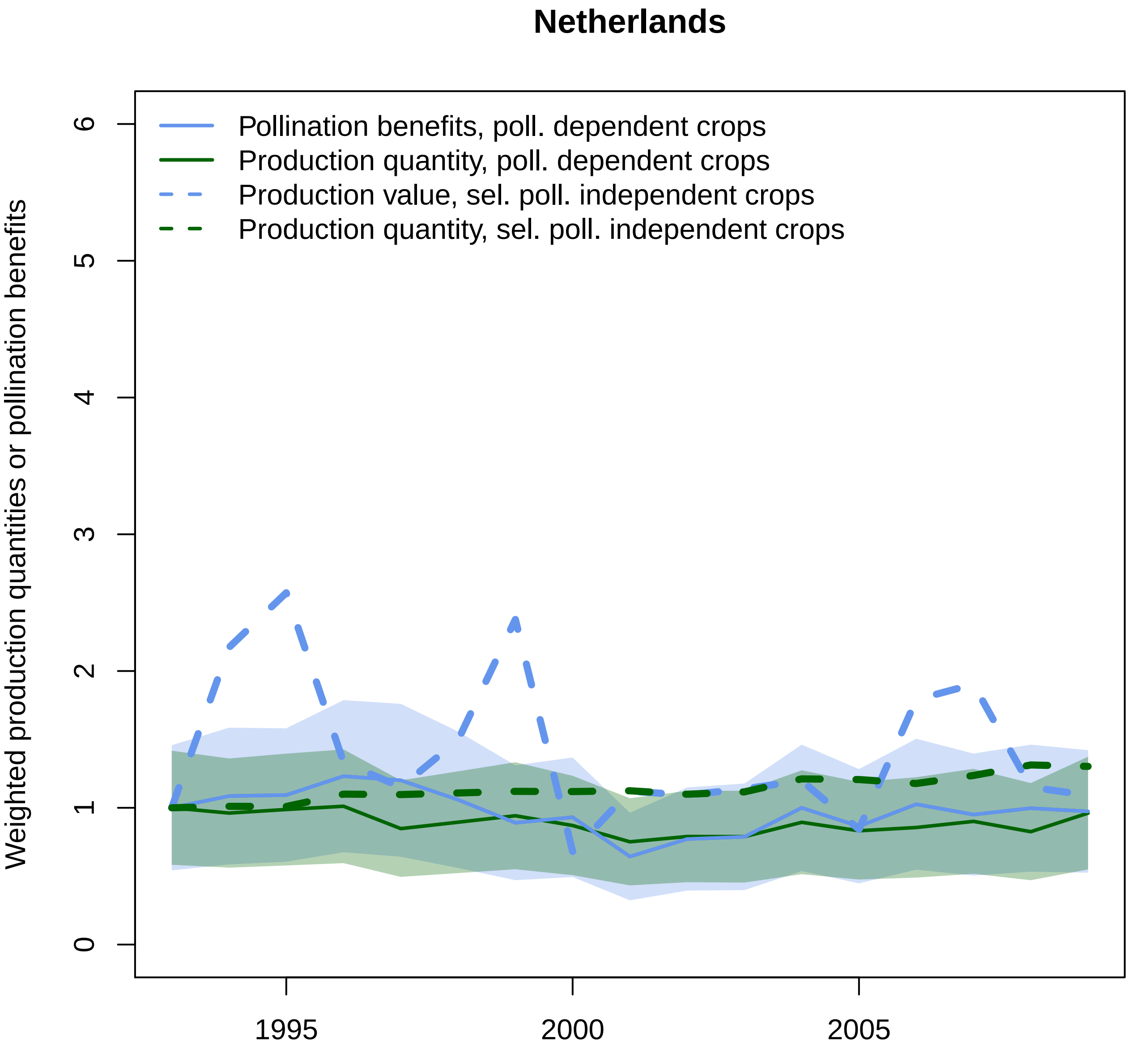

Netherlands
Pollination benefits, poll. dependent crops
Production quantity, poll. dependent crops
Production value, sel. poll. independent crops
Production quantity, sel. poll. independent crops
Weighted production quantities or pollination benefits
0
1
2
3
4
5
6
1995
2000
2005

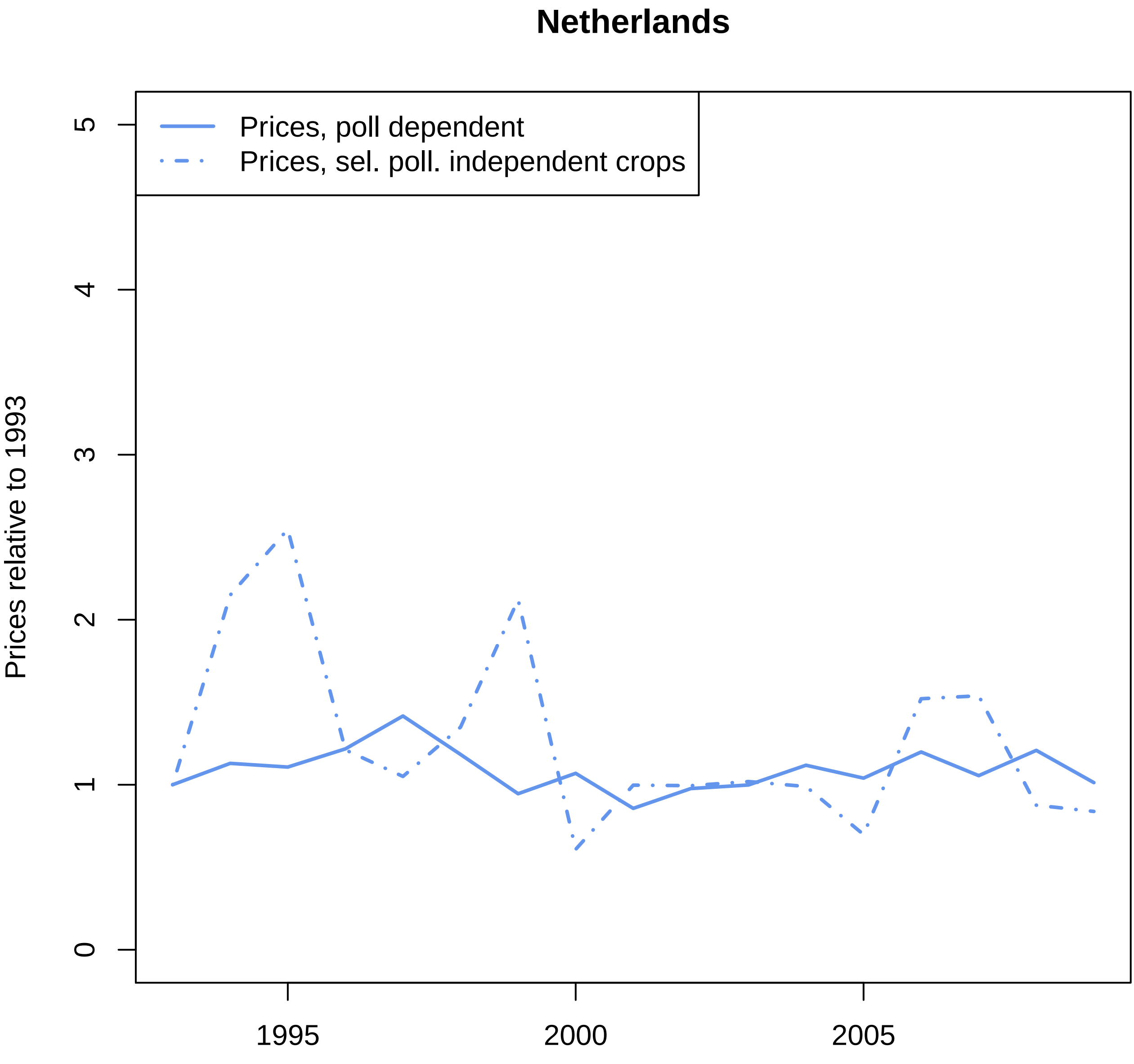

Netherlands
Prices, poll dependent
Prices, sel. poll. independent crops
Prices relative to 1993
0
1
2
3
4
5
1995
2000
2005

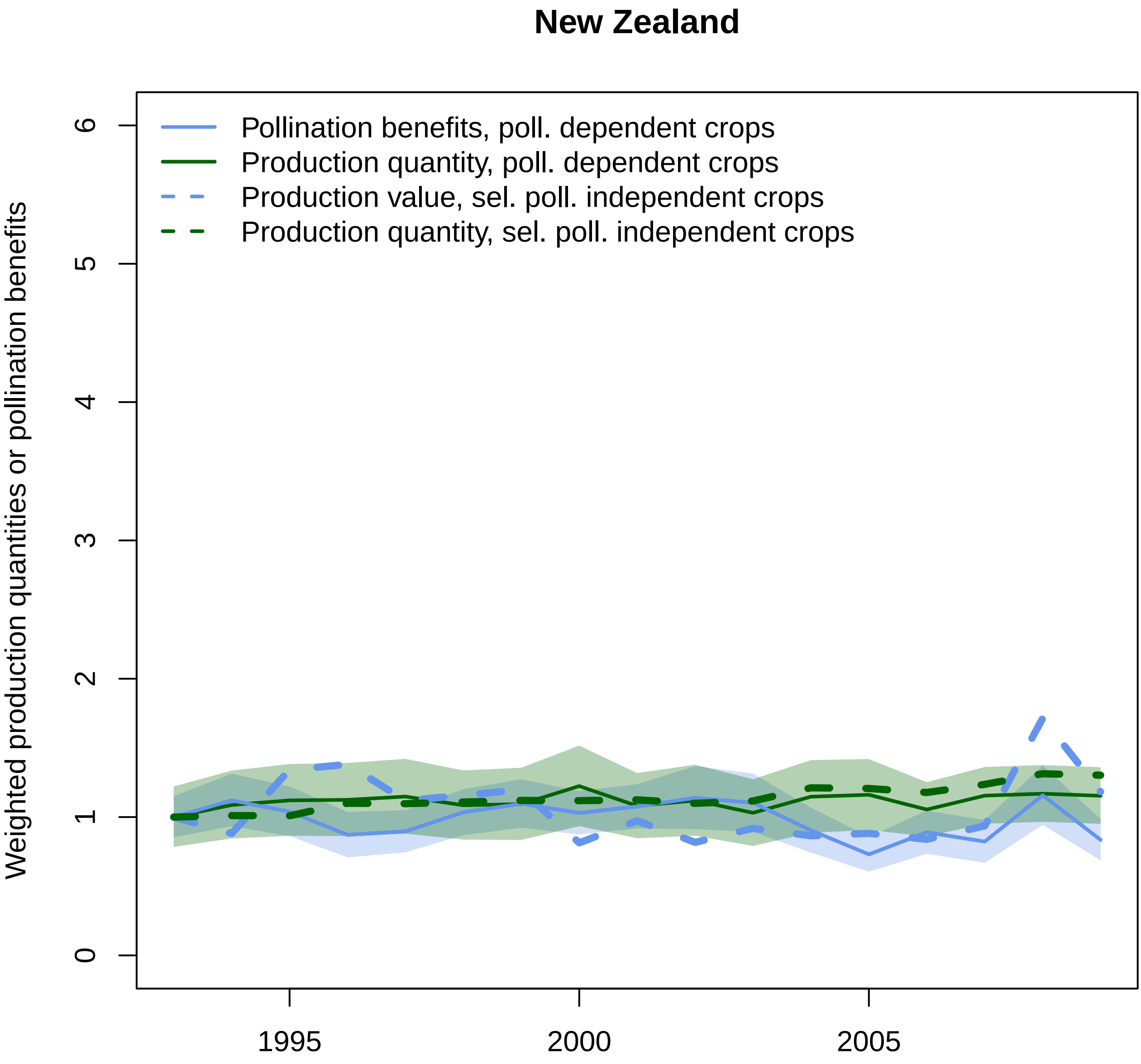
New Zealand
Pollination benefits, poll. dependent crops
Production quantity, poll. dependent crops
Production value, sel. poll. independent crops
Production quantity, sel. poll. independent crops
Weighted production quantities or pollination benefits
0
1
2
3
4
5
6
1995
2000
2005

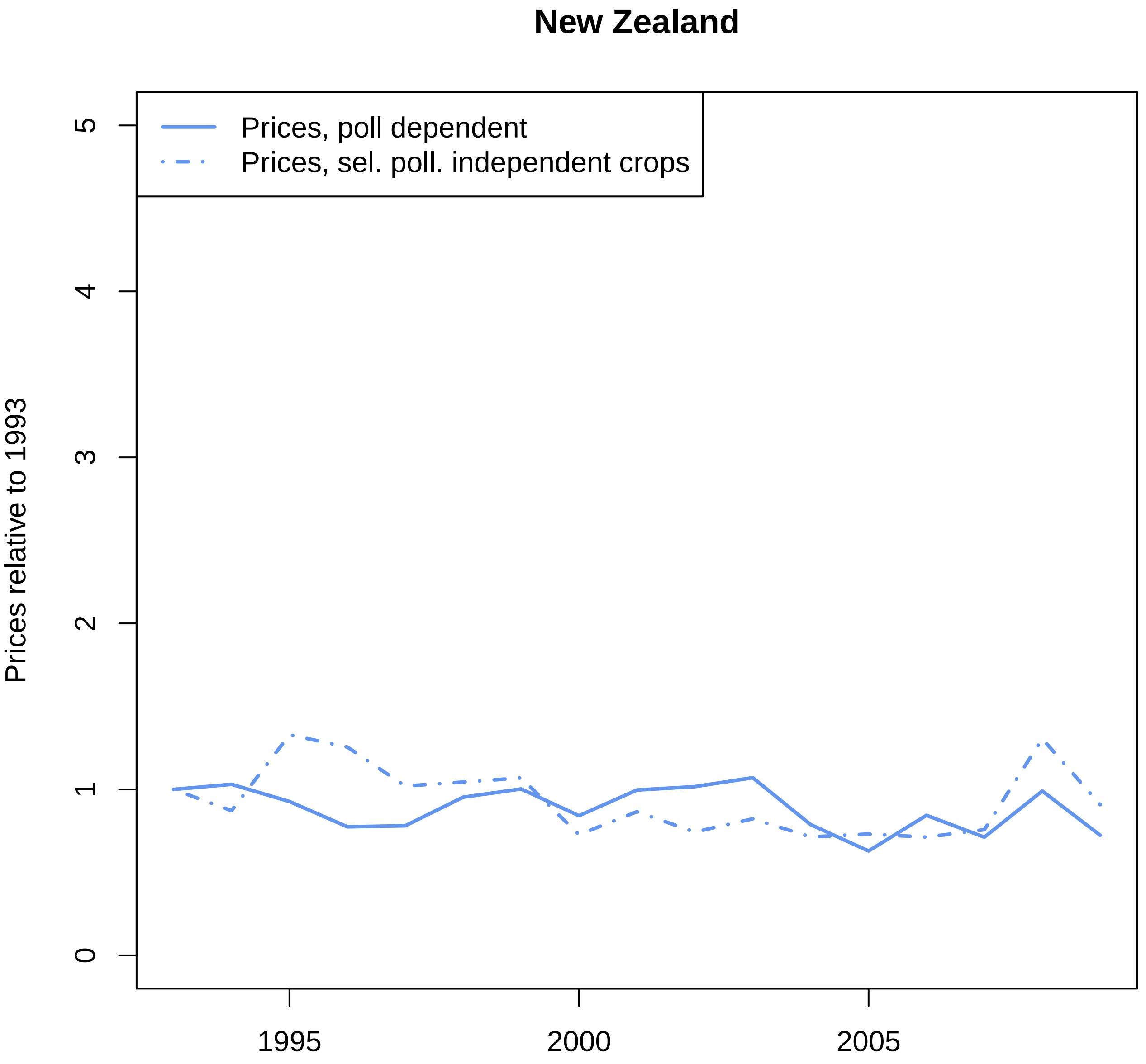
New Zealand
Prices, poll dependent
Prices, sel. poll. independent crops
Prices relative to 1993
0
1
2
3
4
5
1995
2000
2005

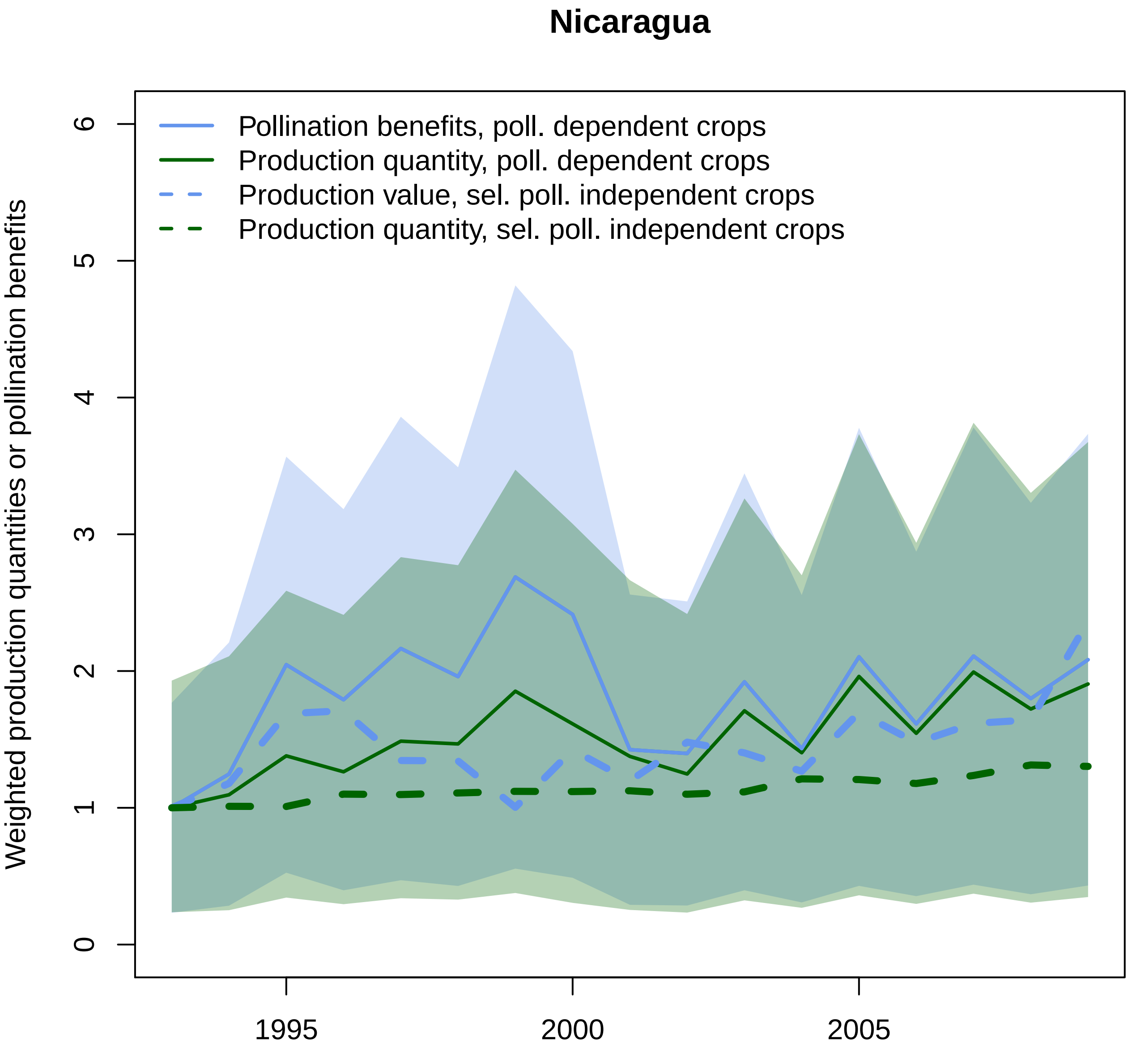

Nicaragua
Pollination benefits, poll. dependent crops
Production quantity, poll. dependent crops
Production value, sel. poll. independent crops
Production quantity, sel. poll. independent crops
Weighted production quantities or pollination benefits
0
1
2
3
4
5
6
1995
2000
2005

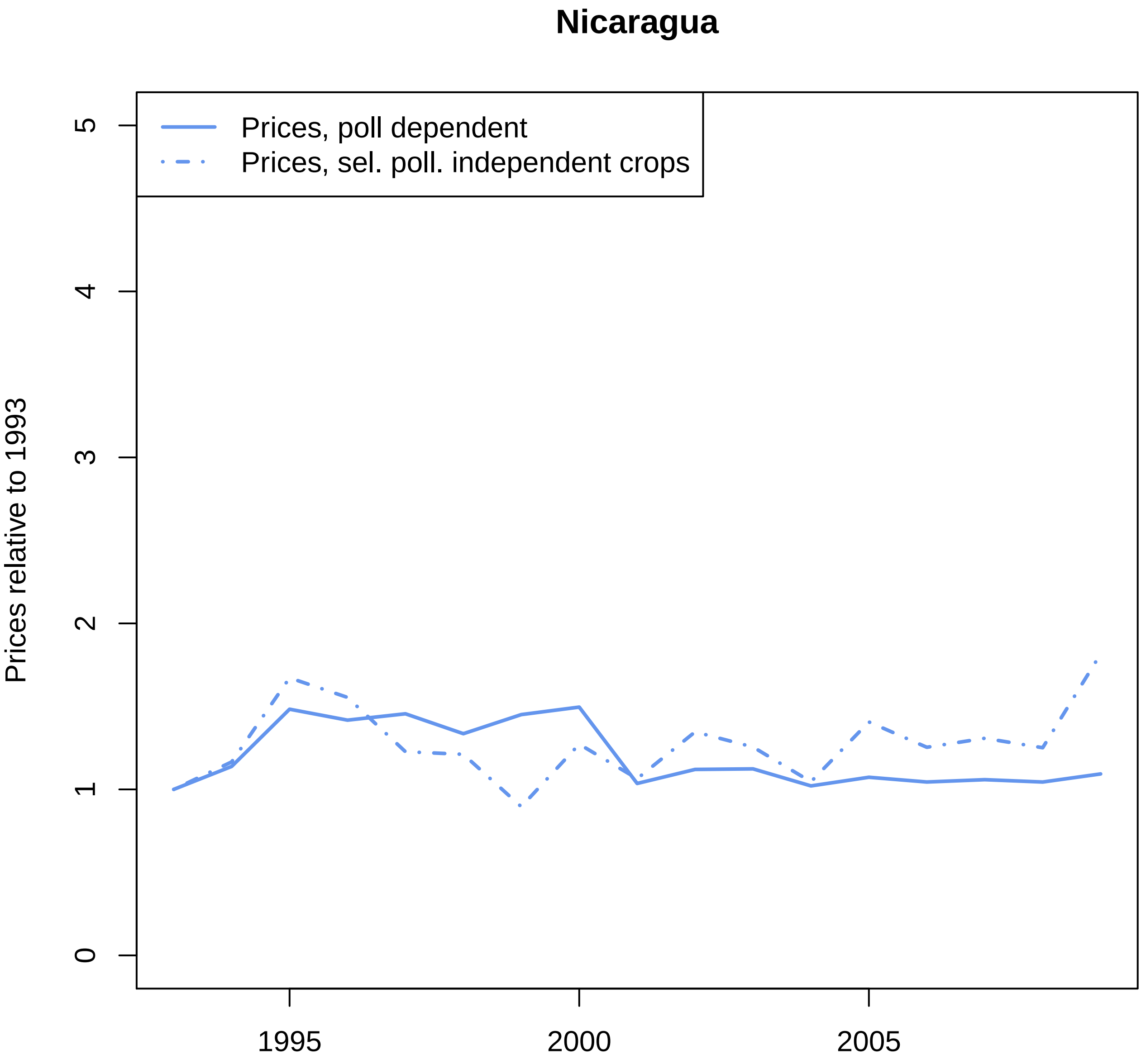

Nicaragua
Prices, poll dependent
Prices, sel. poll. independent crops
Prices relative to 1993
0
1
2
3
4
5
1995
2000
2005

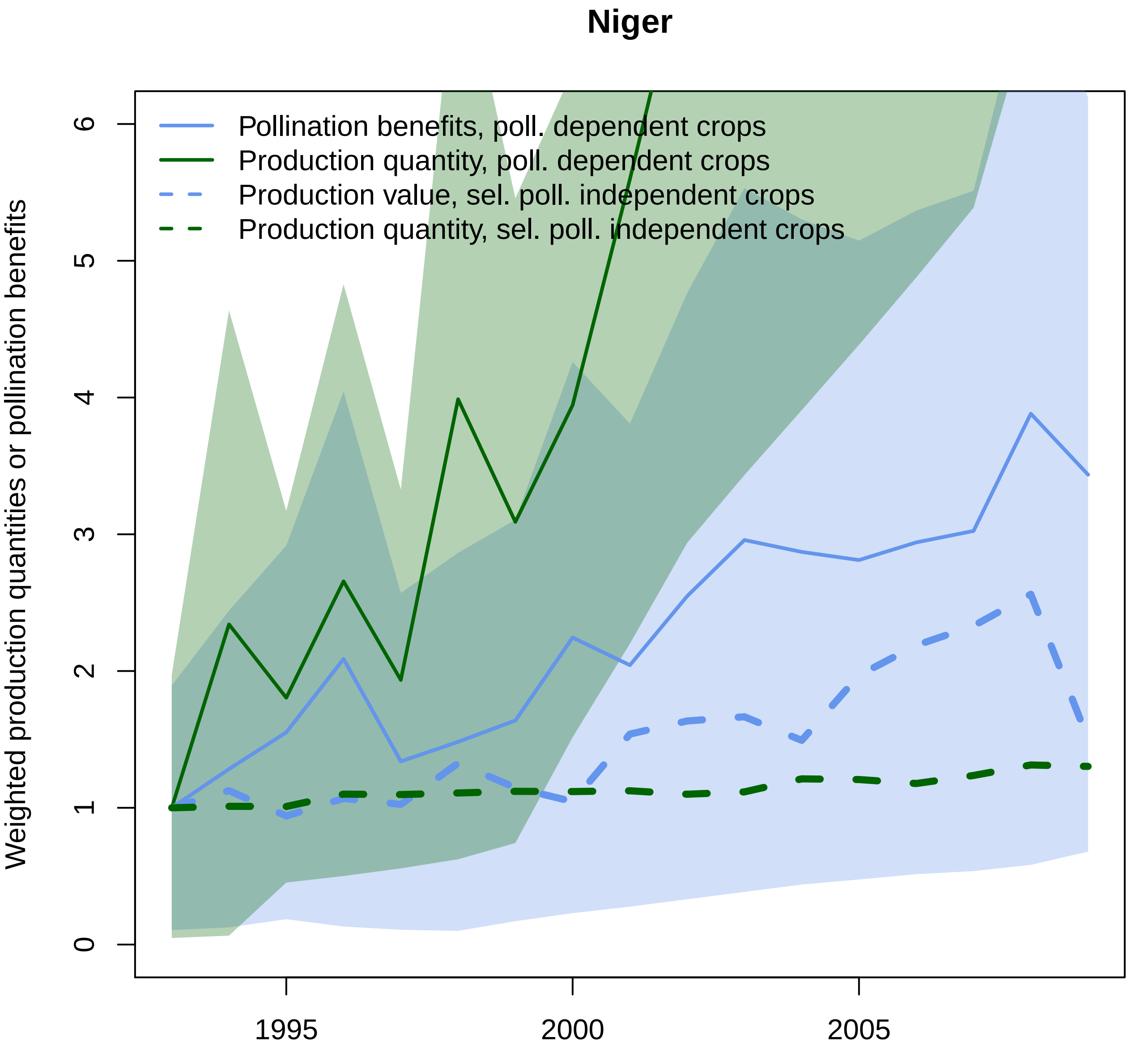

Niger
Pollination benefits, poll. dependent crops
Production quantity, poll. dependent crops
Production value, sel. poll. independent crops
Production quantity, sel. poll. independent crops
Weighted production quantities or pollination benefits
0
1
2
3
4
5
6
1995
2000
2005

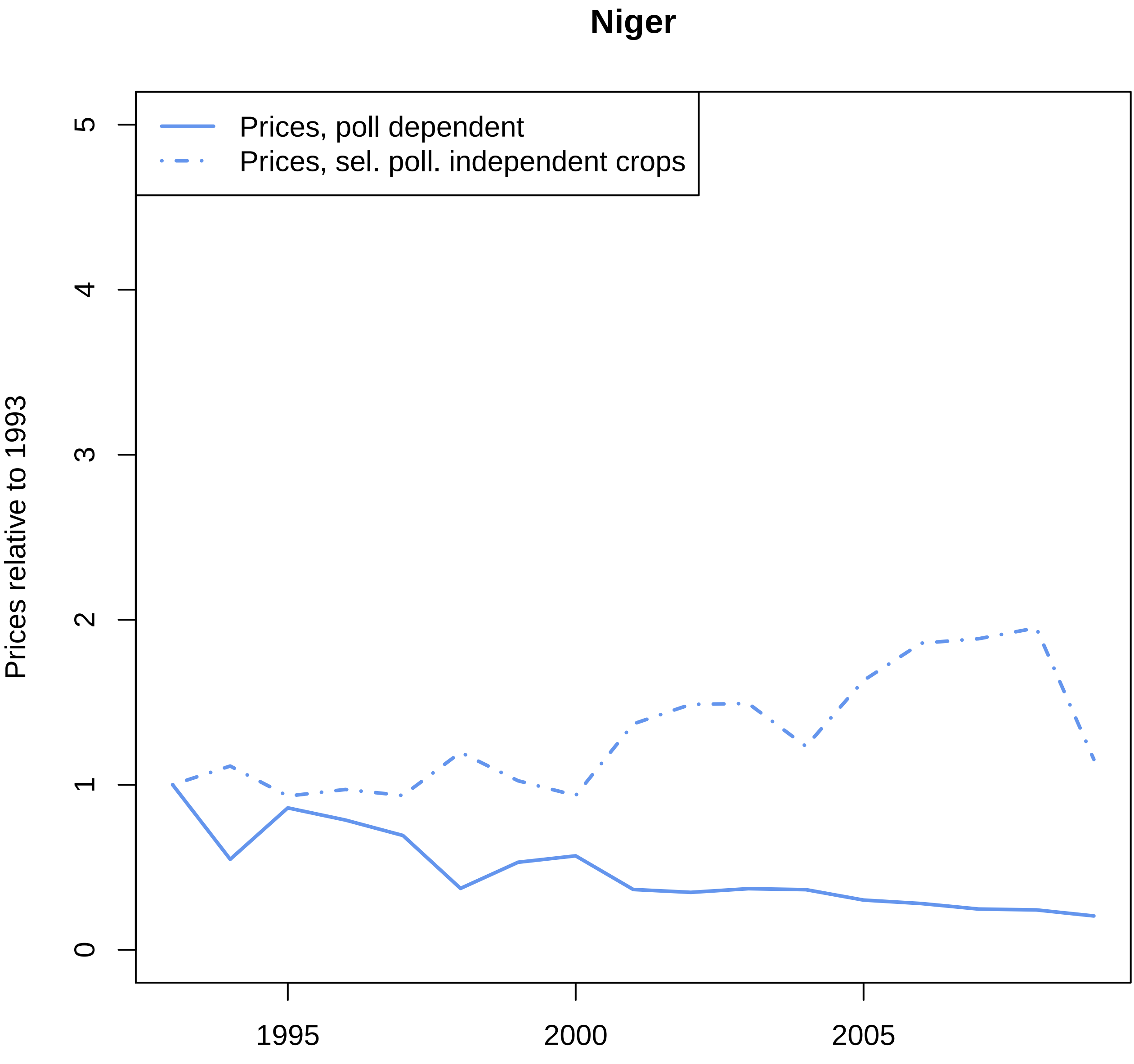

Niger
Prices, poll dependent
Prices, sel. poll. independent crops
Prices relative to 1993
0
1
2
3
4
5
1995
2000
2005

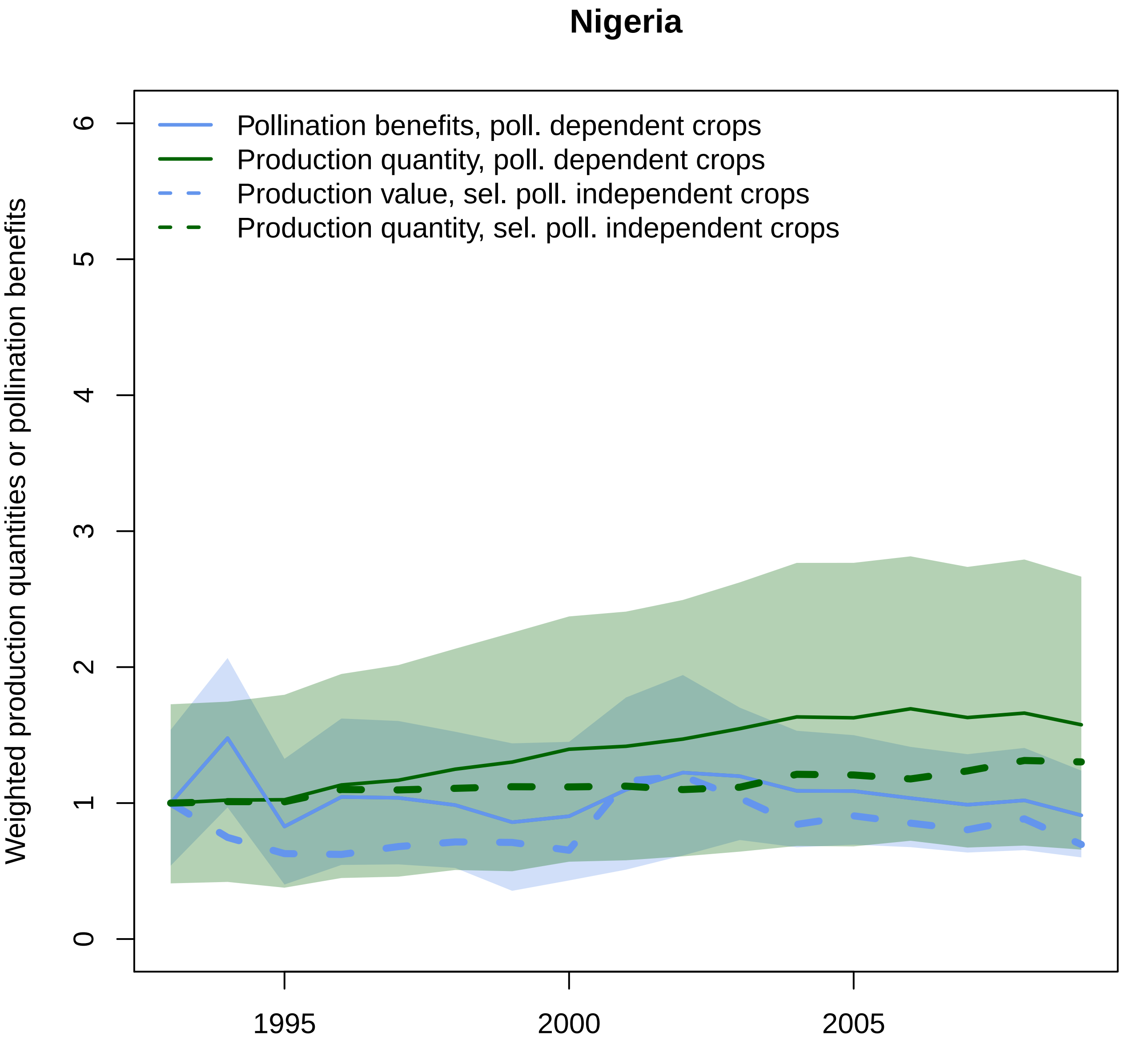

Nigeria
Pollination benefits, poll. dependent crops
Production quantity, poll. dependent crops
Production value, sel. poll. independent crops
Production quantity, sel. poll. independent crops
Weighted production quantities or pollination benefits
0
1
2
3
4
5
6
1995
2000
2005

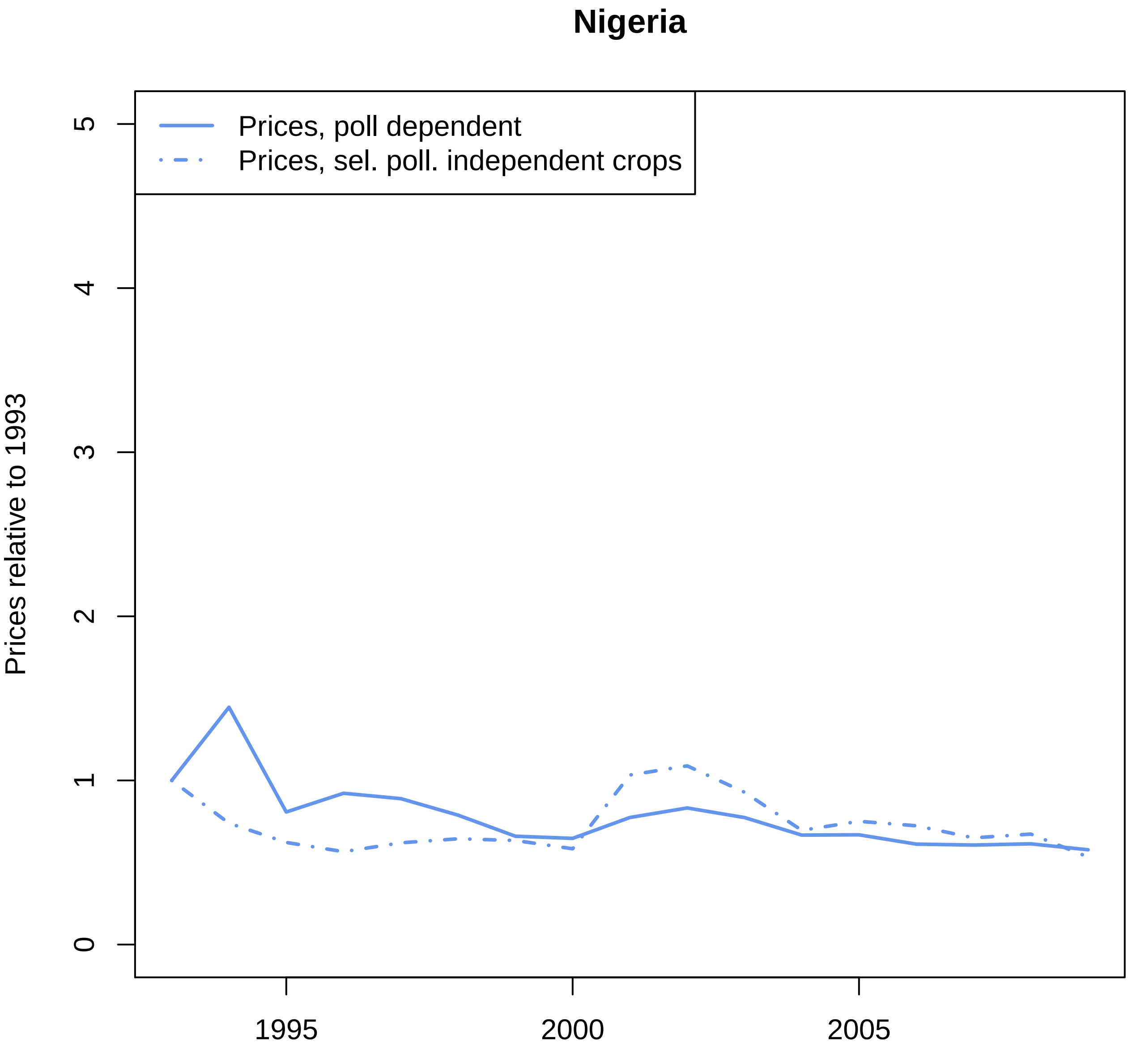
Nigeria
Prices, poll dependent
Prices, sel. poll. independent crops
Prices relative to 1993
0
1
2
3
4
5
1995
2000
2005

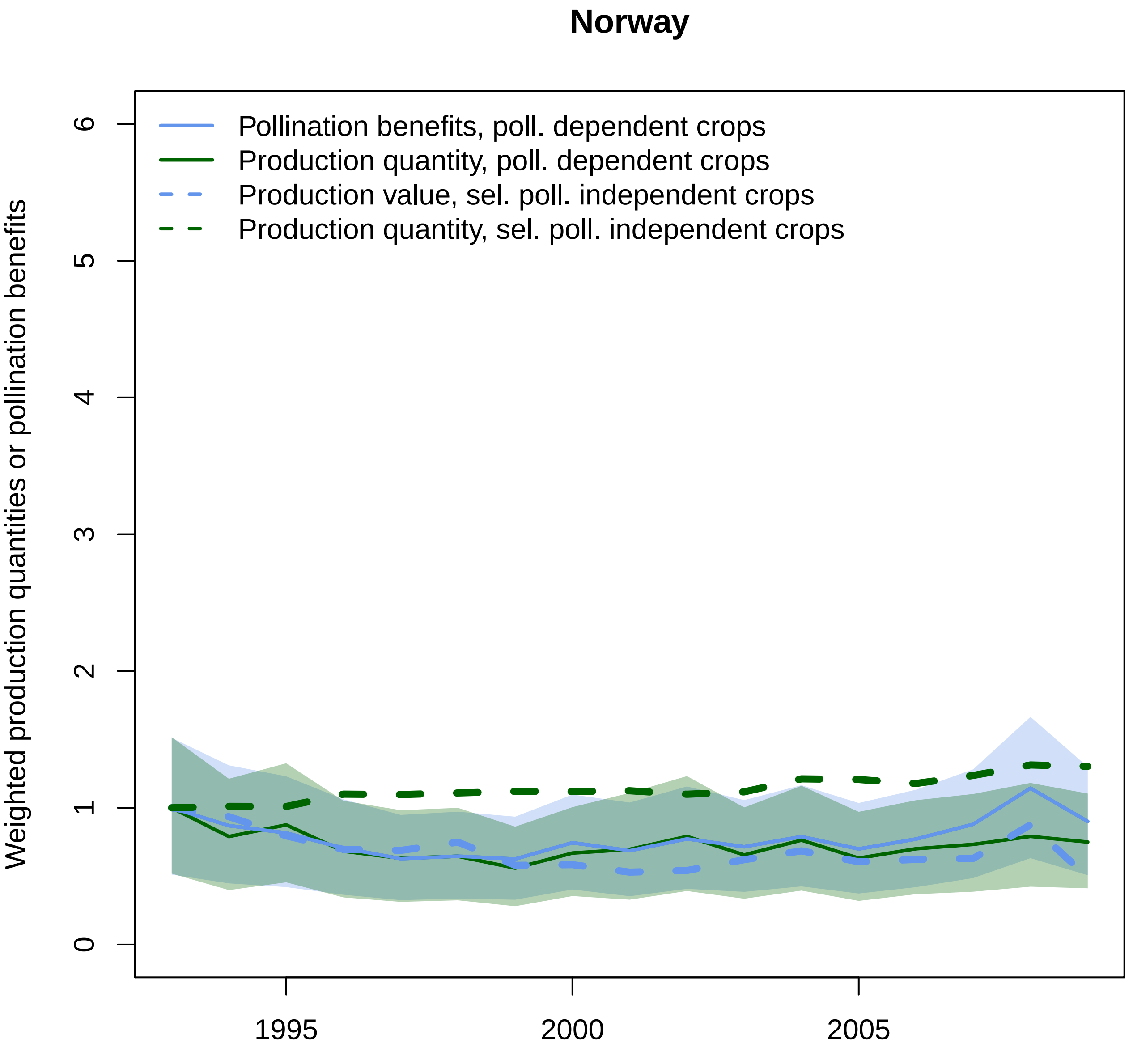

Norway
Pollination benefits, poll. dependent crops
Production quantity, poll. dependent crops
Production value, sel. poll. independent crops
Production quantity, sel. poll. independent crops
Weighted production quantities or pollination benefits
0
1
2
3
4
5
6
1995
2000
2005

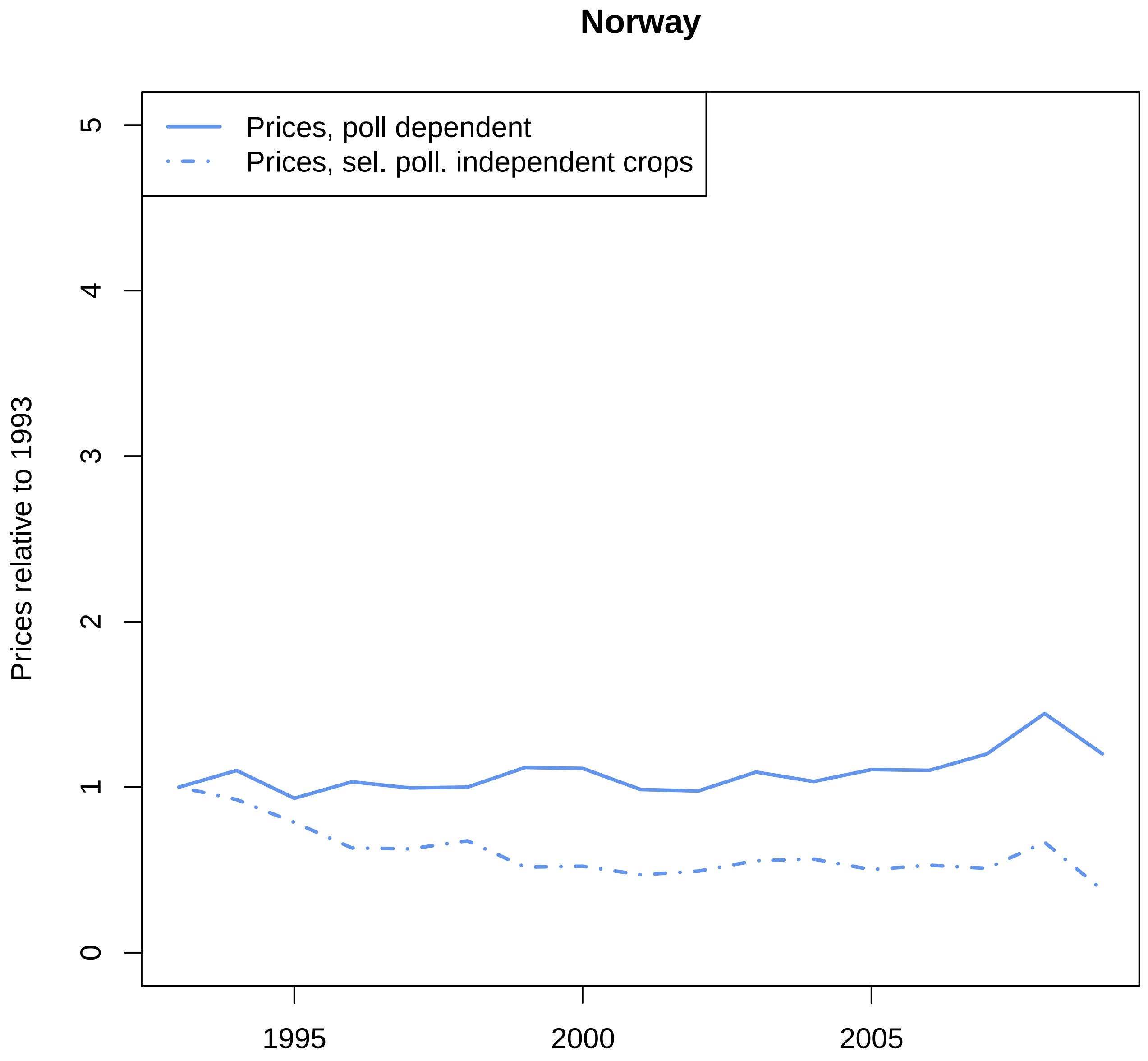

Norway
Prices, poll dependent
Prices, sel. poll. independent crops
Prices relative to 1993
0
1
2
3
4
5
1995
2000
2005

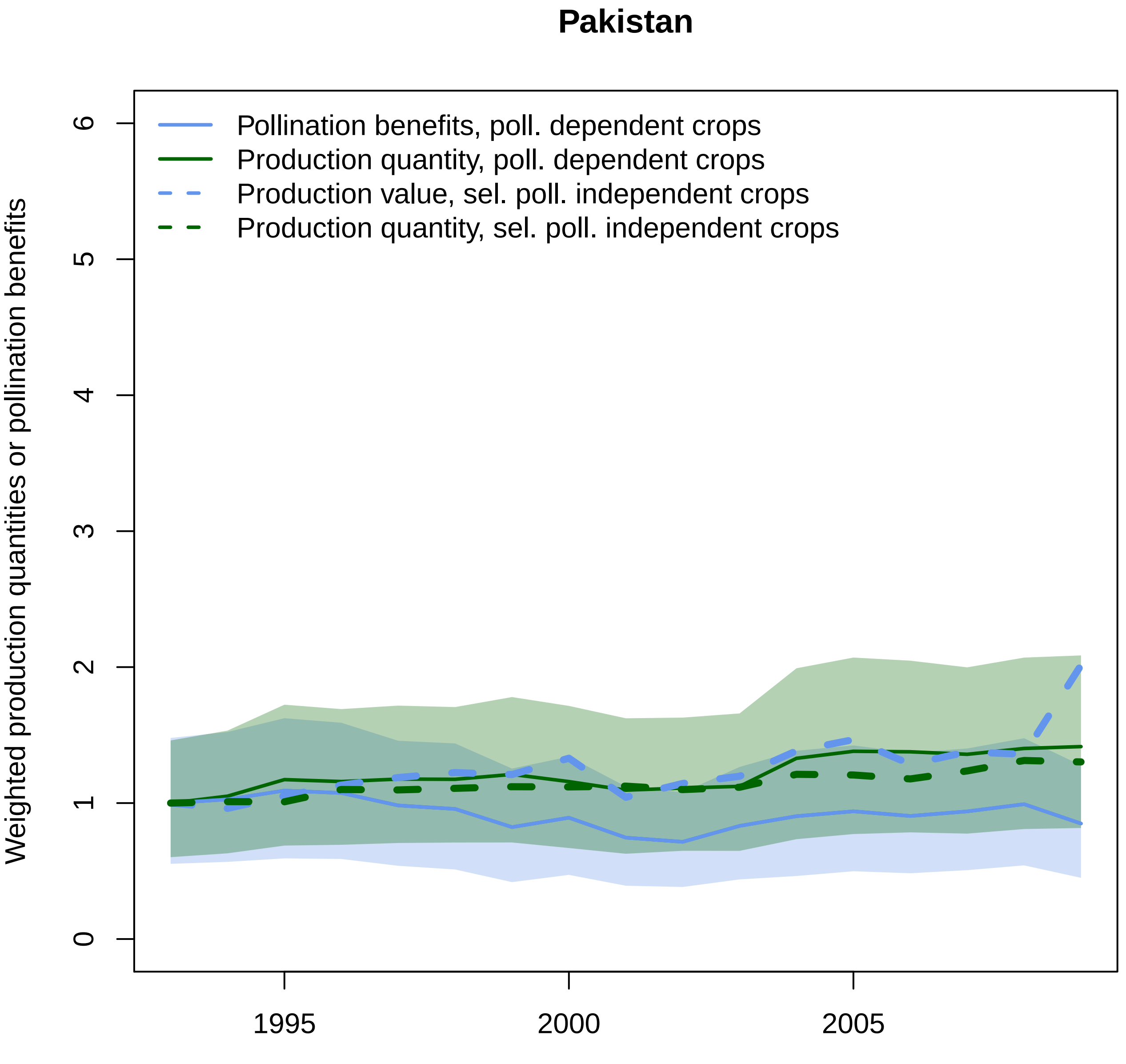
Pakistan
Pollination benefits, poll. dependent crops
Production quantity, poll. dependent crops
Production value, sel. poll. independent crops
Production quantity, sel. poll. independent crops
Weighted production quantities or pollination benefits
0
1
2
3
4
5
6
1995
2000
2005

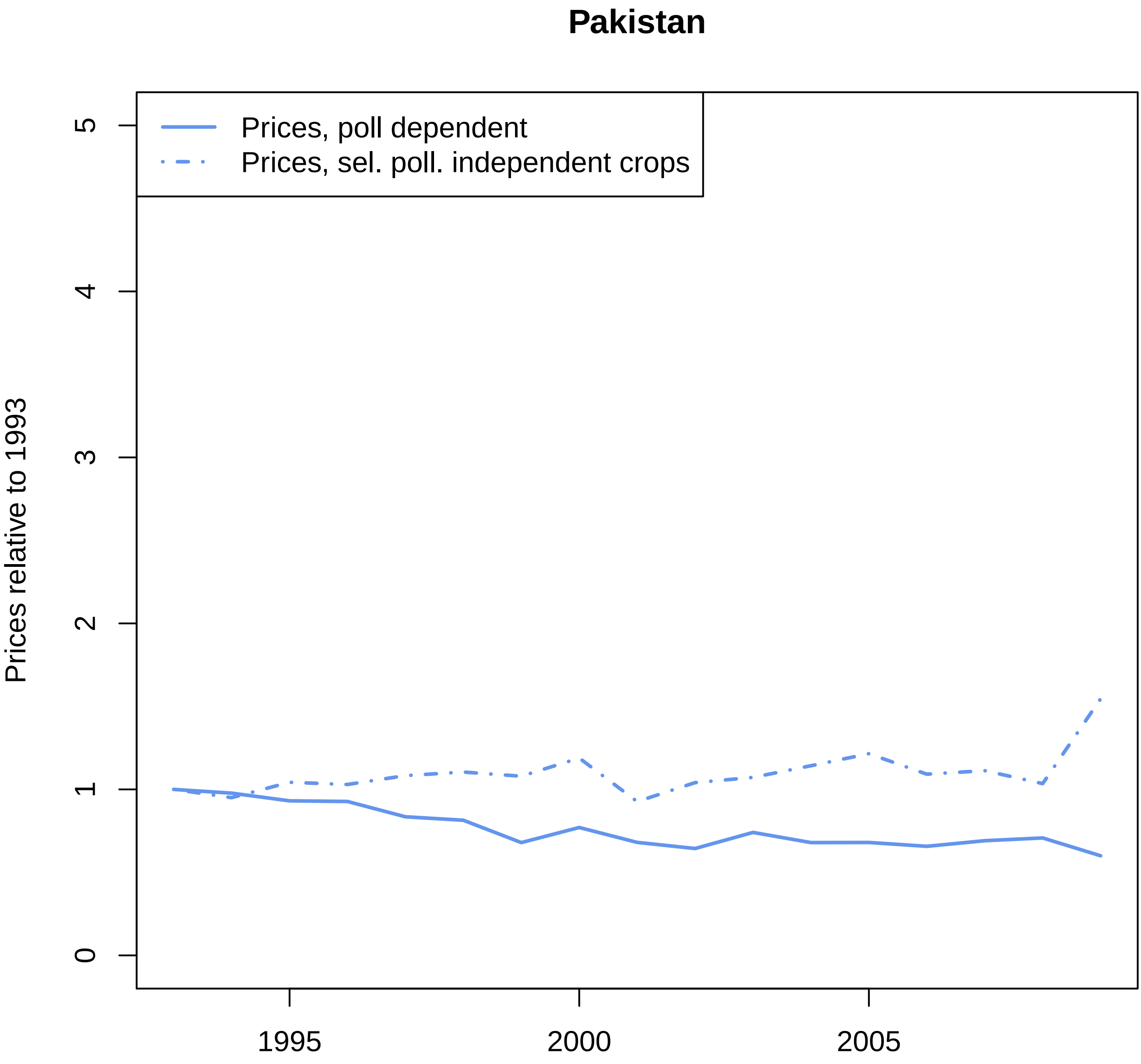
Pakistan
Prices, poll dependent
Prices, sel. poll. independent crops
Prices relative to 1993
0
1
2
3
4
5
1995
2000
2005

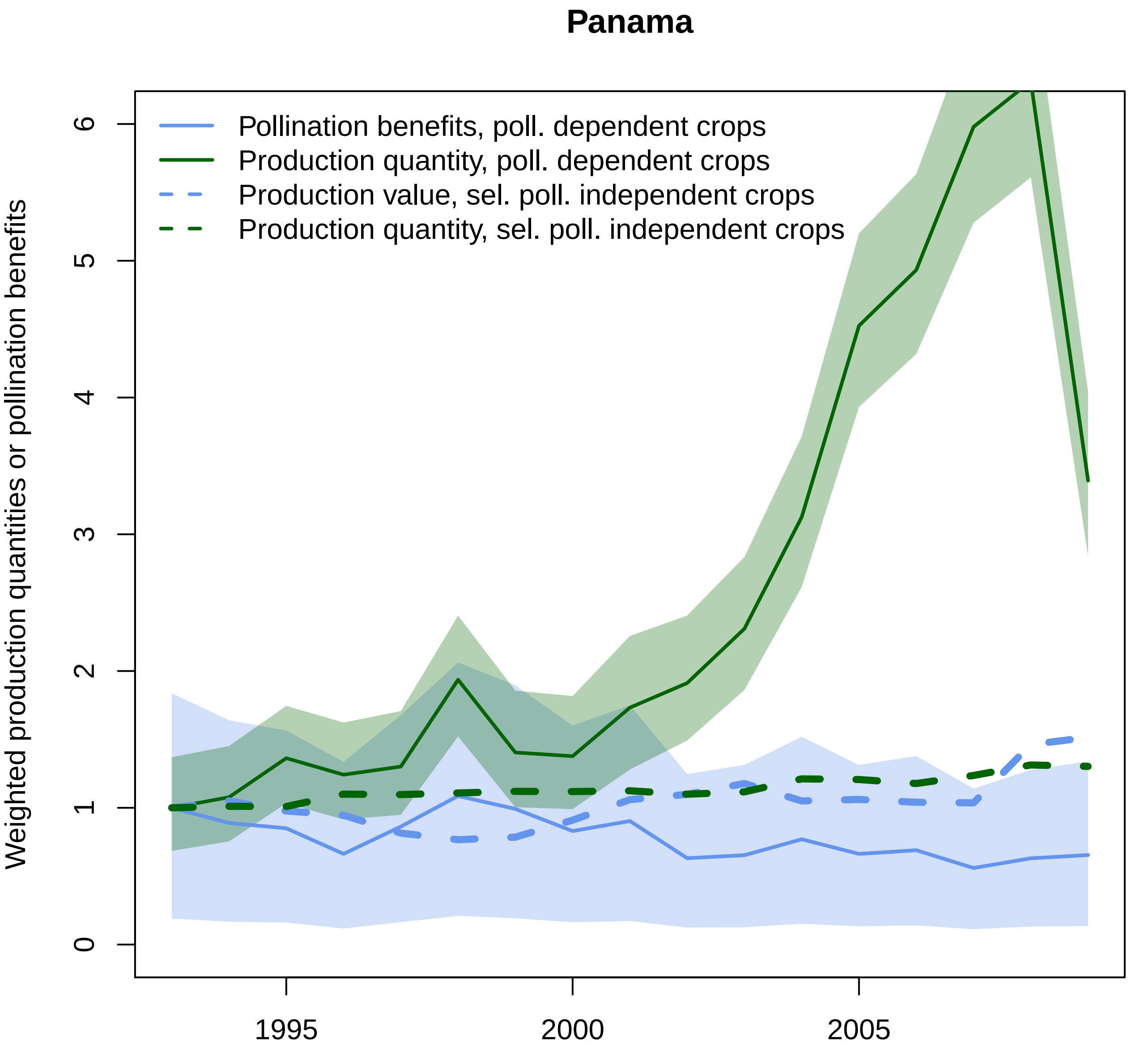
Panama
Pollination benefits, poll. dependent crops
Production quantity, poll. dependent crops
Production value, sel. poll. independent crops
Production quantity, sel. poll. independent crops
Weighted production quantities or pollination benefits
0
1
2
3
4
5
6
1995
2000
2005

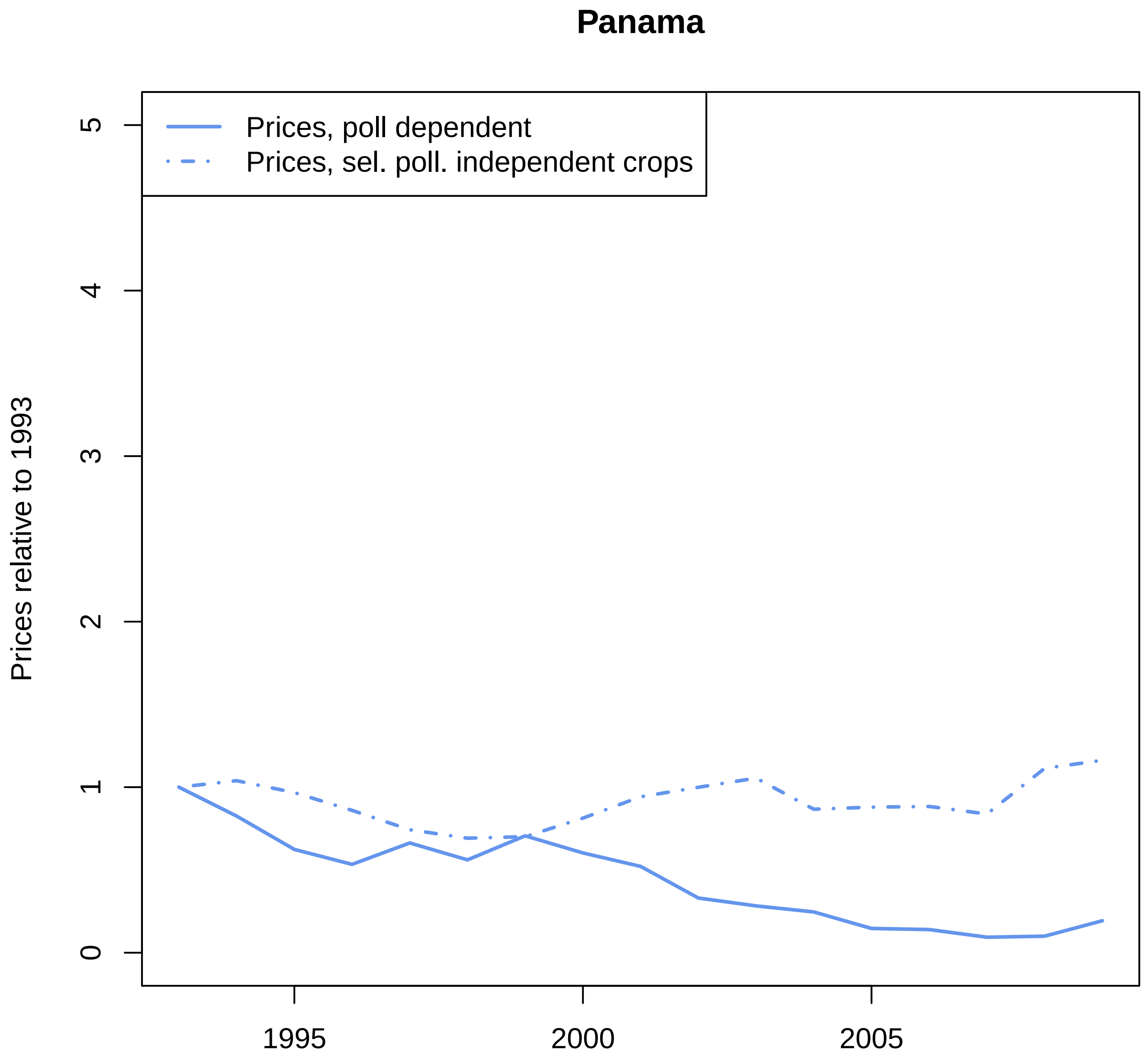

Panama
Prices, poll dependent
Prices, sel. poll. independent crops
Prices relative to 1993
0
1
2
3
4
5
1995
2000
2005

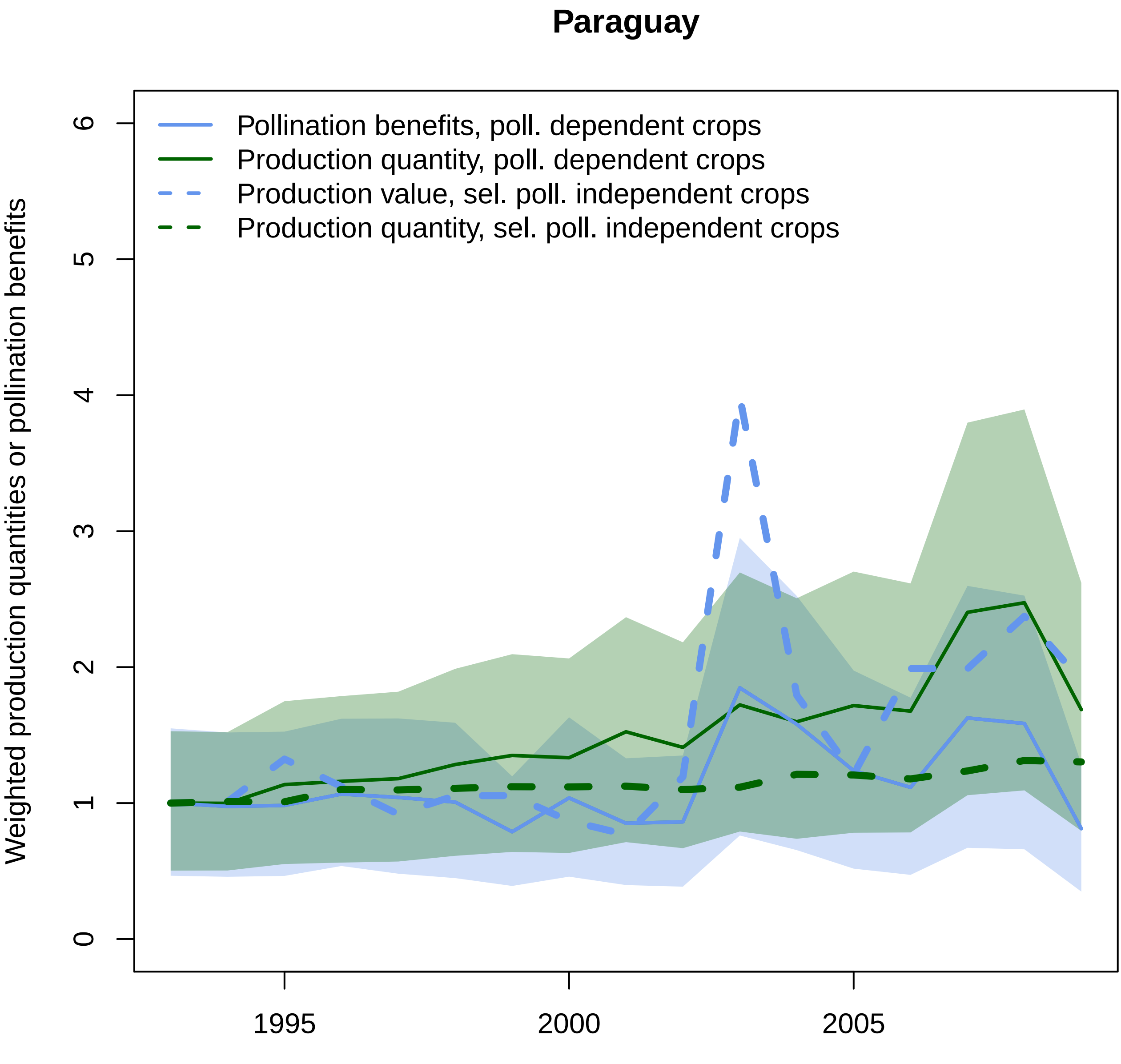
Paraguay
Pollination benefits, poll. dependent crops
Production quantity, poll. dependent crops
Production value, sel. poll. independent crops
Production quantity, sel. poll. independent crops
Weighted production quantities or pollination benefits
0
1
2
3
4
5
6
1995
2000
2005

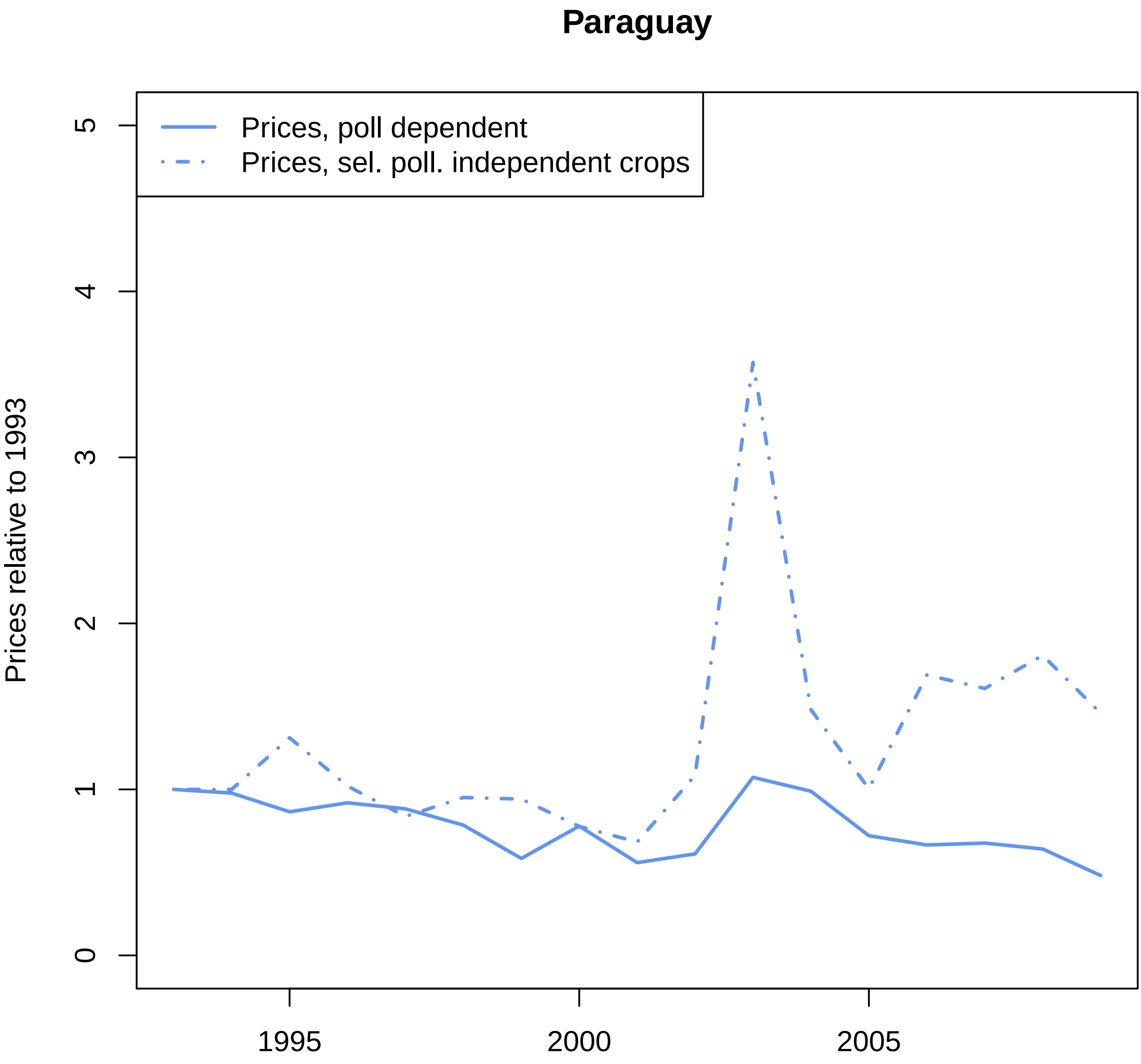

Paraguay
Prices, poll dependent
Prices, sel. poll. independent crops
Prices relative to 1993
0
1
2
3
4
5
1995
2000
2005

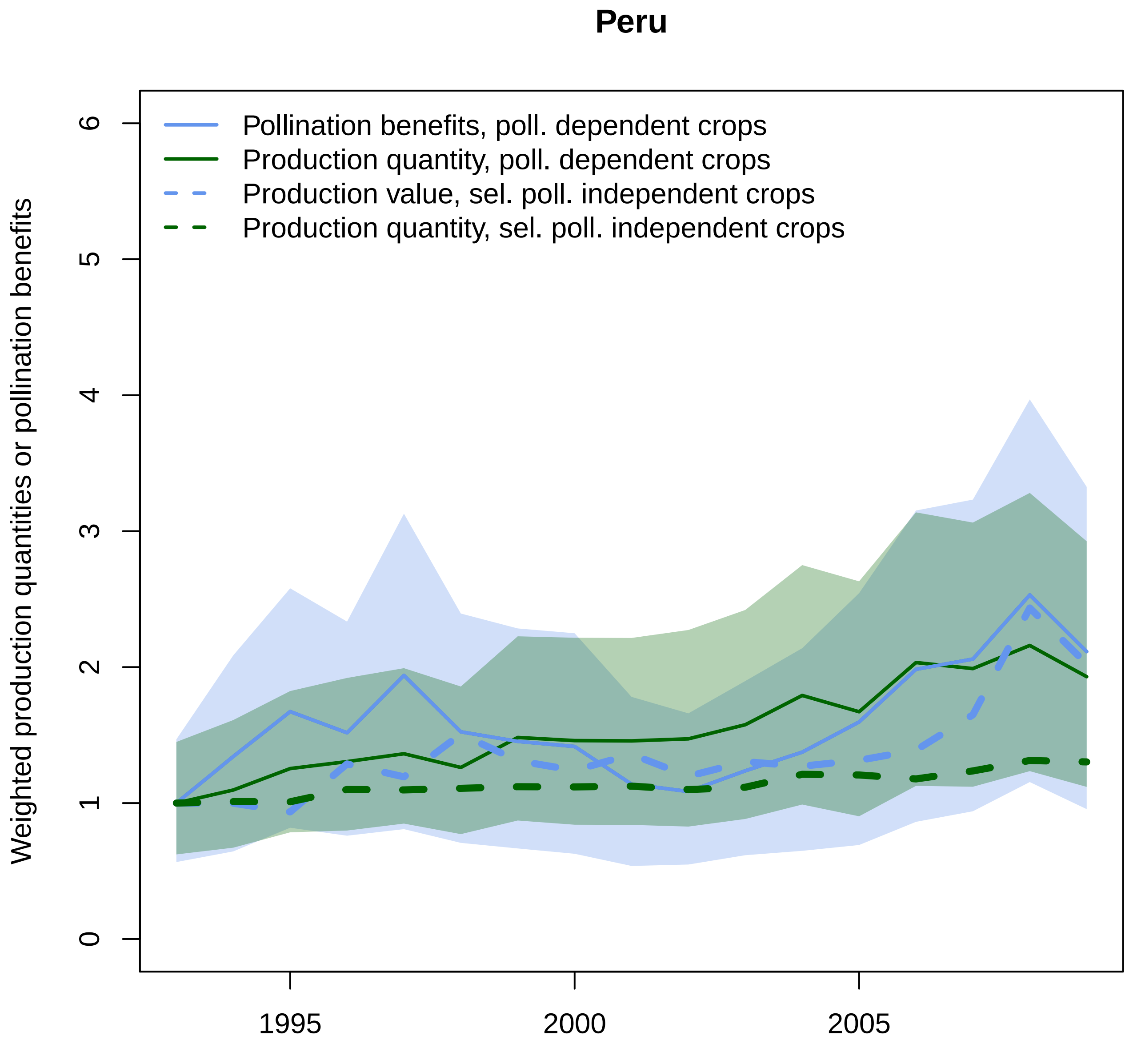

Peru
Pollination benefits, poll. dependent crops
Production quantity, poll. dependent crops
Production value, sel. poll. independent crops
Production quantity, sel. poll. independent crops
Weighted production quantities or pollination benefits
0
1
2
3
4
5
6
1995
2000
2005

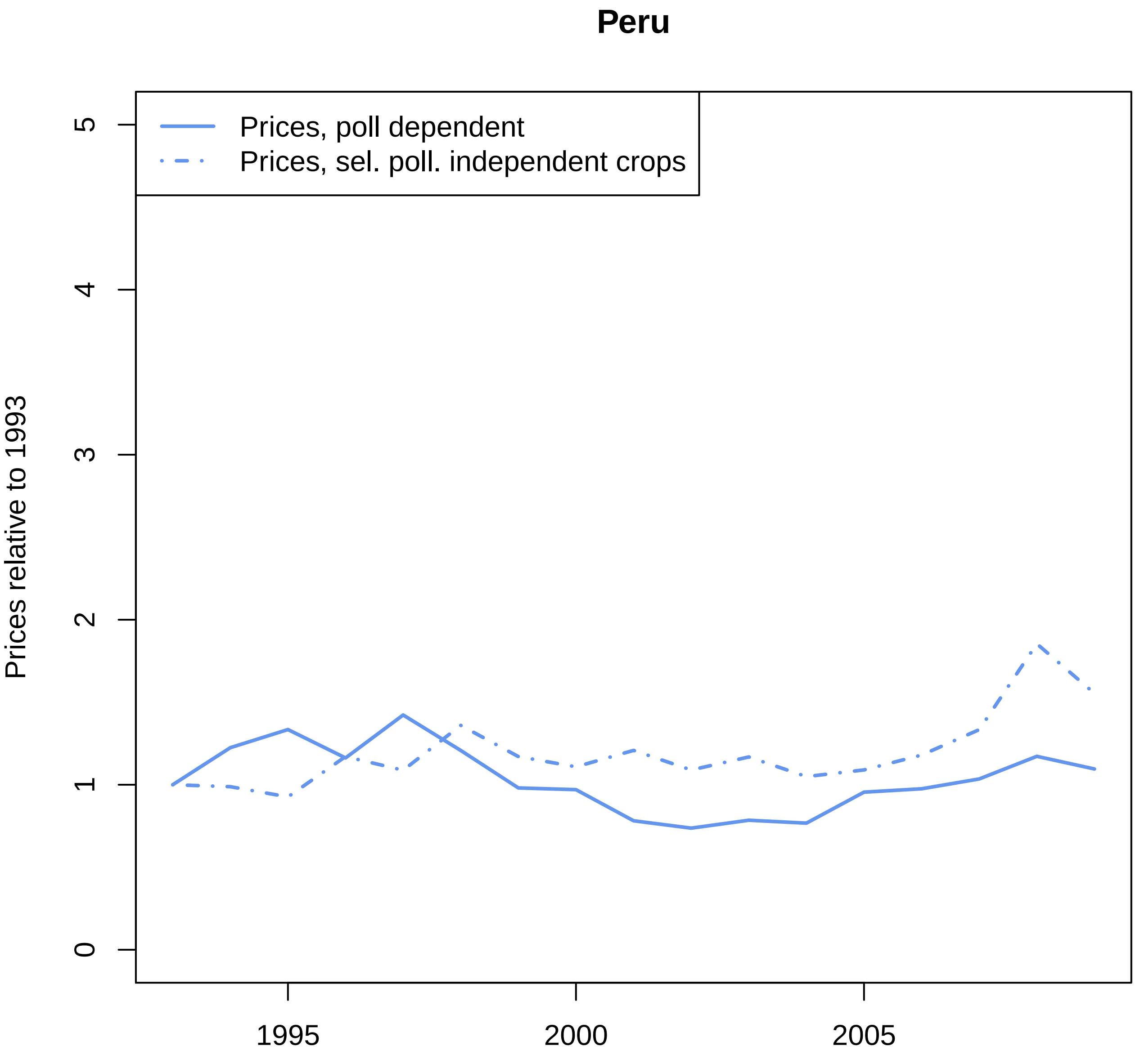
Peru
Prices, poll dependent
Prices, sel. poll. independent crops
Prices relative to 1993
0
1
2
3
4
5
1995
2000
2005

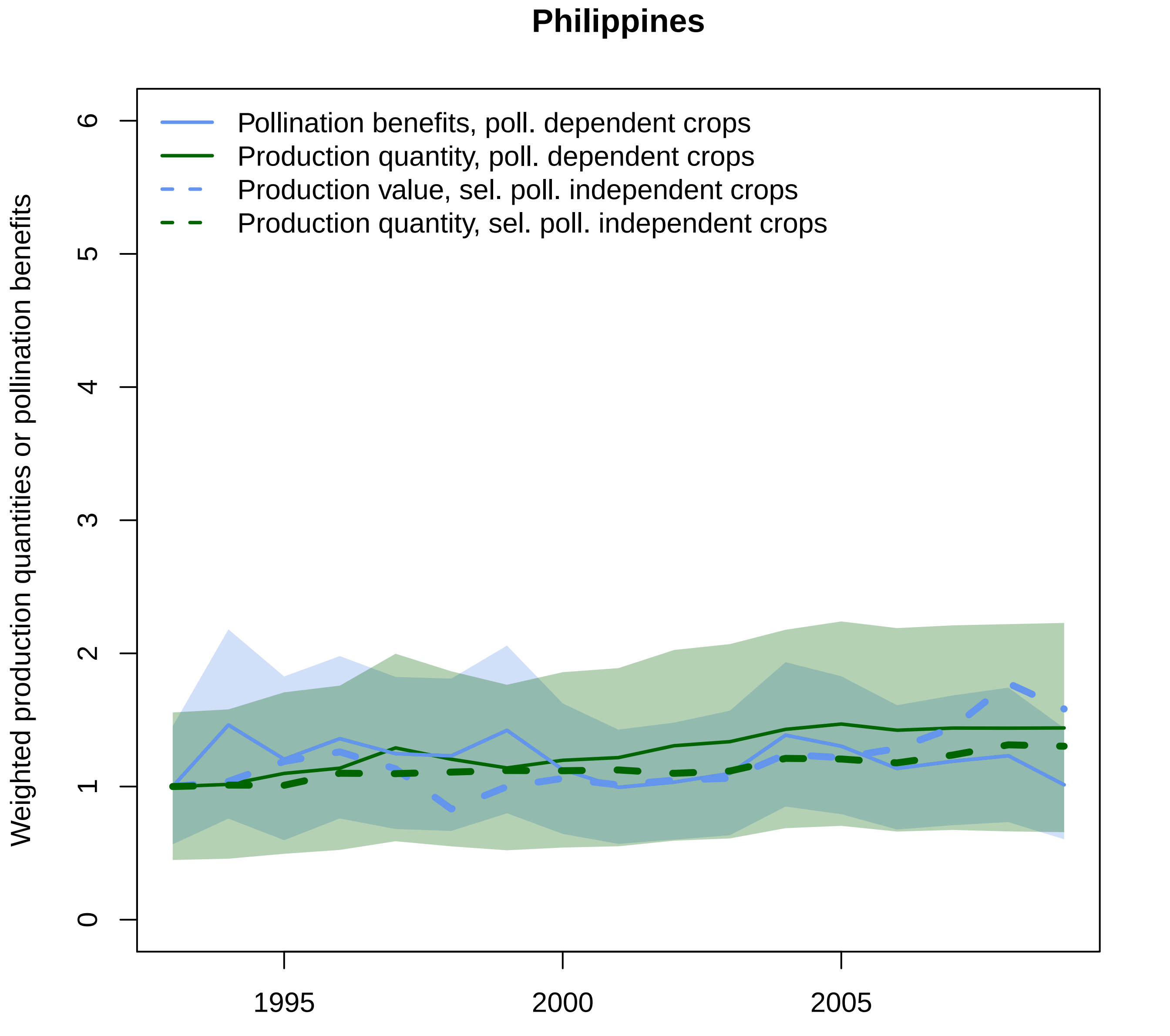

Philippines
Pollination benefits, poll. dependent crops
Production quantity, poll. dependent crops
Production value, sel. poll. independent crops
Production quantity, sel. poll. independent crops
Weighted production quantities or pollination benefits
0
1
2
3
4
5
6
1995
2000
2005

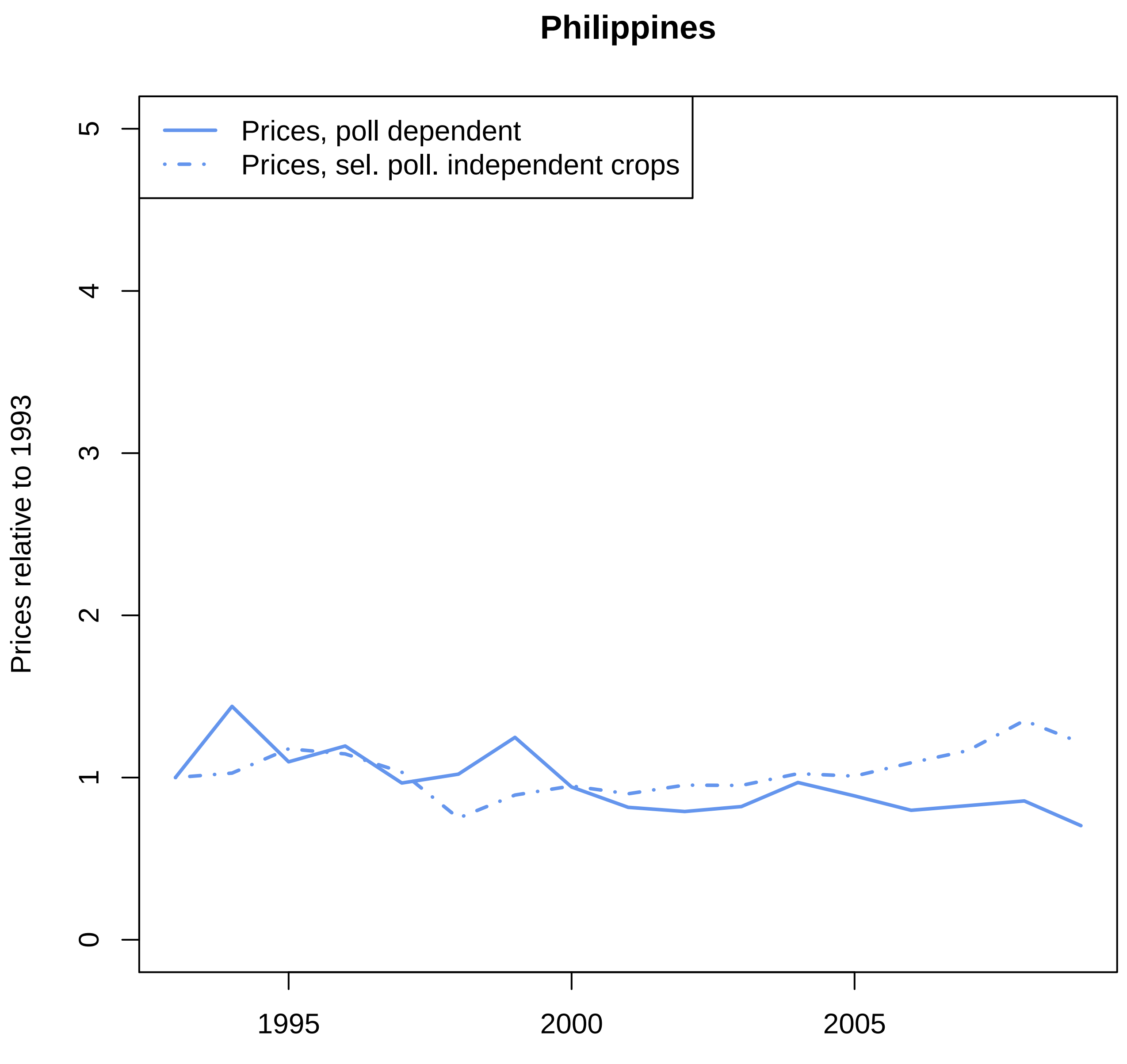
Philippines
Prices, poll dependent
Prices, sel. poll. independent crops
Prices relative to 1993
0
1
2
3
4
5
1995
2000
2005

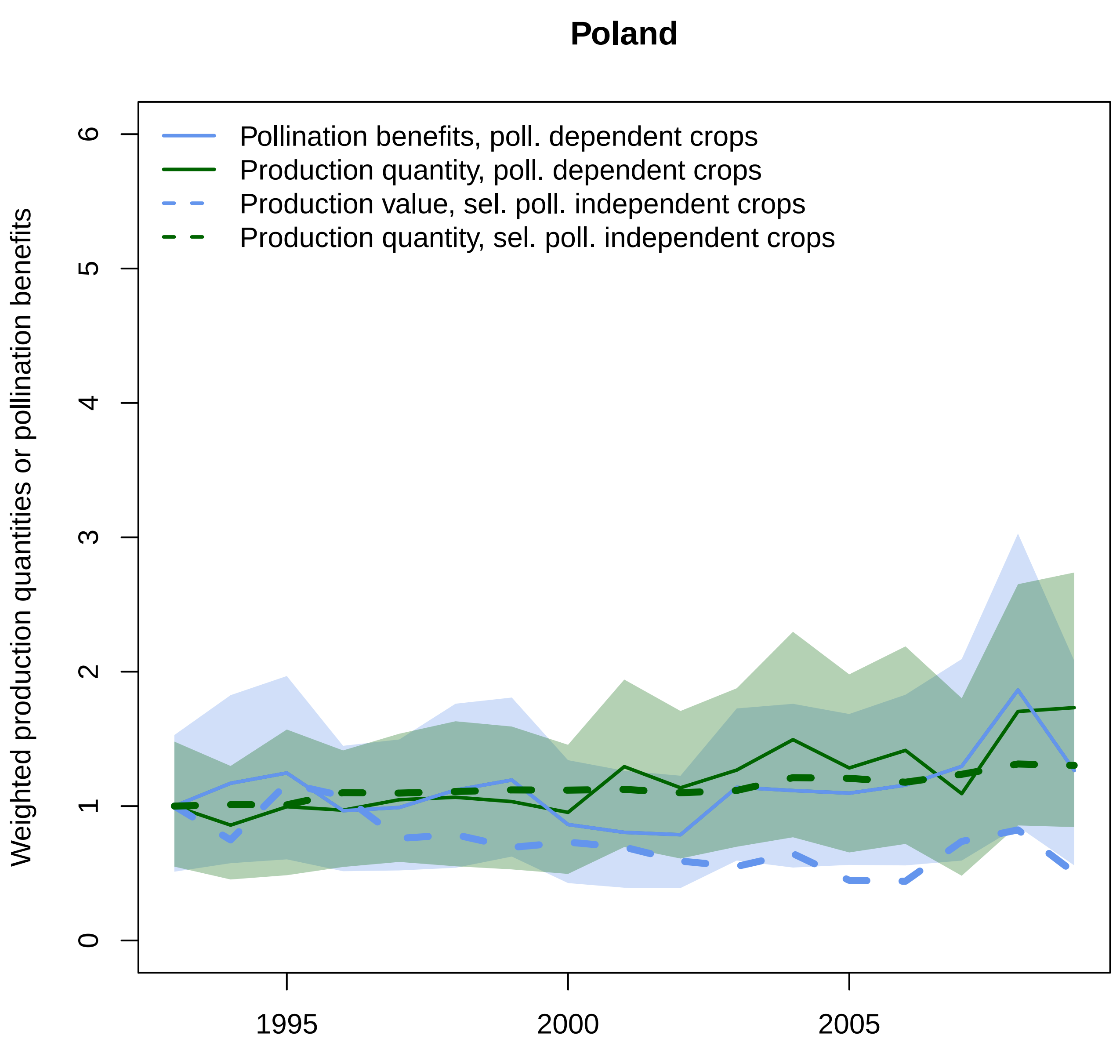
Poland
Pollination benefits, poll. dependent crops
Production quantity, poll. dependent crops
Production value, sel. poll. independent crops
Production quantity, sel. poll. independent crops
Weighted production quantities or pollination benefits
0
1
2
3
4
5
6
1995
2000
2005

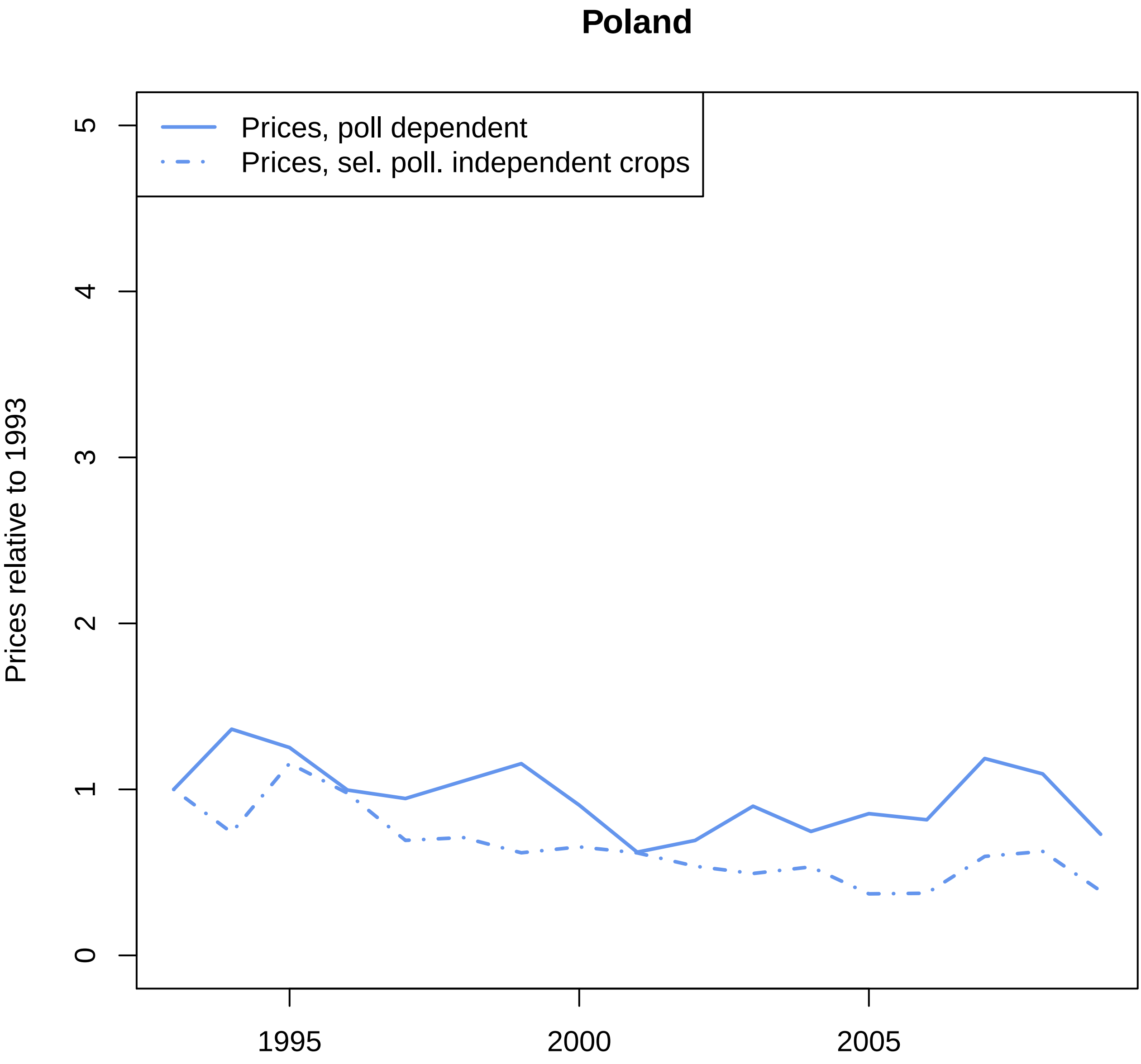
Poland
Prices, poll dependent
Prices, sel. poll. independent crops
Prices relative to 1993
0
1
2
3
4
5
1995
2000
2005

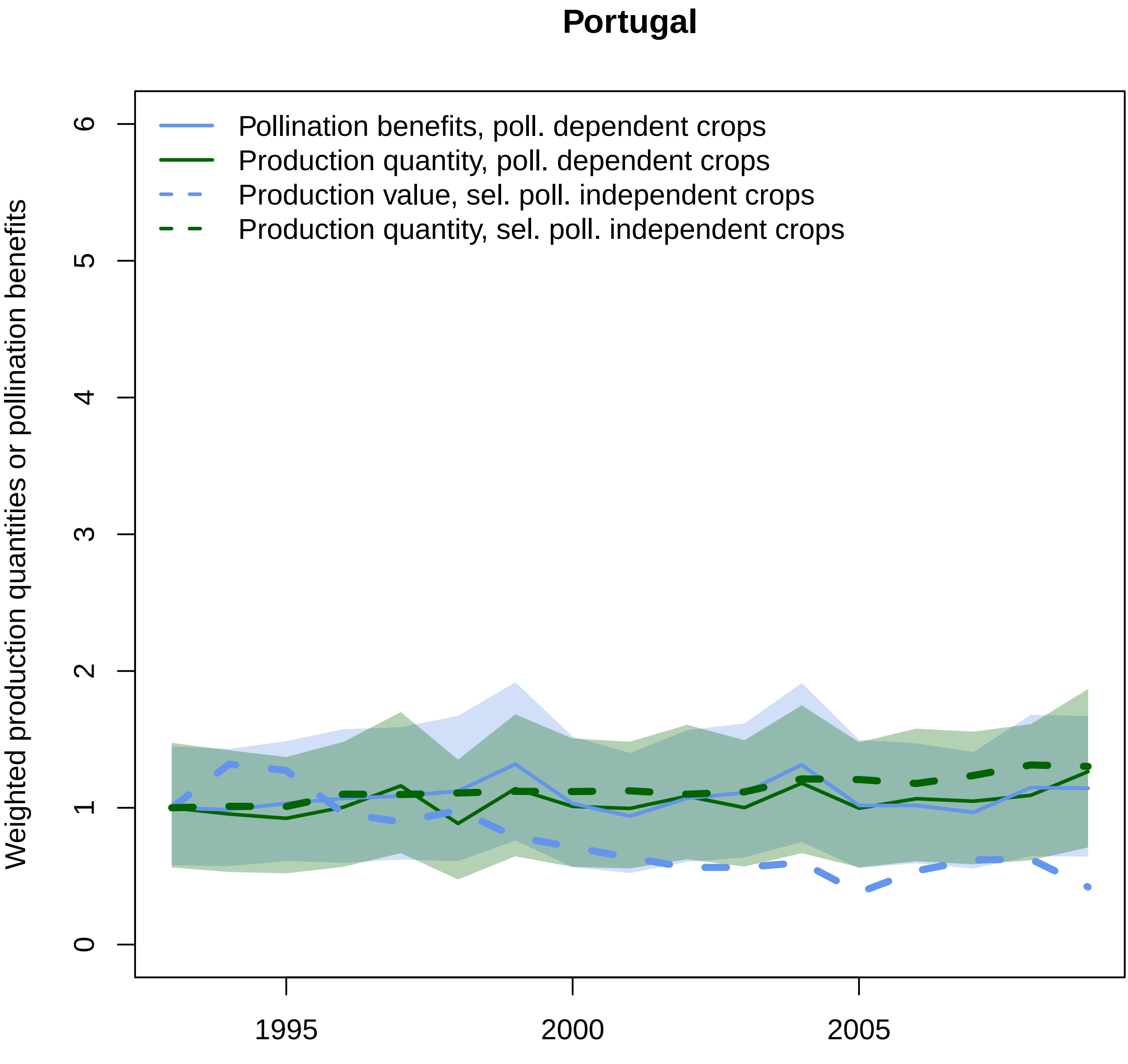

Portugal
Pollination benefits, poll. dependent crops
Production quantity, poll. dependent crops
Production value, sel. poll. independent crops
Production quantity, sel. poll. independent crops
Weighted production quantities or pollination benefits
0
1
2
3
4
5
6
1995
2000
2005

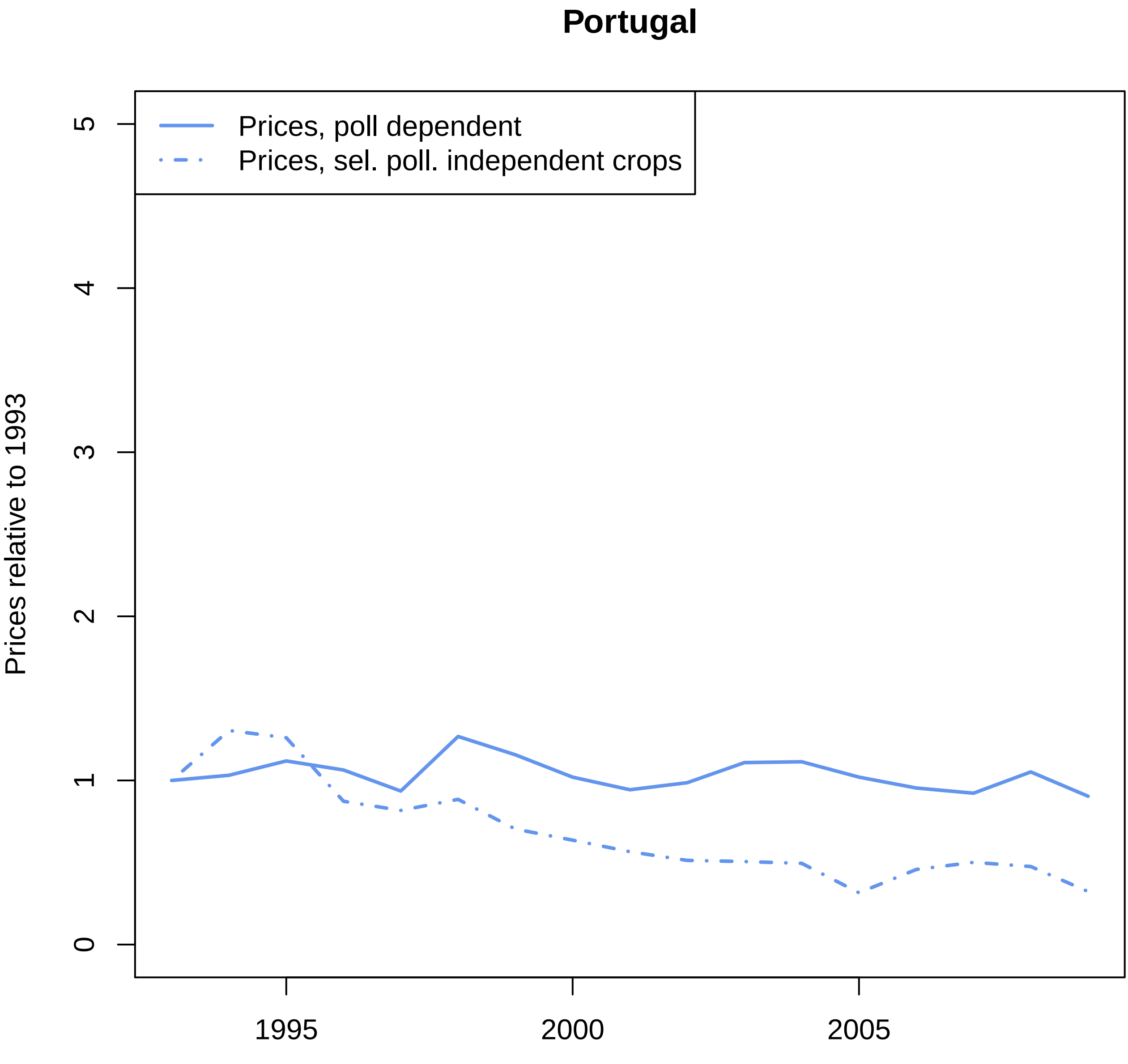

Portugal
Prices, poll dependent
Prices, sel. poll. independent crops
Prices relative to 1993
0
1
2
3
4
5
1995
2000
2005

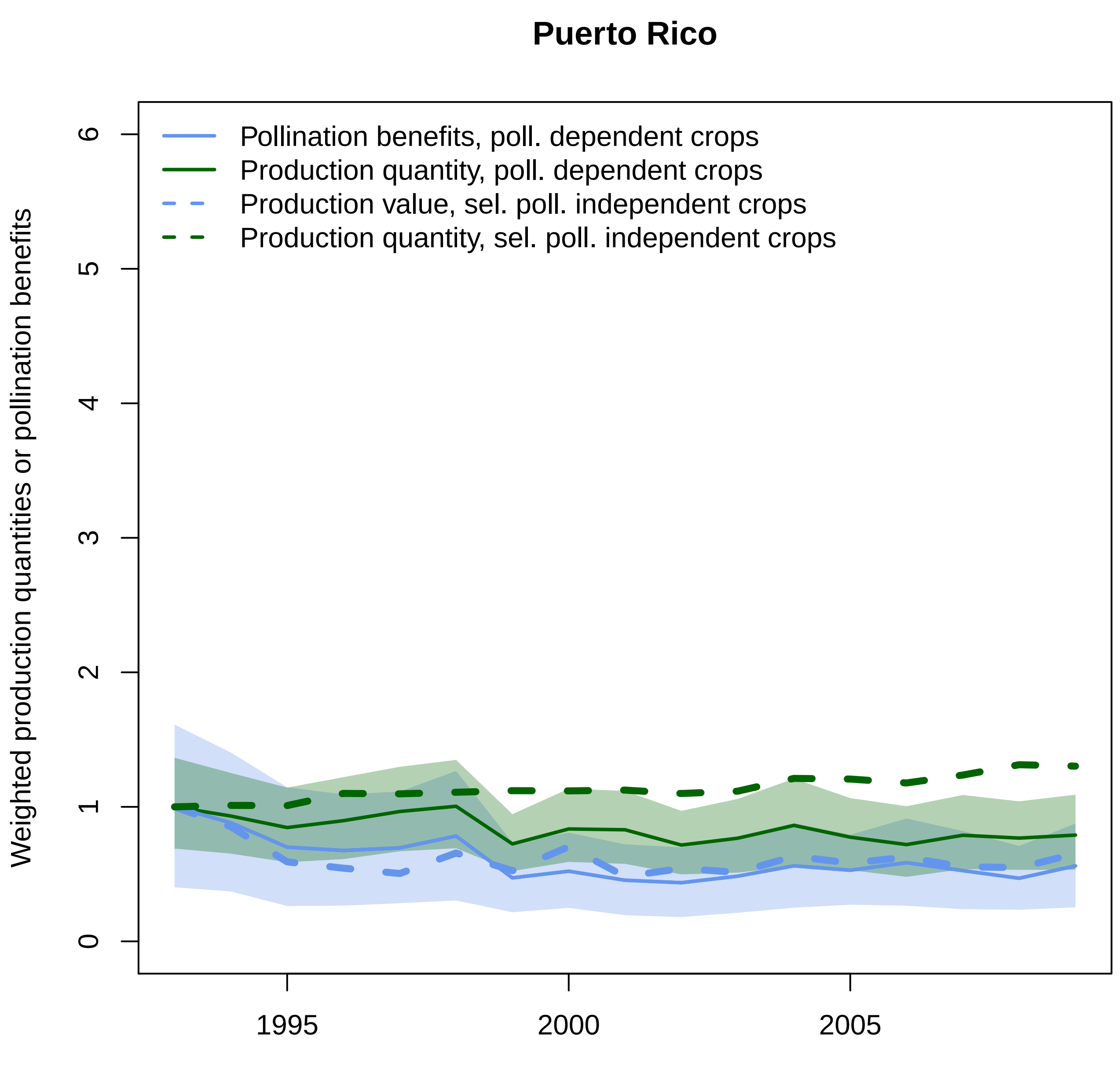
Puerto Rico
Pollination benefits, poll. dependent crops
Production quantity, poll. dependent crops
Production value, sel. poll. independent crops
Production quantity, sel. poll. independent crops
Weighted production quantities or pollination benefits
0
1
2
3
4
5
6
1995
2000
2005

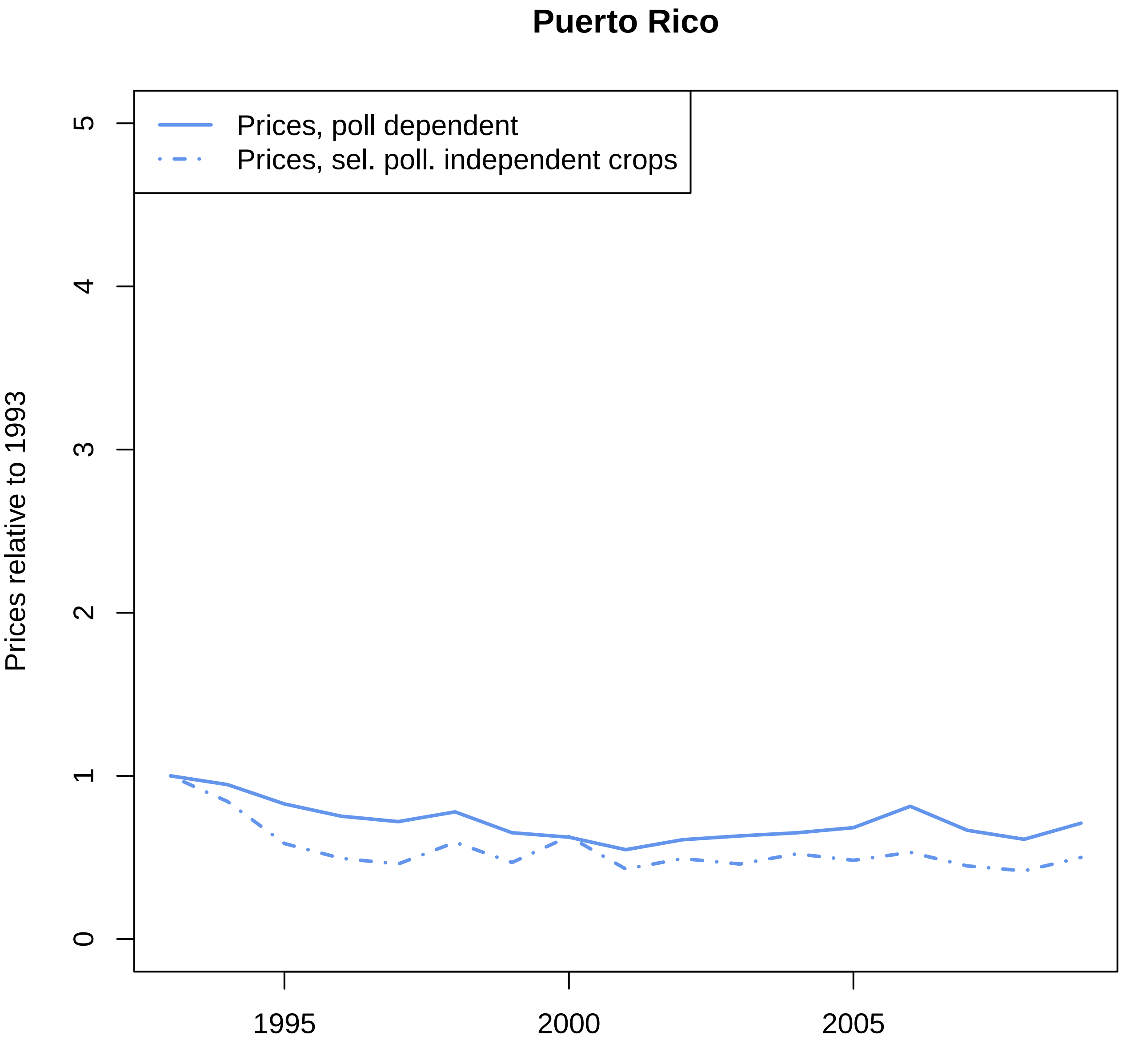
Puerto Rico
Prices, poll dependent
Prices, sel. poll. independent crops
Prices relative to 1993
0
1
2
3
4
5
1995
2000
2005

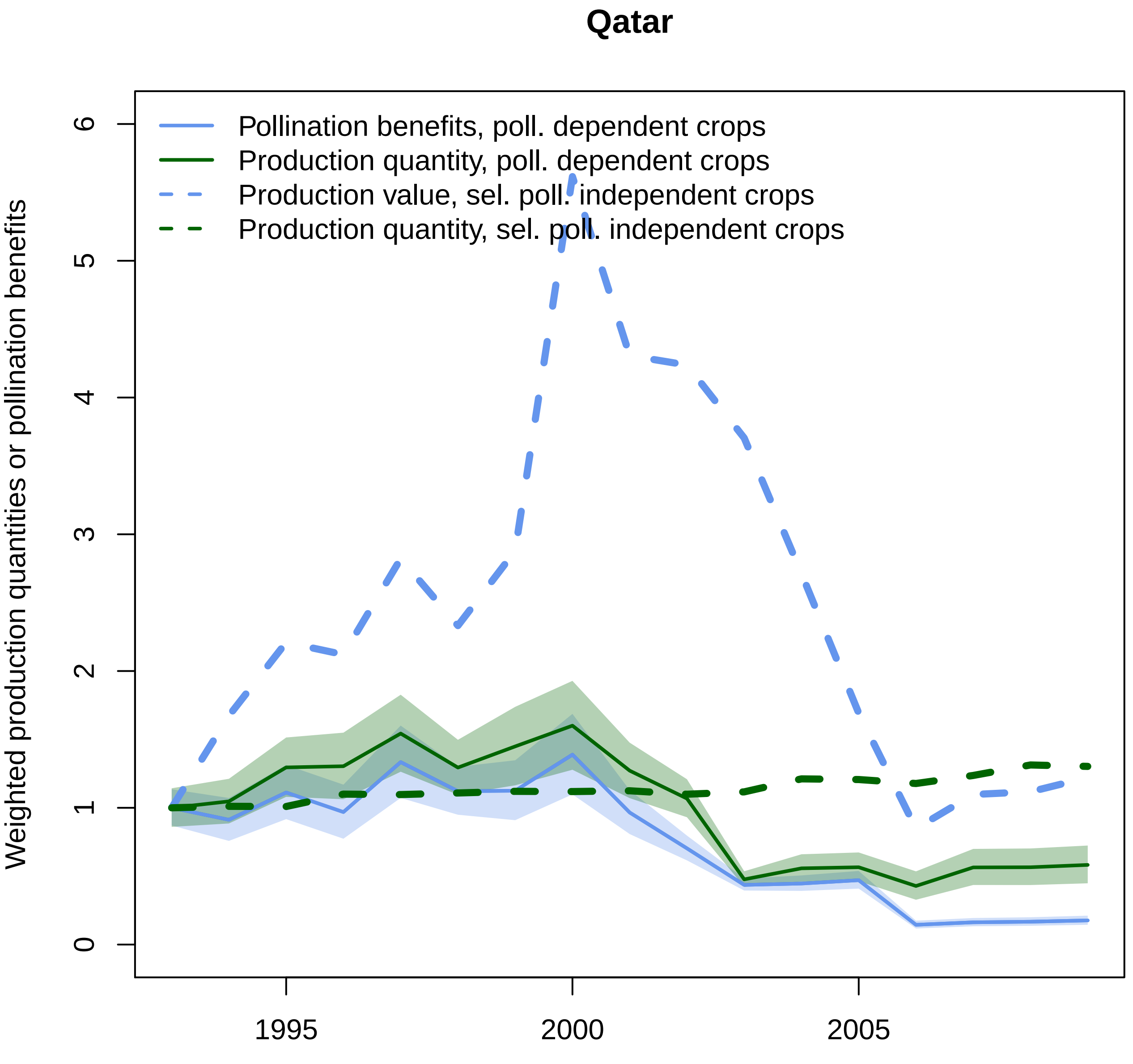
Qatar
Pollination benefits, poll. dependent crops
Production quantity, poll. dependent crops
Production value, sel. poll. independent crops
Production quantity, sel. poll. independent crops
Weighted production quantities or pollination benefits
0
1
2
3
4
5
6
1995
2000
2005

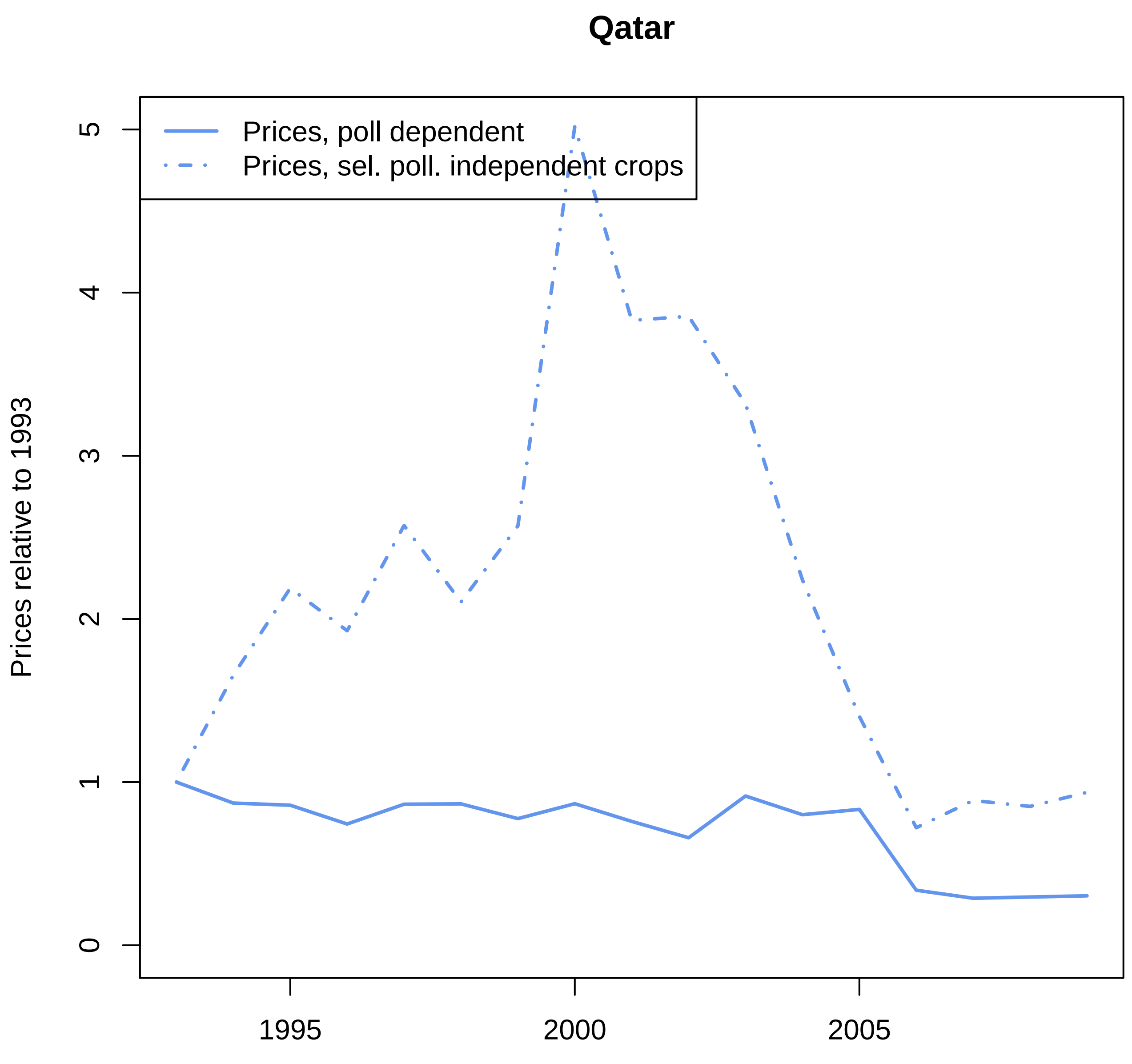
Qatar
Prices, poll dependent
Prices, sel. poll. independent crops
Prices relative to 1993
0
1
2
3
4
5
1995
2000
2005

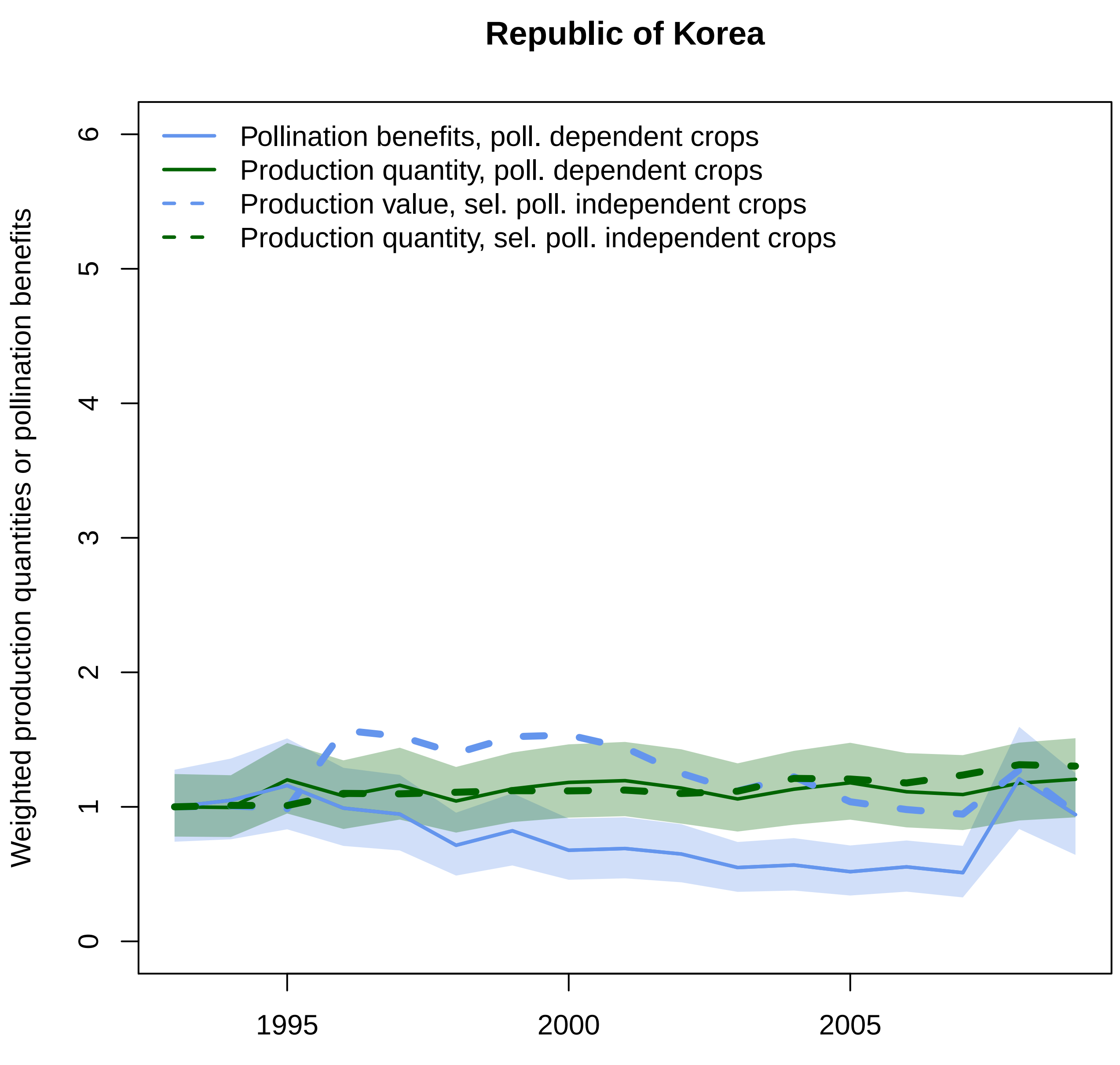

Republic of Korea
Pollination benefits, poll. dependent crops
Production quantity, poll. dependent crops
Production value, sel. poll. independent crops
Production quantity, sel. poll. independent crops
Weighted production quantities or pollination benefits
0
1
2
3
4
5
6
1995
2000
2005

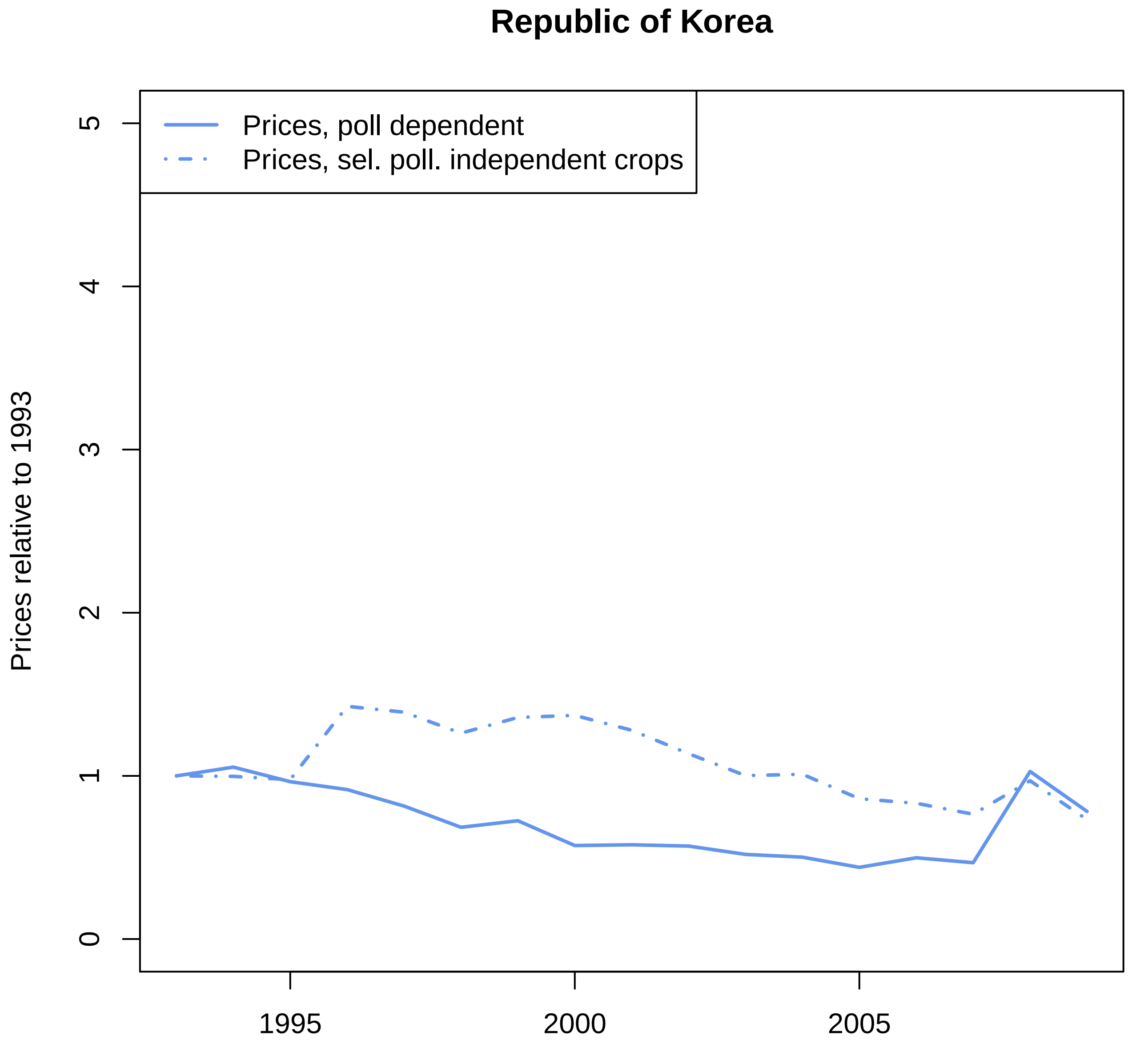
Republic of Korea
Prices, poll dependent
Prices, sel. poll. independent crops
Prices relative to 1993
0
1
2
3
4
5
1995
2000
2005

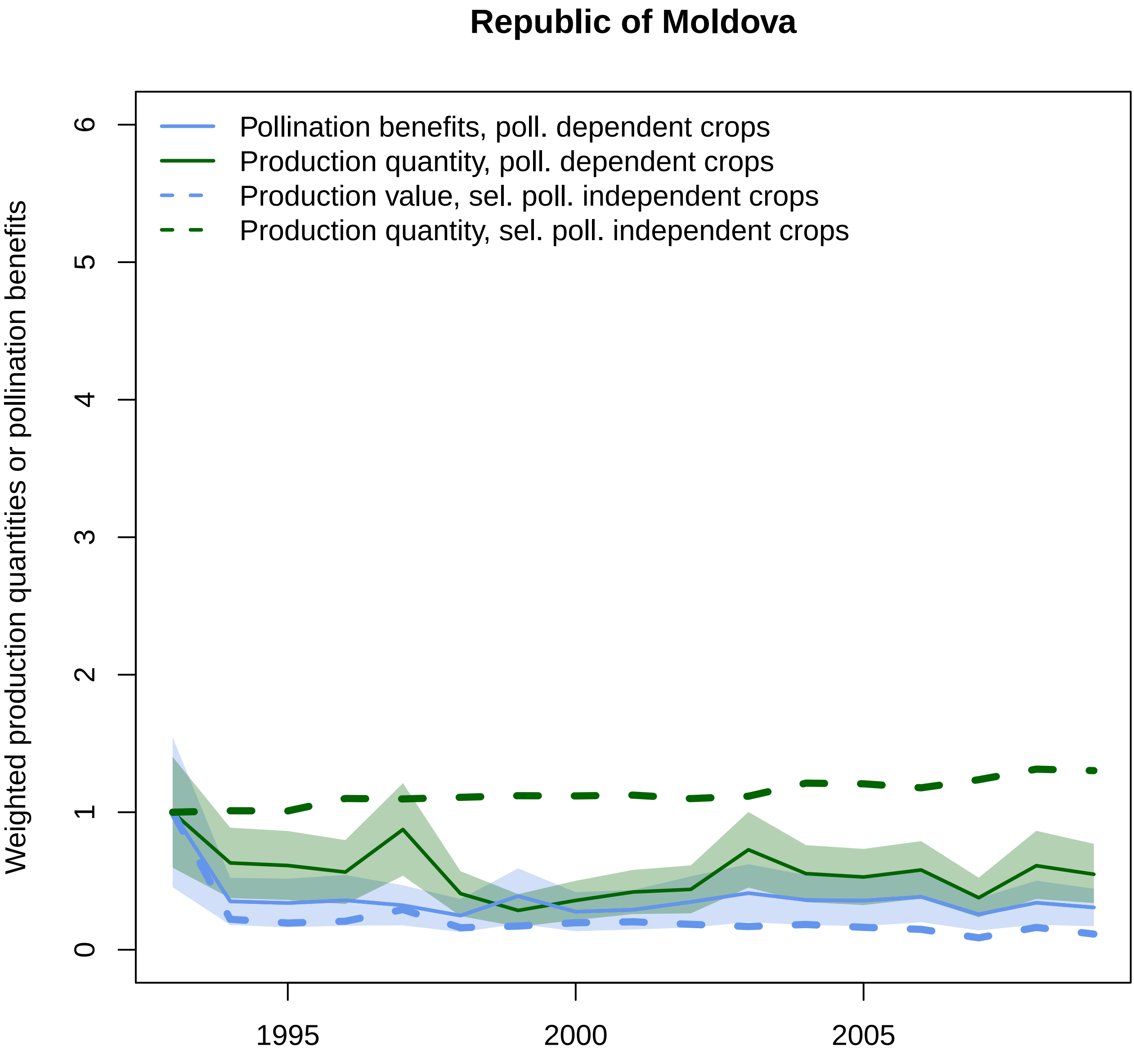
Republic of Moldova
Pollination benefits, poll. dependent crops
Production quantity, poll. dependent crops
Production value, sel. poll. independent crops
Production quantity, sel. poll. independent crops
Weighted production quantities or pollination benefits
0
1
2
3
4
5
6
1995
2000
2005

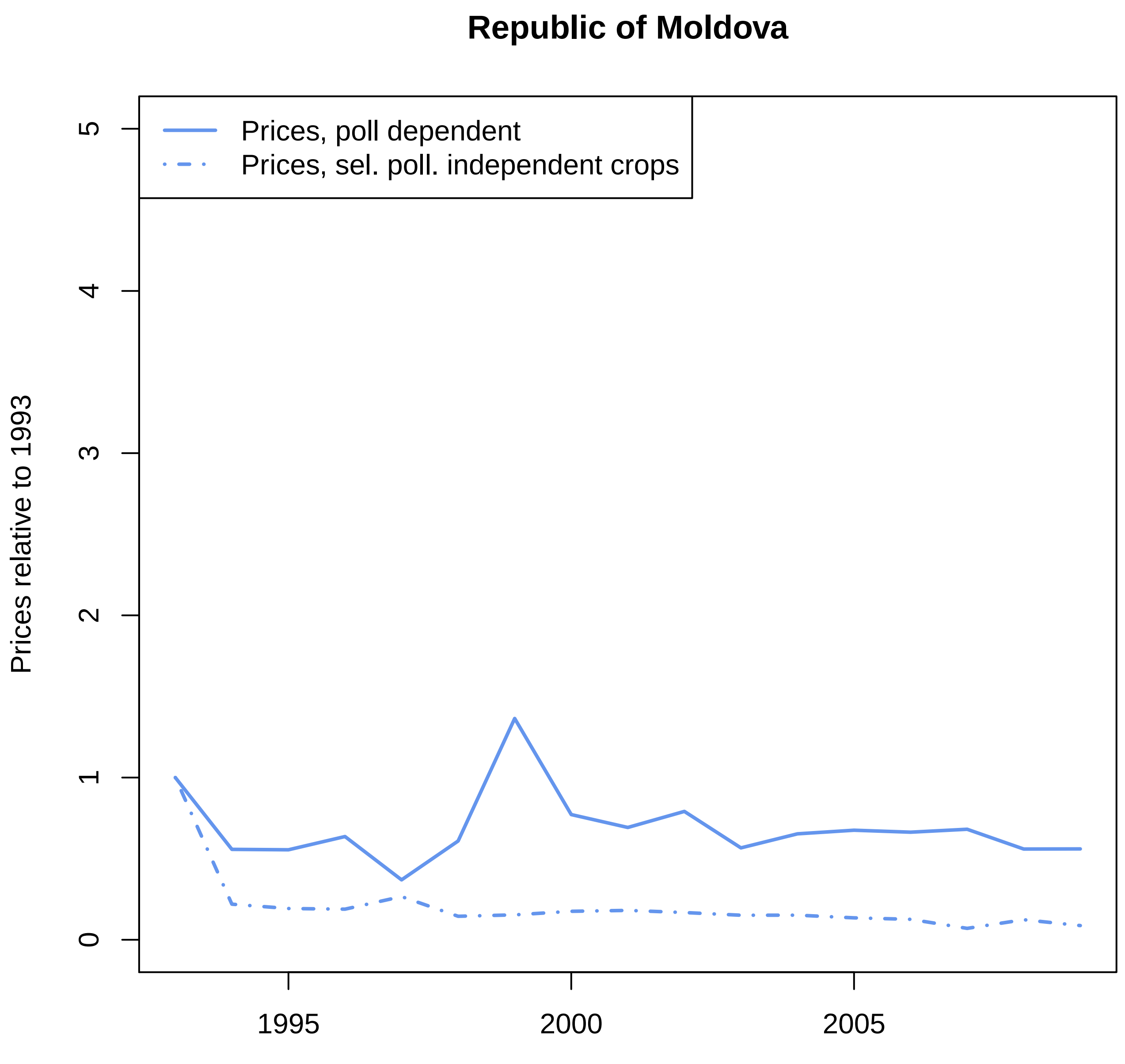
Republic of Moldova
Prices, poll dependent
Prices, sel. poll. independent crops
Prices relative to 1993
0
1
2
3
4
5
1995
2000
2005

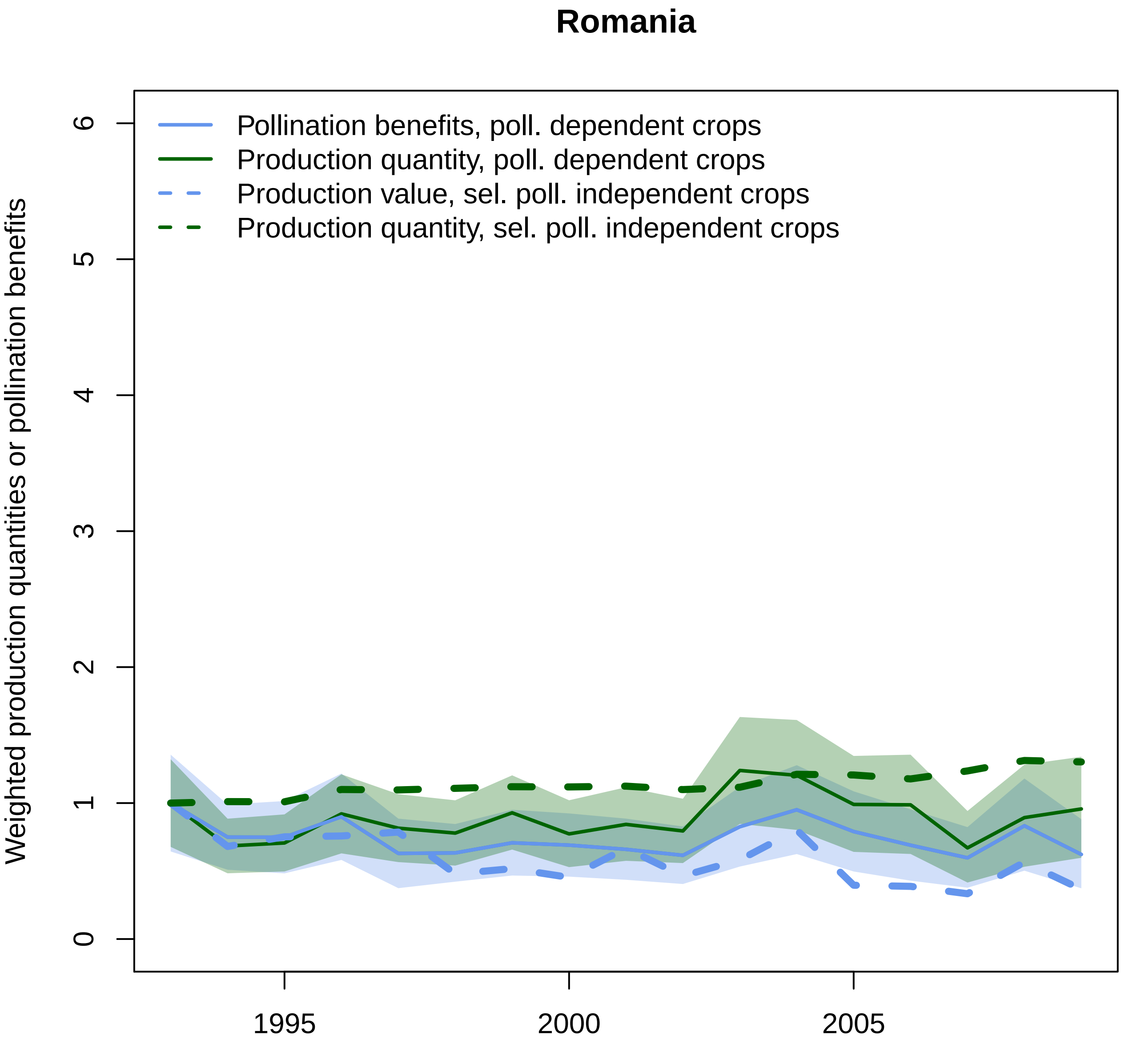

Romania
Pollination benefits, poll. dependent crops
Production quantity, poll. dependent crops
Production value, sel. poll. independent crops
Production quantity, sel. poll. independent crops
Weighted production quantities or pollination benefits
0
1
2
3
4
5
6
1995
2000
2005

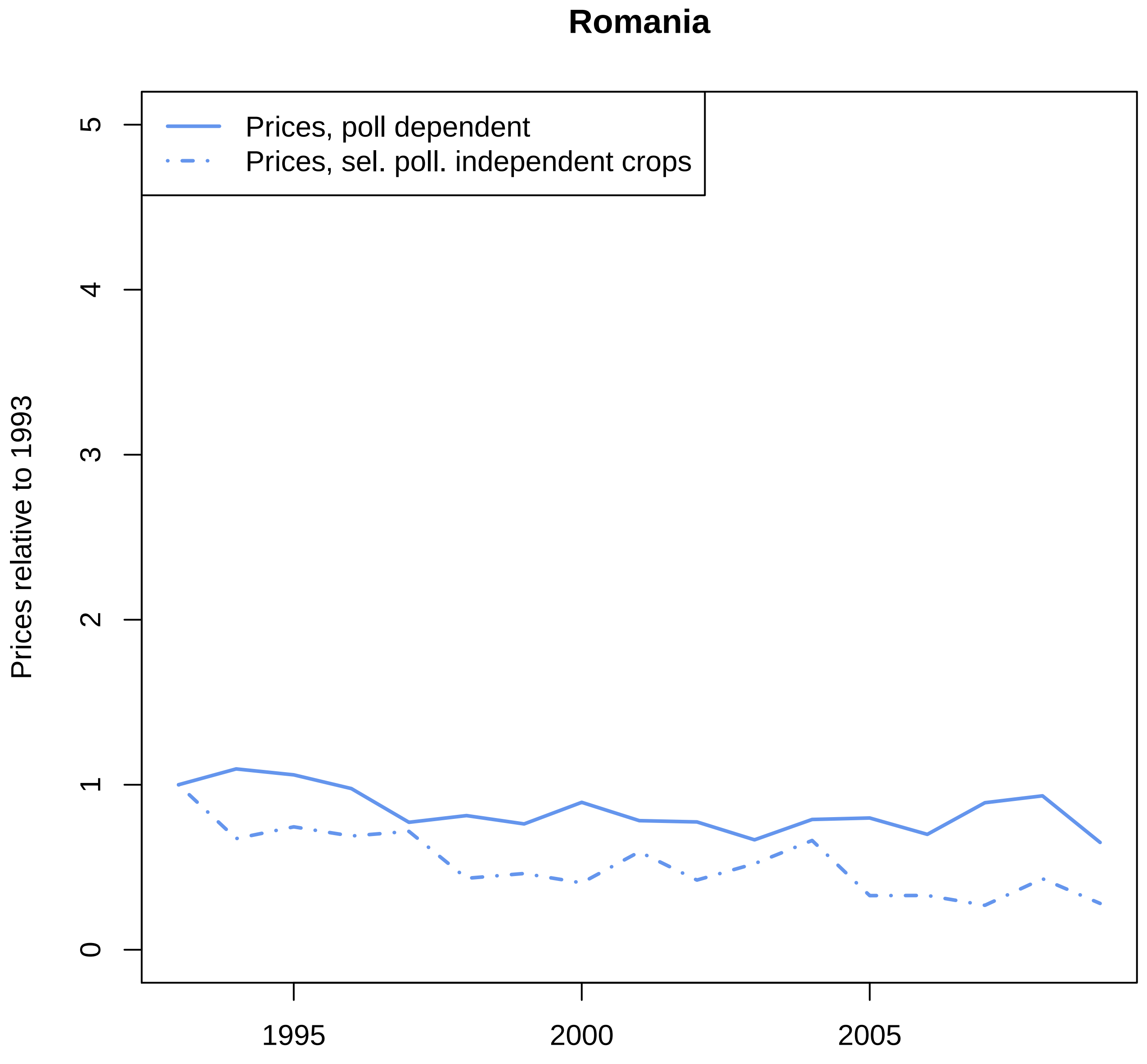

Romania
Prices, poll dependent
Prices, sel. poll. independent crops
Prices relative to 1993
0
1
2
3
4
5
1995
2000
2005

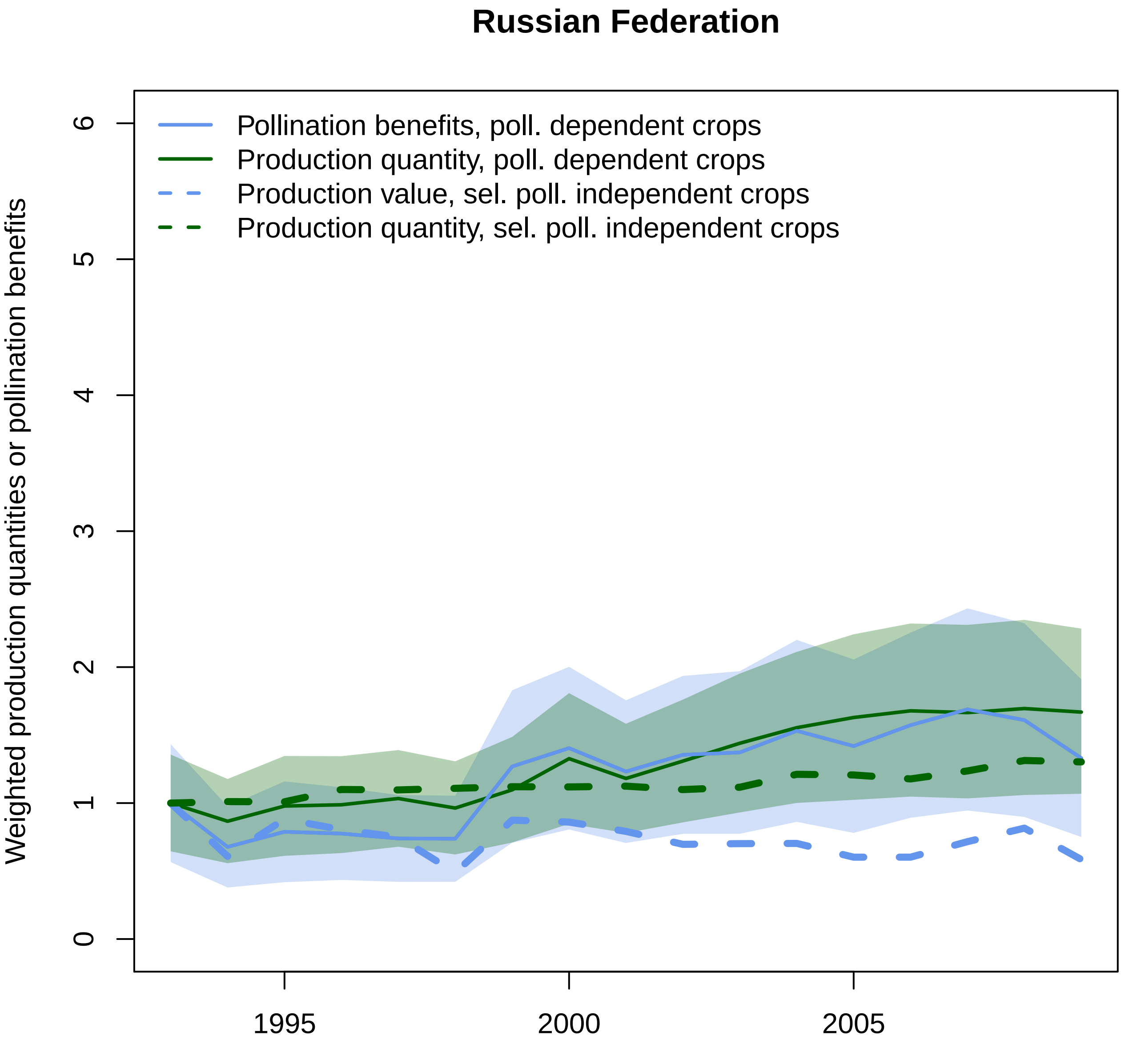

Russian Federation
Pollination benefits, poll. dependent crops
Production quantity, poll. dependent crops
Production value, sel. poll. independent crops
Production quantity, sel. poll. independent crops
Weighted production quantities or pollination benefits
0
1
2
3
4
5
6
1995
2000
2005

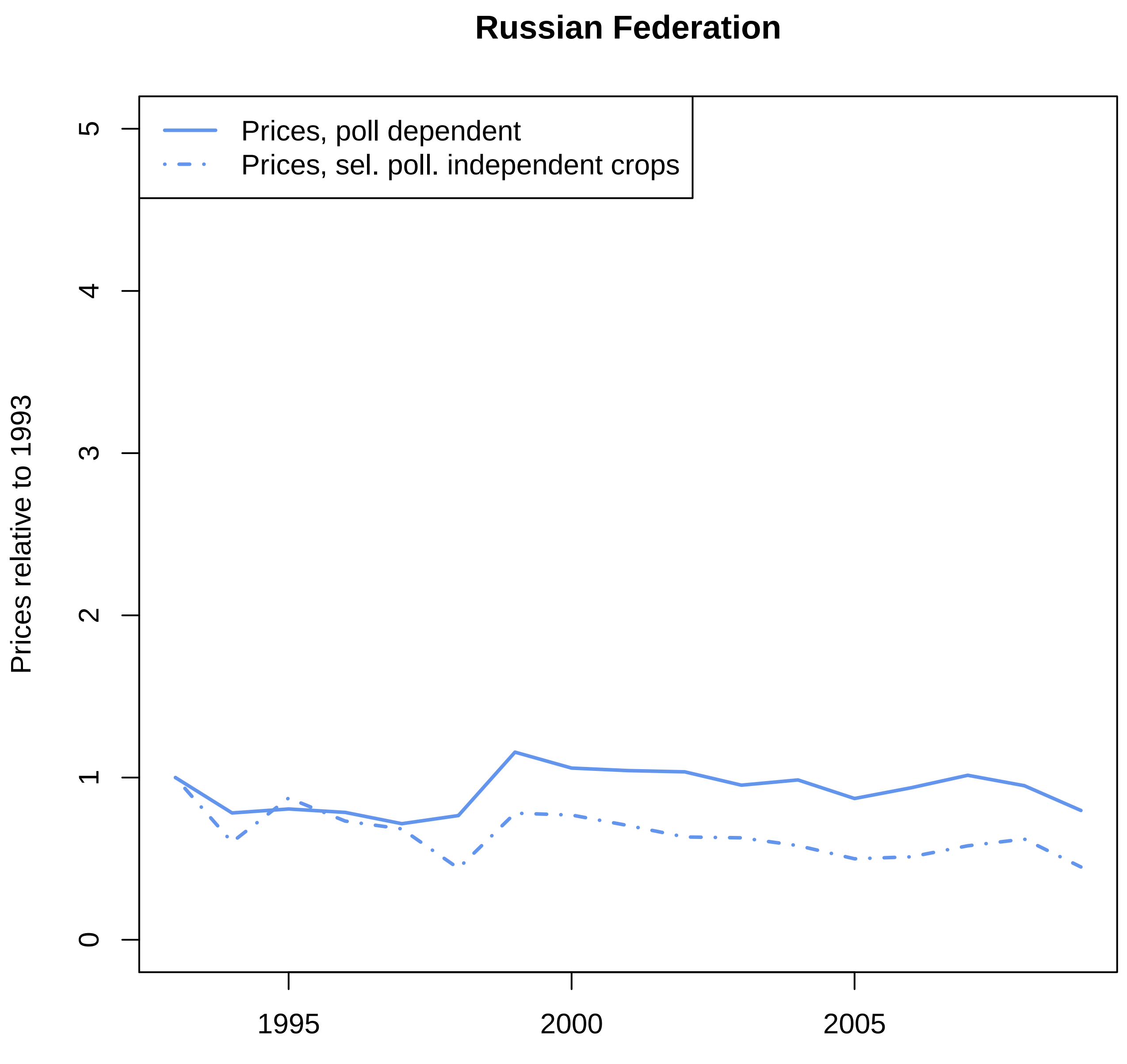

Russian Federation
Prices, poll dependent
Prices, sel. poll. independent crops
Prices relative to 1993
0
1
2
3
4
5
1995
2000
2005

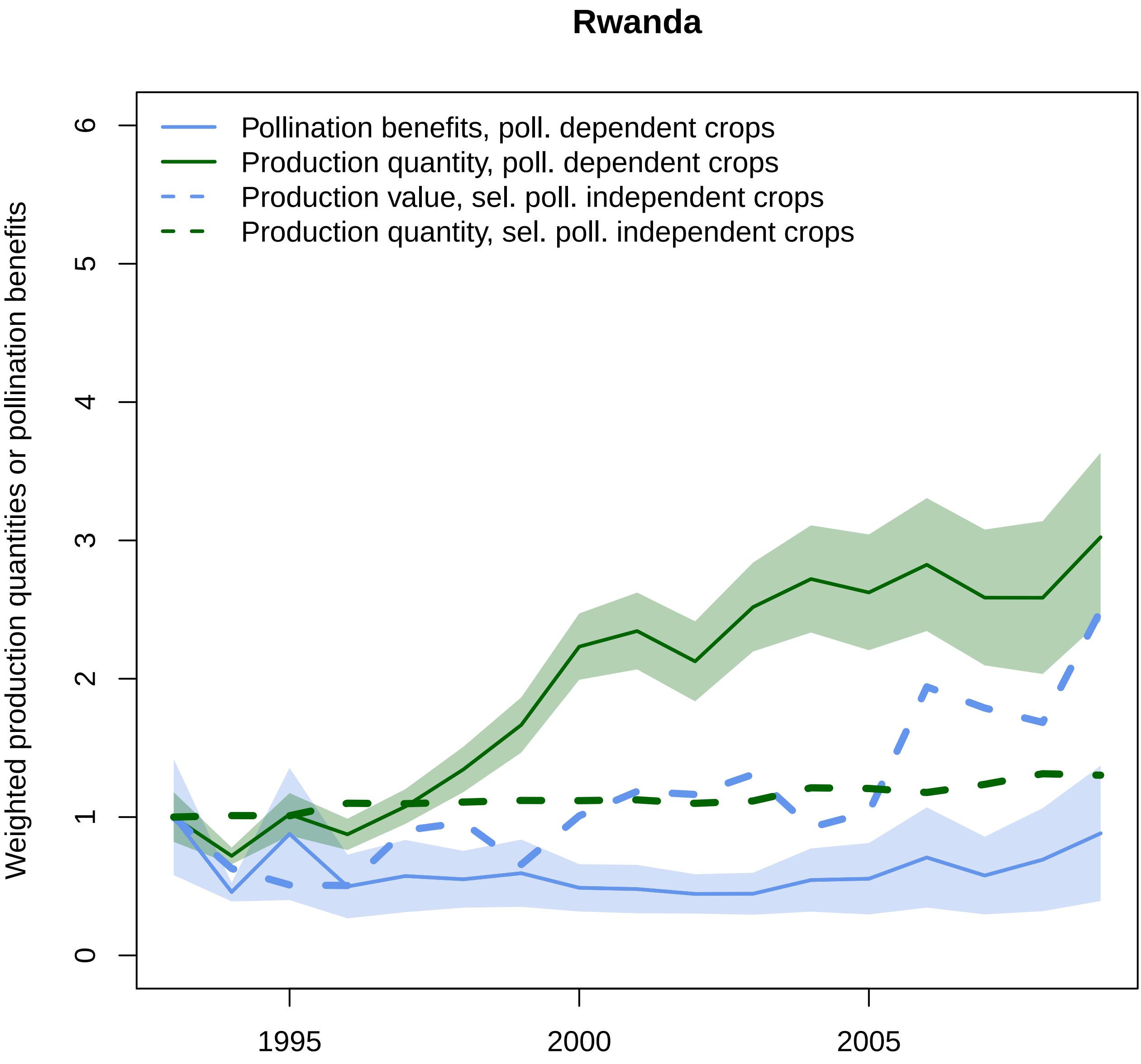

Rwanda
Pollination benefits, poll. dependent crops
Production quantity, poll. dependent crops
Production value, sel. poll. independent crops
Production quantity, sel. poll. independent crops
Weighted production quantities or pollination benefits
0
1
2
3
4
5
6
1995
2000
2005

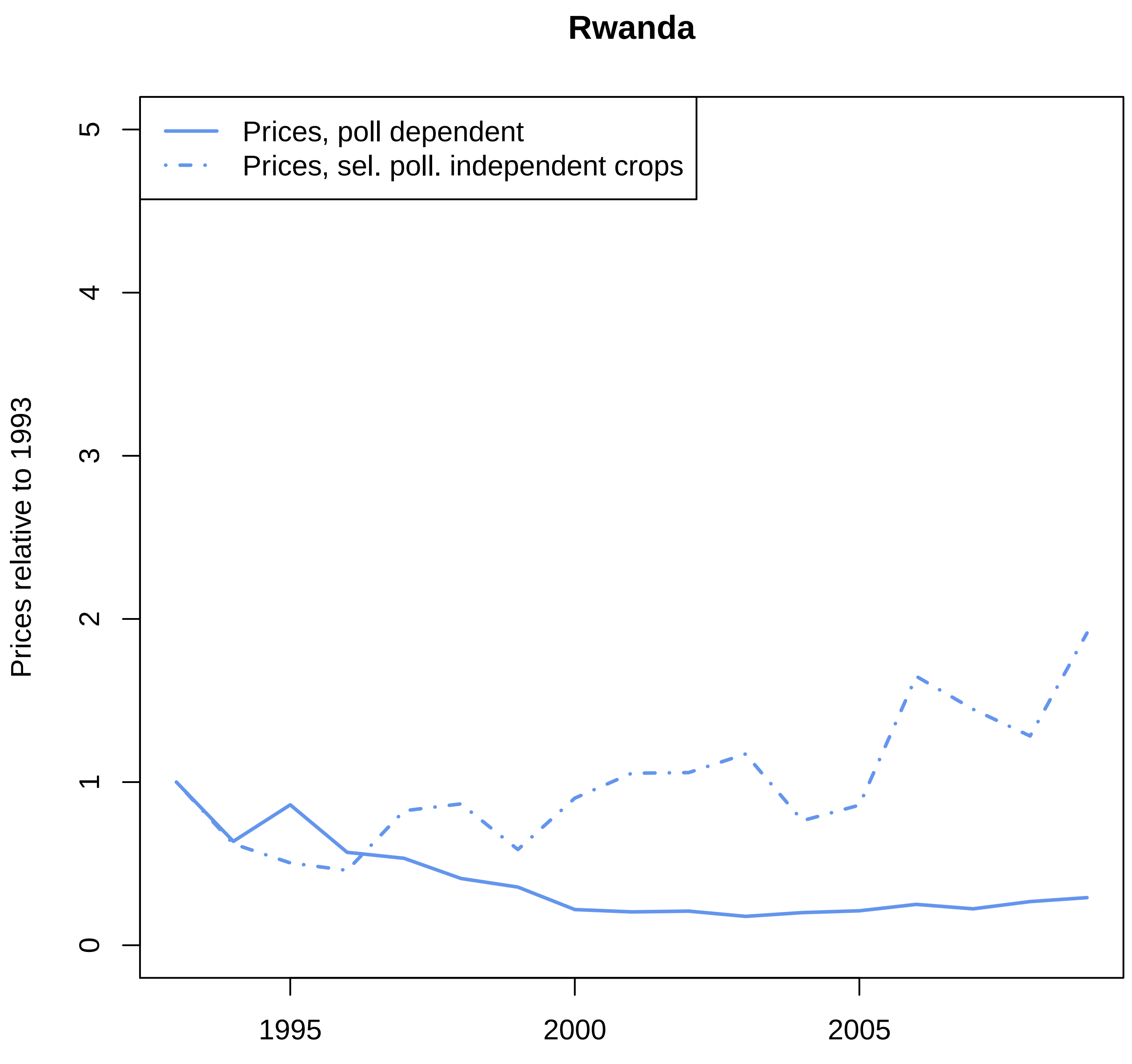
Rwanda
Prices, poll dependent
Prices, sel. poll. independent crops
Prices relative to 1993
0
1
2
3
4
5
1995
2000
2005

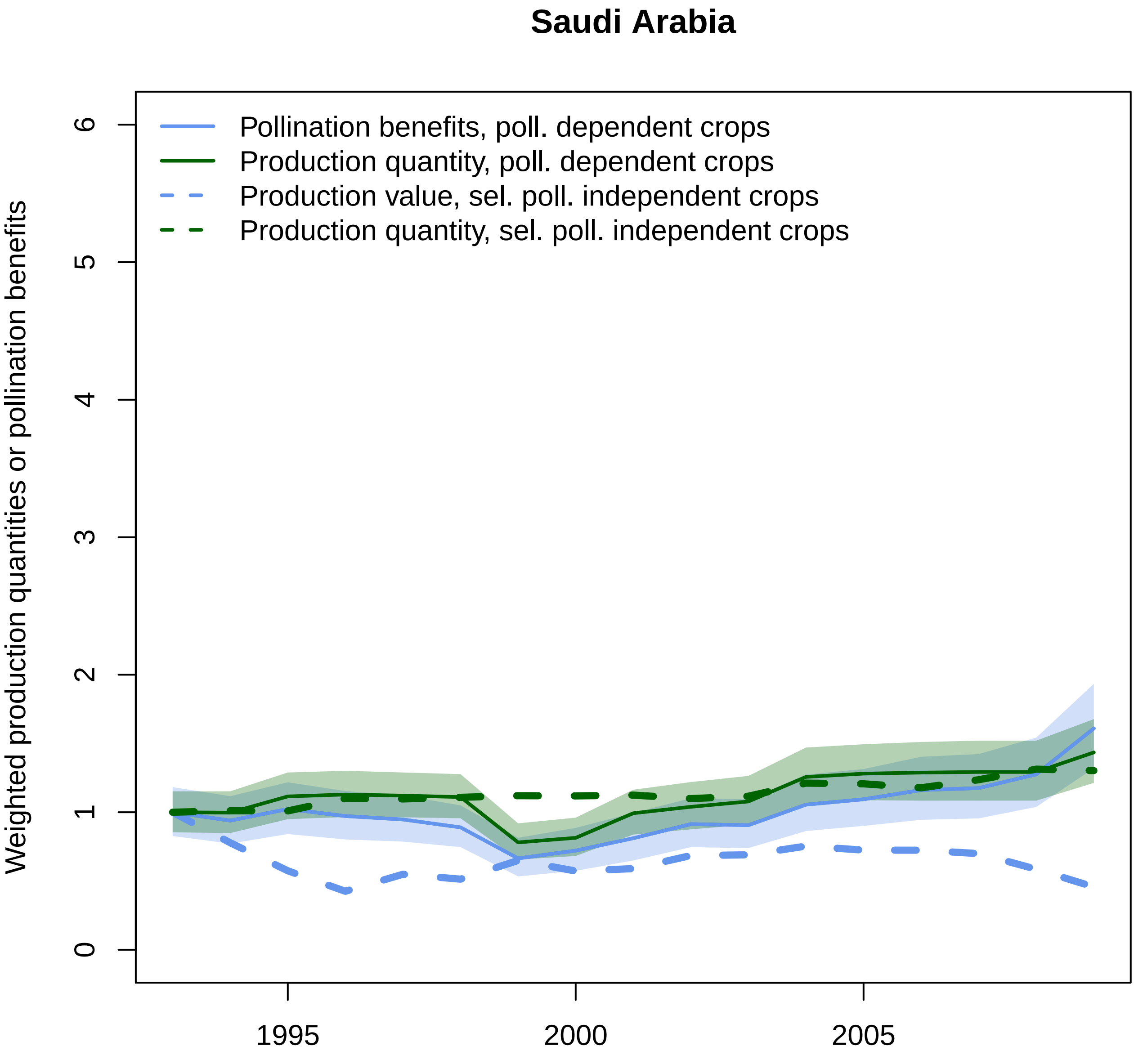

Saudi Arabia
Pollination benefits, poll. dependent crops
Production quantity, poll. dependent crops
Production value, sel. poll. independent crops
Production quantity, sel. poll. independent crops
Weighted production quantities or pollination benefits
0
1
2
3
4
5
6
1995
2000
2005

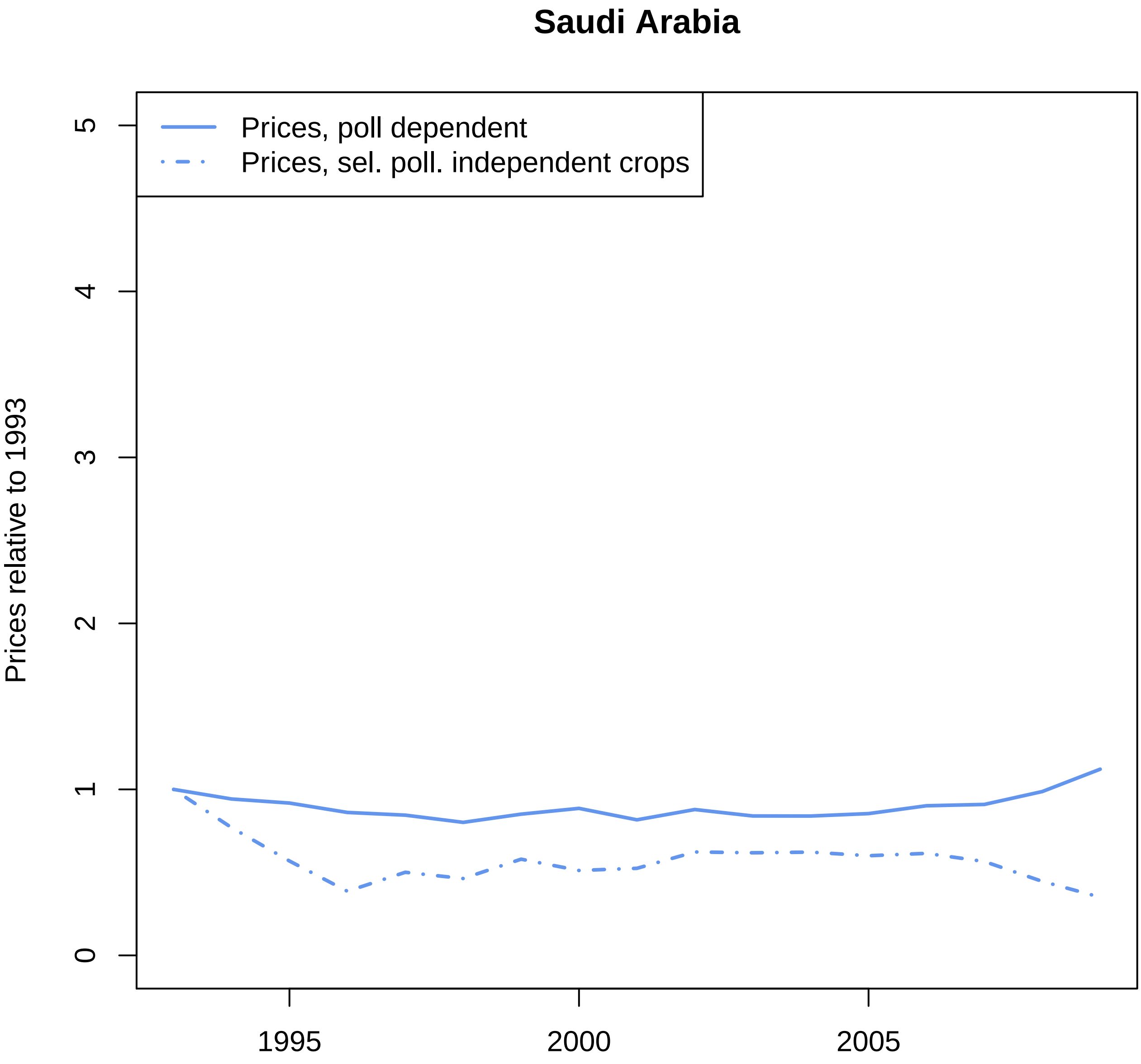

Saudi Arabia
Prices, poll dependent
Prices, sel. poll. independent crops
Prices relative to 1993
0
1
2
3
4
5
1995
2000
2005

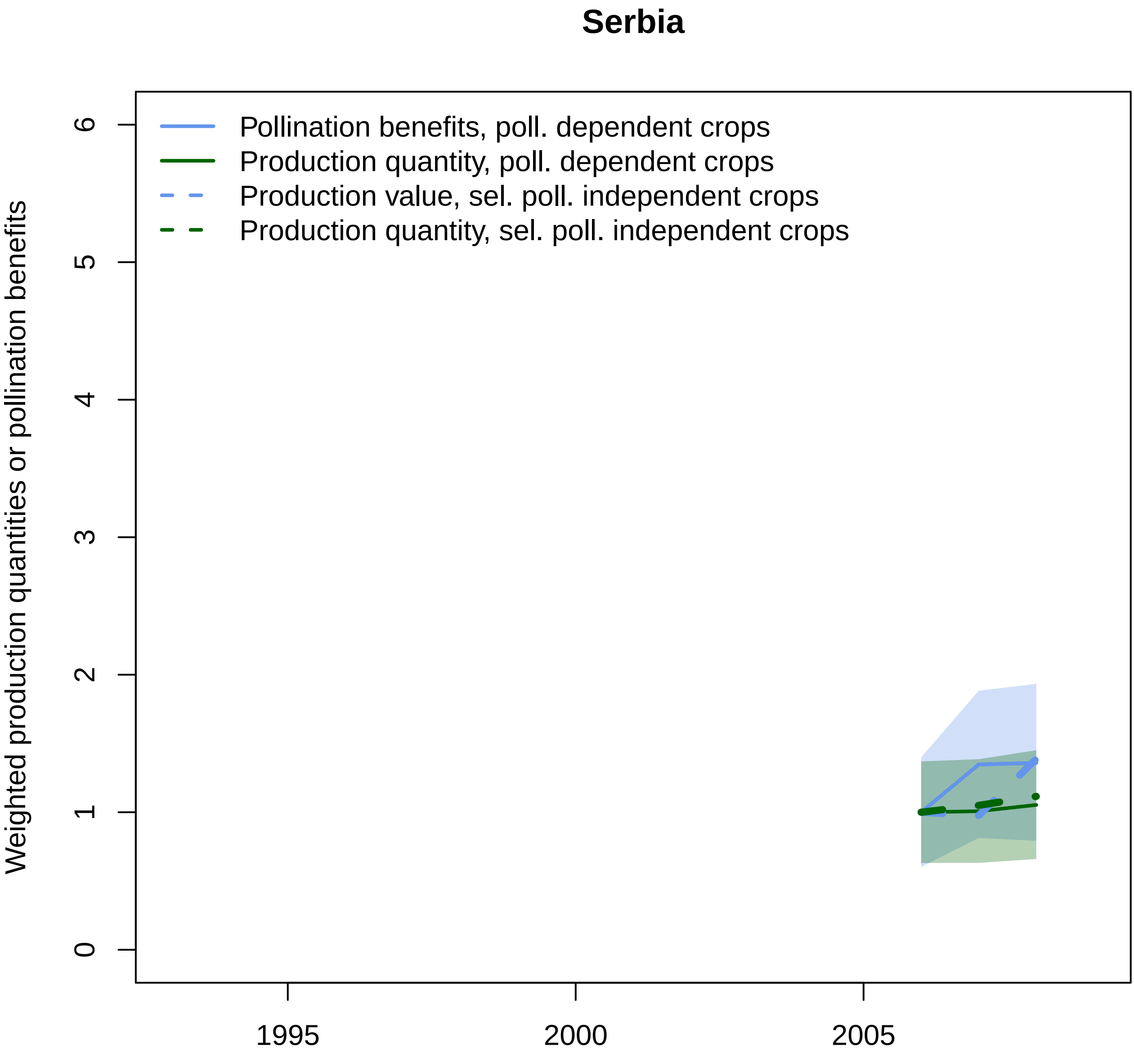
Serbia
Pollination benefits, poll. dependent crops
Production quantity, poll. dependent crops
Production value, sel. poll. independent crops
Production quantity, sel. poll. independent crops
Weighted production quantities or pollination benefits
0
1
2
3
4
5
6
1995
2000
2005

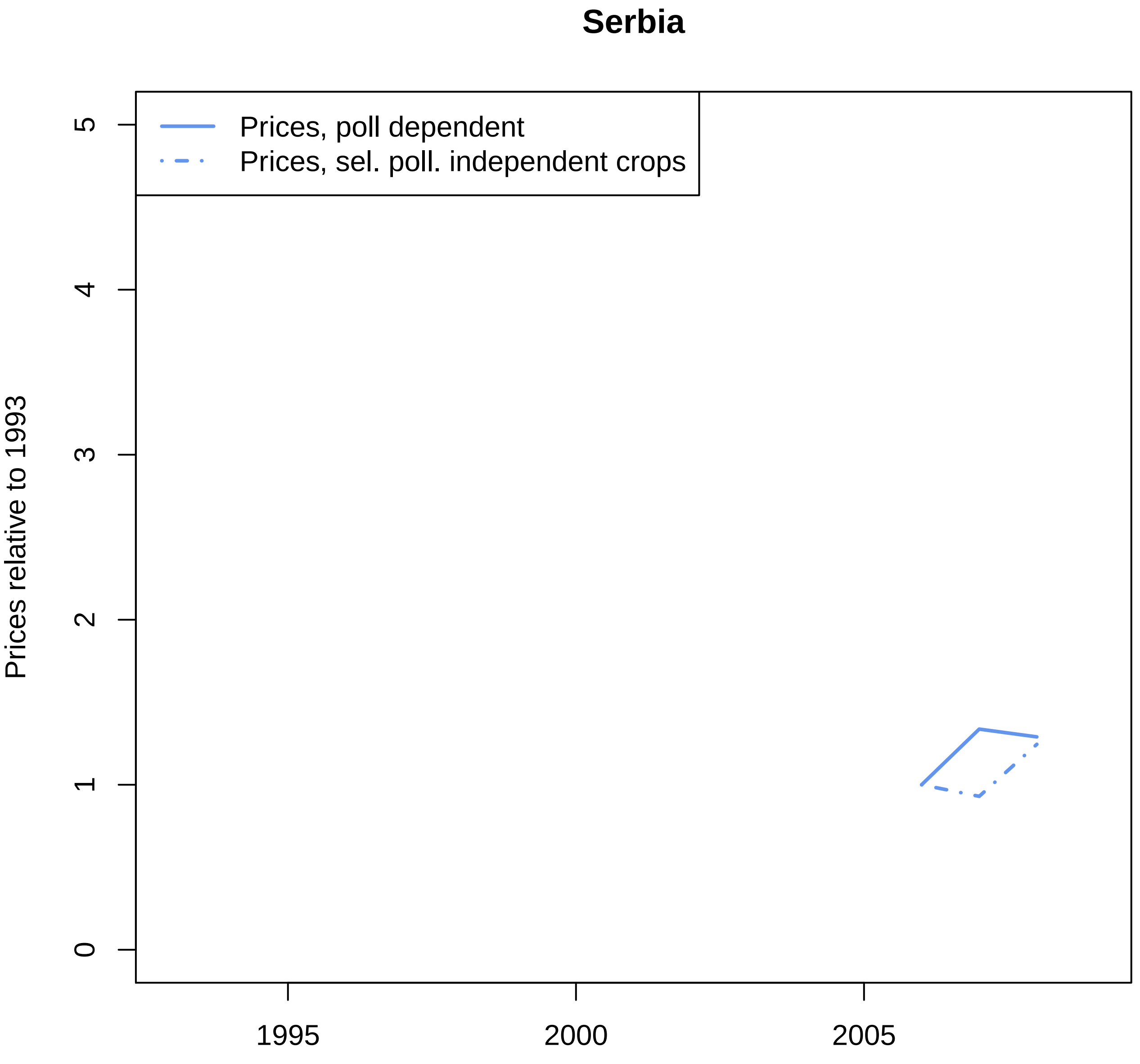
Serbia
Prices, poll dependent
Prices, sel. poll. independent crops
Prices relative to 1993
0
1
2
3
4
5
1995
2000
2005

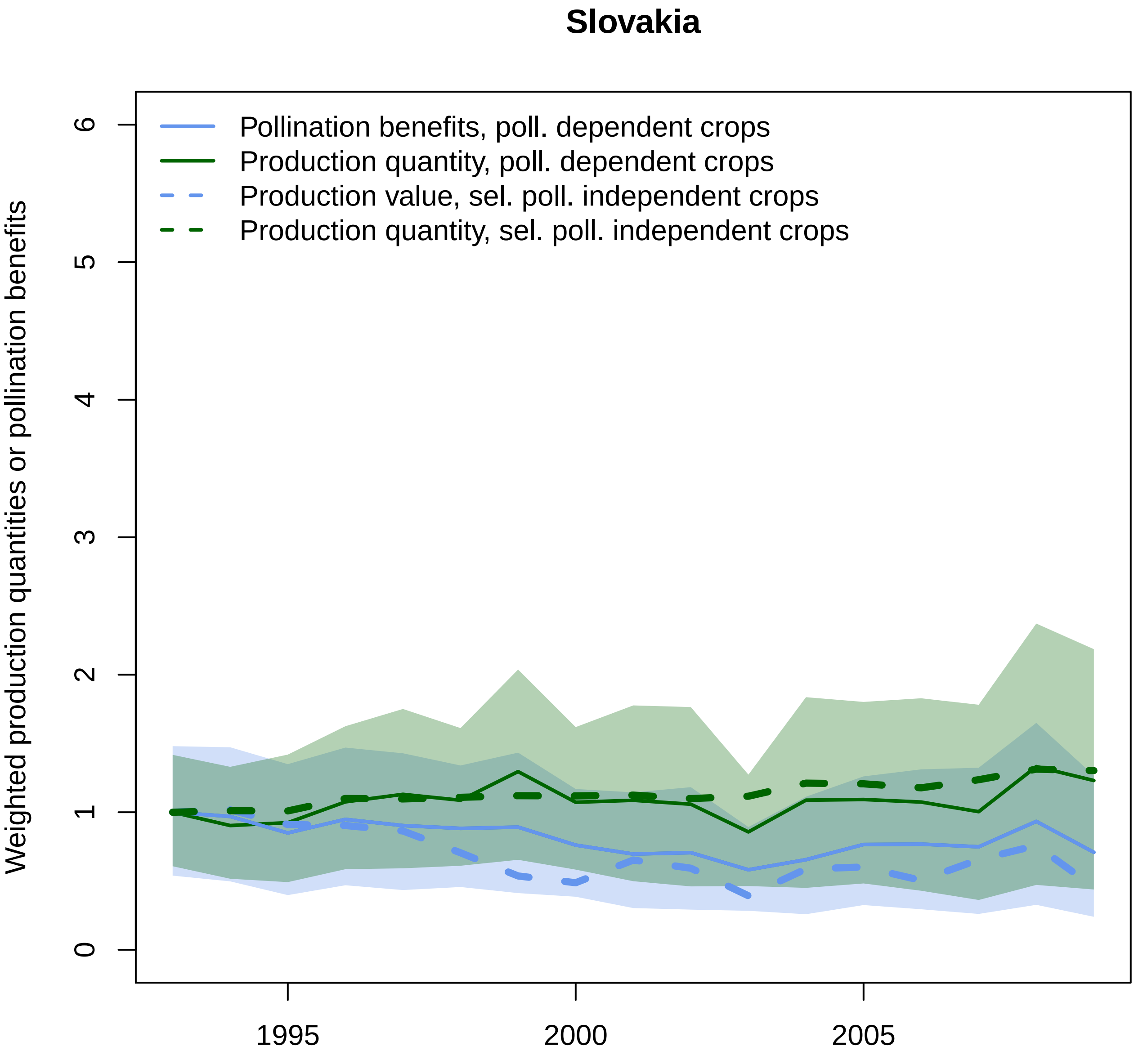
Slovakia
Pollination benefits, poll. dependent crops
Production quantity, poll. dependent crops
Production value, sel. poll. independent crops
Production quantity, sel. poll. independent crops
Weighted production quantities or pollination benefits
0
1
2
3
4
5
6
1995
2000
2005

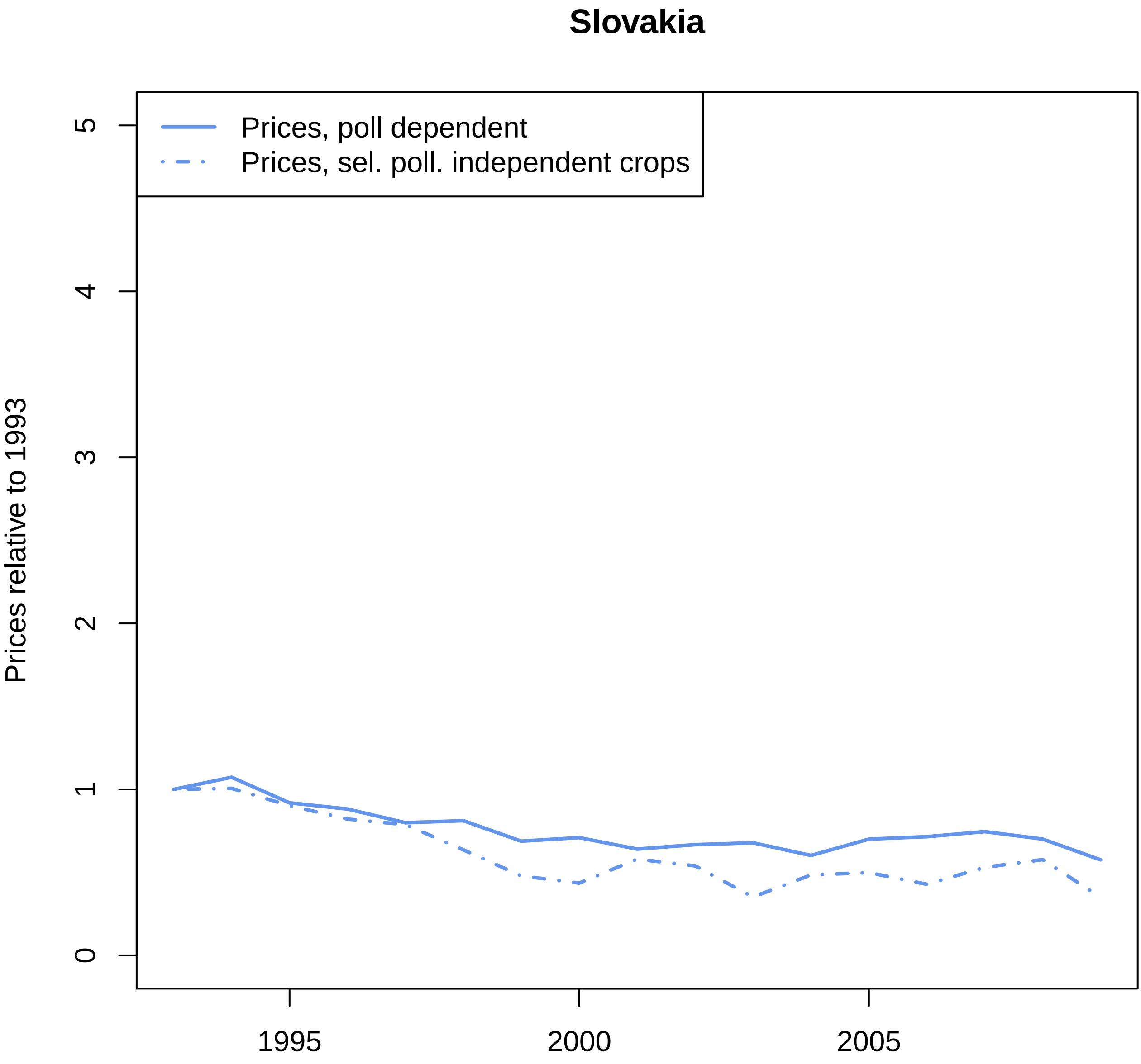

Slovakia
Prices, poll dependent
Prices, sel. poll. independent crops
Prices relative to 1993
0
1
2
3
4
5
1995
2000
2005

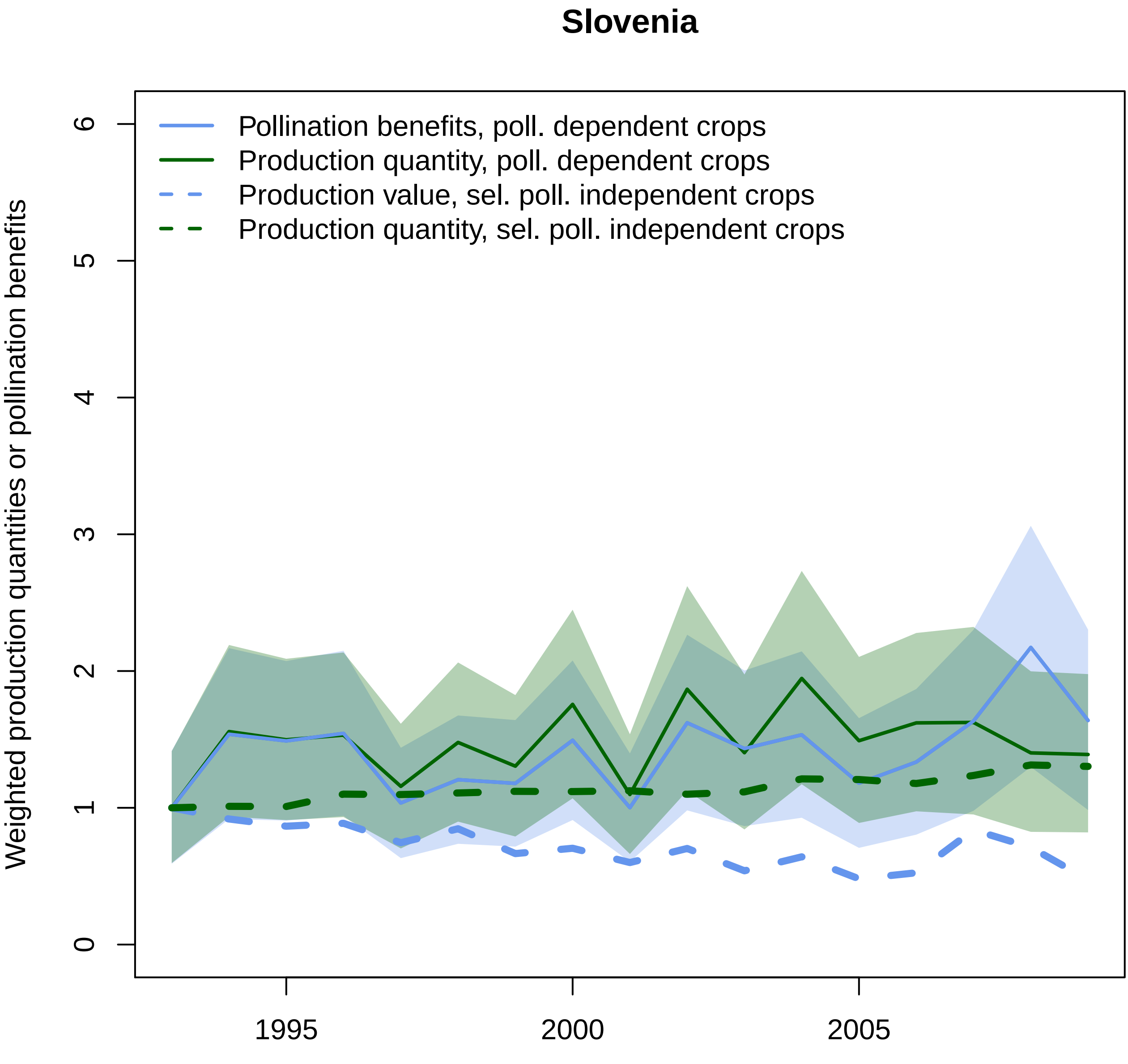

Slovenia
Pollination benefits, poll. dependent crops
Production quantity, poll. dependent crops
Production value, sel. poll. independent crops
Production quantity, sel. poll. independent crops
Weighted production quantities or pollination benefits
0
1
2
3
4
5
6
1995
2000
2005

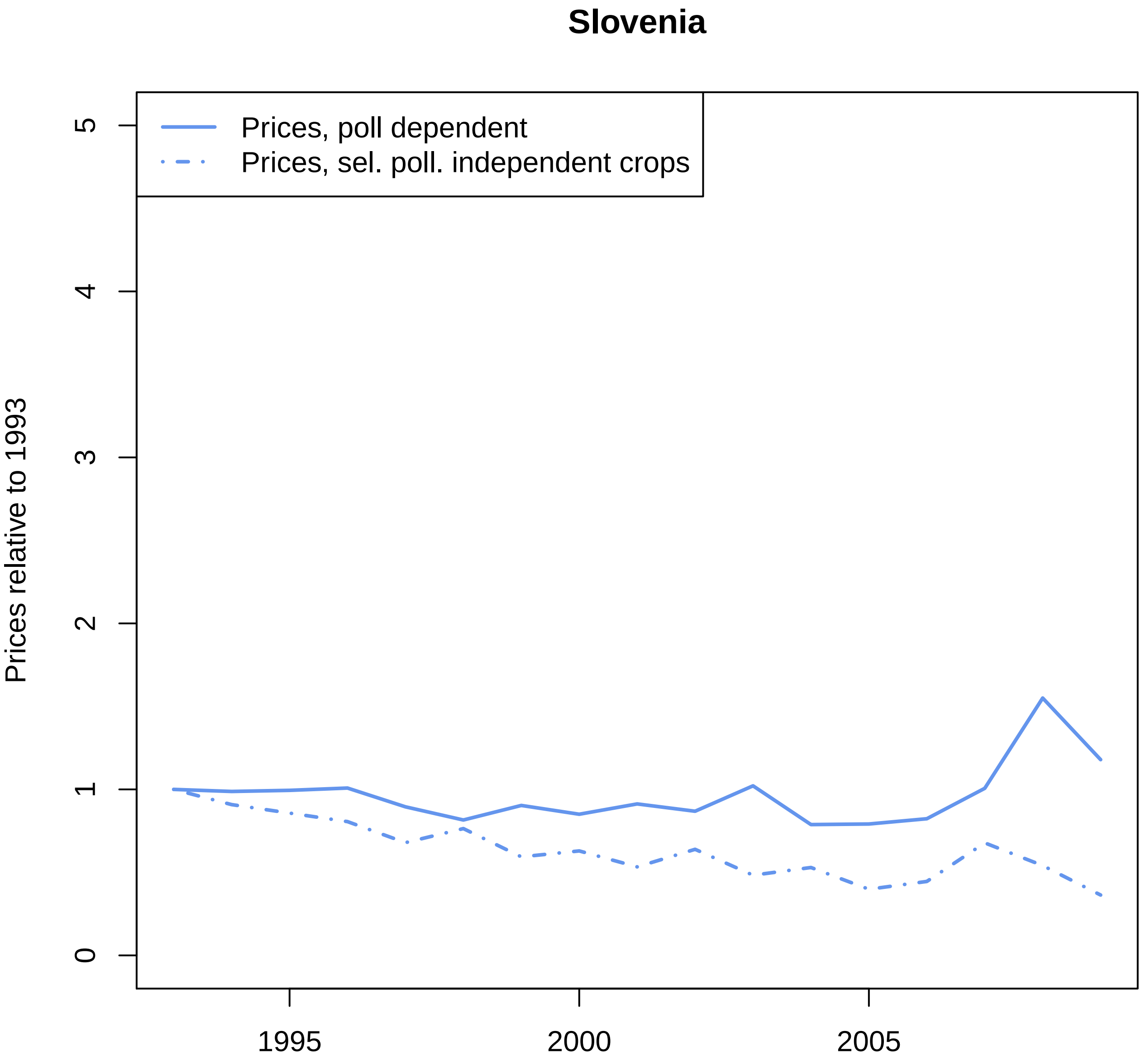
Slovenia
Prices, poll dependent
Prices, sel. poll. independent crops
Prices relative to 1993
0
1
2
3
4
5
1995
2000
2005

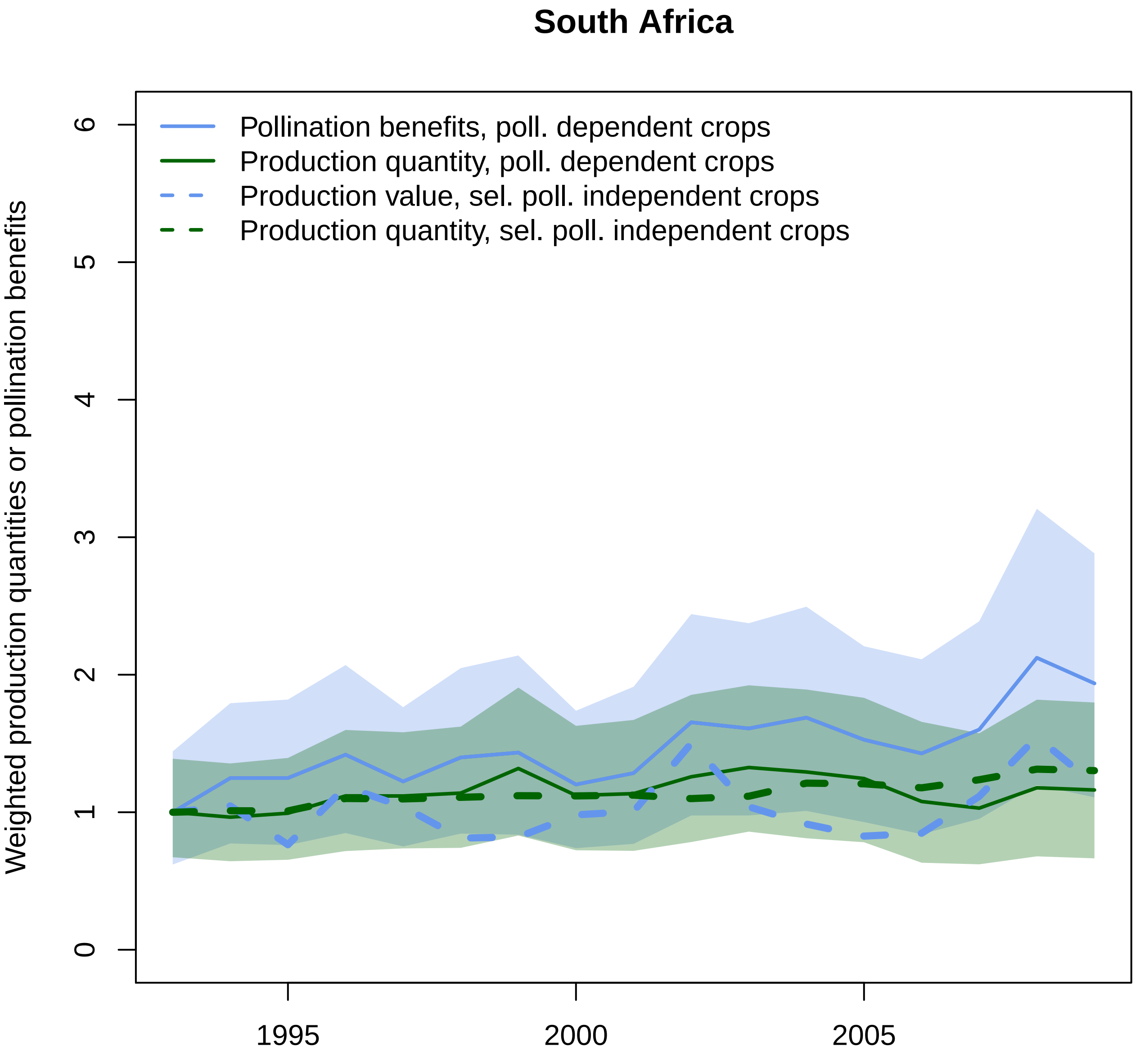

South Africa
Pollination benefits, poll. dependent crops
Production quantity, poll. dependent crops
Production value, sel. poll. independent crops
Production quantity, sel. poll. independent crops
Weighted production quantities or pollination benefits
0
1
2
3
4
5
6
1995
2000
2005

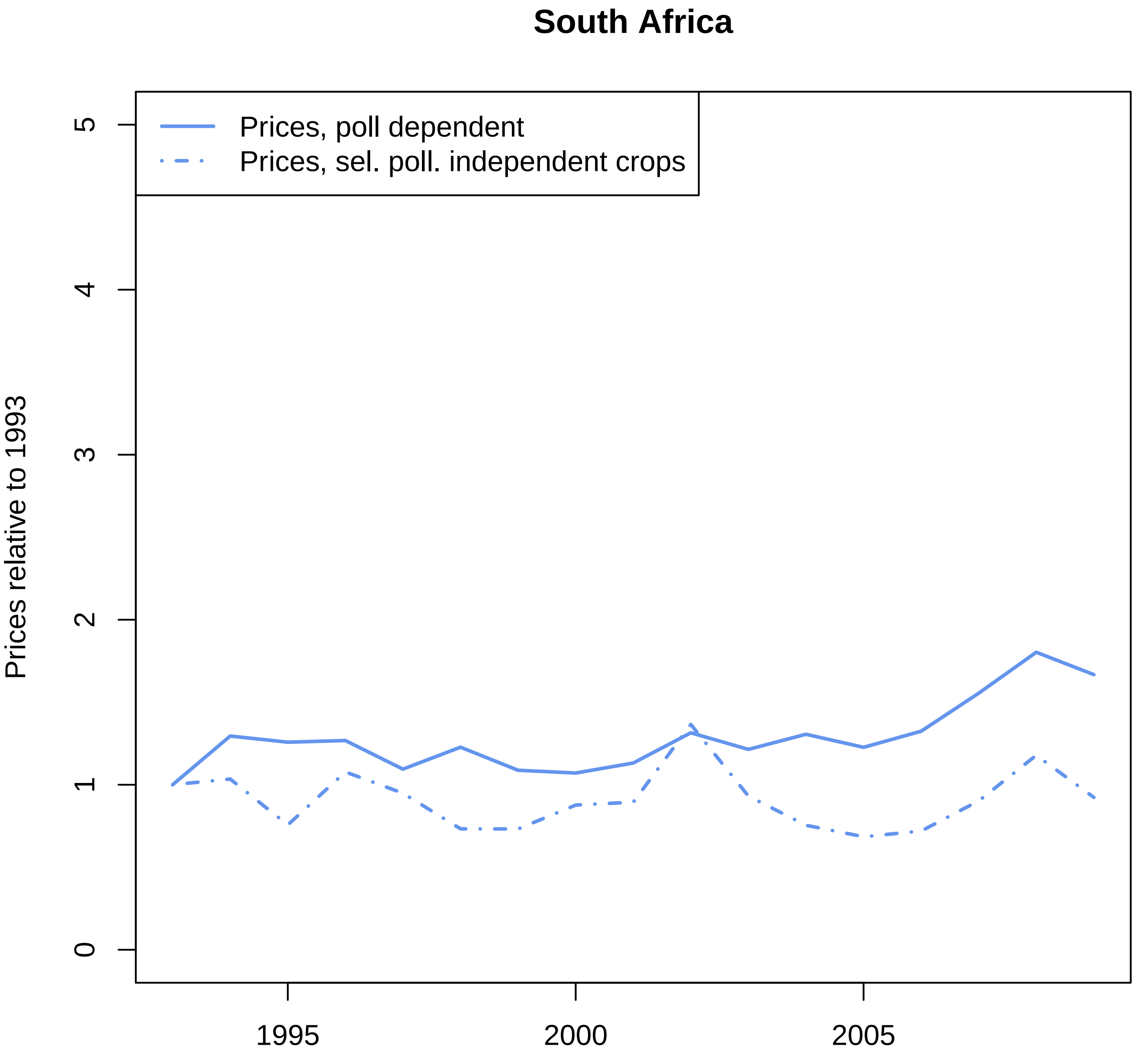
South Africa
Prices, poll dependent
Prices, sel. poll. independent crops
Prices relative to 1993
0
1
2
3
4
5
1995
2000
2005

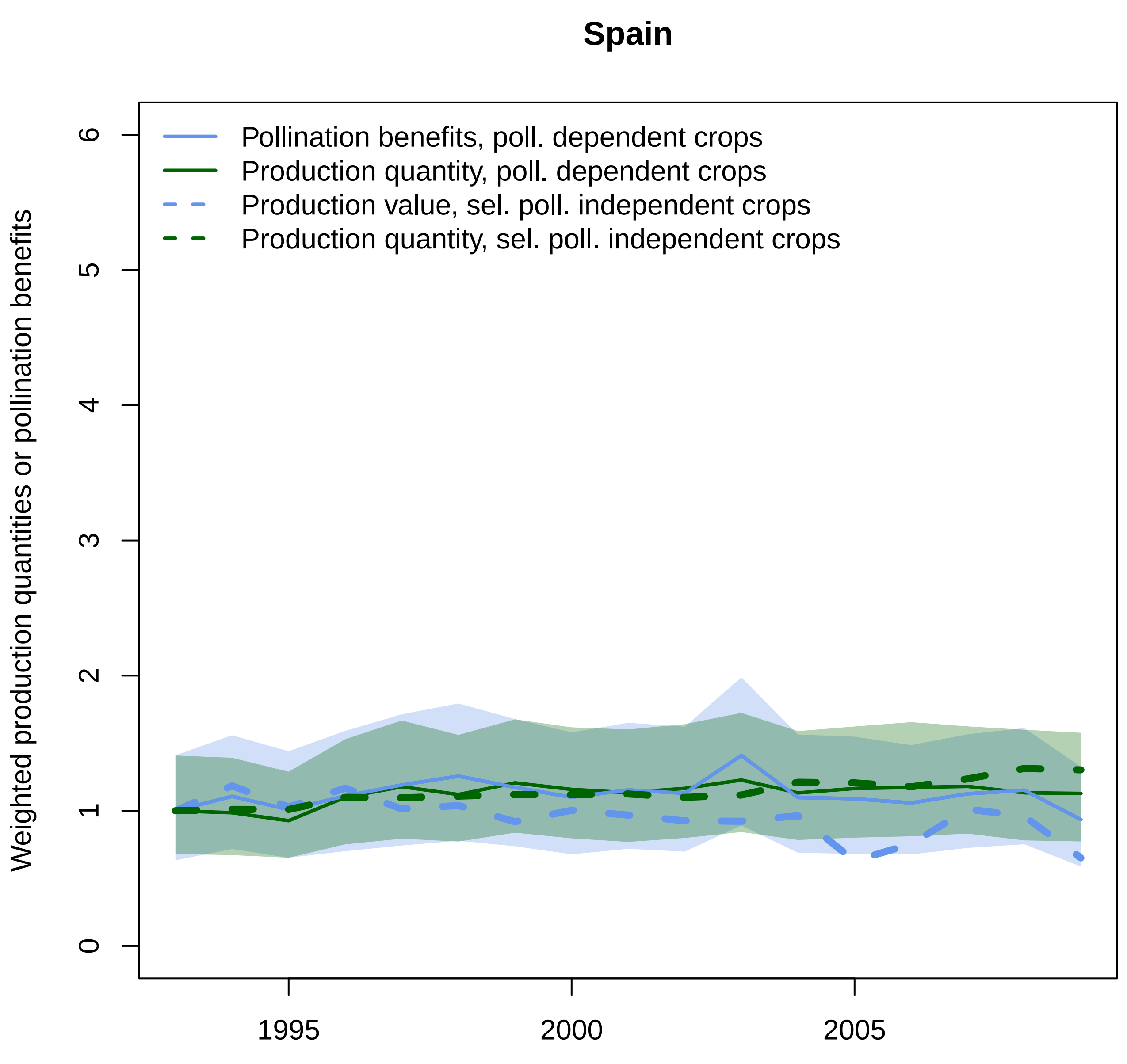
Spain
Pollination benefits, poll. dependent crops
Production quantity, poll. dependent crops
Production value, sel. poll. independent crops
Production quantity, sel. poll. independent crops
Weighted production quantities or pollination benefits
0
1
2
3
4
5
6
1995
2000
2005

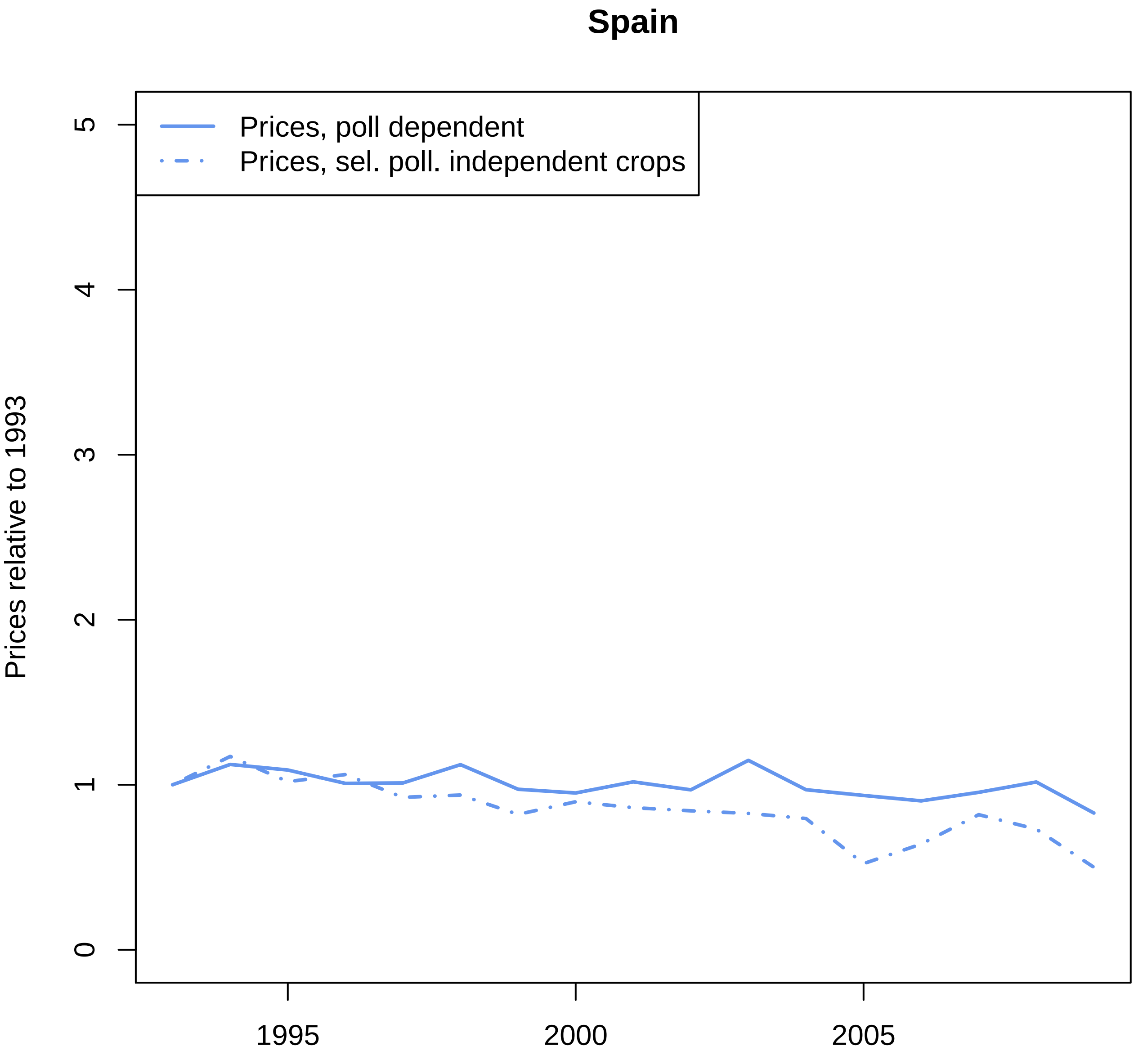
Spain
Prices, poll dependent
Prices, sel. poll. independent crops
Prices relative to 1993
0
1
2
3
4
5
1995
2000
2005

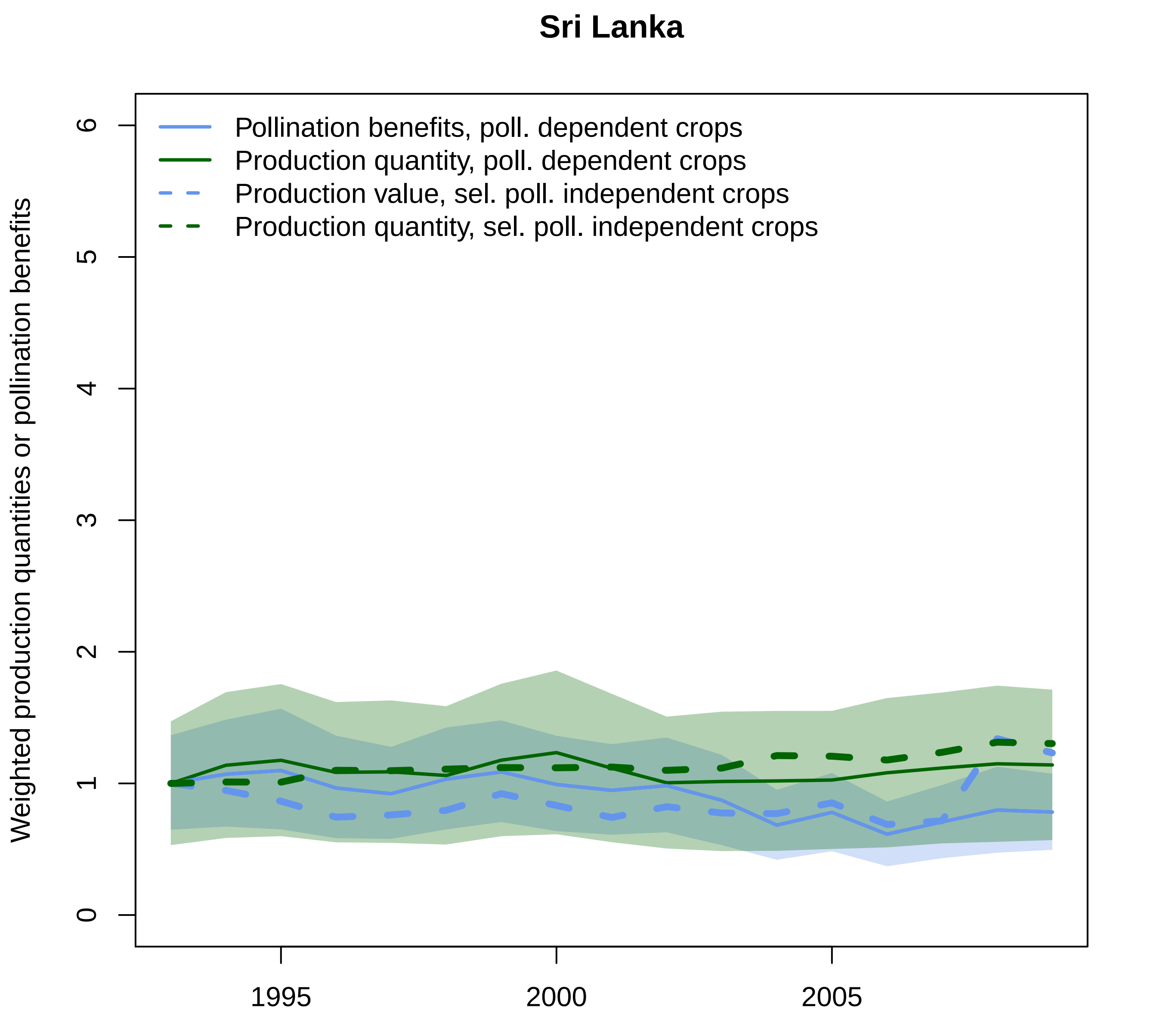
Sri Lanka
Pollination benefits, poll. dependent crops
Production quantity, poll. dependent crops
Production value, sel. poll. independent crops
Production quantity, sel. poll. independent crops
Weighted production quantities or pollination benefits
0
1
2
3
4
5
6
1995
2000
2005

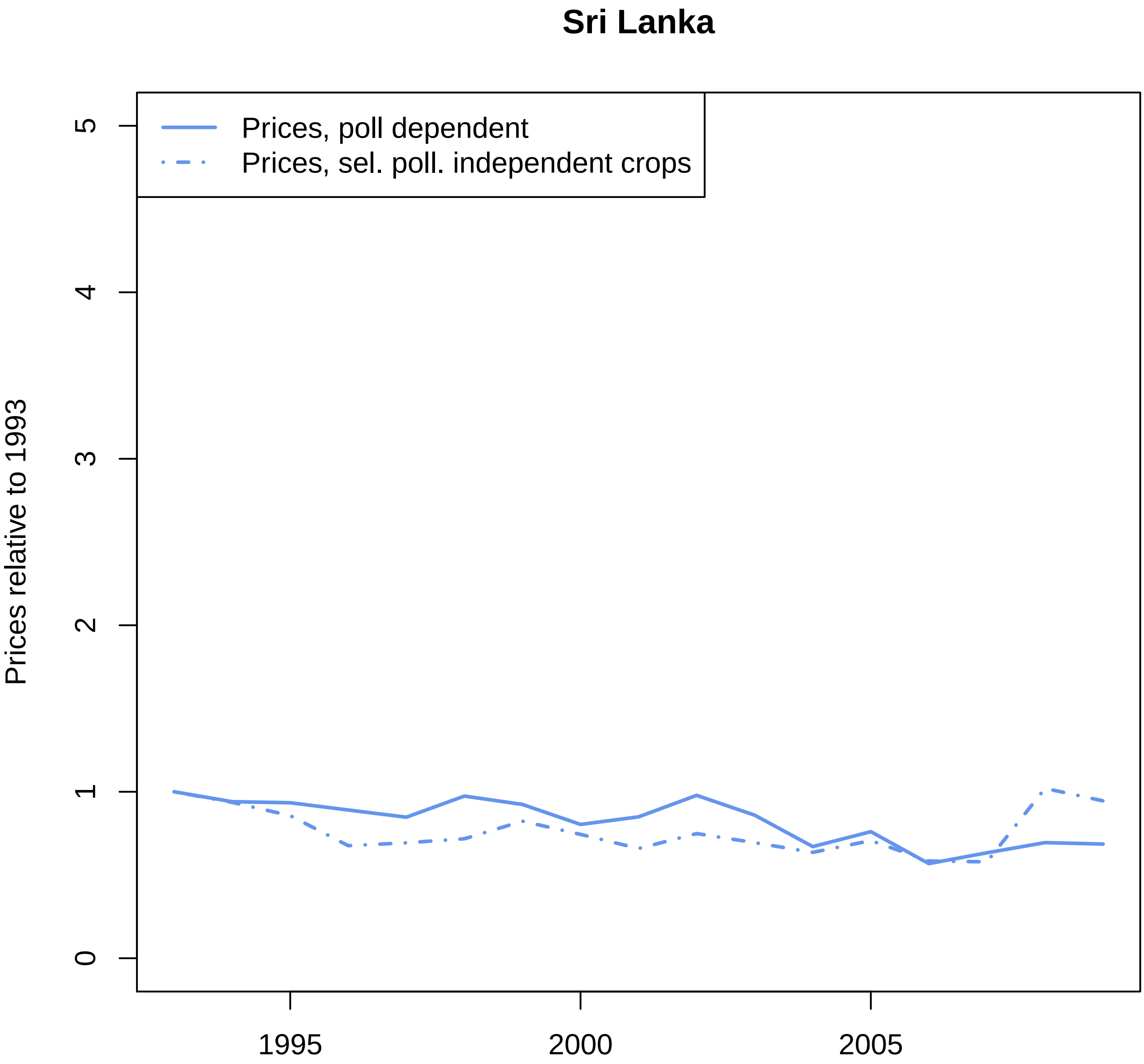

Sri Lanka
Prices, poll dependent
Prices, sel. poll. independent crops
Prices relative to 1993
0
1
2
3
4
5
1995
2000
2005

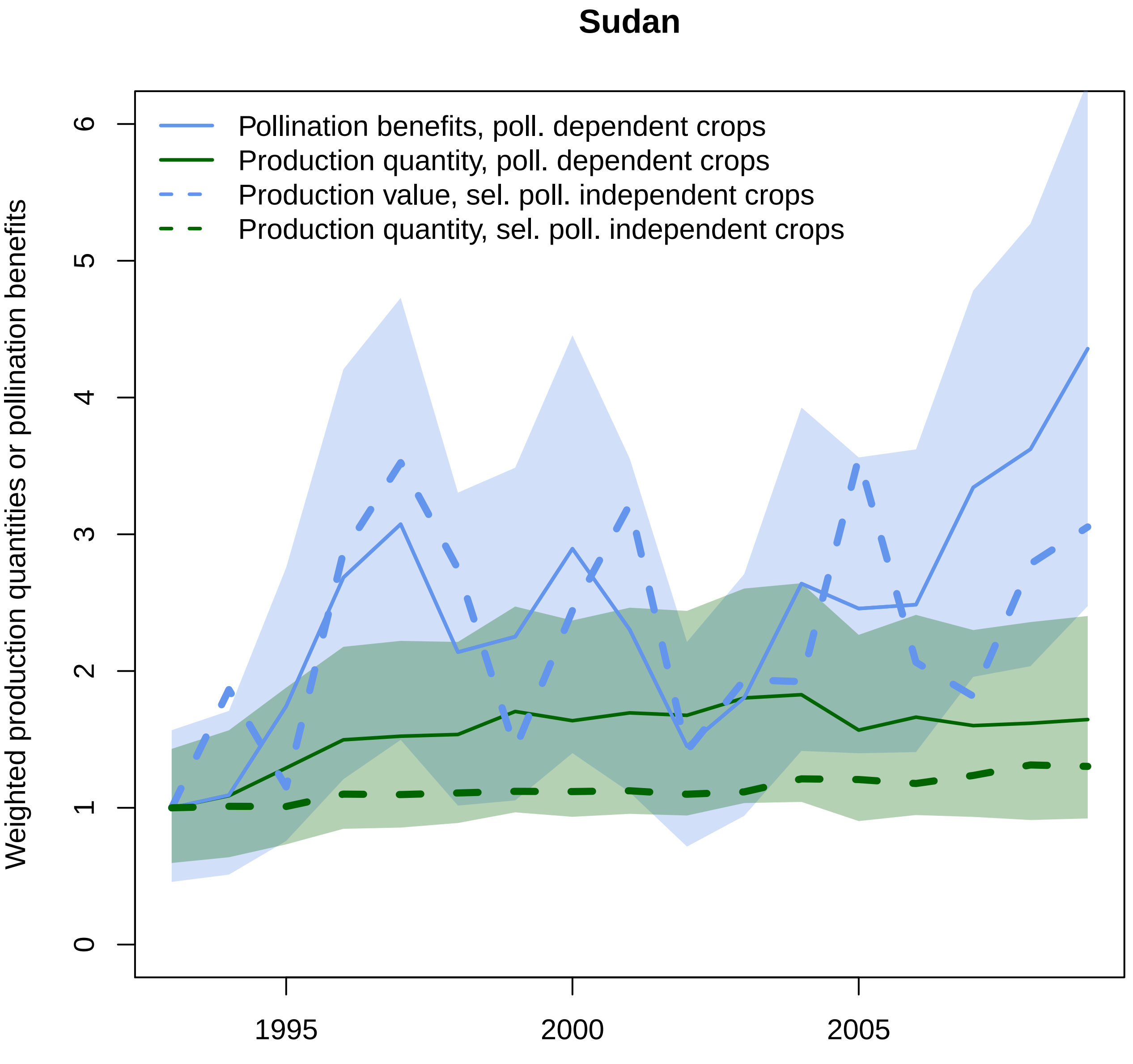

Sudan
Pollination benefits, poll. dependent crops
Production quantity, poll. dependent crops
Production value, sel. poll. independent crops
Production quantity, sel. poll. independent crops
Weighted production quantities or pollination benefits
0
1
2
3
4
5
6
1995
2000
2005

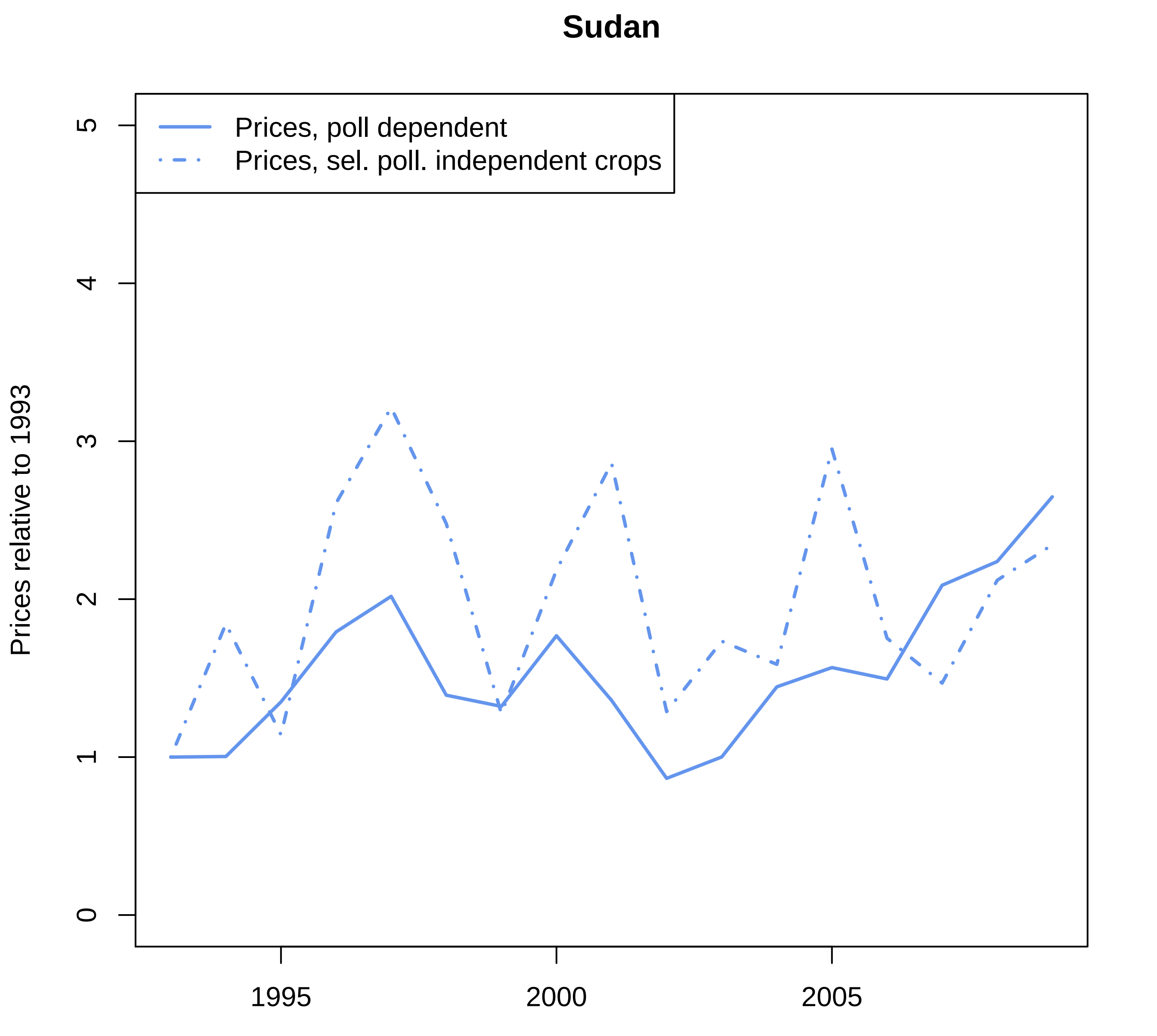

Sudan
Prices, poll dependent
Prices, sel. poll. independent crops
Prices relative to 1993
0
1
2
3
4
5
1995
2000
2005

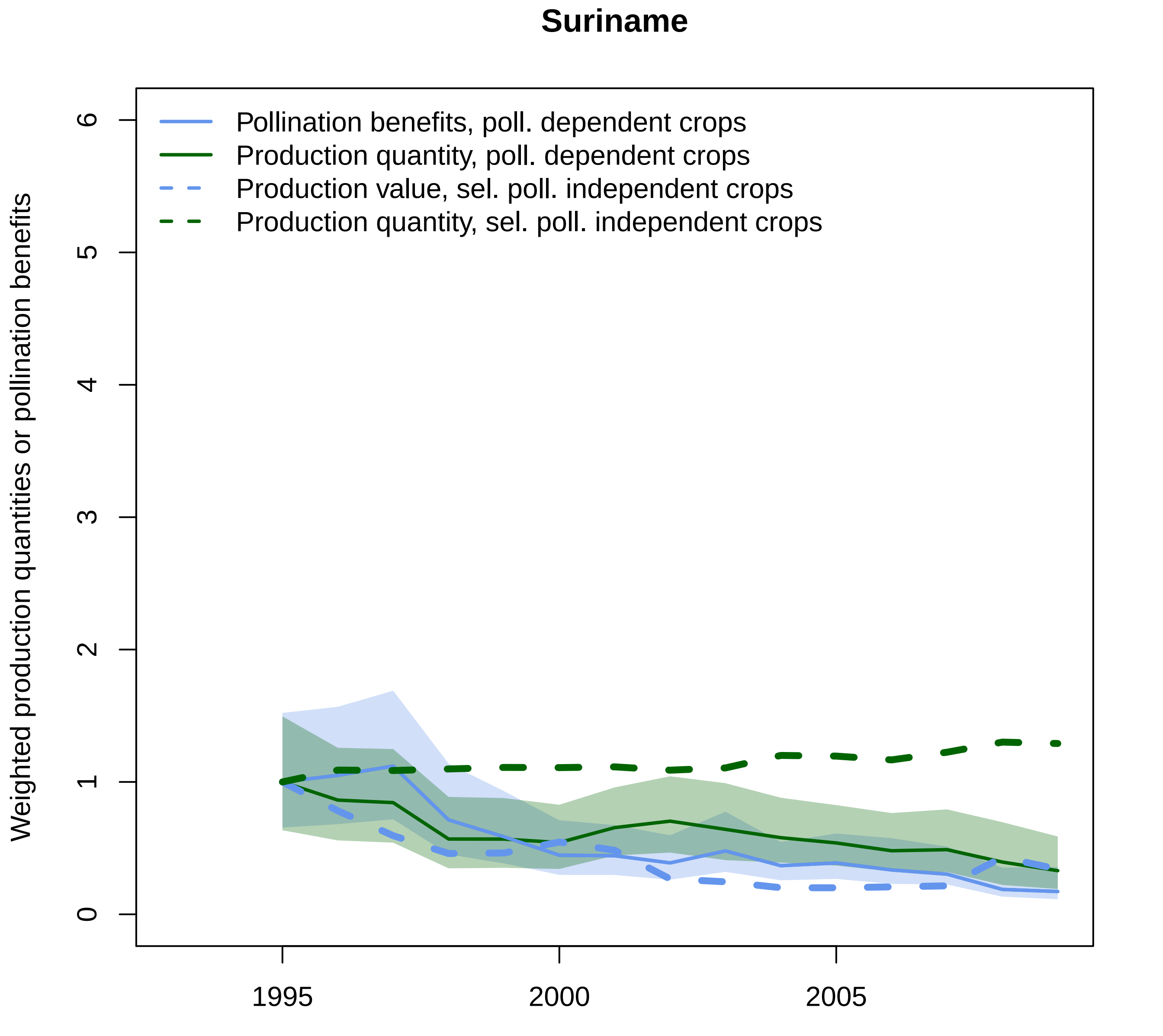
Suriname
Pollination benefits, poll. dependent crops
Production quantity, poll. dependent crops
Production value, sel. poll. independent crops
Production quantity, sel. poll. independent crops
Weighted production quantities or pollination benefits
0
1
2
3
4
5
6
1995
2000
2005

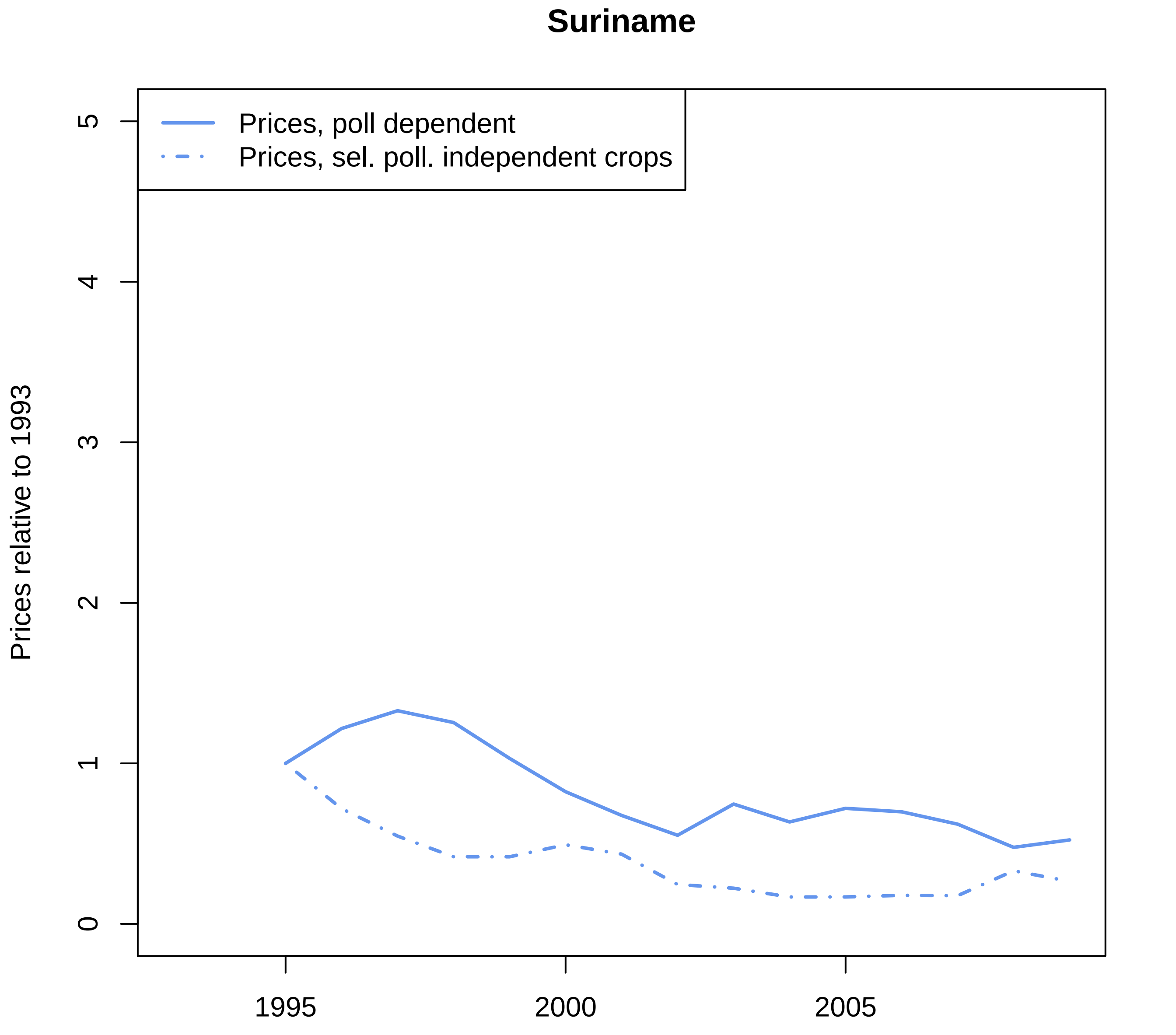

Suriname
Prices, poll dependent
Prices, sel. poll. independent crops
Prices relative to 1993
0
1
2
3
4
5
1995
2000
2005

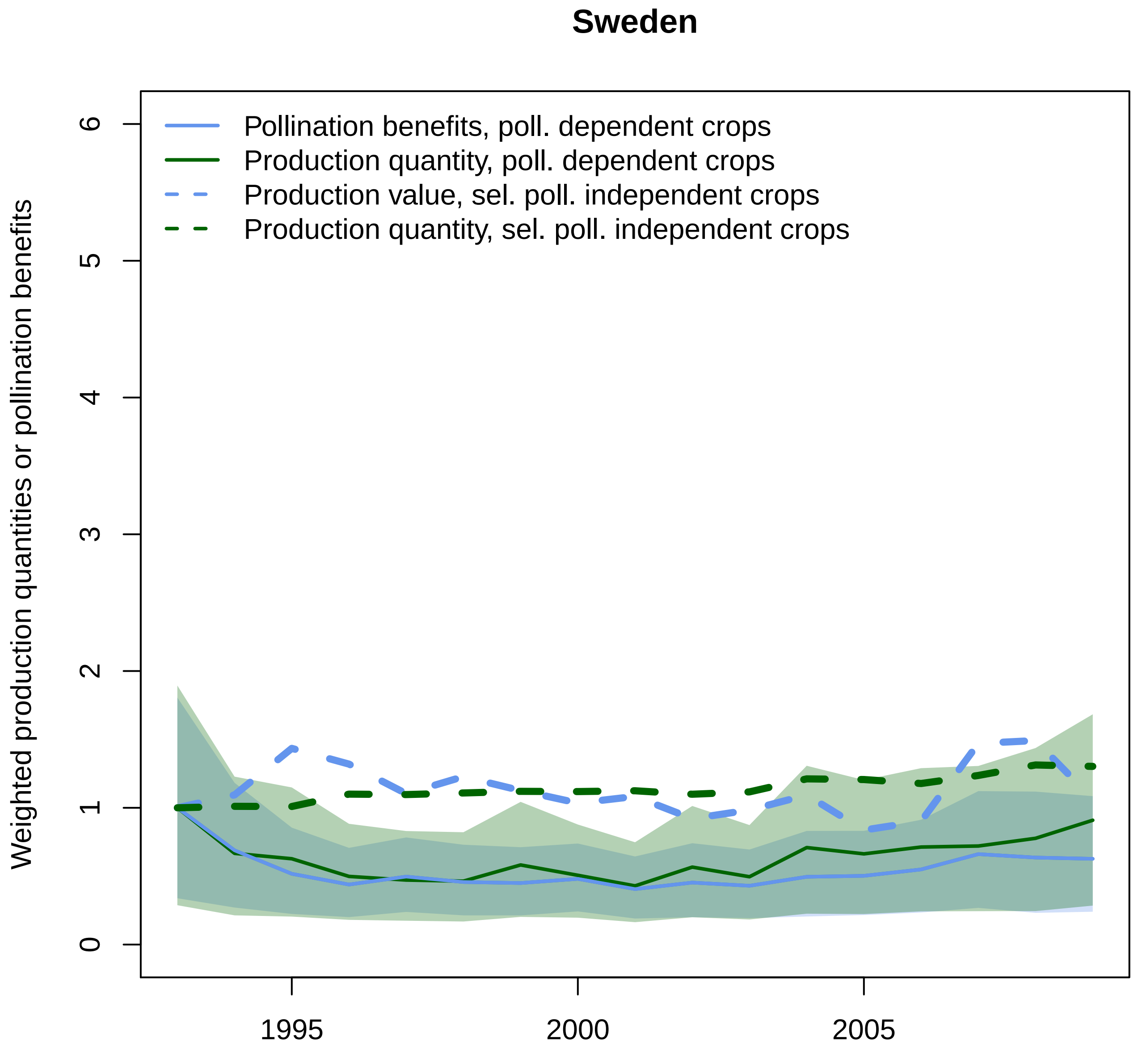
Sweden
Pollination benefits, poll. dependent crops
Production quantity, poll. dependent crops
Production value, sel. poll. independent crops
Production quantity, sel. poll. independent crops
Weighted production quantities or pollination benefits
0
1
2
3
4
5
6
1995
2000
2005

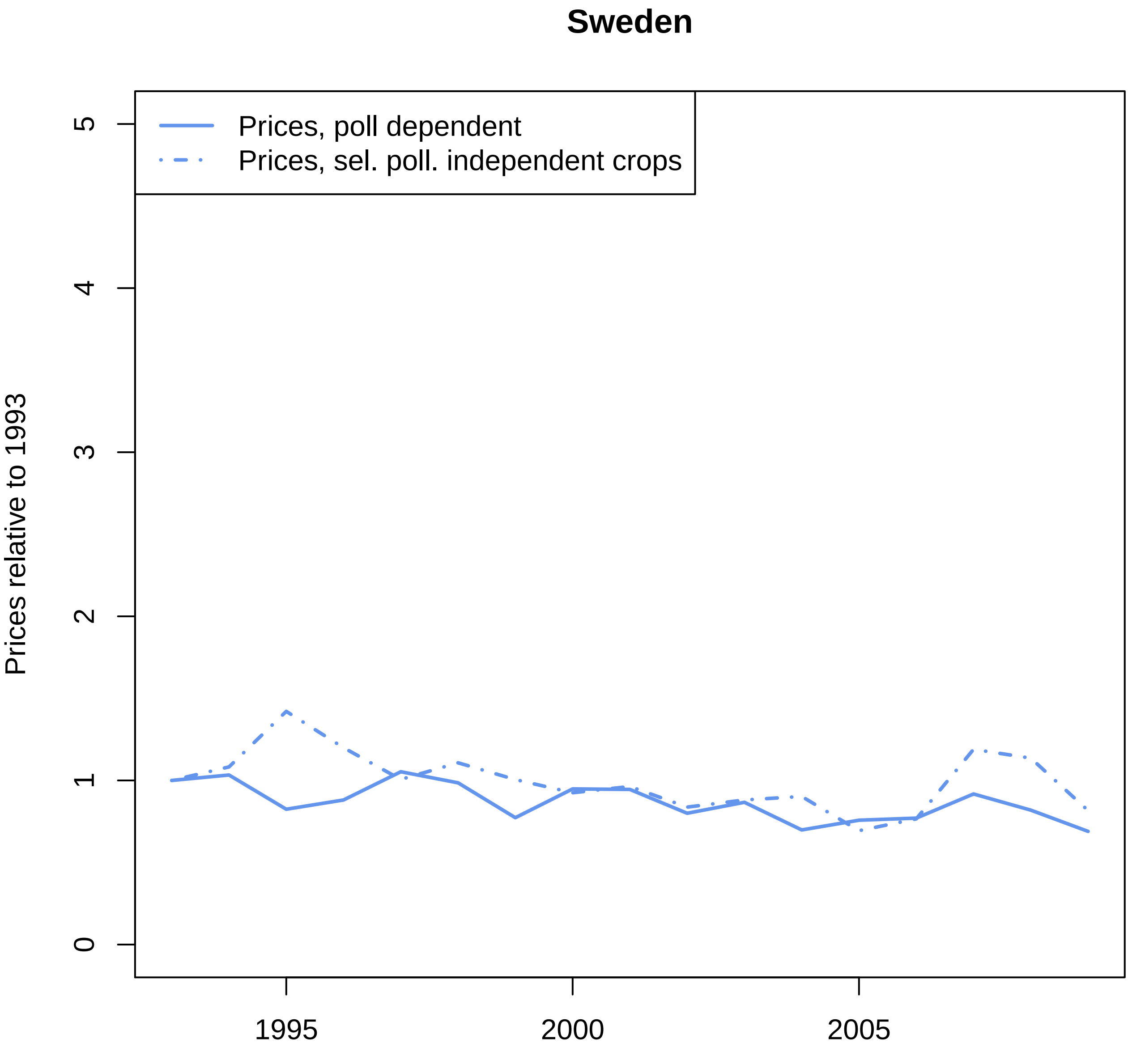
Sweden
Prices, poll dependent
Prices, sel. poll. independent crops
Prices relative to 1993
0
1
2
3
4
5
1995
2000
2005

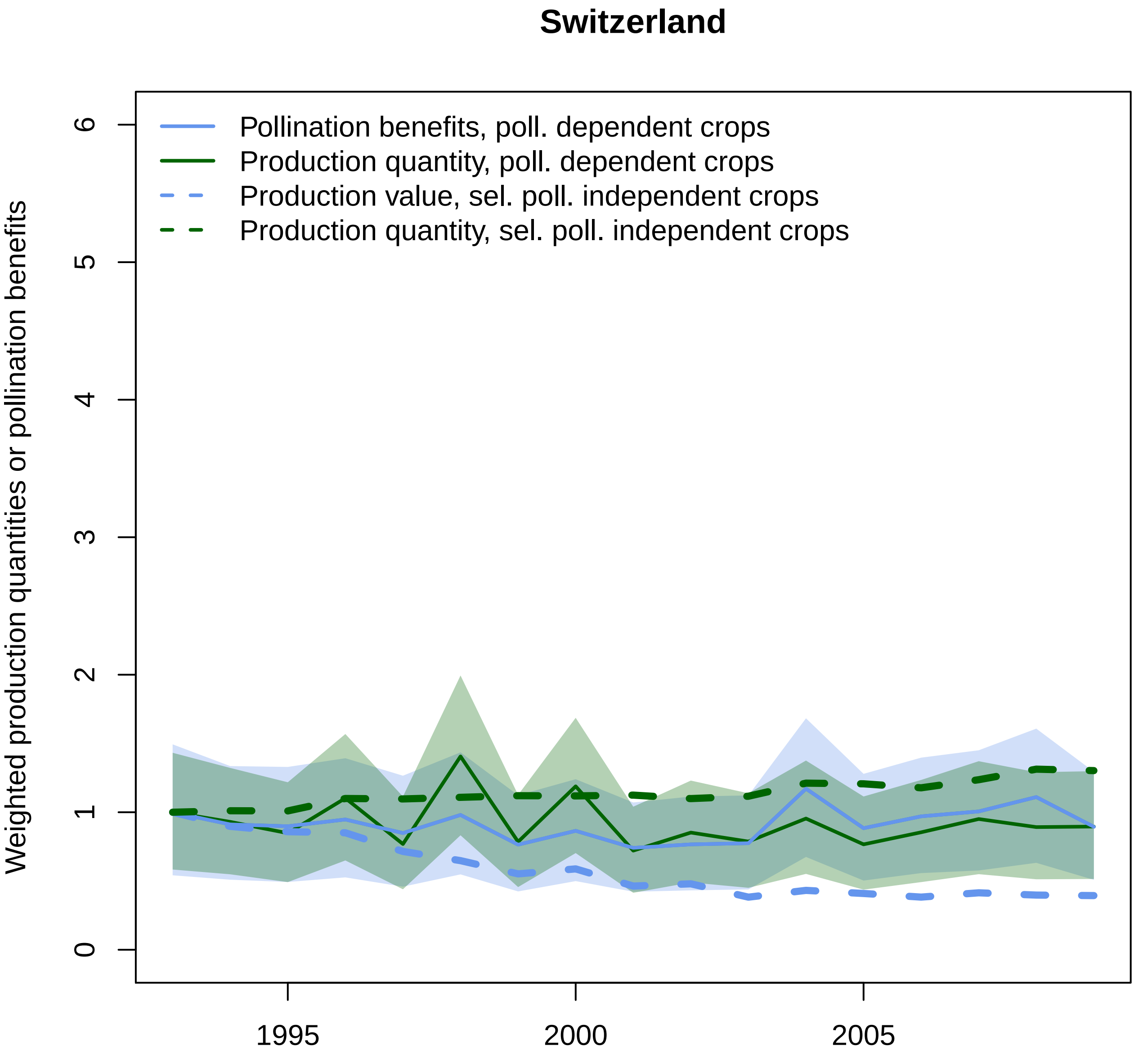

Switzerland
Pollination benefits, poll. dependent crops
Production quantity, poll. dependent crops
Production value, sel. poll. independent crops
Production quantity, sel. poll. independent crops
Weighted production quantities or pollination benefits
0
1
2
3
4
5
6
1995
2000
2005

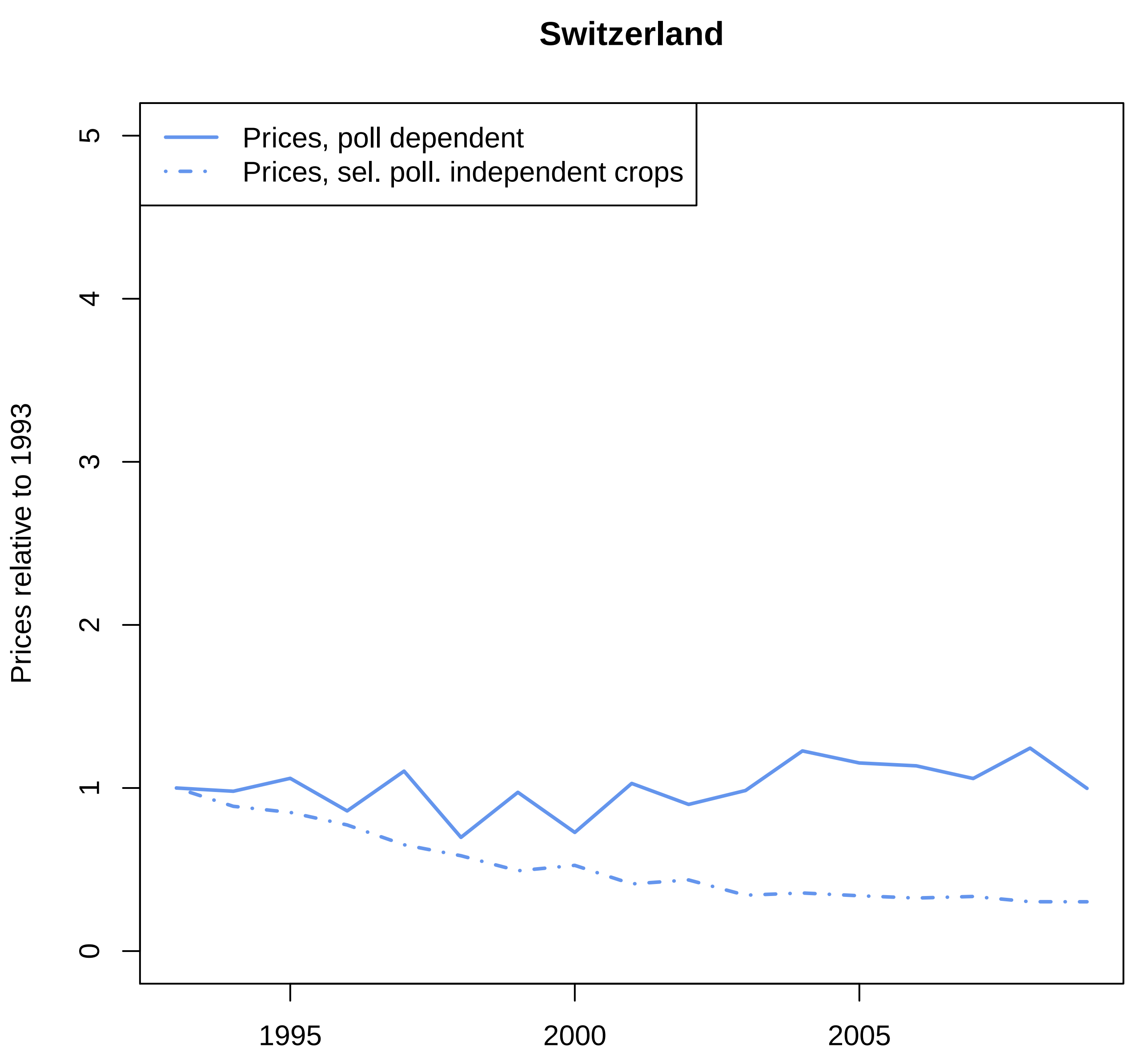
Switzerland
Prices, poll dependent
Prices, sel. poll. independent crops
Prices relative to 1993
0
1
2
3
4
5
1995
2000
2005

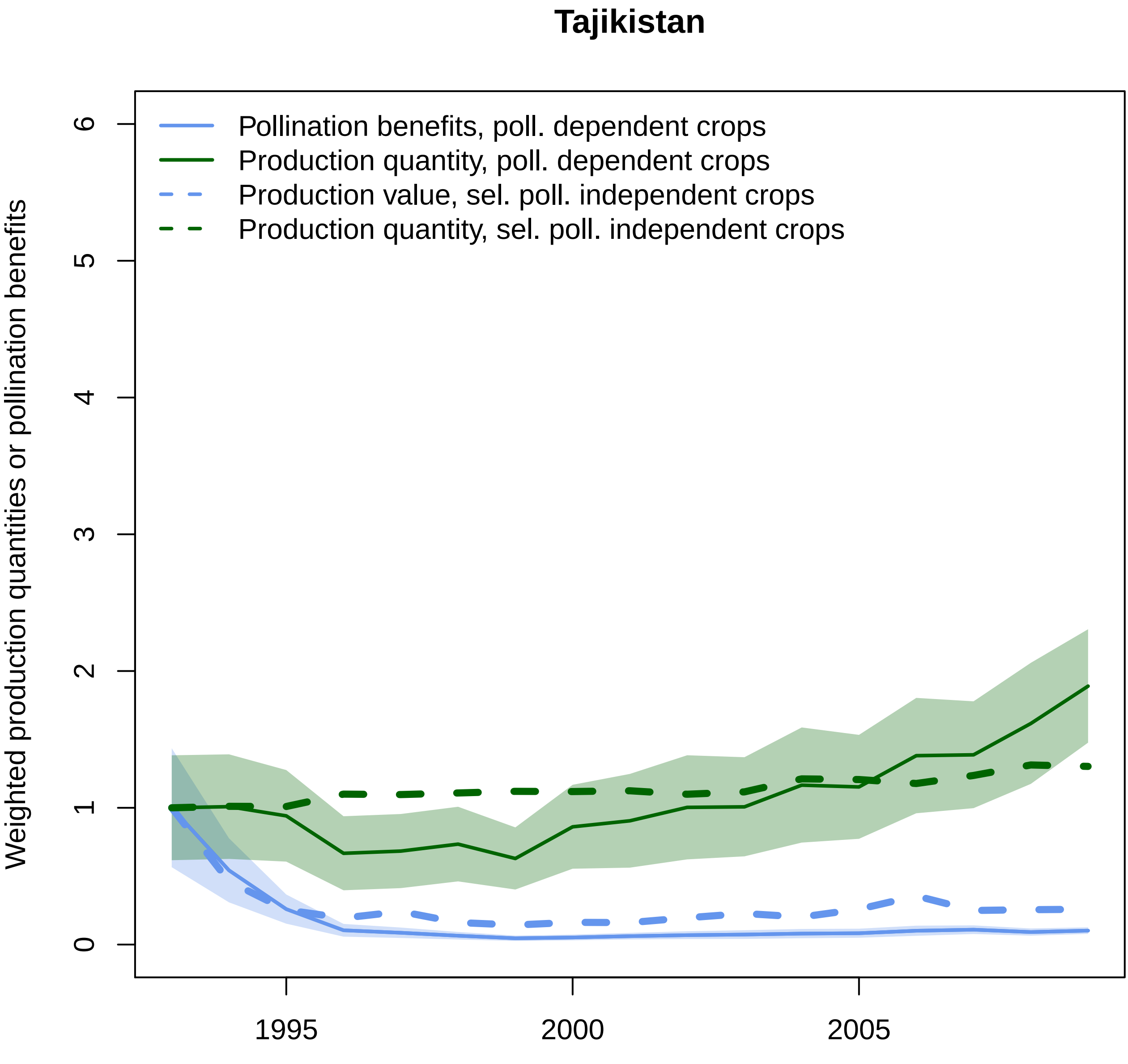

Tajikistan
Pollination benefits, poll. dependent crops
Production quantity, poll. dependent crops
Production value, sel. poll. independent crops
Production quantity, sel. poll. independent crops
Weighted production quantities or pollination benefits
0
1
2
3
4
5
6
1995
2000
2005

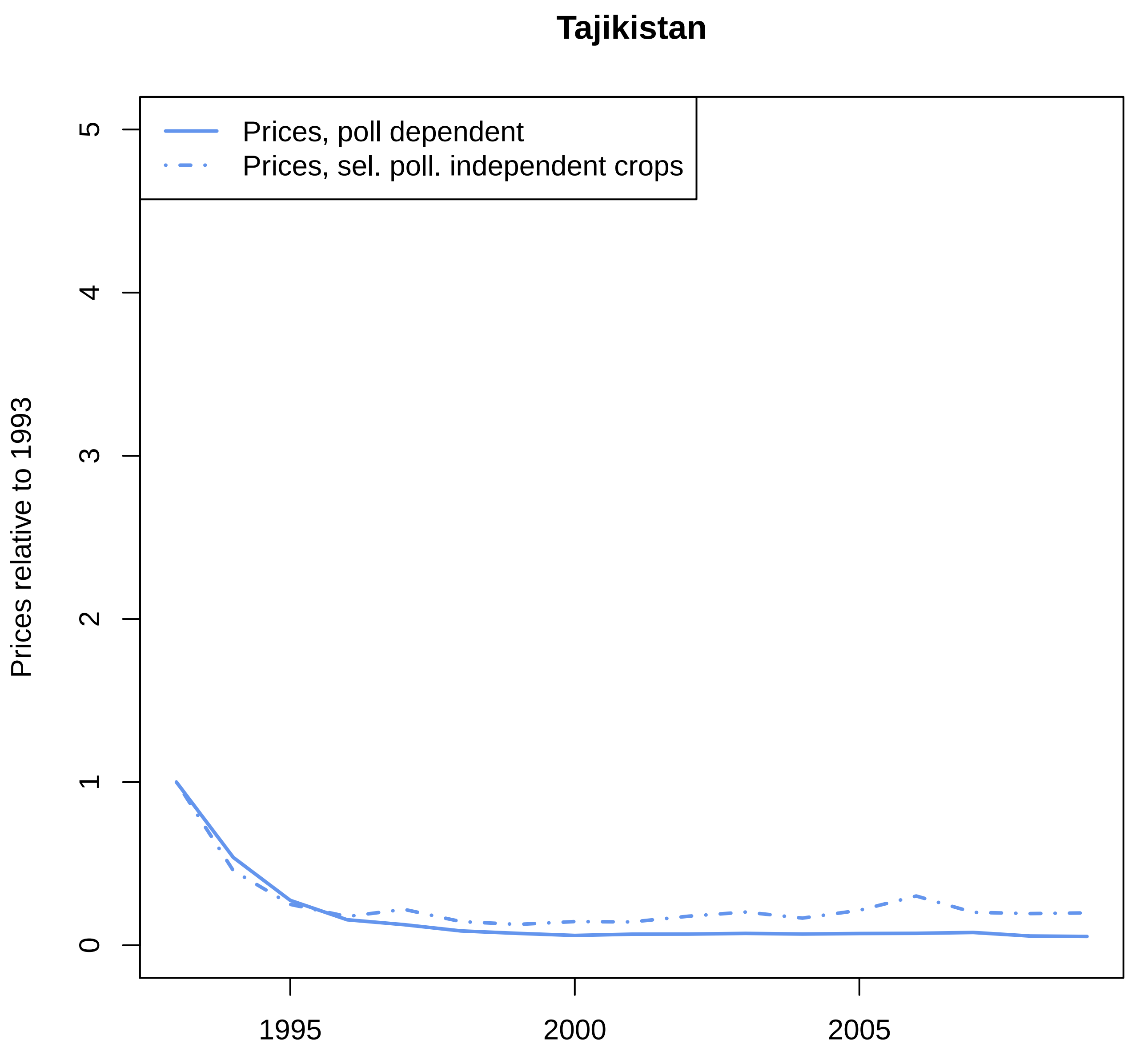

Tajikistan
Prices, poll dependent
Prices, sel. poll. independent crops
Prices relative to 1993
0
1
2
3
4
5
1995
2000
2005

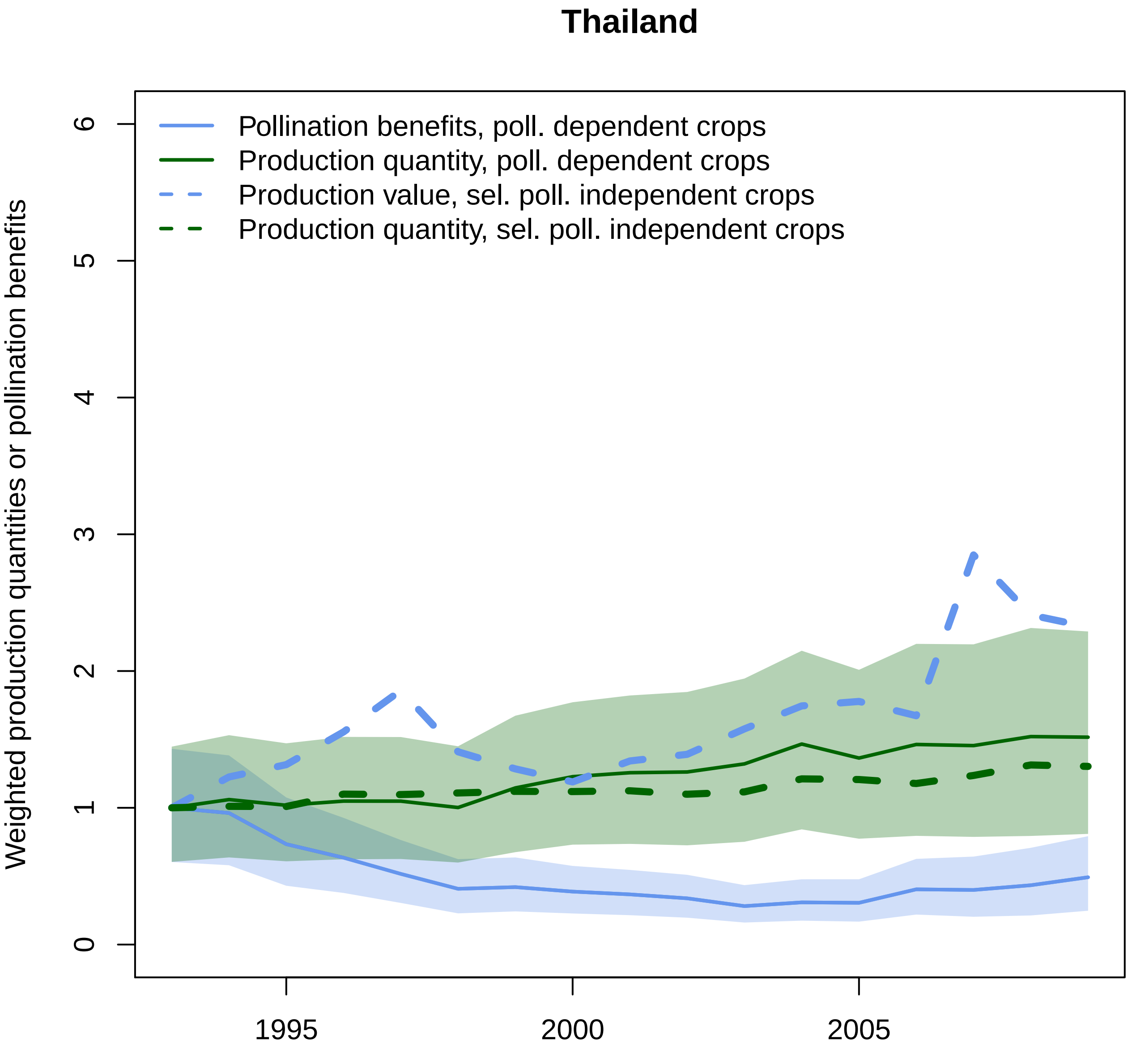

Thailand
Pollination benefits, poll. dependent crops
Production quantity, poll. dependent crops
Production value, sel. poll. independent crops
Production quantity, sel. poll. independent crops
Weighted production quantities or pollination benefits
0
1
2
3
4
5
6
1995
2000
2005

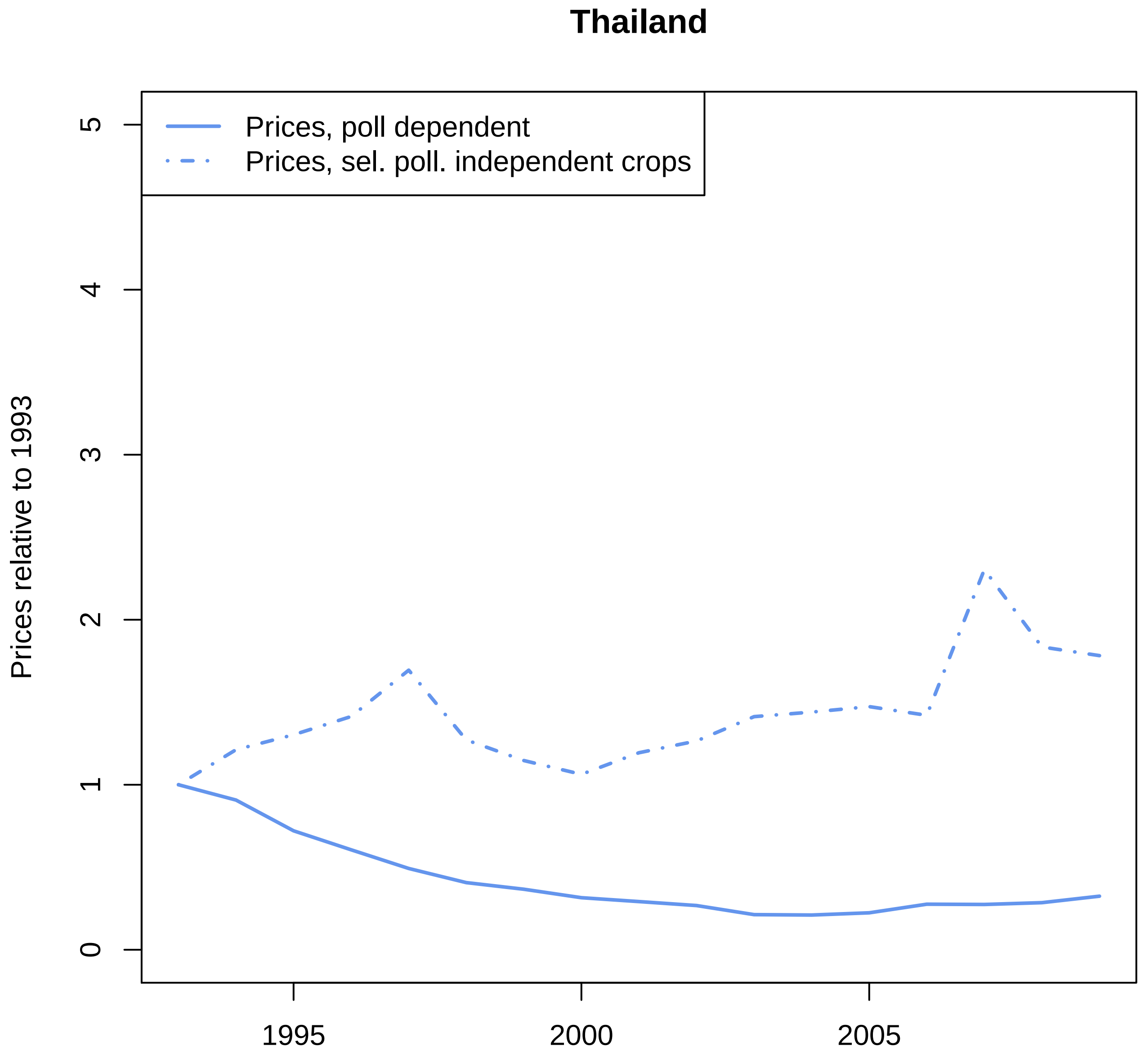

Thailand
Prices, poll dependent
Prices, sel. poll. independent crops
Prices relative to 1993
0
1
2
3
4
5
1995
2000
2005

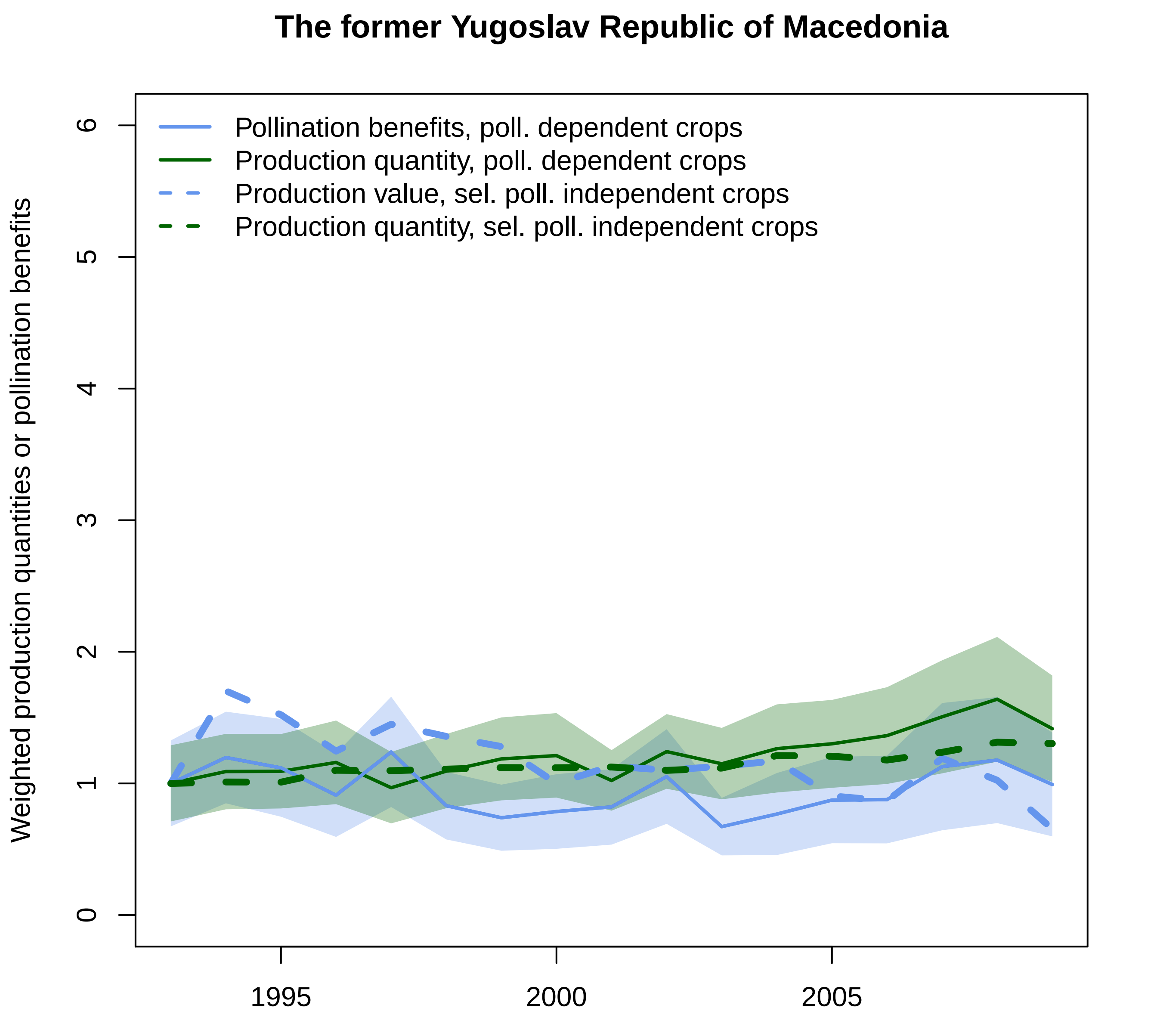

The former Yugoslav Republic of Macedonia
Pollination benefits, poll. dependent crops
Production quantity, poll. dependent crops
Production value, sel. poll. independent crops
Production quantity, sel. poll. independent crops
Weighted production quantities or pollination benefits
0
1
2
3
4
5
6
1995
2000
2005

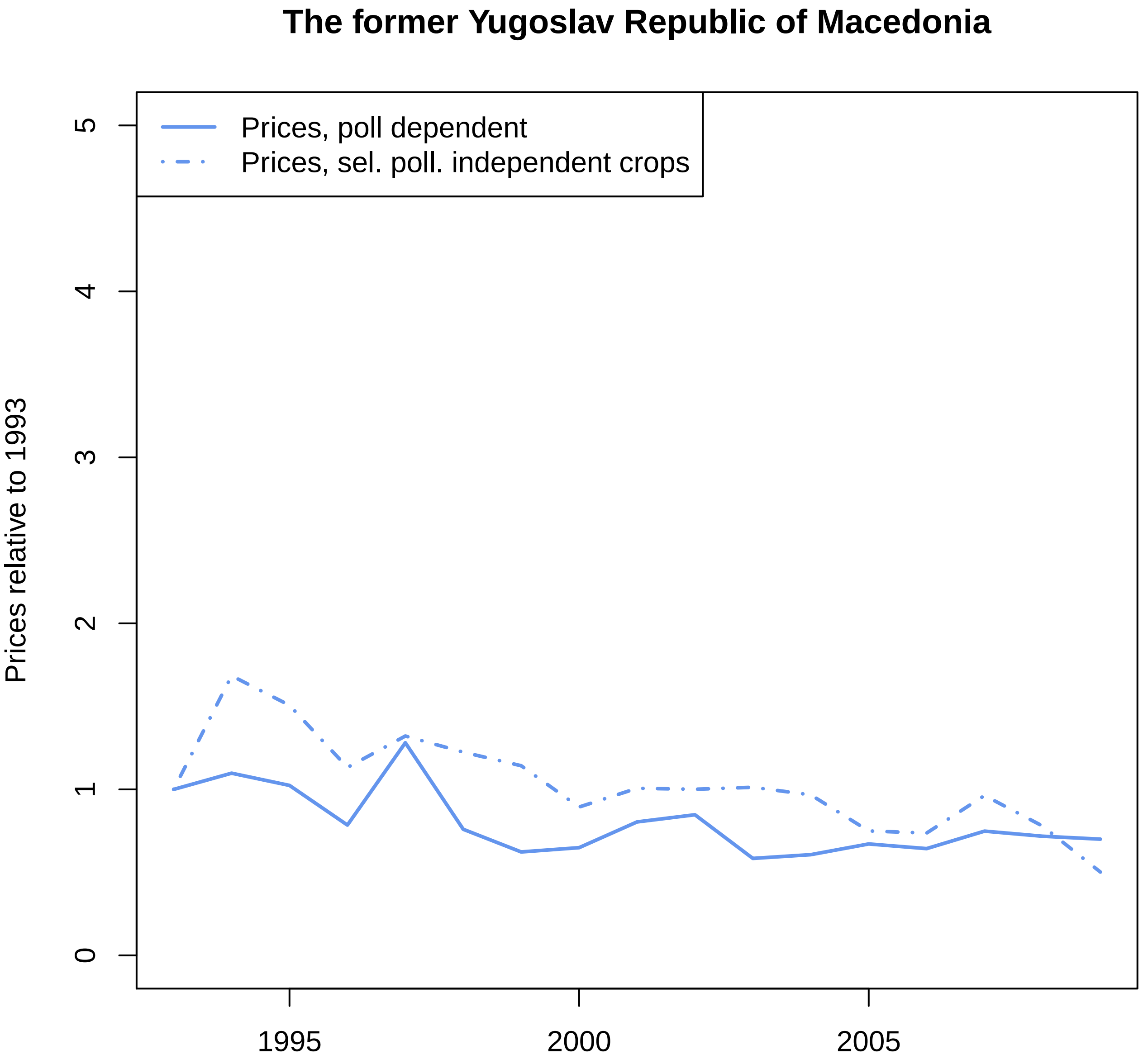
The former Yugoslav Republic of Macedonia
Prices, poll dependent
Prices, sel. poll. independent crops
Prices relative to 1993
0
1
2
3
4
5
1995
2000
2005

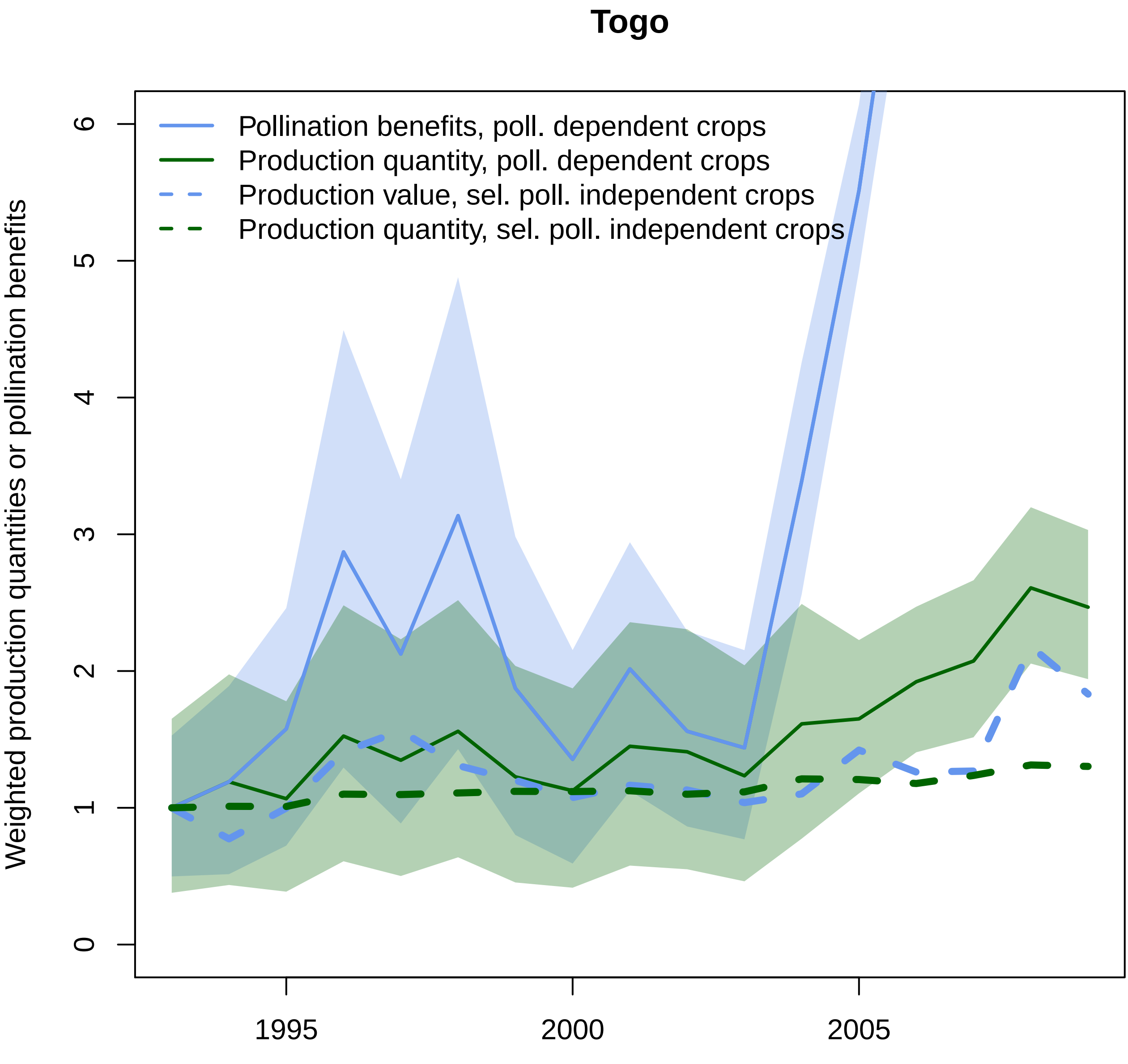
Togo
Pollination benefits, poll. dependent crops
Production quantity, poll. dependent crops
Production value, sel. poll. independent crops
Production quantity, sel. poll. independent crops
Weighted production quantities or pollination benefits
0
1
2
3
4
5
6
1995
2000
2005

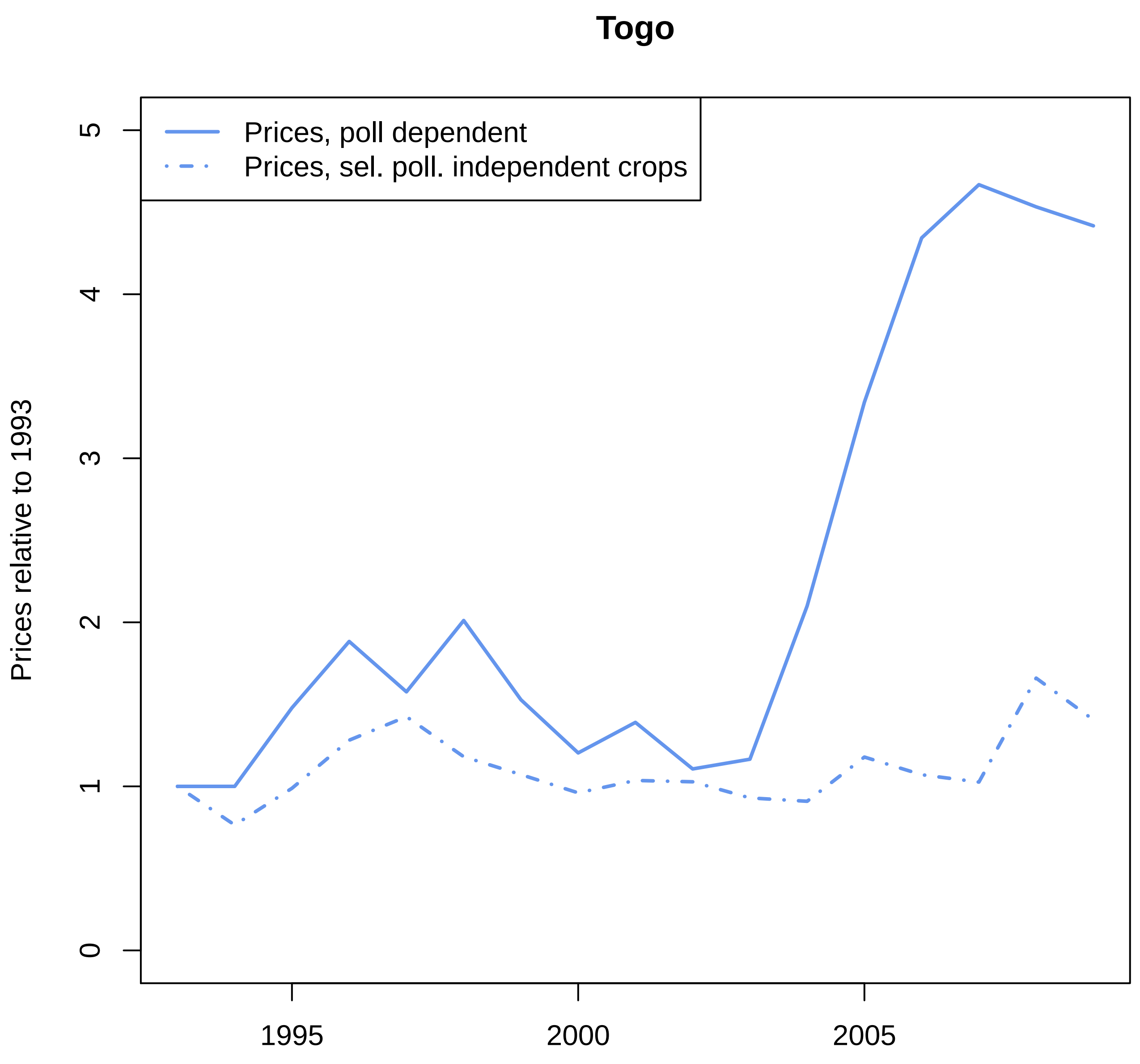
Togo
Prices, poll dependent
Prices, sel. poll. independent crops
Prices relative to 1993
0
1
2
3
4
5
1995
2000
2005

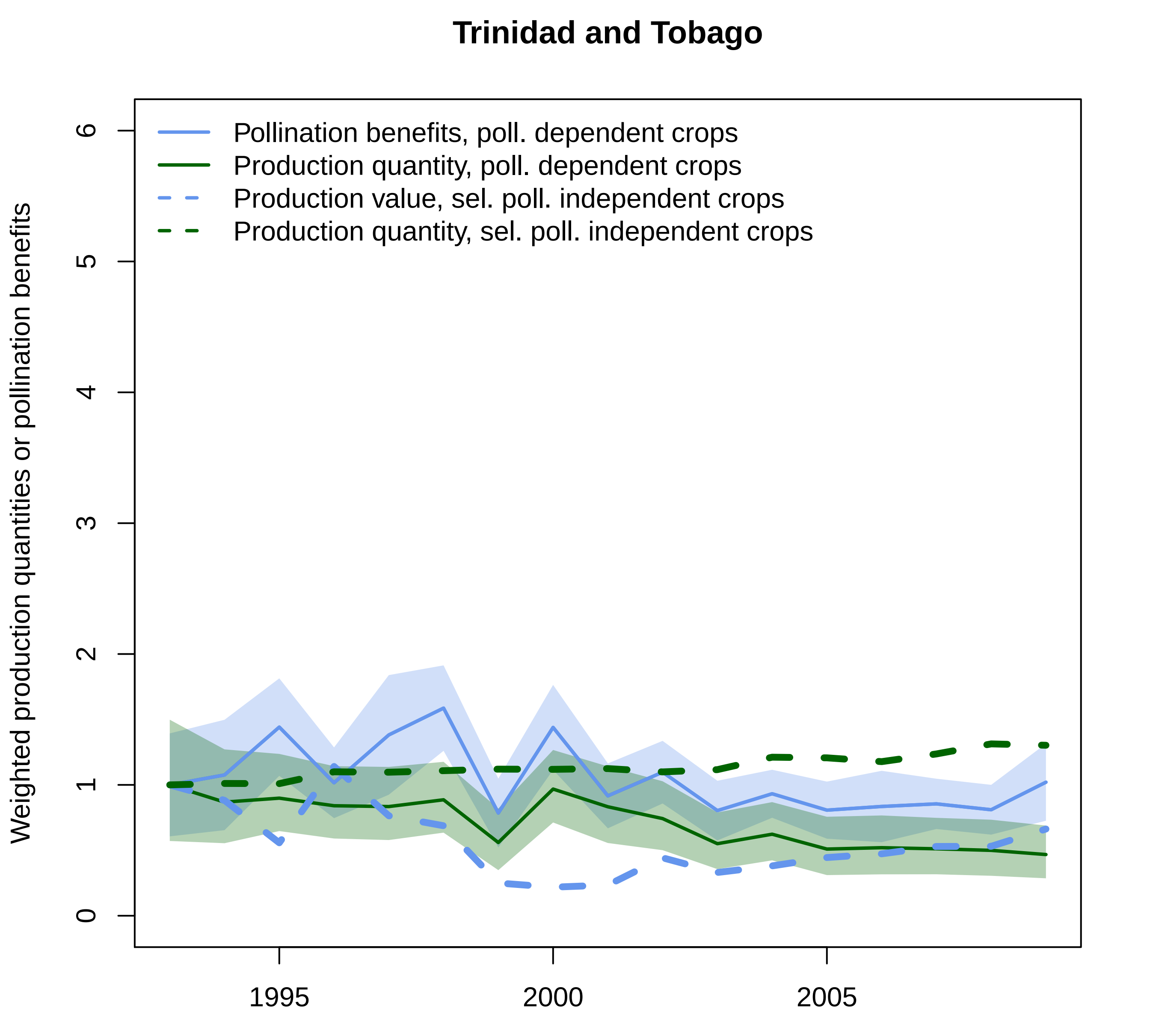

Trinidad and Tobago
Pollination benefits, poll. dependent crops
Production quantity, poll. dependent crops
Production value, sel. poll. independent crops
Production quantity, sel. poll. independent crops
Weighted production quantities or pollination benefits
0
1
2
3
4
5
6
1995
2000
2005

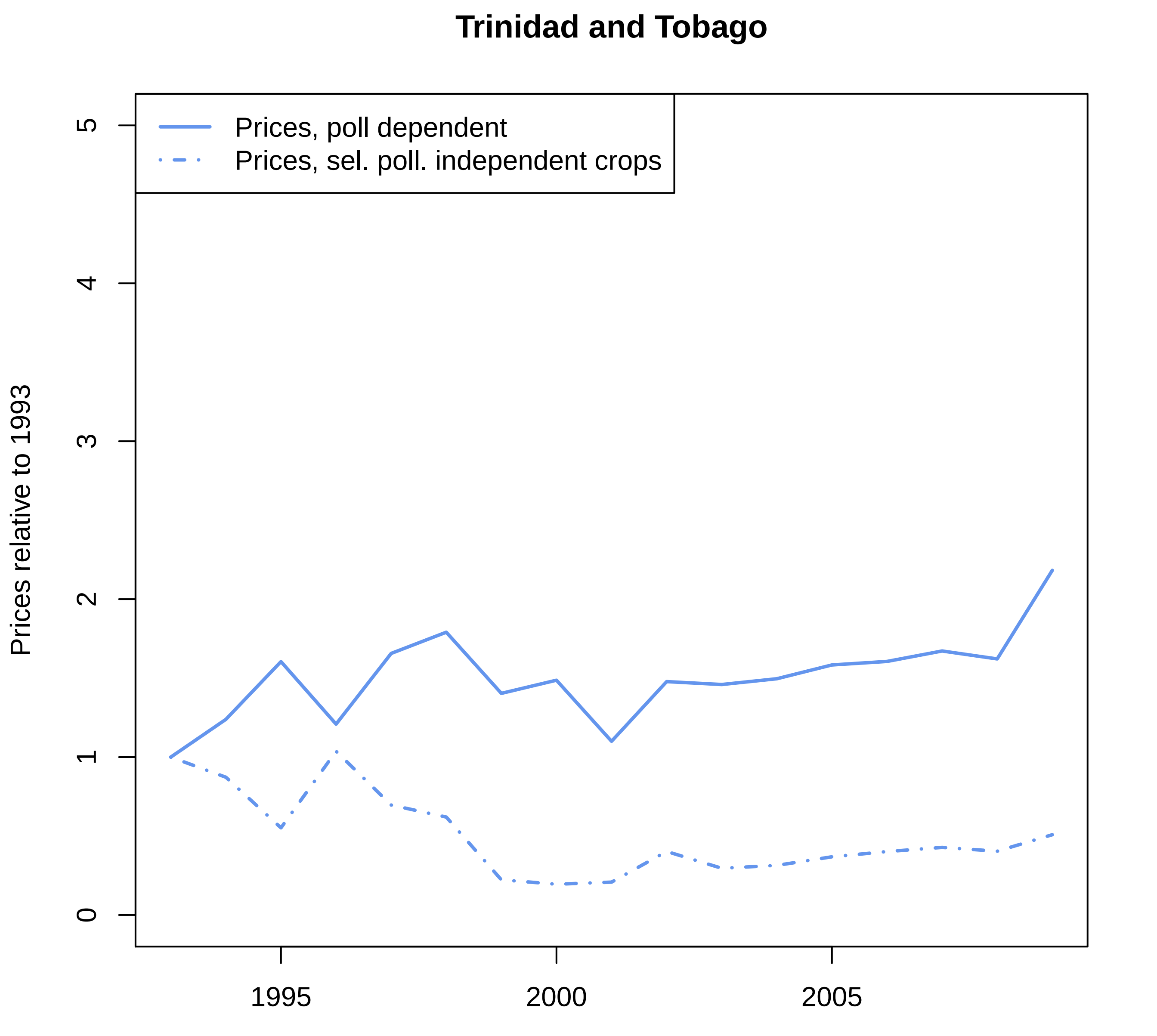
Trinidad and Tobago
Prices, poll dependent
Prices, sel. poll. independent crops
Prices relative to 1993
0
1
2
3
4
5
1995
2000
2005

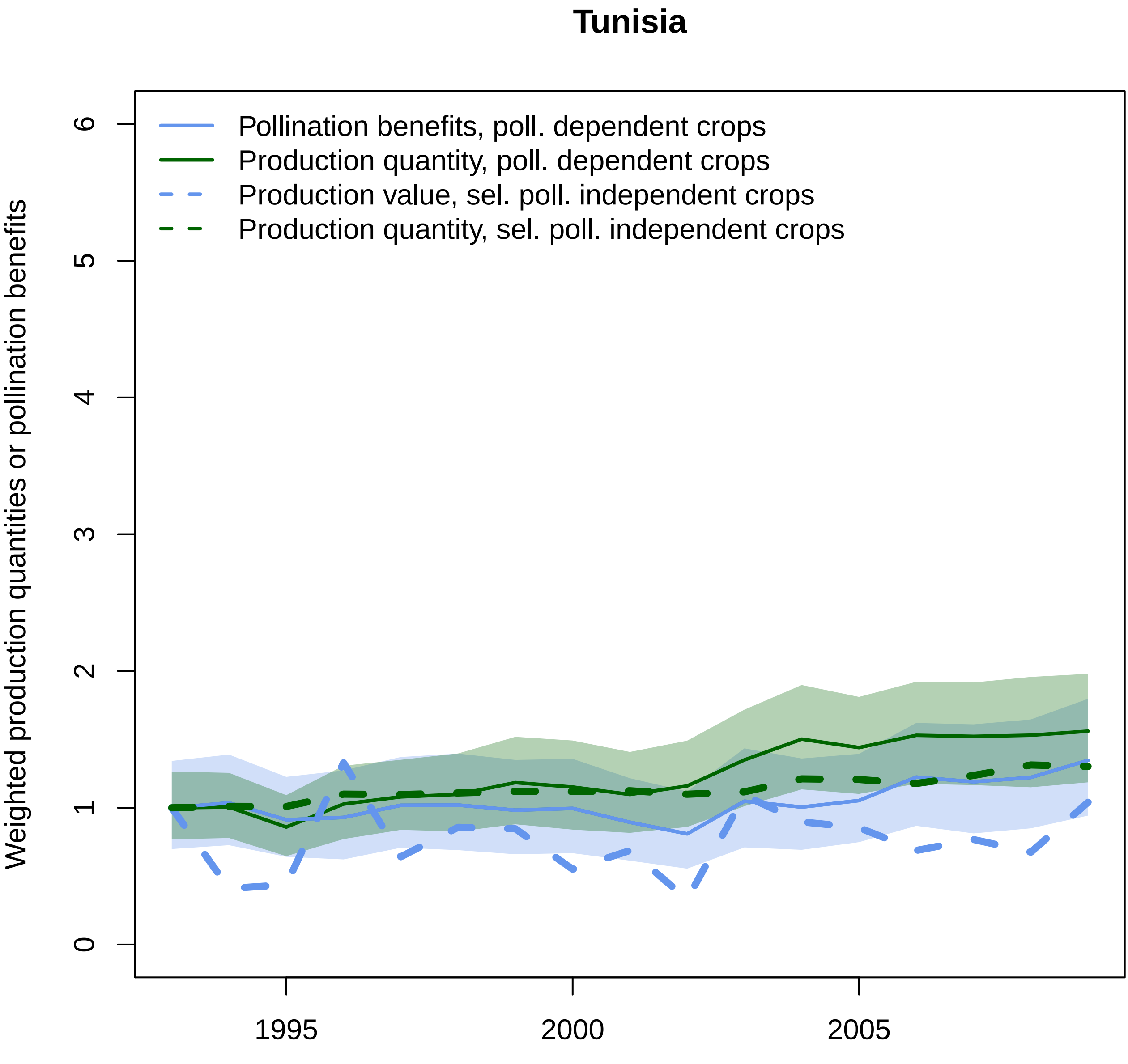

Tunisia
Pollination benefits, poll. dependent crops
Production quantity, poll. dependent crops
Production value, sel. poll. independent crops
Production quantity, sel. poll. independent crops
Weighted production quantities or pollination benefits
0
1
2
3
4
5
6
1995
2000
2005

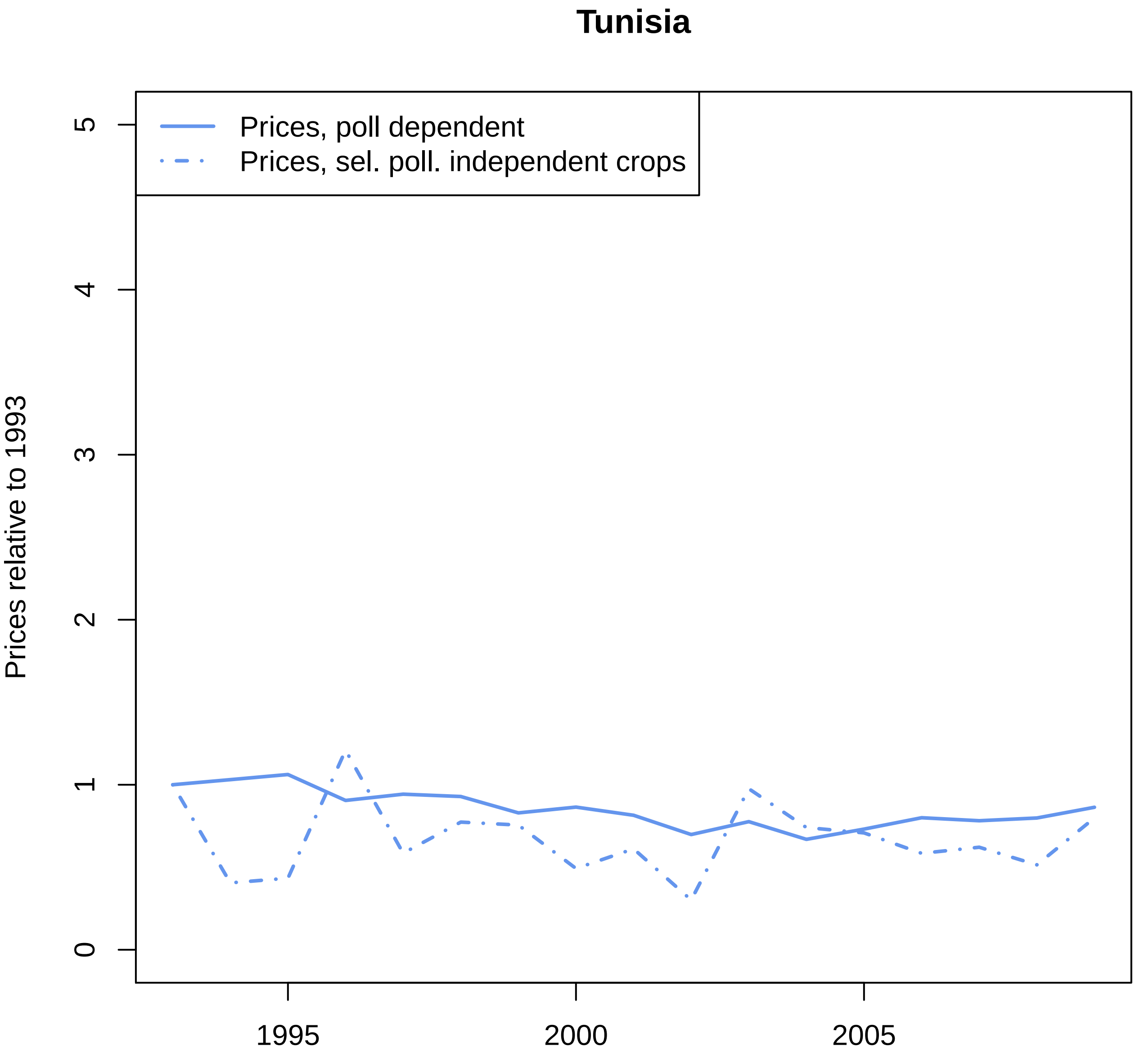

Tunisia
Prices, poll dependent
Prices, sel. poll. independent crops
Prices relative to 1993
0
1
2
3
4
5
1995
2000
2005

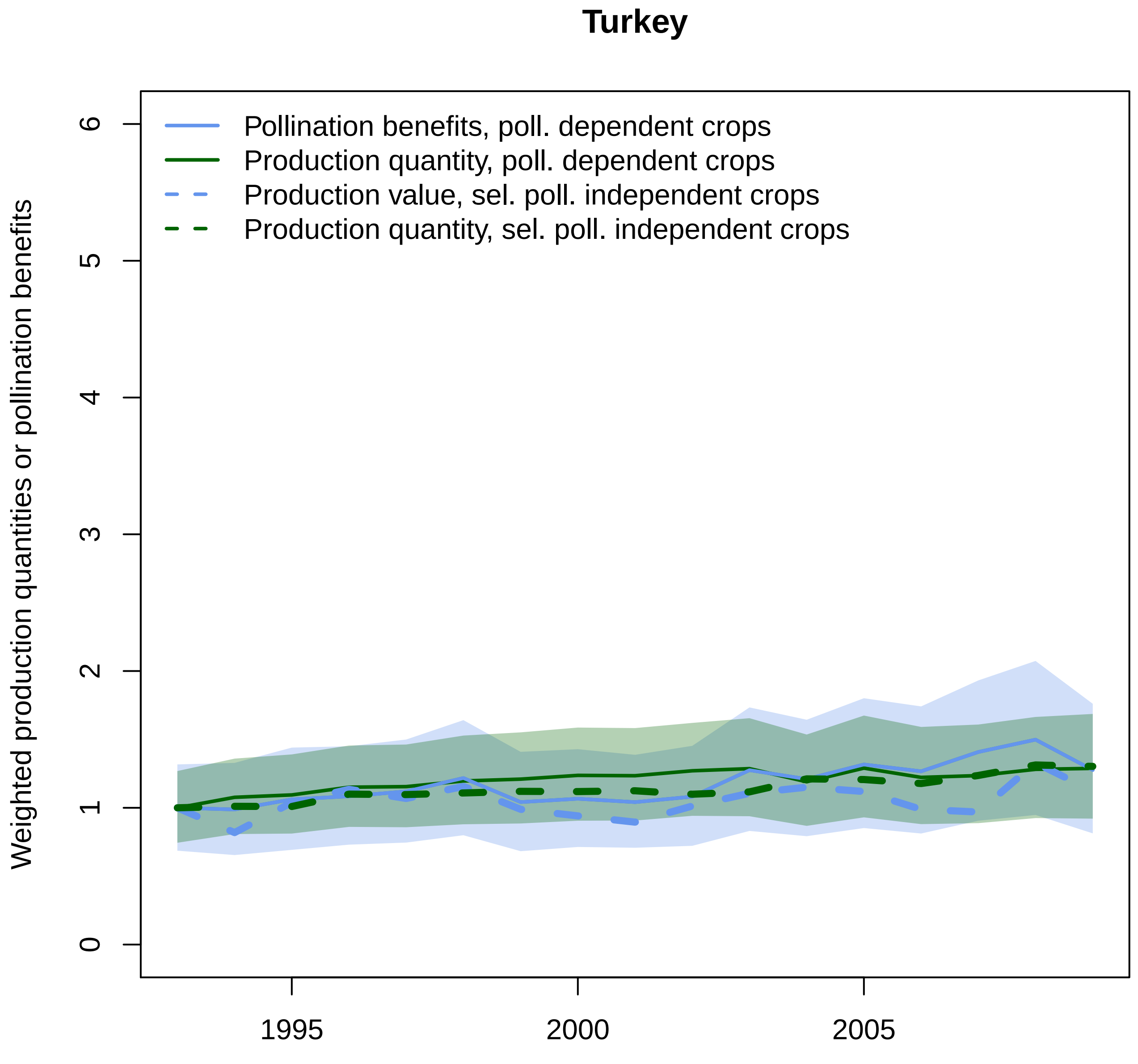
Turkey
Pollination benefits, poll. dependent crops
Production quantity, poll. dependent crops
Production value, sel. poll. independent crops
Production quantity, sel. poll. independent crops
Weighted production quantities or pollination benefits
0
1
2
3
4
5
6
1995
2000
2005

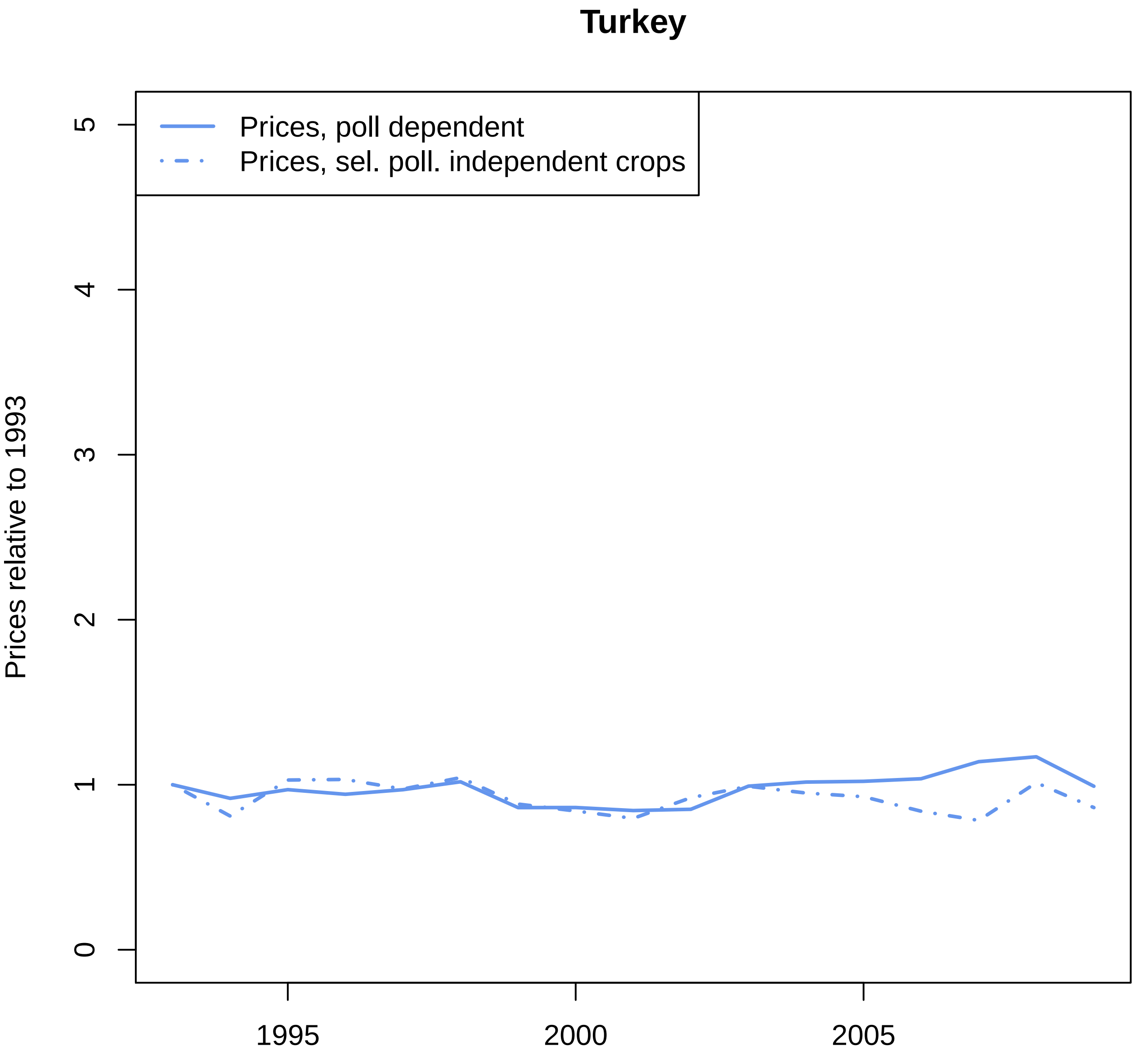

Turkey
Prices, poll dependent
Prices, sel. poll. independent crops
Prices relative to 1993
0
1
2
3
4
5
1995
2000
2005

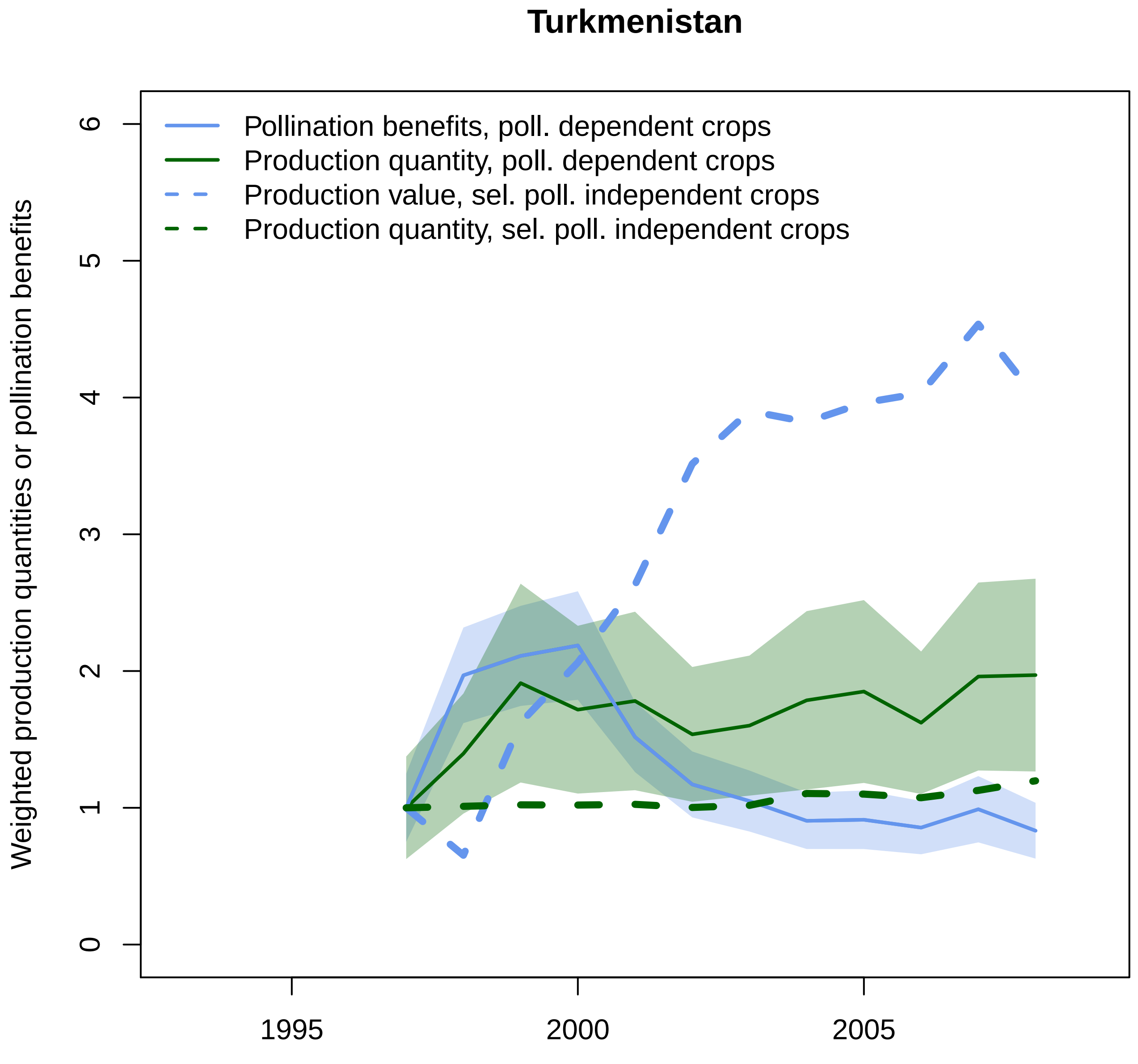
Turkmenistan
Pollination benefits, poll. dependent crops
Production quantity, poll. dependent crops
Production value, sel. poll. independent crops
Production quantity, sel. poll. independent crops
Weighted production quantities or pollination benefits
0
1
2
3
4
5
6
1995
2000
2005

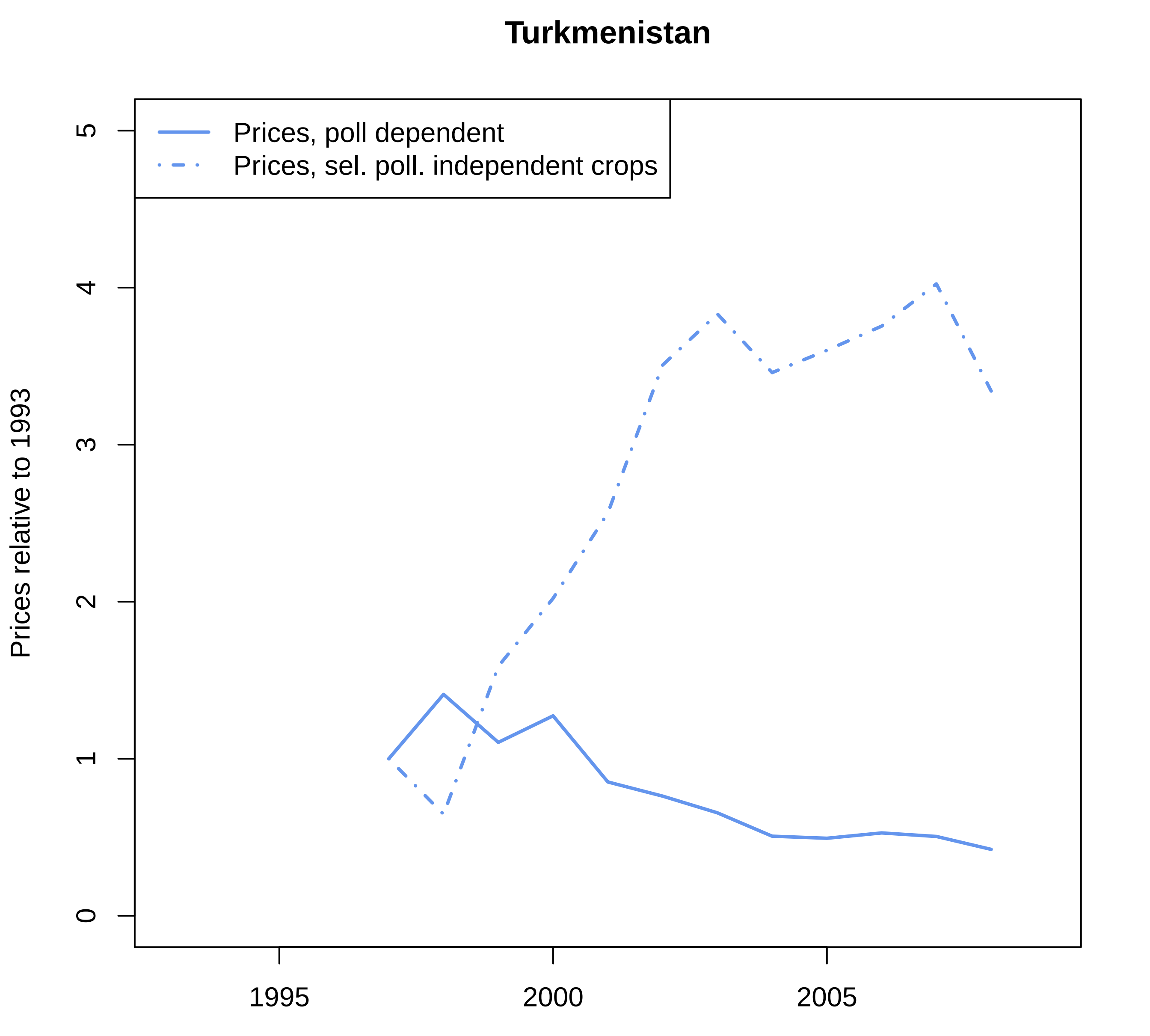

Turkmenistan
Prices, poll dependent
Prices, sel. poll. independent crops
Prices relative to 1993
0
1
2
3
4
5
1995
2000
2005

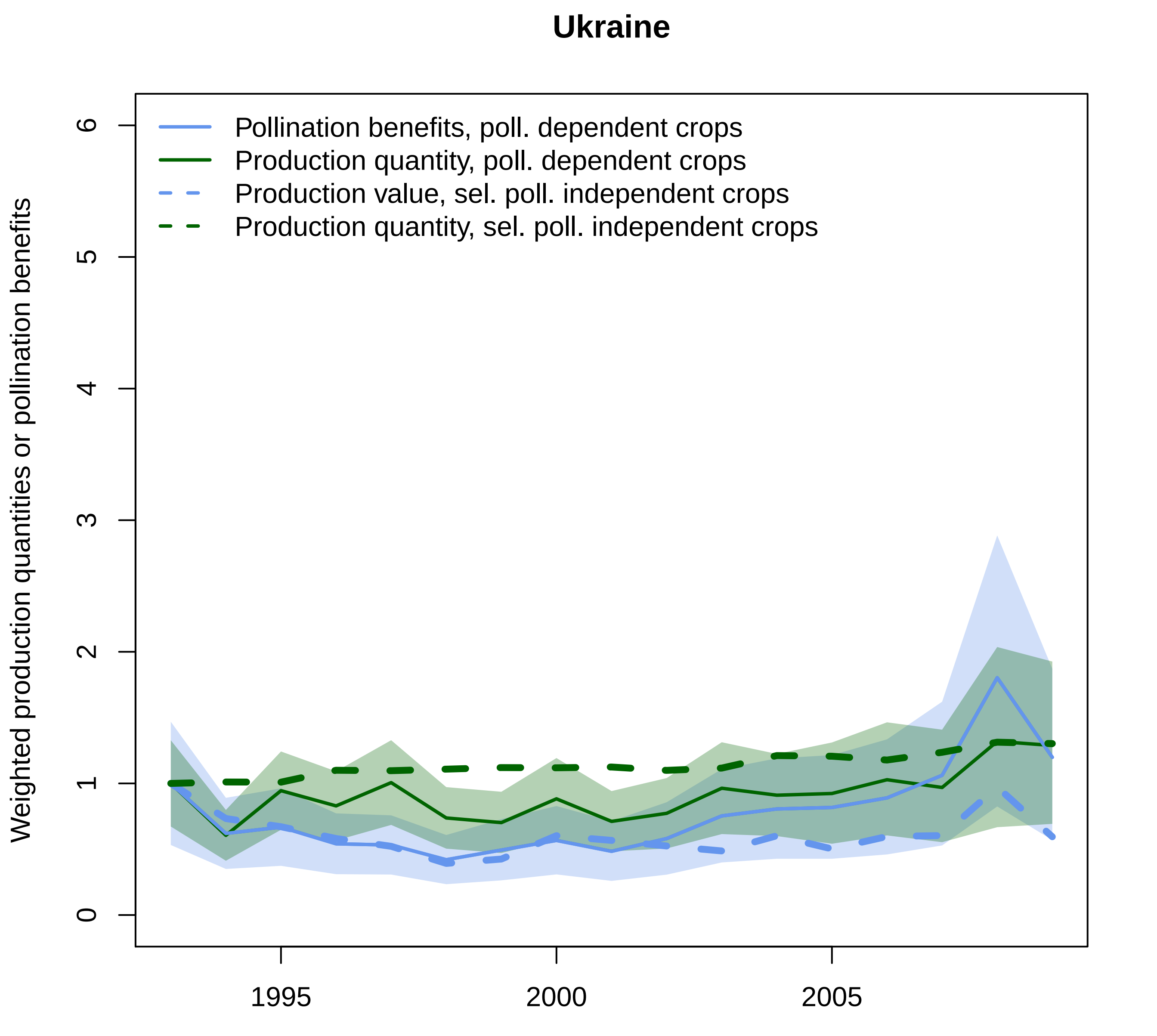

Ukraine
Pollination benefits, poll. dependent crops
Production quantity, poll. dependent crops
Production value, sel. poll. independent crops
Production quantity, sel. poll. independent crops
Weighted production quantities or pollination benefits
0
1
2
3
4
5
6
1995
2000
2005

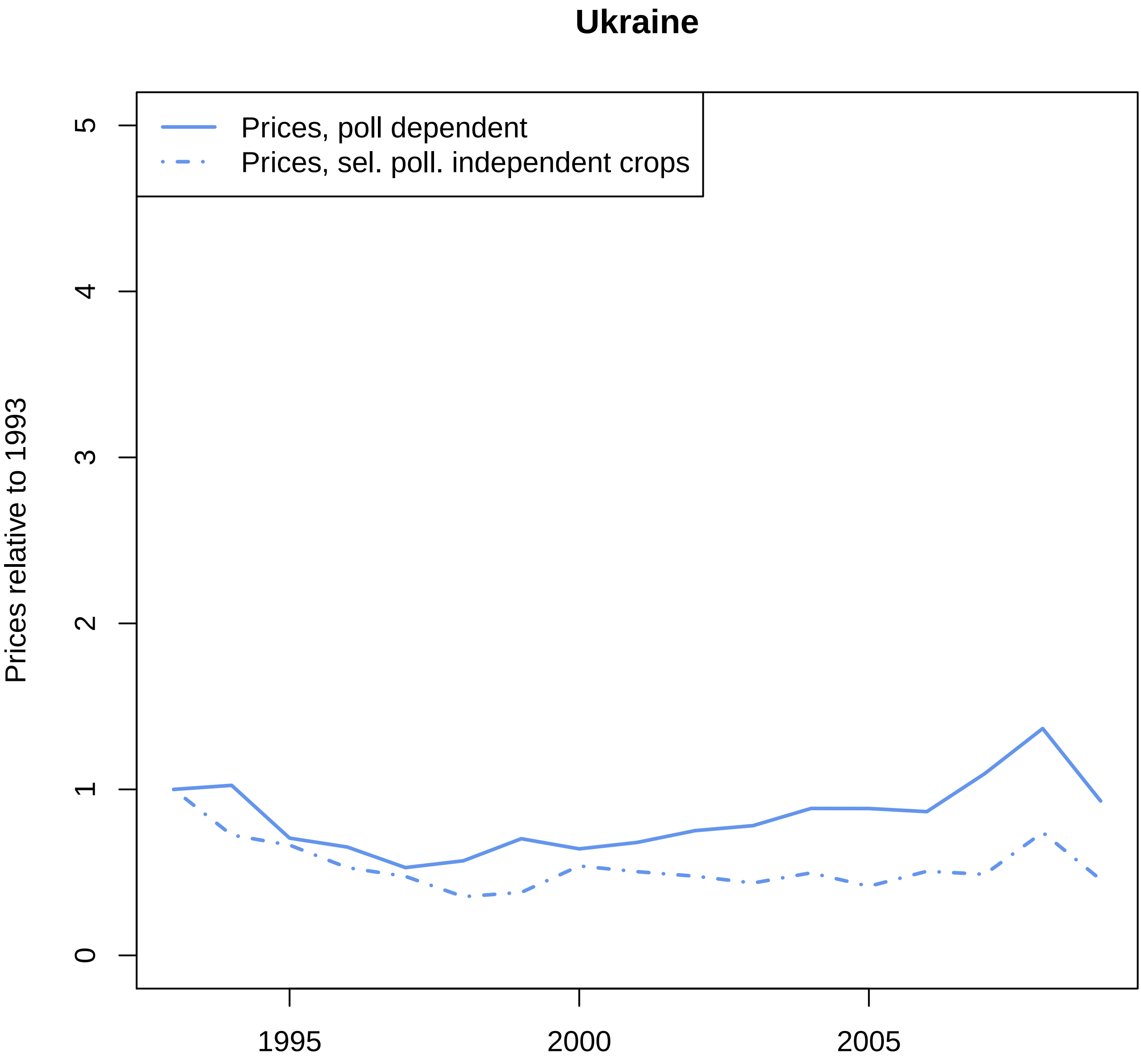

Ukraine
Prices, poll dependent
Prices, sel. poll. independent crops
Prices relative to 1993
0
1
2
3
4
5
1995
2000
2005

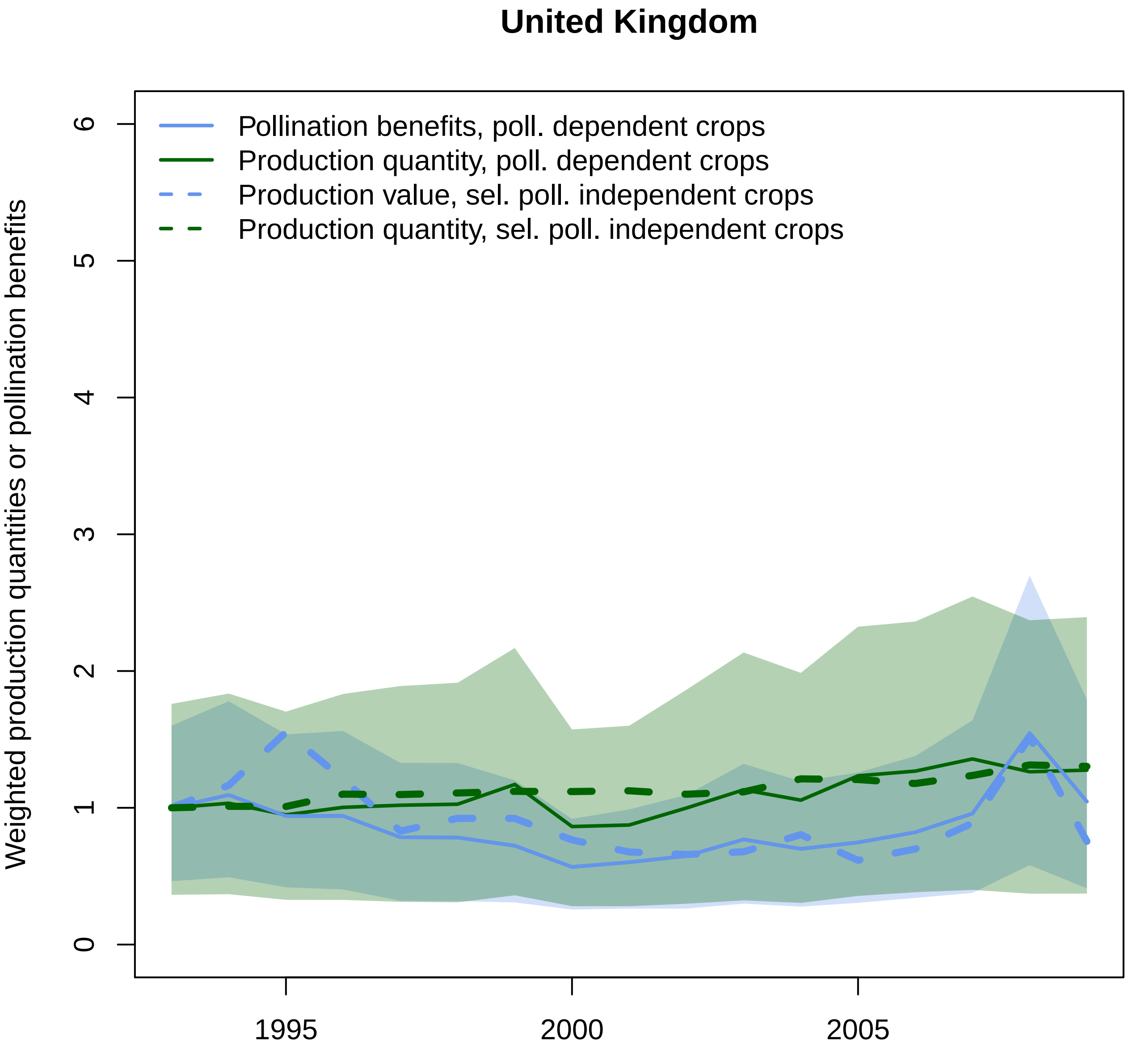

United Kingdom
Pollination benefits, poll. dependent crops
Production quantity, poll. dependent crops
Production value, sel. poll. independent crops
Production quantity, sel. poll. independent crops
Weighted production quantities or pollination benefits
0
1
2
3
4
5
6
1995
2000
2005

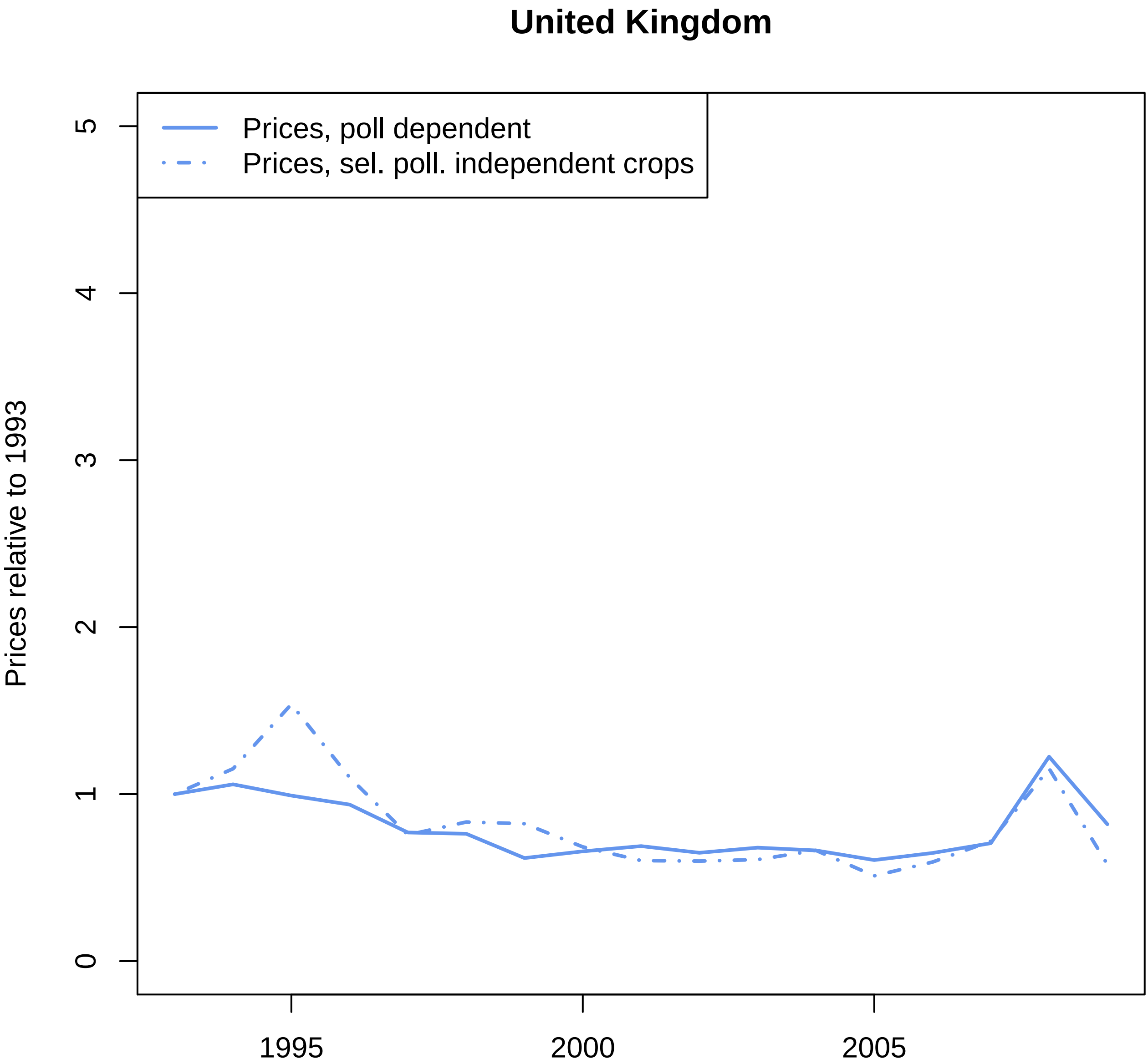
United Kingdom
Prices, poll dependent
Prices, sel. poll. independent crops
Prices relative to 1993
0
1
2
3
4
5
1995
2000
2005

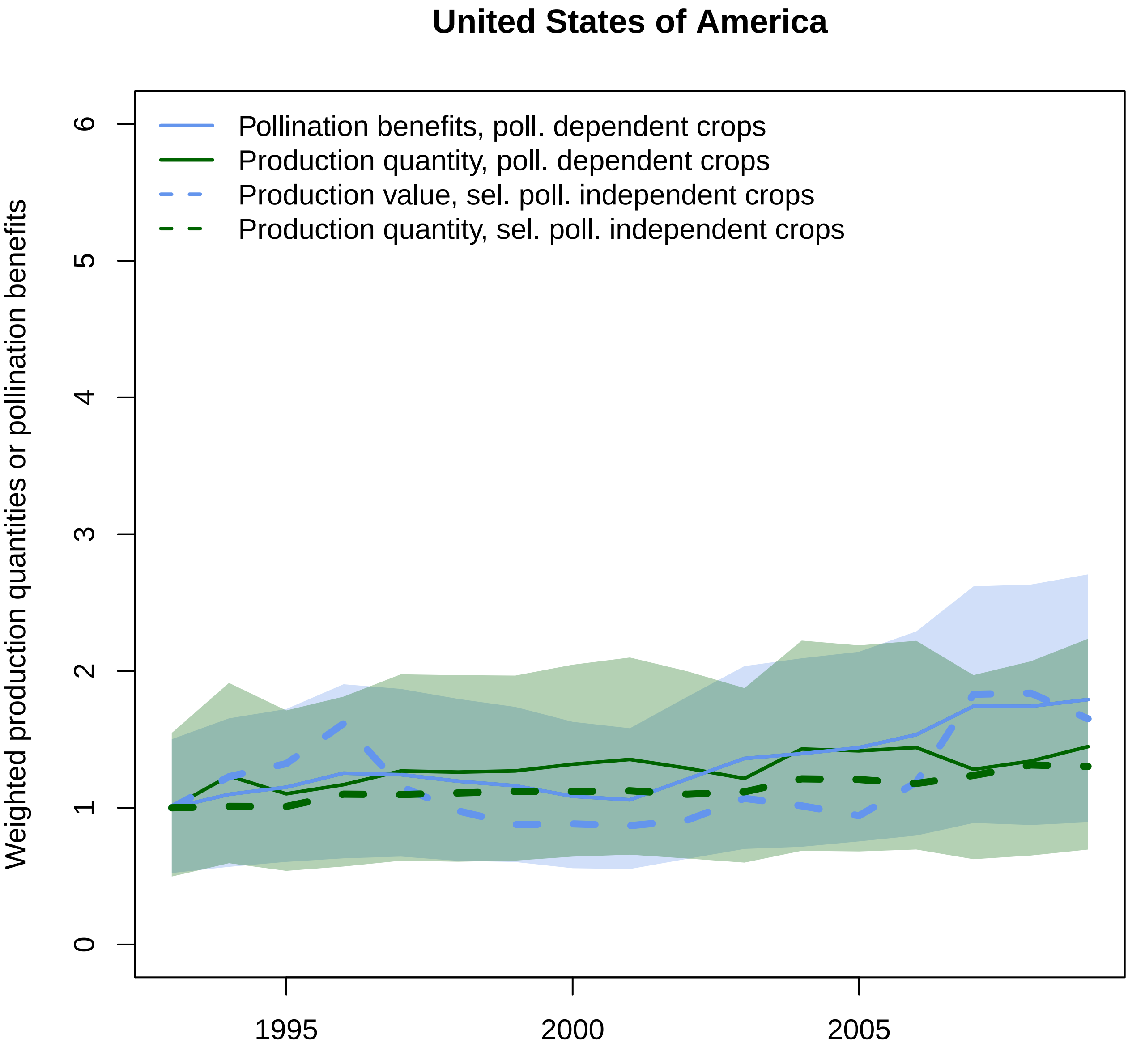
United States of America
Weighted production quantities or pollination benefits
0
1
2
3
4
5
6
1995
2000
2005
Pollination benefits, poll. dependent crops
Production quantity, poll. dependent crops
Production value, sel. poll. independent crops
Production quantity, sel. poll. independent crops

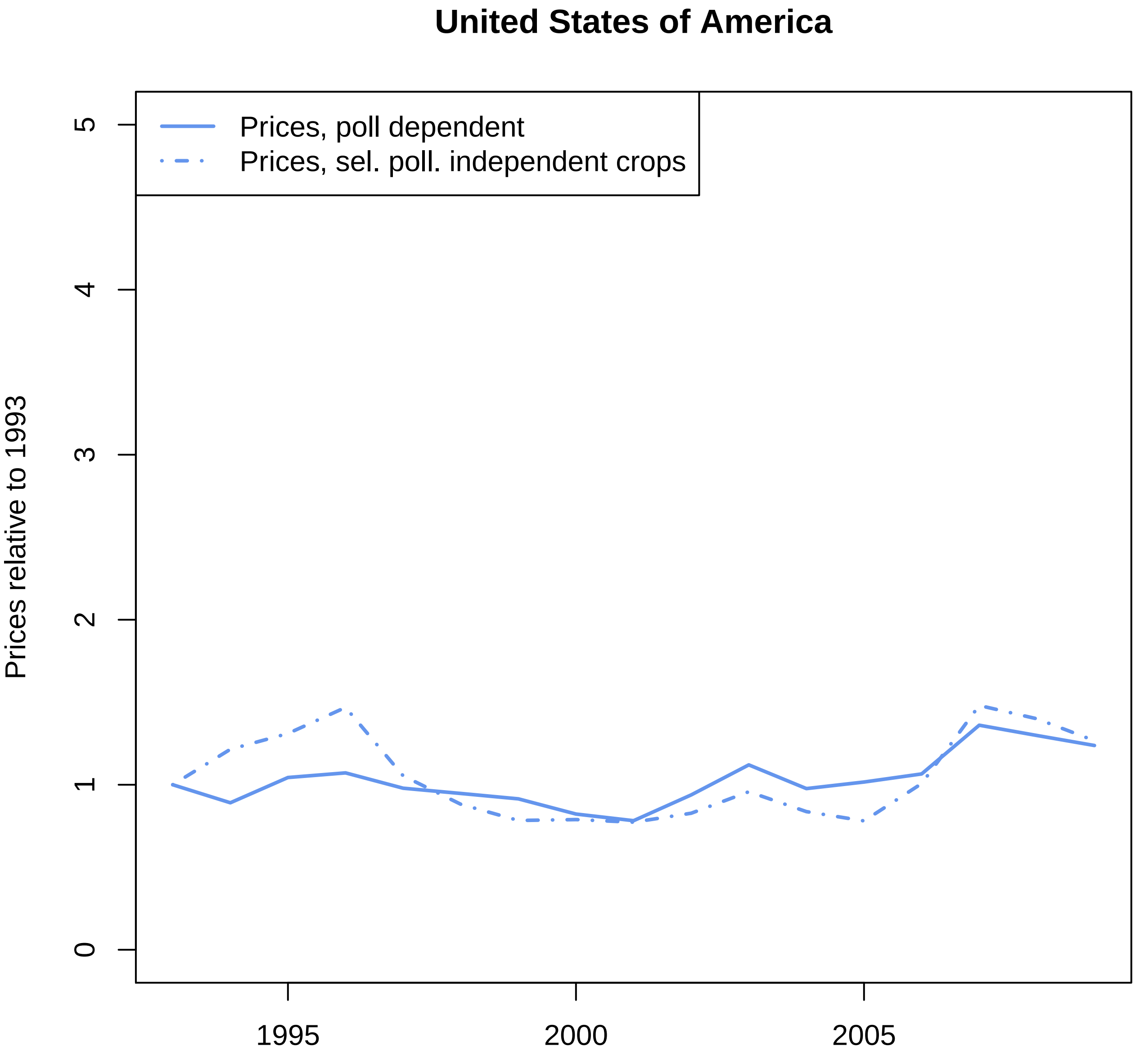

United States of America
Prices, poll dependent
Prices, sel. poll. independent crops
Prices relative to 1993
0
1
2
3
4
5
1995
2000
2005

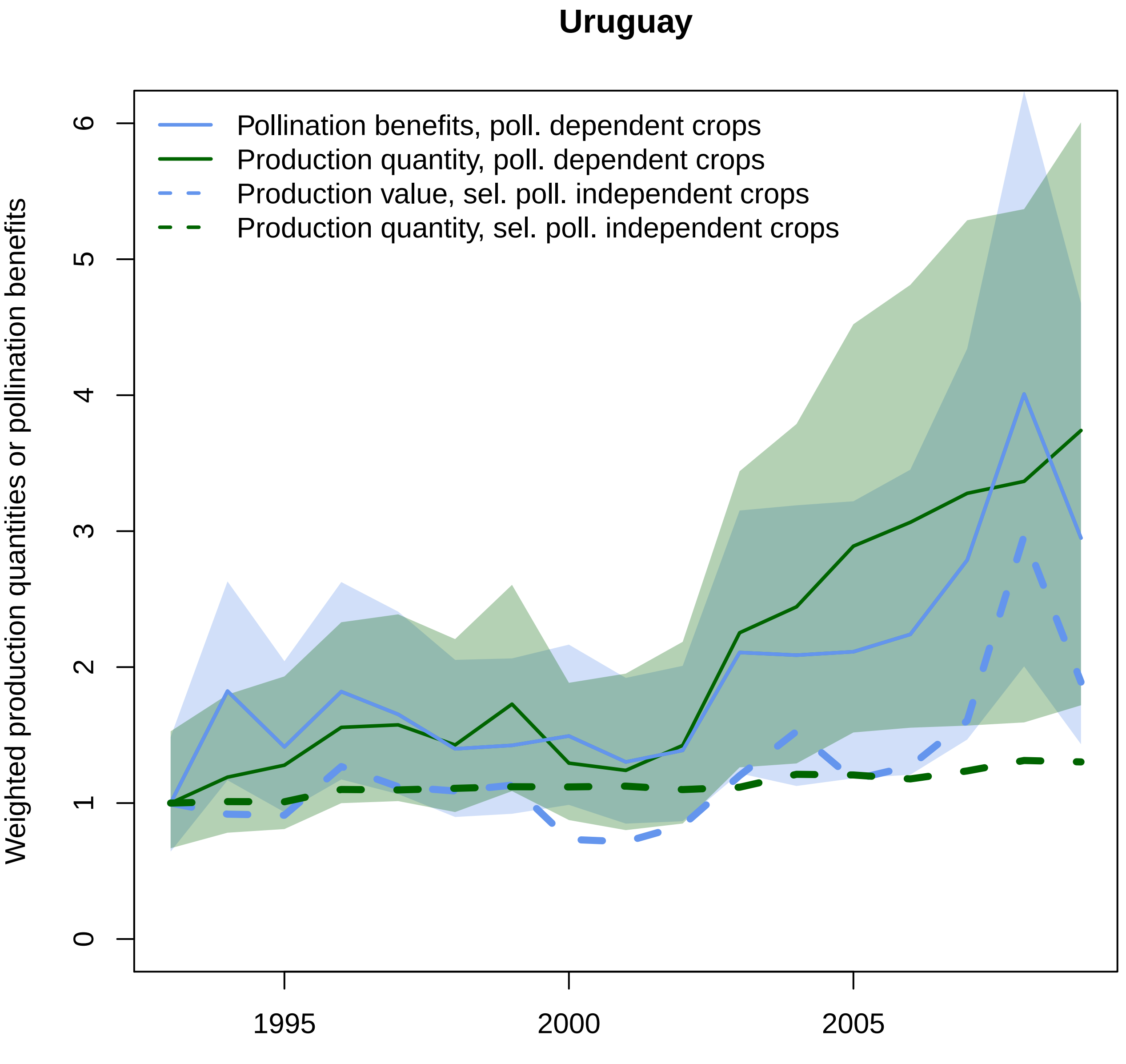

Uruguay
Pollination benefits, poll. dependent crops
Production quantity, poll. dependent crops
Production value, sel. poll. independent crops
Production quantity, sel. poll. independent crops
Weighted production quantities or pollination benefits
0
1
2
3
4
5
6
1995
2000
2005

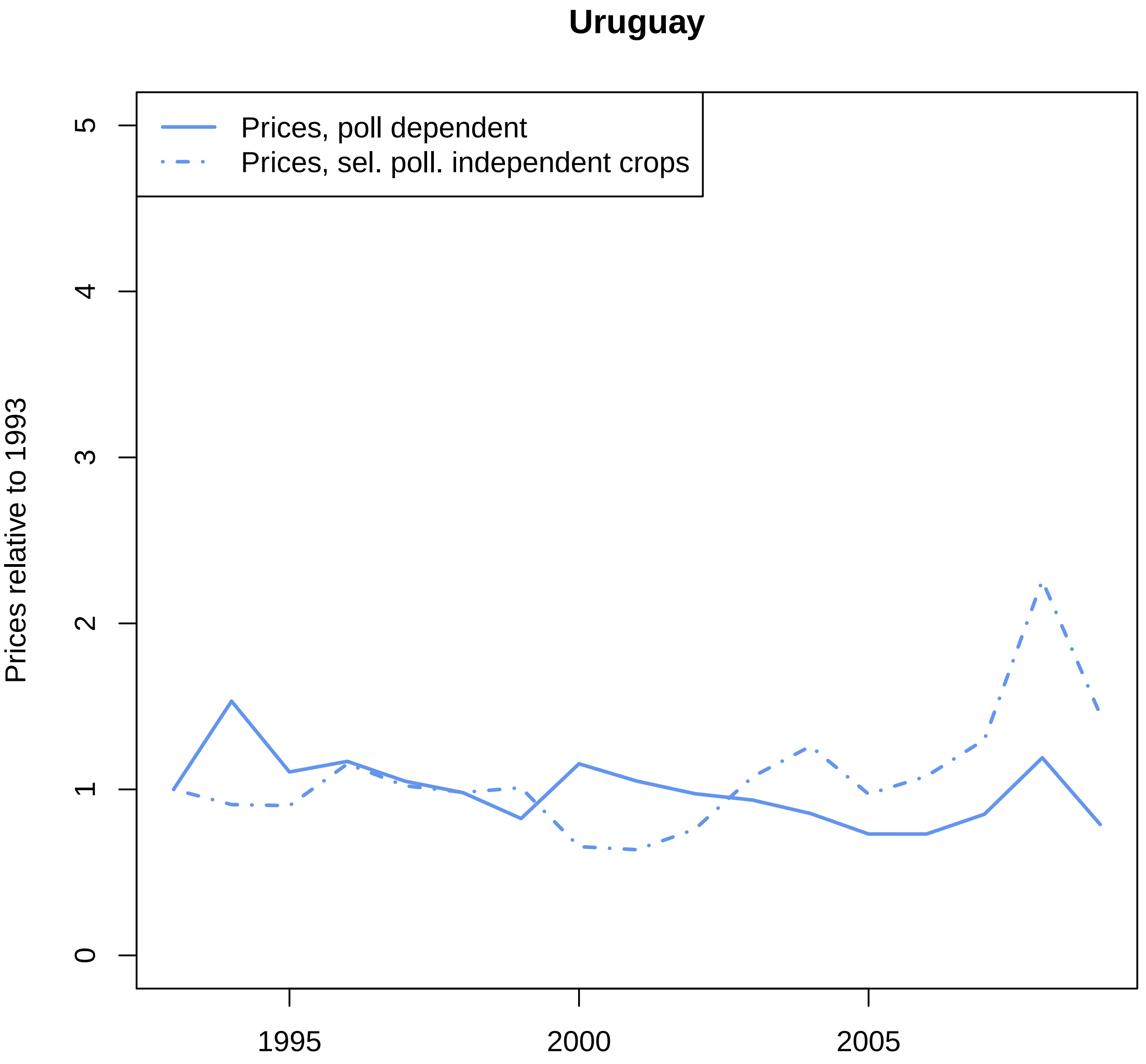

Uruguay
Prices, poll dependent
Prices, sel. poll. independent crops
Prices relative to 1993
0
1
2
3
4
5
1995
2000
2005

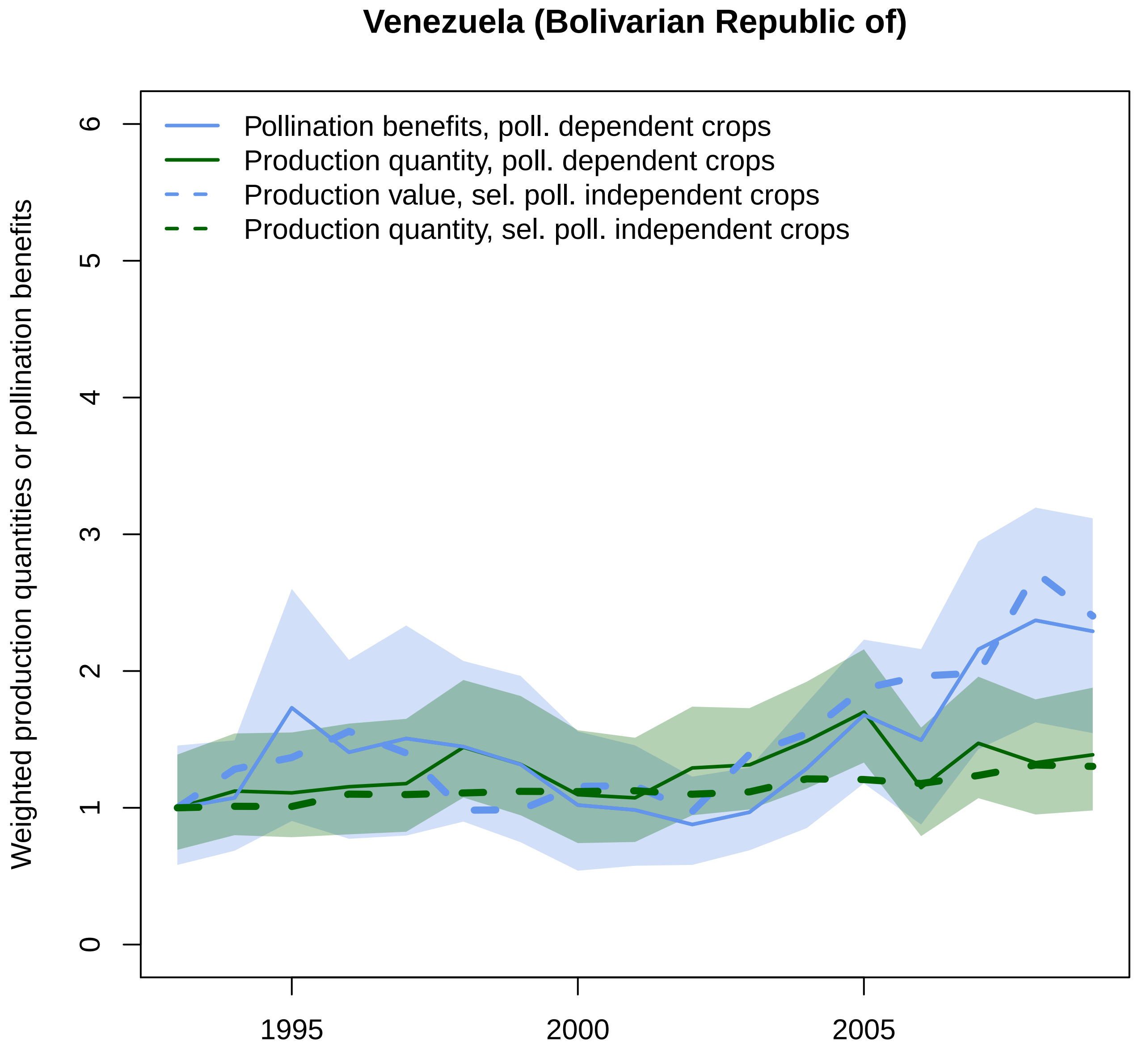
Venezuela (Bolivarian Republic of)
Pollination benefits, poll. dependent crops
Production quantity, poll. dependent crops
Production value, sel. poll. independent crops
Production quantity, sel. poll. independent crops
Weighted production quantities or pollination benefits
0
1
2
3
4
5
6
1995
2000
2005

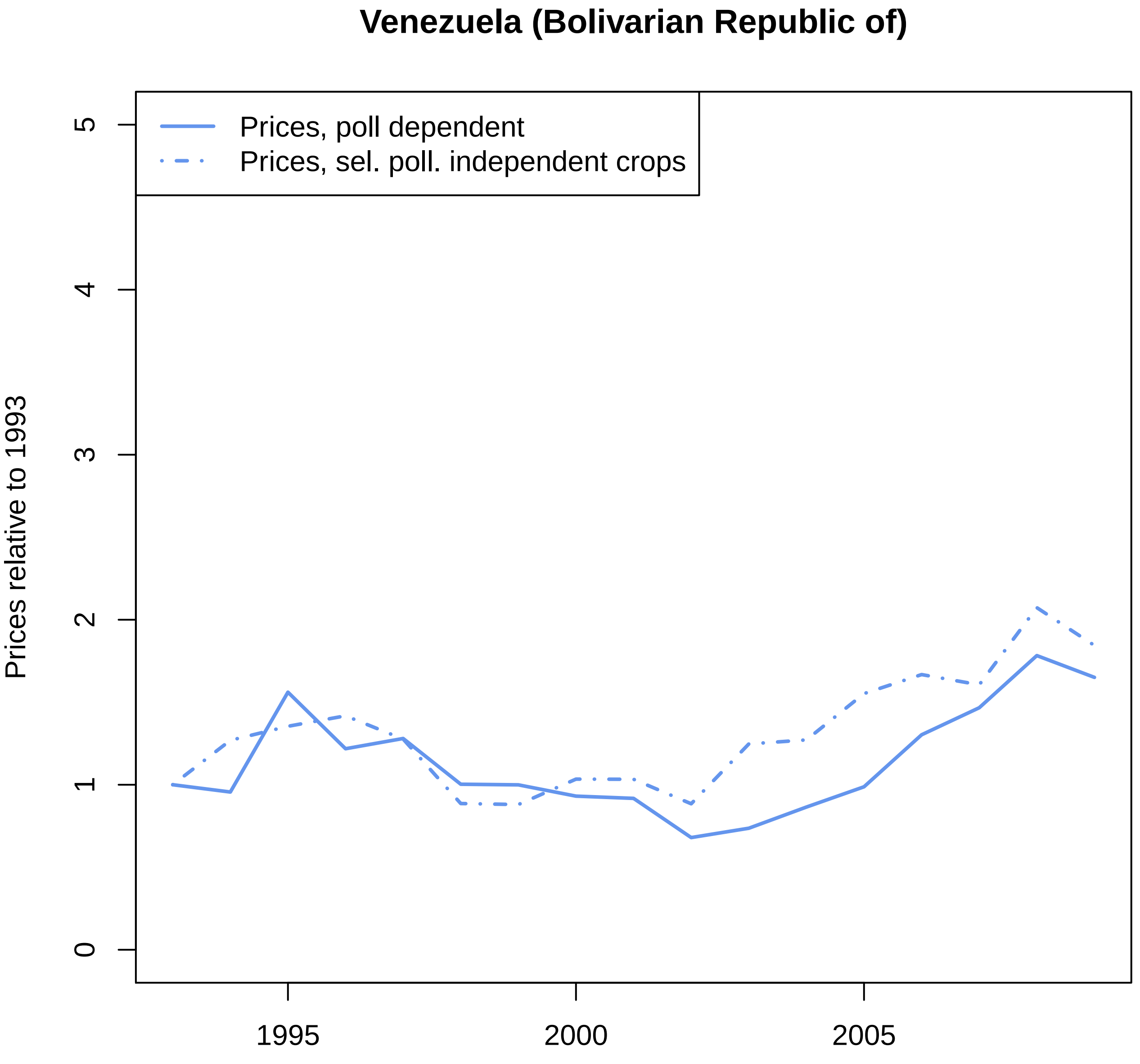
Venezuela (Bolivarian Republic of)
Prices, poll dependent
Prices, sel. poll. independent crops
Prices relative to 1993
0
1
2
3
4
5
1995
2000
2005

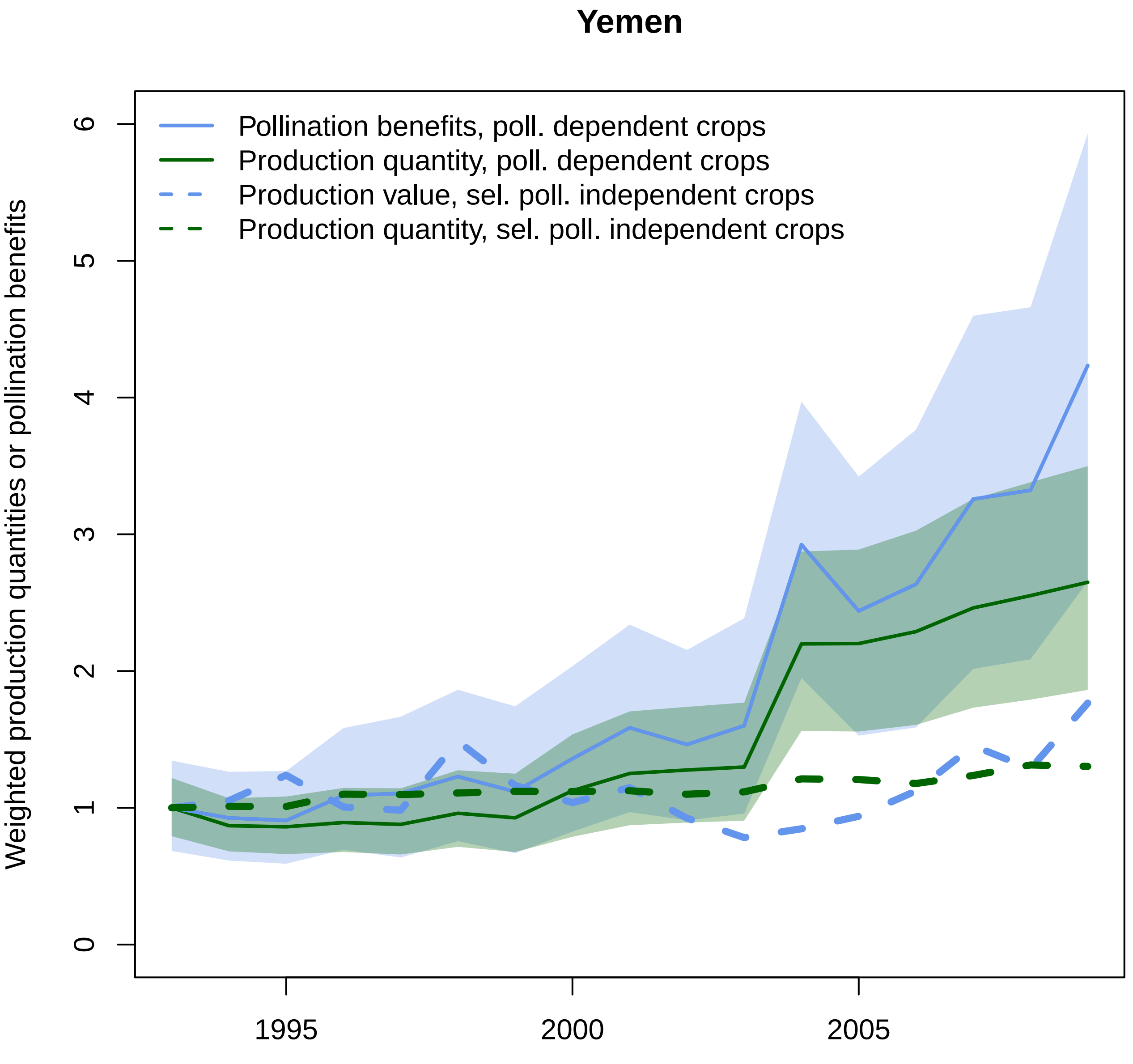

Yemen
Pollination benefits, poll. dependent crops
Production quantity, poll. dependent crops
Production value, sel. poll. independent crops
Production quantity, sel. poll. independent crops
Weighted production quantities or pollination benefits
0
1
2
3
4
5
6
1995
2000
2005

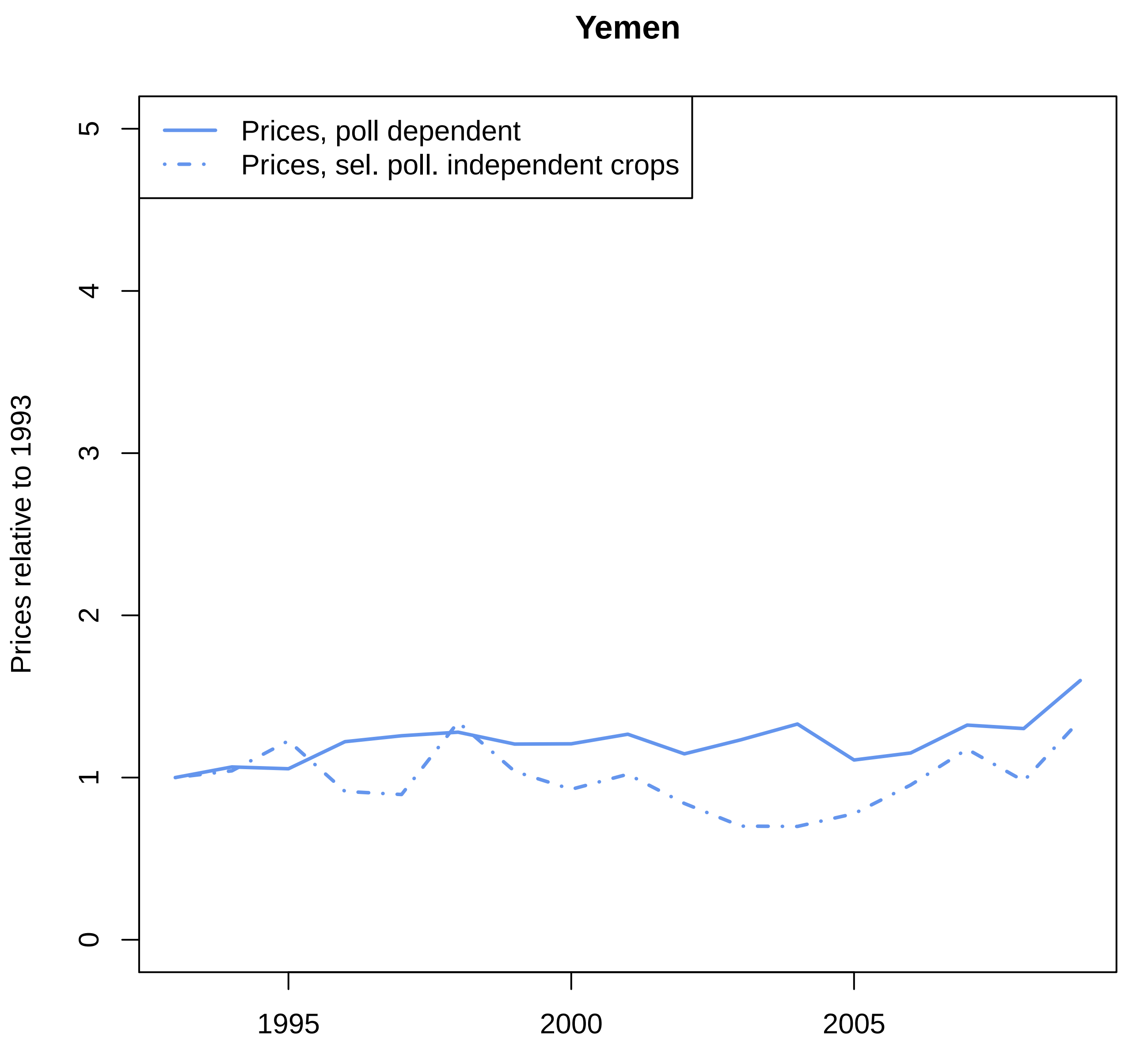

Yemen
Prices, poll dependent
Prices, sel. poll. independent crops
Prices relative to 1993
0
1
2
3
4
5
1995
2000
2005
