# Supplementary material for: Spatial and Temporal Trends of Global Pollination Benefit
Source: PLoS One. 2012 Apr 26;7(4):e35954. doi: 10.1371/journal.pone.0035954 (PMC3338563; doi:10.1371/journal.pone.0035954)
Supplement: Figure S2 — Lower bound of pollination benefits. Values are given as US $ for the year 2000. The values have been corrected for inflation (to the year 2009) as well as for purchasing power parities. The area we relate yields to is the total area of the raster cell. (PDF) [file pone.0035954.s002.pdf]

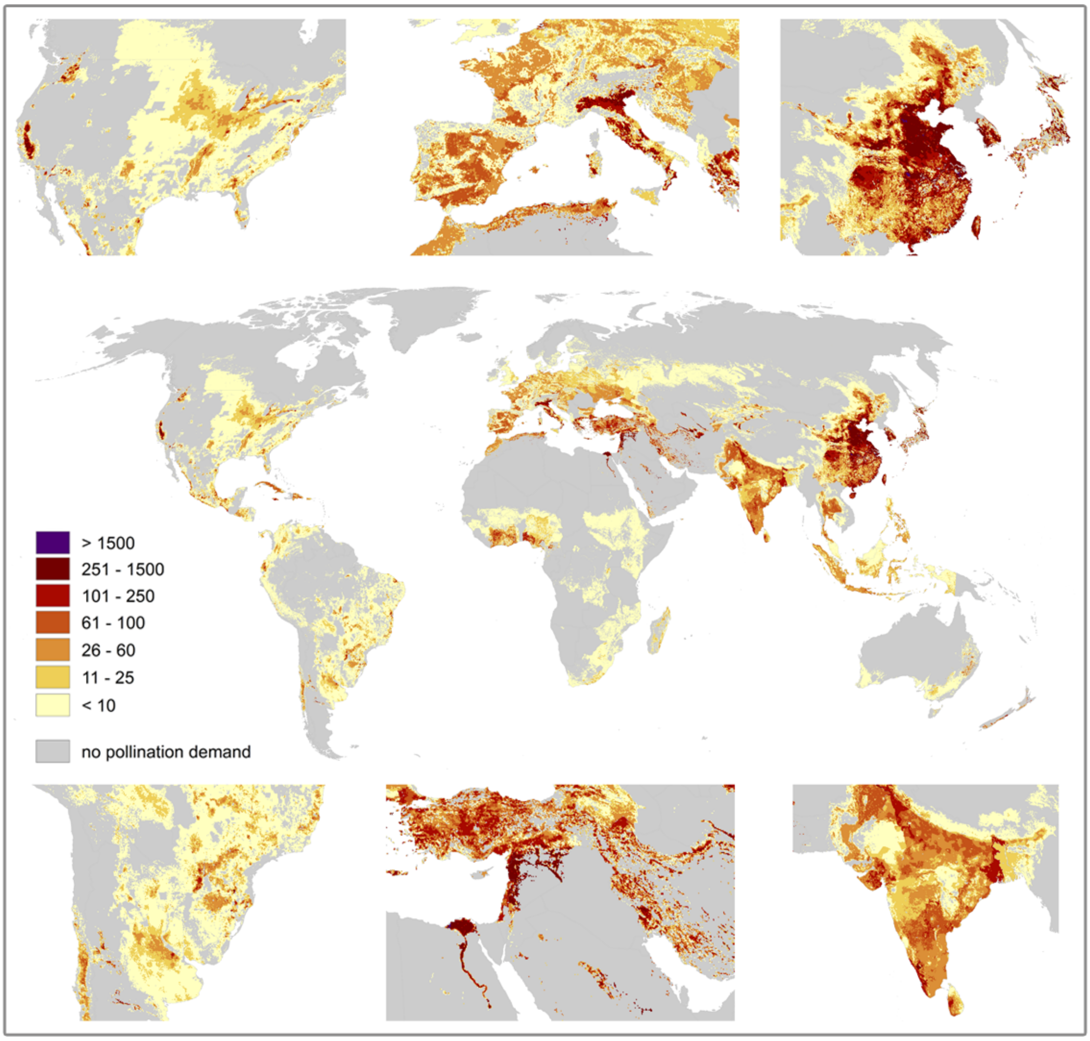

Figure S2. Lower bound of pollination benefits. Values are given as US $ for the year 2000. The values have been corrected for inflation (to the year 2009) as well as for purchasing power parities. The area we relate yields to is the total area of the raster cell.
