# Supplementary material for: Spatial and Temporal Trends of Global Pollination Benefit
Source: PLoS One. 2012 Apr 26;7(4):e35954. doi: 10.1371/journal.pone.0035954 (PMC3338563; doi:10.1371/journal.pone.0035954)
Supplement: Figure S5 — Temporal trends of the vulnerability indicator for individual countries. The 80 countries with the highest average part of the agricultural GDP that depends on pollination benefits have been selected for display. Values above 100% indicate incompatibilities between FAO and World Bank data. (PDF) [file pone.0035954.s005.pdf]

Figure S5. Temporal trend of vulnerability indicator for individual countries. The 80 countries with the highest average part of the agricultural GDP that depends on pollination benefits have been selected for display. Values above 100% indicate incompatibilities between FAO and World Bank data.

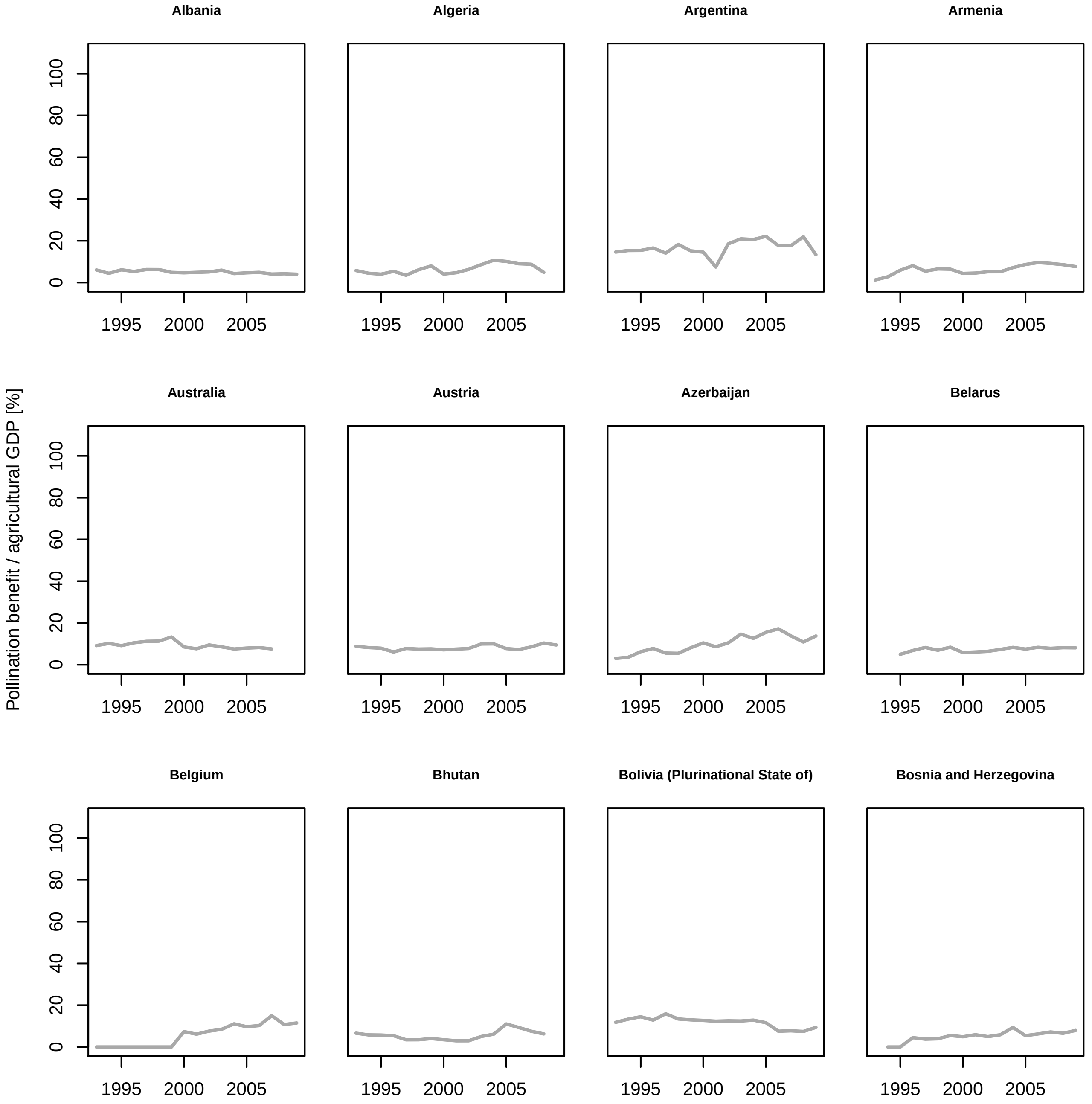
Albania
Algeria
Argentina
Armenia
Australia
Austria
Azerbaijan
Belarus
Belgium
Bhutan
Bolivia (Plurinational State of)
Bosnia and Herzegovina
Pollination benefit / agricultural GDP [%]
0
20
40
60
80
100
1995
2000
2005

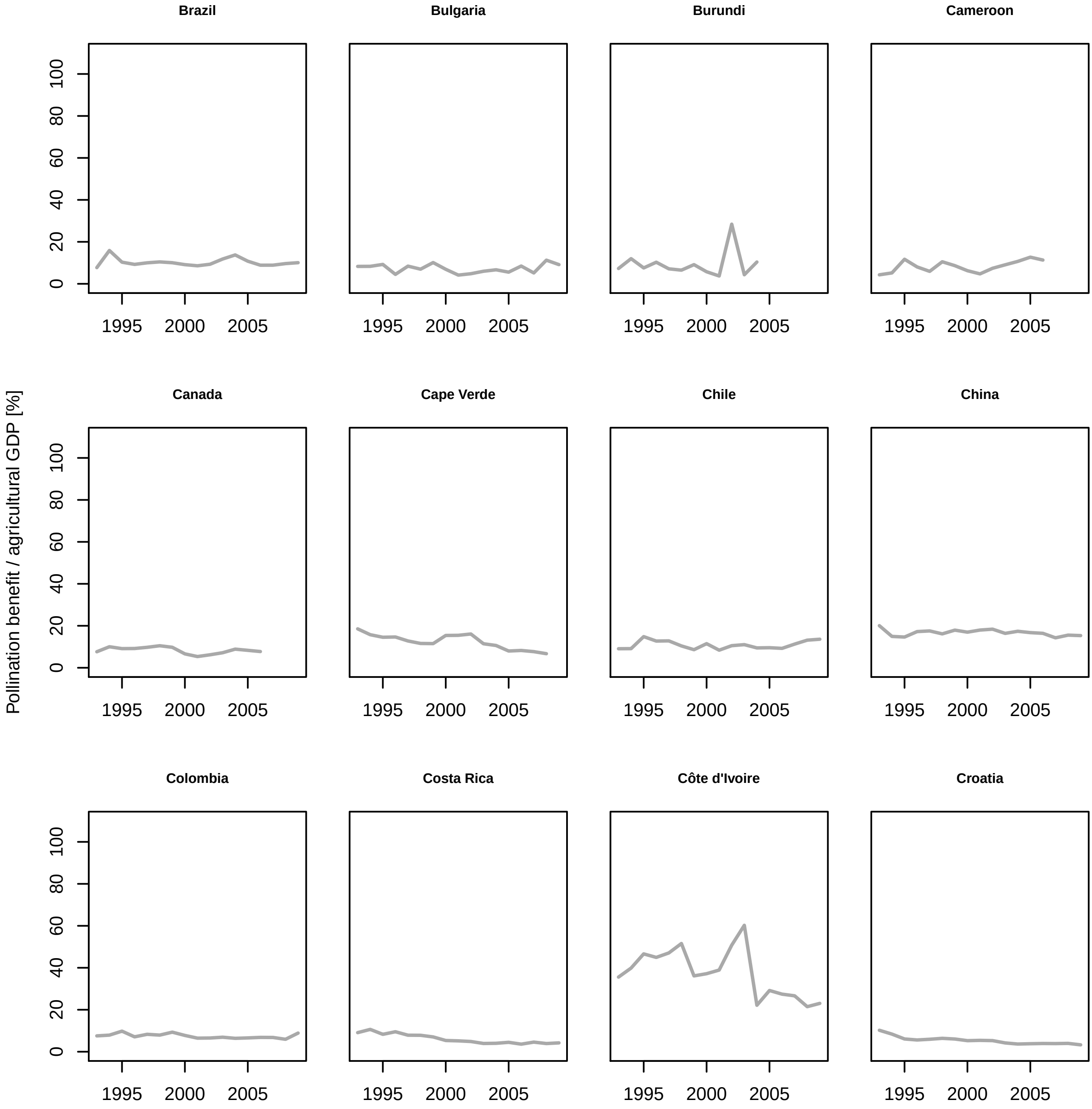
Brazil
Bulgaria
Burundi
Cameroon
Canada
Cape Verde
Chile
China
Colombia
Costa Rica
Côte d'Ivoire
Croatia
Pollination benefit / agricultural GDP [%]
0
20
40
60
80
100
1995
2000
2005

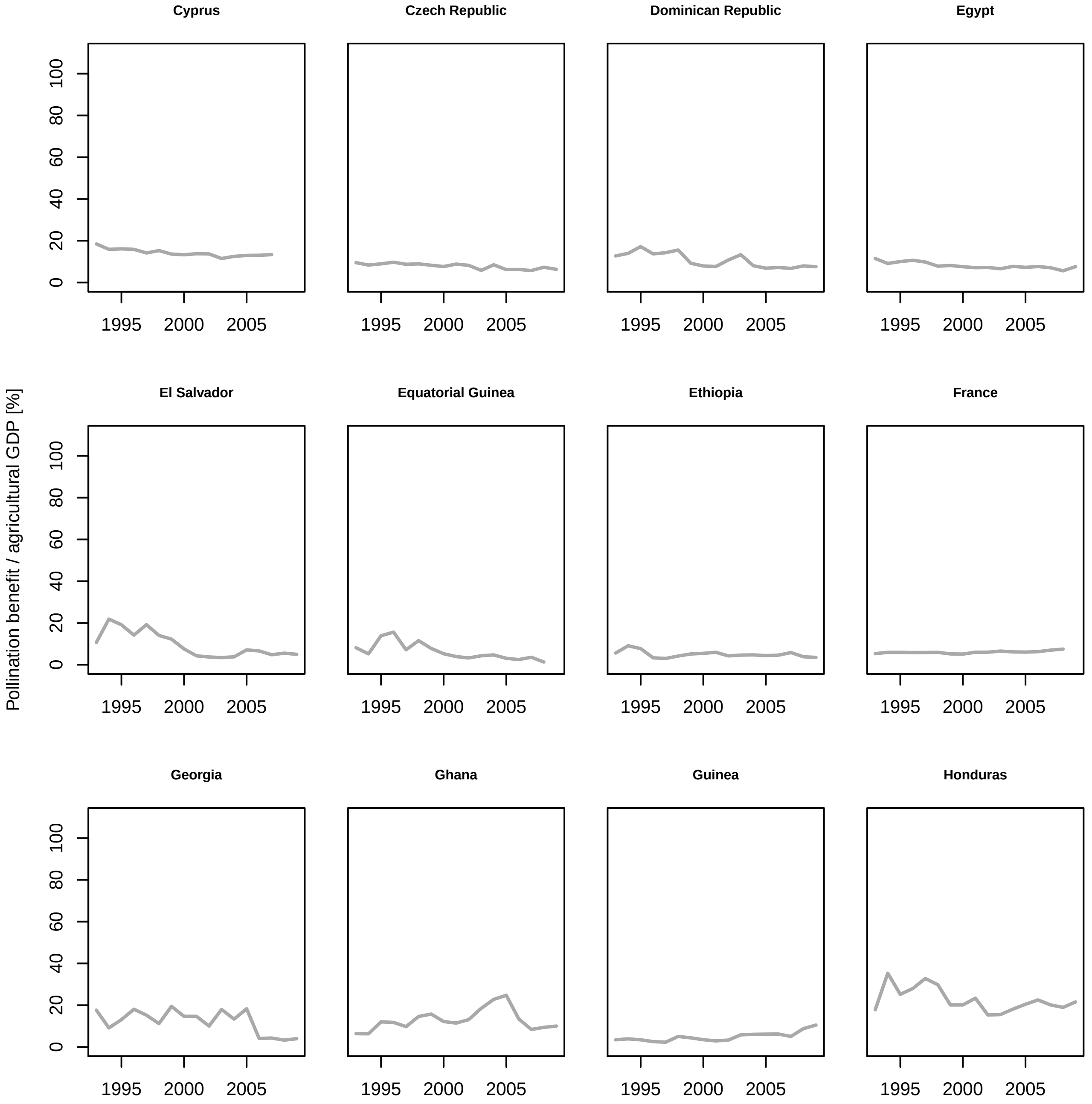

Cyprus
Czech Republic
Dominican Republic
Egypt
El Salvador
Equatorial Guinea
Ethiopia
France
Georgia
Ghana
Guinea
Honduras
Pollination benefit / agricultural GDP [%]
0
20
40
60
80
100
1995
2000
2005

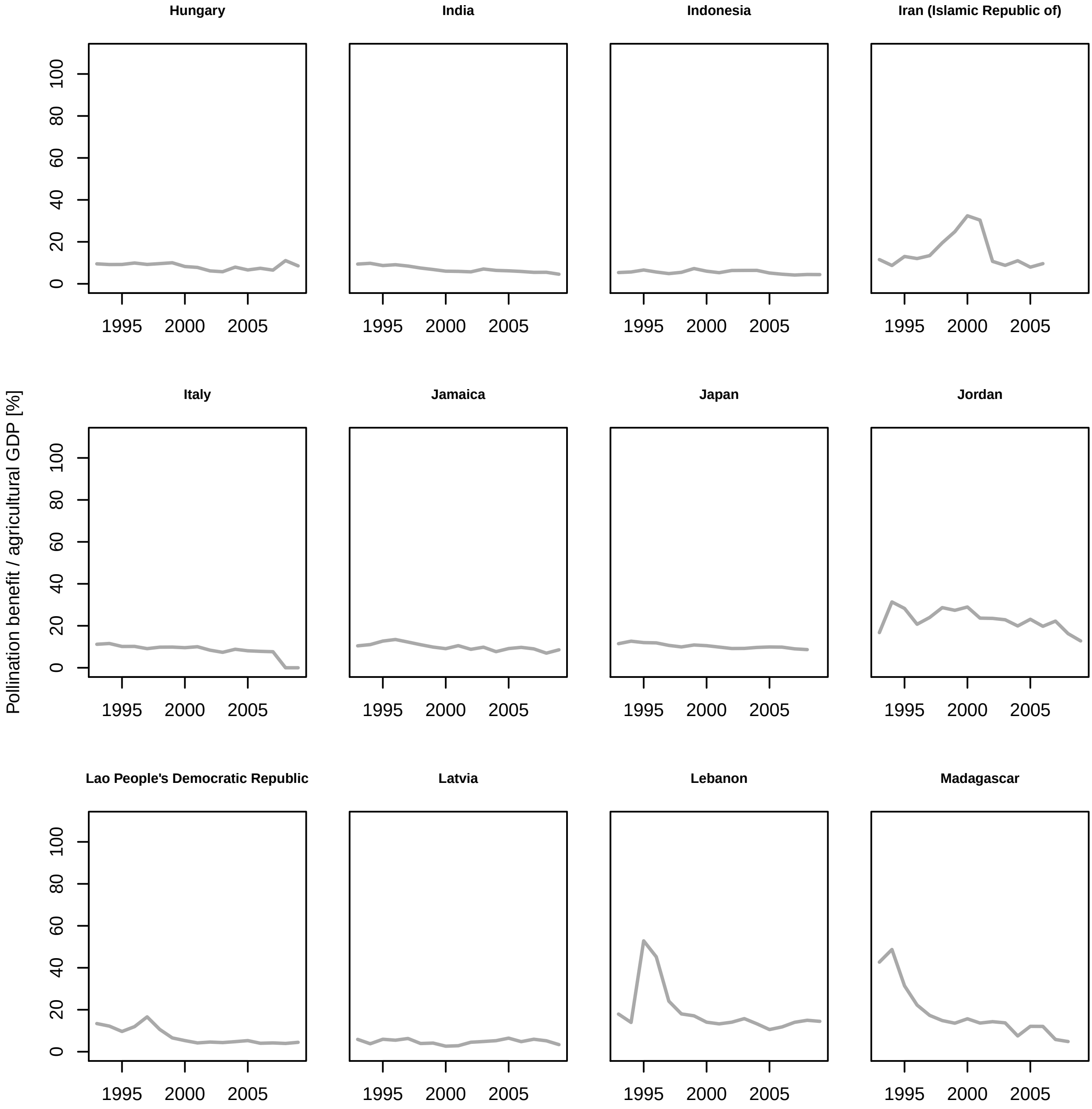

Hungary
India
Indonesia
Iran (Islamic Republic of)
Italy
Jamaica
Japan
Jordan
Lao People's Democratic Republic
Latvia
Lebanon
Madagascar
Pollination benefit / agricultural GDP [%]
0
20
40
60
80
100
1995
2000
2005

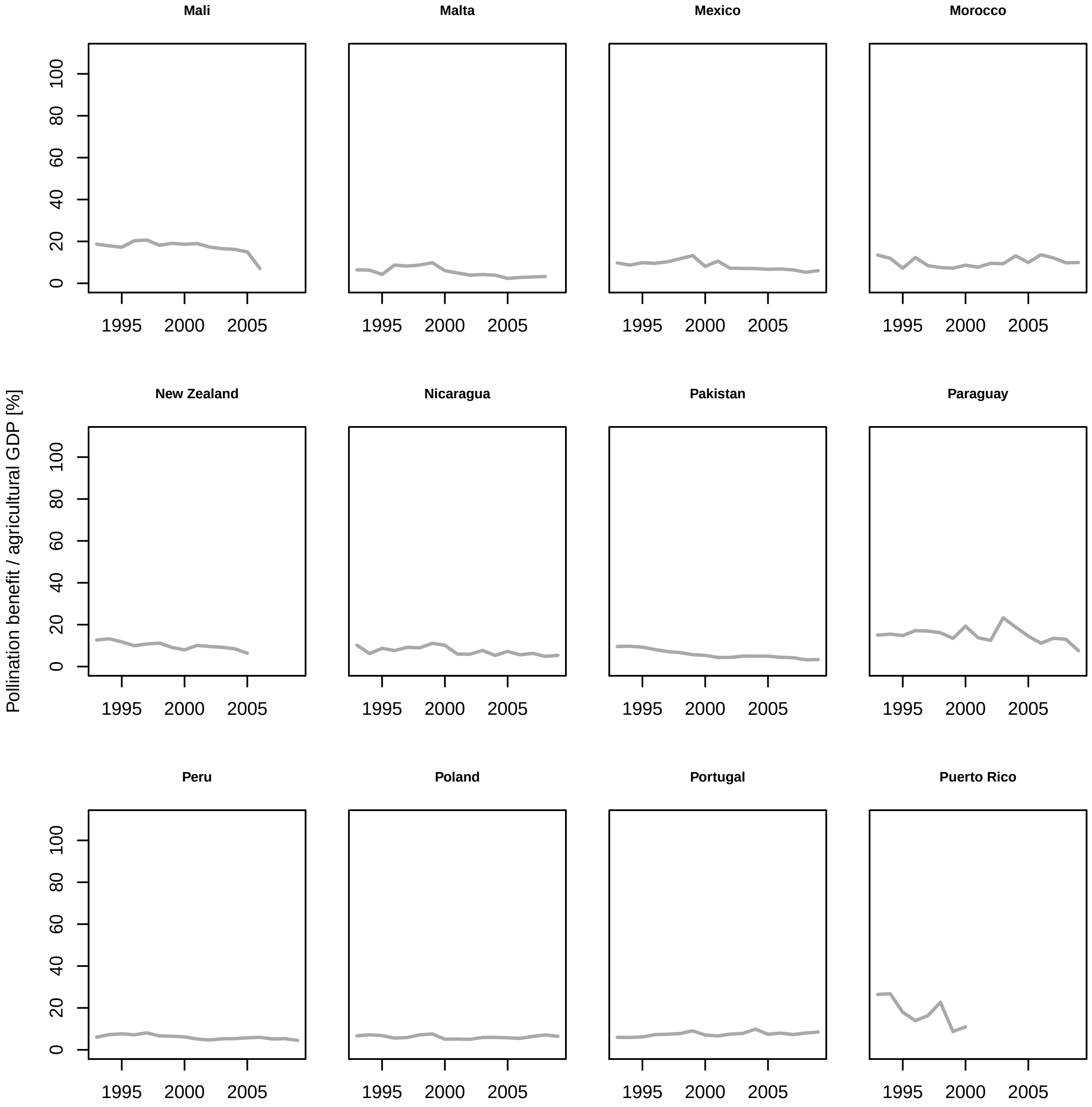

Mali
Malta
Mexico
Morocco
New Zealand
Nicaragua
Pakistan
Paraguay
Peru
Poland
Portugal
Puerto Rico
Pollination benefit / agricultural GDP [%]
0
20
40
60
80
100
1995
2000
2005

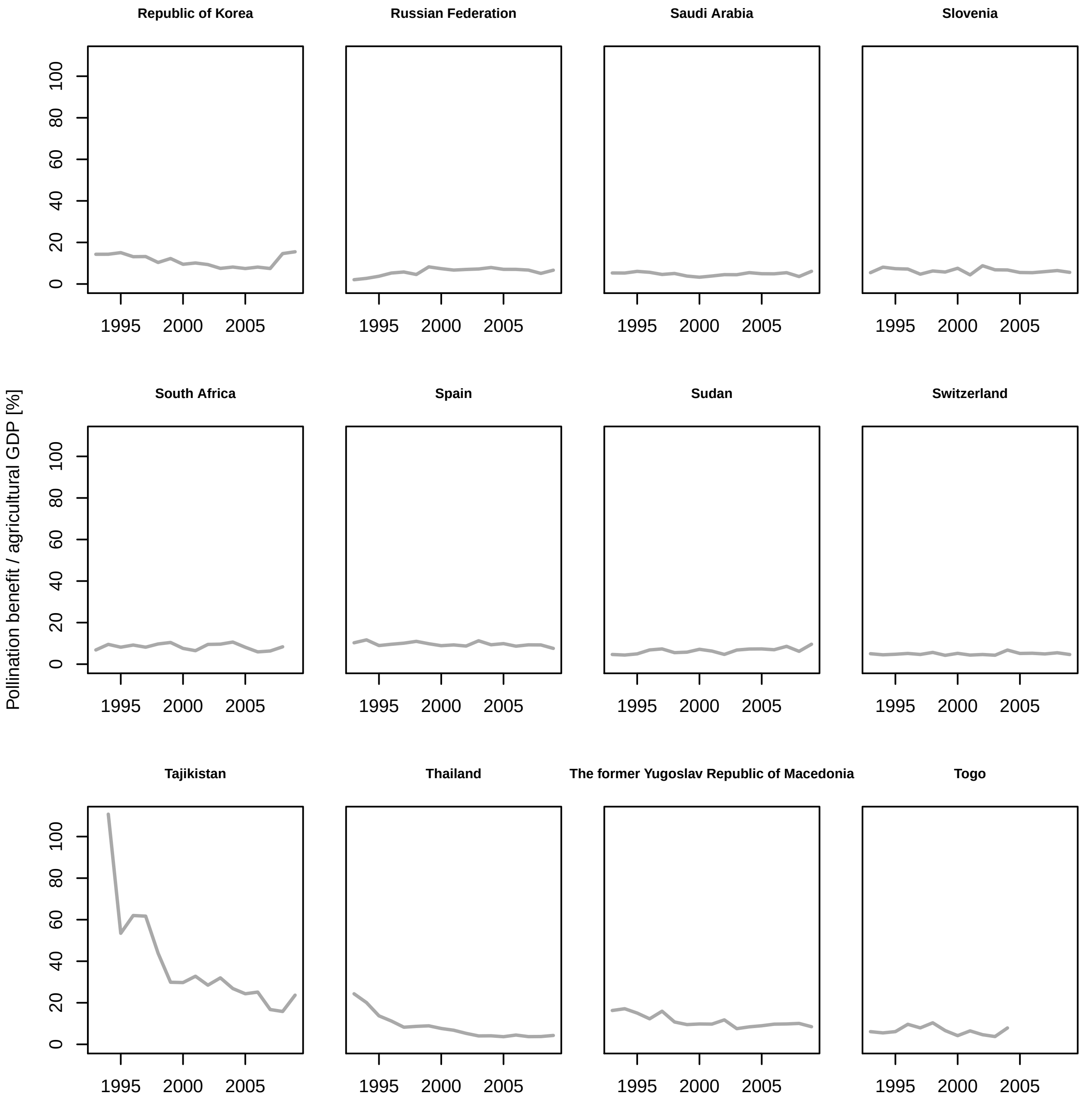
Republic of Korea
Russian Federation
Saudi Arabia
Slovenia
South Africa
Spain
Sudan
Switzerland
Tajikistan
Thailand
The former Yugoslav Republic of Macedonia
Togo
Pollination benefit / agricultural GDP [%]
0
20
40
60
80
100
1995
2000
2005

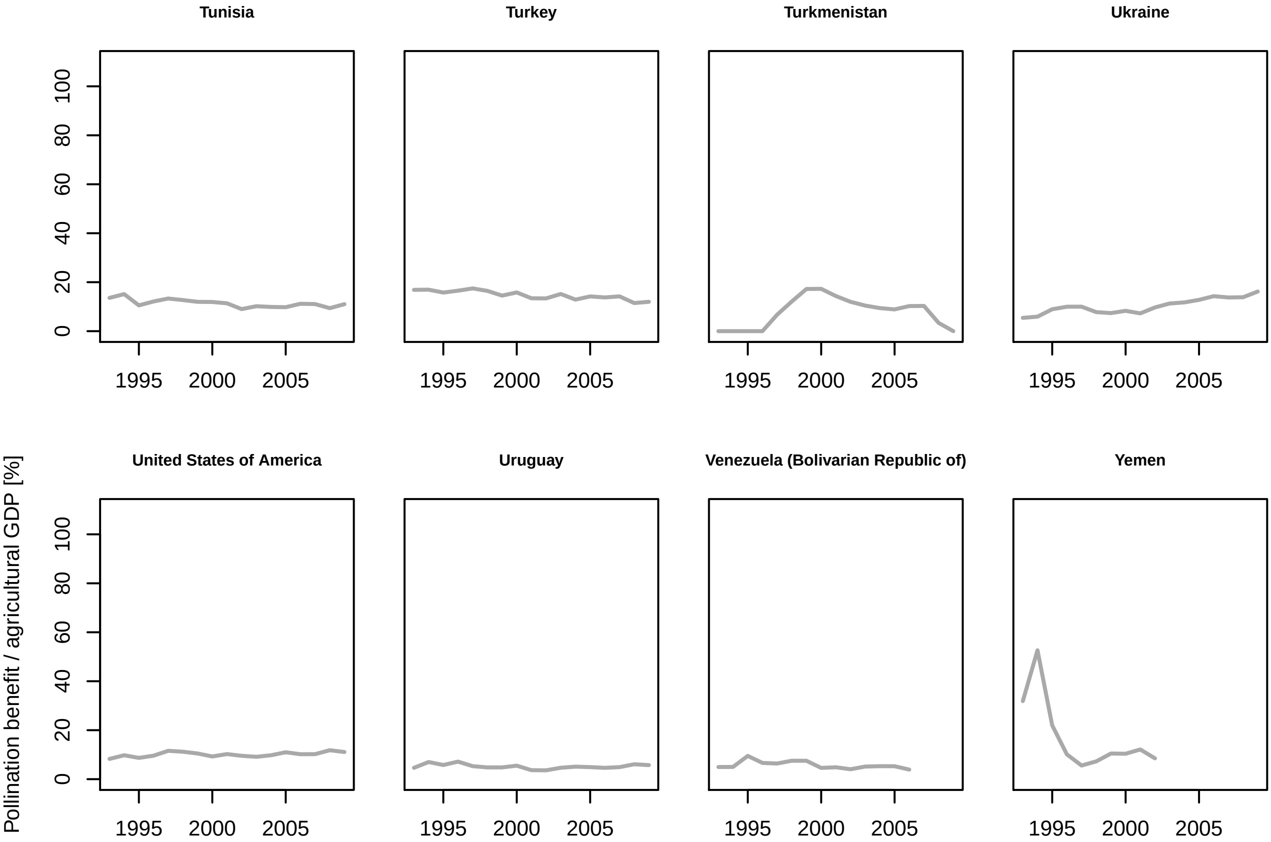
Tunisia
Turkey
Turkmenistan
Ukraine
United States of America
Uruguay
Venezuela (Bolivarian Republic of)
Yemen
Pollination benefit / agricultural GDP [%]
0
20
40
60
80
100
1995
2000
2005
