# Supplementary material for: Spatial and Temporal Trends of Global Pollination Benefit
Source: PLoS One. 2012 Apr 26;7(4):e35954. doi: 10.1371/journal.pone.0035954 (PMC3338563; doi:10.1371/journal.pone.0035954)
Supplement: Figure S6 — Trends for pollination benefits per crop for the 10 most important producing countries. (PDF) [file pone.0035954.s006.pdf]

Figure S6. Trends for pollination benefits per crop for the 10 most important producing countries.

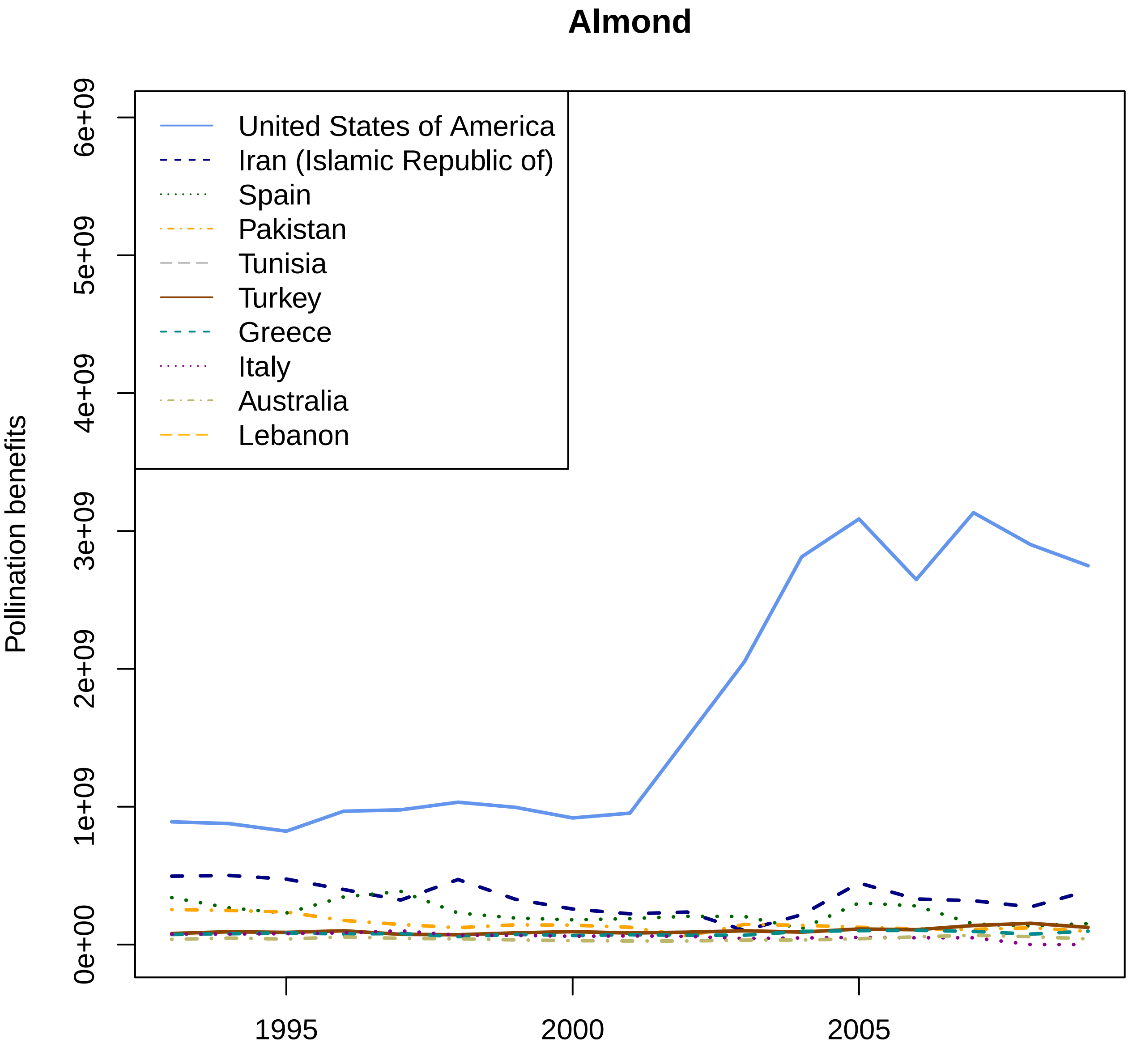

Almond
Pollination benefits
0e+00
1e+09
2e+09
3e+09
4e+09
5e+09
6e+09
1995
2000
2005
United States of America
Iran (Islamic Republic of)
Spain
Pakistan
Tunisia
Turkey
Greece
Italy
Australia
Lebanon

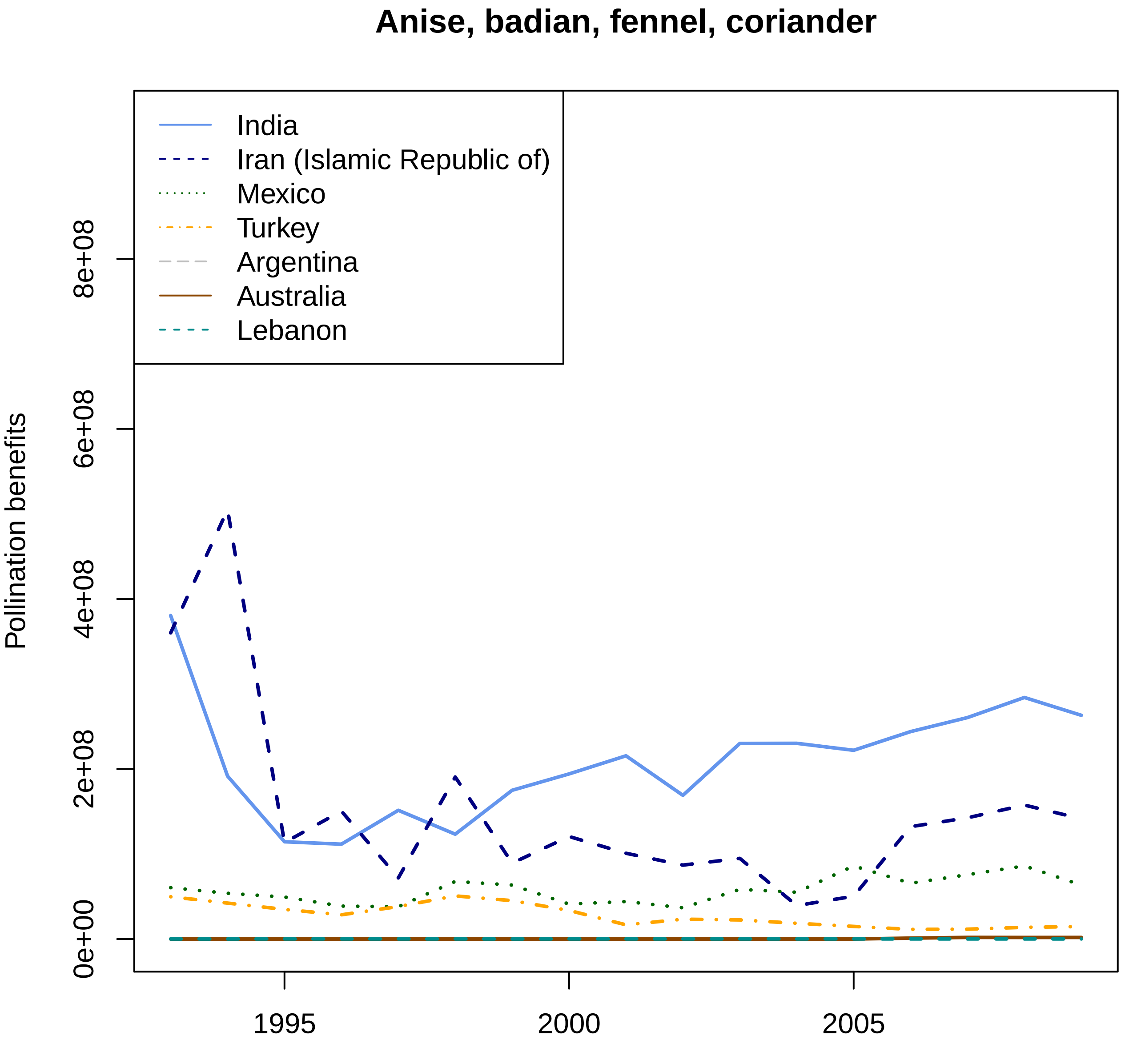
Anise, badian, fennel, coriander
India
Iran (Islamic Republic of)
Mexico
Turkey
Argentina
Australia
Lebanon
Pollination benefits
0e+00
2e+08
4e+08
6e+08
8e+08
1995
2000
2005

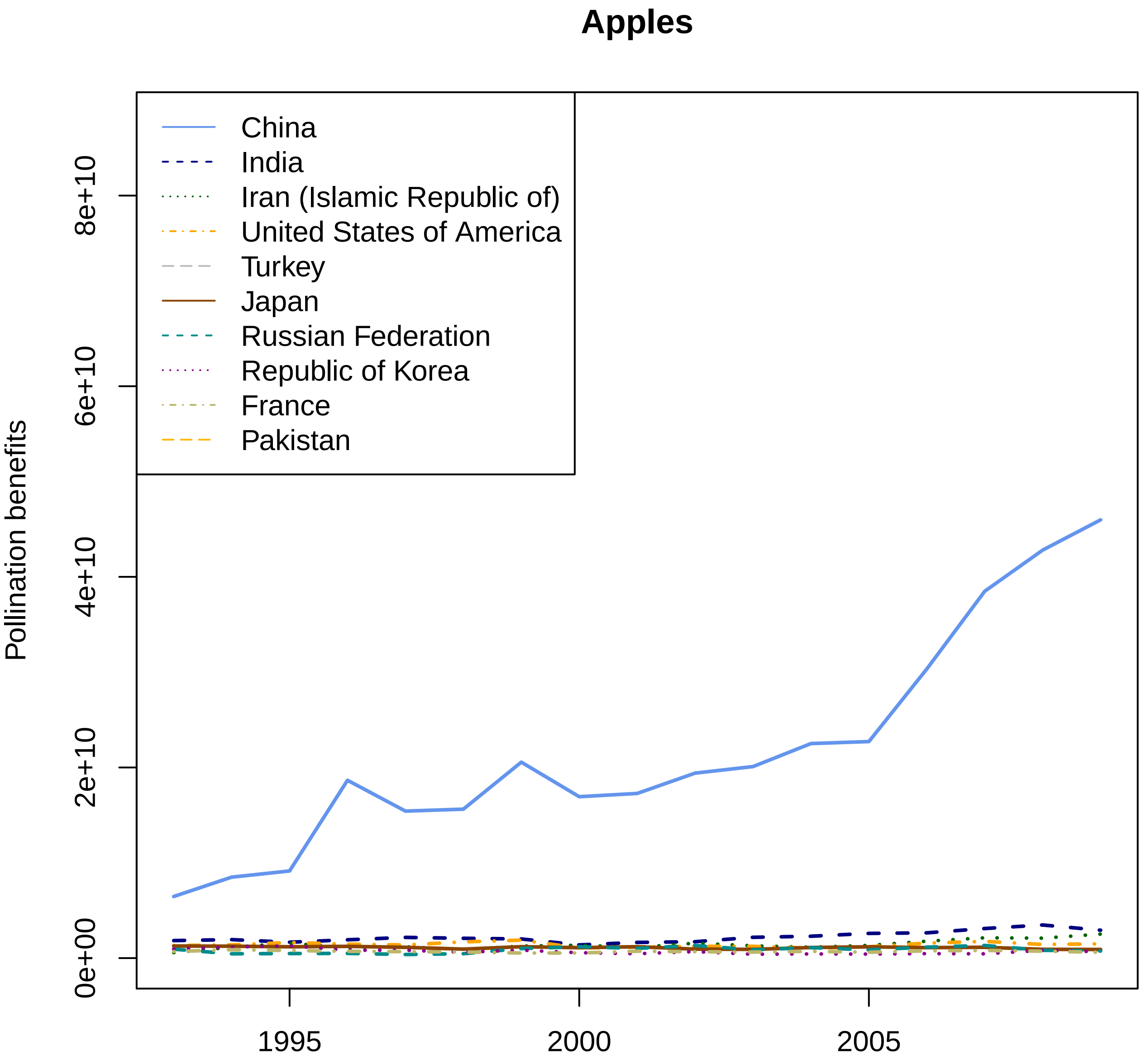
Apples
Pollination benefits
0e+00
2e+10
4e+10
6e+10
8e+10
1995
2000
2005
China
India
Iran (Islamic Republic of)
United States of America
Turkey
Japan
Russian Federation
Republic of Korea
France
Pakistan

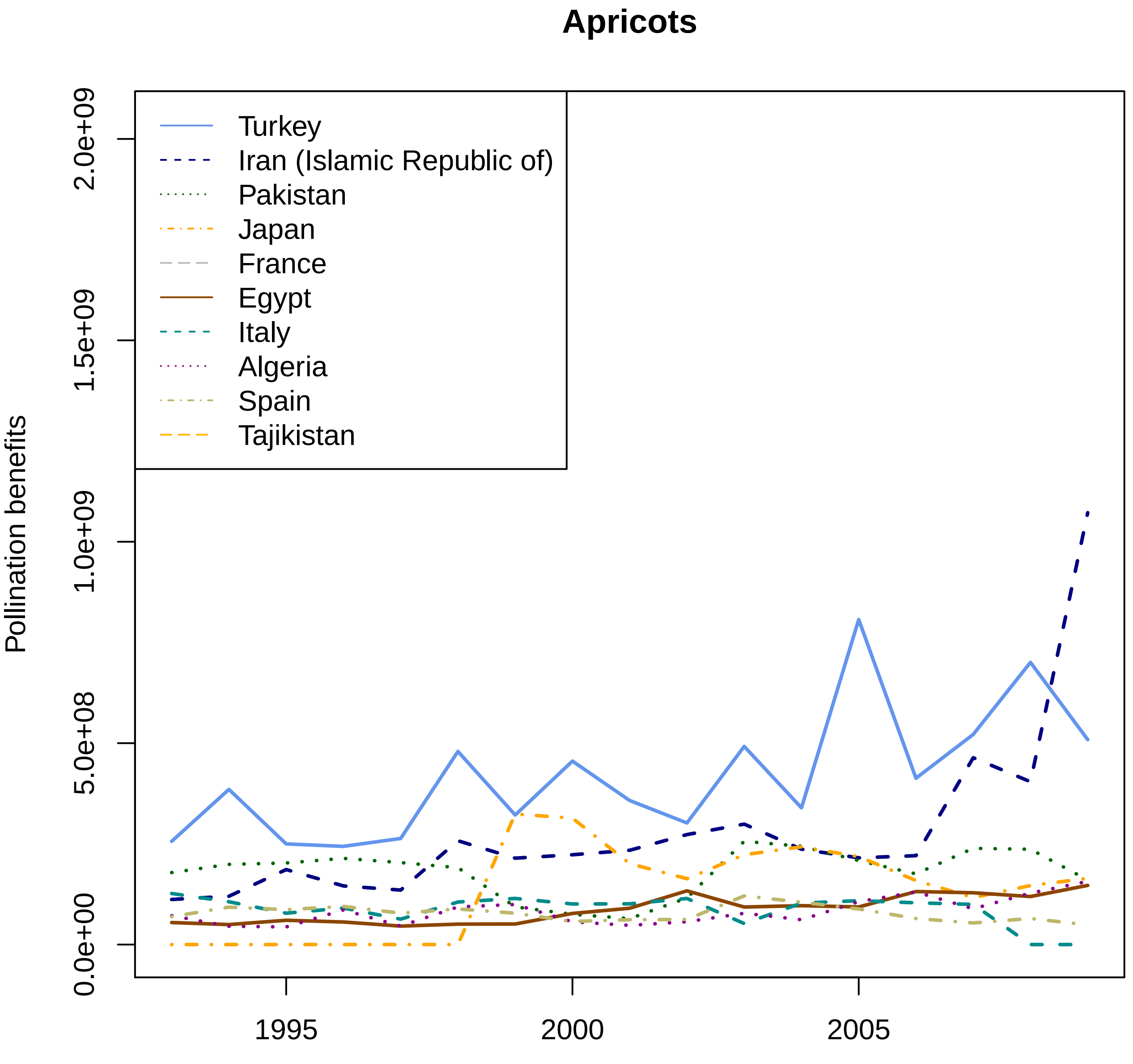
Apricots
Pollination benefits
0.0e+00
5.0e+08
1.0e+09
1.5e+09
2.0e+09
1995
2000
2005
Turkey
Iran (Islamic Republic of)
Pakistan
Japan
France
Egypt
Italy
Algeria
Spain
Tajikistan

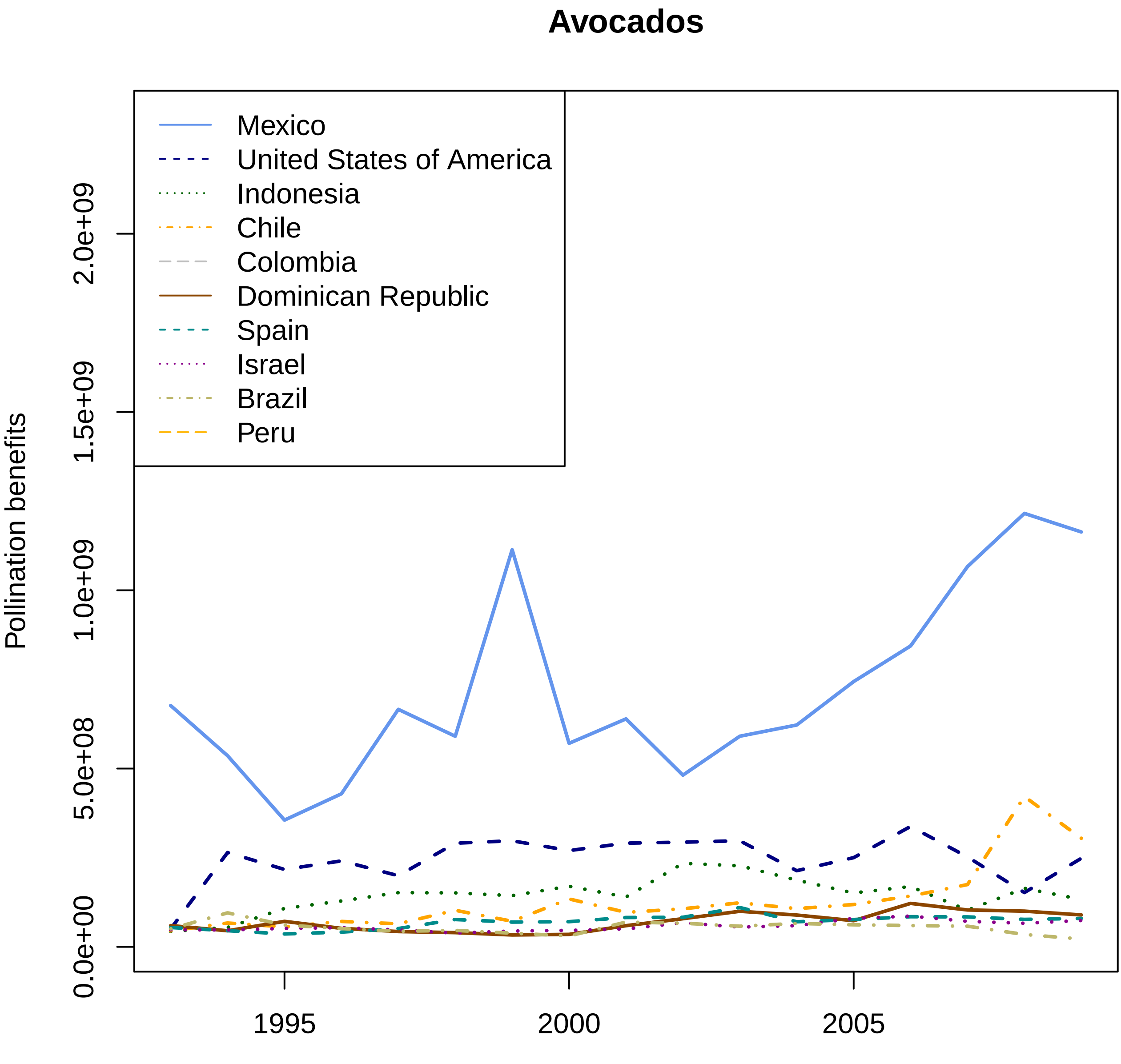

Avocados
Mexico
United States of America
Indonesia
Chile
Colombia
Dominican Republic
Spain
Israel
Brazil
Peru
Pollination benefits
0.0e+00
5.0e+08
1.0e+09
1.5e+09
2.0e+09
1995
2000
2005

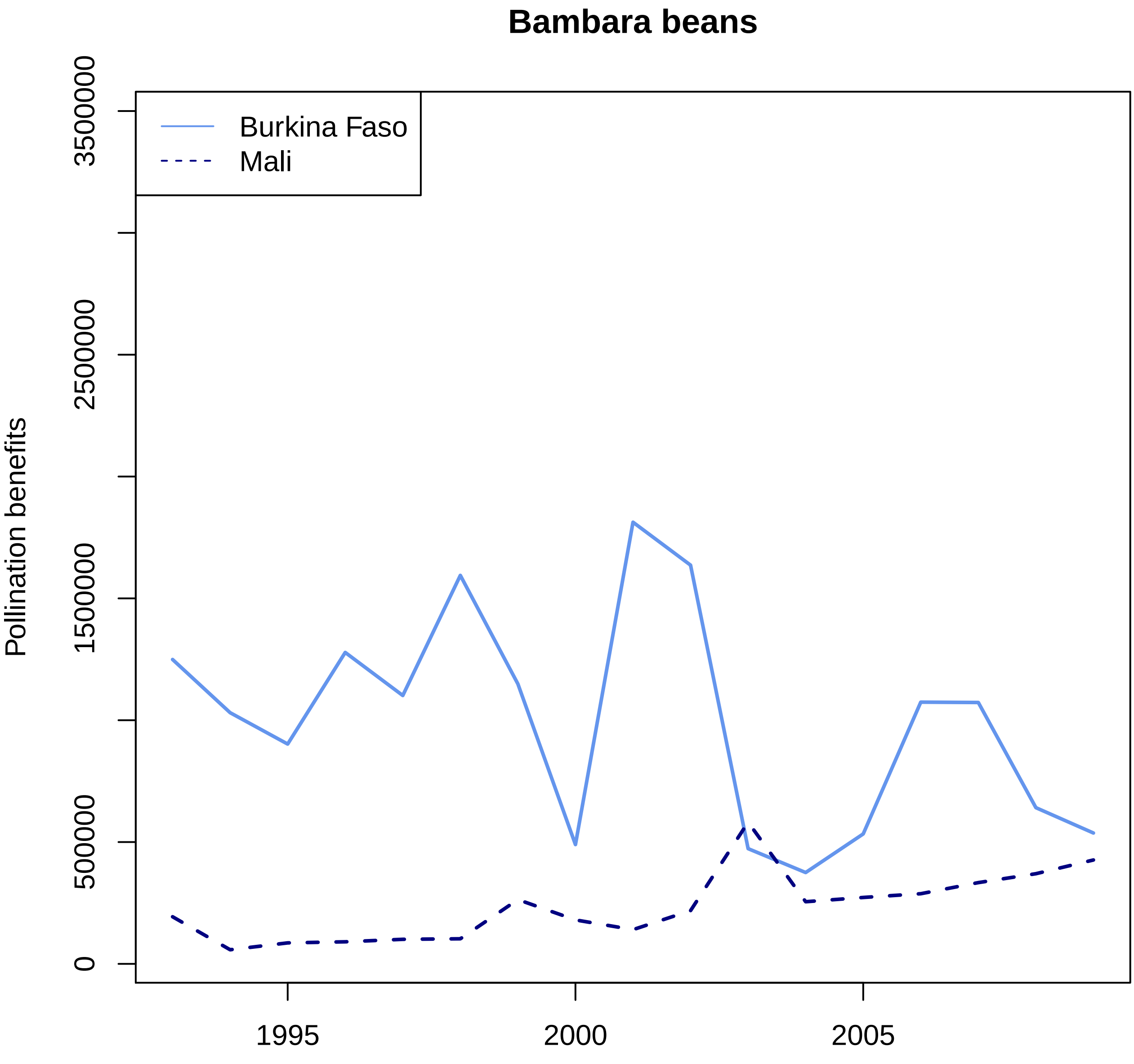

Bambara beans
Burkina Faso
Mali
Pollination benefits
0
500000
1500000
2500000
3500000
1995
2000
2005

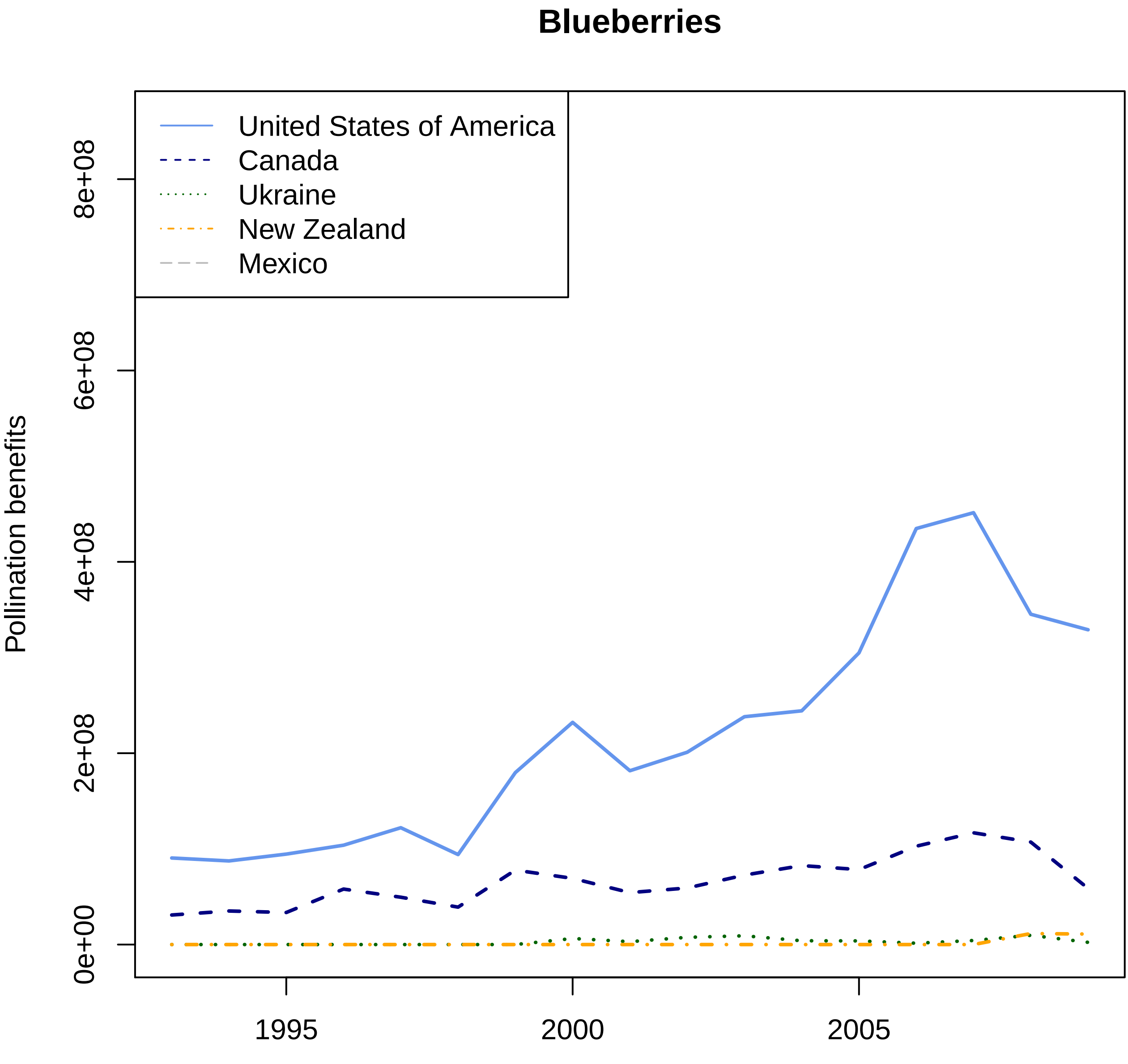
Blueberries
United States of America
Canada
Ukraine
New Zealand
Mexico
Pollination benefits
0e+00
2e+08
4e+08
6e+08
8e+08
1995
2000
2005

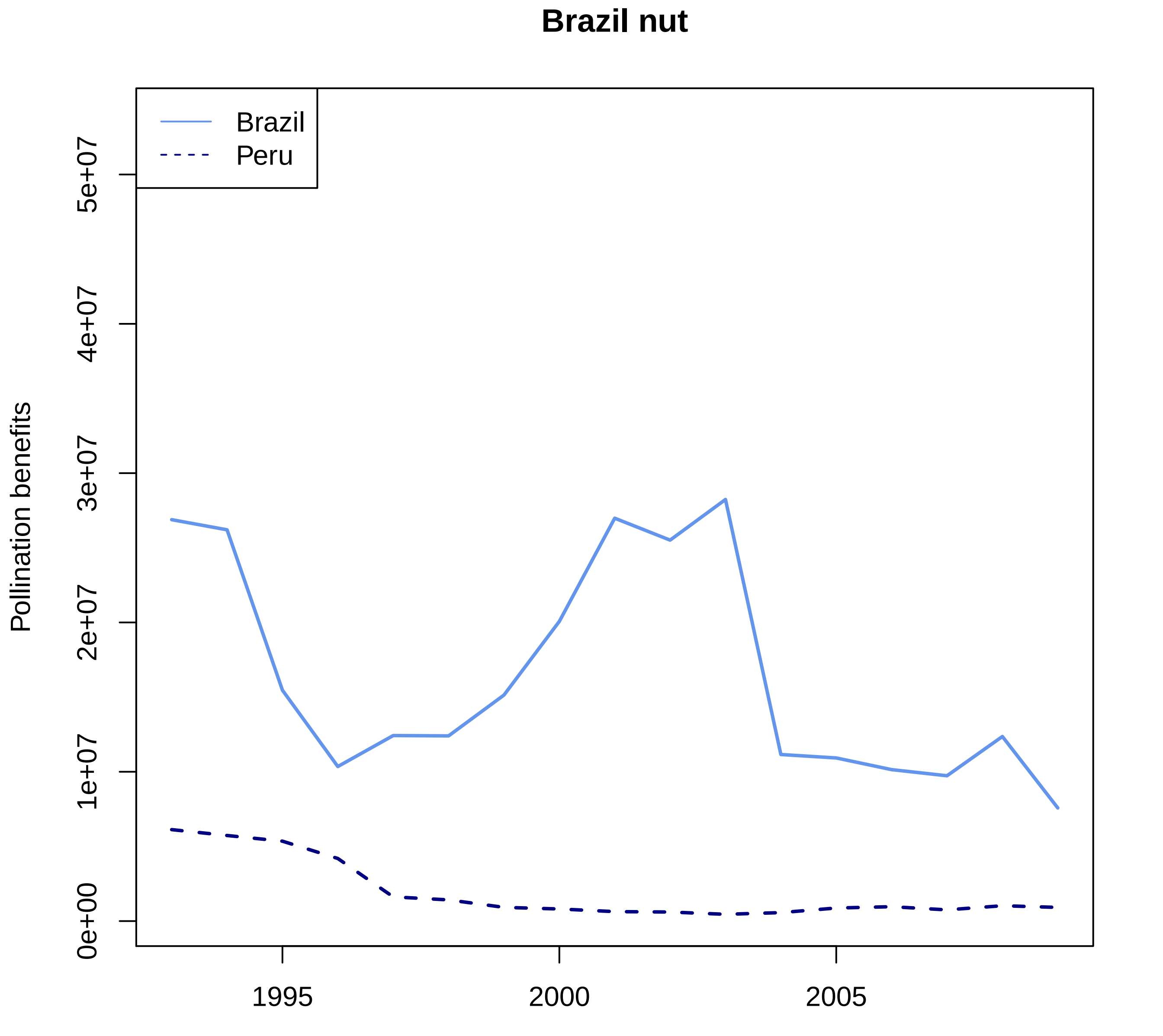

Brazil nut
Brazil
Peru
Pollination benefits
0e+00
1e+07
2e+07
3e+07
4e+07
5e+07
1995
2000
2005

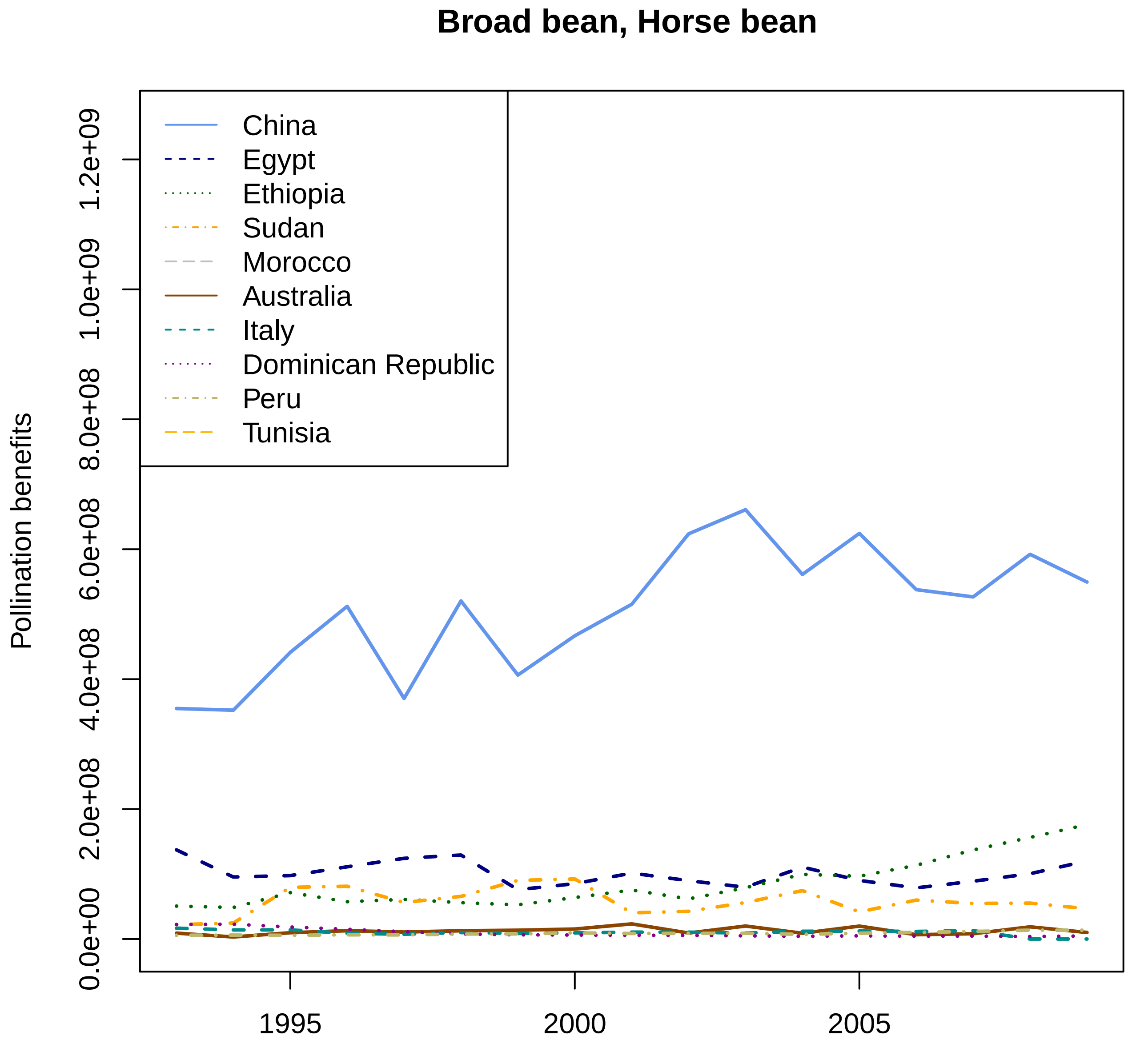
Broad bean, Horse bean
China
Egypt
Ethiopia
Sudan
Morocco
Australia
Italy
Dominican Republic
Peru
Tunisia
Pollination benefits
0.0e+00
2.0e+08
4.0e+08
6.0e+08
8.0e+08
1.0e+09
1.2e+09
1995
2000
2005

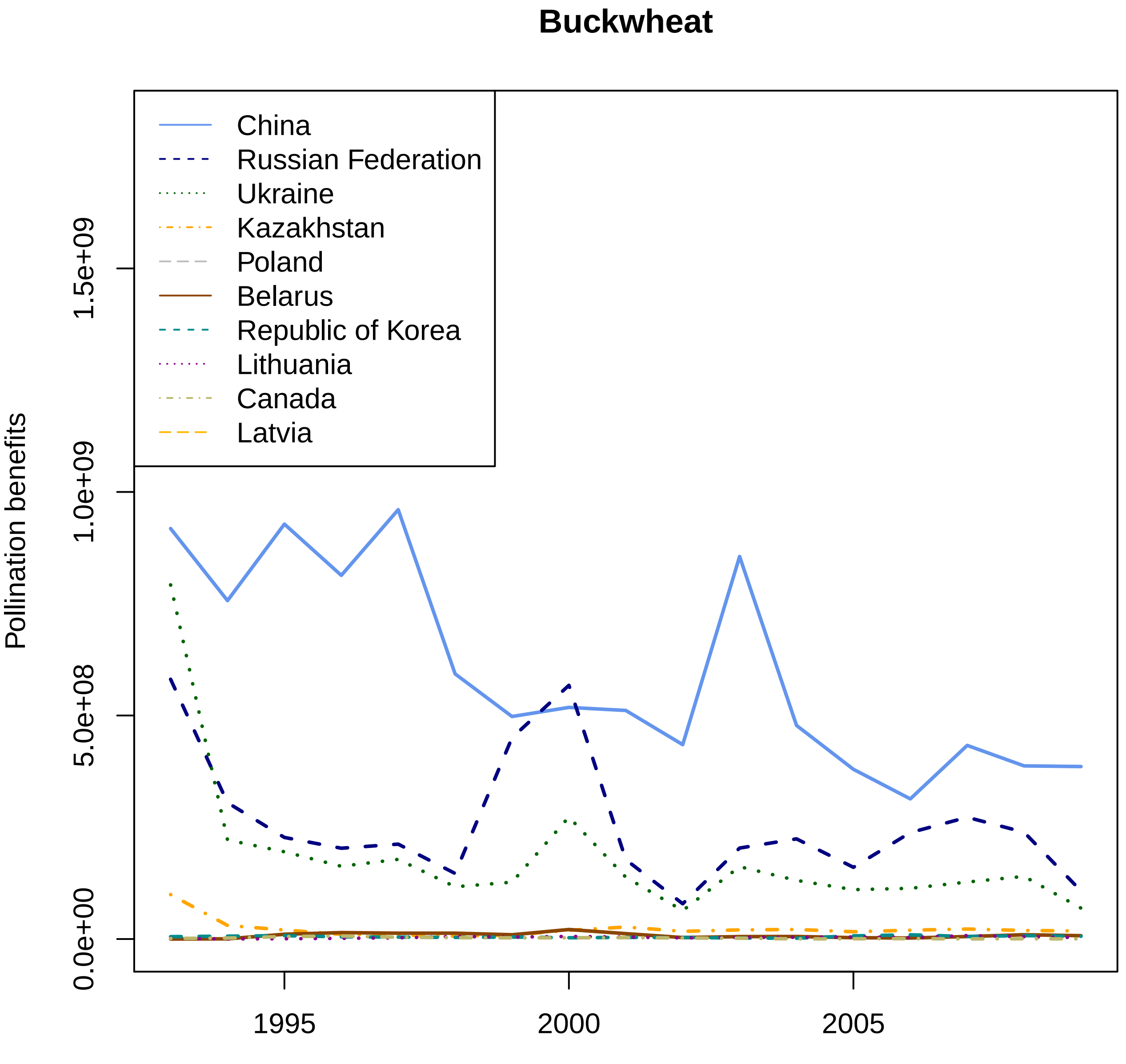

Buckwheat
China
Russian Federation
Ukraine
Kazakhstan
Poland
Belarus
Republic of Korea
Lithuania
Canada
Latvia
Pollination benefits
0.0e+00
5.0e+08
1.0e+09
1.5e+09
1995
2000
2005

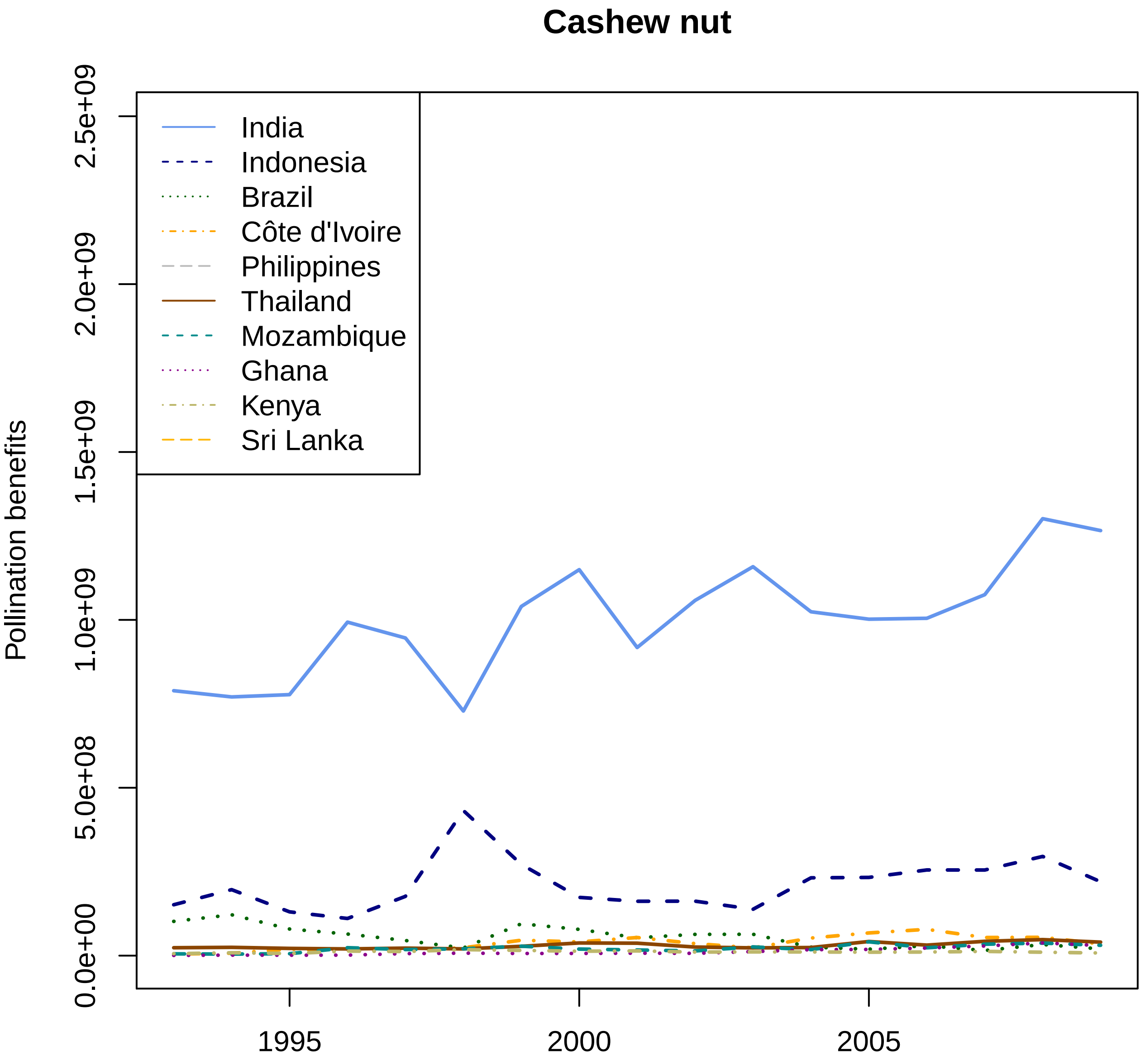
Cashew nut
India
Indonesia
Brazil
Côte d'Ivoire
Philippines
Thailand
Mozambique
Ghana
Kenya
Sri Lanka
Pollination benefits
0.0e+00
5.0e+08
1.0e+09
1.5e+09
2.0e+09
2.5e+09
1995
2000
2005

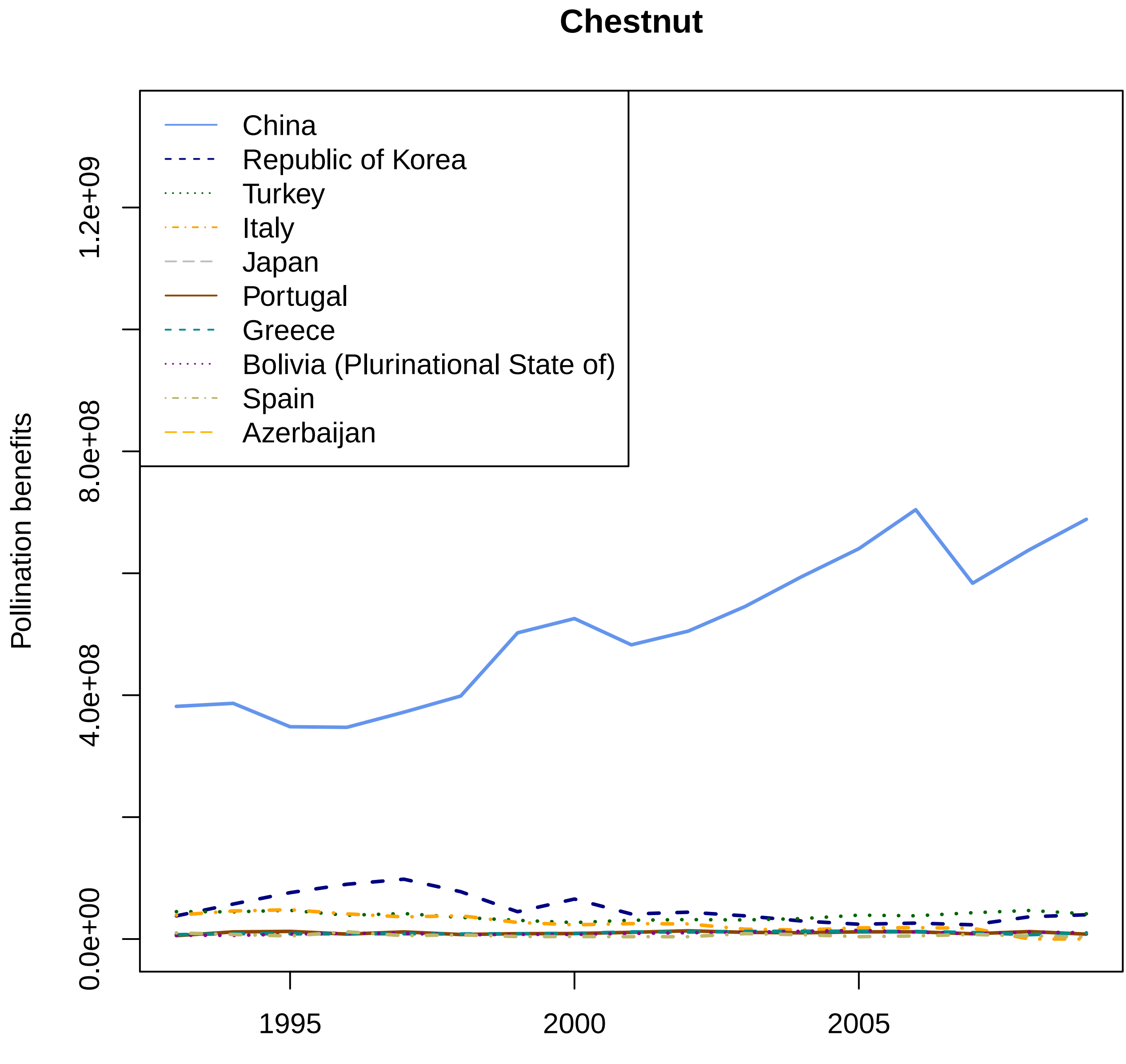
Chestnut
Pollination benefits
0.0e+00
4.0e+08
8.0e+08
1.2e+09
1995
2000
2005
China
Republic of Korea
Turkey
Italy
Japan
Portugal
Greece
Bolivia (Plurinational State of)
Spain
Azerbaijan

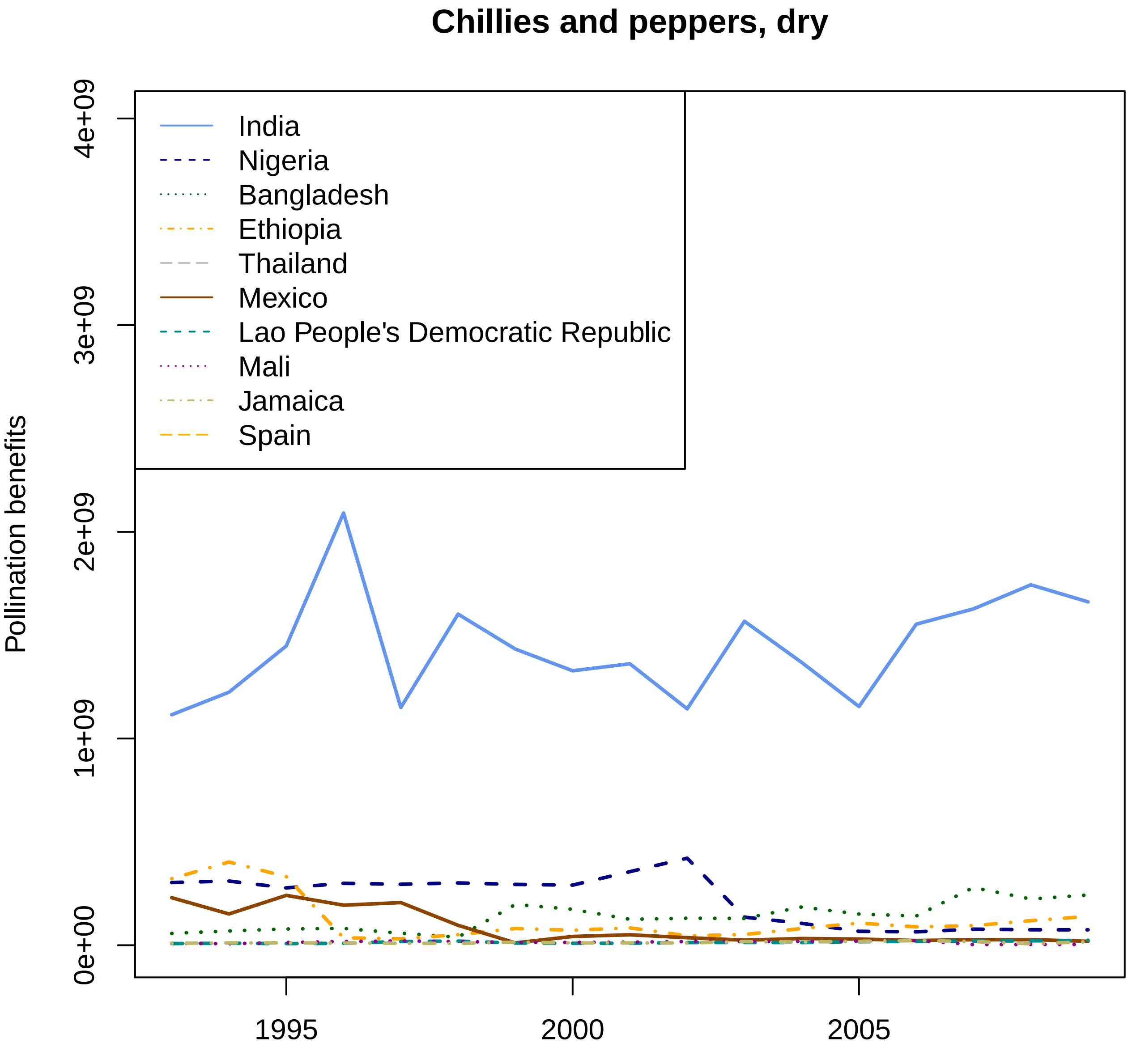

Chillies and peppers, dry
India
Nigeria
Bangladesh
Ethiopia
Thailand
Mexico
Lao People's Democratic Republic
Mali
Jamaica
Spain
Pollination benefits
0e+00
1e+09
2e+09
3e+09
4e+09
1995
2000
2005

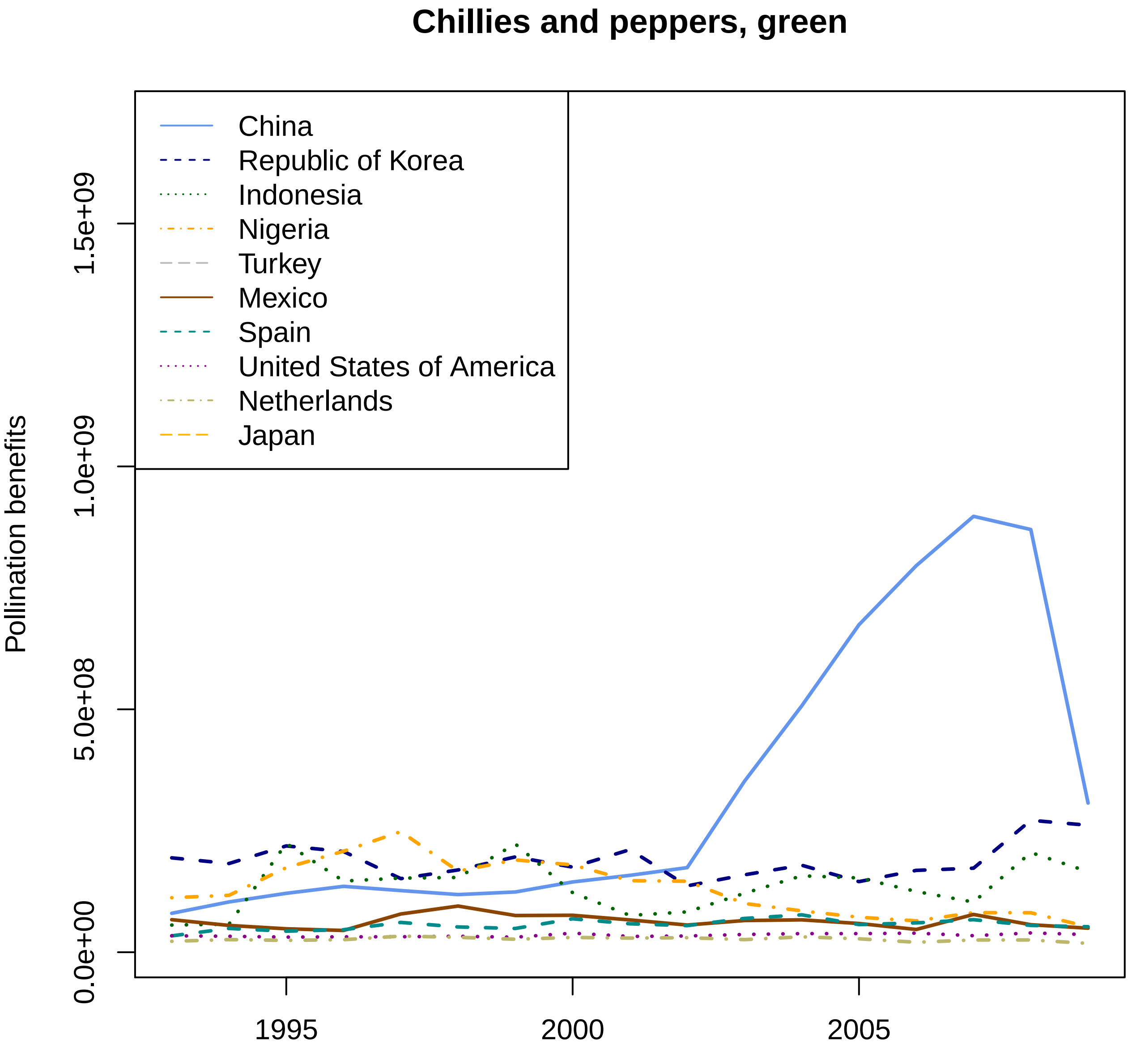

Chillies and peppers, green
Pollination benefits
0.0e+00
5.0e+08
1.0e+09
1.5e+09
1995
2000
2005
China
Republic of Korea
Indonesia
Nigeria
Turkey
Mexico
Spain
United States of America
Netherlands
Japan

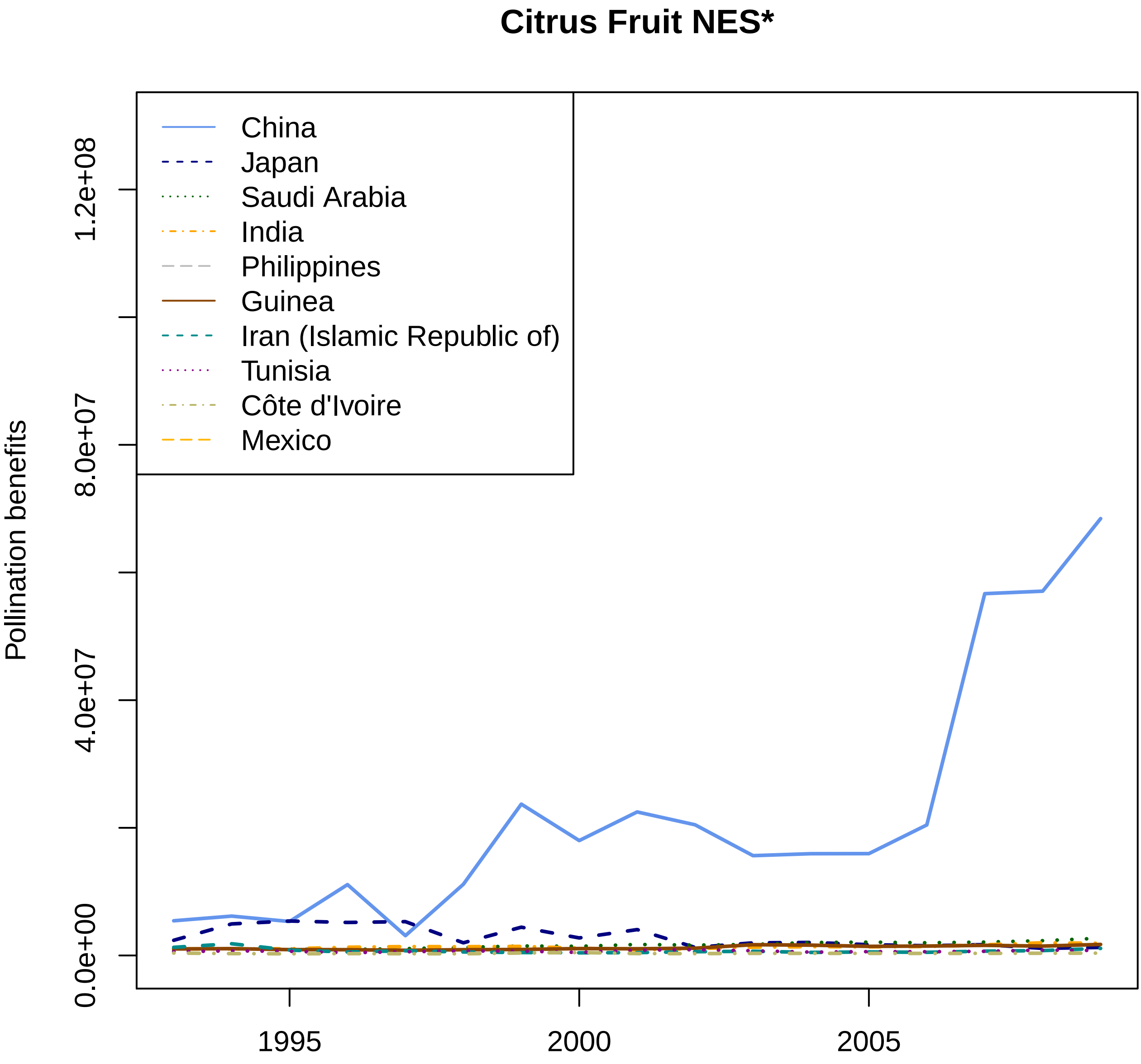

Citrus Fruit NES*
China
Japan
Saudi Arabia
India
Philippines
Guinea
Iran (Islamic Republic of)
Tunisia
Côte d'Ivoire
Mexico
Pollination benefits
0.0e+00
4.0e+07
8.0e+07
1.2e+08
1995
2000
2005

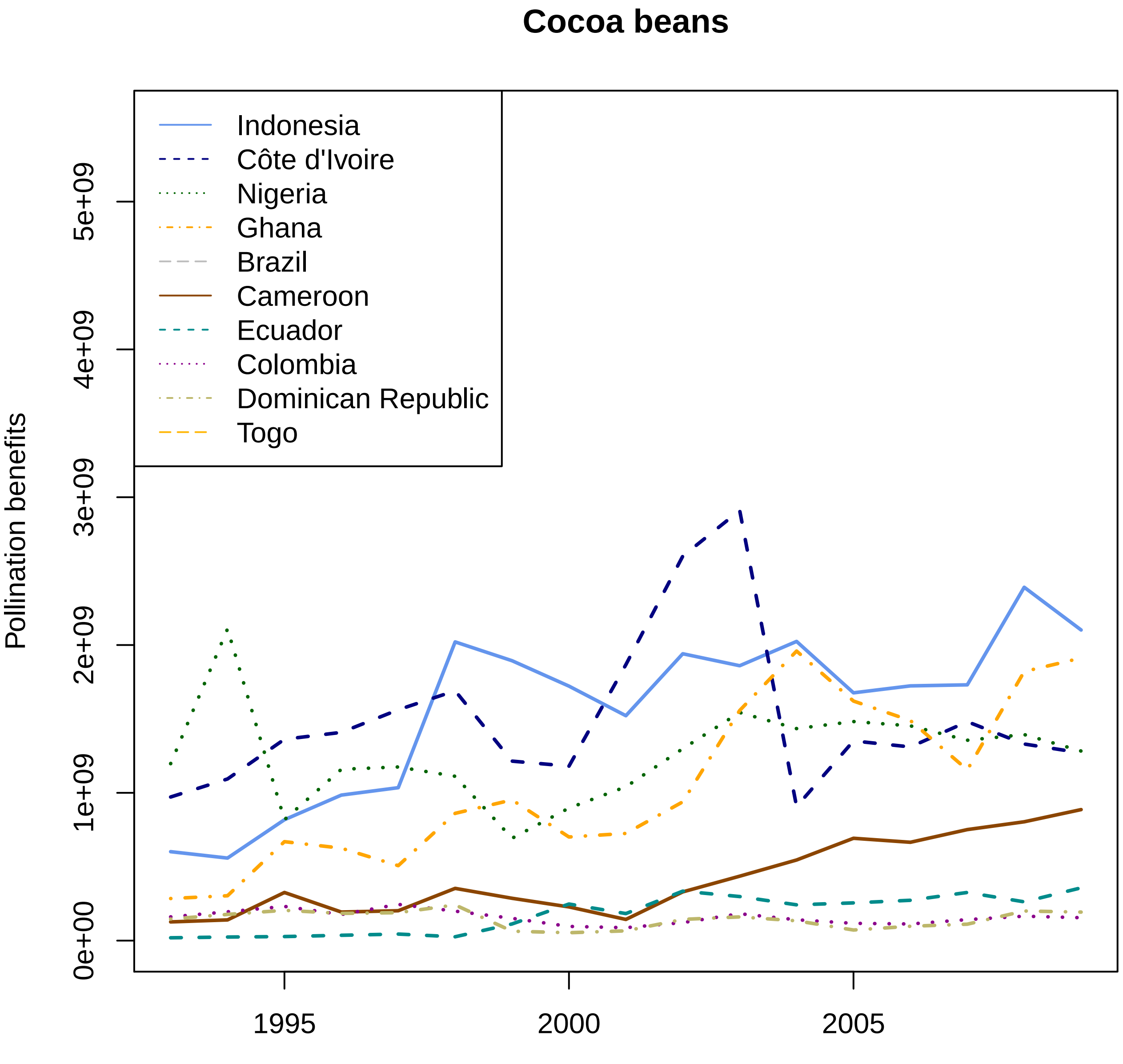

Cocoa beans
Indonesia
Côte d'Ivoire
Nigeria
Ghana
Brazil
Cameroon
Ecuador
Colombia
Dominican Republic
Togo
Pollination benefits
0e+00
1e+09
2e+09
3e+09
4e+09
5e+09
1995
2000
2005

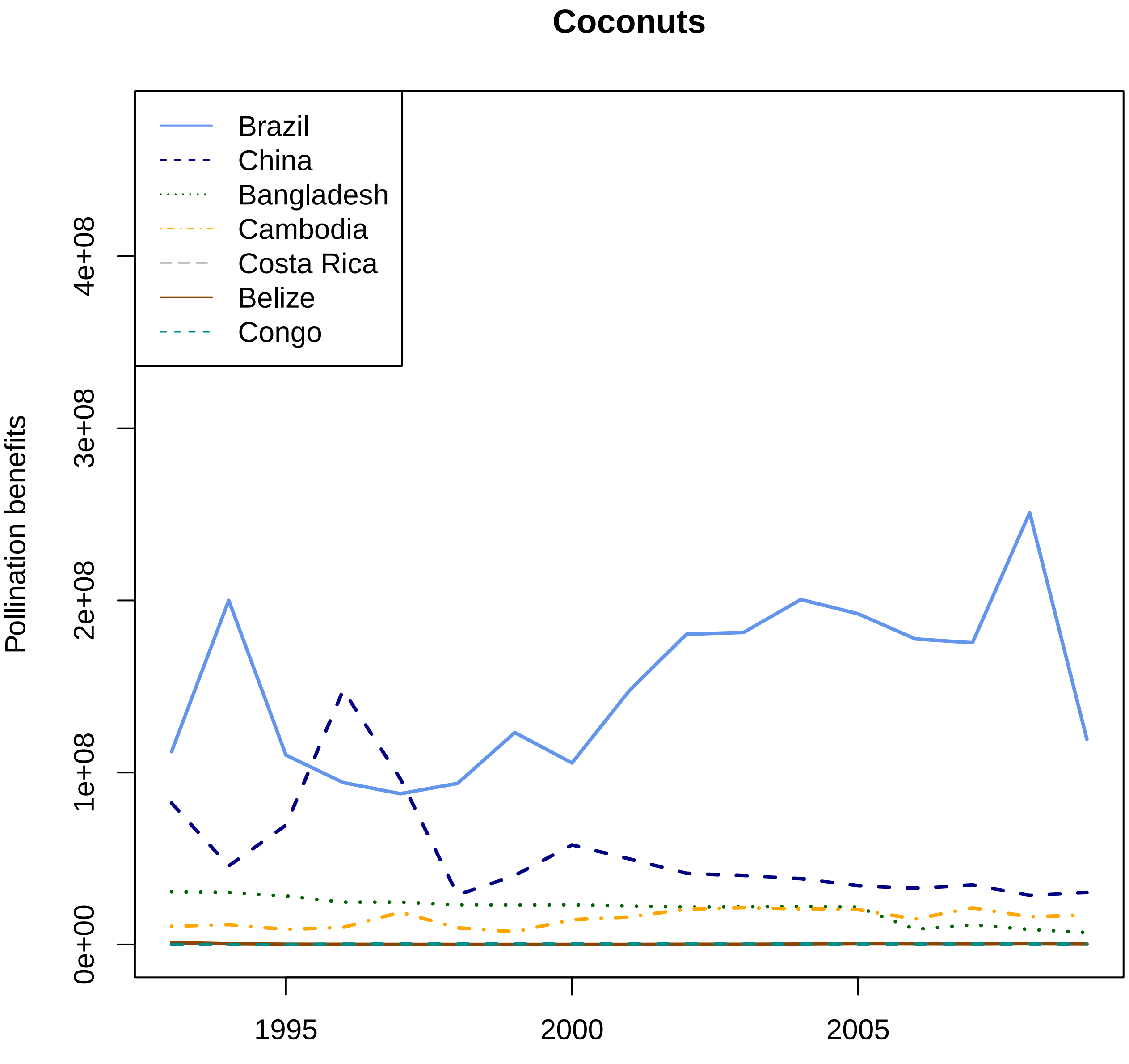
Coconuts
Pollination benefits
0e+00
1e+08
2e+08
3e+08
4e+08
1995
2000
2005
Brazil
China
Bangladesh
Cambodia
Costa Rica
Belize
Congo

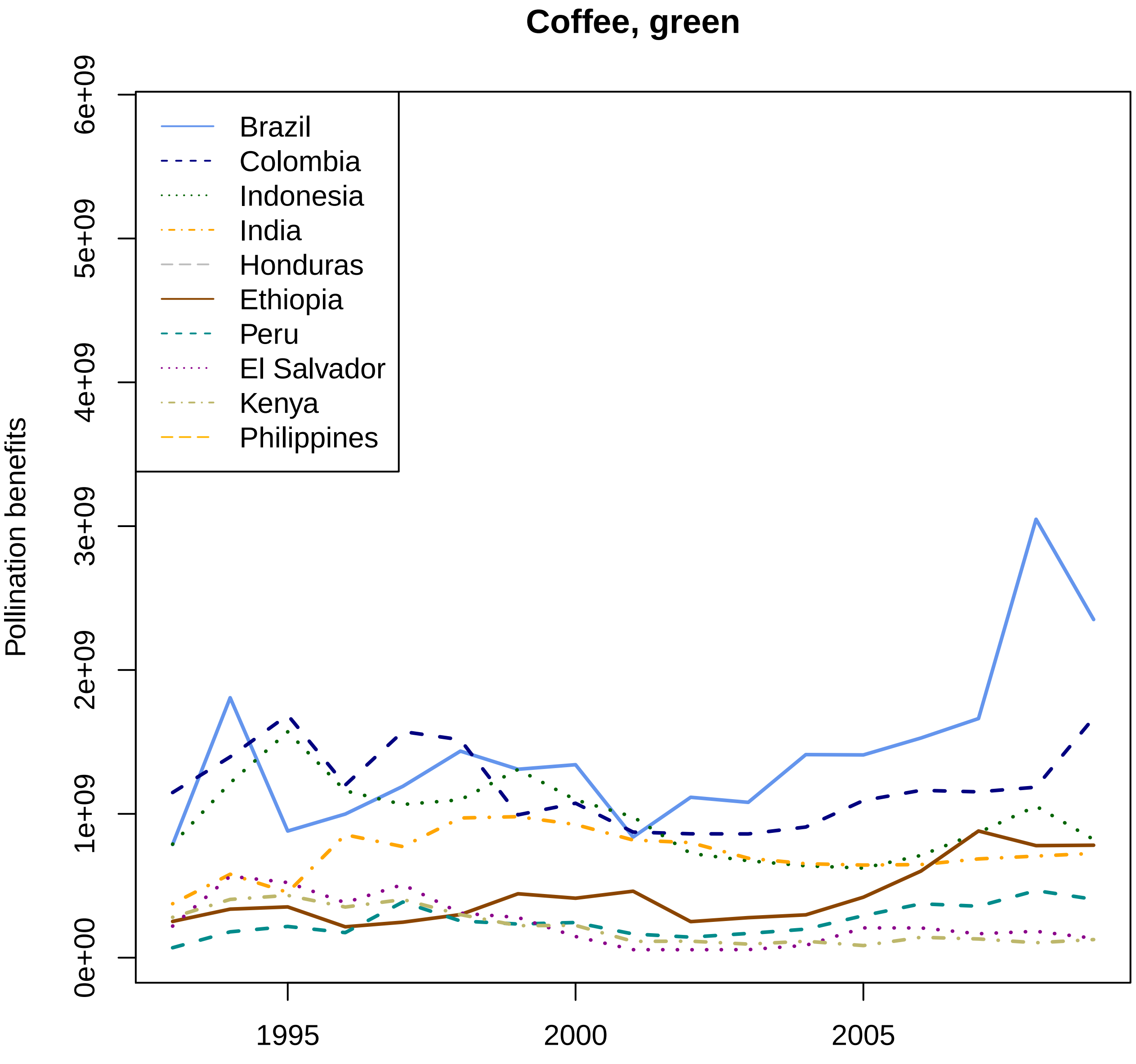
Coffee, green
Pollination benefits
0e+00
1e+09
2e+09
3e+09
4e+09
5e+09
6e+09
1995
2000
2005
Brazil
Colombia
Indonesia
India
Honduras
Ethiopia
Peru
El Salvador
Kenya
Philippines

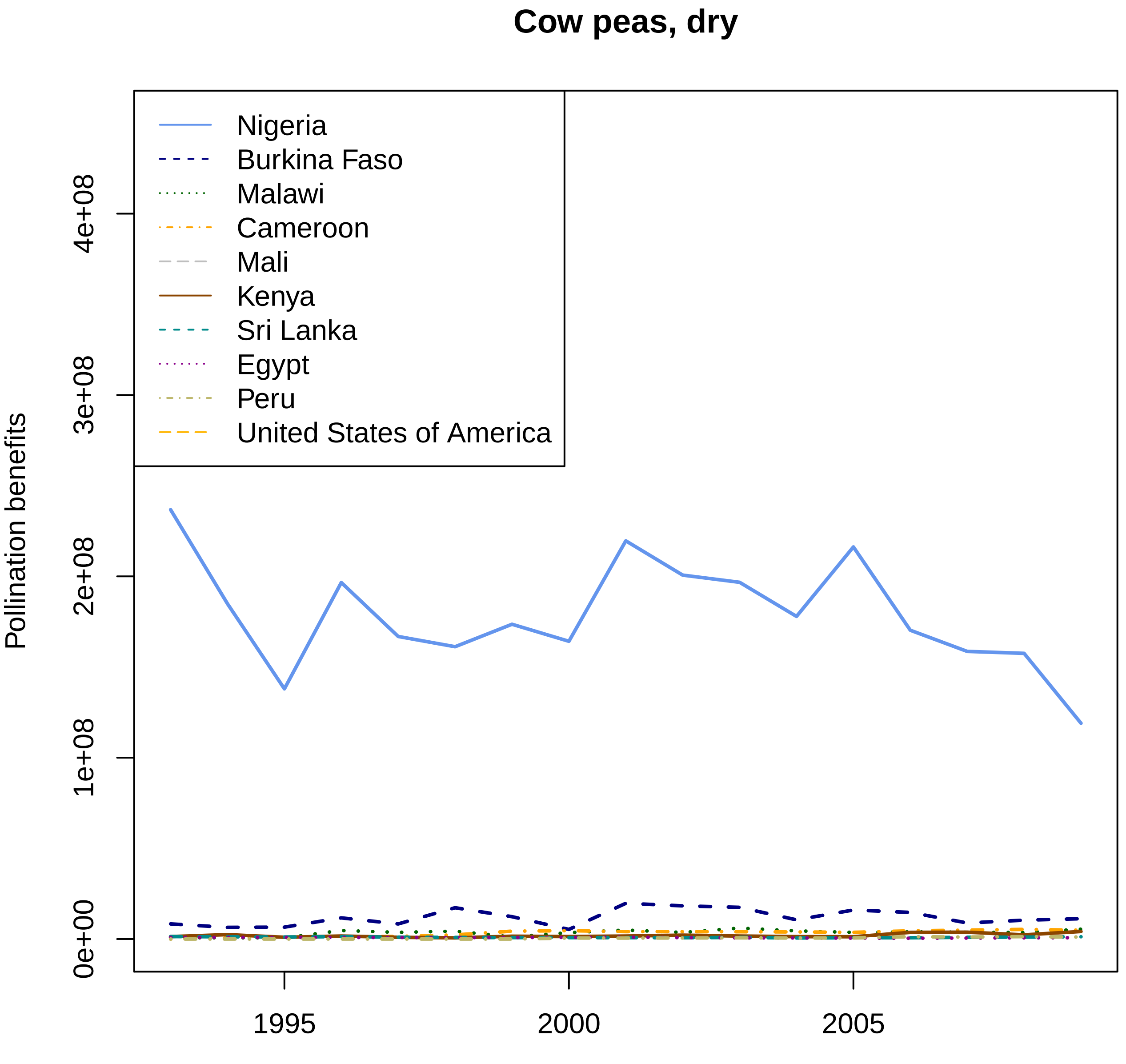

Cow peas, dry
Nigeria
Burkina Faso
Malawi
Cameroon
Mali
Kenya
Sri Lanka
Egypt
Peru
United States of America
Pollination benefits
0e+00
1e+08
2e+08
3e+08
4e+08
1995
2000
2005

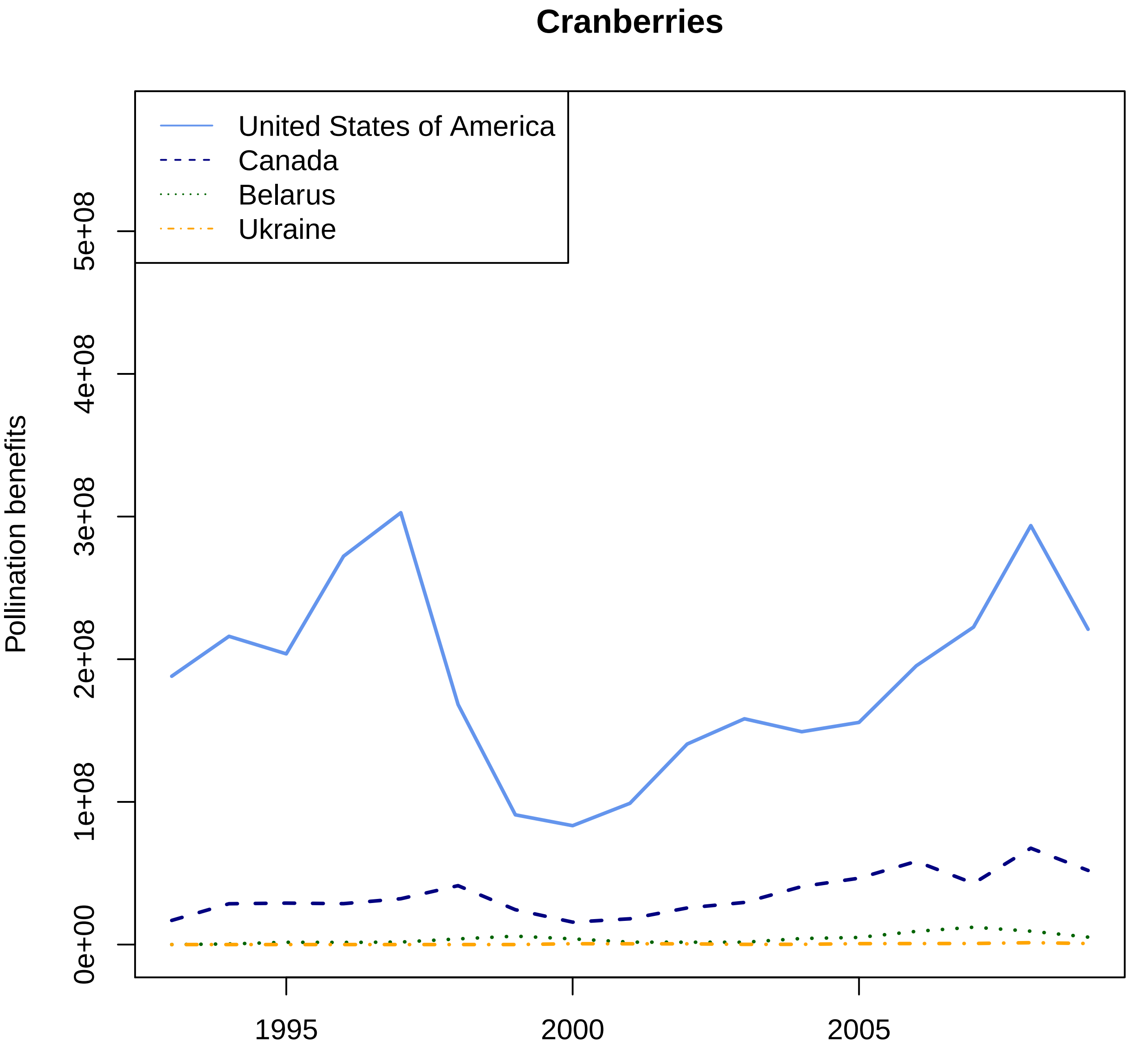
Cranberries
United States of America
Canada
Belarus
Ukraine
Pollination benefits
0e+00
1e+08
2e+08
3e+08
4e+08
5e+08
1995
2000
2005

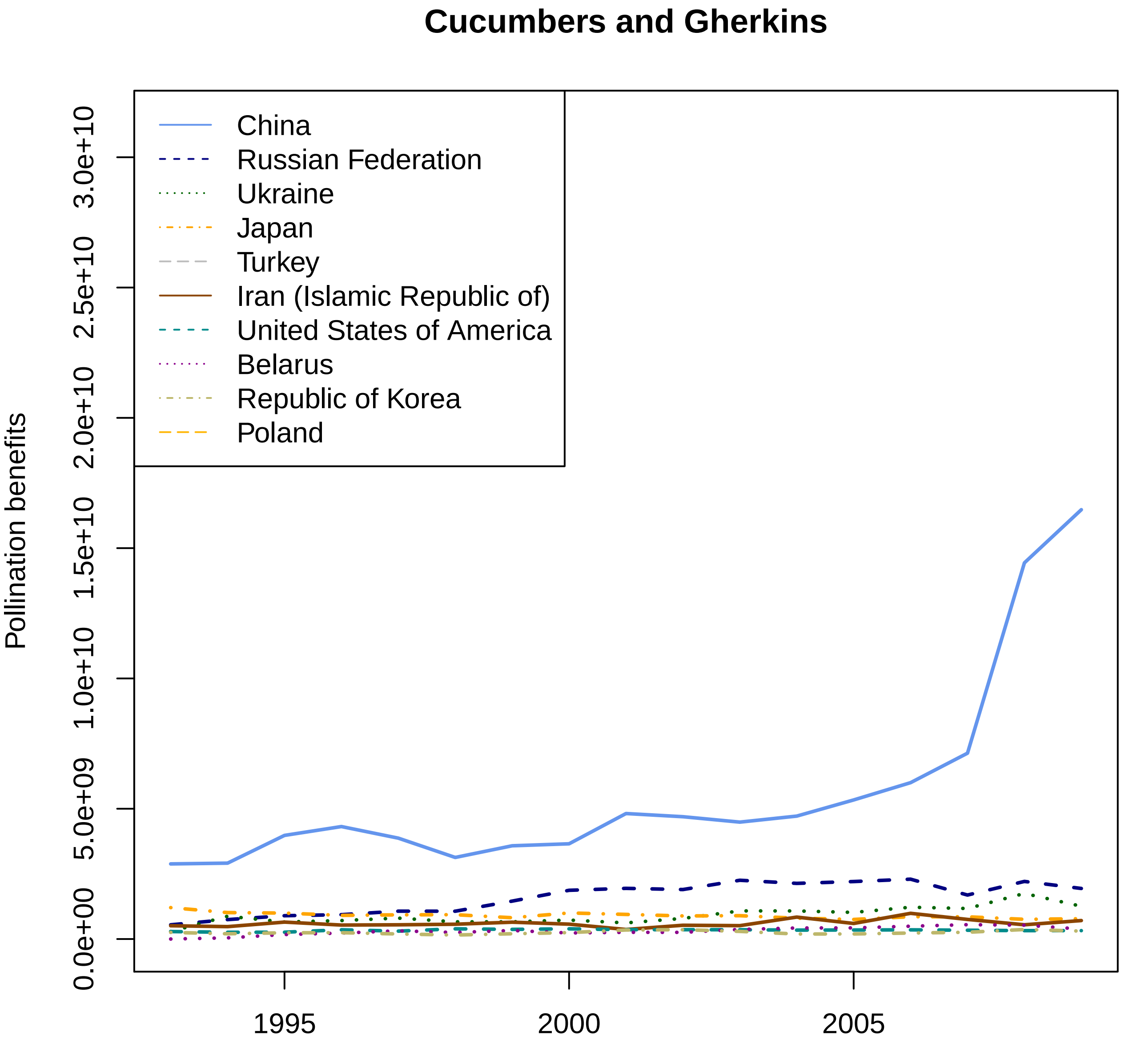

Cucumbers and Gherkins
China
Russian Federation
Ukraine
Japan
Turkey
Iran (Islamic Republic of)
United States of America
Belarus
Republic of Korea
Poland
Pollination benefits
0.0e+00
5.0e+09
1.0e+10
1.5e+10
2.0e+10
2.5e+10
3.0e+10
1995
2000
2005

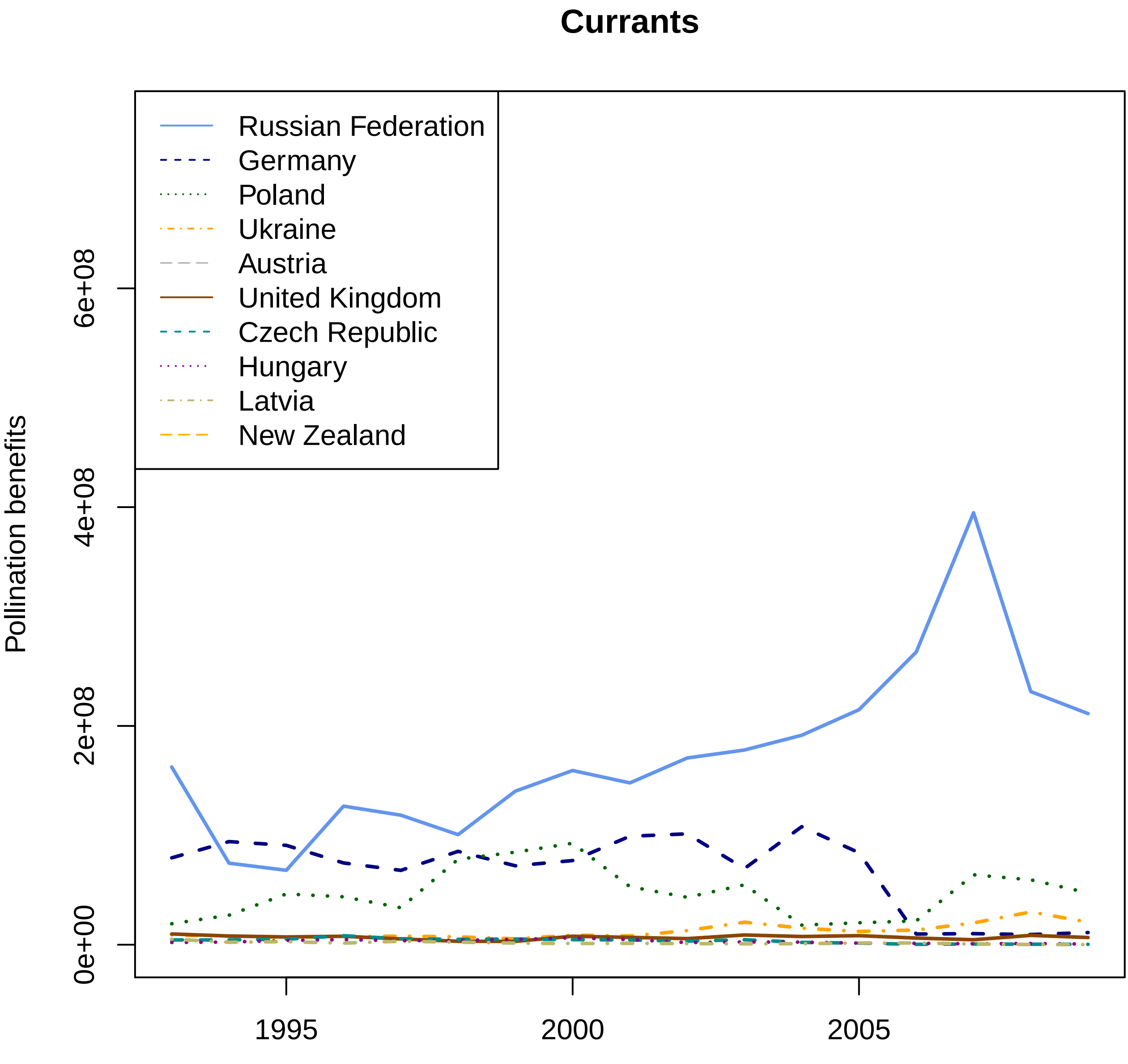
Currants
Russian Federation
Germany
Poland
Ukraine
Austria
United Kingdom
Czech Republic
Hungary
Latvia
New Zealand
Pollination benefits
0e+00
2e+08
4e+08
6e+08
1995
2000
2005

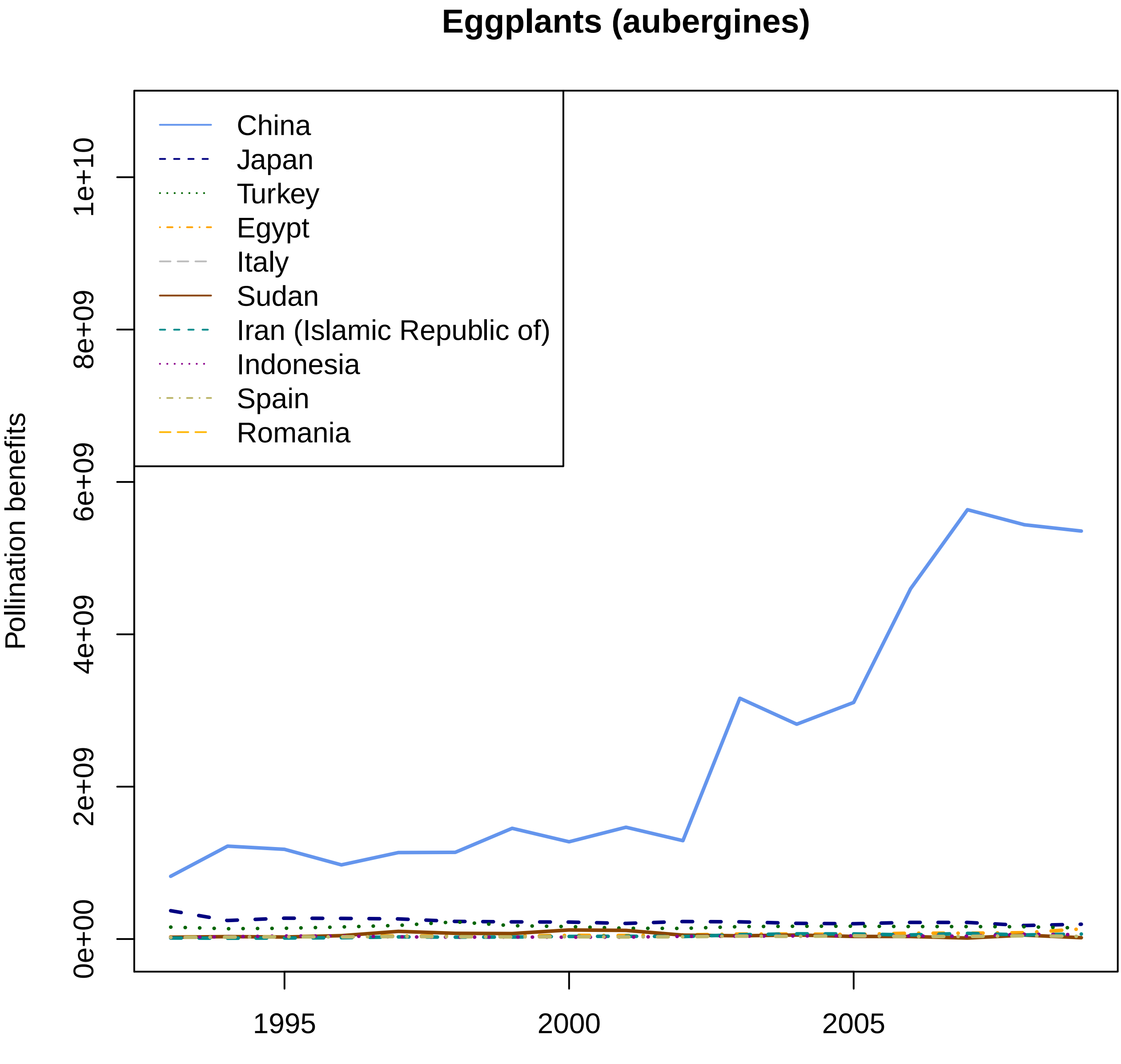

Eggplants (aubergines)
China
Japan
Turkey
Egypt
Italy
Sudan
Iran (Islamic Republic of)
Indonesia
Spain
Romania
Pollination benefits
0e+00
2e+09
4e+09
6e+09
8e+09
1e+10
1995
2000
2005

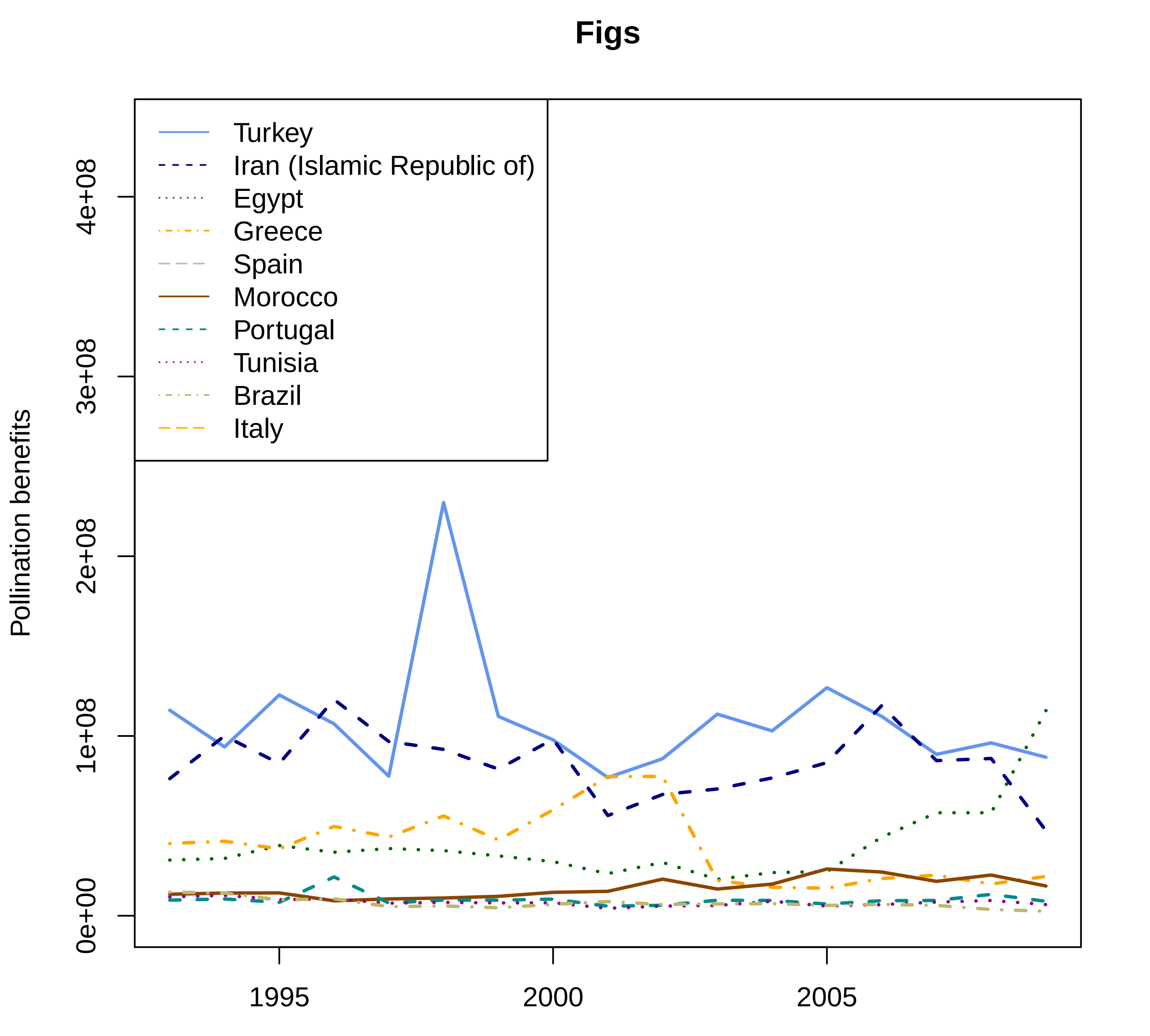

Figs
Turkey
Iran (Islamic Republic of)
Egypt
Greece
Spain
Morocco
Portugal
Tunisia
Brazil
Italy
Pollination benefits
0e+00
1e+08
2e+08
3e+08
4e+08
1995
2000
2005

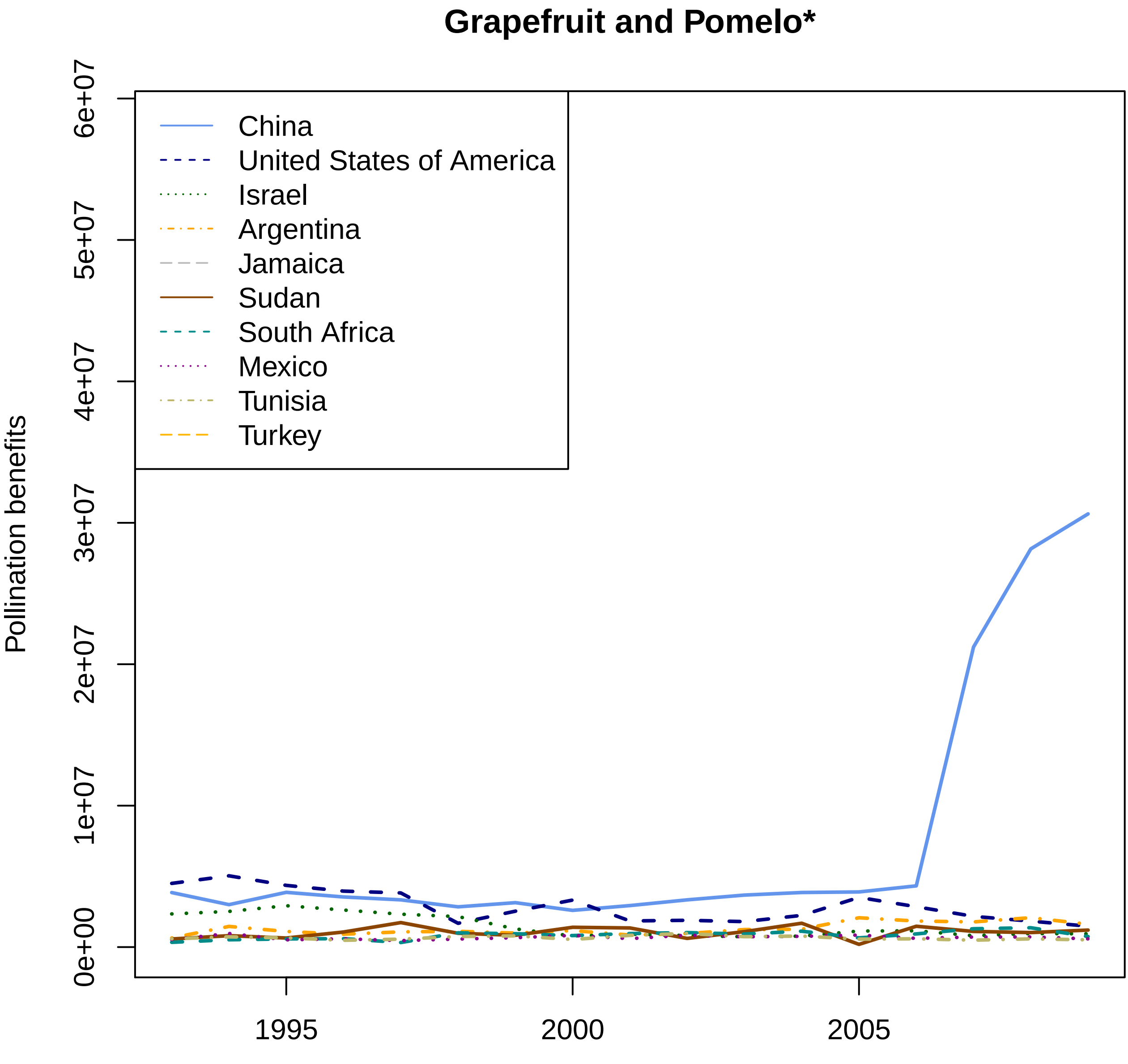

Grapefruit and Pomelo*
Pollination benefits
0e+00
1e+07
2e+07
3e+07
4e+07
5e+07
6e+07
1995
2000
2005
China
United States of America
Israel
Argentina
Jamaica
Sudan
South Africa
Mexico
Tunisia
Turkey

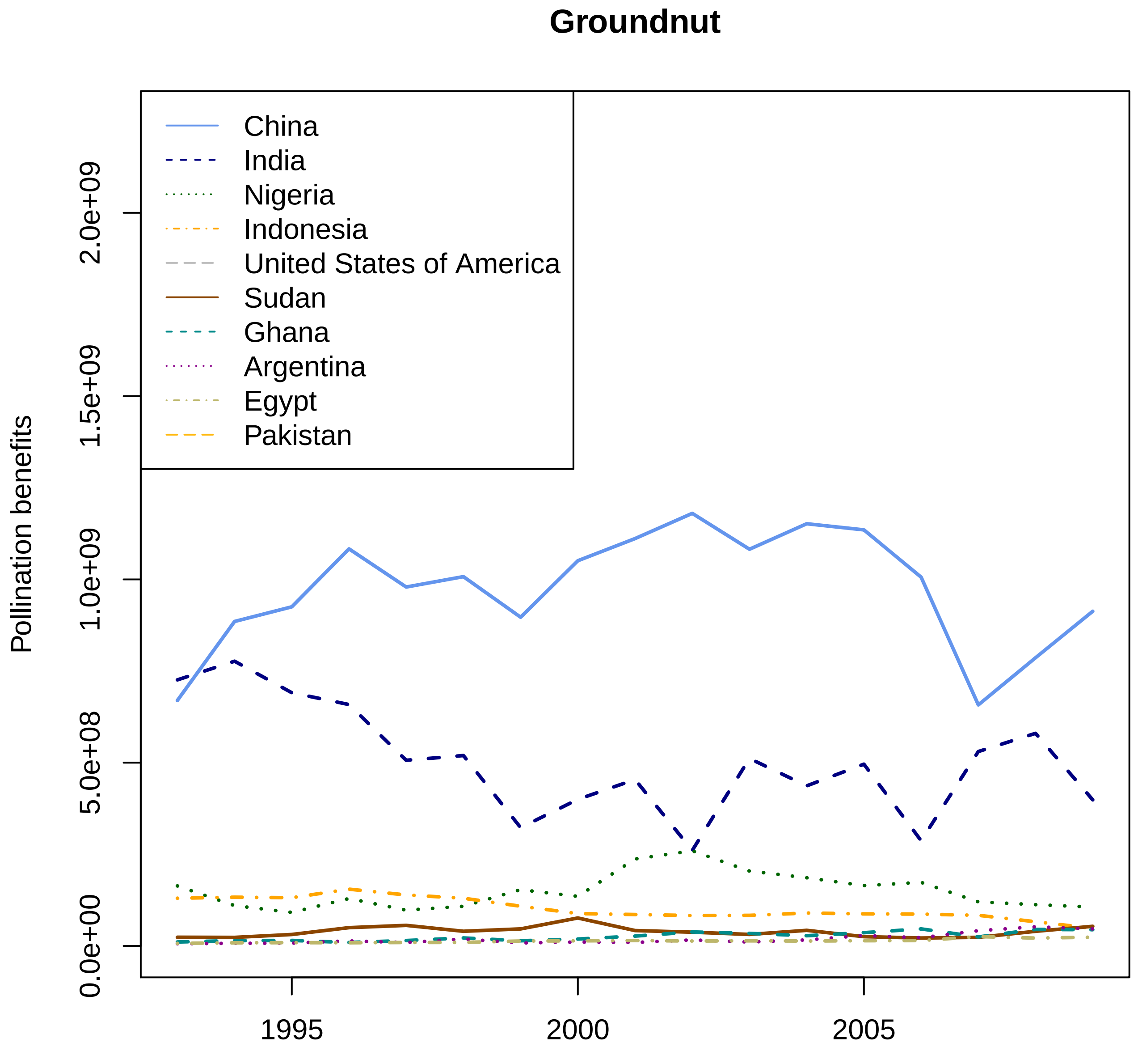
Groundnut
Pollination benefits
0.0e+00
5.0e+08
1.0e+09
1.5e+09
2.0e+09
1995
2000
2005
China
India
Nigeria
Indonesia
United States of America
Sudan
Ghana
Argentina
Egypt
Pakistan

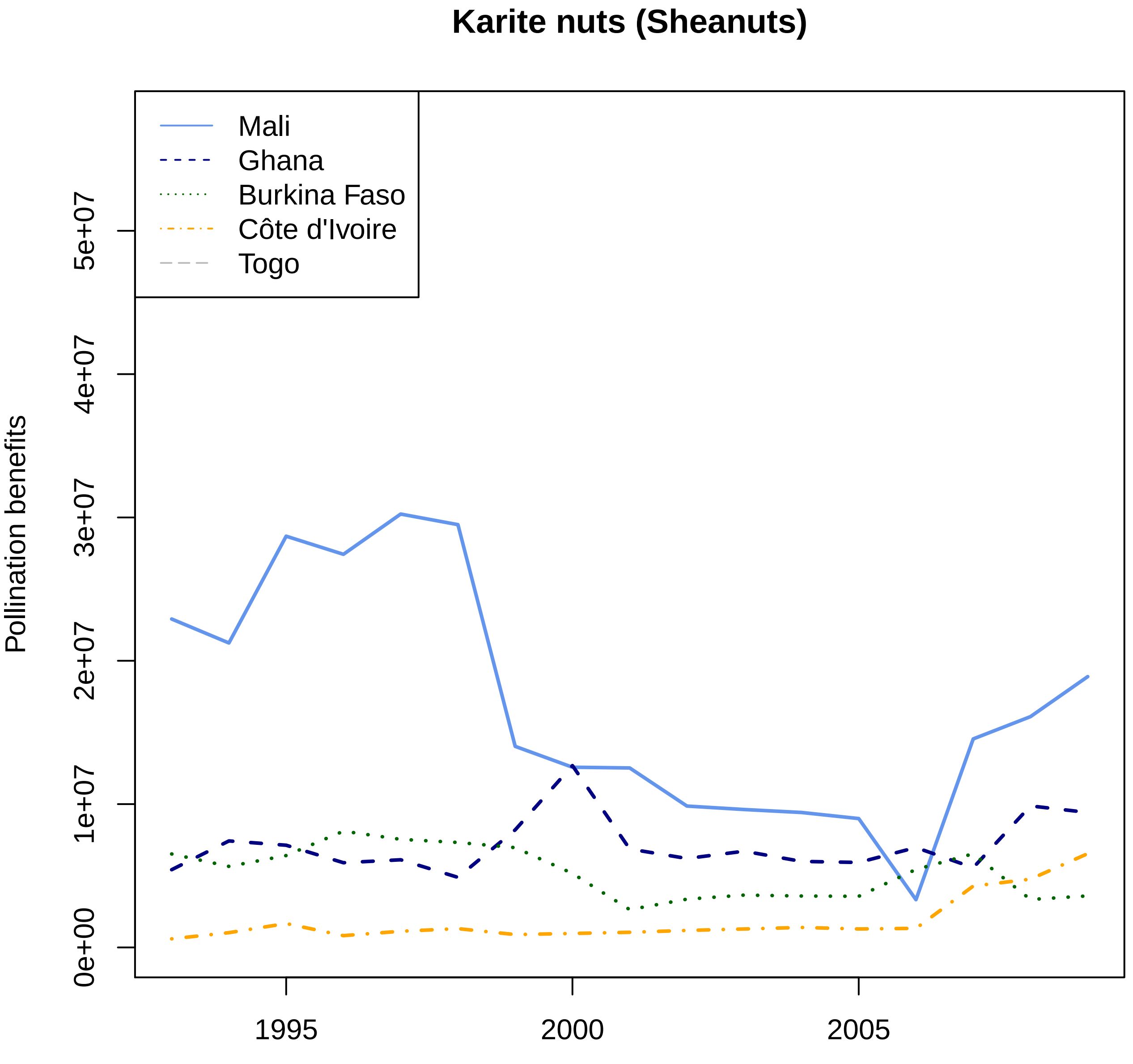
Karite nuts (Sheanuts)
Mali
Ghana
Burkina Faso
Côte d'Ivoire
Togo
Pollination benefits
0e+00
1e+07
2e+07
3e+07
4e+07
5e+07
1995
2000
2005

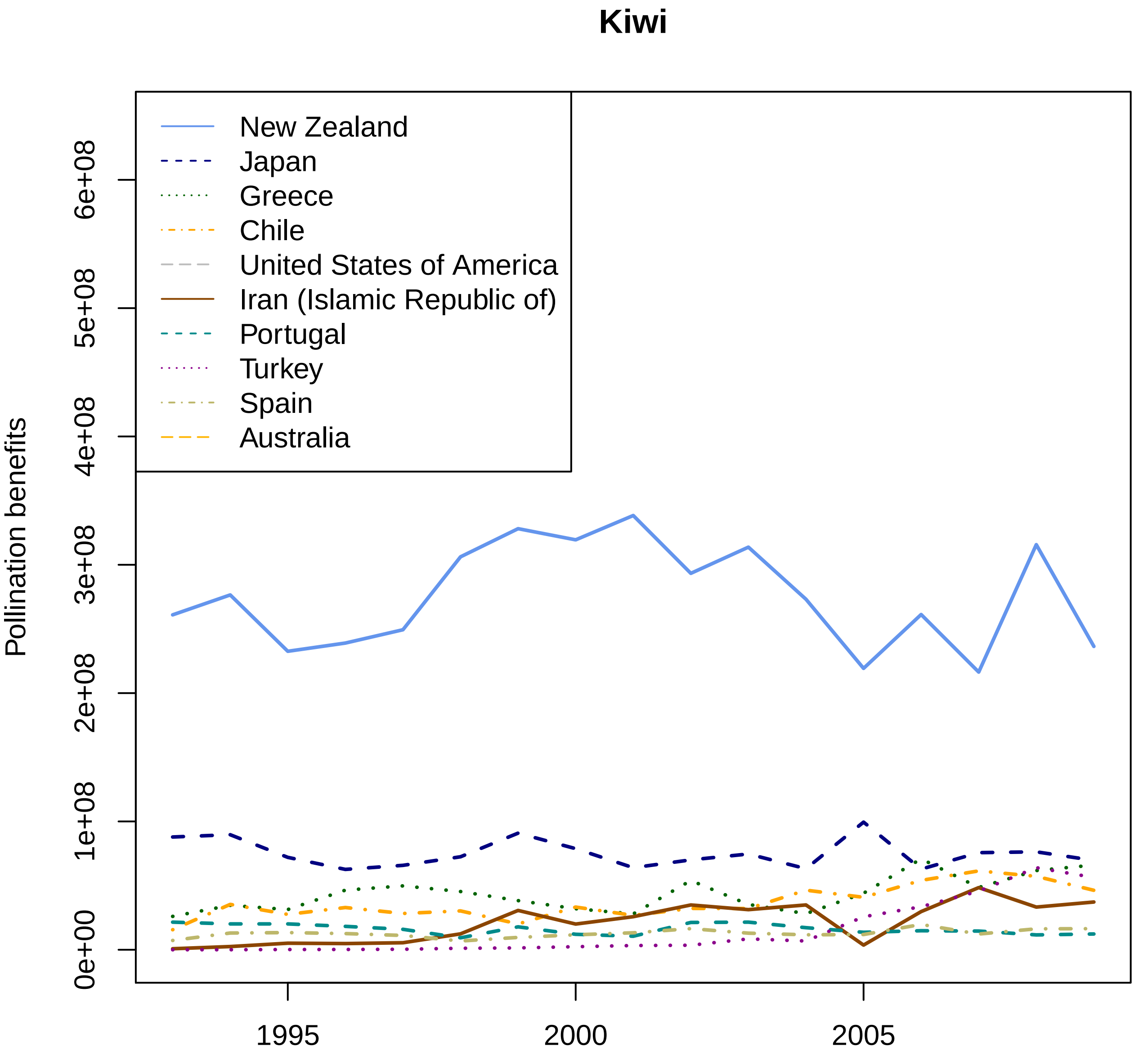

Kiwi
New Zealand
Japan
Greece
Chile
United States of America
Iran (Islamic Republic of)
Portugal
Turkey
Spain
Australia
Pollination benefits
0e+00
1e+08
2e+08
3e+08
4e+08
5e+08
6e+08
1995
2000
2005

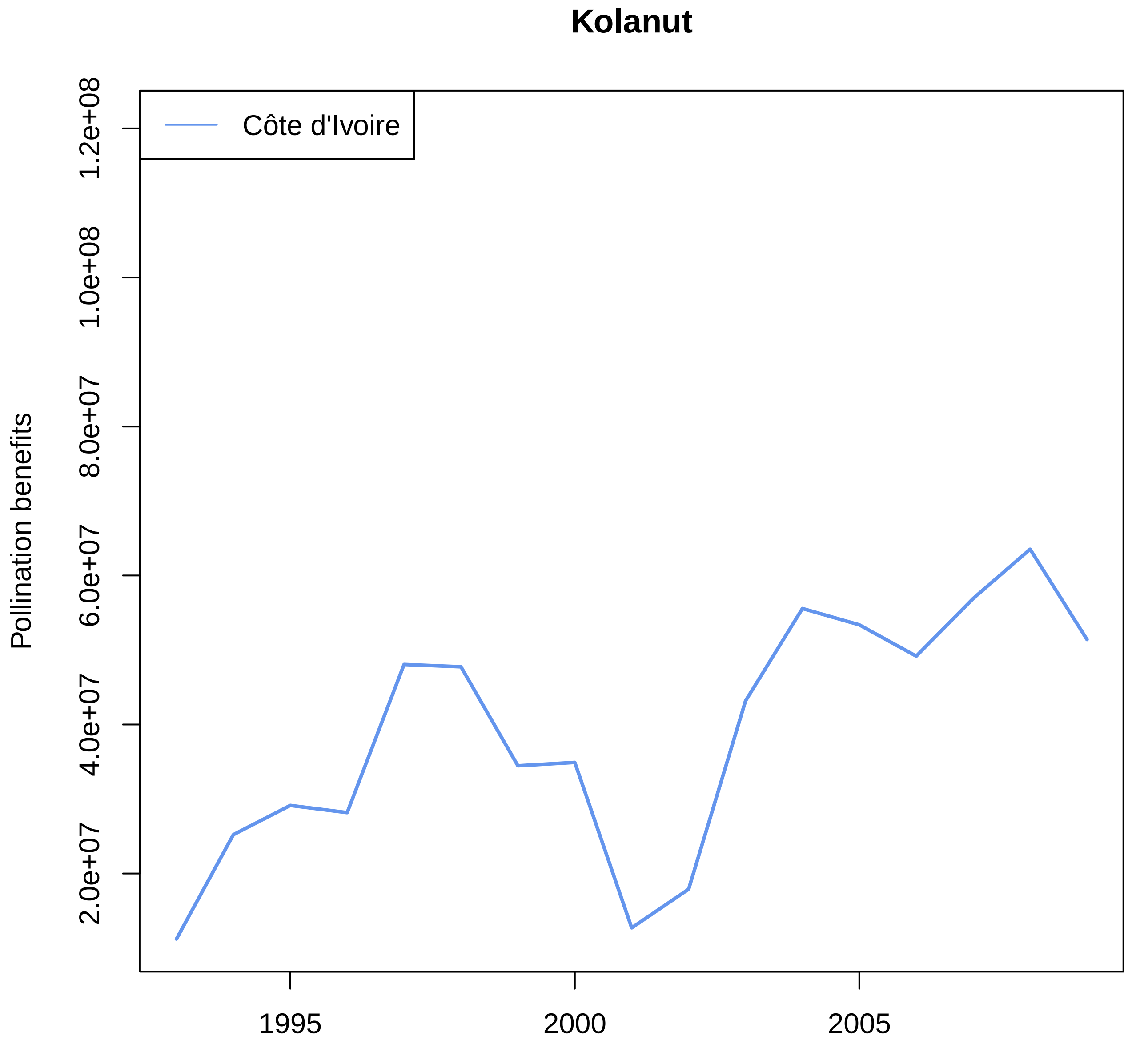
Kolanut
Côte d'Ivoire
Pollination benefits
2.0e+07
4.0e+07
6.0e+07
8.0e+07
1.0e+08
1.2e+08
1995
2000
2005

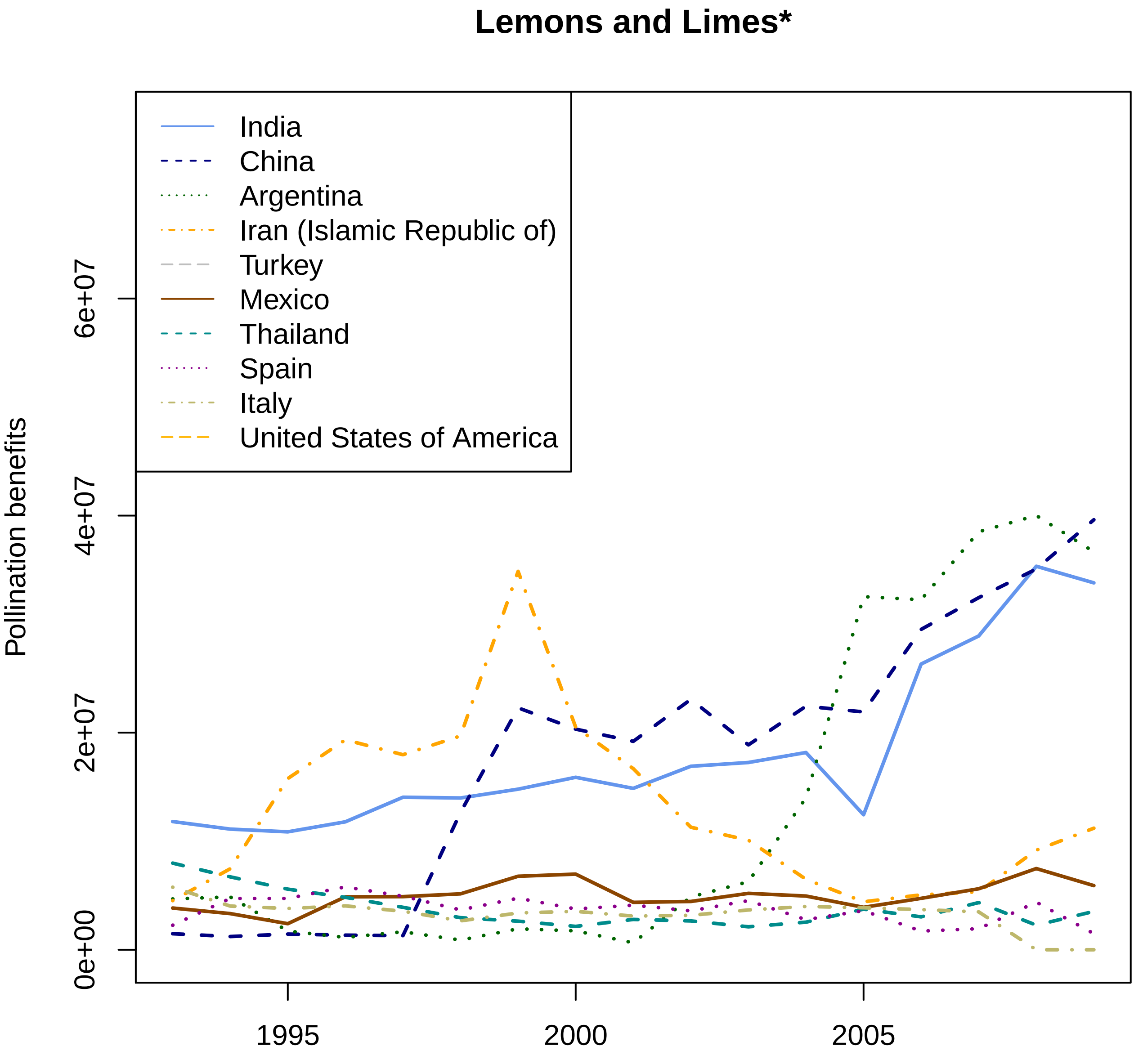
Lemons and Limes*
India
China
Argentina
Iran (Islamic Republic of)
Turkey
Mexico
Thailand
Spain
Italy
United States of America
Pollination benefits
0e+00
2e+07
4e+07
6e+07
1995
2000
2005

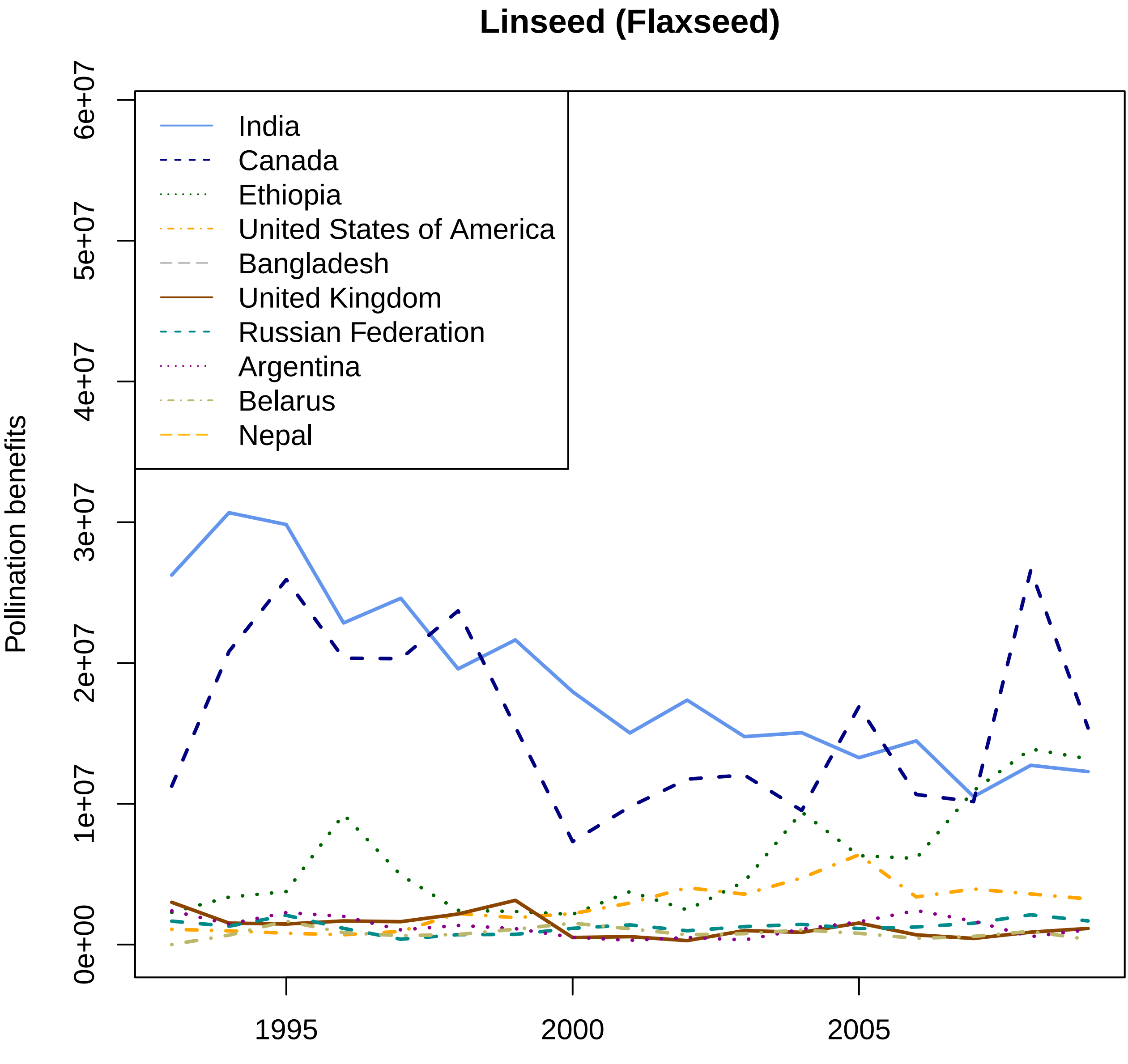

Linseed (Flaxseed)
Pollination benefits
0e+00
1e+07
2e+07
3e+07
4e+07
5e+07
6e+07
1995
2000
2005
India
Canada
Ethiopia
United States of America
Bangladesh
United Kingdom
Russian Federation
Argentina
Belarus
Nepal

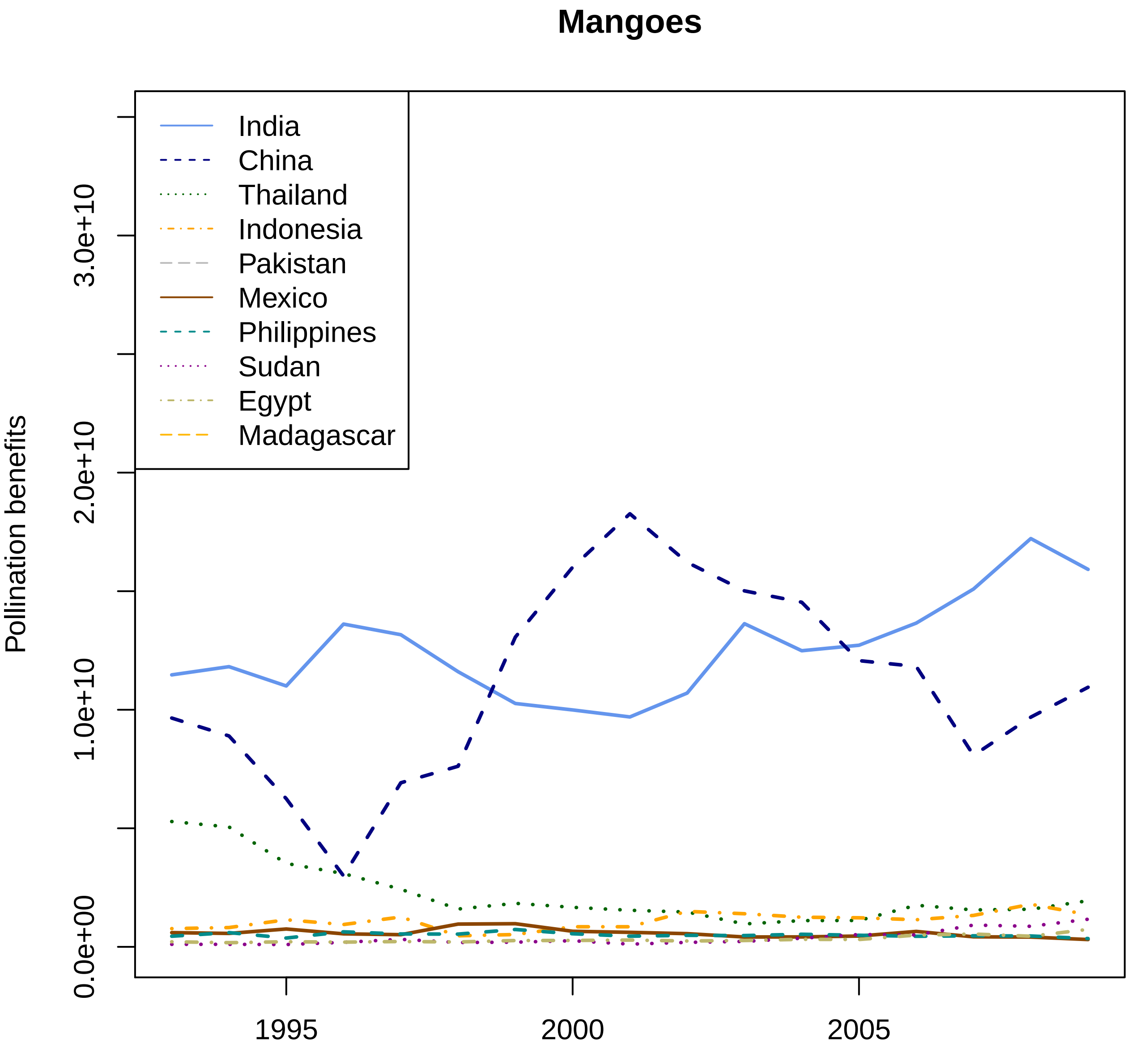
Mangoes
Pollination benefits
0.0e+00
1.0e+10
2.0e+10
3.0e+10
1995
2000
2005
India
China
Thailand
Indonesia
Pakistan
Mexico
Philippines
Sudan
Egypt
Madagascar

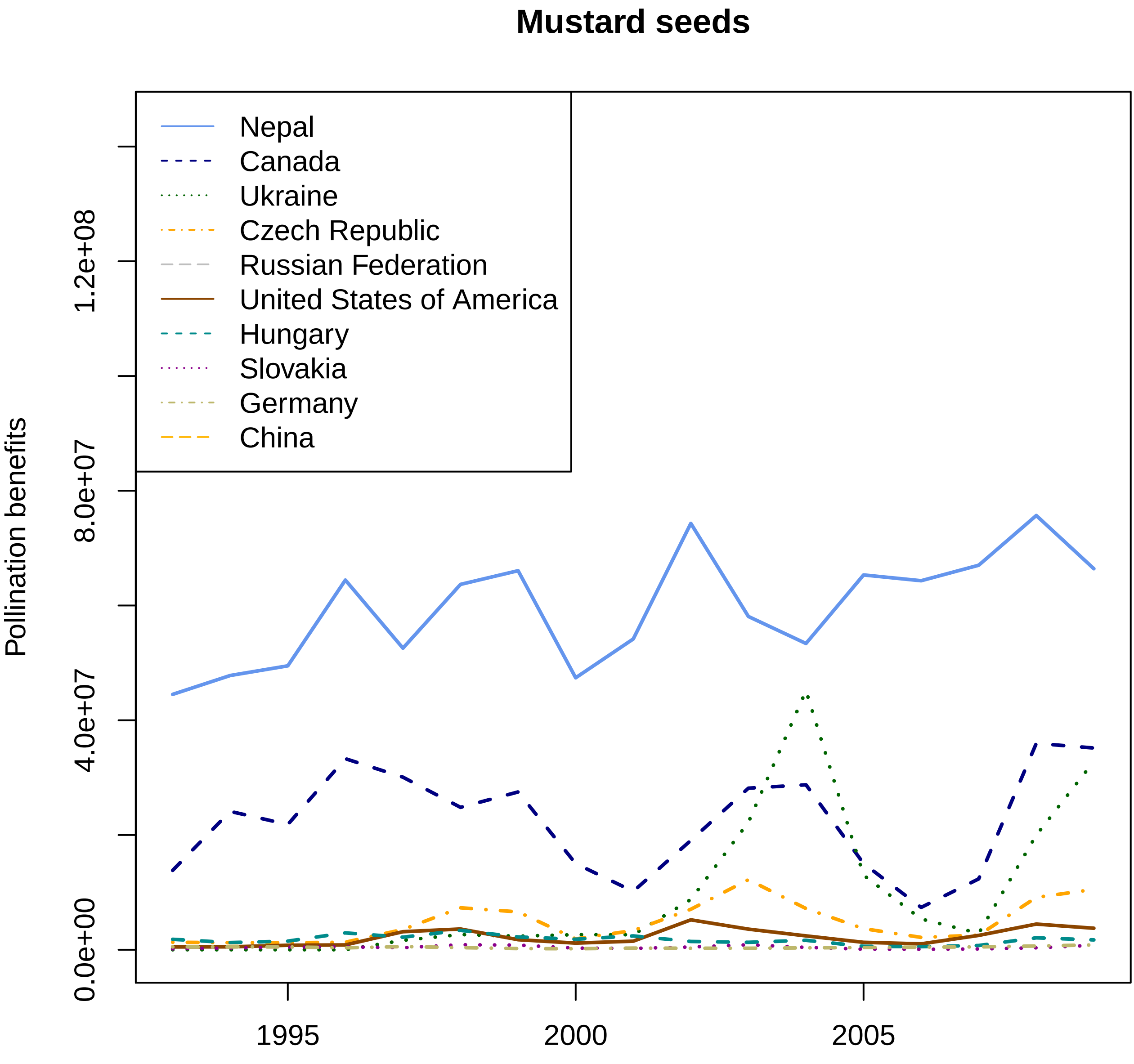

Mustard seeds
Nepal
Canada
Ukraine
Czech Republic
Russian Federation
United States of America
Hungary
Slovakia
Germany
China
Pollination benefits
0.0e+00
4.0e+07
8.0e+07
1.2e+08
1995
2000
2005

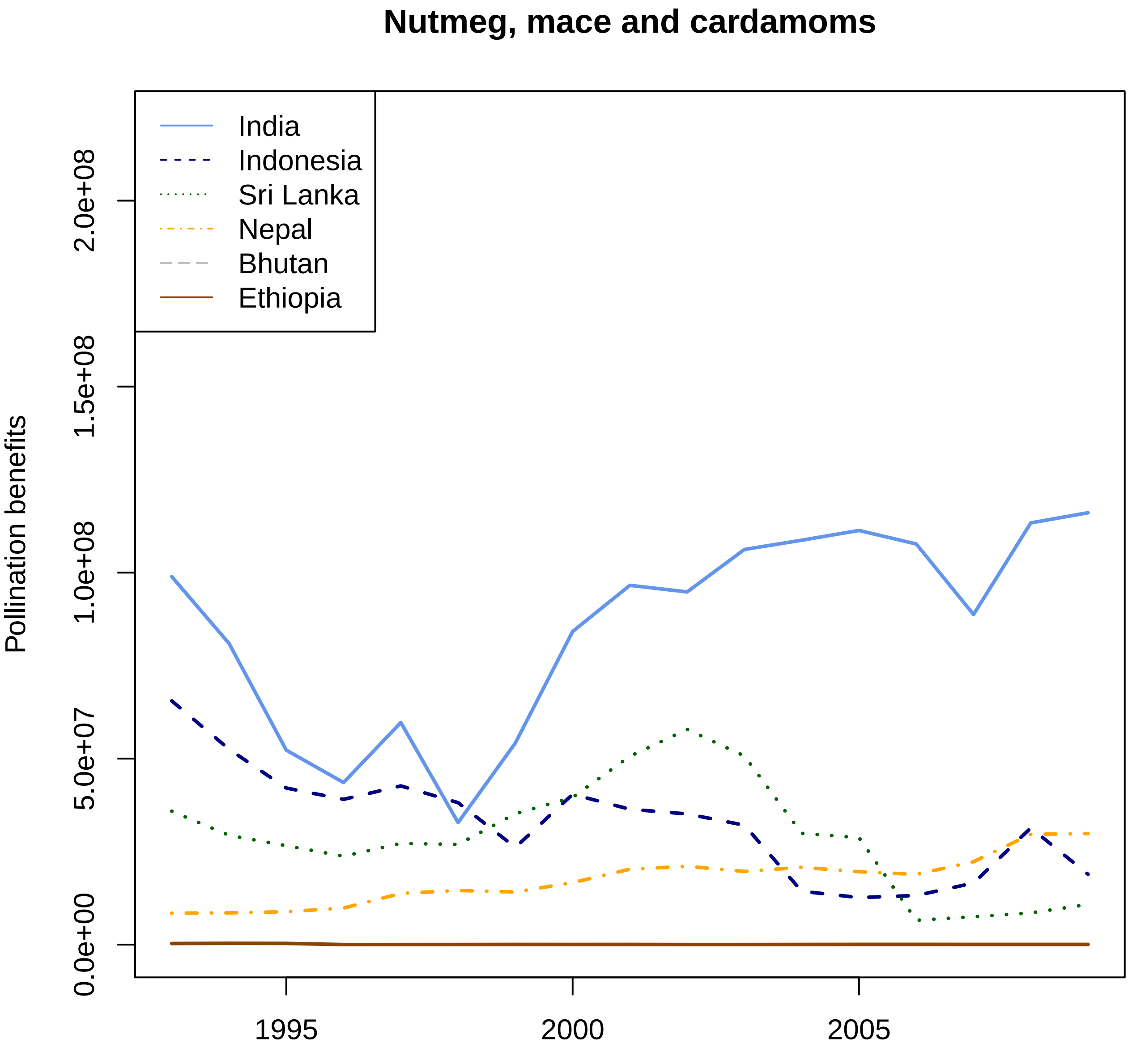

Nutmeg, mace and cardamoms
Pollination benefits
0.0e+00
5.0e+07
1.0e+08
1.5e+08
2.0e+08
1995
2000
2005
India
Indonesia
Sri Lanka
Nepal
Bhutan
Ethiopia

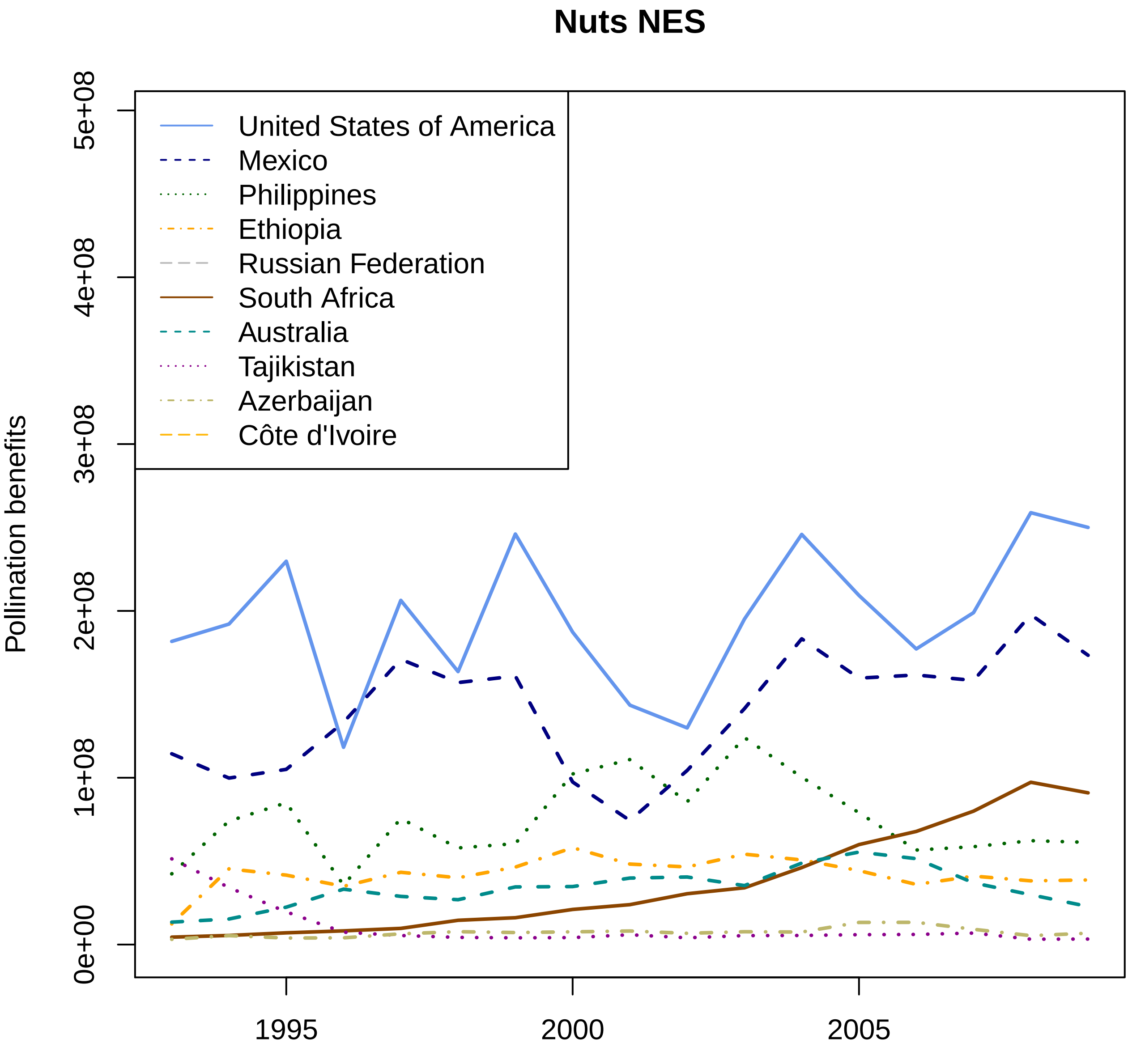

Nuts NES
Pollination benefits
0e+00
1e+08
2e+08
3e+08
4e+08
5e+08
1995
2000
2005
United States of America
Mexico
Philippines
Ethiopia
Russian Federation
South Africa
Australia
Tajikistan
Azerbaijan
Côte d'Ivoire

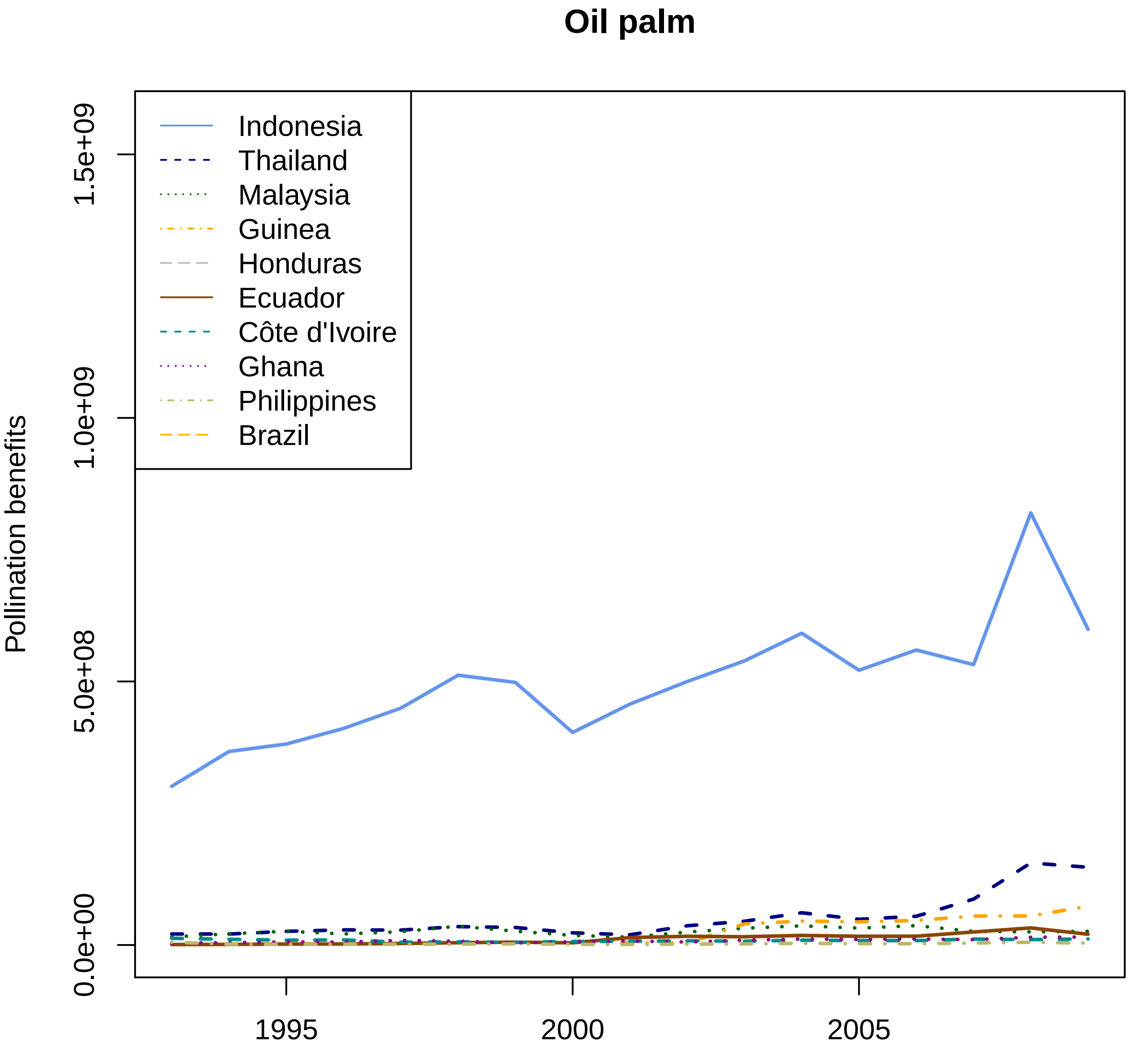

Oil palm
Indonesia
Thailand
Malaysia
Guinea
Honduras
Ecuador
Côte d'Ivoire
Ghana
Philippines
Brazil
Pollination benefits
0.0e+00
5.0e+08
1.0e+09
1.5e+09
1995
2000
2005

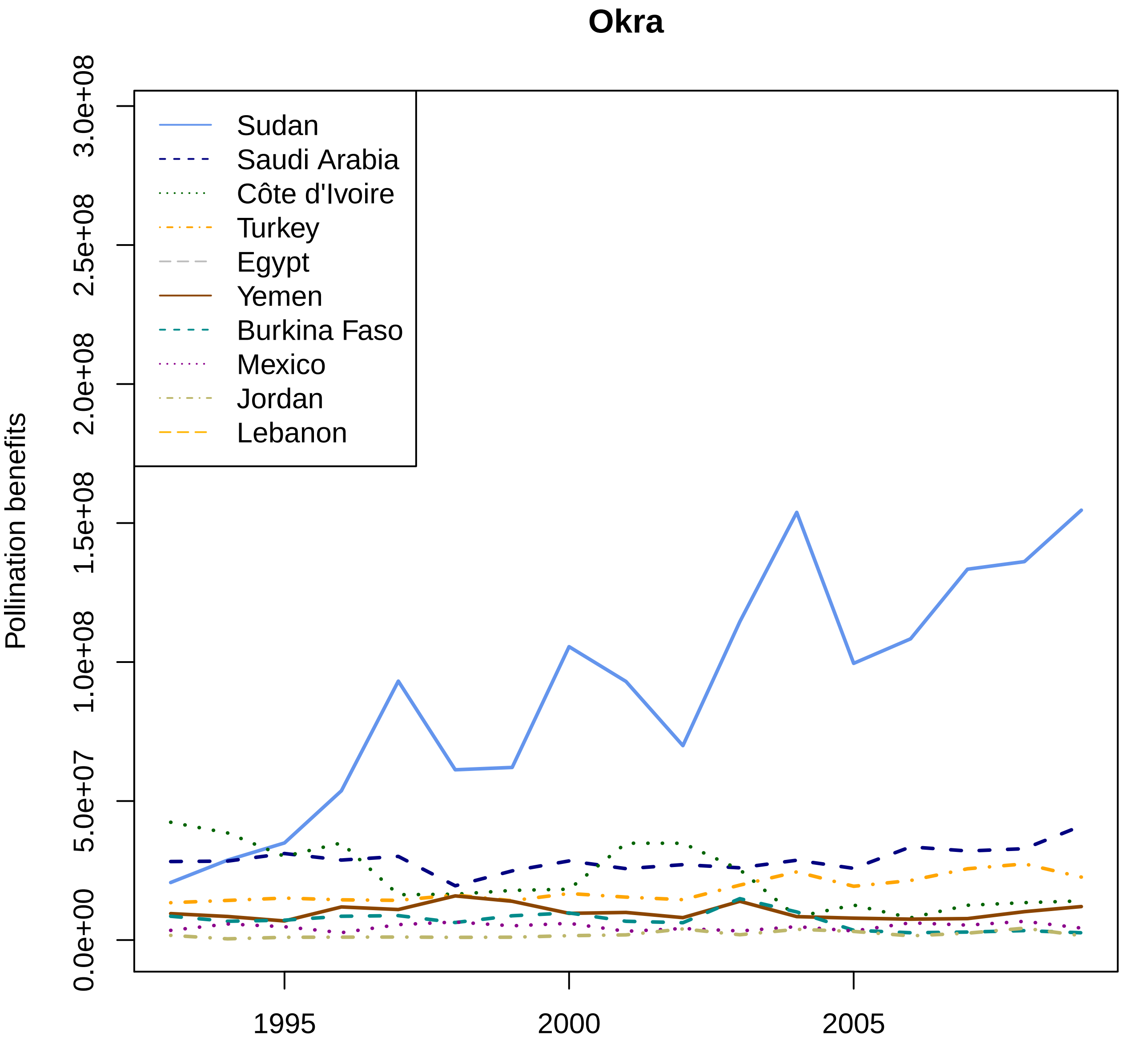

Okra
Pollination benefits
0.0e+00
5.0e+07
1.0e+08
1.5e+08
2.0e+08
2.5e+08
3.0e+08
1995
2000
2005
Sudan
Saudi Arabia
Côte d'Ivoire
Turkey
Egypt
Yemen
Burkina Faso
Mexico
Jordan
Lebanon

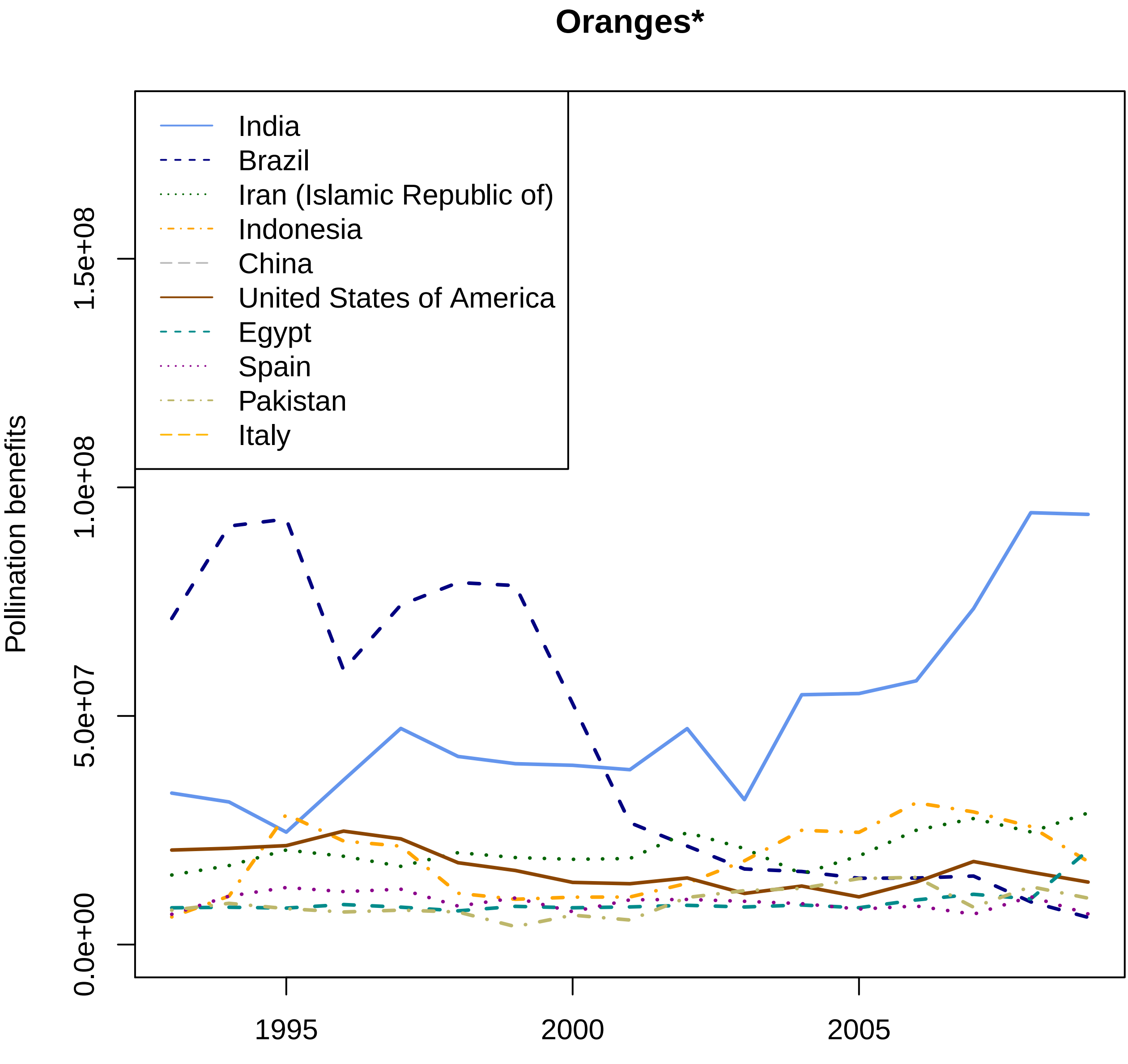

Oranges*
India
Brazil
Iran (Islamic Republic of)
Indonesia
China
United States of America
Egypt
Spain
Pakistan
Italy
Pollination benefits
0.0e+00
5.0e+07
1.0e+08
1.5e+08
1995
2000
2005

Other melons (inc.cantaloupes)

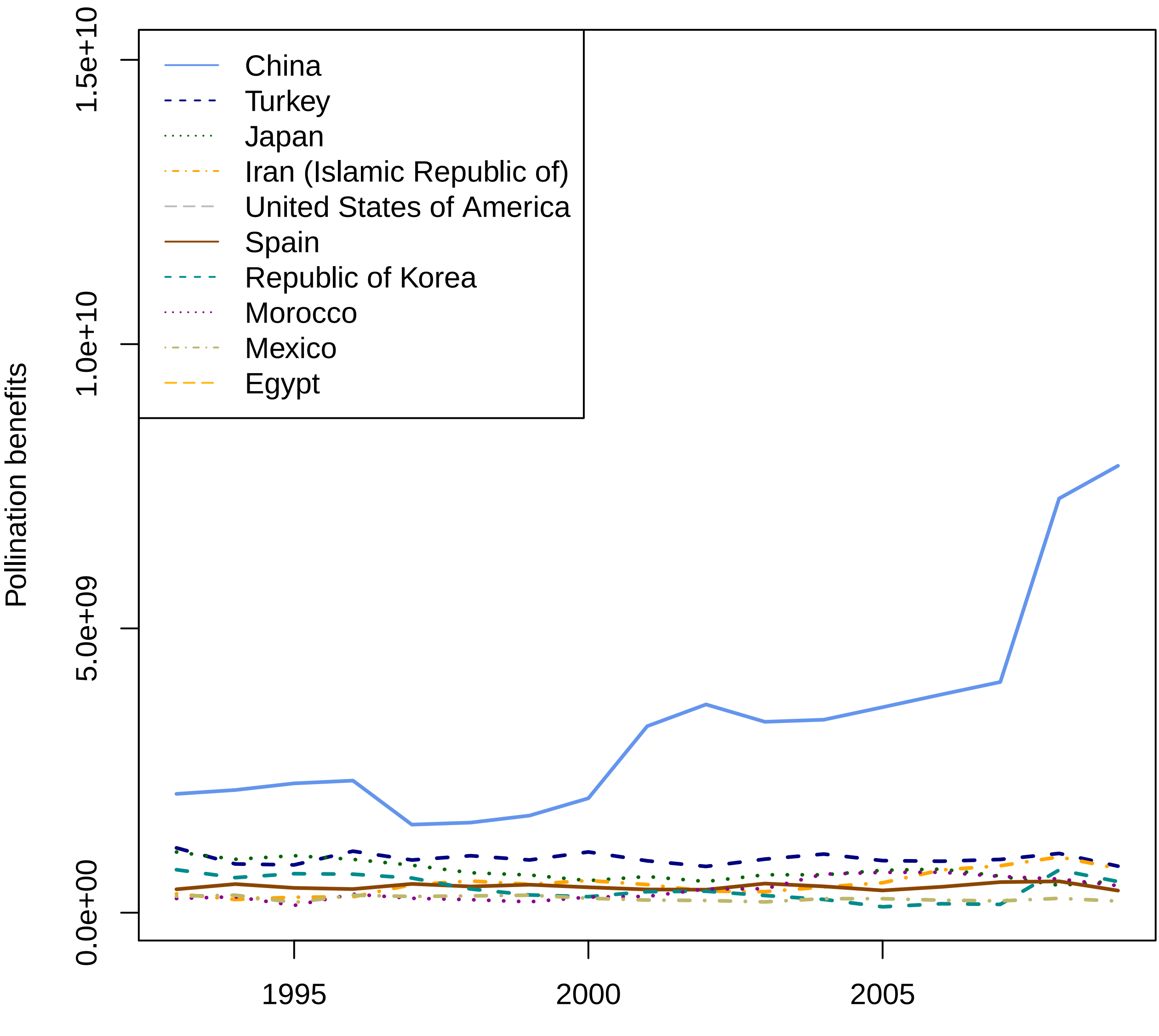

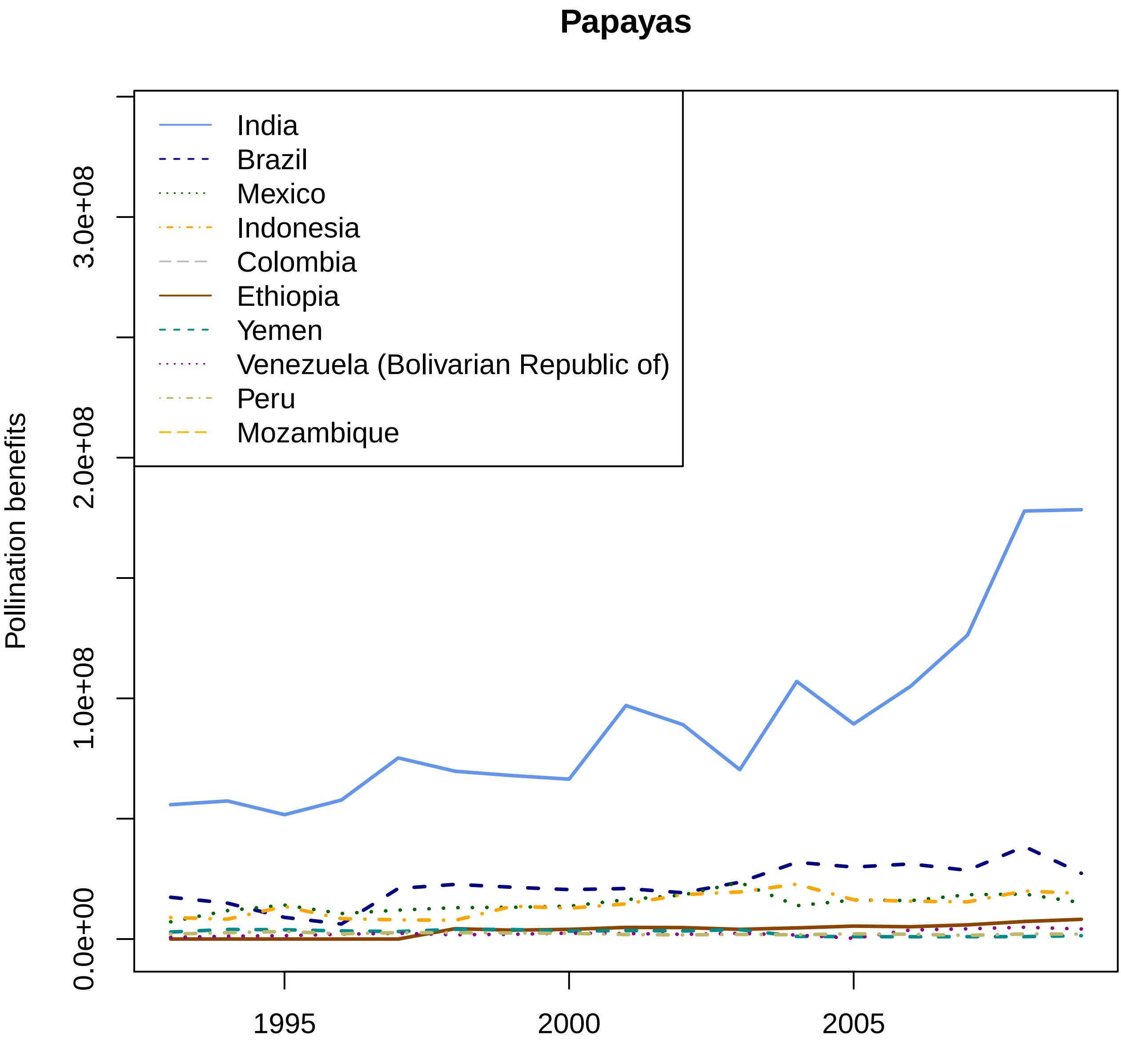
Papayas
India
Brazil
Mexico
Indonesia
Colombia
Ethiopia
Yemen
Venezuela (Bolivarian Republic of)
Peru
Mozambique
Pollination benefits
0.0e+00
1.0e+08
2.0e+08
3.0e+08
1995
2000
2005

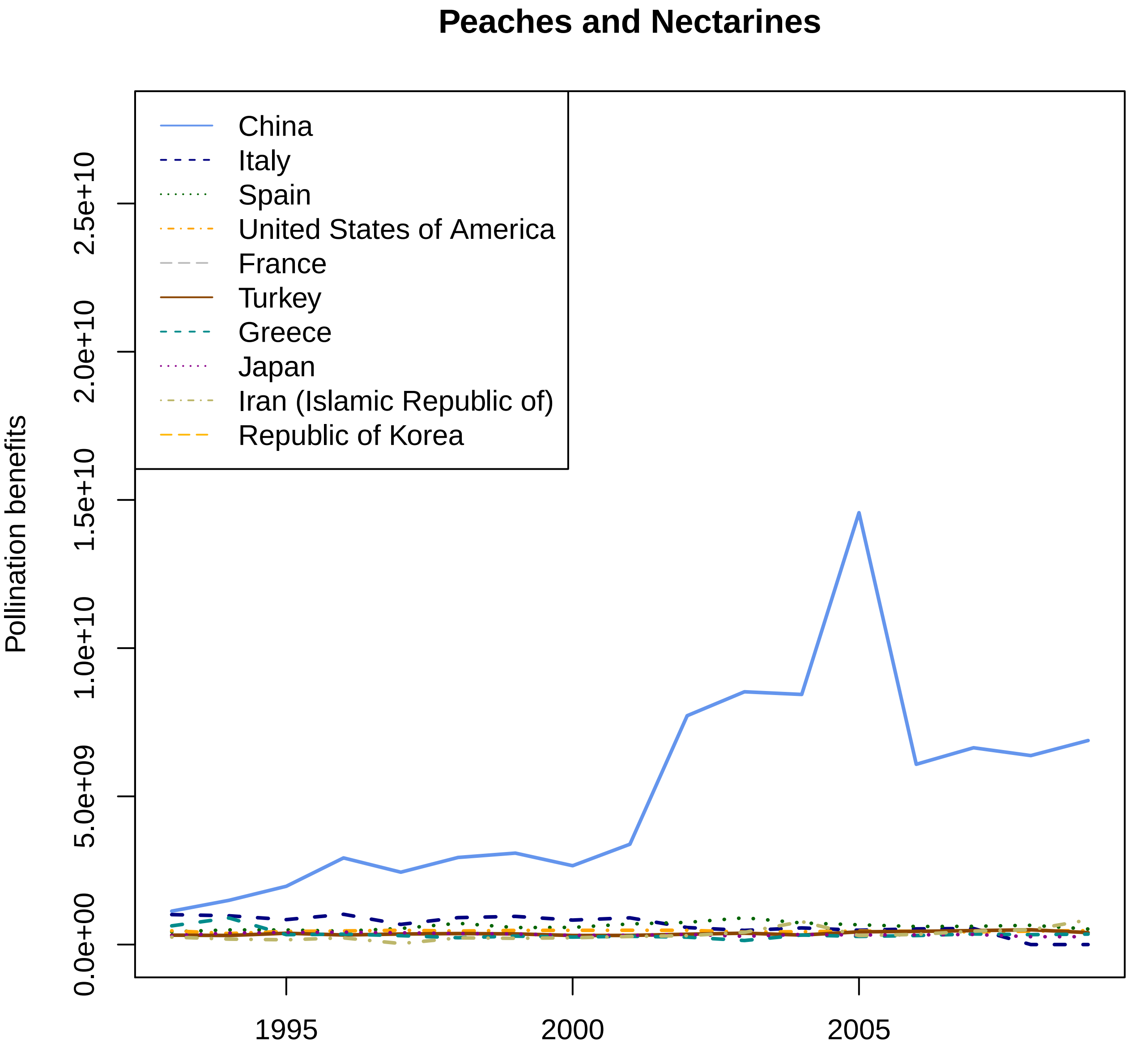

Peaches and Nectarines
Pollination benefits
0.0e+00
5.0e+09
1.0e+10
1.5e+10
2.0e+10
2.5e+10
1995
2000
2005
China
Italy
Spain
United States of America
France
Turkey
Greece
Japan
Iran (Islamic Republic of)
Republic of Korea

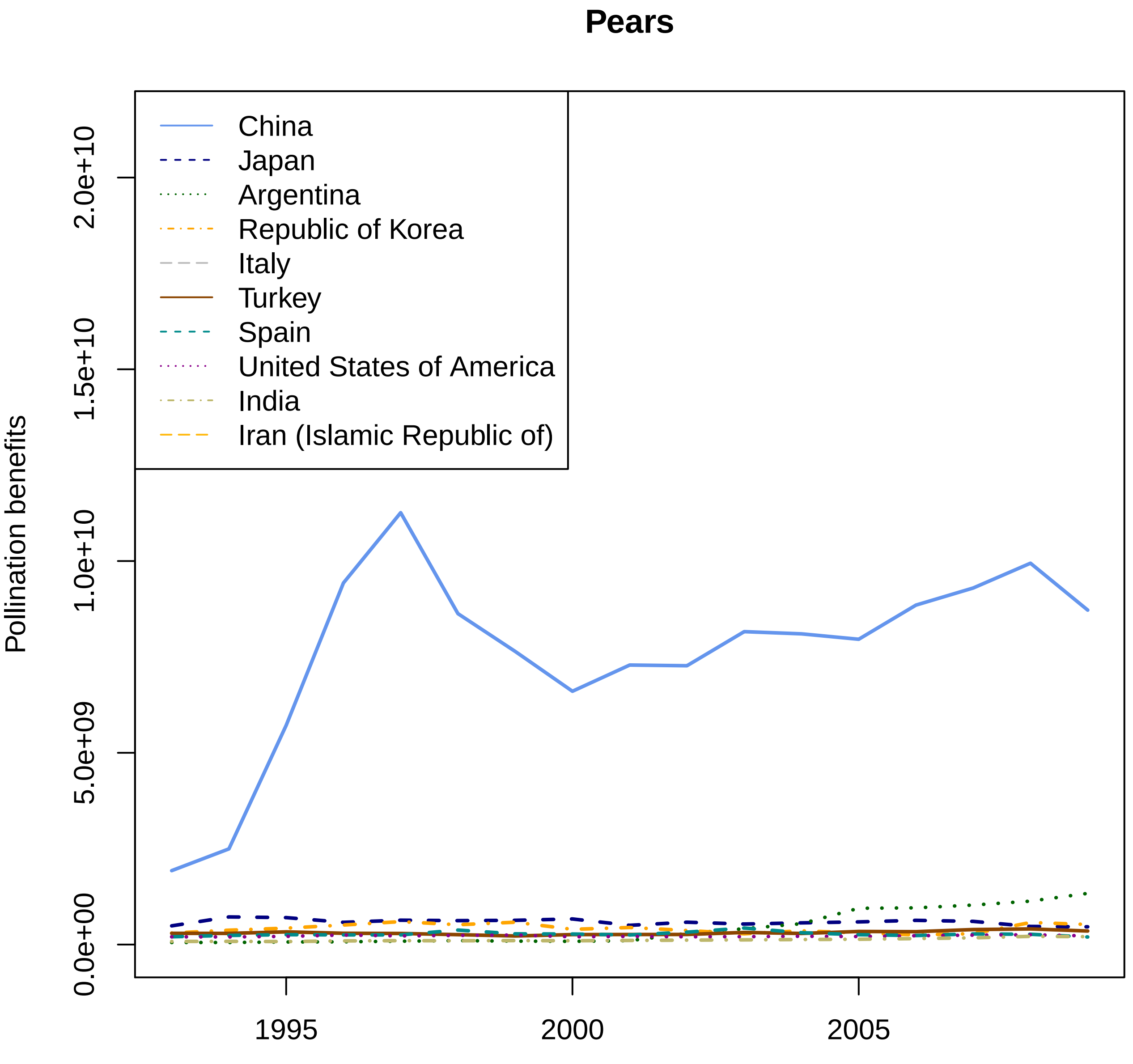
Pears
Pollination benefits
0.0e+00
5.0e+09
1.0e+10
1.5e+10
2.0e+10
1995
2000
2005
China
Japan
Argentina
Republic of Korea
Italy
Turkey
Spain
United States of America
India
Iran (Islamic Republic of)

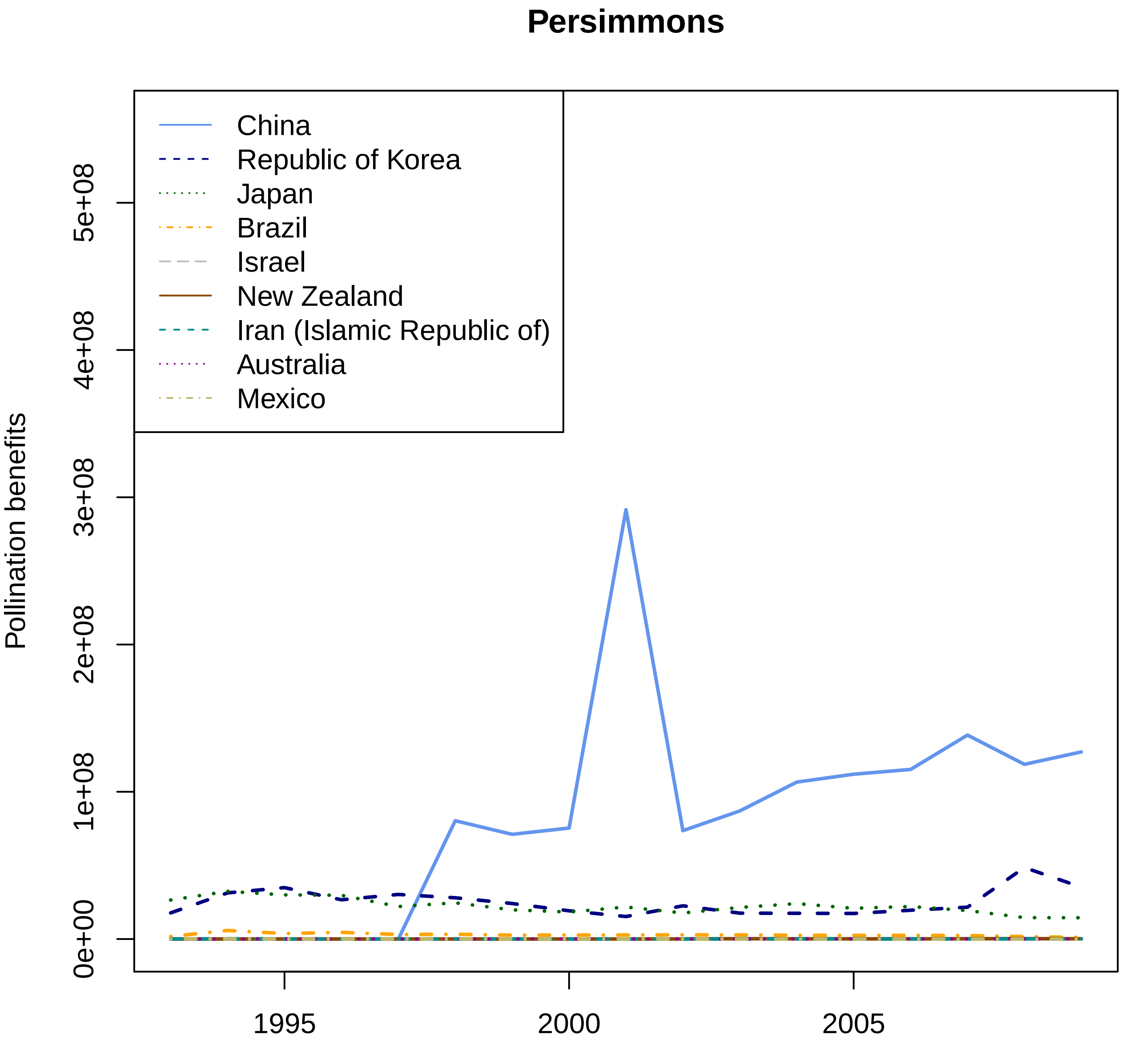

Persimmons
Pollination benefits
0e+00
1e+08
2e+08
3e+08
4e+08
5e+08
1995
2000
2005
China
Republic of Korea
Japan
Brazil
Israel
New Zealand
Iran (Islamic Republic of)
Australia
Mexico

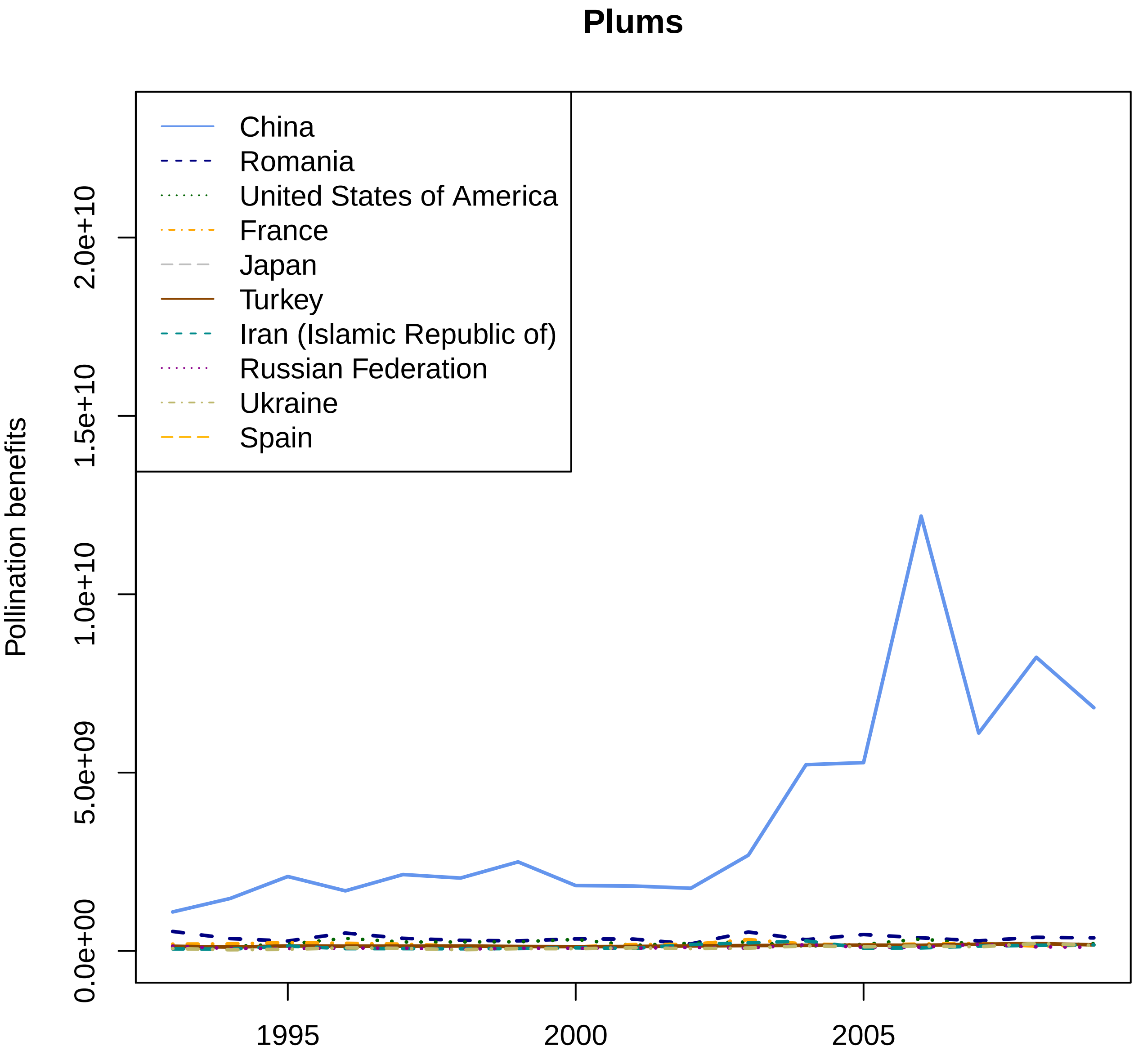

Plums
China
Romania
United States of America
France
Japan
Turkey
Iran (Islamic Republic of)
Russian Federation
Ukraine
Spain
Pollination benefits
0.0e+00
5.0e+09
1.0e+10
1.5e+10
2.0e+10
1995
2000
2005

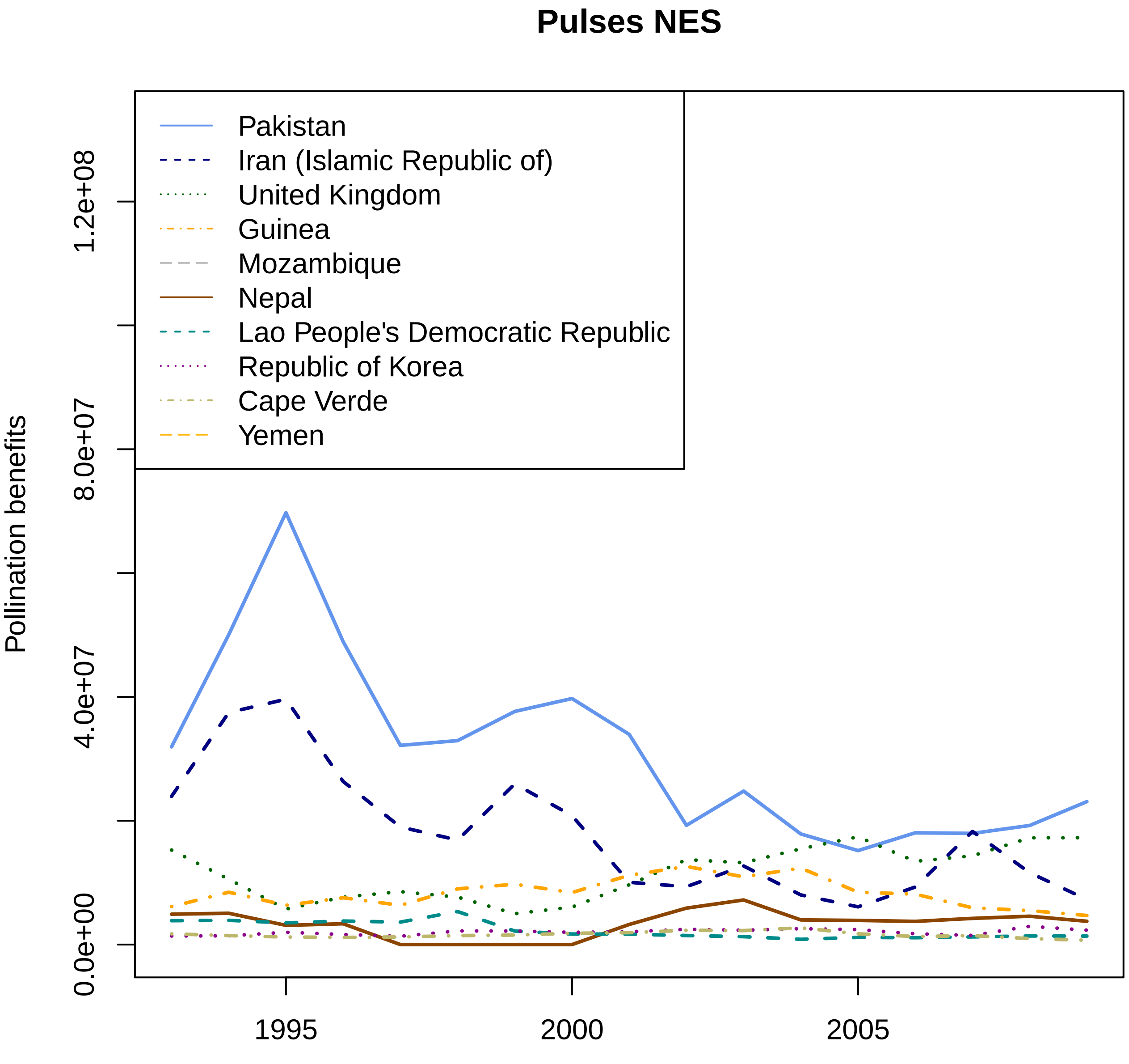
Pulses NES
Pakistan
Iran (Islamic Republic of)
United Kingdom
Guinea
Mozambique
Nepal
Lao People's Democratic Republic
Republic of Korea
Cape Verde
Yemen
Pollination benefits
0.0e+00
4.0e+07
8.0e+07
1.2e+08
1995
2000
2005

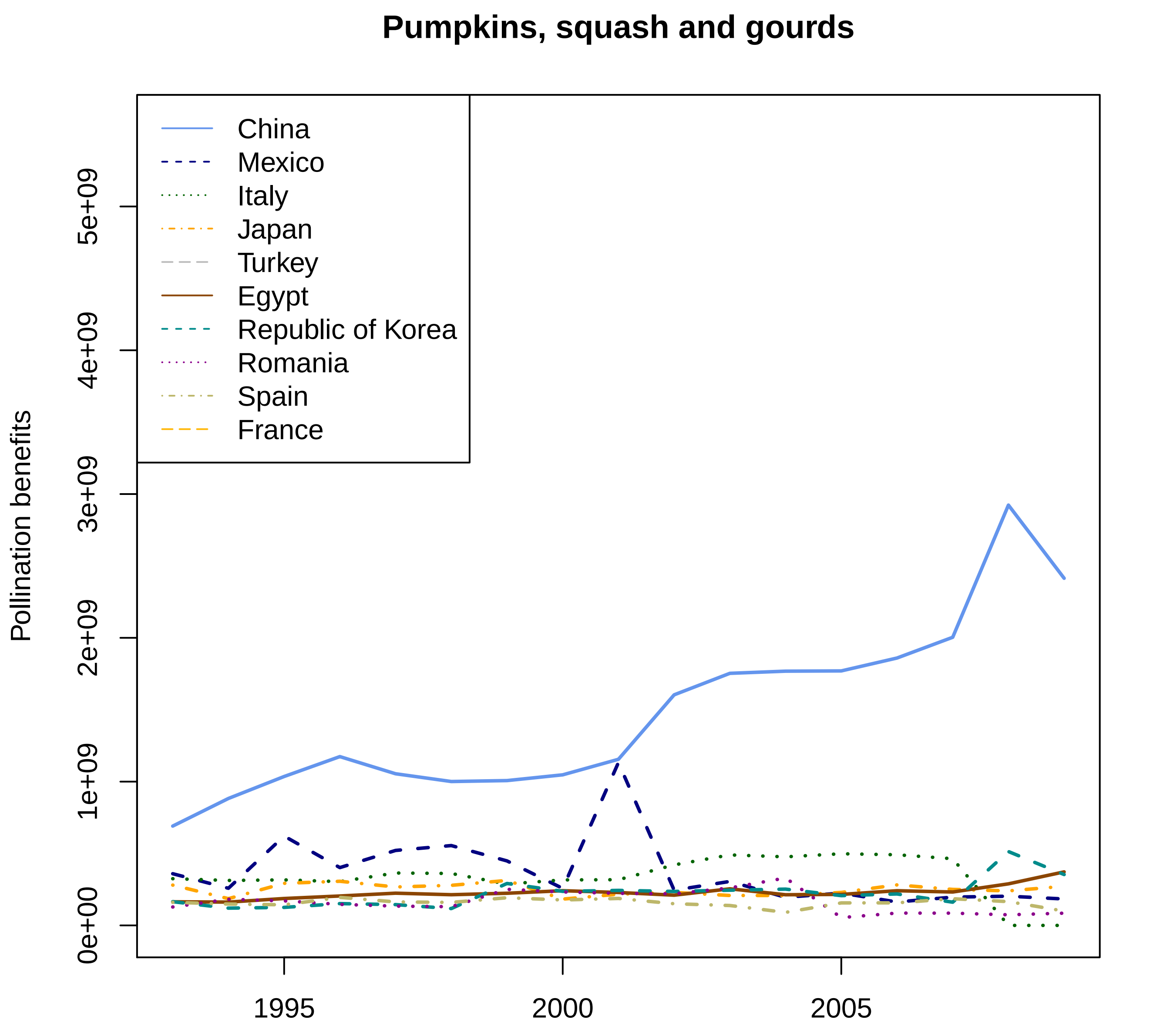
Pumpkins, squash and gourds
Pollination benefits
0e+00
1e+09
2e+09
3e+09
4e+09
5e+09
1995
2000
2005
China
Mexico
Italy
Japan
Turkey
Egypt
Republic of Korea
Romania
Spain
France

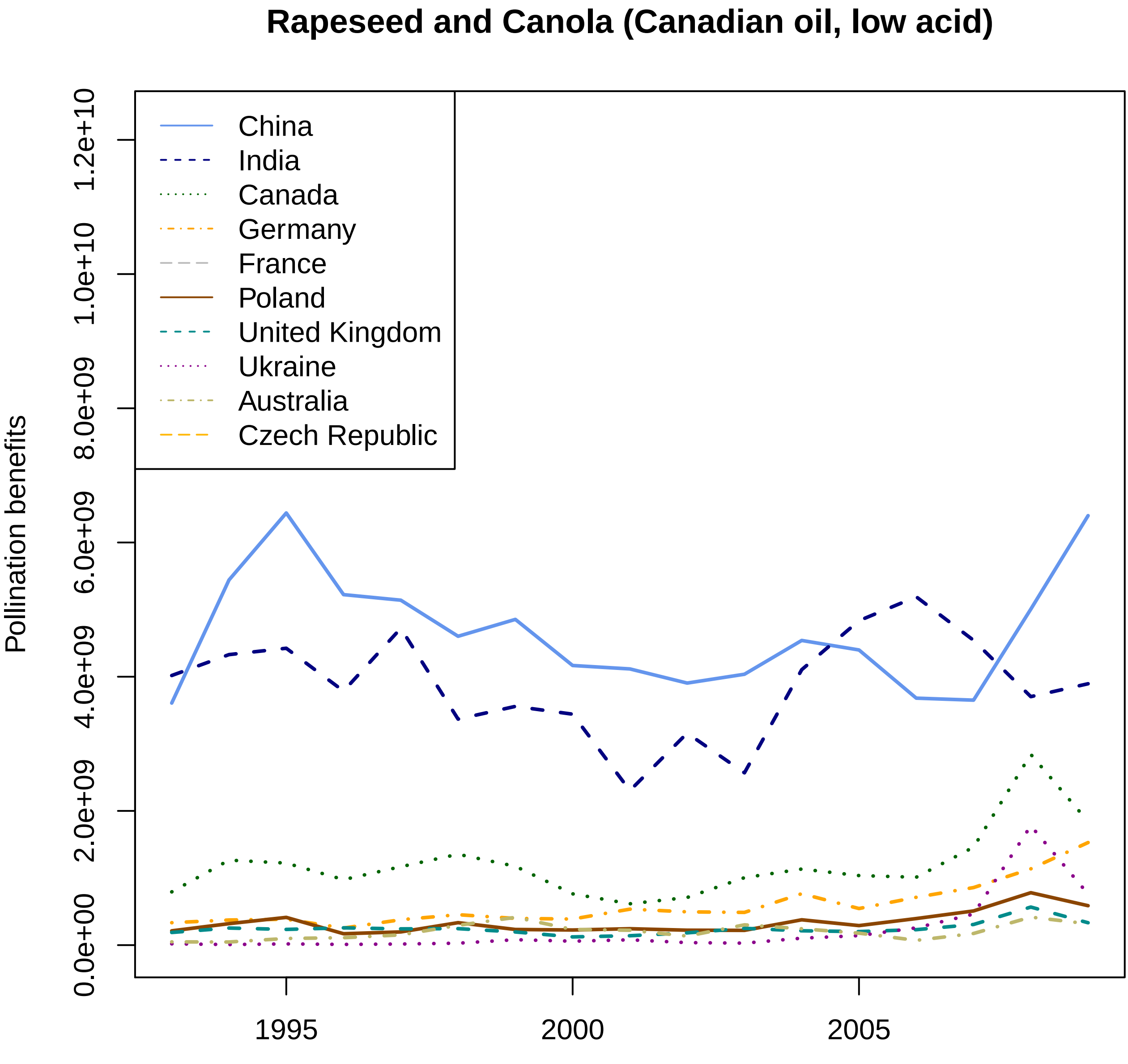

Rapeseed and Canola (Canadian oil, low acid)
Pollination benefits
0.0e+00
2.0e+09
4.0e+09
6.0e+09
8.0e+09
1.0e+10
1.2e+10
1995
2000
2005
China
India
Canada
Germany
France
Poland
United Kingdom
Ukraine
Australia
Czech Republic

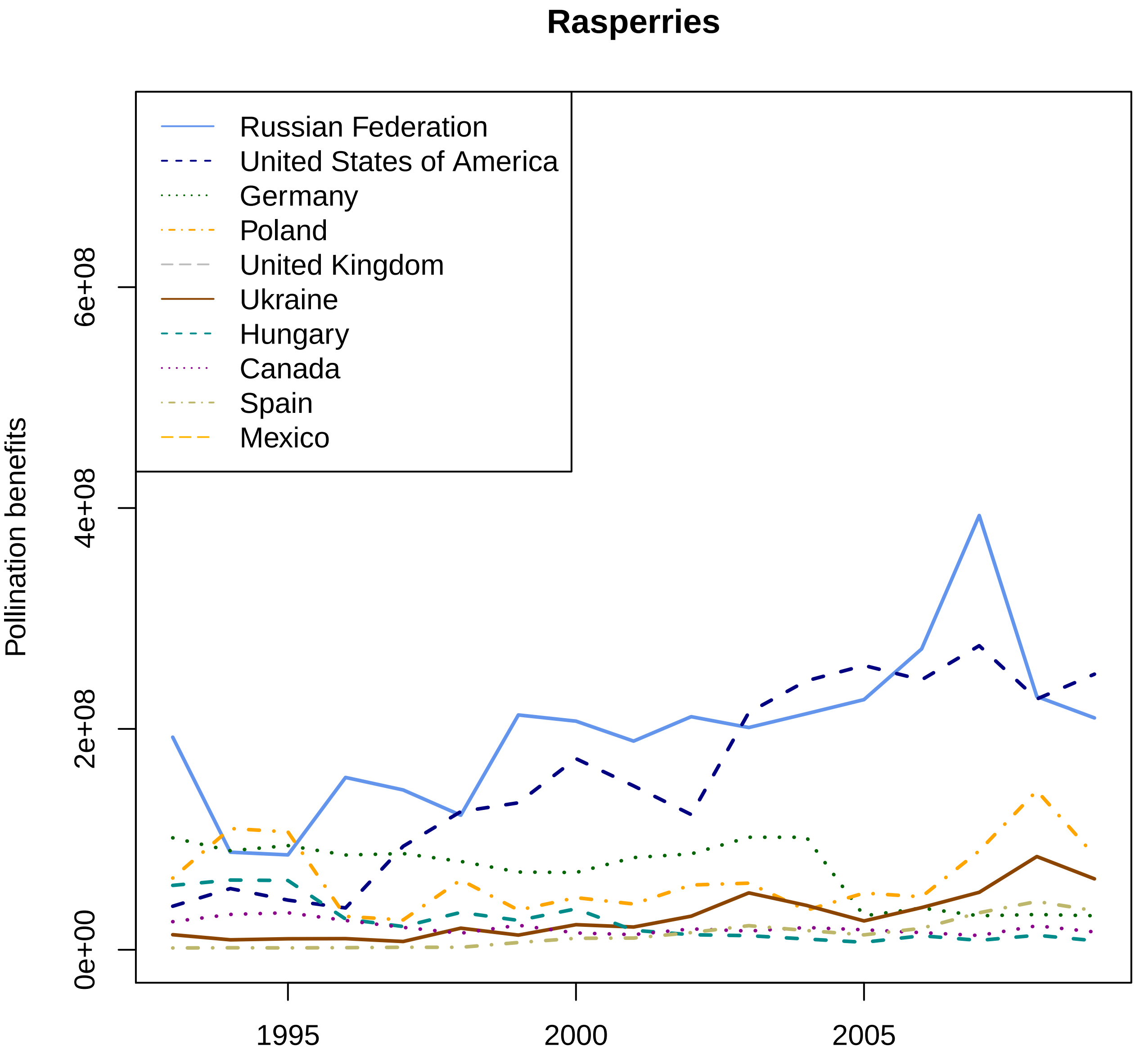
Rasperries
Russian Federation
United States of America
Germany
Poland
United Kingdom
Ukraine
Hungary
Canada
Spain
Mexico
Pollination benefits
0e+00
2e+08
4e+08
6e+08
1995
2000
2005

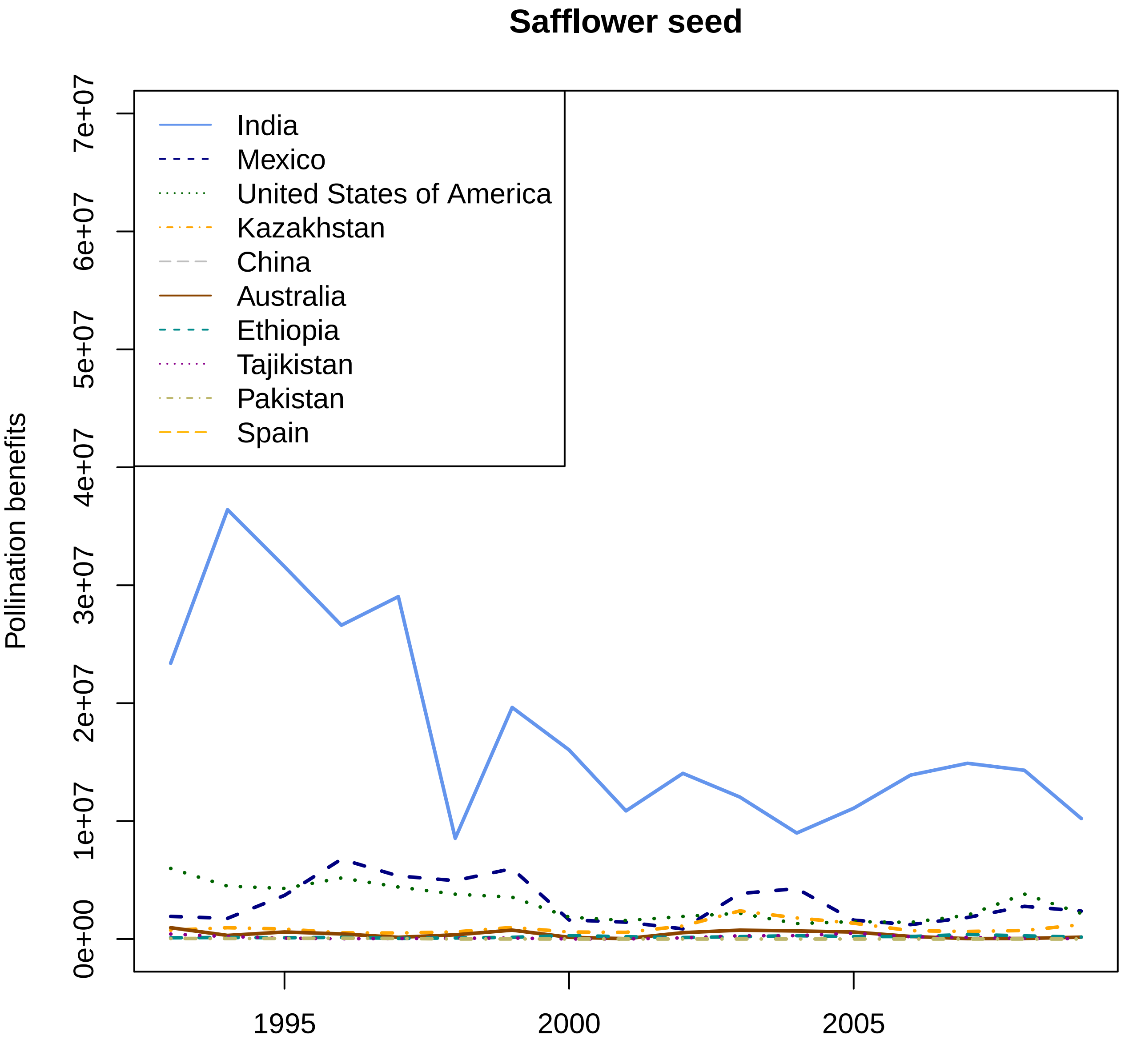

Safflower seed
Pollination benefits
0e+00
1e+07
2e+07
3e+07
4e+07
5e+07
6e+07
7e+07
1995
2000
2005
India
Mexico
United States of America
Kazakhstan
China
Australia
Ethiopia
Tajikistan
Pakistan
Spain

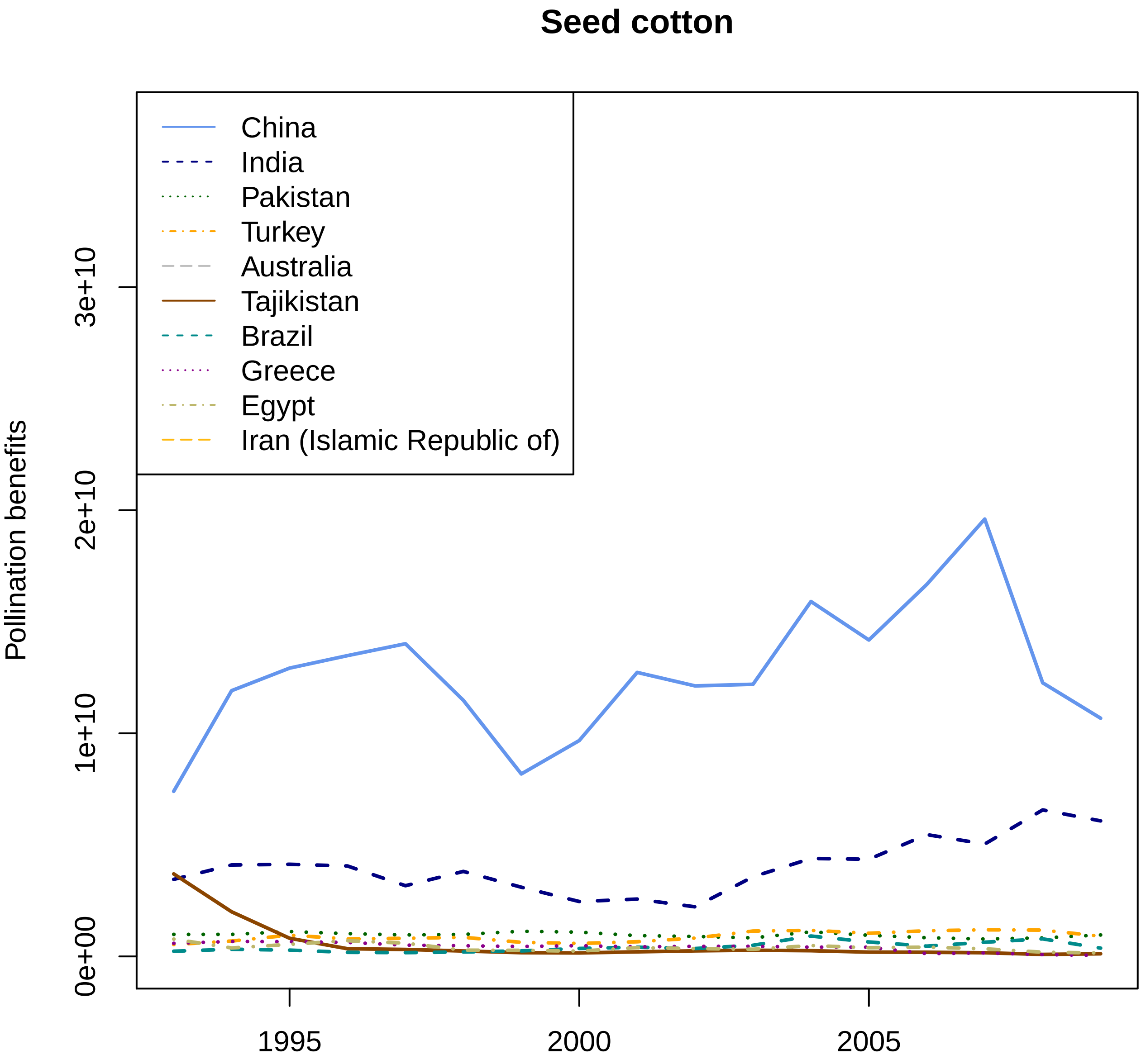
Seed cotton
China
India
Pakistan
Turkey
Australia
Tajikistan
Brazil
Greece
Egypt
Iran (Islamic Republic of)
Pollination benefits
0e+00
1e+10
2e+10
3e+10
1995
2000
2005

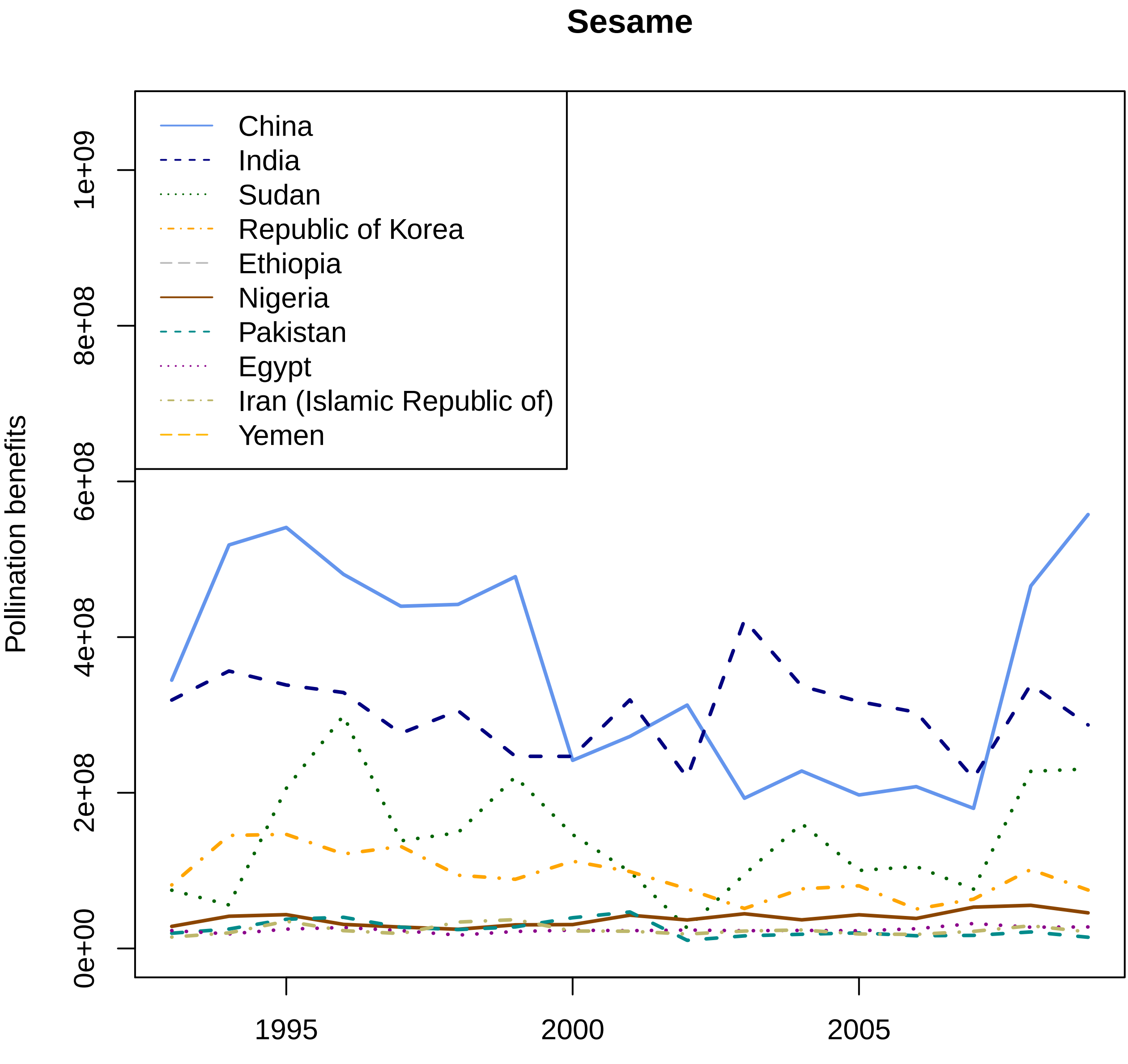

Sesame
Pollination benefits
0e+00
2e+08
4e+08
6e+08
8e+08
1e+09
1995
2000
2005
China
India
Sudan
Republic of Korea
Ethiopia
Nigeria
Pakistan
Egypt
Iran (Islamic Republic of)
Yemen

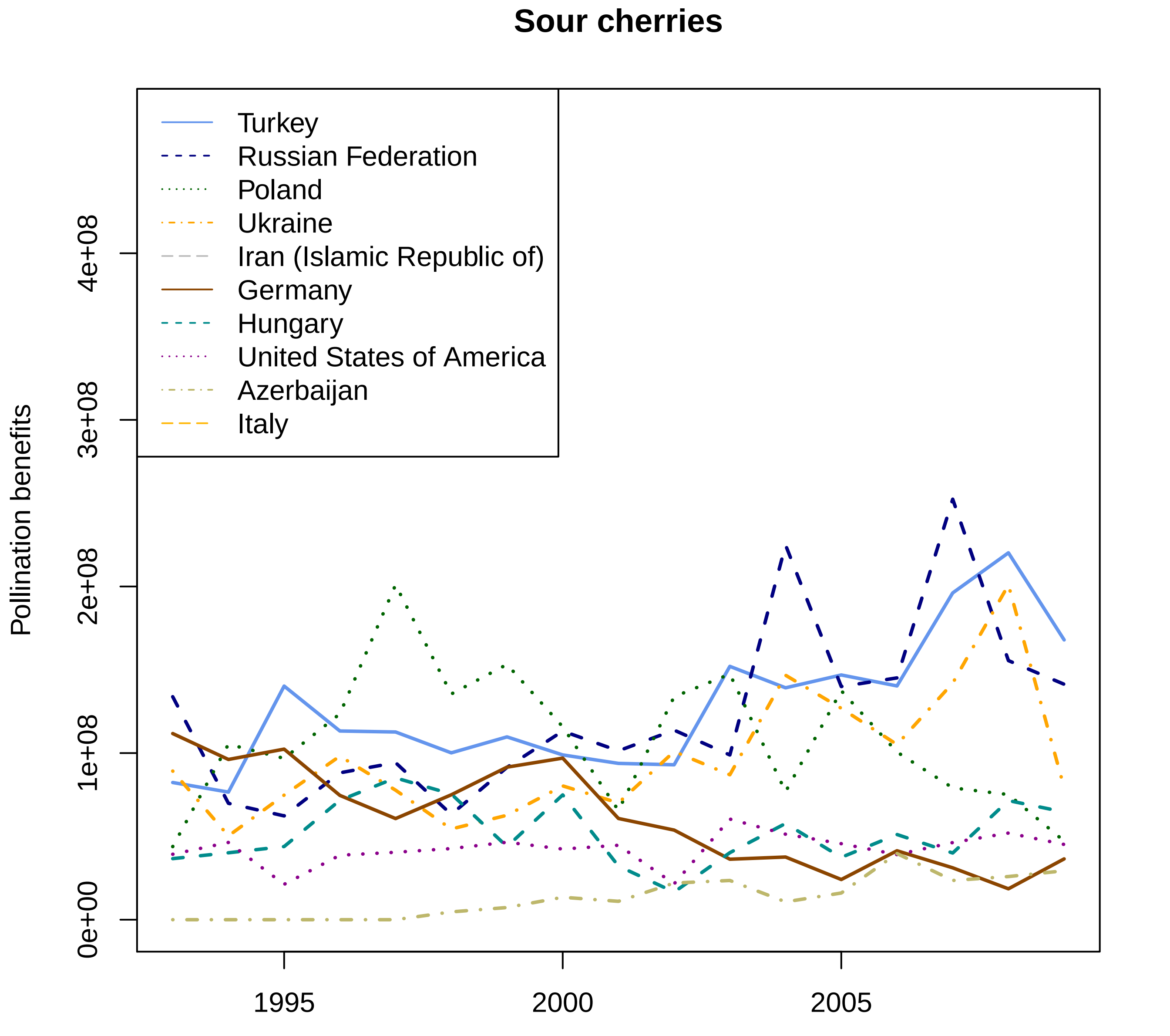
Sour cherries
Turkey
Russian Federation
Poland
Ukraine
Iran (Islamic Republic of)
Germany
Hungary
United States of America
Azerbaijan
Italy
Pollination benefits
0e+00
1e+08
2e+08
3e+08
4e+08
1995
2000
2005

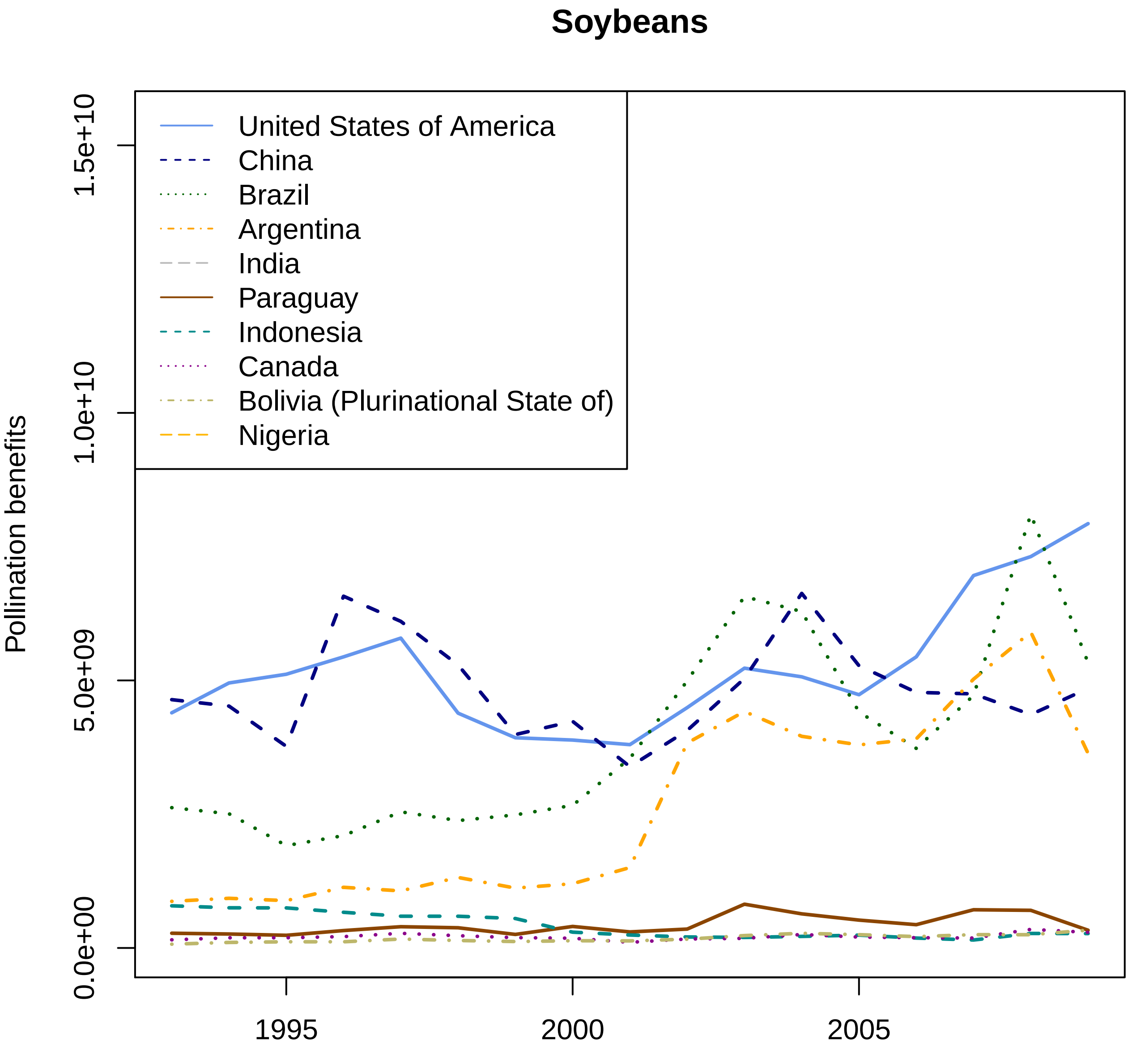

Soybeans
Pollination benefits
0.0e+00
5.0e+09
1.0e+10
1.5e+10
1995
2000
2005
United States of America
China
Brazil
Argentina
India
Paraguay
Indonesia
Canada
Bolivia (Plurinational State of)
Nigeria

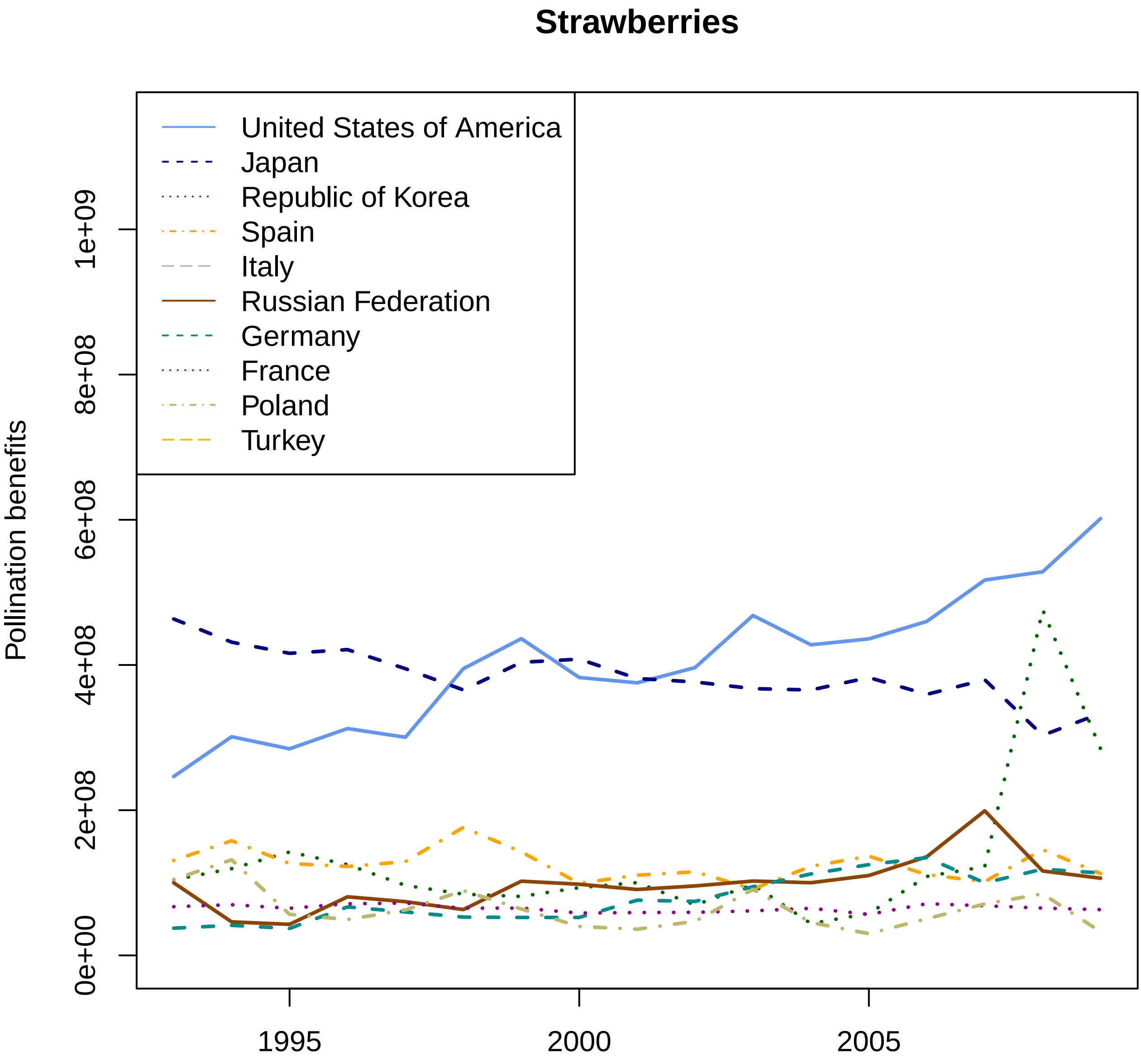
Strawberries
United States of America
Japan
Republic of Korea
Spain
Italy
Russian Federation
Germany
France
Poland
Turkey
Pollination benefits
0e+00
2e+08
4e+08
6e+08
8e+08
1e+09
1995
2000
2005

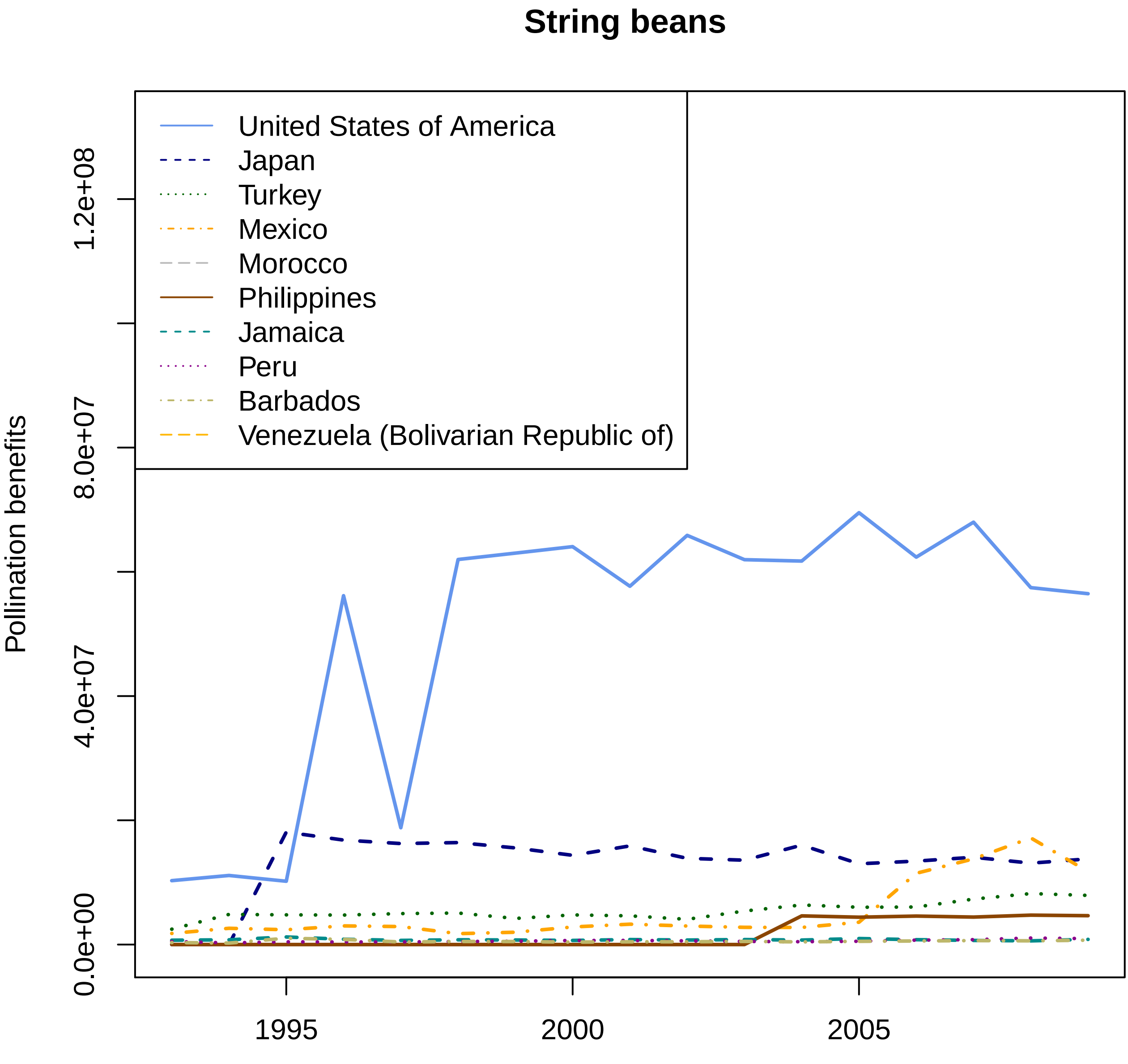
String beans
United States of America
Japan
Turkey
Mexico
Morocco
Philippines
Jamaica
Peru
Barbados
Venezuela (Bolivarian Republic of)
Pollination benefits
0.0e+00
4.0e+07
8.0e+07
1.2e+08
1995
2000
2005

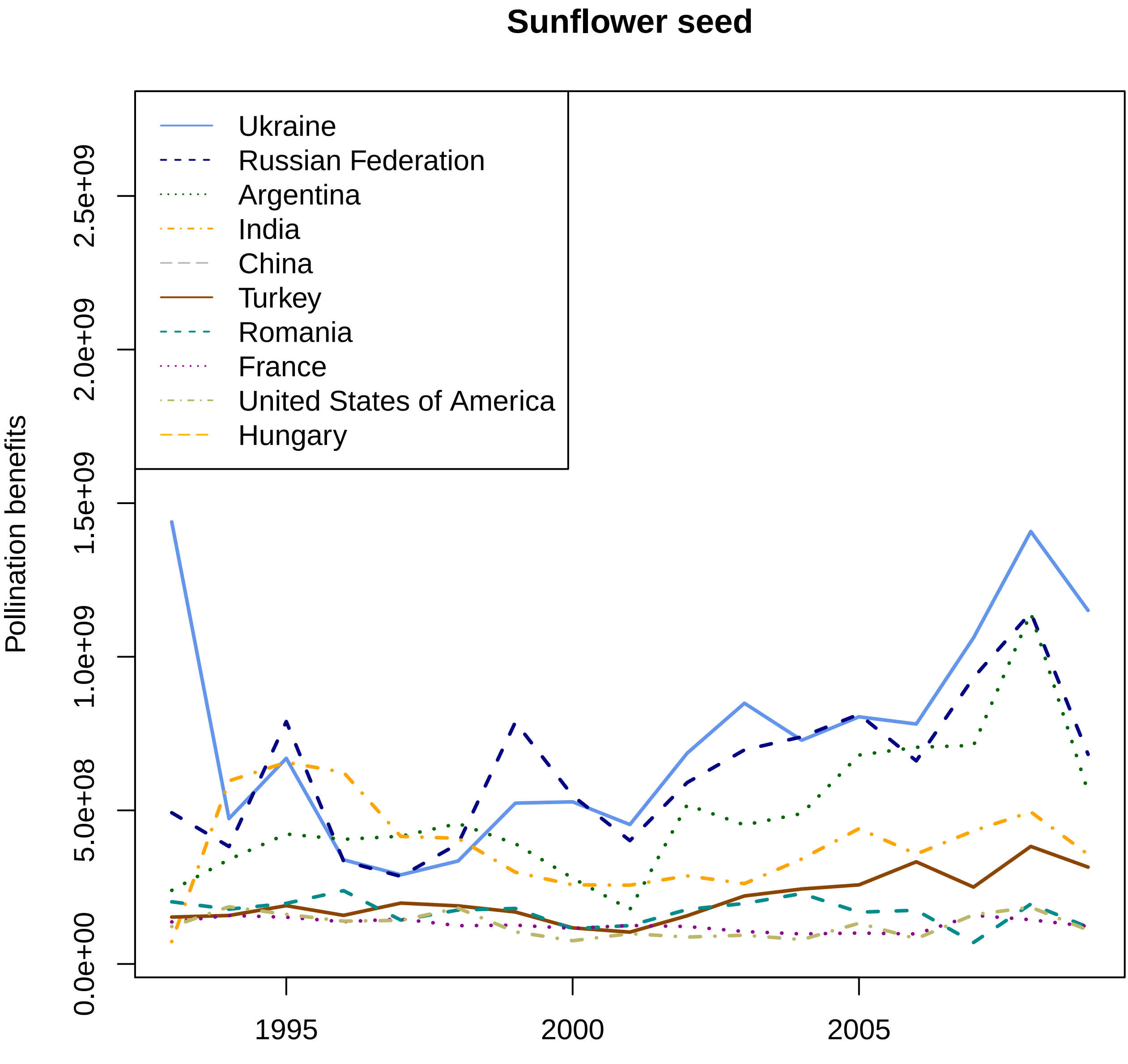

Sunflower seed
Ukraine
Russian Federation
Argentina
India
China
Turkey
Romania
France
United States of America
Hungary
Pollination benefits
0.0e+00
5.0e+08
1.0e+09
1.5e+09
2.0e+09
2.5e+09
1995
2000
2005

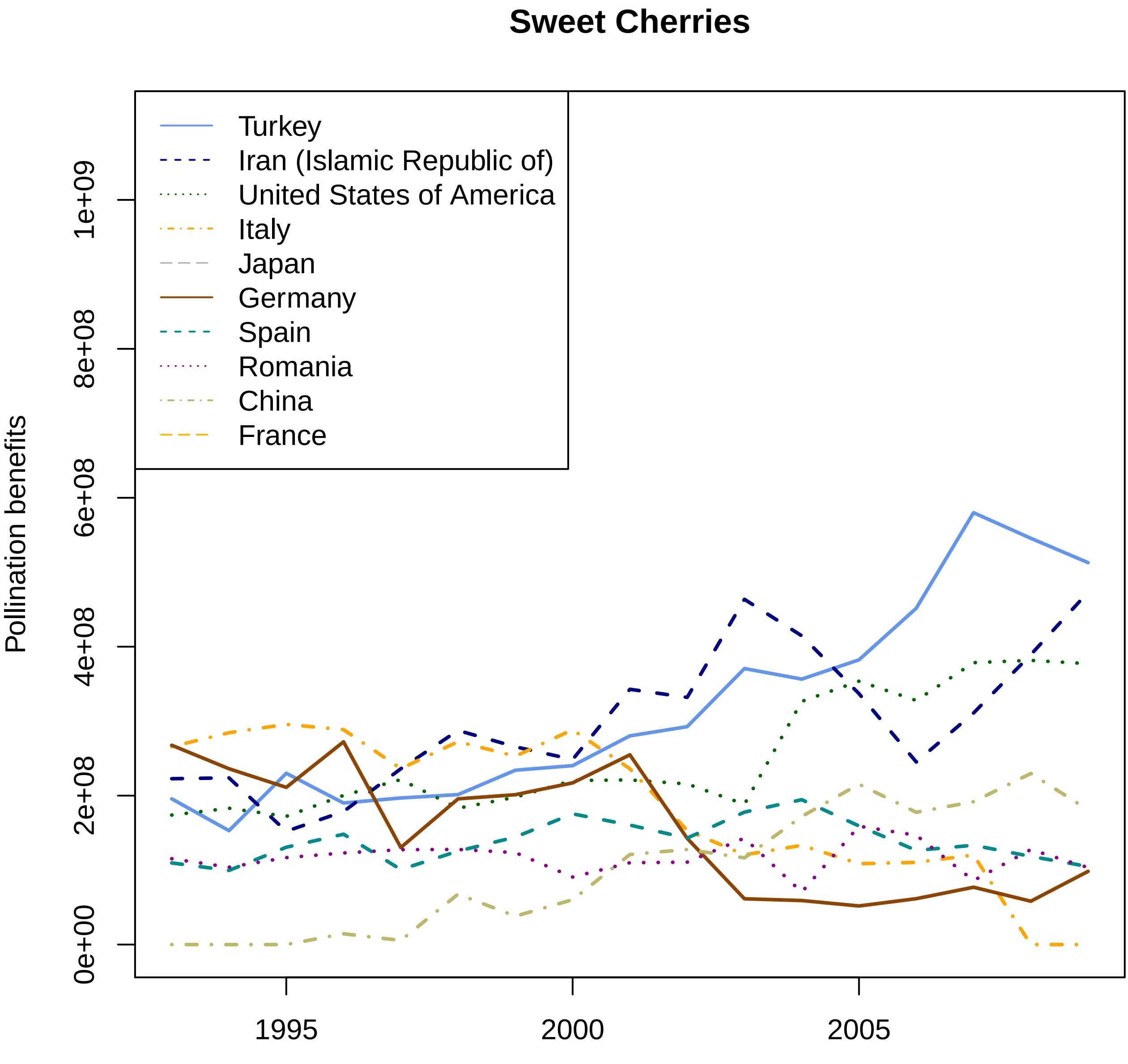
Sweet Cherries
Turkey
Iran (Islamic Republic of)
United States of America
Italy
Japan
Germany
Spain
Romania
China
France
Pollination benefits
0e+00
2e+08
4e+08
6e+08
8e+08
1e+09
1995
2000
2005

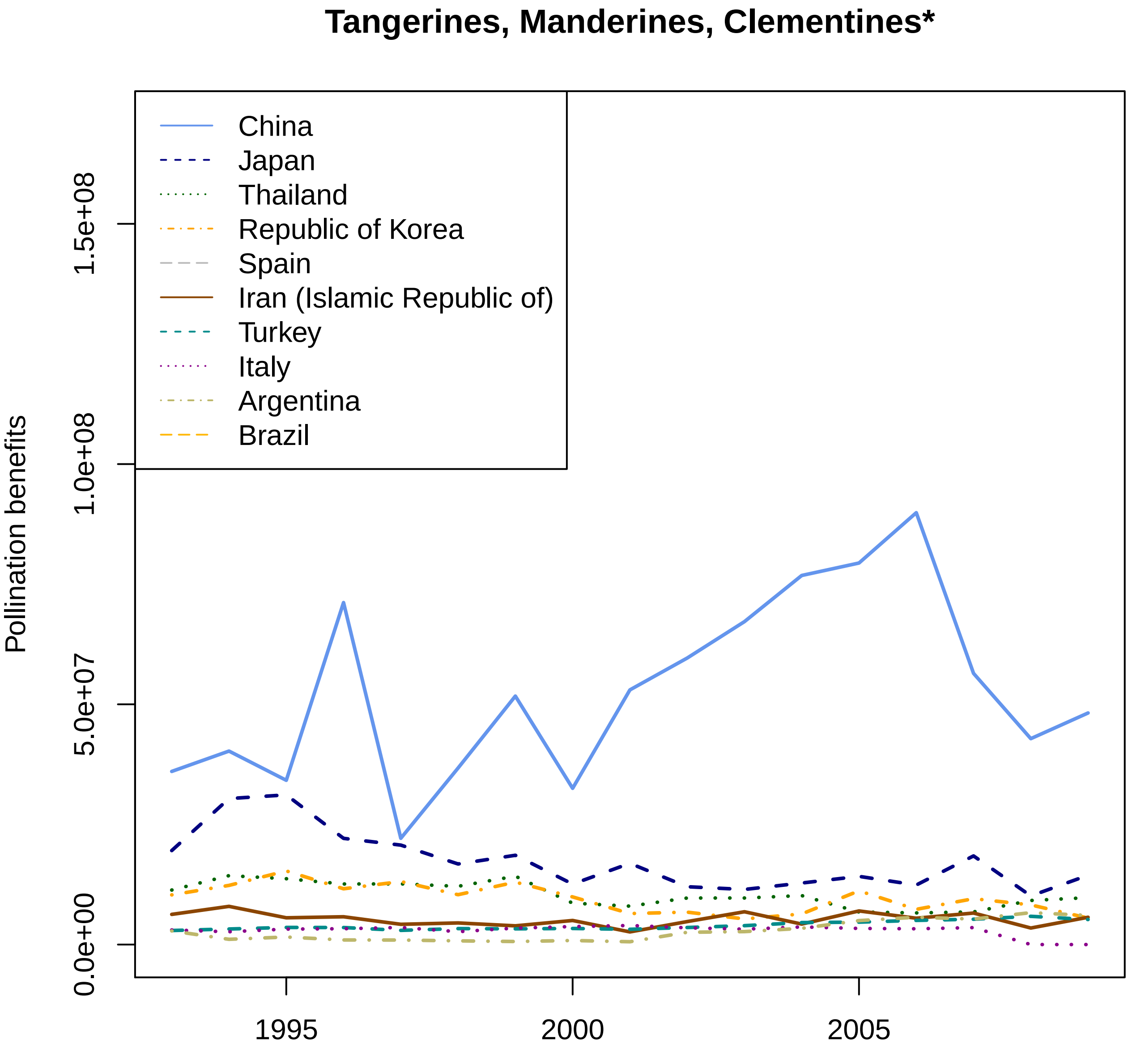
Tangerines, Manderines, Clementines*
China
Japan
Thailand
Republic of Korea
Spain
Iran (Islamic Republic of)
Turkey
Italy
Argentina
Brazil
Pollination benefits
0.0e+00
5.0e+07
1.0e+08
1.5e+08
1995
2000
2005

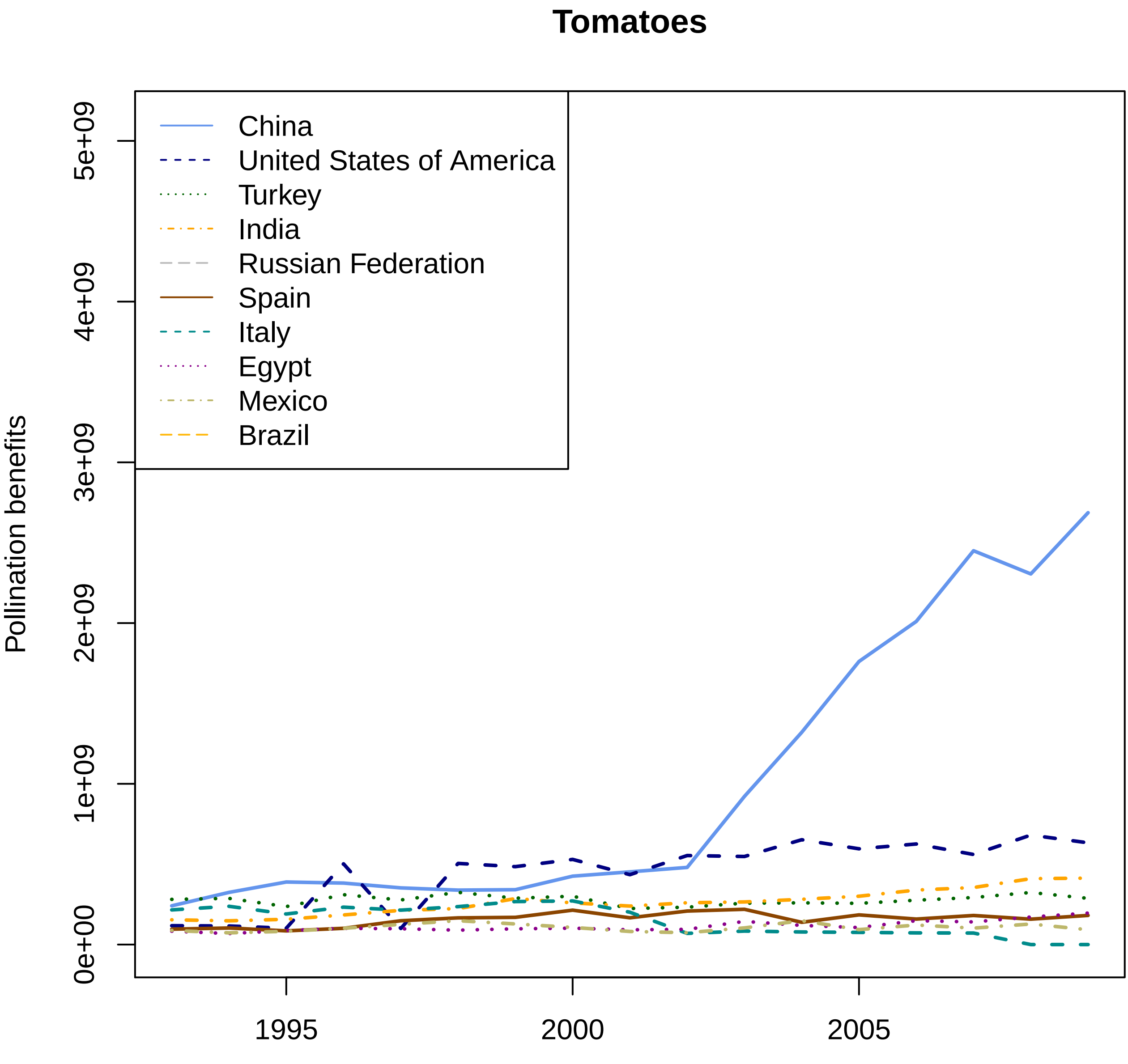

Tomatoes
China
United States of America
Turkey
India
Russian Federation
Spain
Italy
Egypt
Mexico
Brazil
Pollination benefits
0e+00
1e+09
2e+09
3e+09
4e+09
5e+09
1995
2000
2005

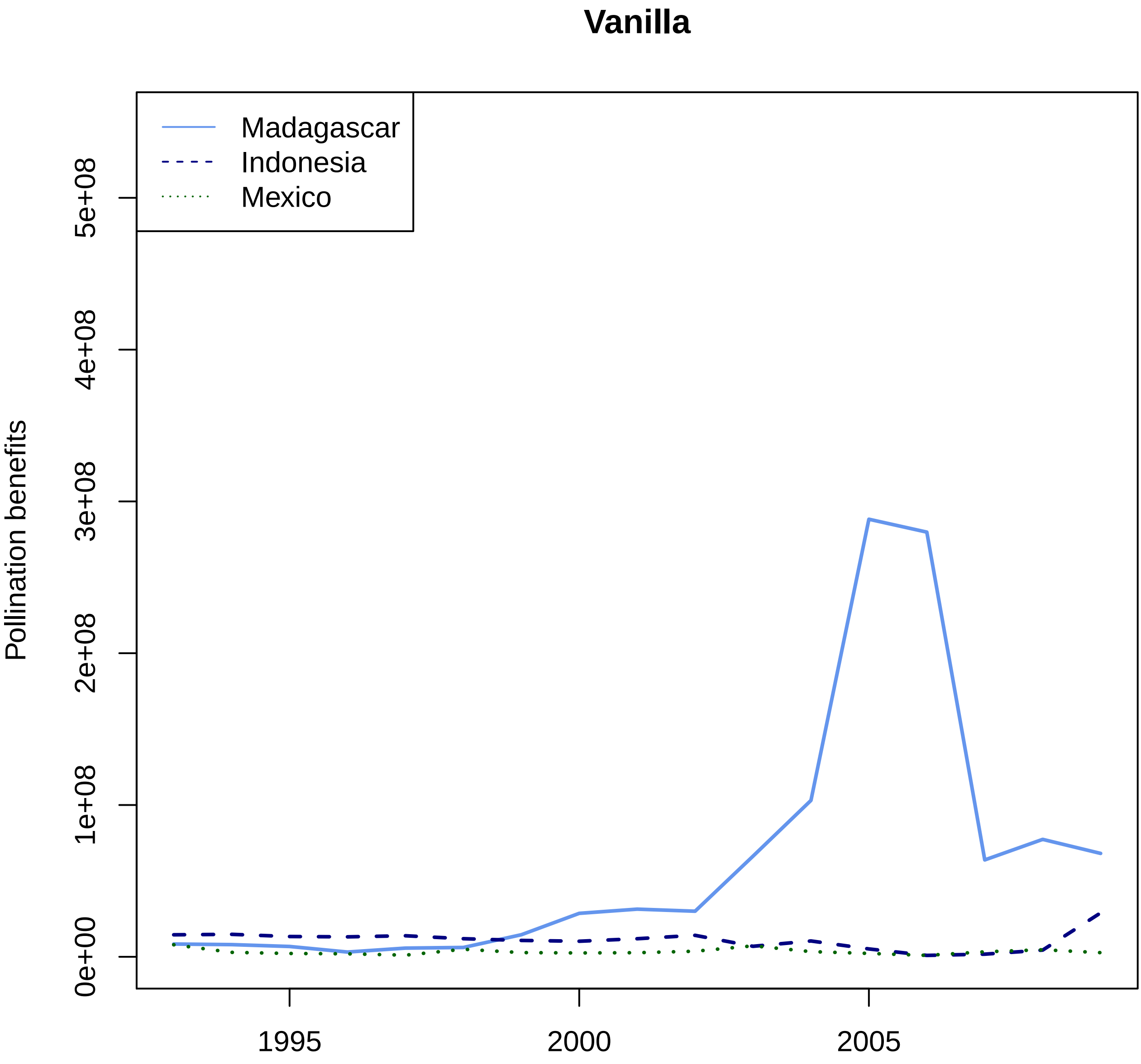
Vanilla
Madagascar
Indonesia
Mexico
Pollination benefits
0e+00
1e+08
2e+08
3e+08
4e+08
5e+08
1995
2000
2005

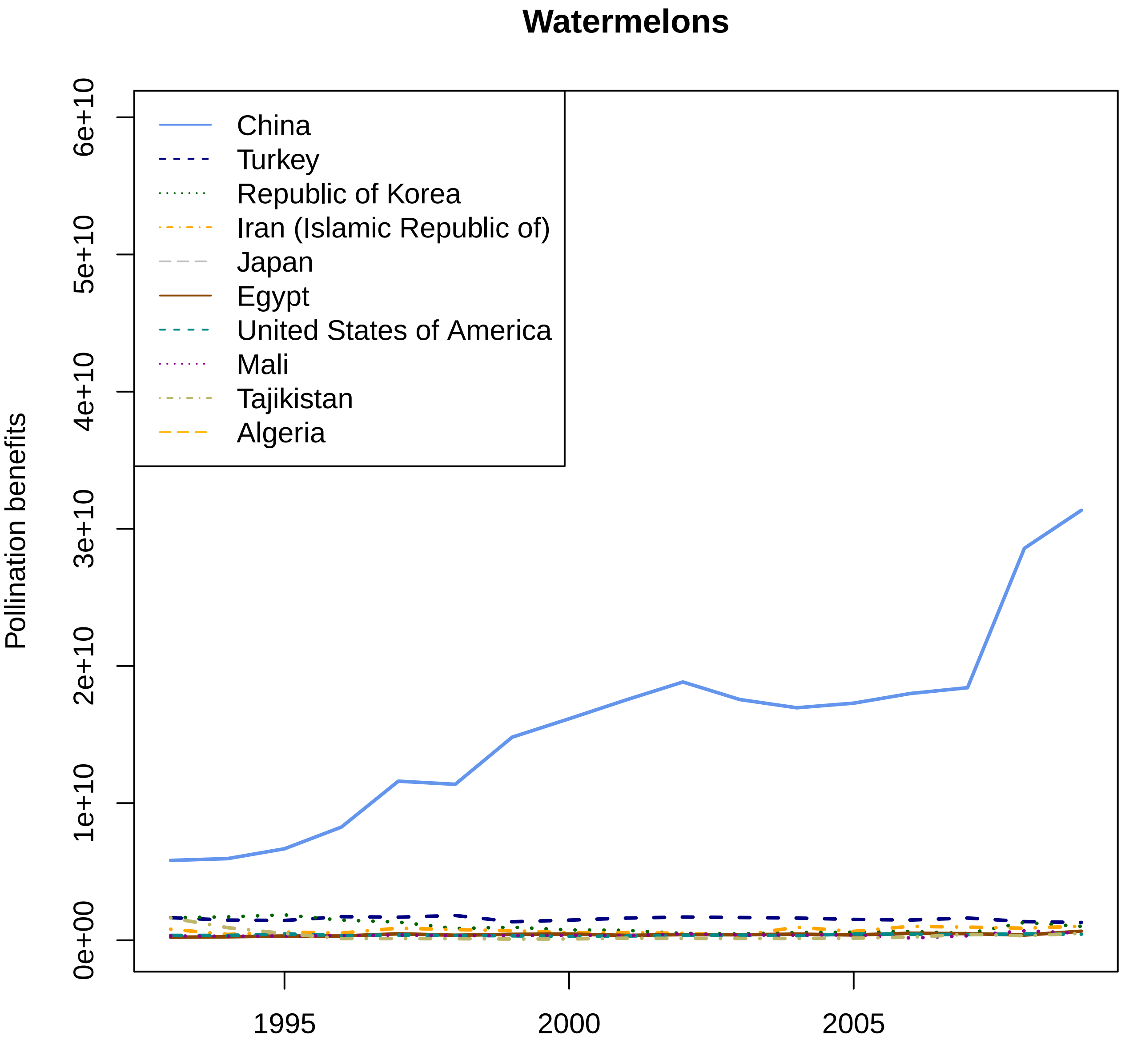
Watermelons
Pollination benefits
0e+00
1e+10
2e+10
3e+10
4e+10
5e+10
6e+10
1995
2000
2005
China
Turkey
Republic of Korea
Iran (Islamic Republic of)
Japan
Egypt
United States of America
Mali
Tajikistan
Algeria
